# Diterpene Biosynthesis from Geranylgeranyl Diphosphate Analogues with Changed Reactivities Expands Skeletal Diversity

**DOI:** 10.1002/anie.202211054

**Published:** 2022-09-21

**Authors:** Heng Li, Jeroen S. Dickschat

**Affiliations:** ^1^ Kekulé-Institute for Organic Chemistry and Biochemistry University of Bonn Gerhard-Domagk-Straße 1 53121 Bonn Germany

**Keywords:** Configuration Determination, Enzymes, Isotopes, Substrate Analogues, Terpenoids

## Abstract

Two analogues of the diterpene precursor geranylgeranyl diphosphate with shifted double bonds, named iso‐GGPP I and iso‐GGPP II, were enzymatically converted with twelve diterpene synthases from bacteria, fungi and protists. The changed reactivity in the substrate analogues resulted in the formation of 28 new diterpenes, many of which exhibit novel skeletons.

## Graphical abstract

Two analogues of geranylgeranyl diphosphate with shifted double bond were enzymatically converted with twelve diterpene synthases. The double bond shift causes a change of reactivity that results in the formation of many diterpenes with novel skeletons. A total number of 28 new diterpenes is reported and their mechanism of formation is discussed.

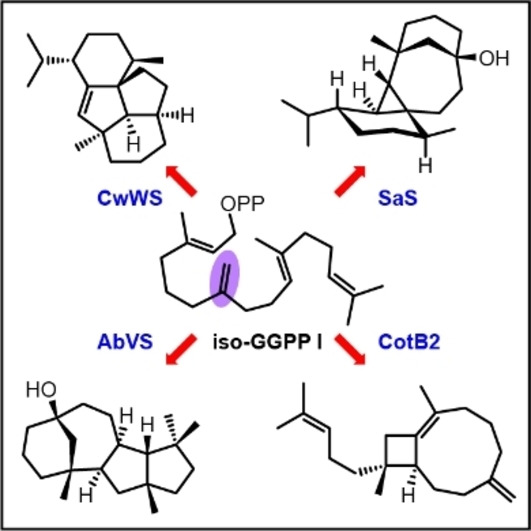

## Introduction

The astonishing structural complexity observed in terpenes is generated from very few acyclic and achiral precursors. Only two C_5_ building blocks, dimethylallyl diphosphate (DMAPP) and isopentenyl diphosphate (IPP), are required that are made via the mevalonate or the deoxyxylulose phosphate pathway.^[1]^ While DMAPP is an electrophile, IPP can react as a nucleophile, allowing their fusion to oligoprenyl diphosphates by prenyltransferases. Starting from DMAPP, successive elongation reactions with IPP first lead to the monoterpene precursor geranyl diphosphate (GPP, C_10_),^[2]^ and then to farnesyl diphosphate (FPP, C_15_)^[3]^ as the precursor to sesquiterpenes, the diterpene precursor geranylgeranyl diphosphate (GGPP, C_20_),^[4]^ geranylfarnesyl diphosphate (GFPP, C_25_)^[5]^ for sesterterpene biosynthesis, and—as recently discovered—even farnesylfarnesyl diphosphate for non‐squalene derived triterpene biosynthesis.^[6]^ Class I terpene synthases (TSs)^[7]^ exhibit several highly conserved residues and motifs, including the aspartate‐rich region (DDXX(X)D)^[7]^ and the NSE triad (ND(L,I,V)XSXX(K,R)E)^[8]^ for binding of a trinuclear (Mg^2+^)_3_ cluster that in turn binds to the substrate's diphosphate. The pyrophosphate sensor,^[9]^ a highly conserved Arg, and the RY pair form hydrogen bridges to the diphosphate.^[10]^ With assistance of an effector triad substrate ionisation by the abstraction of diphosphate is achieved,^[9]^ which initiates a cationic cascade reaction with cyclisations, hydride or proton migrations, and Wagner–Meerwein rearrangements. Termination of the cascade by deprotonation or water quenching ultimately leads to terpene hydrocarbons or alcohols. Further roles of the enzyme include to provide a hydrophobic pocket that defines the reactive substrate conformation,^[11]^ to stabilise cationic intermediates, and to participate in acid base catalysis for which a carbonyl oxygen at the helix G break is responsible in selinadiene biosynthesis.^[12]^

Interference with terpene biosynthesis aiming at novel TS products is possible in two ways: Either the enzyme can be modified through site‐directed mutagenesis,^[13]^ or structurally modified substrate analogues can be used in enzymatic conversions.^[14]^ Recent studies have shown that many TSs accept and convert non‐natural substrate analogues such as halogenated oligoprenyl diphosphates,^[15]^ allyl alcohols and epoxides,^[16]^ ketones,^[17]^ derivatives with heteroatom insertions into the chain,^[18]^ with changed Me group substitutions,[^15d^, ^17b^, ^19^] double bond shifts,[^17^, ^20^] *Z*‐configured or hydrogenated double bonds.[^17a^, ^21^] Most of these studies have used FPP analogues in conjunction with sesquiterpene synthases (STSs), but only in a few cases GGPP analogues were converted with diterpene synthases (DTSs), exemplified by previous work using taxadiene synthase[^15a^, ^21a^] and casbene synthase^[20]^ from plants, and the bacterial catenul‐14‐en‐6‐ol synthase from *Catenulispora acidiphila*.^[21b]^ The often intriguing cyclisation mechanisms of DTSs^[22]^ prompted us to broadly investigate the DTSs recently characterised in our group for their potential to convert GGPP analogues into non‐natural diterpenes. The natural products of the terpene synthases used in this study and their cyclisation mechanisms are summarised in Schemes S1–S12.

## Results and Discussion

**Scheme 1:**
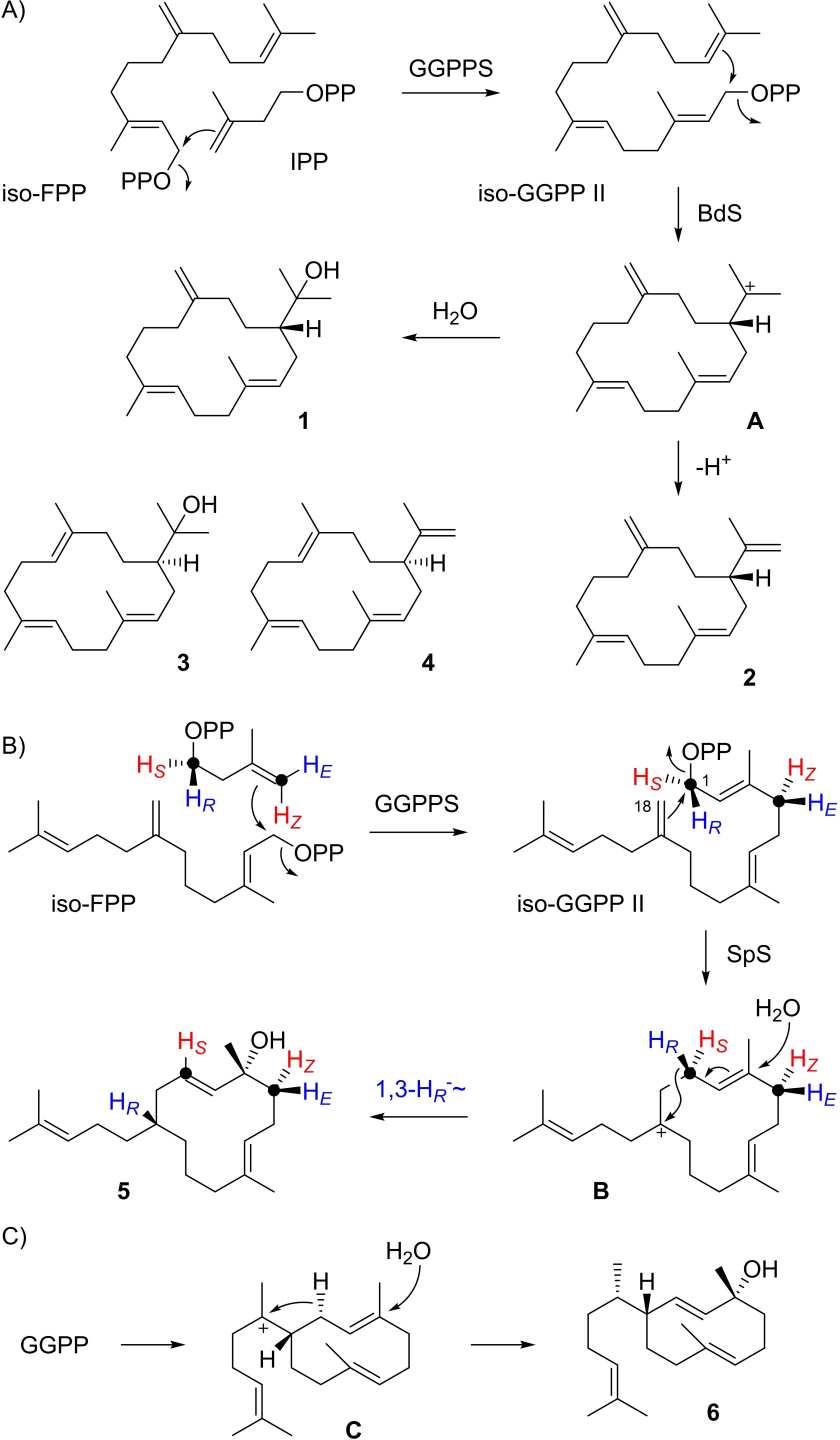
Diterpenes from iso‐FPP. A) Formation of iso‐GGPP II from iso‐FPP and IPP by GGPPS and conversion into **1** and **2** by BdS, B) biosynthesis of **5** using SpS, and C) cyclisation of GGPP to the related natural product **6**. Red and blue hydrogens are substituted with deuterium in the stereoselectively deuterated probes (*R*)‐ and (*S*)‐(1‐^13^C,1‐^2^H)IPP, and (*E*)‐ and (*Z*)‐(4‐^13^C,4‐^2^H)IPP for determination of absolute configurations. Black dots represent ^13^C‐labelled carbons in these probes.

The incubation of iso‐FPP^[20]^ in which the C6=C7 double bond is shifted to a C7=C14 double bond with IPP, GGPP synthase (GGPPS) from *Streptomyces cyaneofuscatus* ^[23]^ and bonnadiene synthase (BdS) from *Allokutzneria albata* ^[24]^ resulted in the formation of a diterpene alcohol and minor amounts of a diterpene hydrocarbon (Figure S1). Both compounds were isolated and structurally characterised by NMR spectroscopy (Tables S2 and S3, Figures S2–S17), revealing macrocyclic structures for which we suggest the names isonephthenol (**1**) and isocembrene A (**2**, Scheme 1A). Generally, compounds obtained in this study in which the shifted double bond of the substrate analogue is reflected, will be named iso‐compounds of the corresponding natural product. Specifically, **1** and **2** are the iso‐compounds corresponding to nephthenol (**3**) and cembrene A (**4**). With GGPP the BdS‐catalysed reaction to bonnadiene starts with an isomerisation to geranyllinalyl diphosphate (GLPP) and subsequent 1,14‐cyclisation to a 2*Z* configured macrocycle, followed by further cyclisation steps (Scheme S1). The structural modification in iso‐GGPP II, formed from iso‐FPP and IPP by GGPPS, leads to a disturbed enzyme‐substrate interaction in which the 1,14‐cyclisation is still promoted, but with retained 2*E* configuration in **A**, while the downstream steps of the cascade are interrupted and direct formation of **1** and **2** is observed.

The enzymatic conversion of iso‐FPP and IPP with GGPPS and spata‐13,17‐diene synthase (SpS) from *S. xinhaiensis* ^[25]^ gave a complex product mixture (Figure S18) from which a diterpene alcohol **5** was isolated (Table S4, Figures S19–S26). With GGPP as substrate SpS catalyses an initial 1,10‐cyclisation (Scheme S2), which is not possible with iso‐GGPP II. Remarkably, a very similar 1,18‐cyclisation to **B** substitutes for the natural cyclisation mode. A subsequent 1,3‐hydride shift and attack of water result in **5** (Scheme 1B). Compound **5** was named pseudoobscuronatin, because its formation follows a very similar mechanism as for obscuronatin (**6**)^[26]^ that proceeds by 1,10‐cyclisation of GGPP to **C**, followed by 1,3‐hydride shift and attack of water (Scheme 1C).^[21b]^ Compounds isolated in this study will be named pseudo‐derivatives of known natural products with a similar cyclisation cascade, if this cascade is logically modified because of the double bond shift in the substrate analogue.

**Scheme 2:**
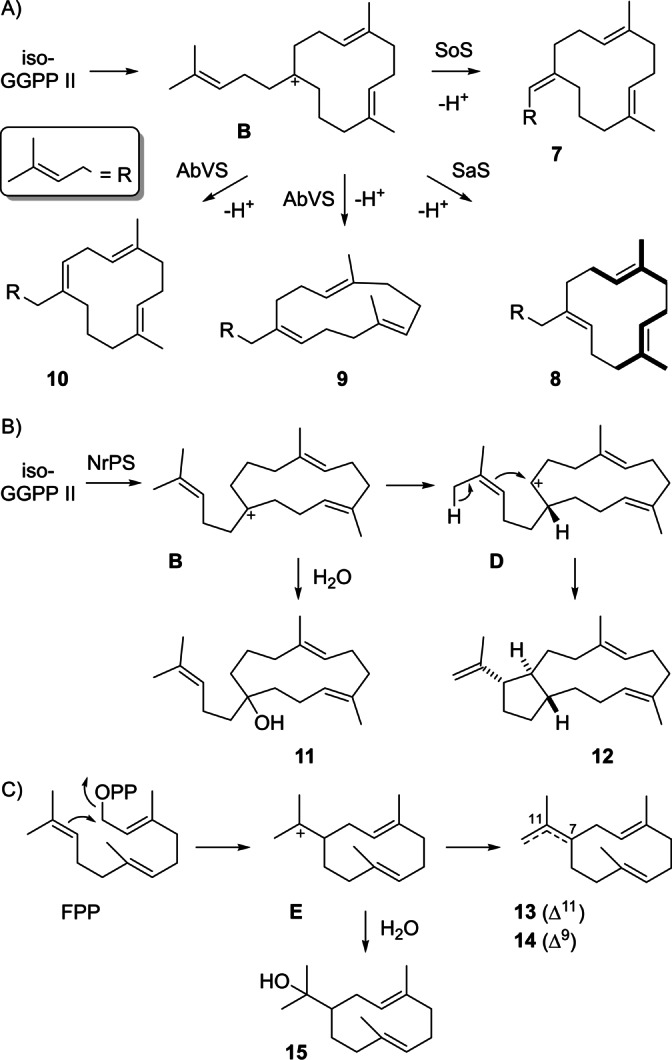
Diterpenes from iso‐FPP. A) Formation of **7** by SoS, **8** by SaS, and **9** and **10** by AbVS, B) formation of **11** and **12** by NrPS, and C) biosynthesis of related sesquiterpenes **13**–**15**.

With GGPPS and spinodiene synthase (SoS) from *Saccharopolyspora spinosa* ^[27]^ the substrates iso‐FPP and IPP were efficiently converted into one main diterpene, besides traces of a few side products (Figure S27). The purified main compound showed no optical rotation, suggesting an achiral product, which was confirmed by NMR spectroscopy (Table S5, Figures S28–S35), resulting in the structure of **7** (Scheme 2A). The natural spinodiene cyclisation cascade is initiated with a 1,11–10,14 cyclisation (Scheme S3), which is altered with iso‐GGPP II to the similar 1,18‐cyclisation to **B**. Herein, the second ring closure is not realised, because a tertiary cationic intermediate could only be reached through formation of a strained cyclobutane ring. Consequently, **B** simply reacts by deprotonation to **7**. Because of the similarities between the cyclisation reactions to **7** and to germacrene A (**13**, Scheme 2C), compound **7** was named prenylpseudogermacrene A.

With FPP as substrate alternative reactions from intermediate **E** include the deprotonation to germacrene B (**14**) or quenching with water to hedycaryol (**15**, Scheme 2C). Analogous reactions were observed for the conversion of iso‐GGPP II with other diterpene synthases. *A. albata* spiroalbatene synthase (SaS)^[28]^ resulted in the highly selective formation (Figure S36) of the new compound prenylpseudogermacrene B (**8**, Table S6, Figures S37–S44). The pseudo‐*C*_3_ symmetry of the macrocycle in **8** resulted in almost identical chemical shifts for carbons in corresponding positions of the two isoprene units marked in bold. An unambiguous assignment was possible using iso‐FPP in conjunction with the five isotopomers of singly labelled (^13^C)IPP and product analysis through ^13^C NMR (Figure S45). With GGPP SaS shows an initial 1,14‐cyclisation (Scheme S4) that is surprisingly turned into a 1,18‐cyclisation with iso‐GGPP II, although the C14=C15 double bond remains unchanged in this substrate analogue. A possible explanation lies in the second ring closure in spiroalbatene biosynthesis that happens between C1 and C10, suggesting that the C10=C11 double bond of GGPP is also close to C1 in the active site of SaS. Therefore, only a small conformational change for iso‐GGPP II may lead to the observed altered cyclisation mode.

Further alternative deprotonation products from cation **B** were obtained by conversion of iso‐GGPP II with variediene synthase from *Aspergillus brasiliensis* (AbVS, Scheme 2A, Figure S46).^[29]^ This bifunctional fungal enzyme contains a prenyltransferase (PT) domain for the biosynthesis of GGPP and a TS domain for its cyclisation. Therefore, the substrate iso‐GGPP II can be formed in situ from iso‐FPP and IPP without addition of GGPPS. Through this approach the two compounds prenylpseudogermacrene C (**9**) and prenylpseudogermacrene D (**10**) were obtained (Tables S7 and S8, Figures S47–S62). With phomopsene synthase from *Nocardiopsis rhamnosiphilia* (NrPS,^[30]^ Scheme 2B) iso‐GGPP II gave besides the main product **8** (Figure S63) smaller amounts of **7**, **9** and **10**, and the new diterpene alcohol prenylpseudohedycaryol (**11**, Table S9, Figures S64–S71). Notably, as observed for SoS also AbVS and NrPS change the natural initial 1,11–10,14 cyclisation with GGPP (Schemes S5 and S6) to a 1,18‐cyclisation with iso‐GGPP II. Another minor product obtained from this enzyme was characterised as pseudodollabella‐3,7,18‐triene (**12**, Table S10, Figures S72–S79). This compound requires a 1,2‐hydride shift to **D**, cyclisation and deprotonation.

Different patterns of compounds **1**, **2** and **5**–**12** were also observed among the products obtained from iso‐GGPP II with several other bacterial DTSs. Especially the prenylpseudogermacrenes A–D (**7**–**10**) and prenylpseudohedycaryol (**11**) were frequently found (Figures S18, S27, S46, S63, S80–S82).

The absolute configurations of terpenes can be determined with stereoselectively deuterated precursors, setting additional stereogenic centers in the terpene synthase products of known configuration. The relative configuration of the naturally present stereogenic centers to the stereogenic centers at the deuterated carbons can be determined through NOESY spectroscopy and allows to conclude on the absolute configuration of the terpene. Our method makes use of additional ^13^C‐labels for a highly sensitive analysis by HSQC. For this purpose, the labelled probes (*R*)‐ and (*S*)‐(1‐^13^C,1‐^2^H)IPP,^[31]^ and (*E*)‐ and (*Z*)‐(4‐^13^C,4‐^2^H)IPP^[24]^ were developed that can be used to elongate FPP and its analogue iso‐FPP with GGPPS through a known stereochemical course.^[32]^ Unfortunately, this method is not always suitable for the determination of absolute configurations of macrocyclic ring compounds, because their conformational flexibility sometimes does not allow for an unambiguous interpretation of NOESY spectra. However, the absolute configurations of **1** and **2** could be tentatively assigned from their optical rotations (**1**: [α]_D_^25^=+11.4, *c* 0.07, Me_2_CO, **2**: [α]_D_^25^=+84.6, *c* 0.13, Me_2_CO) which are of opposite sign to the reported optical rotations of (*R*)‐**3** ([α]_D_^25^=−46.3, *c* 1.20, CHCl_3_)^[33]^ and (*R*)‐**4** ([α]_D_^25^=−14.2, *c* 0.48, CHCl_3_)^[34]^ from plants. For **5** NOESY based assignments of diastereotopic hydrogens were possible (Figure S19), and isotopic labelling experiments with (*E*)‐ and (*Z*)‐(4‐^13^C,4‐^2^H)IPP (Figure S83) pointed to the absolute configuration as shown in Scheme 1. For compound **11** no assignment could be made from labelling experiments, but the results showed a high enantiomeric purity of **11** obtained with HdS (90 % *ee*, Figure S84), but only 70 % *ee* for **11** obtained with NrPS (Figure S85). For **12** the production was too low to gain conclusive insights into its absolute configuration from labelling experiments.

**Scheme 3:**
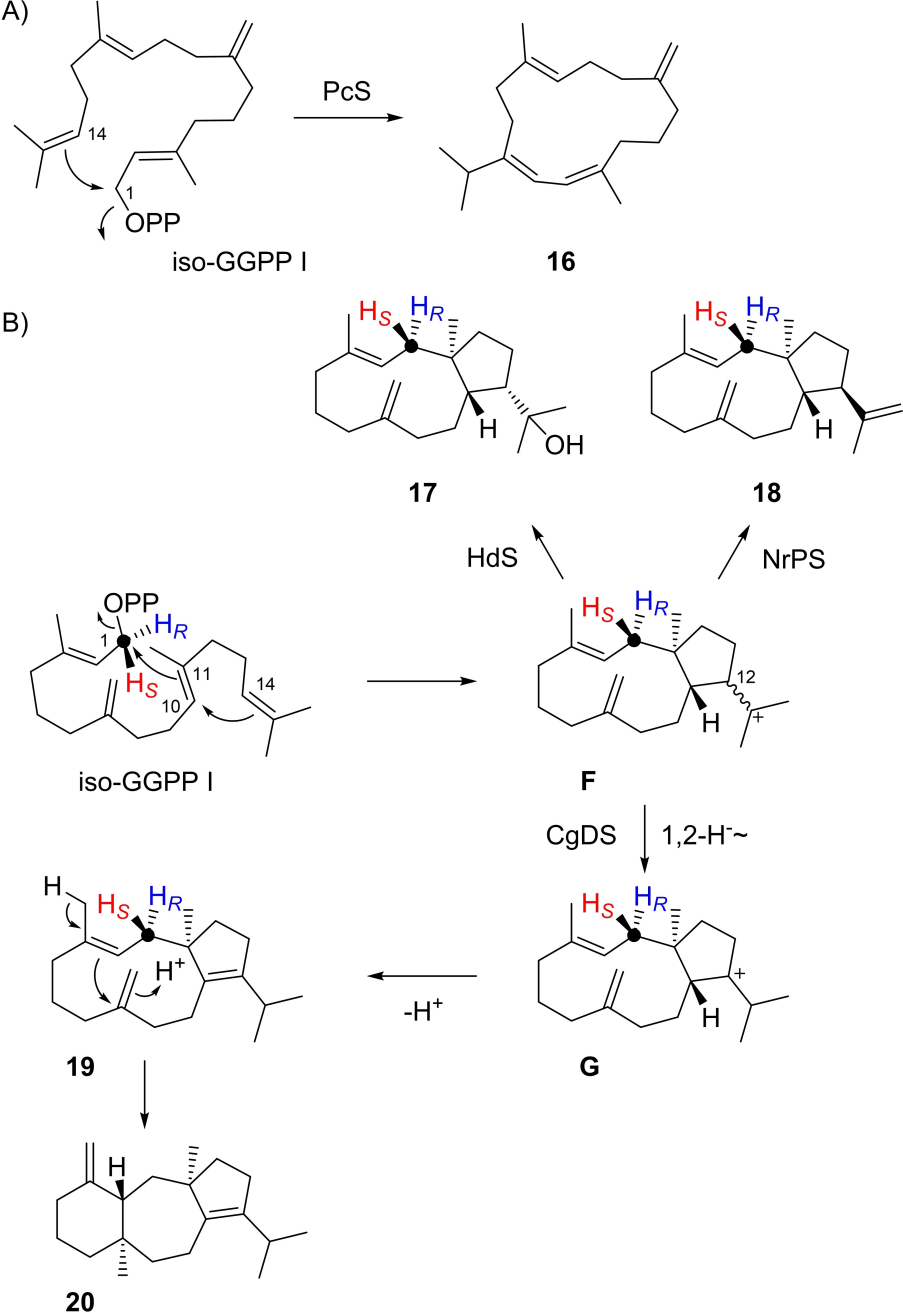
Diterpenes from iso‐GGPP I. A) Formation of **16** by PcS, B) formation of **17** by HdS, **18** by NrPS, and **19** and **20** by CgDS. Red and blue hydrogens are substituted with deuterium in the stereoselectively deuterated probes (*R*)‐ and (*S*)‐(1‐^13^C,1‐^2^H)‐iso‐GGPP for determination of absolute configurations. Black dots represent ^13^C‐labelled carbons in these probes.

The conversion of iso‐GGPP I with the C6=C7 double bond of GGPP shifted to C7=C14 with several DTSs resulted in the iso‐compounds showing the corresponding double bond shift in comparison to the native products from GGPP. Specifically, β‐pinacene synthase (PcS) from the protist *Dyctostelium discoideum* ^[35]^ gave iso‐β‐pinacene (**16**) as main product (Scheme 3A, Figure S86). Notably, most ^1^H signals and the ^13^C signals of the geminal Me groups of **16** show line broadening in the NMR spectra, even at an elevated temperature of 70 °C, and despite the fact that **16** is achiral each aliphatic methylene group shows distinct signals for the two hydrogens (Table S11, Figures S87–S94), suggesting that **16** exists in two enantiomeric slowly interconverting conformers. With 18‐hydroxydolabella‐3,7‐diene synthase (HdS) from *Chitinophaga pinensis* ^[36]^ iso‐GGPP I is converted into 18‐hydroxydolabella‐3,8(17)‐diene (**17**) as the main product (Scheme 3B, Table S12, Figures S95–S103). In both cases of **16** and **17** a cyclisation cascade is followed similar to that for the native enzyme products (Schemes S7 and S8). For a few other DTSs an interruption of the cyclisation cascade at an early intermediate was observed, e.g. with NrPS the bicyclic compound dolabella‐3,8(17),18‐triene (**18**) was obtained as the main product (Table S13, Figures S104–S112). Compounds **17** and **18** arise through cation **F**, but with different configurations at C12, which reflects in both cases the stereochemistry of the natural intermediate from GGPP (Schemes S6 and S8).

Incubation of iso‐GGPP I with the bifunctional fungal PT‐TS enzyme *Colletotrichum gloeosporioides* Dolasta‐1(15),8‐diene Synthase (CgDS)^[37]^ gave a mixture of one diterpene alcohol (**25**) which will be explained below and several diterpene hydrocarbons (Scheme 3B, Figure S113). The hydrocarbon fraction contained **18**, besides two additional compounds that were isolated and identified as the new compound dolabella‐3,8(17),11‐triene (**19**, Table S14, Figures S114–S121) and dolasta‐1(15),8‐diene (**20**), the natural product of CgDS from GGPP.^[37]^ Their formation can be explained by a 1,2‐hydride shift from **F** to **G** and deprotonation to **19**, followed by reprotonation at the olefinic methylene carbon to induce a second ring closure to **20**. Notably, the mechanism towards **20** is very similar to its formation from GGPP, only with the reprotonated double bond in the neutral intermediate being located in a different position (Scheme S9).

**Scheme 4:**
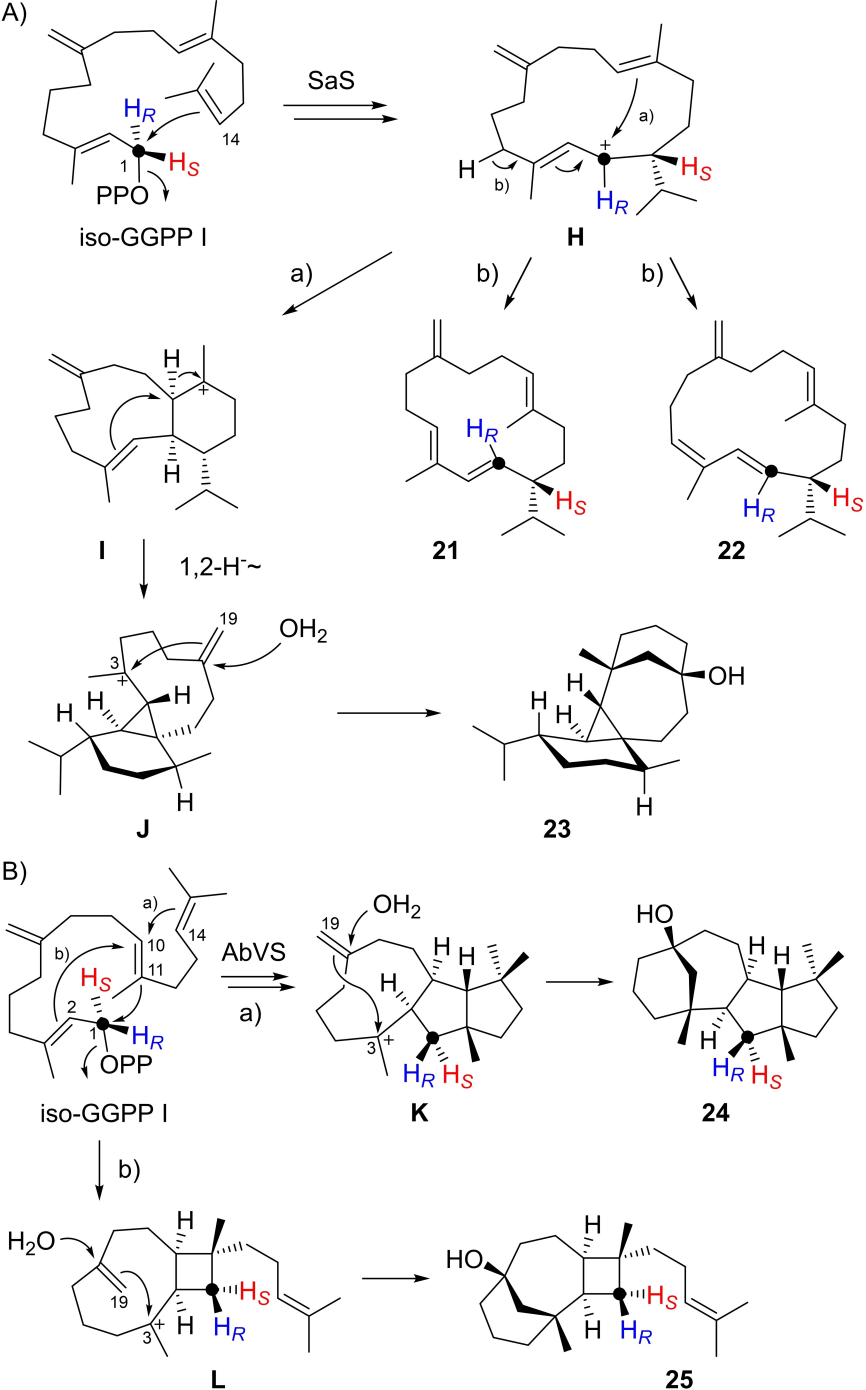
Diterpenes from iso‐GGPP I. A) Formation of **21**–**23** by SaS, B) formation of **24** and **25** by AbVS.

With SaS iso‐GGPP I was converted into the hydrocarbons isothunbergene A (**21**) and B (**22**), besides the diterpene alcohol albataxenol (**23**) as the main product (Tables S15–S17, Figures S122–S146). Notably, the NMR spectra of **23** recorded at 293 K showed line broadening, but sharper signals could be observed at 344 K. The isothunbergenes require 1,14‐cyclisation of iso‐GGPP I and two sequential 1,2‐hydride shifts to **H**, in analogy to the reactions with the native substrate GGPP (Scheme S4). Subsequent deprotonation with formation of an (*E*,*E*) or (*E*,*Z*)‐diene portion leads to **21** and **22** (Scheme 4A). If the cyclisation cascade is continued as for GGPP, another cyclisation leads to **I** which is followed by 1,2‐hydride shift and cyclisation to **J**. At this stage the last remaining double bond becomes involved, which is the one that is shifted in iso‐GGPP I, resulting in a change of the cyclisation mode with attack of water to give **23**. Its skeleton can only be reached from iso‐GGPP I that can undergo 3,19‐cyclisation, but not from GGPP with a Me19 group. The name albataxenol for **23** refers to the foreign nature of this compound (ancient Greek ξϵνοσ=strange, foreign).

With variediene synthase (AbVS) iso‐GGPP I was converted into a complex mixture of several diterpene hydrocarbons and two alcohols (Figure S147). The alcohols were isolated and structurally characterised as variexenol A (**24**) and B (**25**, Tables S18 and S19, Figures S148–S163). Also in case of **24** the initial cyclisation steps towards **K** are analogous to those observed with GGPP (Scheme S5), but later the exo‐methylene C19 takes part in a 3,19‐cyclisation with attack of water, leading to new skeletons (Scheme 4B). For **25** the initial 1,11–10,14 cyclisation is modified to a 1,11–2,10 cyclisation to **L**, followed by 3,19‐cyclisation and attack of water.

**Scheme 5:**
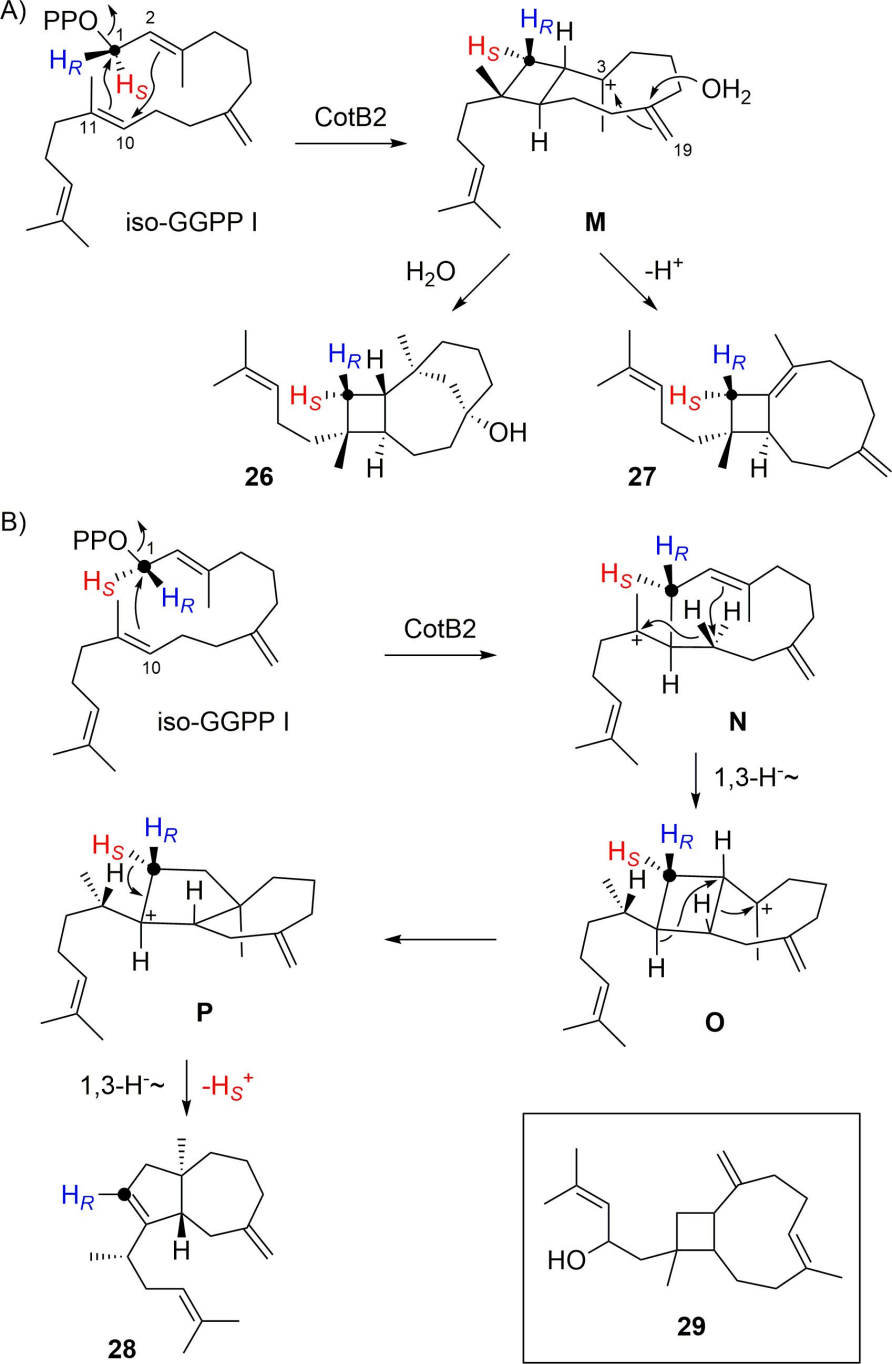
Diterpenes from iso‐GGPP I. A) Formation of **26** and **27** by CotB2, B) formation of **28** by CotB2. Box: structure of xeniaphyllenol (**29**).

Similar findings have been made for cyclooctat‐9‐en‐7‐ol synthase (CotB2)^[38]^ from *Streptomyces iakyrus* that converted iso‐GGPP into 2,3,7‐*triepi*‐variexenol B (**26**), isoxeniaphyllene (**27**) and prenylisodauca‐3,7(14)‐diene (**28**, Tables S20–S22, Figures S164–S188). The name of **27** was assigned, because this compound exhibits the same skeleton as xeniaphyllenol (**29**, box in Scheme 5) from *Xenia macrospiculata*.^[39]^ The formation of **26** and **27** can be explained through 1,11–2,10‐cyclization to **M**, followed by another 3,19‐cyclisation and attack of water to **26**, or by deprotonation to **27** (Scheme 5A). Compound **28** can be formed by 1,10‐cyclisation of iso‐GGPP I to **N**, followed by 1,3‐hydride shift to **O**, rearrangement to **P** and 1,3‐hydride shift with deprotonation from C1 (Scheme 5B). Incubation of (*R*)‐ and (*S*)‐(1‐^13^C,1‐^2^H)‐iso‐GGPP I with CotB2 showed deprotonation from C1 with specific loss of the 1‐*pro*‐*S* and retainment of the 1‐*pro*‐*R* hydrogen (Figure S189). The cyclisation modes observed here for the three compounds **26**–**28** deviate from the natural 1,11–10,14‐cyclisation of GGPP (Scheme S10).

With catenul‐14‐en‐6‐ol synthase from *Catenulispora acidiphila* (CaCS),^[21b]^ iso‐GGPP I was converted into one diterpene hydrocarbon and one alcohol (Figure S190). Their structures were elucidated as precatenulixenol (**30**) and catenulixenol (**31**, Tables S23 and S24, Figures S191–S206). Their formation follows the natural cyclisation cascade with GGPP closely (compare Scheme 6 and Scheme S11) and proceeds through 1,10‐cyclisation of GGPP to **Q**, 1,3‐hydride shift to **R**, and 1,14‐cyclisation to **S**. Deprotonation yields **30**, which can undergo 3,19‐cyclisation to **31**, instead of the naturally observed 2,7‐cyclisation to catenul‐14‐en‐6‐ol.

**Scheme 6:**
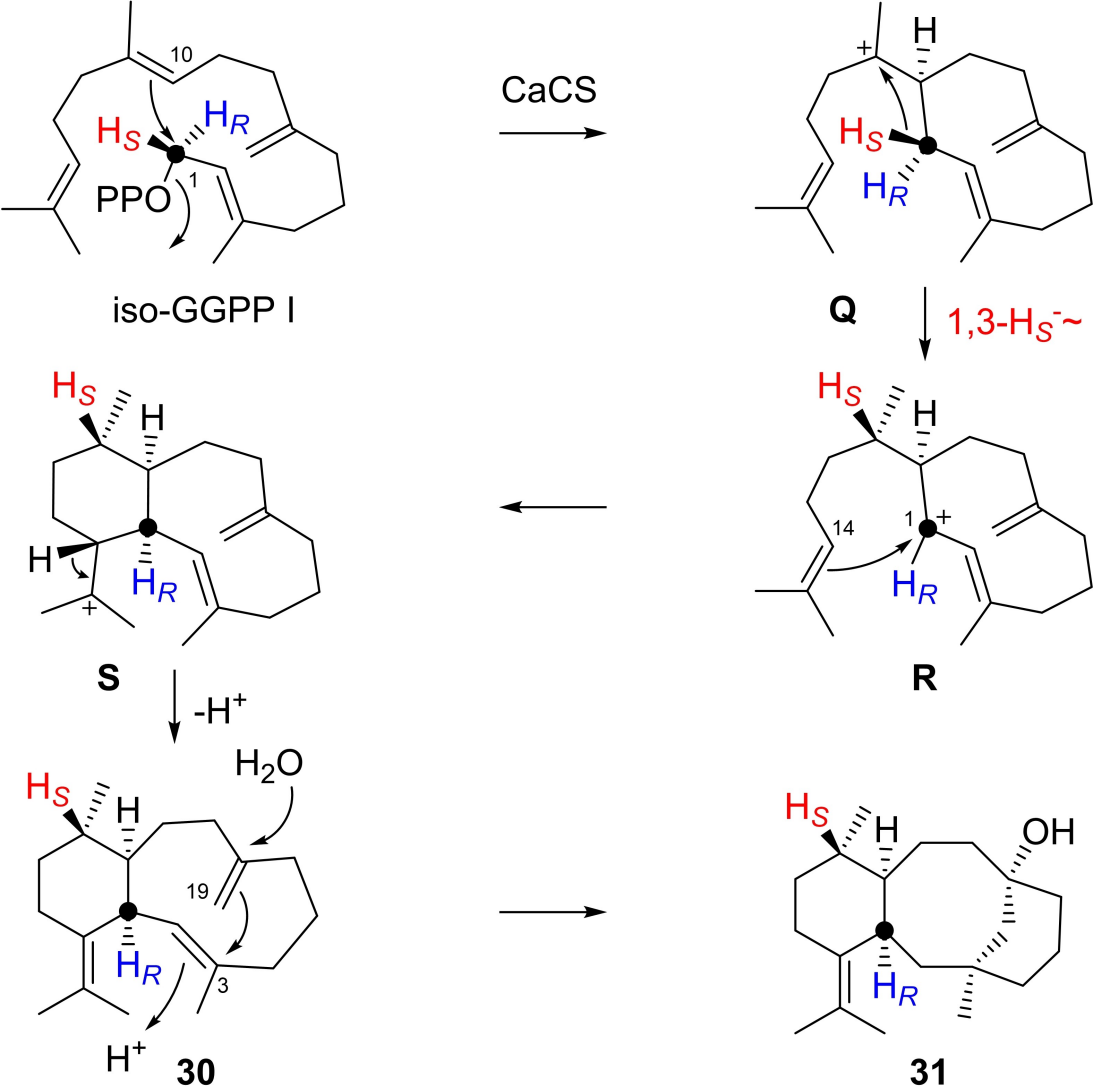
Diterpenes from iso‐GGPP I. Formation of **30** and **31** by CaCS.

**Scheme 7:**
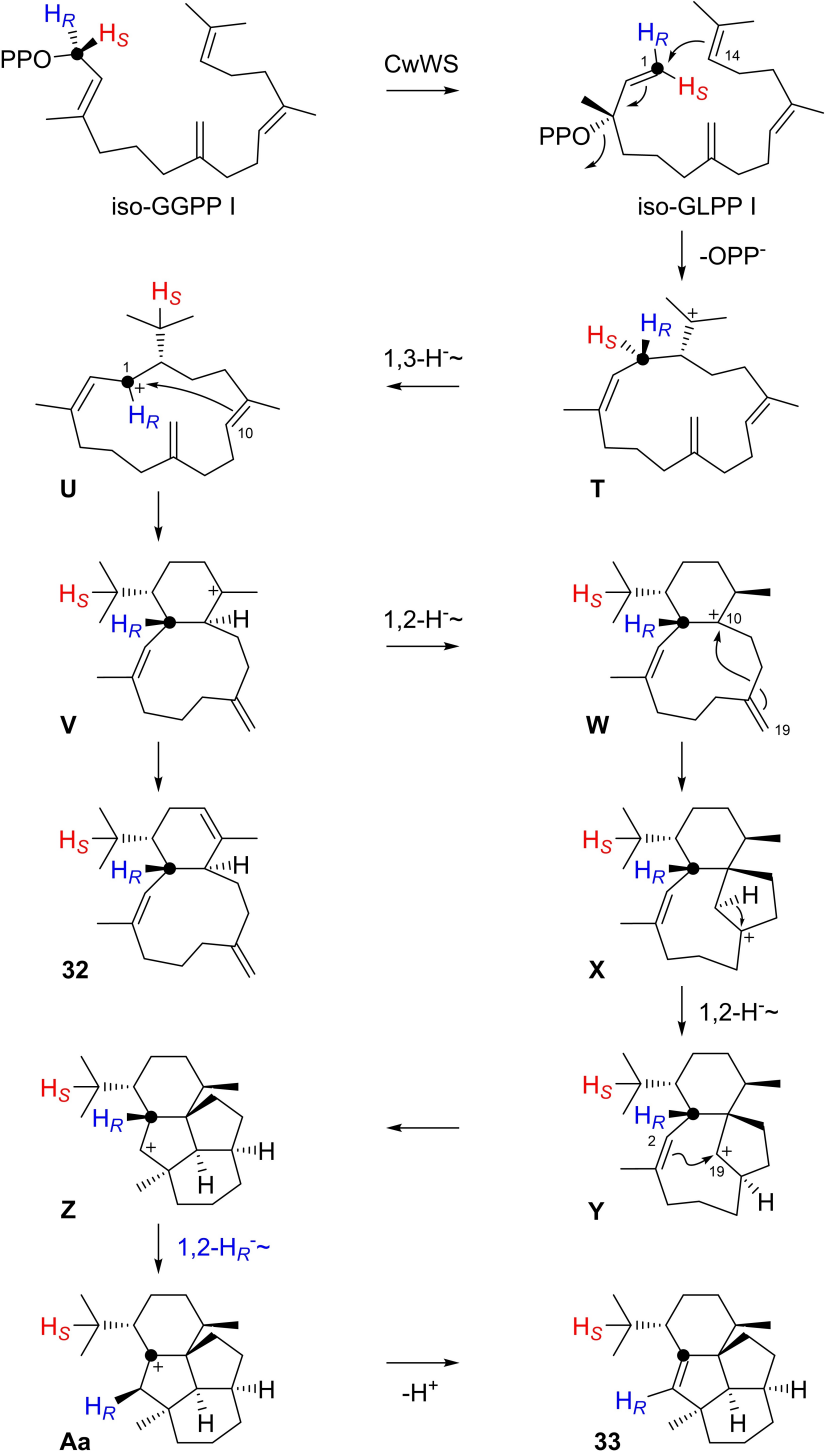
Diterpenes from iso‐GGPP I. Formation of **32** and **33** by CwWS.

Wanjudiene synthase from *Chryseobacterium wanjuense* (CwWS)^[40]^ transformed iso‐GGPP I into two diterpene hydrocarbons (Figure S207). Both compounds were isolated and identified as prewanjuxenene (**32**) and wanjuxenene (**33**, Tables S25 and S26, Figures S208–S223). Also in this case the cyclisation cascade follows in its initial steps the reactions towards natural wanjudiene (Scheme S12), starting with an isomerisation of iso‐GGPP I to iso‐geranyllinalyl diphosphate I (iso‐GLPP I), followed by a 1,14‐cyclisation to **T**, 1,3‐hydride migration to **U** and 1,10‐cyclisation to **V** (Scheme 7). Deprotonation of **V** yields **32**, or the cascade is continued through 1,2‐hydride shift to **W** and 10,19‐cyclisation to **X**. Here again the different double bond location in iso‐GGPP I in comparison to GGPP leads to a distinct cyclisation mode towards a novel skeleton. A 1,2‐hydride shift to **Y** and 2,19‐cyclisation result in a secondary cation **Z** that may be a transient species that is stabilised by a 1,2‐hydride shift to **Aa**. Its deprotonation finally leads to **33**. The 1,3‐hydride shift from **T** to **U** was demonstrated by conversion of (*R*)‐ and (*S*)‐(1‐^13^C,1‐^2^H)‐iso‐GGPP I and GC/MS analysis of the products, showing the cleavage of a deuterated iPr group from the *S*‐configured substrate (Figure S224). Initially, the 1,2‐hydride shift from **Z** to **Aa** was not taken into consideration, but rather a direct deprotonation of **Z** to **33** was assumed. However, the deuterium atoms from both substrates (*R*)‐ and (*S*)‐(1‐^13^C,1‐^2^H)‐iso‐GGPP I remained in the product (Figure S224), with incorporation of deuterium from (*R*)‐(1‐^13^C,1‐^2^H)‐iso‐GGPP I into a neighbouring position of C1 as indicated by a small upfield shift for the labelled carbon (Δ*δ*=−0.1 ppm, Figure S225). Remarkably, the olefinic methylene C19 in iso‐GGPP I becomes involved in two cyclisation events towards **33**.

**Scheme 8:**
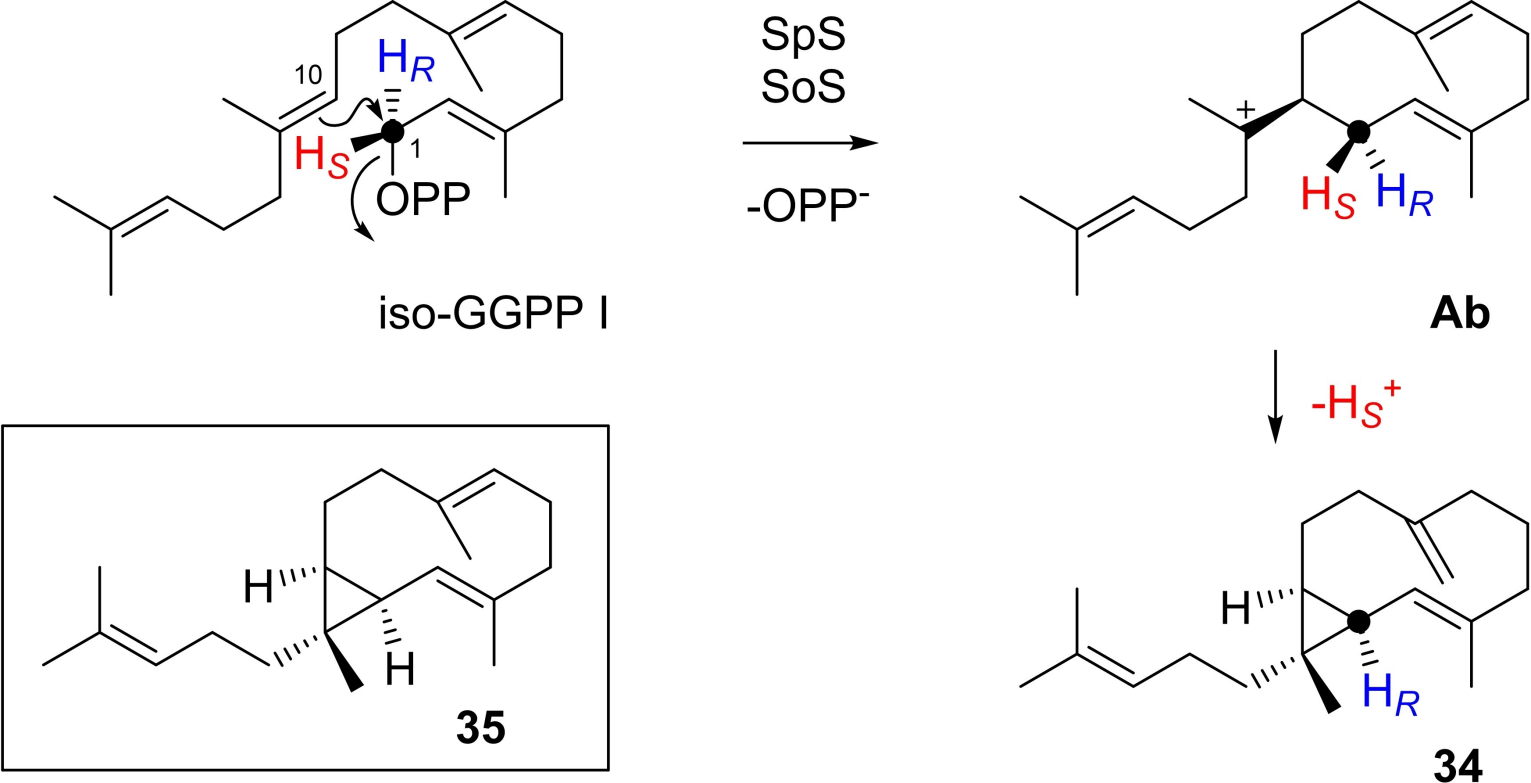
Diterpenes from iso‐GGPP I. Formation of **34** by SpS and SoS.

The enzyme reaction of iso‐GGPP I with SpS^[25]^ yielded a single diterpene hydrocarbon (Figure S226) whose structure was elucidated as that of isocneorubin Y (**34**, Table S27, Figures S227–S234). This compound can be formed by 1,10‐cyclisation followed by deprotonation with cyclopropanation (Scheme 8). The hydrocarbon **34** is structurally related to cneorubin Y (**35**) that is a neutral intermediate towards the SpS product spata‐13,17‐diene (Scheme S2) and was first isolated from *Cneorum tricoccon*.^[41]^ Interestingly, the same compound **34** was obtained from iso‐GGPP I with SoS (Figure S226),^[27]^ showing that in this case the structural modification in the substrate changes the cyclisation mode from 1,11–10,14‐cyclisation with GGPP (Scheme S3) to a 1,10‐cyclisation.

The absolute configurations of the products derived from iso‐GGPP I were also determined through the enantioselective labelling strategy. For this purpose, (*R*)‐ and (*S*)‐(1‐^13^C,1‐^2^H)‐iso‐GGPP were synthesised (Scheme S13) and their enantiomeric purity was shown to be high by Mosher ester analysis (94 % ee and 96 % ee, respectively; Figure S235). Their conversion with HdS and HSQC analysis of the products allowed for the assignment of the absolute configuration of **17** (Figure S236). Analogously, the conversion with NrPS and CgDS revealed the absolute configuration of **18** (which is the same for both enzymes; Figure S237) and **19** (only obtained with CgDS, Figure S238). In the enzyme reactions with SaS one of the hydrogens at C1 migrates for all products so that no stereogenic anchor can be introduced to allow a secure determination of absolute configurations. However, for **20** and **21** it is the same 1‐*pro*‐*S* hydrogen as for the natural enzyme product spiroalbatene that is shifted (Figures S239 and S240, Scheme S4) so that the absolute configurations should be analogous to the absolute configuration of spiroalbatene.^[28]^ The transformation of (*R*)‐ and (*S*)‐(1‐^13^C,1‐^2^H)‐iso‐GGPP with AbVS established the absolute configurations of **24** and **25** (Figure S241 and S242), and with CotB2 the absolute configurations of **26** and **27** were shown (Figure S243 and S244). For the enzyme reactions with CaCS to **30** and **31** one of the C1 hydrogens is migrating (1‐*pro*‐*S*, Figure S245 and S246), but again this is the same hydrogen that also migrates in catenul‐14‐en‐6‐ol biosynthesis (Scheme S11),^[21b]^ suggesting that these compounds have analogous absolute configurations. For CwWS the 1‐*pro*‐*S* hydrogen is shifted into the iPr group in the formation of **32** (Figure S247) and **33** (Figure S224), which is the same hydrogen that also migrates in wanjudiene biosynthesis (Scheme S12).^[40]^ Finally, the formation of **34** with both enzymes SpS and SoS proceeds with loss of the 1‐*pro*‐*S* hydrogen in the cyclopropanation (Figure S248), which is also observed for the SpS reaction to spata‐13,17‐diene (Scheme S2).^[25]^ Therefore, the absolute configurations of **32**–**34** should also be analogous to their natural counterparts.

## Conclusion

In conclusion, this work shows that diterpene synthases not only have a remarkable catalytic potential for the efficient conversion of the natural substrate GGPP, but also broadly accept substrate analogues, e.g. with altered double bond positions as used here. The product yields up to 9 % for a single isolated compound are satisfactory and comparable to the yields observed with GGPP. Acceptance of the substrate analogues iso‐GGPP I and iso‐GGPP II reveal a remarkable plasticity of terpene synthases. However, as shown in this study, many of the investigated diterpene synthases showed a conversion of the substrate analogues iso‐GGPP I and iso‐GGPP II through reactions that especially in the initial steps closely follow the cyclisation cascades observed with GGPP, suggesting that the substrate analogues in comparison to GGPP adopt very similar conformational folds in the active sites. Moreover, in iso‐GGPP I and iso‐GGPP II carbons C18 and C19, represented by non‐reactive Me groups in GGPP, are activated so that they can directly get involved in the cyclisation cascades. Numerous examples in this study have shown that with iso‐GGPP II 1,18‐ instead of 1,11–10,14‐cyclisations can become possible, while with iso‐GGPP I the initial cyclisation steps are often similar to those observed with GGPP, but later in the biosynthesis 3,19‐ or 10,19‐cyclisations can occur. These changes in the cyclisation modes lead to skeletons that are unknown to nature and would be very difficult to make through chemical synthesis. The absolute configurations of all compounds reflect those of the natural enzyme products, which is not unexpected and further demonstrates that the substrate analogues with their minor structural differences compared to GGPP adopt a similar conformation in the enzymes’ active sites as GGPP does. We will continue to investigate the enzymatic potential of terpene synthases with other specifically designed substrate analogues in our future research.

## Conflict of interest

The authors declare no conflict of interest.

1

## Supporting information

As a service to our authors and readers, this journal provides supporting information supplied by the authors. Such materials are peer reviewed and may be re‐organized for online delivery, but are not copy‐edited or typeset. Technical support issues arising from supporting information (other than missing files) should be addressed to the authors.

Supporting Information

## Data Availability

The data that support the findings of this study are available in the supplementary material of this article.
