# Supplementary material for: Diterpene Biosynthesis from Geranylgeranyl Diphosphate Analogues with Changed Reactivities Expands Skeletal Diversity
Source: Angew Chem Int Ed Engl. 2022 Sep 21;61(43):e202211054. doi: 10.1002/anie.202211054 (PMC9826473; doi:10.1002/anie.202211054)
Supplement: Supplementary file 1 — Supporting Information [file ANIE-61-0-s001.pdf]

Angewandte Chemie
Eine Zeitschrift der Gesellschaft Deutscher Chemiker

Supporting Information

Diterpene Biosynthesis from Geranylgeranyl Diphosphate Analogues with Changed Reactivities Expands Skeletal Diversity

*H. Li, J. S. Dickschat**

Table of Contents

Analytical methods	2
Gene cloning, expression and protein purification	3
Enzymatic conversions	4
Compound isolation, spectroscopic and physical characterisation	5
Cyclisation mechanism from GGPP to bonnadiene by BdS	9
Cyclisation mechanism from GGPP to spata-13,17-diene by SpS	10
Cyclisation mechanism from GGPP to spinodienes by SoS	11
Cyclisation mechanism from GGPP to spiroalbatene	12
Cyclisation mechanism from GGPP to variediene by AbVS	13
Cyclisation mechanism from GGPP to phomopsene by NrPS	14
Cyclisation mechanism from GGPP to β-pinacene by PcS	15
Cyclisation mechanism from GGPP to 18-hydroxydolabella-3,7-diene by HdS	16
Cyclisation mechanism from GGPP to dolasta-1(15),8-diene by CgDS	17
Cyclisation mechanism from GGPP to cyclooctat-9-en-7-ol by CotB2	18
Cyclisation mechanism from GGPP to catenul-14-en-6-ol by CaCS	19
Cyclisation mechanism from GGPP to wanjudiene by CwWS	20
Structure elucidation of products formed with BdS from iso-GGPP II	21
Structure elucidation of products formed with SpS from iso-GGPP II	38
Structure elucidation of products formed with SoS from iso-GGPP II	47
Structure elucidation of products formed with SaS from iso-GGPP II	56
Structure elucidation of products formed with AbVS from iso-GGPP II	66
Structure elucidation of products formed with NrPS from iso-GGPP II	83
Products formed from iso-GGPP II with CpCS, CotB2, CgDS, CaCS, CyS	100
Determination of absolute configurations of products from iso-GGPP II	103
Structure elucidation of products formed with PcS from iso-GGPP I	106
Structure elucidation of products formed with HdS from iso-GGPP I	115
Structure elucidation of products formed with NrPS from iso-GGPP I	124
Structure elucidation of products formed with CgDS from iso-GGPP I	133
Structure elucidation of products formed with SaS from iso-GGPP I	142
Structure elucidation of products formed with AbVS from iso-GGPP I	167
Structure elucidation of products formed with CotB2 from iso-GGPP I	184
Structure elucidation of products formed with CaCS from iso-GGPP I	210
Structure elucidation of products formed with CwWS from iso-GGPP I	227
Structure elucidation of products formed with SpS and SoS from iso-GGPP I	246
Synthesis of (*R*)- and (*S*)-(1-^{13}C,1-^{2}H)-iso-GGPP I	255
Determination of absolute configurations of products from iso-GGPP I	259
References	272

Analytical methods

GC/MS

GC/MS analyses were carried out on a 7890B/5977A series gas chromatography/mass selective detector (Agilent, Santa Clara, CA, USA). The GC was equipped with an HP5-MS fused silica capillary column (30 m, 0.25 mm i. d., 0.50 μm film; Agilent) and operated using the settings 1) inlet pressure: 77.1 kPa, He at 23.3 mL min^{-1}, 2) injection volume: 1 – 2 μL, 3) temperature program: 5 min at 50 °C then increasing 5 °C min^{-1} to 320 °C, 4) 60 s valve time, and 5) carrier gas: He at 1.2 mL min^{-1}. The MS was operated with settings 1) source: 230 °C, 2) transfer line: 250 °C, 3) quadrupole: 150 °C and 4) electron energy: 70 eV.

HRMS

High resolution mass spectra (APCI) were recorded on an Orbitrap XL instrument (Thermo Fisher Scientific, Waltham, MA, USA) or using a 7890B/7200 series gas chromatography/accurate mass Q-ToF detector system (Agilent). The GC was equipped with a HP5-MS fused silica capillary column (30 m, 0.25 mm i. d., 0.50 mm film). GC settings were 1) injection volume: 1 μL, 2) temperature program: 5 min at 50 °C, increasing 10 °C min^{-1} to 320 °C, 3) split ratio: 5:1, 60 s valve time and 4) carrier gas flow: He at 1 mL min^{-1}. MS settings were 1) inlet pressure: 83.2 kPa, He flow at 24.6 ml min^{-1}, 2) transfer line temperature: 250 °C, 3) ionization energy: 70 eV.

NMR spectroscopy

NMR spectra were recorded at 298 K on a Bruker (Billerica, MA, USA) Avance III HD Cryo (700 MHz) NMR spectrometer. Spectra were measured in C_6D_6 and referenced against solvent signals (^{1}H-NMR, residual proton signal: δ = 7.16 ppm; ^{13}C-NMR: δ = 128.06 ppm).[1] Coupling constants are given in Hz.

IR spectroscopy

IR spectra were recorded on a Bruker α infrared spectrometer with a diamond ATR probehead. Peak intensities are given as s (strong), m (medium), w (weak) and br (broad).

Optical rotations

Optical rotations were recorded on a Modular Compact Polarimeter MCP 100 (Anton Paar, Graz, Austria). The temperature setting was 25 °C; the wavelength of the light used was 589 nm (sodium D line); the path-length was 10 cm, the compound concentrations *c* are given in g 100 mL^{-1}.

Gene cloning of a CotB2 homolog from *Streptomyces iakyrus*

Genomic DNA was isolated from *Streptomyces iakyrus* DSM 40482. The homologous gene (gene locus tag OO68_RS00455) to the gene encoding CotB2 in *S. melanosporofaciens* was amplified using Q5 High-Fidelity DNA Polymerase (New England Biolabs) and primers P1f (ATGACCGCGGATCTGACCG) and P1r (CTACTGGATCCGTGAGTTGACGTCG). The amplificate was used as template in another PCR amplification using primers P2f (GGCAGCCATATGGCTAGCATGACTGGTGGAATGACCGCGGATCTGACCG) and P2r (TCTCAGTGGTGGTGGTGGTGGTGCTCGAGTCTACTGGATCCGTGAGTTGACGTCG) with homology arm extensions for cloning through homologous recombination in yeast. The DNA fragment was homologously recombined to the linearised (*Hind*III and *Eco*RI digestion) expression plasmid pYE-Express in yeast.[2,3] The yeast cells were plated SM-URA agar plates (425 mg yeast nitrogen base, 1.25 g ammonium sulphate, 5 g glucose, 192.5 mg nutritional supplement minus uracil, 5 g agar, 250 mL water) and grown at 28 °C for 3 days. Plasmid DNA was isolated (Zymoprep Yeast Plasmid Miniprep II kit; Zymo Research, Irvine, CA, USA) and used for electroporation of *E. coli* BL21(DE3) electrocompetent cells. After growth overnight at 37 °C on LB agar amended with kanamycin (50 μg mL^{-1}), single colonies were selected to inoculate LB medium (5 mL) with kanamycin. Cultures were grown overnight at 37 °C followed by isolation of plasmid DNA. The correct insertion of the desired gene was confirmed by sequencing.

Gene expression and protein purification

A preculture of LB medium (10 mL) supplied with kanamycin (50 μg/mL final concentration) was inoculated with the desired *E. coli* expression strain and grown with shaking overnight at 37 °C. The precultures were then used to inoculate main cultures (1/500) in LB medium with kanamycin. The cultures were grown with shaking at 37 °C until OD_{600} = 0.4 – 0.6 was reached. After cooling to 18 °C, expression was induced by the addition of IPTG (0.4 mM final concentration). The cultures were shaken at 18 °C overnight and then centrifuged (5000 x g, 35 min, 4 °C). The cell pellets were resuspended in binding buffer (10 mL/L culture; 20 mM Na_2HPO_4, 500 mM NaCl, 20 mM imidazole, 1 mM $MgCl_2$, pH = 7.4, 4 °C) and lysed by ultra sonification on ice (5x 1 min). The resulting suspension was centrifuged (5400 x g, 3x 7 min, 4 °C) and the supernatant containing the soluble protein was filtrated and loaded on a Ni^{2+}-NTA affinity chromatography column (Ni-NTA superflow, Qiagen, Venlo, Netherlands). The bound target protein was washed with binding buffer (2x 10 mL/L culture) and eluted from the column with elution buffer (2x 6.25 mL/L culture; 20 mM Na_2HPO_4, 500 mM NaCl, 500 mM imidazole, 1 mM $MgCl_2$, pH = 7.4, 4 °C). Protein containing fractions were analysed by SDS-PAGE, yielding satisfyingly pure proteins in all cases as reported previously (references for individual proteins are given in Table S1) and used for enzymatic conversions. Protein concentrations were determined by Bradford assay.[4]

For gene cloning, expression and protein purification of other enzymes please refer to the original work as cited in Table S1.

Enzymatic conversions

Test enzymatic conversions of iso-FPP and IPP (1 mg each) or of iso-GGPP I (1 mg) were done in incubation buffer (1 mL; 50 mM Tris/HCl, 10 mM $MgCl_2$, 10% glycerol, pH = 8.2) containing β-cyclodextrin (10 mM). Enzyme preparations were obtained as reported previously (for references cf. Table S1) and enzymes were added at a concentration of 0.3 mg L^{-1}, followed by incubation for 16 h at 30 °C. The reaction mixtures were extracted with n-Hexane, the extracts were dried with $MgSO_4$ and analysed by GC/MS. These conditions were also used for isotopic labelling experiments, followed by extraction with C_6D_6 and analysis by NMR spectroscopy.

Preparative scale enzymatic conversions of iso-FPP and IPP or of iso-GGPP I were done in incubation buffer containing β-cyclodextrin (10 mM) at a substrate concentration of 1 mmol L^{-1} for 16 h at 30 °C. Enzymes were added at a concentration of 0.3 mg L^{-1}, followed by incubation for 16 h at 30 °C. The reaction mixtures were extracted with n-hexane, the extracts were dried with $MgSO_4$ and the solvent was evaporated.

Table S1. Enzymatic conversions.

enzyme(s)	substrate(s)	scale[a]	isolated compounds
BdS,[5] GGPPS[6]	iso-FPP, IPP	25 mg	**1**, **2**
SpS,[7] GGPPS[6]	iso-FPP, IPP	50 mg	**5**
SoS,[8] GGPPS[6]	iso-FPP, IPP	50 mg	**7**
SaS,[9] GGPPS[6]	iso-FPP, IPP	25 mg	**8**
AbVS[10]	iso-FPP, IPP	75 mg	**9**, **10**
NrPS[11]	iso-FPP, IPP	40 mg	**11**, **12**
PcS[12]	iso-GGPP I	60 mg	**16**
HdS[13]	iso-GGPP I	40 mg	**17**
NrPS[11]	iso-GGPP I	30 mg	**18**
CgDS[14]	iso-GGPP I	100 mg	**19**, **20**[14]
SaS[9]	iso-GGPP I	200 mg	**21**, **22**, **23**
AbVS[10]	iso-GGPP I	75 mg	**24**, **25**
CotB2[15]	iso-GGPP I	40 mg	**26**, **27**, **28**
CaCS[16]	iso-GGPP I	80 mg	**30**, **31**
CwWS[17]	iso-GGPP I	50 mg	**32**, **33**
SpS[7]	iso-GGPP I	60 mg	**34**
SoS[8]	iso-GGPP I	40 mg	**34**

[a] The amounts refer to iso-FPP or iso-GGPP I. In the incubations with iso-FPP, IPP was used in equimolar amounts.

Compound isolation, spectroscopic and physical characterisation

Isonephthenol (1). This compound was isolated by column chromatography on silica gel with pentane/Et_2O = 2:1. Yield: 0.7 mg (2.4 μmol, 4 %). TLC (pentane/Et_2O = 2:1): R_f = 0.52. $[\alpha]_D^{25}$ = +11.4 (*c* 0.07, acetone), HRMS (EI): $[M]^+$ calcd. for $C_{20}H_{34}O^+$ *m*/*z* 290.2604; found *m*/*z* 290.2619. GC (HP5-MS): *I* = 2185. IR (diamond ATR): ṽ / cm^{-1} = 3002 (m), 2927 (m), 2856 (w), 1738 (s), 1447 (w), 1366 (s), 1216 (s), 1092 (w), 1020 (w), 886 (w), 794 (w), 524 (w). For NMR data cf. Table S2.

Isocembrene A (2). This compound was isolated by Column chromatography on silica gel with pentane. Yield: 1.3 mg (4.8 μmol, 8 %). TLC (pentane): R_f = 0.49. $[\alpha]_D^{25}$ = +84.6 (*c* 0.13, acetone), HRMS (EI): $[M]^+$ calcd. for $C_{20}H_{32}^+$ *m*/*z* 272.2498; found *m*/*z* 272.2507. GC (HP5-MS): *I* = 1994. IR (diamond ATR): ṽ / cm^{-1} = 3069 (w), 2928 (s), 2871 (m), 2145 (w), 1969 (w), 1738 (s), 1643 (w), 1441 (m), 1372 (m), 1260 (m), 1206 (m), 1092 (w), 1021 (w), 886 (m), 803 (m), 839 (w), 519 (w), 452 (w). For NMR data cf. Table S3.

Pseudoobscuronatin (5). This compound was isolated by column chromatography on silica gel with pentane/Et_2O = 1:1. Yield: 0.7 mg (2.4 μmol, 2 %). TLC (pentane/Et_2O = 1:1): R_f = 0.70. $[\alpha]_D^{25}$ = –1.5 (*c* 0.07, acetone), HRMS (EI): $[M]^+$ calcd. for $C_{20}H_{34}O^+$ *m*/*z* 290.2604; found *m*/*z* 272.2504 (–H_2O). GC (HP5-MS): *I* = 2058. IR (diamond ATR): ṽ / cm^{-1} = 2958 (w), 2921 (s), 2851 (m), 1739 (m), 1673 (w), 1632 (w), 1451 (w), 1374 (w), 1260 (m), 1216 (w), 1097 (m), 1022 (m), 798 (m). For NMR data cf. Table S4.

Prenylpseudogermacrene A (7). This compound was isolated by column chromatography on silica gel with pentane. Yield: 0.8 mg (2.9 μmol, 3 %). TLC (pentane): R_f = 0.43. HRMS (EI): $[M]^+$ calcd. for $C_{20}H_{32}^+$ *m*/*z* 272.2498; found *m*/*z* 272.2508. GC (HP5-MS): *I* = 2068. IR (diamond ATR): ṽ / cm^{-1} = 2970 (w), 2924 (w), 2852 (w), 1738 (s), 1440 (w), 1365 (m), 1206 (m), 1093 (m), 517 (w). For NMR data cf. Table S5.

Prenylpseudogermacrene B (8). This compound was isolated by column chromatography on silica gel with pentane. Yield: 1.2 mg (4.4 μmol, 8 %). TLC (pentane): R_f = 0.41. HRMS (EI): $[M]^+$ calcd. for $C_{20}H_{32}^+$ *m*/*z* 272.2498; found *m*/*z* 273.2575 (+H). GC (HP5-MS): *I* = 2034. IR (diamond ATR): ṽ / cm^{-1} = 2953 (s), 2915 (s), 2850 (s), 1738 (m), 1669 (w), 1438 (m), 1365 (m), 1260 (w), 1228 (m), 1216 (m), 1205 (m), 1091 (m), 1020 (m), 819 (m), 804 (m). For NMR data cf. Table S6.

Prenylpseudogermacrene C (9). This compound was isolated by column chromatography on silica gel followed by HPLC. Yield: 0.9 mg (3.3 μmol, 2 %). TLC (pentane): R_f = 0.51. HRMS (EI): $[M]^+$ calcd. for $C_{20}H_{32}^+$ *m*/*z* 272.2498; found *m*/*z* 272.2506. GC (HP5-MS): *I* = 2055. IR (diamond ATR): ṽ / cm^{-1} = 2954 (m), 2920 (s), 2853 (m), 2182 (w), 1951 (w), 1741 (w), 1671 (w), 1450 (m), 1377 (w), 1260 (w), 1102 (w), 1031 (w), 834 (w), 791 (w). For NMR data cf. Table S7.

Prenylpseudogermacrene D (10). This compound was isolated by column chromatography on silica gel followed by HPLC. Yield: 0.8 mg (2.9 μmol, 2 %). TLC (pentane): R_f = 0.51. HRMS (EI): $[M]^+$ calcd. for $C_{20}H_{32}^+$ *m*/*z* 272.2498; found *m*/*z* 272.2495. GC (HP5-MS): *I* = 2076. IR (diamond ATR): ṽ / cm^{-1} = 2969 (m), 2924 (m), 2853 (m), 1738 (s), 1439 (w), 1365 (m), 1228 (m), 1104 (w), 1021 (w), 800 (w), 516 (w). For NMR data cf. Table S8.

Prenylpseudohedycaryol (11). This compound was isolated by column chromatography on silica gel with pentane/Et_2O = 2:1. Yield: 1.2 mg (4.1 μmol, 4 %). TLC (pentane/Et_2O = 2:1): R_f = 0.59. $[\alpha]_D^{25}$ = –11.7 (*c* 0.12, acetone), HRMS (EI): $[M]^+$ calcd. for $C_{20}H_{34}O^+$ *m*/*z* 290.2604; found *m*/*z* 290.2608. GC (HP5-MS): *I* = 2241. IR (diamond ATR): ṽ / cm^{-1} = 3453 (w), 2923

(s), 2852 (s), 1737 (w), 1668 (w), 1443 (m), 1380 (m), 1216 (w), 1203 (w), 1106 (w), 1076 (w), 822 (w), 489 (w). For NMR data cf. Table S9.

Pseudodollabella-3,7,18-triene (12). This compound was isolated by column chromatography on silica gel with pentane. Yield: 0.5 mg (1.8 μmol, 2 %). TLC (pentane): R_f = 0.64. $[\alpha]_D^{25}$ = +2.0 (*c* 0.05, acetone), HRMS (EI): $[M]^+$ calcd. for $C_{20}H_{32}^+$ *m*/*z* 272.2498; found *m*/*z* 273.2571 (+H). GC (HP5-MS): *I* = 2032. IR (diamond ATR): $\tilde{\nu}$ / cm^{-1} = 3363 (w), 2956 (m), 2922 (s), 2852 (m), 1659 (w), 1632 (w), 1467 (w), 1444 (w), 1260 (m), 1094 (m), 1061 (m), 884 (w), 799 (m), 689 (w). For NMR data cf. Table S10.

Iso-β-pinacene (16). This compound was isolated by column chromatography on silica gel with pentane. Yield: 1.0 mg (3.7 μmol, 3 %). TLC (pentane): R_f = 0.46. $[\alpha]_D^{25}$ = +4.0 (*c* 0.10, acetone), HRMS (EI): $[M]^+$ calcd. for $C_{20}H_{32}^+$ *m*/*z* 272.2498; found *m*/*z* 273.2574 (+H). GC (HP5-MS): *I* = 1991. IR (diamond ATR): $\tilde{\nu}$ / cm^{-1} = 2957 (m), 2925 (s), 2853 (m), 2408 (w), 2164 (w), 2016 (w), 1985 (w), 1641 (w), 1461 (w), 1444 (w), 880 (w). For NMR data cf. Table S11.

18-Hydroxydolabella-3,8(17)-diene (17). This compound was isolated by column chromatography on silica gel with pentane/Et_2O = 1:1. Yield: 2.0 mg (6.9 μmol, 9 %). TLC (pentane/Et_2O = 5:1): R_f = 0.35. $[\alpha]_D^{25}$ = +18.3 (*c* 0.18, acetone), HRMS (EI): $[M]^+$ calcd. for $C_{20}H_{34}O^+$ *m*/*z* 290.2604; found *m*/*z* 291.2680 (+H). GC (HP5-MS): *I* = 2214. IR (diamond ATR): $\tilde{\nu}$ / cm^{-1} = 2970 (s), 2929 (s), 2865 (m), 2265 (w), 2141 (w), 2019 (w), 1738 (m), 1642 (w), 1444 (w), 1365 (m), 1228 (m), 1216 (m), 1133 (w), 882 (w). For NMR data cf. Table S12.

Dolabella-3,8(17),18-triene (18). This compound was isolated by column chromatography on silica gel with pentane. Yield: 0.7 mg (2.4 μmol, 4 %). TLC (pentane): R_f = 0.60. $[\alpha]_D^{25}$ = +17.2 (*c* 0.06, acetone), HRMS (EI): $[M]^+$ calcd. for $C_{20}H_{32}^+$ *m*/*z* 272.2498; found *m*/*z* 273.2574 (+H). GC (HP5-MS): *I* = 1984. IR (diamond ATR): $\tilde{\nu}$ / cm^{-1} = 3068 (w), 2926 (s), 2854 (m), 1736 (m), 1645 (w), 1455 (m), 1376 (m), 1228 (m), 1216 (m), 885 (m), 512 (w). For NMR data cf. Table S13.

Dolabella-3,8(17),11-triene (19). This compound was isolated by column chromatography on silica gel with pentane. Yield: 1.0 mg (3.7 μmol, 2 %). TLC (pentane): R_f = 0.69. $[\alpha]_D^{25}$ = –12.0 (*c* 0.10, acetone), HRMS (EI): $[M]^+$ calcd. for $C_{20}H_{32}^+$ *m*/*z* 272.2498; found *m*/*z* 272.2497. GC (HP5-MS): *I* = 1893. IR (diamond ATR): $\tilde{\nu}$ / cm^{-1} = 2968 (s), 2930 (s), 2863 (m), 1738 (s), 1453 (w), 1366 (m), 1216 (m), 889 (w), 538 (w), 517 (w). For NMR data cf. Table S14.

Isothunbergene A (21). This compound was isolated by column chromatography on silica gel followed by HPLC. Yield: 0.8 mg (2.9 μmol, 1 %). TLC (pentane): R_f = 0.60. $[\alpha]_D^{25}$ = +21.3 (*c* 0.08, acetone), HRMS (EI): $[M]^+$ calcd. for $C_{20}H_{32}^+$ *m*/*z* 272.2498; found *m*/*z* 272.2502. GC (HP5-MS): *I* = 1973. IR (diamond ATR): $\tilde{\nu}$ / cm^{-1} = 2954 (s), 2924 (s), 2870 (s), 2854 (s), 1974 (w), 1736 (w), 1641 (w), 1443 (m), 1383 (m), 1365 (m), 1248 (br), 1023 (m), 964 (m), 884 (m), 804 (w), 544 (w). For NMR data cf. Table S15.

Isothunbergene B (22). This compound was isolated by column chromatography on silica gel followed by HPLC. Yield: 1.7 mg (6.2 μmol, 2 %). TLC (pentane): R_f = 0.60. $[\alpha]_D^{25}$ = +25.3 (*c* 0.17, acetone), HRMS (EI): $[M]^+$ calcd. for $C_{20}H_{32}^+$ *m*/*z* 272.2498; found *m*/*z* 272.2504. GC (HP5-MS): *I* = 1973. IR (diamond ATR): $\tilde{\nu}$ / cm^{-1} = 2955 (s), 2923 (s), 2871 (s), 1738 (m), 1645 (w), 1440 (m), 1376 (m), 1229 (w), 1216 (w), 1095 (w), 1029 (m), 961 (m), 888 (m), 797 (w), 543 (w). For NMR data cf. Table S16.

Albataxenol (23). This compound was isolated by column chromatography on silica gel with pentane/Et_2O = 2:1. Yield: 1.4 mg (4.8 μmol, 1 %). TLC (pentane/Et_2O = 2:1): R_f = 0.20. $[\alpha]_D^{25}$ = +8.5 (*c* 0.13, acetone), HRMS (EI): $[M]^+$ calcd. for $C_{20}H_{34}O^+$ *m*/*z* 290.2604; found *m*/*z*

290.2606. GC (HP5-MS): I = 2084. IR (diamond ATR): $\tilde{\nu}$ / cm^{-1} = 3389 (m), 2951 (s), 2923 (s), 2866 (s), 1461 (w), 1370 (m), 1091 (w), 1050 (w), 987 (w), 429 (w). For NMR data cf. Table S17.

Variexenol A (24). This compound was isolated by column chromatography on silica gel with pentane/Et_2O = 3:1. Yield: 0.6 mg (2.1 µmol, 1 %). TLC (pentane/Et_2O = 1:1): R_f = 0.92. $[\alpha]_D^{25}$ = +11.7 (*c* 0.06, acetone), HRMS (EI): $[M]^+$ calcd. for $C_{20}H_{34}O^+$ *m*/*z* 290.2604; found *m*/*z* 290.2610. GC (HP5-MS): I = 2103. IR (diamond ATR): $\tilde{\nu}$ / cm^{-1} = 3015 (w), 2925 (m), 2857 (w), 1738 (s), 1455 (w), 1365 (m), 1206 (m), 527 (w), 515 (w). For NMR data cf. Table S18.

Variexenol B (25). This compound was isolated by column chromatography on silica gel with pentane/Et_2O = 3:1. Yield: 0.6 mg (2.1 µmol, 1 %). TLC (pentane/Et_2O = 1:1): R_f = 0.88. $[\alpha]_D^{25}$ = –3.6 (*c* 0.06, acetone), HRMS (EI): $[M]^+$ calcd. for $C_{20}H_{34}O^+$ *m*/*z* 290.2604; found *m*/*z* 290.1612. GC (HP5-MS): I = 2193. IR (diamond ATR): $\tilde{\nu}$ / cm^{-1} = 2924 (s), 2853 (m), 1715 (w), 1458 (w), 1376 (w), 1361 (w), 1262 (w), 1024 (w). For NMR data cf. Table S19.

2,3,7-*triepi*-Variexenol B (26). This compound was isolated by column chromatography on silica gel with pentane/Et_2O = 2:1. Yield: 1.0 mg (3.4 µmol, 4 %). TLC (pentane/Et_2O = 2:1): R_f = 0.29. $[\alpha]_D^{25}$ = +3.0 (*c* 0.10, acetone), HRMS (EI): $[M]^+$ calcd. for $C_{20}H_{34}O^+$ *m*/*z* 290.2604; found *m*/*z* 291.2682 (+H). GC (HP5-MS): I = 2134. IR (diamond ATR): $\tilde{\nu}$ / cm^{-1} = 2951 (m), 2925 (m), 2863 (m), 1738 (s), 1454 (w), 1366 (m), 1228 (m), 1216 (m), 538 (w). For NMR data cf. Table S20.

Isoxeniaphyllene (27). This compound was isolated by column chromatography on silica gel with pentane. Yield: 0.9 mg (3.3 µmol, 4 %). TLC (pentane): R_f = 0.60. $[\alpha]_D^{25}$ = –1.2 (*c* 0.09, acetone), HRMS (EI): $[M]^+$ calcd. for $C_{20}H_{32}^+$ *m*/*z* 272.2498; found *m*/*z* 272.2498. GC (HP5-MS): I = 2013. IR (diamond ATR): $\tilde{\nu}$ / cm^{-1} = 3751 (w), 3363 (w), 2924 (s), 2853 (m), 2361 (w), 2194 (w), 1990 (w), 1447 (w), 1375 (w), 1099 (w), 887 (w), 783 (w), 546 (w). For NMR data cf. Table S21.

Prenylisodauca-3,7(14)-diene (28). This compound was isolated by column chromatography on silica gel with pentane. Yield: 0.8 mg (2.9 µmol, 4 %). TLC (pentane): R_f = 0.75. $[\alpha]_D^{25}$ = +37.5 (*c* 0.08, acetone), HRMS (EI): $[M]^+$ calcd. for $C_{20}H_{32}^+$ *m*/*z* 272.2498; found *m*/*z* 272.2502. GC (HP5-MS): I = 1919. IR (diamond ATR): $\tilde{\nu}$ / cm^{-1} = 3067 (w), 3017 (m), 2956 (s), 2859 (m), 2042 (w), 1967 (w), 1868 (w), 1645 (w), 1448 (m), 1373 (m), 1208 (w), 1117 (w), 1016 (w), 880 (m), 639 (w). For NMR data cf. Table S22.

Precatenulixenol (30). This compound was isolated by column chromatography on silica gel with pentane. Yield: 0.4 mg (1.5 µmol, 1 %). TLC (pentane): R_f = 0.54. $[\alpha]_D^{25}$ = –16.7 (*c* 0.04, acetone), HRMS (EI): $[M]^+$ calcd. for $C_{20}H_{32}^+$ *m*/*z* 272.2498; found *m*/*z* 272.2507. GC (HP5-MS): I = 2056. IR (diamond ATR): $\tilde{\nu}$ / cm^{-1} = 3069 (w), 2963 (m), 2919 (s), 2849 (m), 2047 (w), 1868 (w), 1693 (w), 1446 (m), 1375 (w), 1262 (w), 877 (m). For NMR data cf. Table S23.

Catenulixenol (31). This compound was isolated by column chromatography on silica gel with pentane/Et_2O = 2:1. Yield: 1.4 mg (4.8 µmol, 3 %). TLC (pentane/Et_2O = 2:1): R_f = 0.24. $[\alpha]_D^{25}$ = –64.3 (*c* 0.14, acetone), HRMS (EI): $[M]^+$ calcd. for $C_{20}H_{34}O^+$ *m*/*z* 290.2604; found *m*/*z* 290.2590. GC (HP5-MS): I = 2222. IR (diamond ATR): $\tilde{\nu}$ / cm^{-1} = 3369 (w), 2948 (s), 2921 (s), 2485 (s), 2165 (w), 1672 (w), 1456 (m), 1375 (m), 1245 (w), 1069 (m), 1011 (m), 902 (w), 621 (w). For NMR data cf. Table S24.

Prewanjuxenene (32). This compound was isolated by by column chromatography on silica gel (dealt with silver nitrate) with pentane. Yield: 1.8 mg (6.6 µmol, 7 %). TLC (pentane): R_f = 0.86. $[\alpha]_D^{25}$ = –47.1 (*c* 0.17, acetone), HRMS (EI): $[M]^+$ calcd. for $C_{20}H_{32}^+$ *m*/*z* 272.2498; found *m*/*z* 272.2502. GC (HP5-MS): I = 1921. IR (diamond ATR): $\tilde{\nu}$ / cm^{-1} = 2953 (s), 2921 (s), 2563

(s), 2830 (m), 1639 (w), 1449 (m), 1365 (w), 1164 (w), 1059 (w), 881 (s), 539 (w). For NMR data cf. Table S25.

Wanjuxenene (33). This compound was isolated by by column chromatography on silica gel with pentane. Yield: 0.5 mg (1.8 μmol, 2 %). TLC (pentane): R_f = 0.96. $[\alpha]_D^{25}$ = +5.0 (*c* 0.04, acetone), HRMS (EI): $[M]^+$ calcd. for $C_{20}H_{32}^+$ *m*/*z* 272.2498; found *m*/*z* 272.2498. GC (HP5-MS): *I* = 1882. IR (diamond ATR): $\tilde{\nu}$ / cm^{-1} = 2953 (s), 2923 (s), 2853 (s), 1738 (w), 1656 (w), 1458 (m), 1365 (m), 1343 (m), 1216 (w), 1098 (w), 854 (w), 540 (m). For NMR data cf. Table S26.

Isocneorubin Y (34). This compound was isolated by by column chromatography on silica gel with pentane. Yield: 1.1 mg (4.0 μmol, 5 %) from SoS, 0.63mg (2.3 μmol, 2 %) from SpS. TLC (pentane): R_f = 0.60. $[\alpha]_D^{25}$ = –33.0 (*c* 0.11, C_6D_6), HRMS (EI): $[M]^+$ calcd. for $C_{20}H_{32}^+$ *m*/*z* 272.2498; found *m*/*z* 273.2575 (+H). GC (HP5-MS): *I* = 2017. IR (diamond ATR): $\tilde{\nu}$ / cm^{-1} = 2969 (w), 2923 (w), 2852 (w), 2205 (w), 1738 (s), 1435 (w), 1365 (m), 1229 (m), 1092 (w), 1018 (w), 880 (w), 538 (w). For NMR data cf. Table S27.

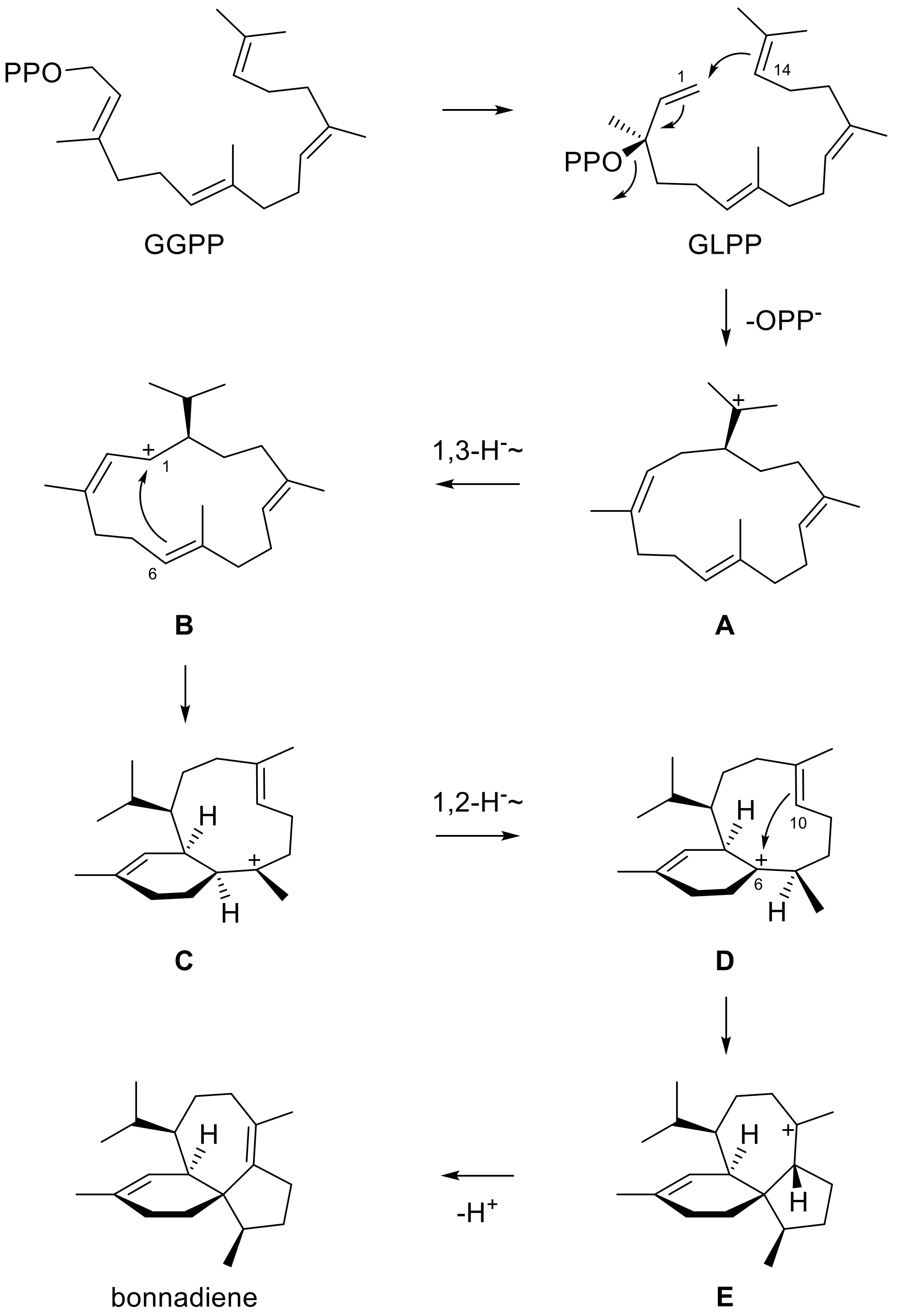

Scheme S1. Cyclisation mechanism from GGPP to bonnadiene by BdS from *A. albata*.[5]

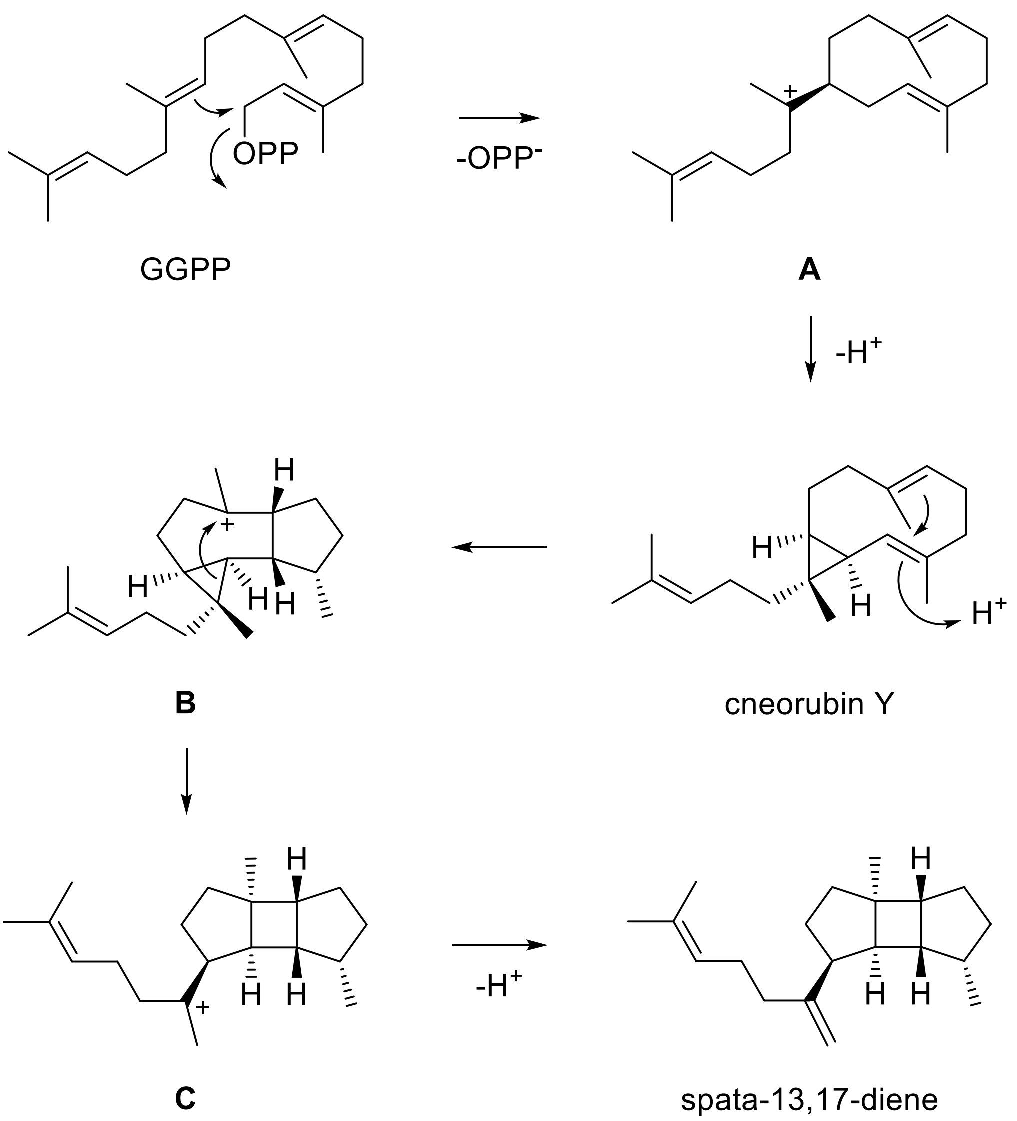

Scheme S2. Cyclisation mechanism from GGPP to spata-13,17-diene by SpS from *S. xinhaiensis*.[7]

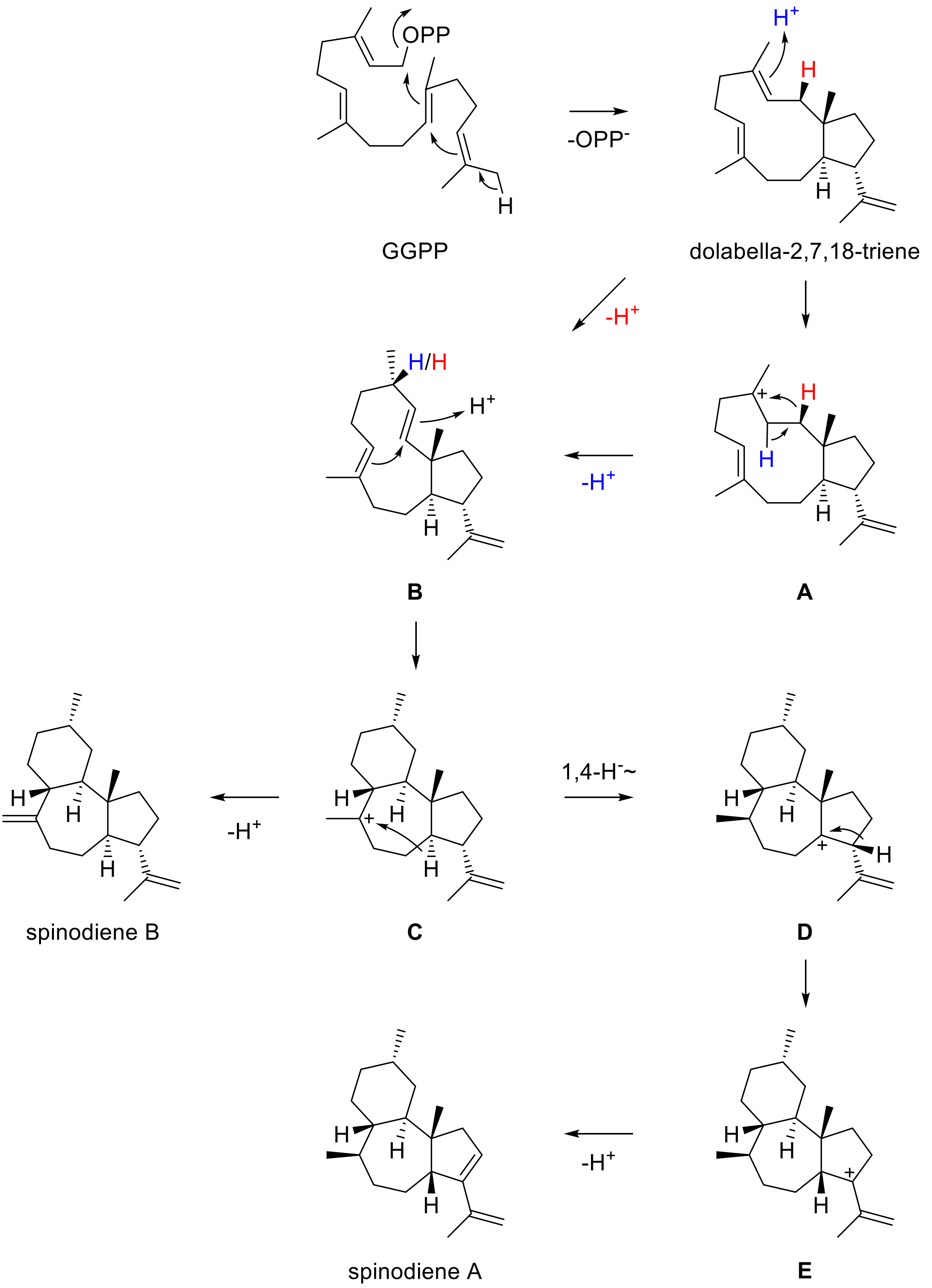

Scheme S3. Cyclisation mechanism from GGPP to spinodienes by SoS from *S. spinosa*.[8]

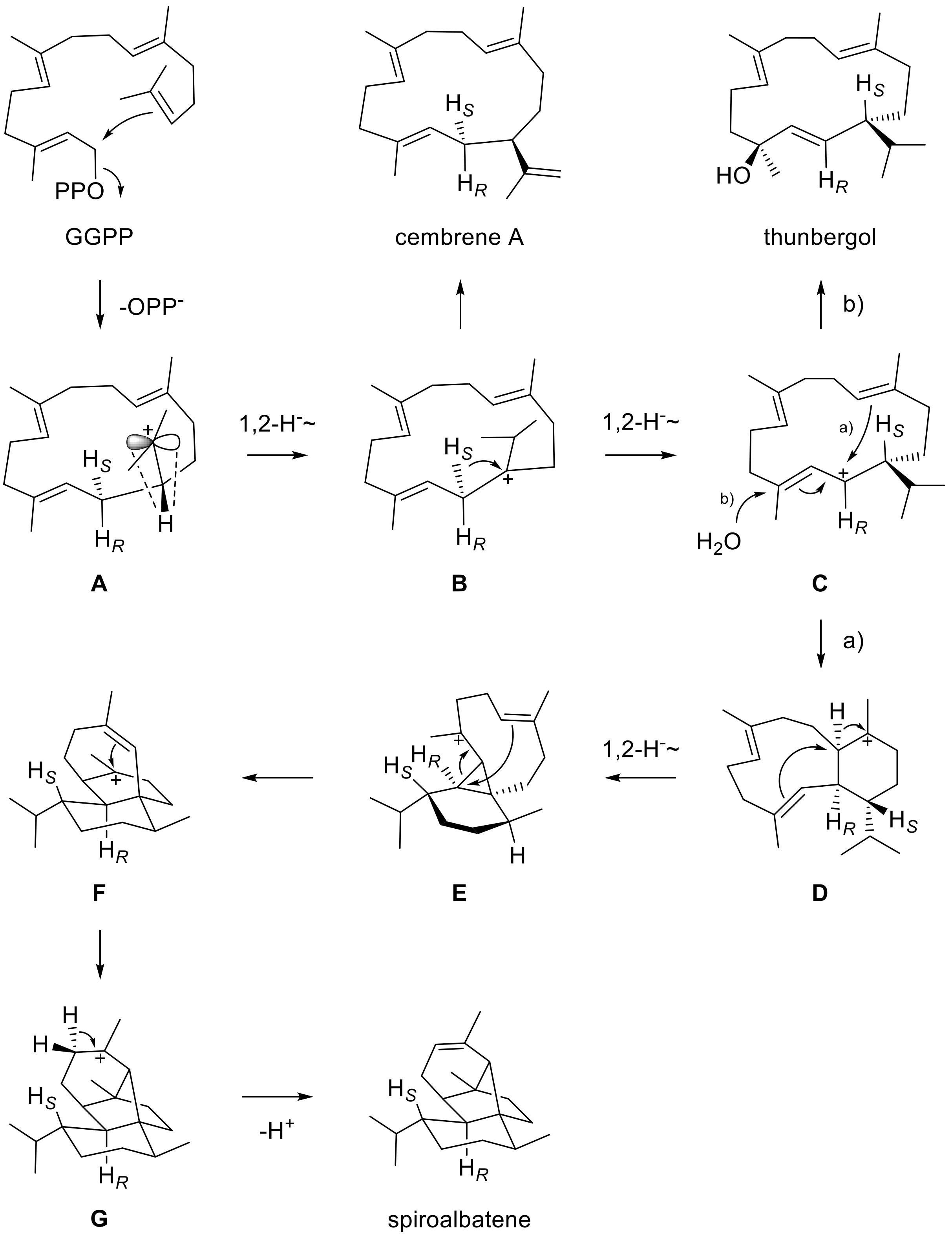

Scheme S4. Cyclisation mechanism from GGPP to spiroalbatene by SaS from *A. albata*.[9]

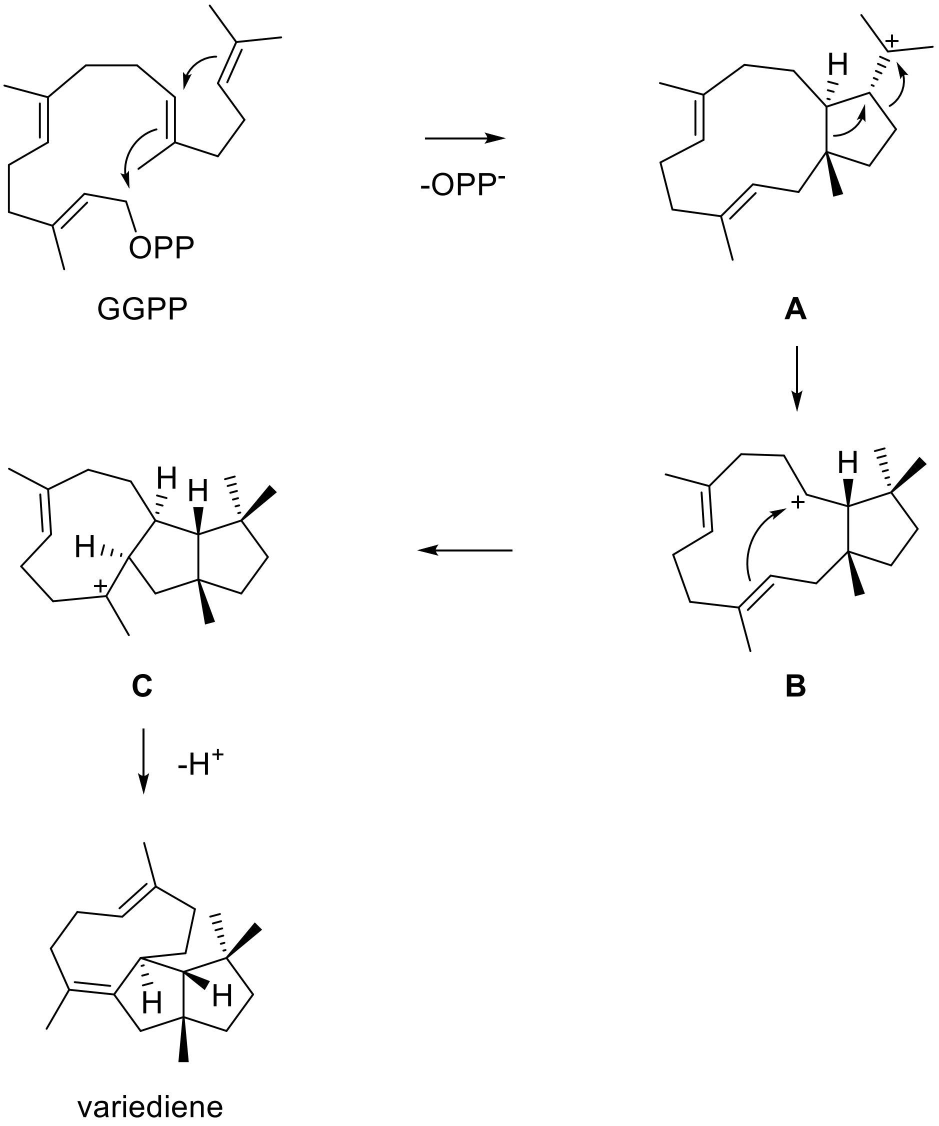

Scheme S5. Cyclisation mechanism from GGPP to variediene by AbVS from *A. brasiliensis*.[10]

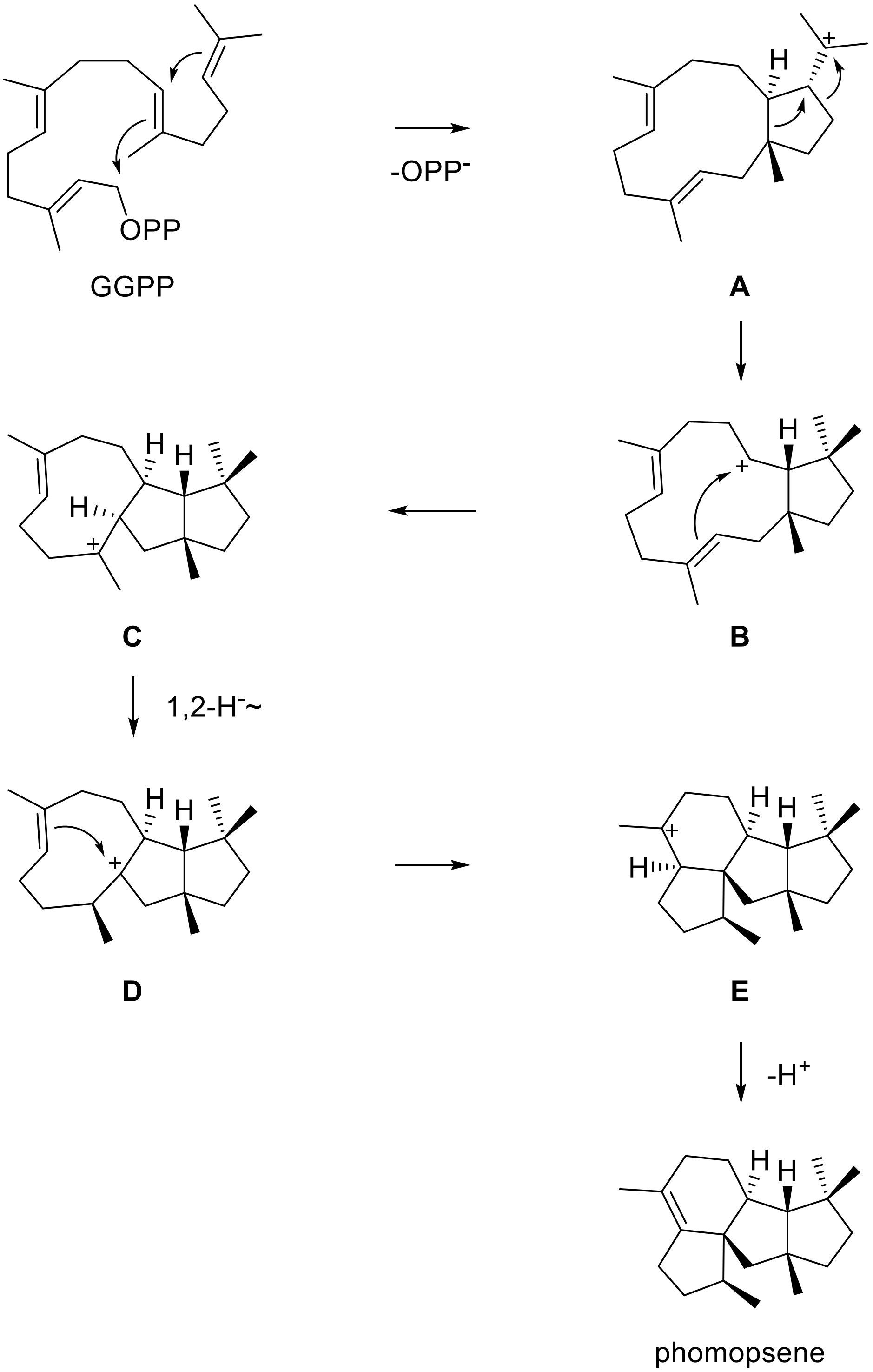

Scheme S6. Cyclisation mechanism from GGPP to phomopsene by NrPS from *N. rhamnosiphilia*.[11]

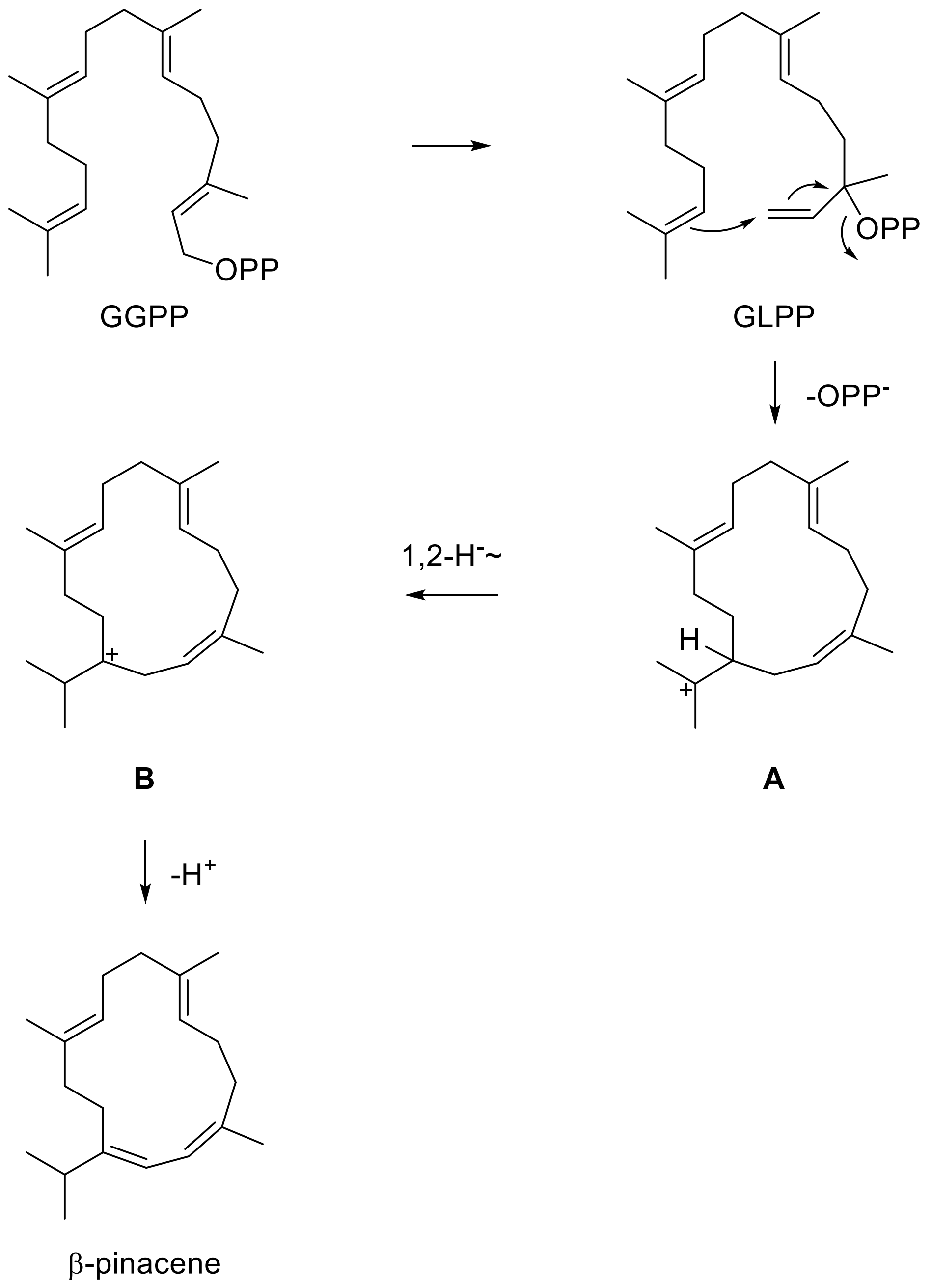

Scheme S7. Cyclisation mechanism from GGPP to β-pinacene by PcS from *D. discoideum*.[12]

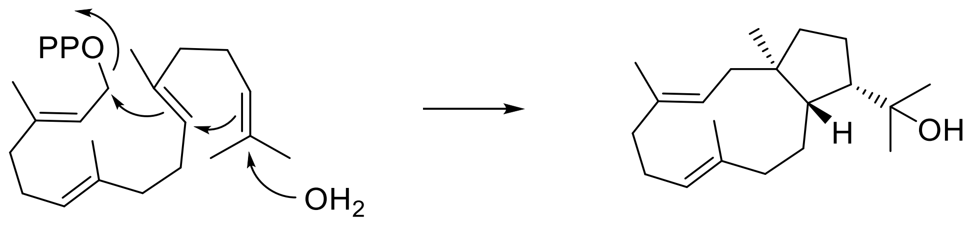

18-hydroxydolabella-3,7-diene

Scheme S8. Cyclisation mechanism from GGPP to 18-hydroxydolabella-3,7-diene by HdS from *C. pinensis*.[13]

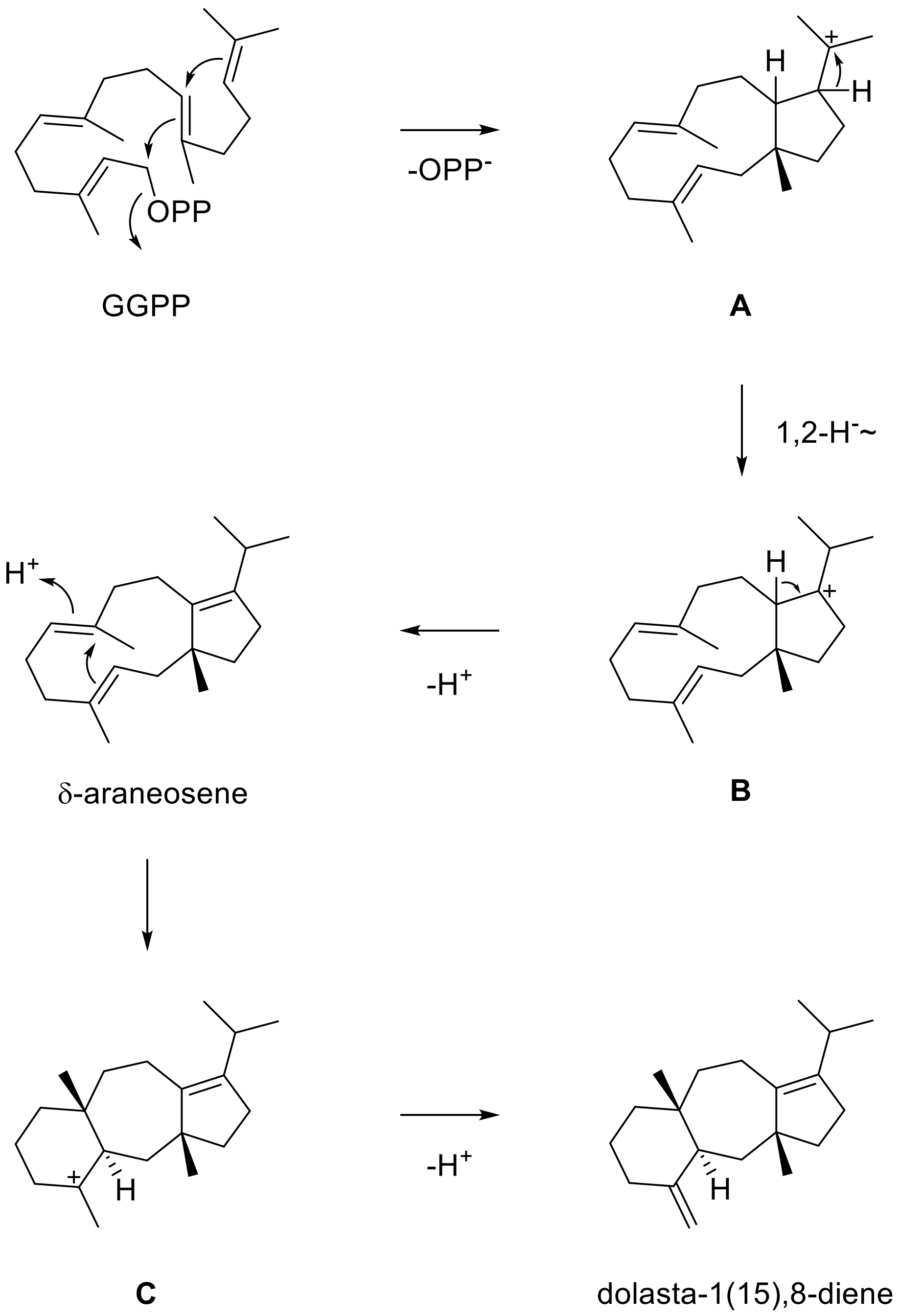

Scheme S9. Cyclisation mechanism from GGPP to dolasta-1(15),8-diene by CgDS from *C. gloeosporioides*.[14]

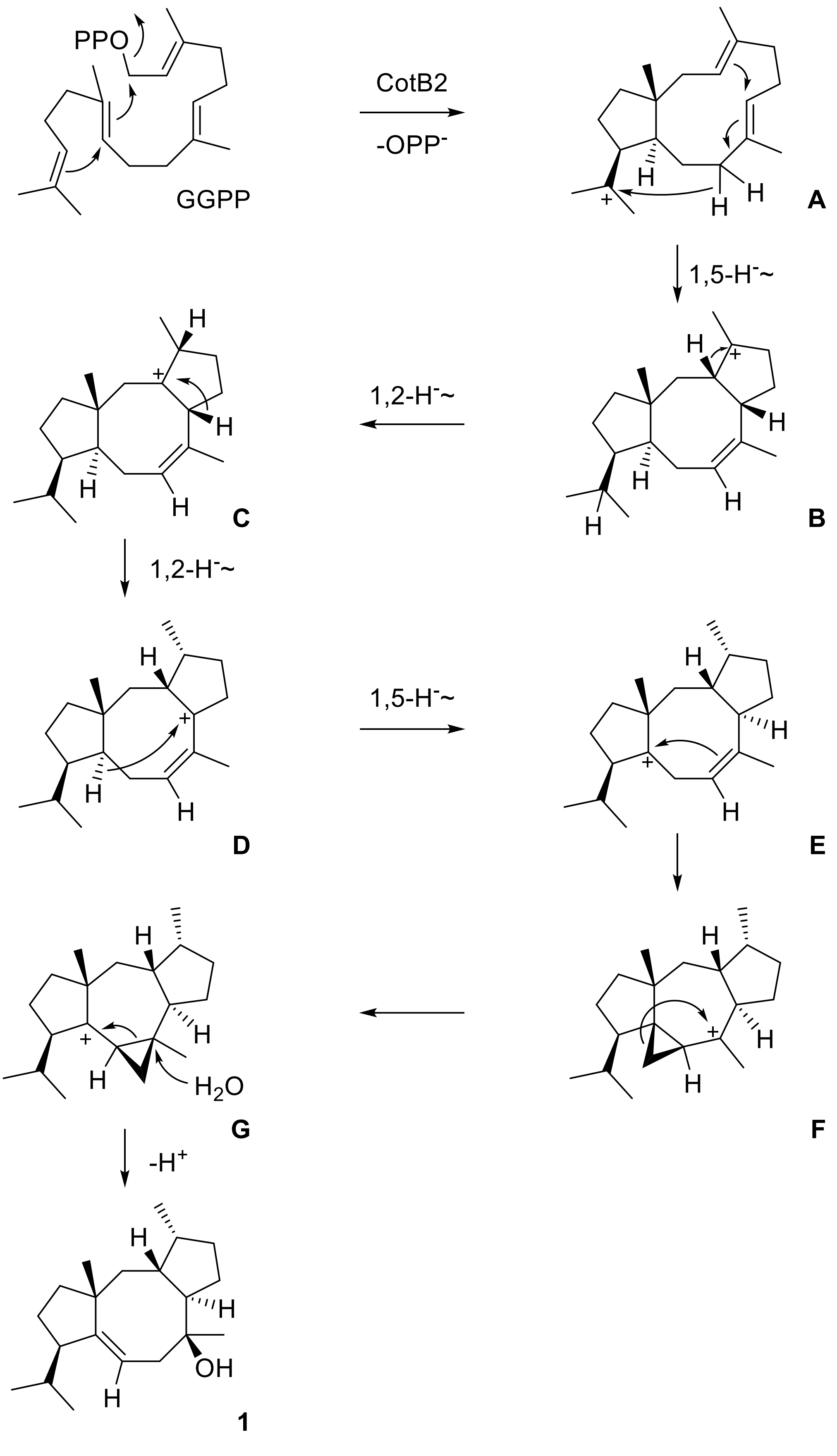

Scheme S10. Cyclisation mechanism from GGPP to cyclooctat-9-en-7-ol by CotB2 from *S. melanosporofaciens*.[15]

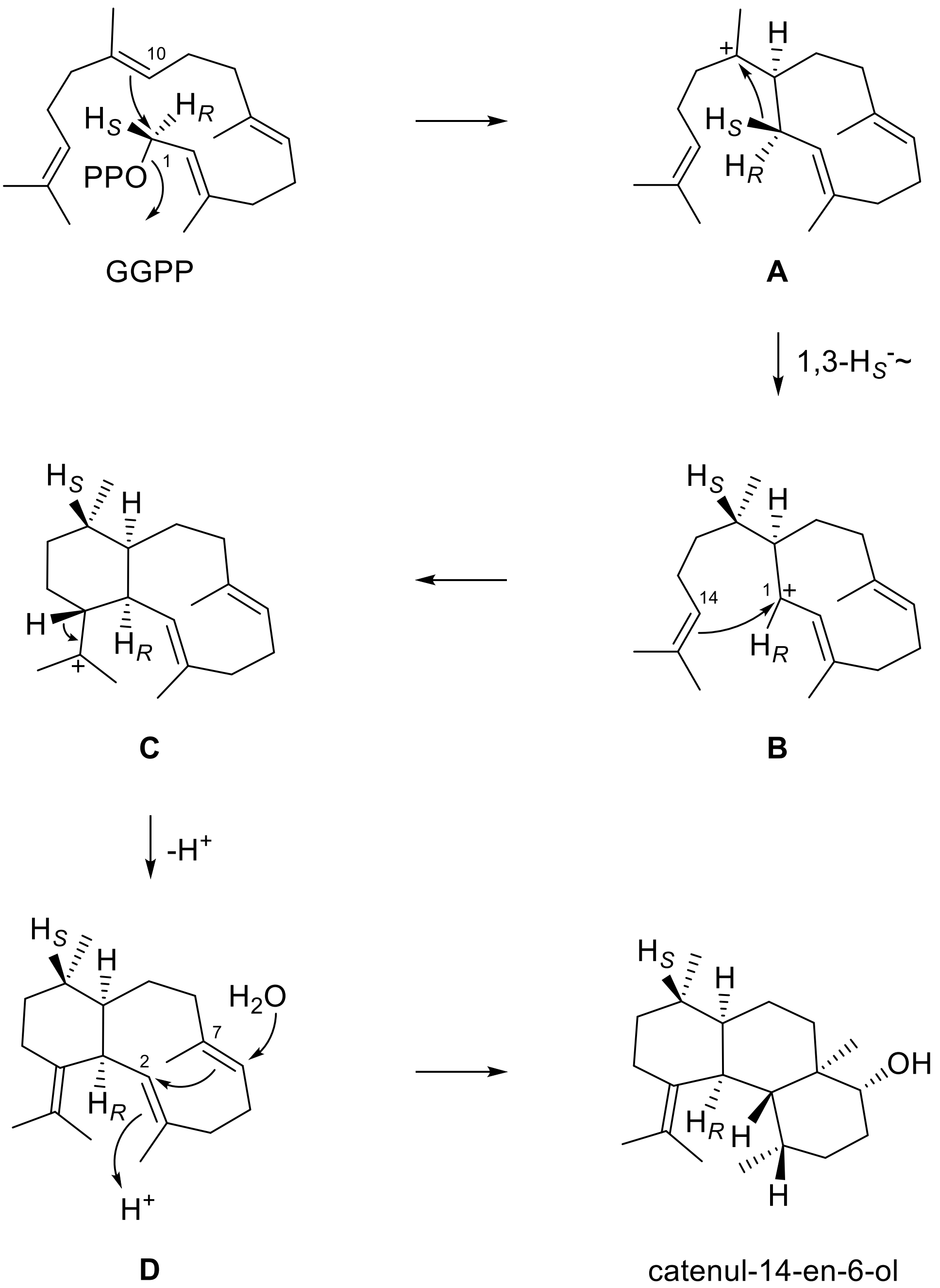

Scheme S11. Cyclisation mechanism from GGPP to catenul-14-en-6-ol by CaCS from *C. acidiphila*.[16]

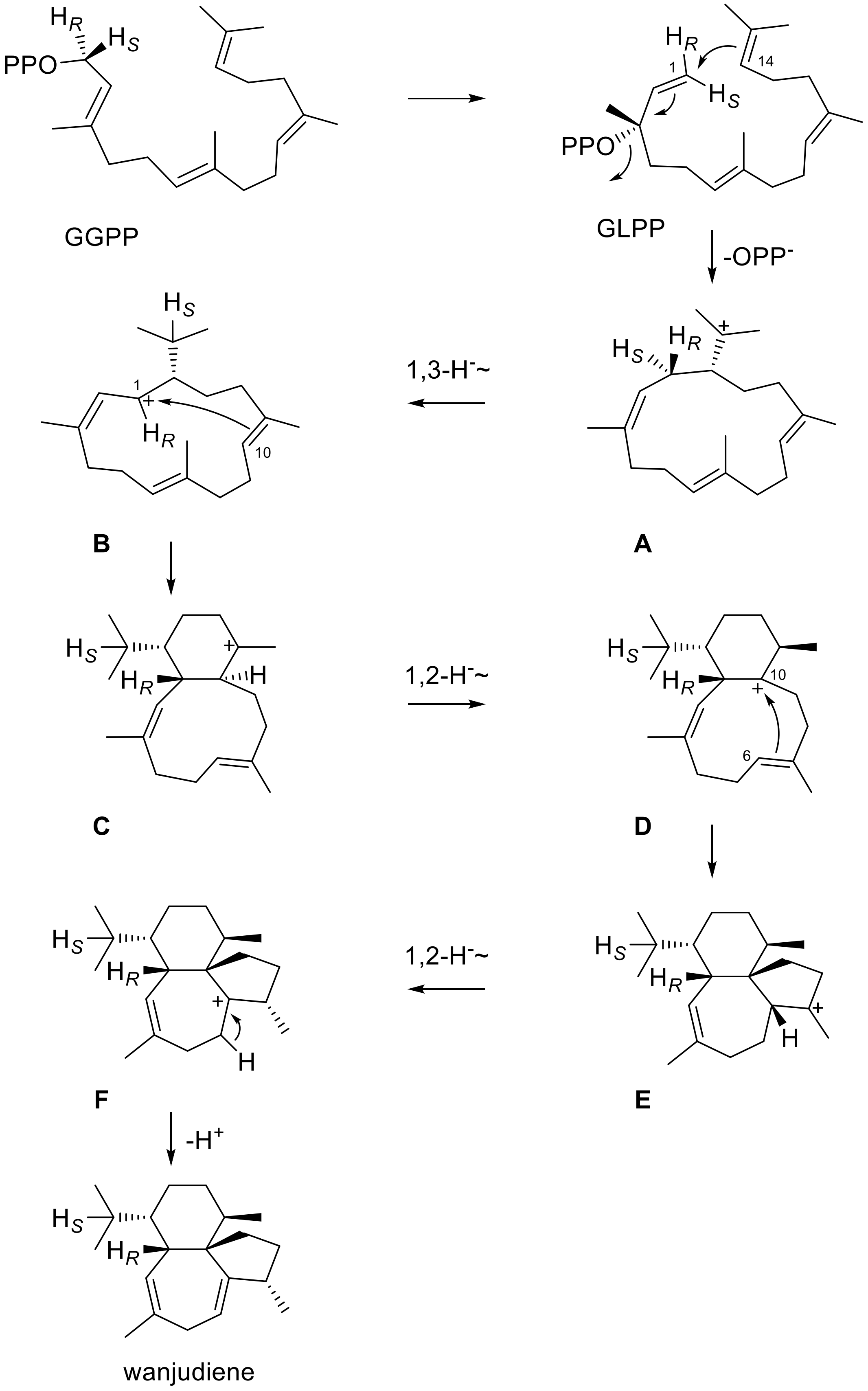

Scheme S12. Cyclisation mechanism from GGPP to wanjudiene by CwWS from *C. wanjuense*.[17]

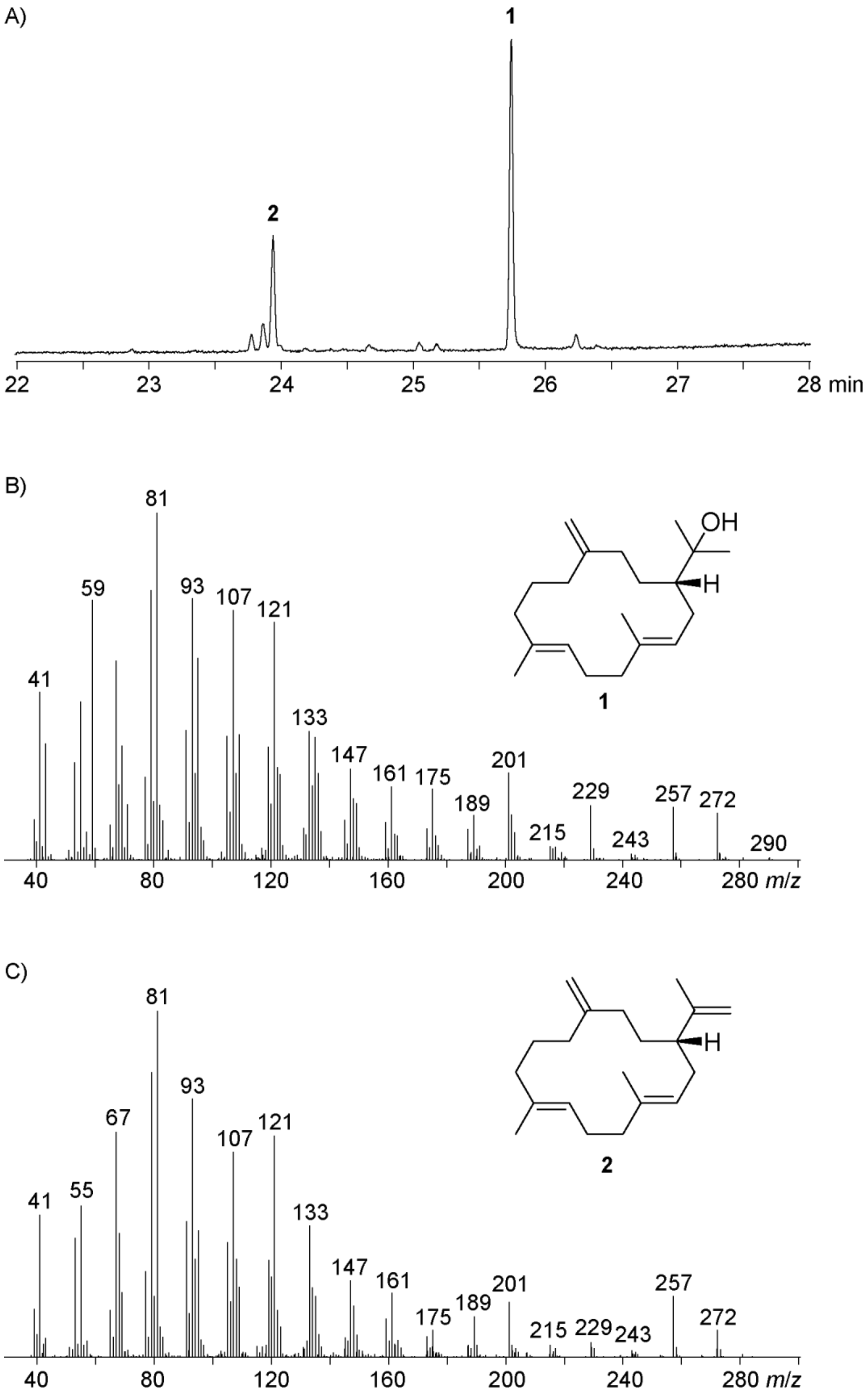

Figure S1. Product mixture formed from iso-FPP and IPP with GGPPS and BdS. A) Total ion chromatogram of the crude extract from the enzyme incubation, B) EI mass spectrum of **1**, and C) EI mass spectrum of **2**.

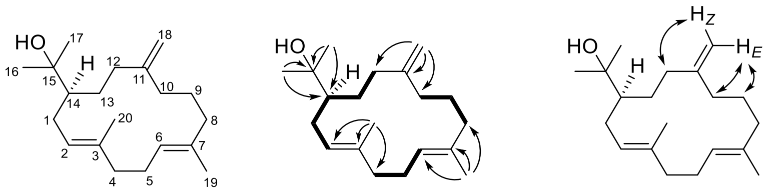

1

Figure S2. Structure elucidation of isonephthenol (**1**). Bold: ^{1}H,^{1}H-COSY, single-headed arrows: key HMBC, and double-headed arrows: key NOESY correlations.

Table S2. NMR data of isonephthenol (**1**) in C_6D_6 recorded at 298 K.

C[a]	type	^{13}C[b]	^{1}H[b]
1	CH_2	30.66	1.84 (dd, ^2J = 14.2, ^3J = 10.2)
			2.17 (m)
2	CH	126.17	5.13 (m)
3	C_q	134.59	–
4	CH_2	39.94	2.07 (m)
			2.13 (m)
5	CH_2	25.19	2.09 (m)
			2.23 (m)
6	CH	125.24	5.07 (m)
7	C_q	133.80	–
8	CH_2	38.29	1.99 (m)
			2.02 (m)
9	CH_2	25.49	1.55 (m, 2H)
10	CH_2	33.26	2.00 (m, 2H)
11	C_q	151.20	–
12	CH_2	37.33	2.22 (m)
			2.32 (dt, ^2J = 13.6, ^3J = 8.3)
13	CH_2	28.86	1.45 (m, 2H)
14	CH	48.99	1.28 (m)
15	C_q	73.48	–
16	CH_3	27.20	1.058 (s)
17	CH_3	28.27	1.061 (s)
18	CH_2	110.06	4.86 (br s, H_E)
			4.90 (br s, H_Z)
19	CH_3	16.57	1.52 (br s)
20	CH_3	15.41	1.51 (br s)

[a] Carbon numbering as shown in Figure S2. [b] Chemical shifts δ in ppm, multiplicity: s = singlet, d = doublet, t= triplet, m = multiplet, br = broad, coupling constants J are given in Hertz.

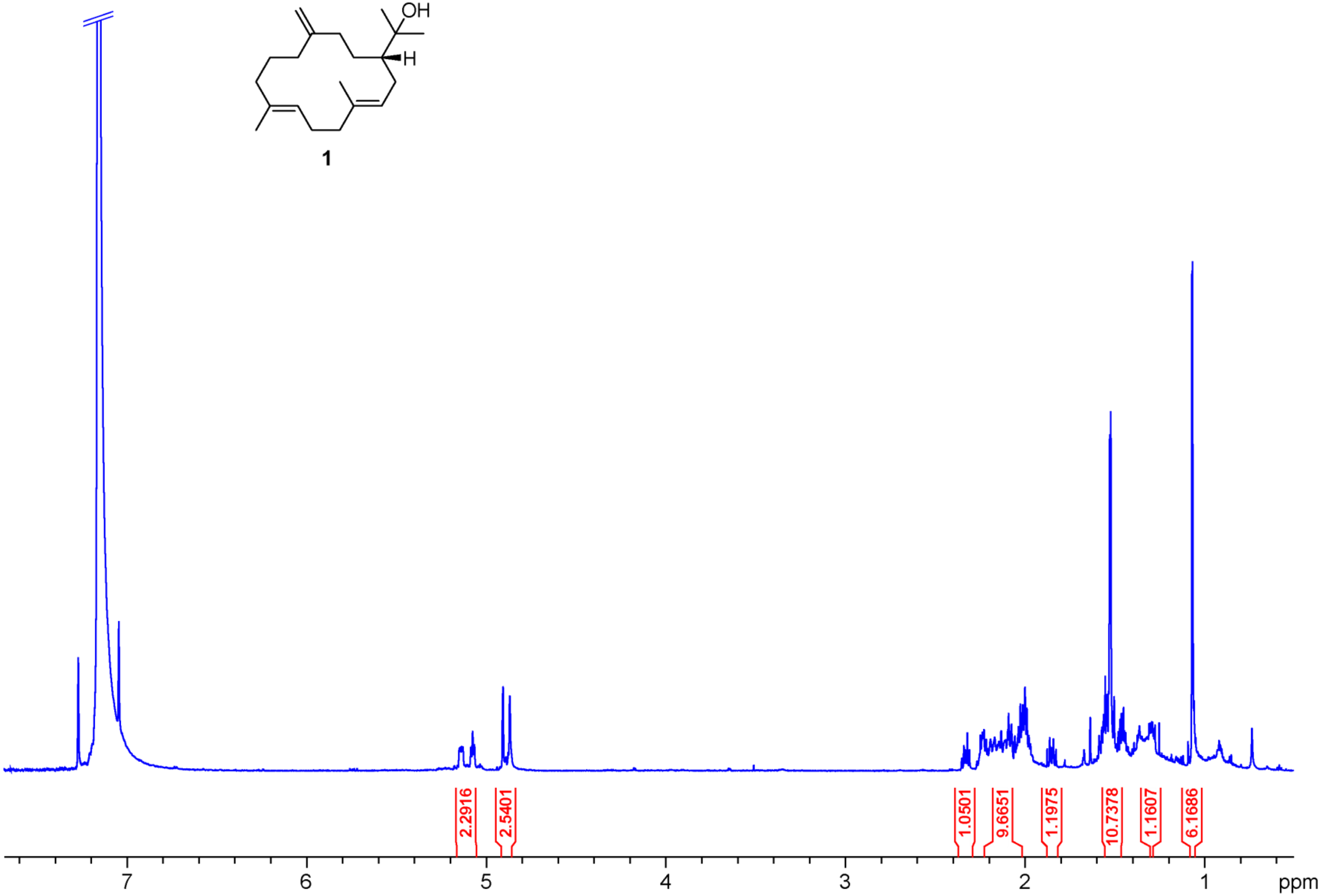

Figure S3. ^{1}H-NMR spectrum (700 MHz, C_6D_6) of **1**.

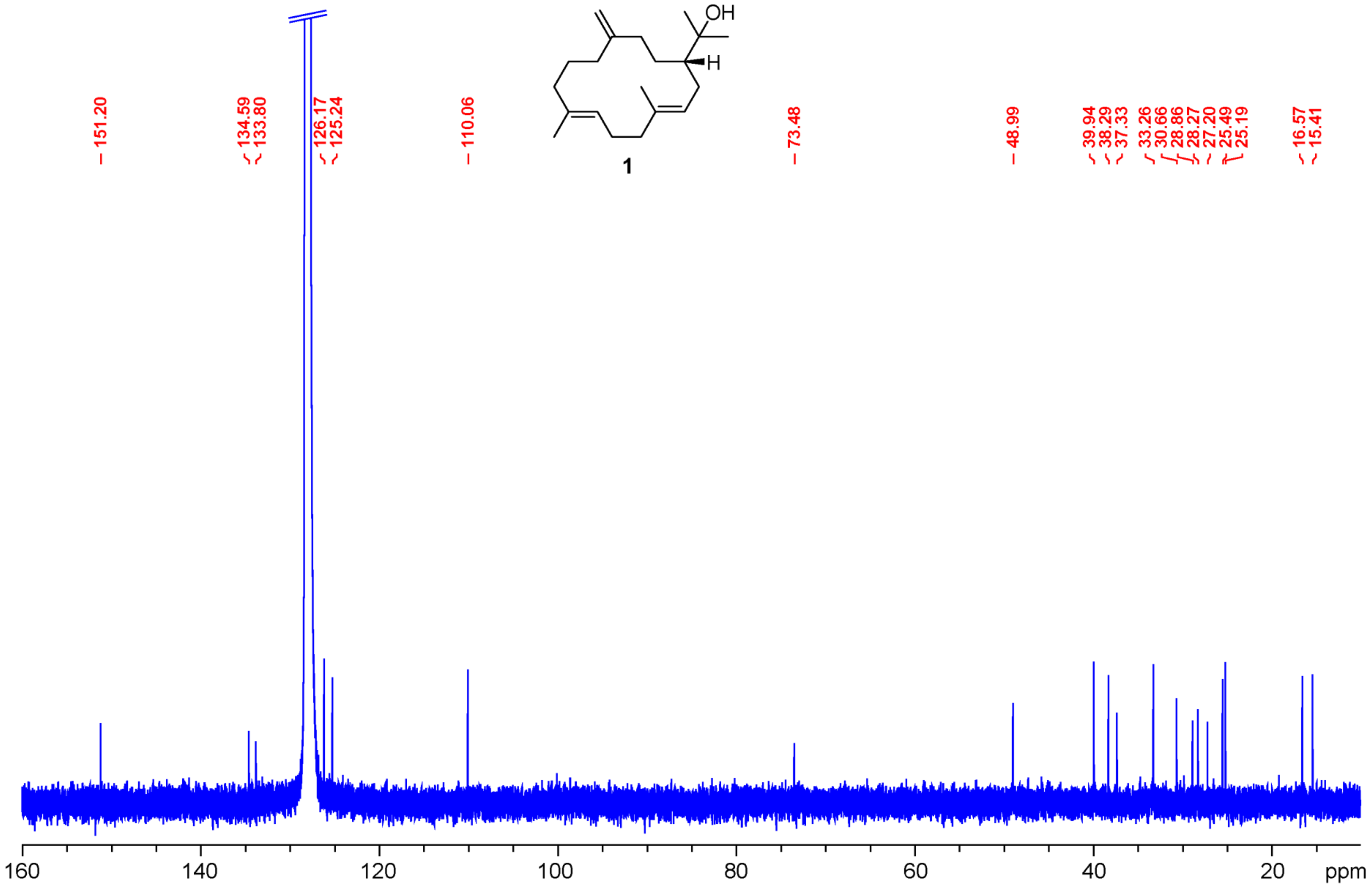

Figure S4. ^{13}C-NMR spectrum (176 MHz, C_6D_6) of **1**.

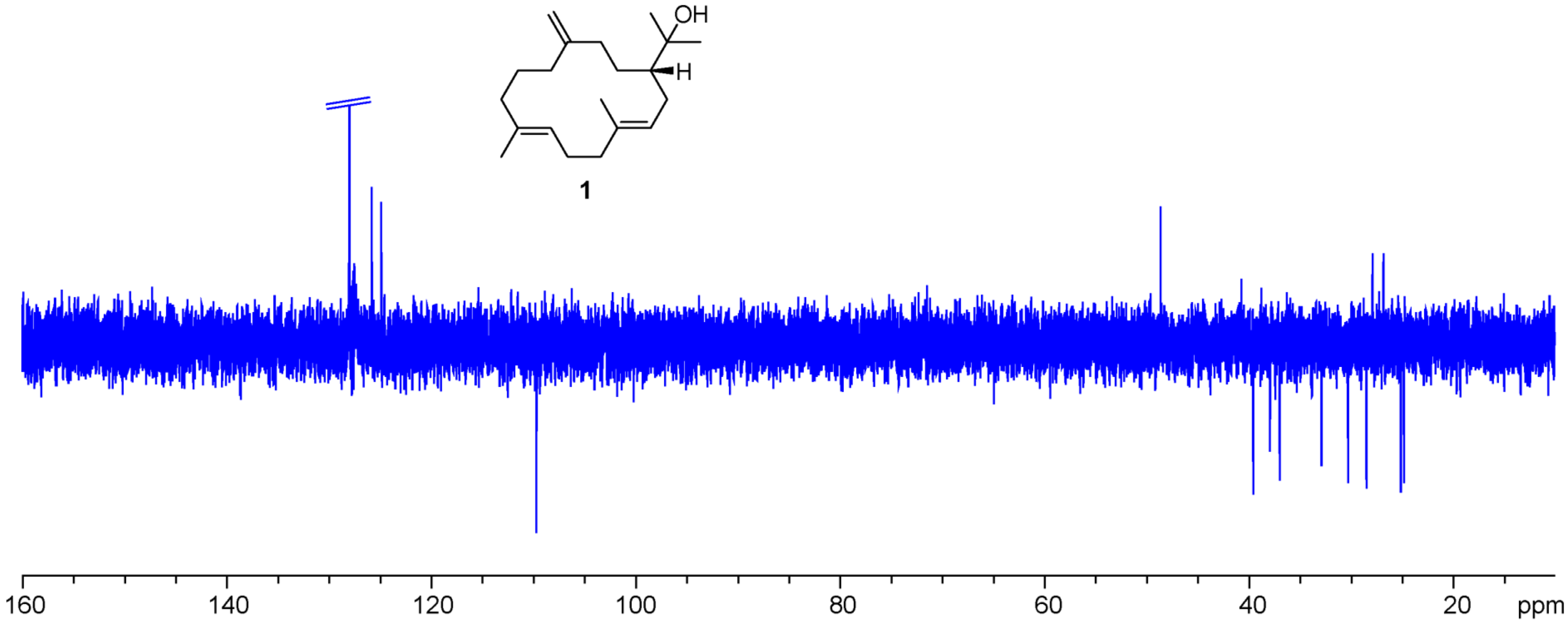

Figure S5. ^{13}C-DEPT135 spectrum (176 MHz, C_6D_6) of **1**.

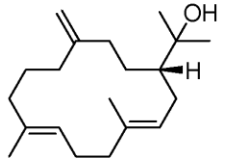

1

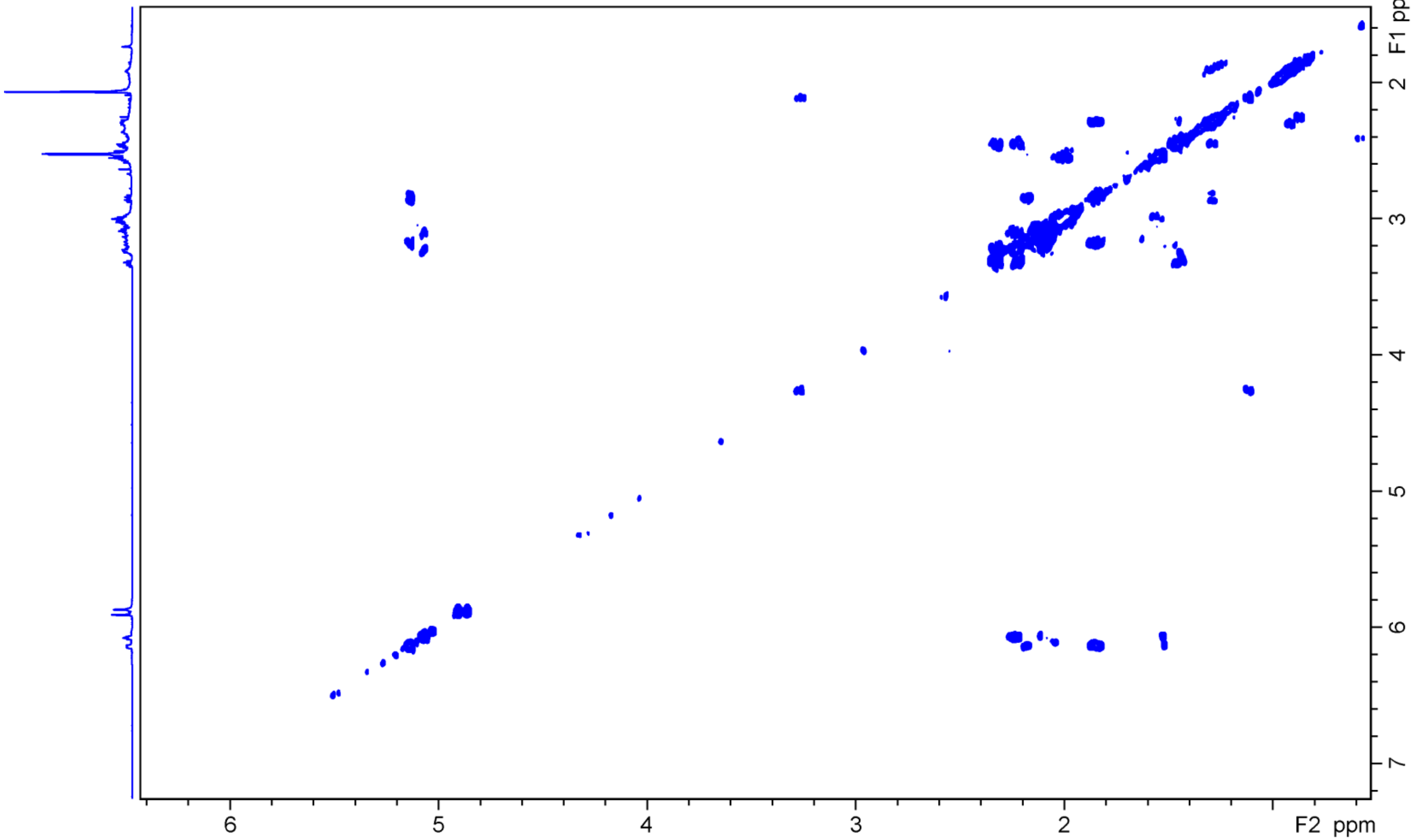

Figure S6. $^{1}H,^{1}H$-COSY spectrum (C_6D_6) of **1**.

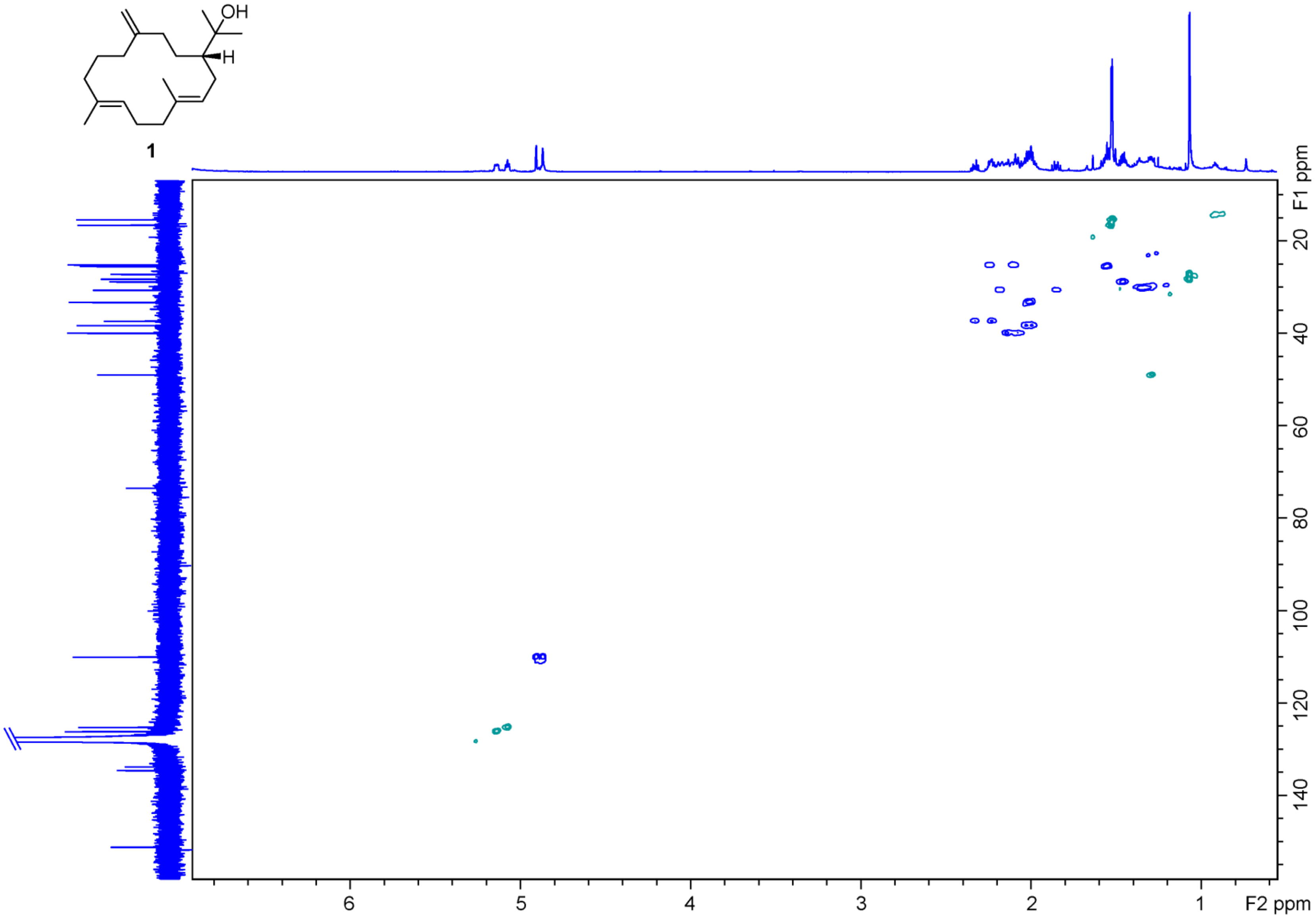

Figure S7. HSQC spectrum (C_6D_6) of **1**.

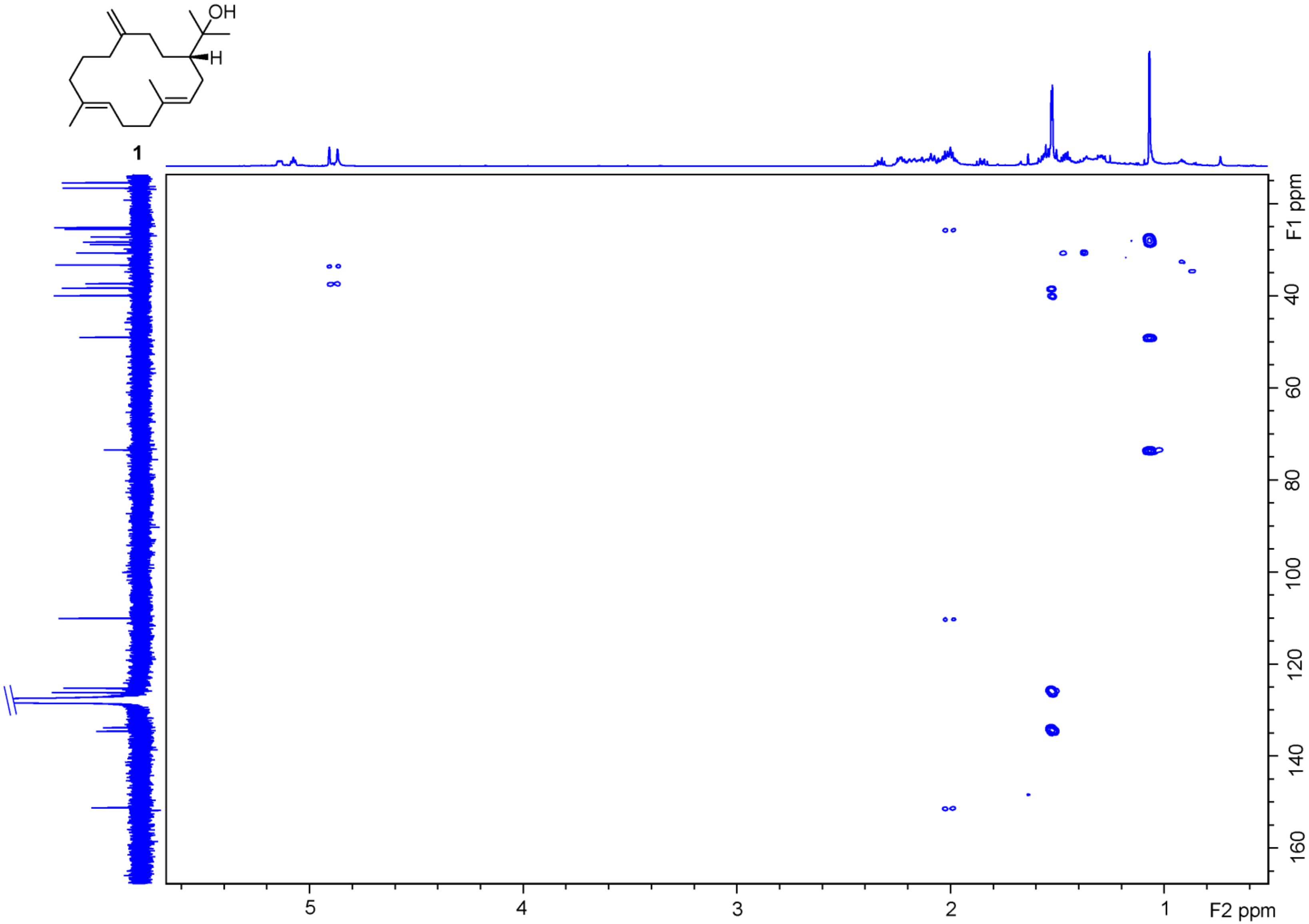

Figure S8. HMBC spectrum (C_6D_6) of **1**.

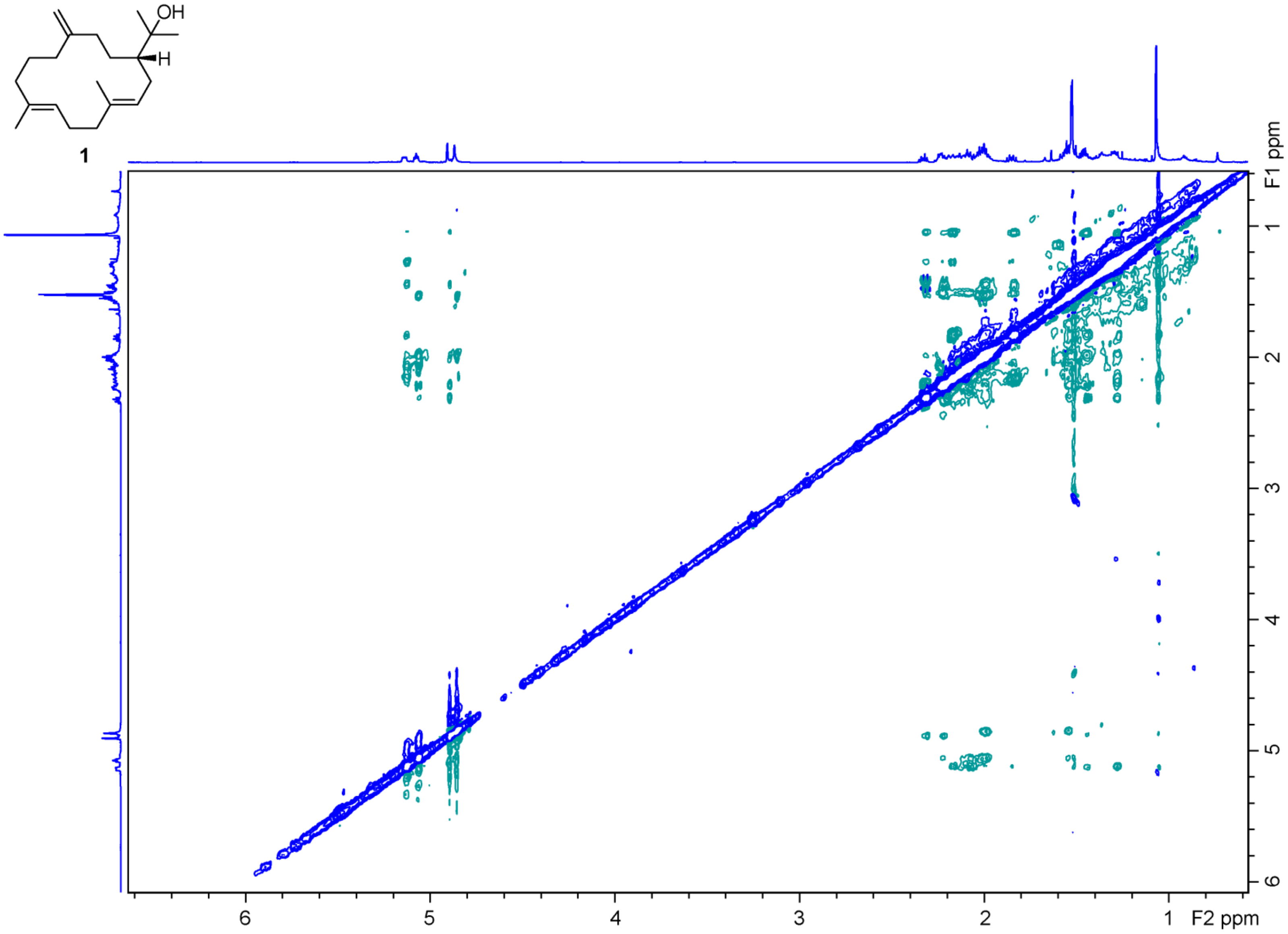

Figure S9. NOESY spectrum (C_6D_6) of **1**.

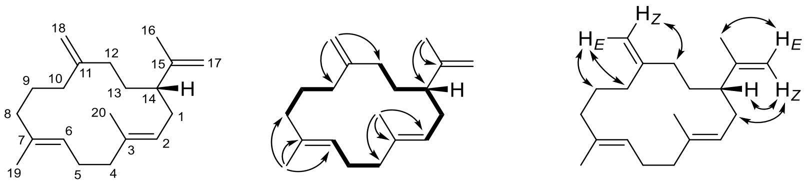

2

Figure S10. Structure elucidation of isocembrene A (**2**). Bold: ^{1}H,^{1}H-COSY, single-headed arrows: key HMBC, and double-headed arrows: key NOESY correlations.

Table S3. NMR data of isocembrene A (**2**) in C_6D_6 recorded at 298 K.

C[a]	type	^{13}C[b]	^{1}H[b]
1	CH_2	34.17	2.03 (m, 2H)
2	CH	125.37	5.10 (m)
3	C_q	135.26	–
4	CH_2	40.06	2.03 (m)
			2.12 (m)
5	CH_2	25.50	2.09 (m)
			2.23 (m)
6	CH	125.55	5.03 (m)
7	C_q	133.79	–
8	CH_2	39.03	1.95 (m)
			1.99 (m)
9	CH_2	24.20	1.44 (m)
			1.52 (m)
10	CH_2	33.02	1.96 (m)
			2.01 (m)
11	C_q	149.26	–
12	CH_2	33.35	2.14 (m)
			2.18 (m)
13	CH_2	26.53	1.15 (m)
			1.60 (m)
14	CH	45.72	2.00 (m)
15	C_q	148.28	–
16	CH_3	19.21	1.63 (q, ^{4}J = 0.8)
17	CH_2	111.08	4.87 (m, H_E)
			4.90 (m, H_Z)
18	CH_2	111.14	4.85 (m, H_Z)
			4.88 (m, H_E)
19	CH_3	16.65	1.53 (br s)
20	CH_3	15.36	1.49 (br s)

[a] Carbon numbering as shown in Figure S10. [b] Chemical shifts δ in ppm, multiplicity: s = singlet, q = quartet, m = multiplet, br = broad, coupling constants J are given in Hertz.

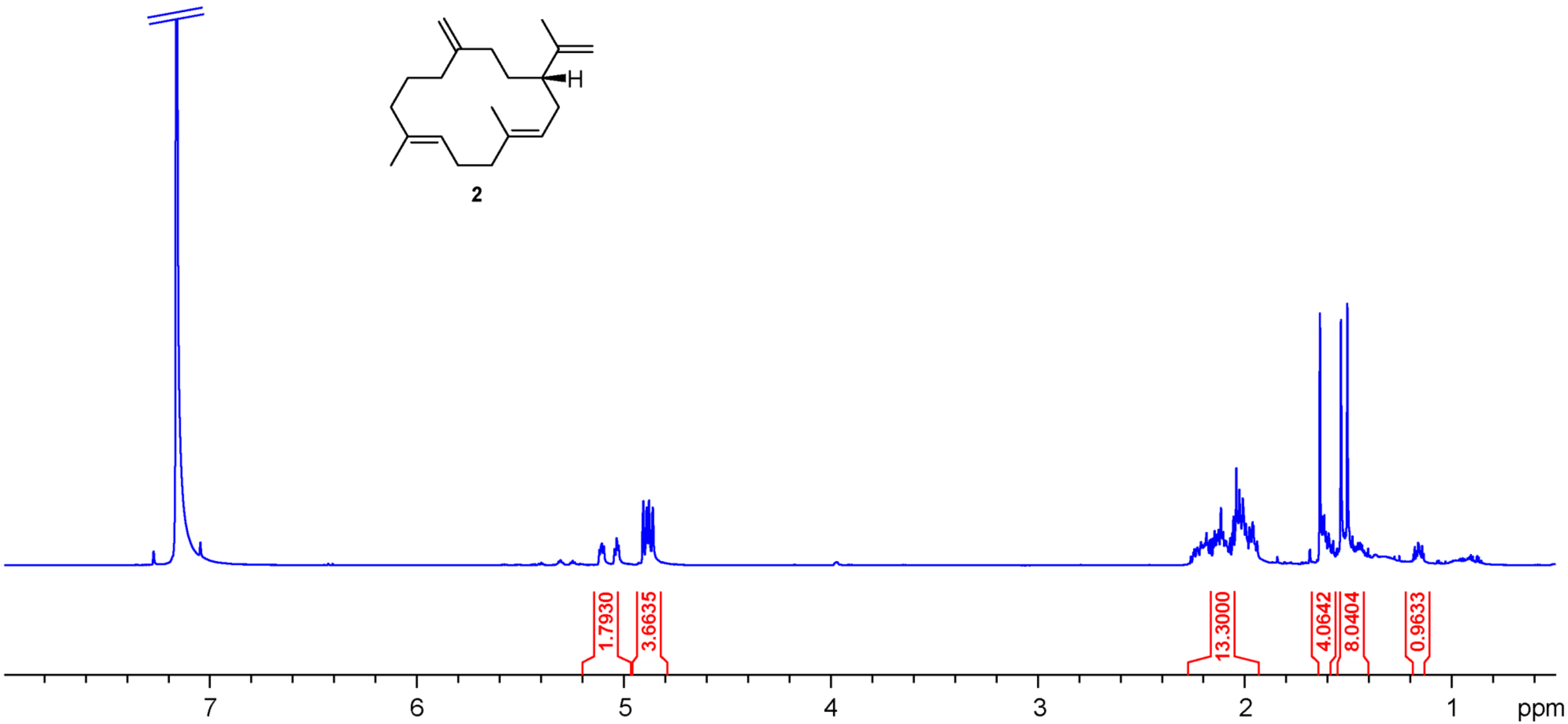

Figure S11. ^{1}H-NMR spectrum (700 MHz, C_6D_6) of **2**.

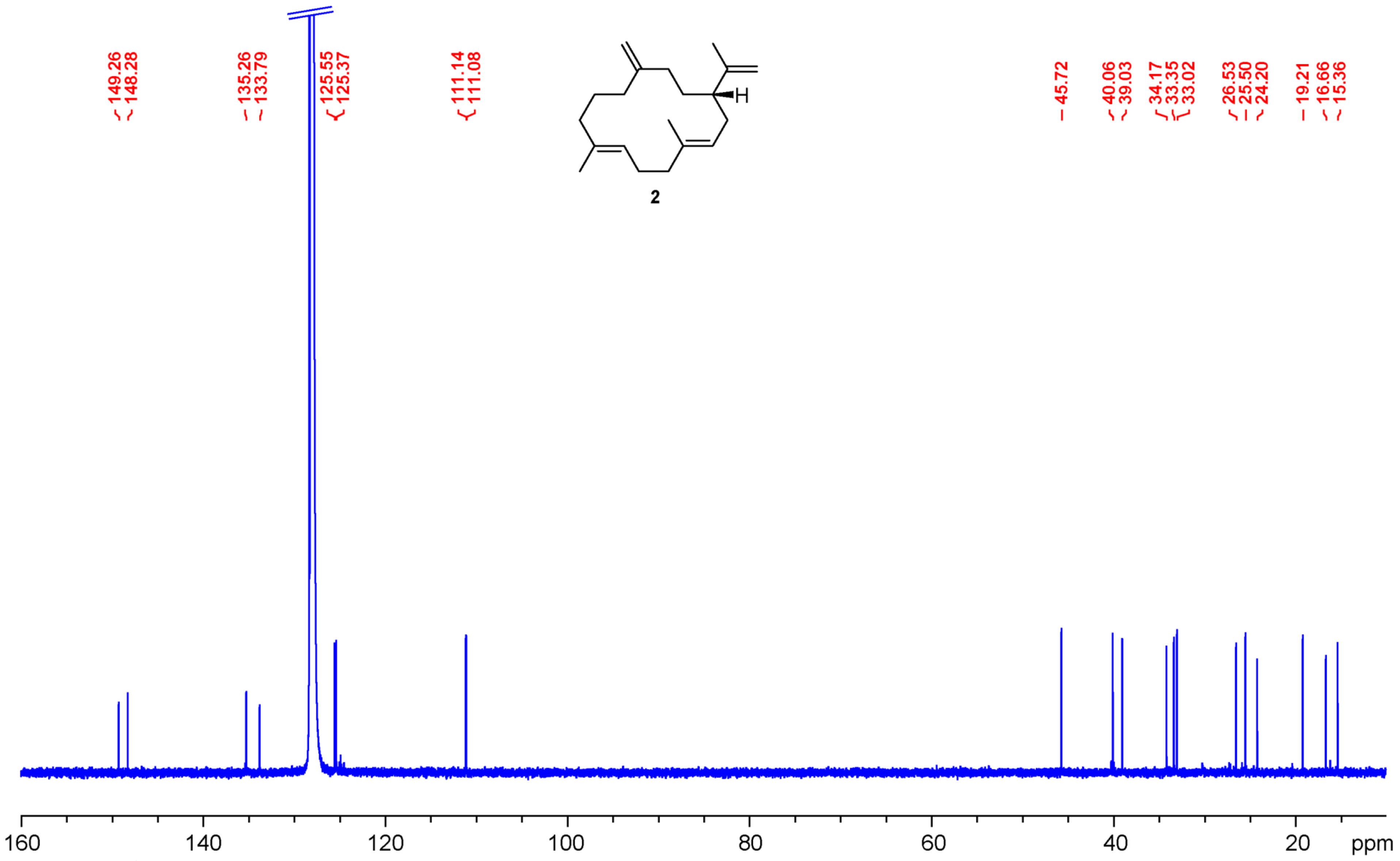

Figure S12. ^{13}C-NMR spectrum (176 MHz, C_6D_6) of **2**.

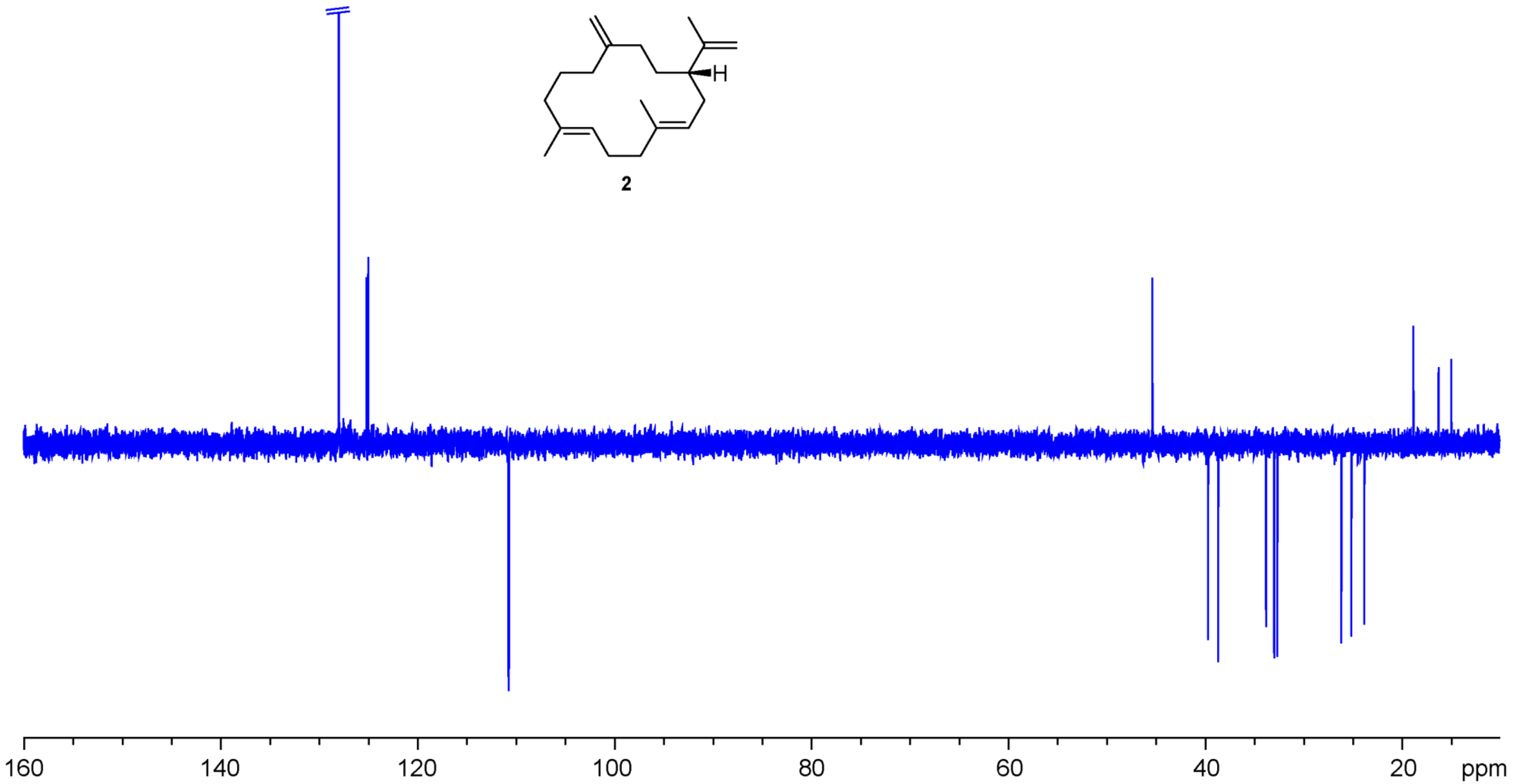

Figure S13. ^{13}C-DEPT135 spectrum (176 MHz, C_6D_6) of **2**.

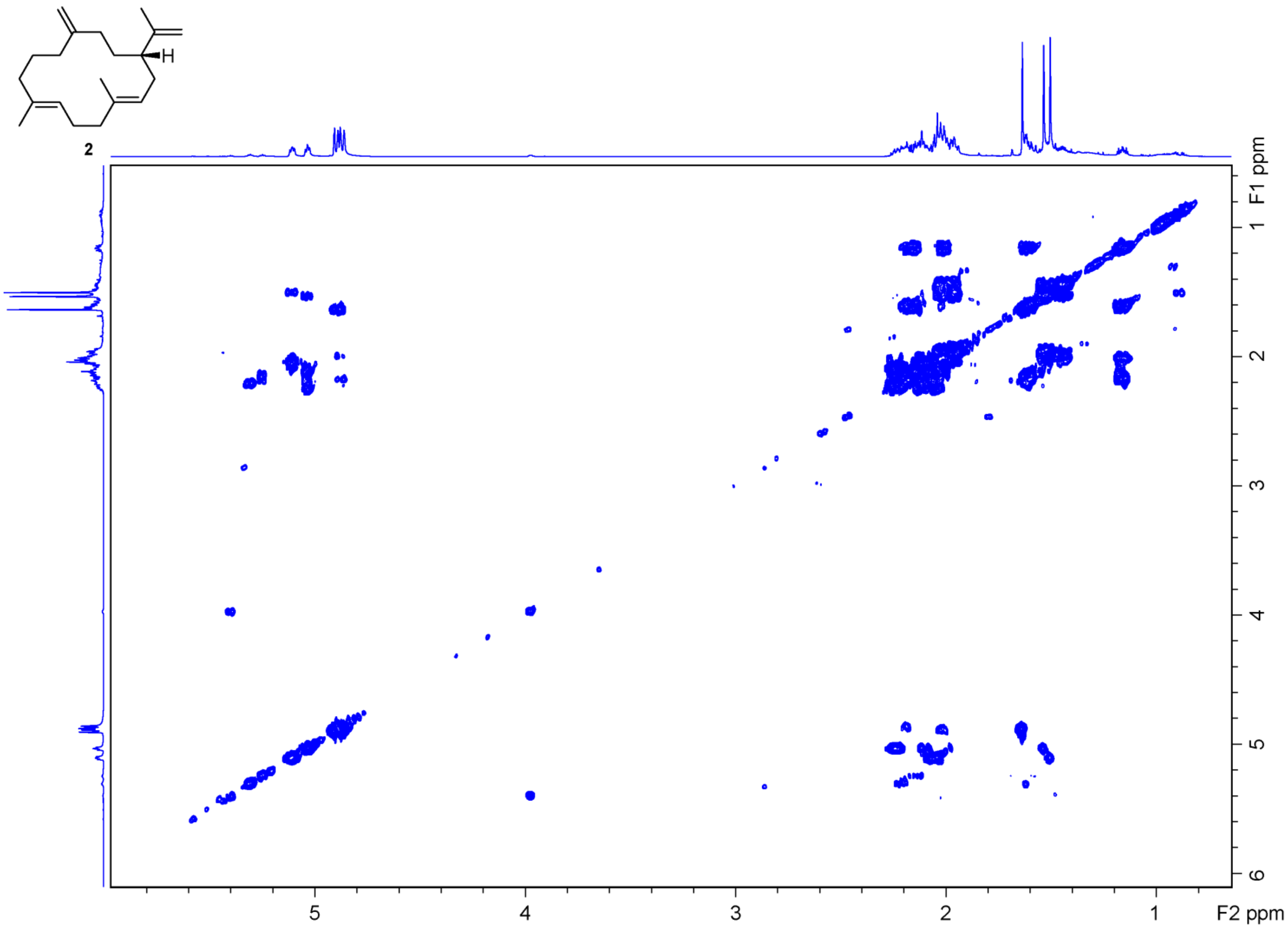

Figure S14. $^{1}H,^{1}H$-COSY spectrum (C_6D_6) of **2**.

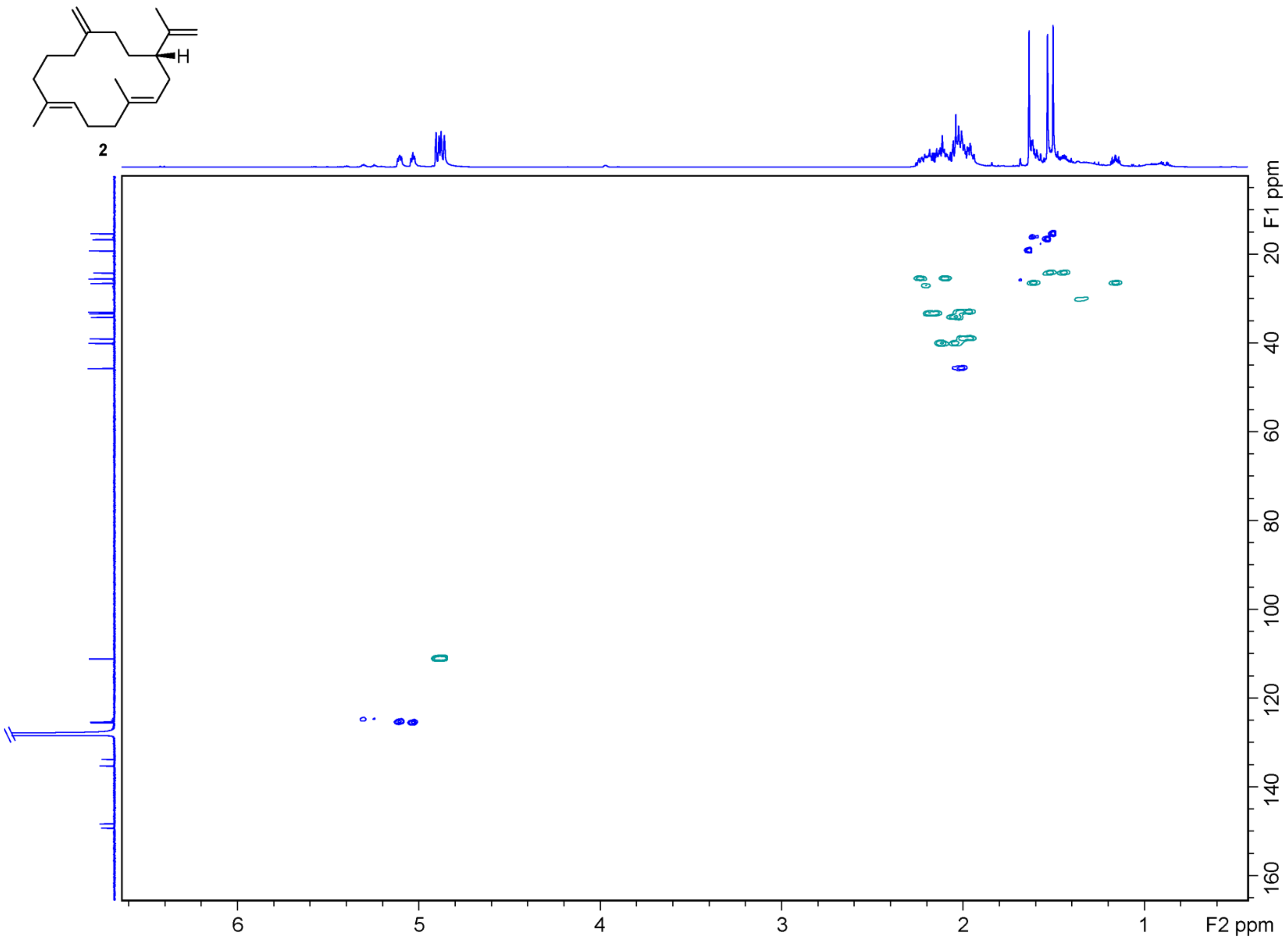

Figure S15. HSQC spectrum (C_6D_6) of **2**.

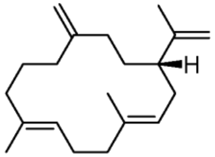

2

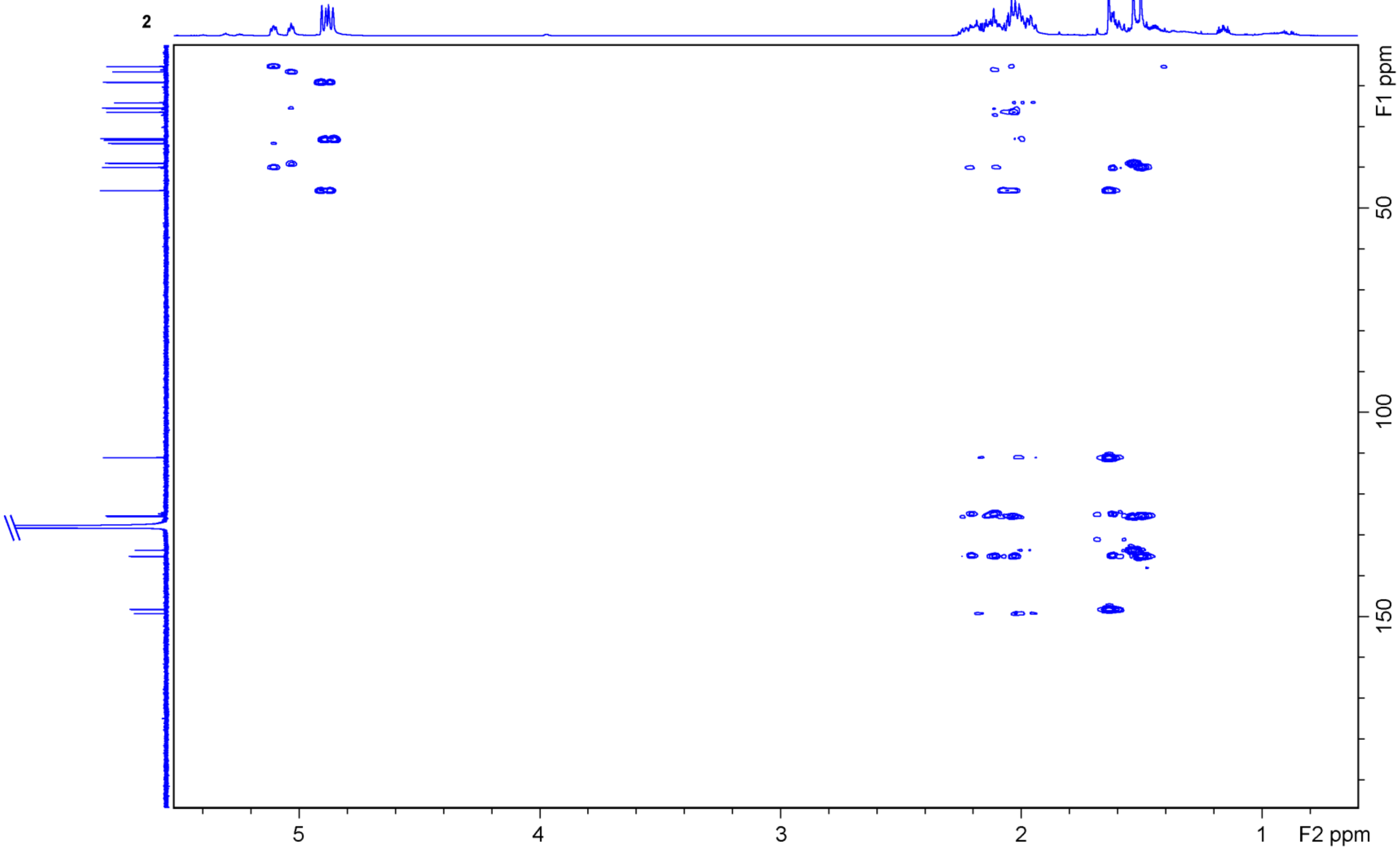

Figure S16. HMBC spectrum (C_6D_6) of **2**.

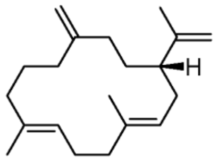

2

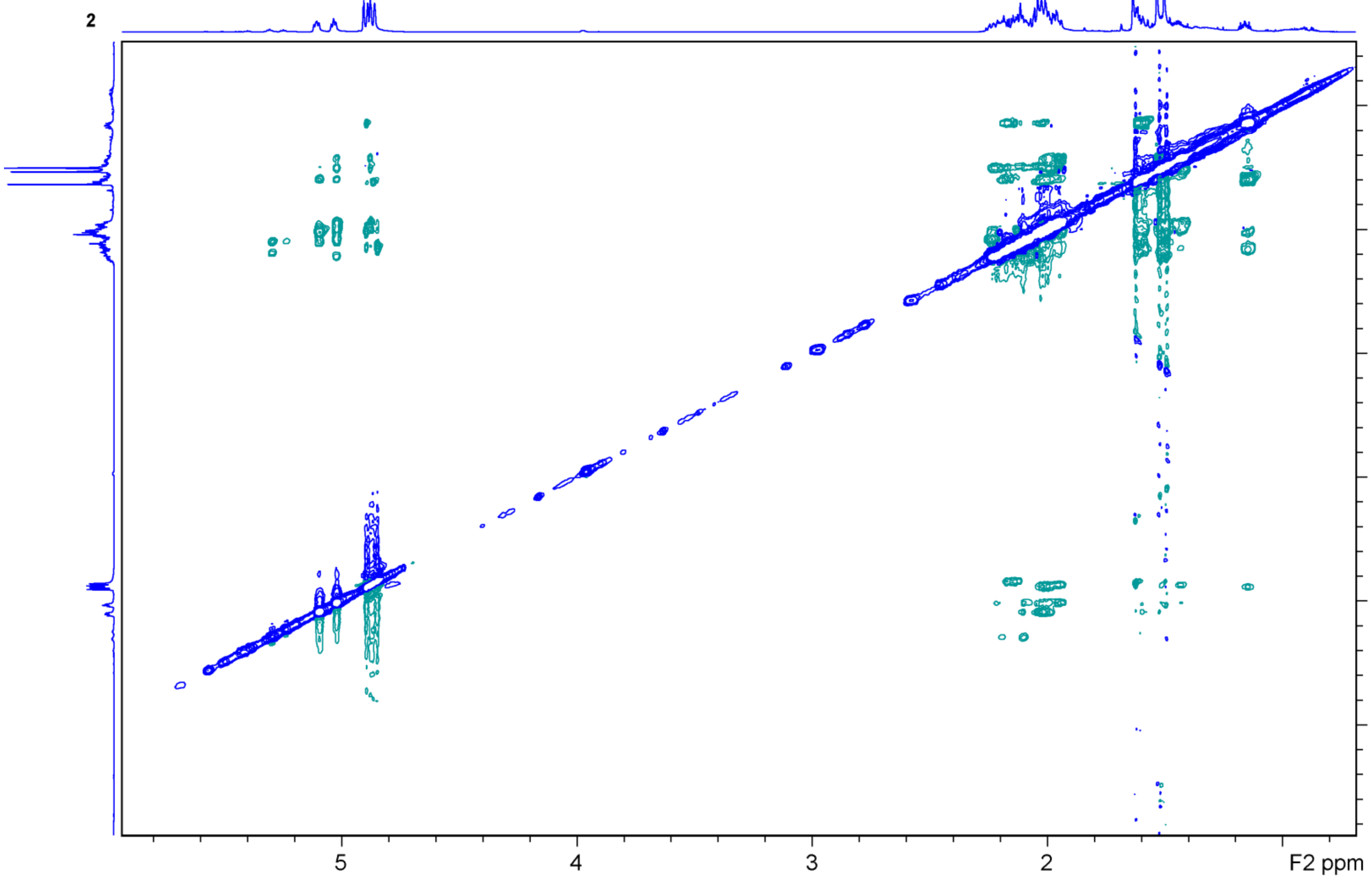

Figure S17. NOESY spectrum (C_6D_6) of **2**.

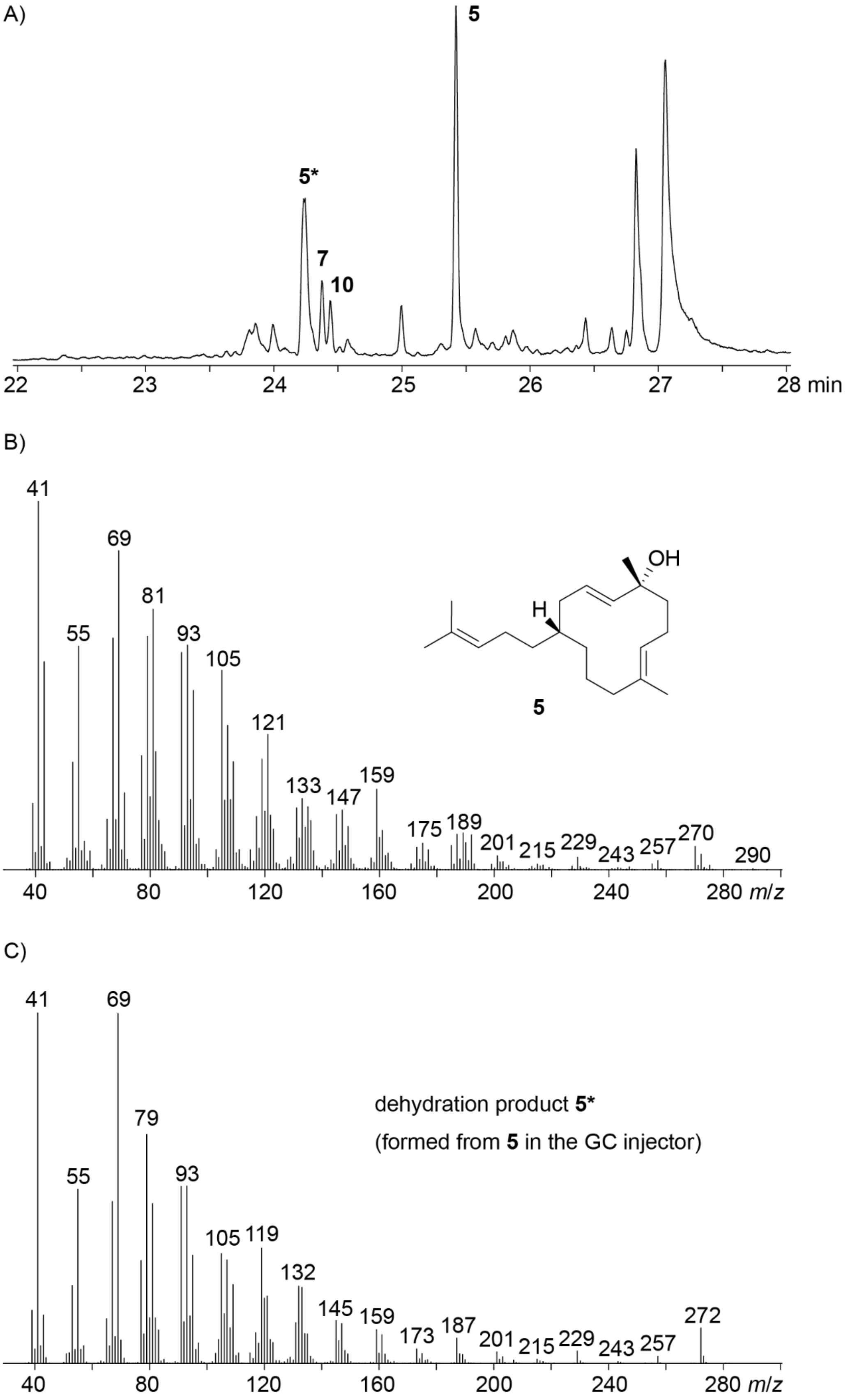

Figure S18. Product mixture formed from iso-FPP and IPP with GGPPS and SpS. A) Total ion chromatogram of the crude extract from the enzyme incubation, B) EI mass spectrum of **5**, C) EI mass spectrum of a dehydration product **5*** presumably formed from **5** in the GC injector. This compound is also observed upon injection of purified **5**. The minor compounds **7** and **10** are explained below.

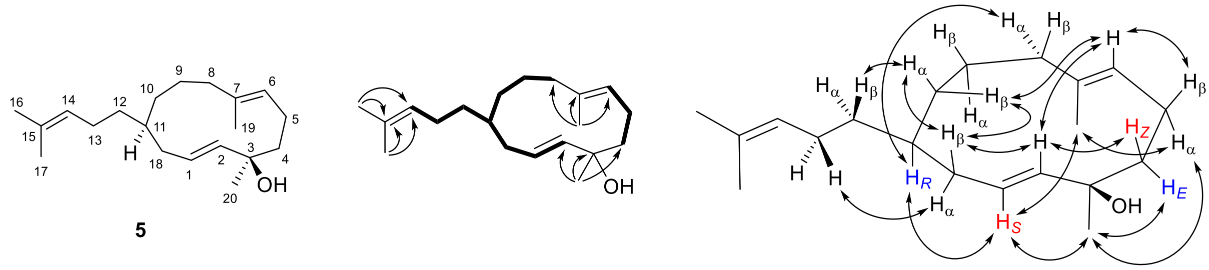

Figure S19. Structure elucidation of pseudoobscuronatin (**5**). Bold: ^{1}H,^{1}H-COSY correlations, single-headed arrows: key HMBC, and double-headed arrows: key NOESY correlations.

Table S4. NMR data of pseudoobscuronatin (**5**) in C_6D_6 recorded at 298 K.

C[a]	type	^{13}C[b]	^{1}H[b]
1	CH	124.89	5.40 (ddd, ^{2}J = 15.8, ^{3}J = 8.5, 6.0, H_S)
2	CH	14041	5.35 (dd, ^{2}J = 15.8, ^{4}J = 0.7)
3	C_q	72.32	–
4	CH_2	43.43	1.64 (m, H_E)
			1.58 (m, H_Z)
5	CH_2	23.89	2.27 (m, H_α)
			2.02 (m, H_β)
6	CH	128.54	5.08 (dd, ^{3}J = 7.2, 7.2)
7	C_q	132.15	–
8	CH_2	37.52	1.94 (m, H_α)
			1.90 (m, H_β)
9	CH_2	21.98	1.33 (m, 2H)
10	CH_2	28.66	1.33 (m, H_α)
			1.10 (m, H_β)
11	CH	36.10	1.40 (m, H_R)
12	CH_2	35.14	1.42 (m, H_β)
			1.21 (m, H_α)
13	CH_2	26.36	2.08 (m, 2H)
14	CH	125.56	5.25 (thept, ^{3}J = 7.1, ^{4}J = 1.4)
15	C_q	131.08	–
16	CH_3	25.92	1.71 (d, ^{4}J = 0.9)
17	CH_3	17.82	1.60 (br s)
18	CH_2	36.21	2.15 (m, H_β)
			1.64 (m, H_α)
19	CH_3	16.67	1.52 (br s)
20	CH_3	29.58	1.16 (s)

[a] Carbon numbering as shown in Figure S19. [b] Chemical shifts δ in ppm, multiplicity: s = singlet, d = doublet, t = triplet, hept = heptet, m = multiplet, br = broad, coupling constants J are given in Hertz. Hydrogens coloured in red and blue show deuterium incorporation from (*R*)- or (*S*)-(1-^{13}C,1-^{2}H)IPP, or (*E*)- or (*Z*)-(4-^{13}C,4-^{2}H)IPP, respectively.

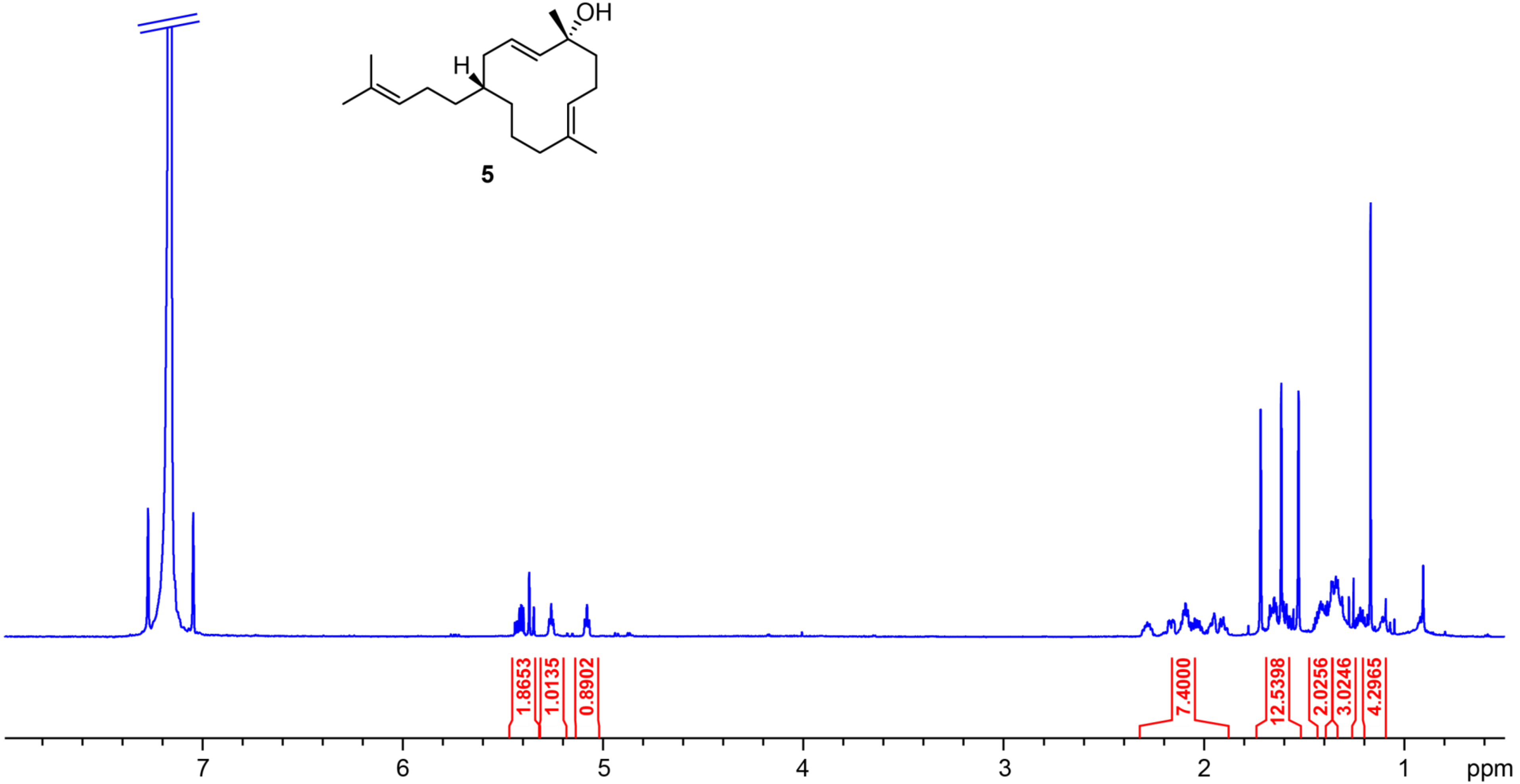

Figure S20. ^{1}H-NMR spectrum (700 MHz, C_6D_6) of **5**.

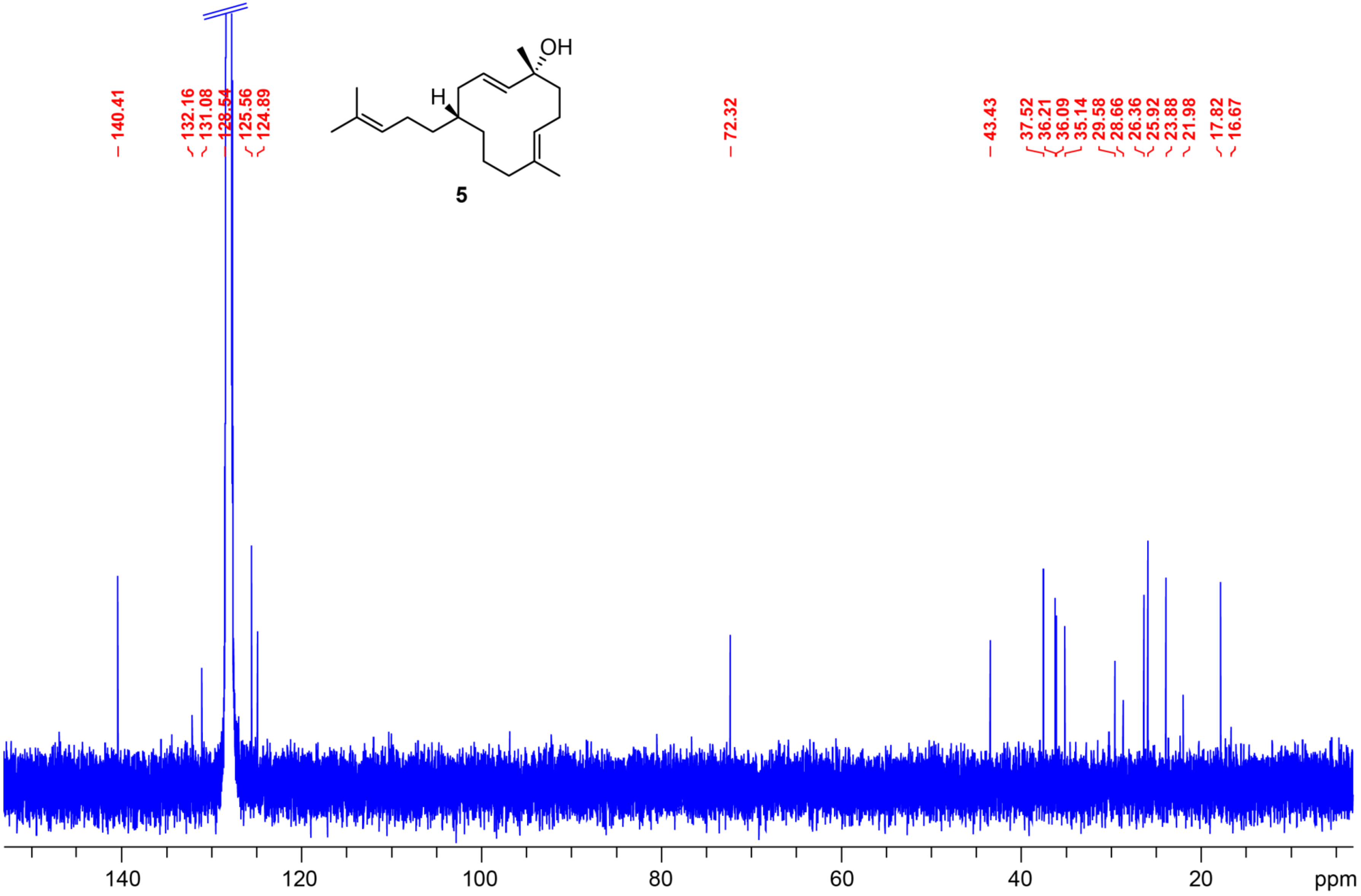

Figure S21. ^{13}C-NMR spectrum (176 MHz, C_6D_6) of **5**.

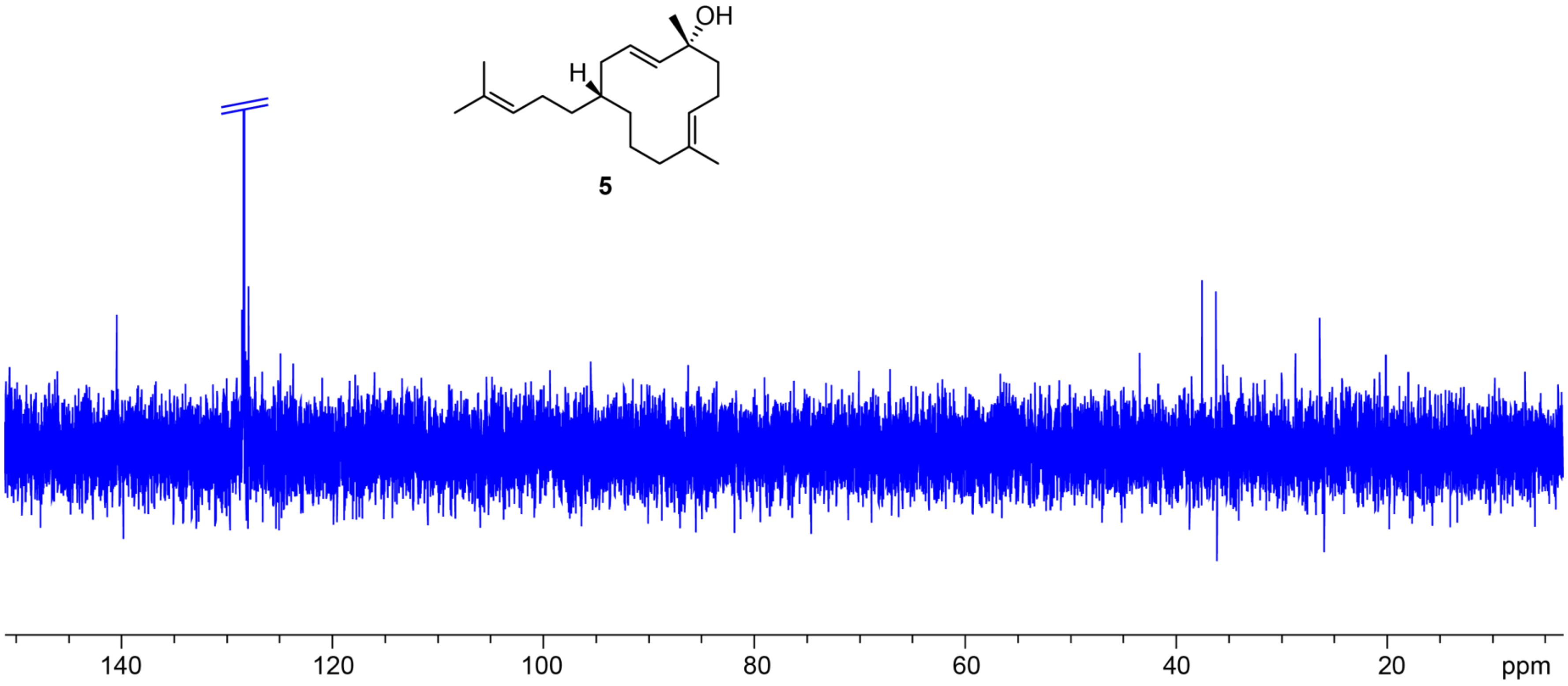

Figure S22. ^{13}C-DEPT135 spectrum (176 MHz, C_6D_6) of **5**.

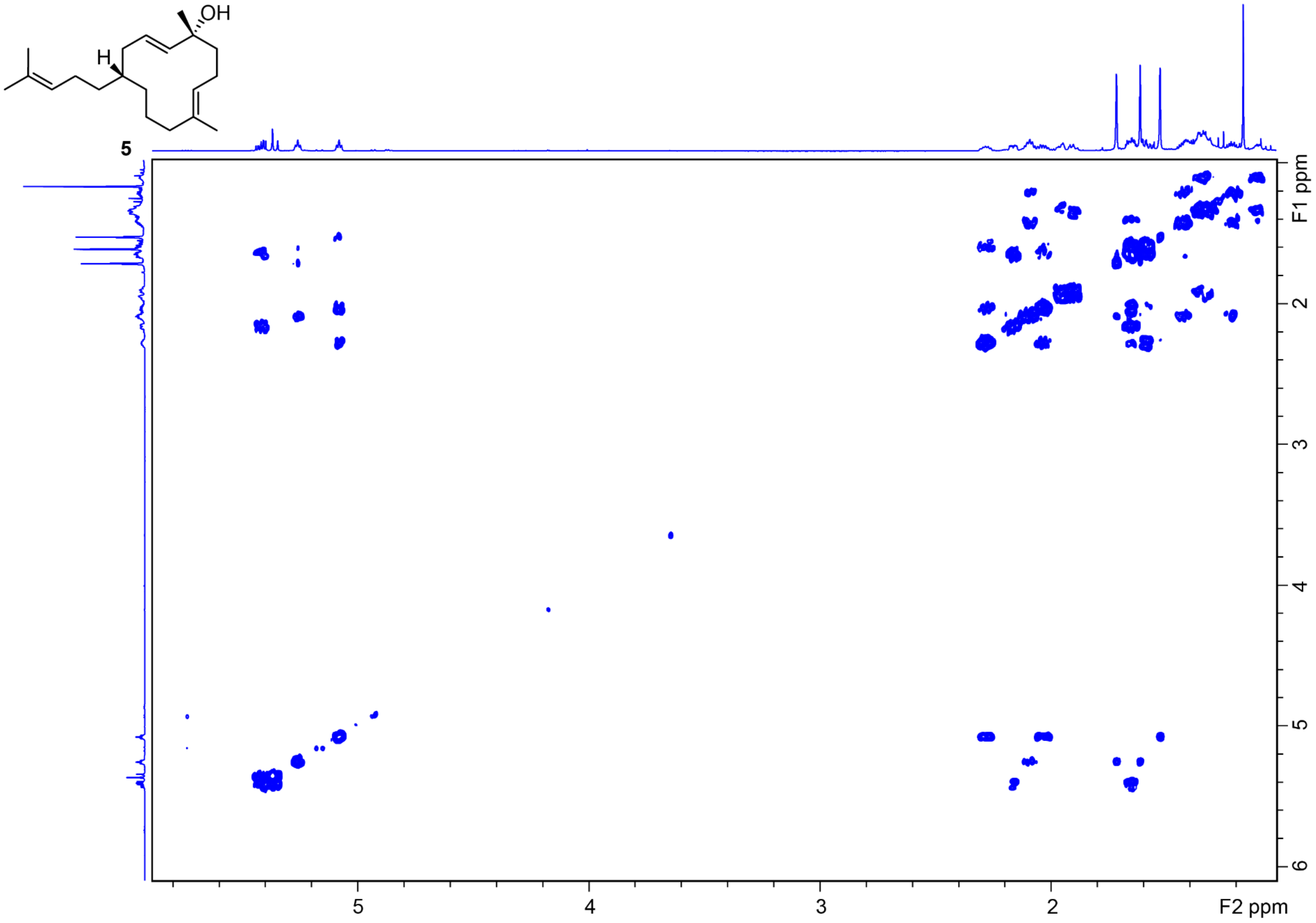

Figure S23. $^{1}H,^{1}H$-COSY spectrum (C_6D_6) of **5**.

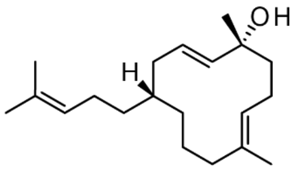

5

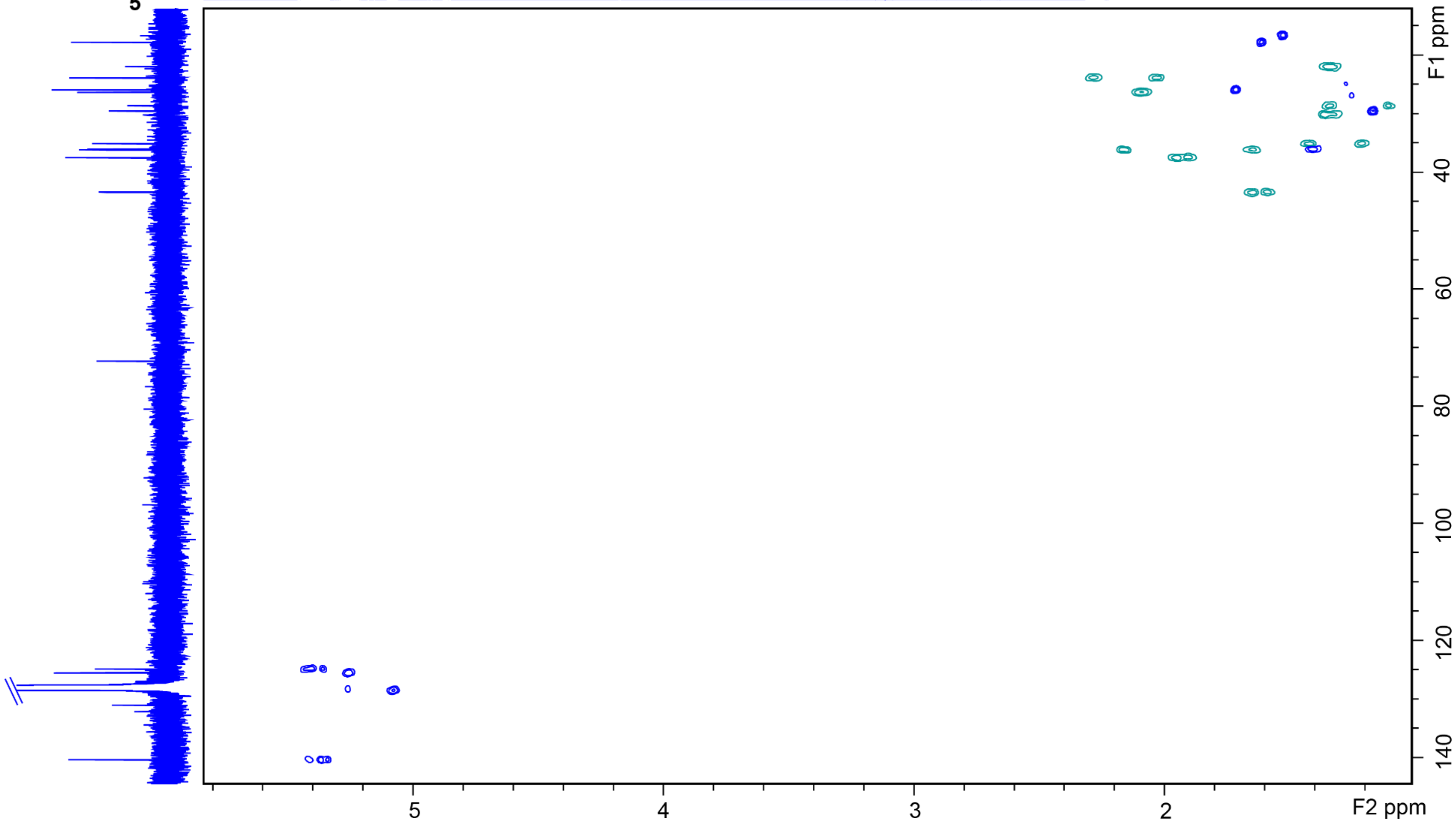

Figure S24. HSQC spectrum (C_6D_6) of **5**.

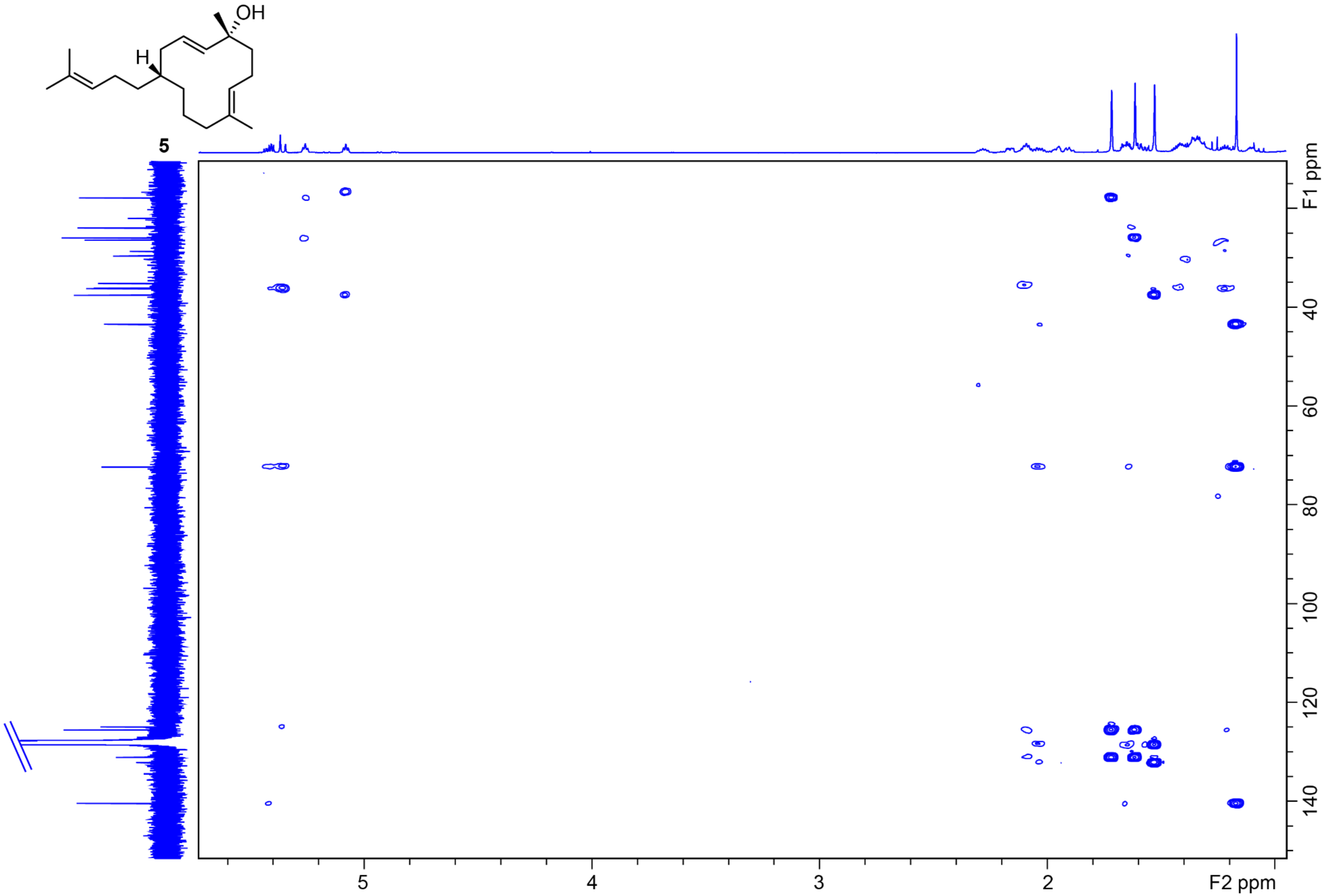

Figure S25. HMBC spectrum (C_6D_6) of **5**.

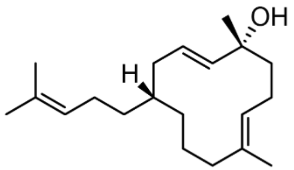

5

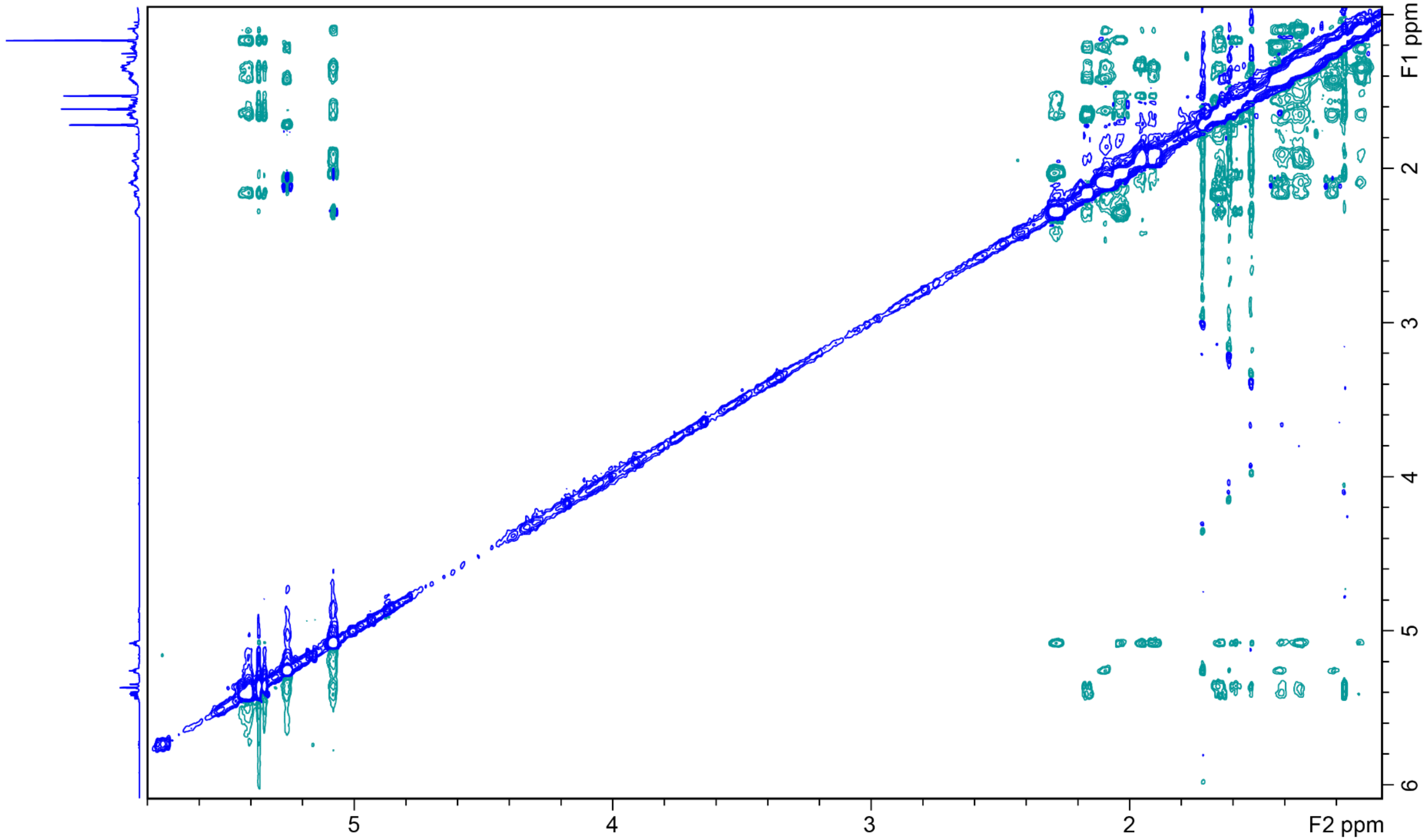

Figure S26. NOESY spectrum (C_6D_6) of **5**.

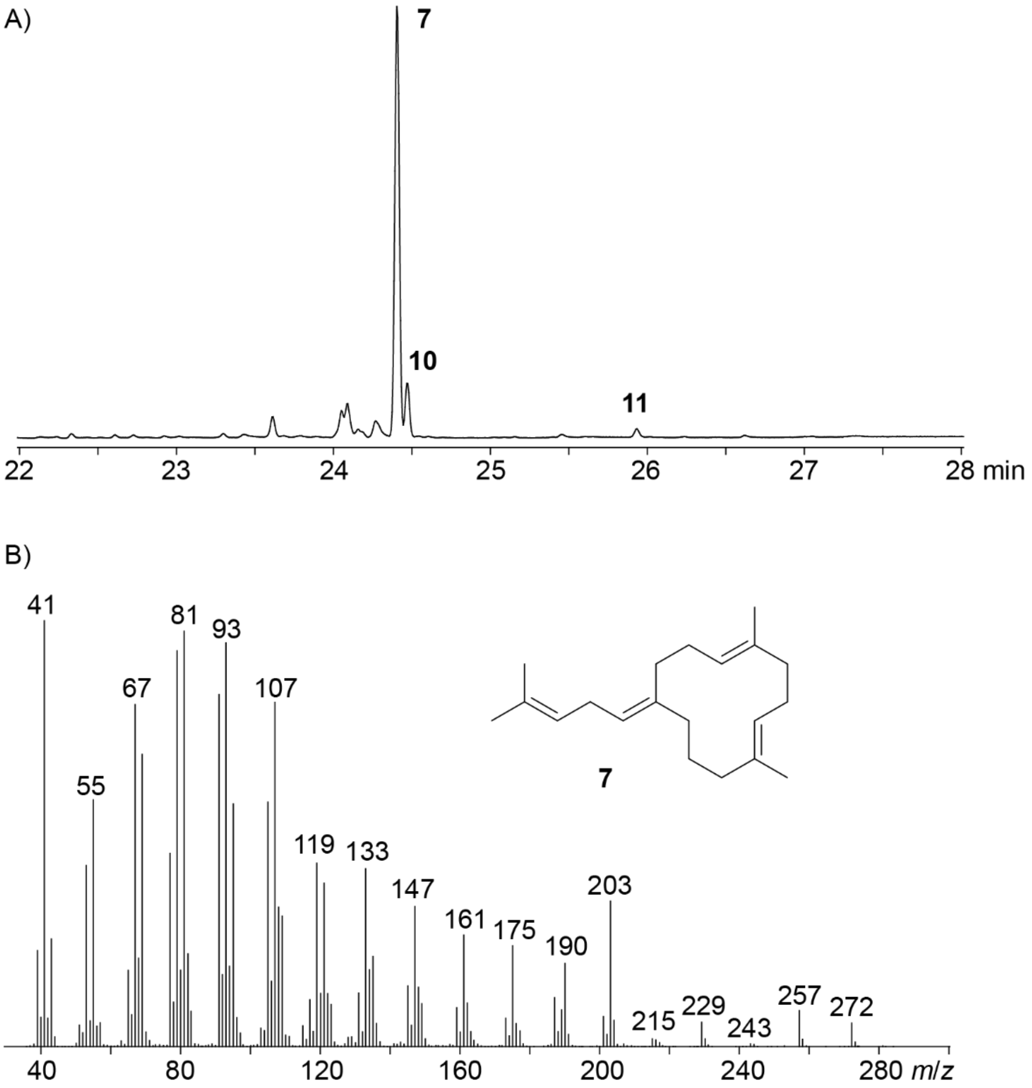

Figure S27. Product mixture formed from iso-FPP and IPP with GGPPS and SoS. A) Total ion chromatogram of the crude extract from the enzyme incubation, B) EI mass spectrum of **7**. The minor compounds **10** and **11** are explained below.

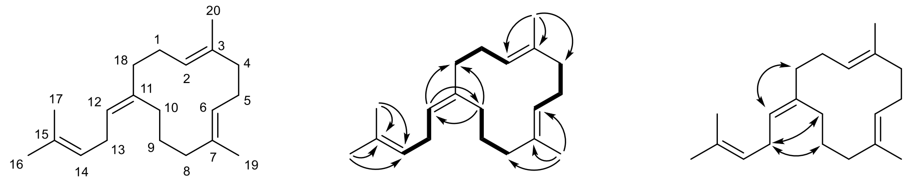

7

Figure S28. Structure elucidation of prenylpseudogermacrene A (**7**). Bold: ^{1}H,^{1}H-COSY, single-headed arrows: key HMBC, and double-headed arrows: key NOESY correlations.

Table S5. NMR data of prenylpseudogermacrene A (**7**) in C_6D_6 recorded at 298 K.

C[a]	type	^{13}C[b]	^{1}H[b]
1	CH_2	29.96	2.14 (m, 2H)
2	CH	128.88	5.29 (m)
3	C_q	133.16	–
4	CH_2	39.81	2.02 (m, 2H)
5	CH_2	26.11	2.15 (m, 2H)
6	CH	126.57	4.93 (br t, ^3J = 8.0)
7	C_q	134.18	–
8	CH_2	39.51	1.93 (m, 2H)
9	CH_2	25.96	1.37 (m, 2H)
10	CH_2	27.60	2.27 (m, 2H)
11	C_q	142.36	–
12	CH	124.73	5.30 (m)
13	CH_2	27.30	2.86 (dd, ^3J = 7.3, 7.3)
14	CH	124.54	5.29 (m)
15	C_q	130.92	–
16	CH_3	25.88	1.68 (d, ^4J = 1.2)
17	CH_3	17.78	1.61 (br s)
18	CH_2	37.11	2.08 (m, 2H)
19	CH_3	17.11	1.52 (m)
20	CH_3	15.02	1.45 (m)

[a] Carbon numbering as shown in Figure S28. [b] Chemical shifts δ in ppm, multiplicity: s = singlet, d = doublet, t = triplet, m = multiplet, br = broad, coupling constants J are given in Hertz.

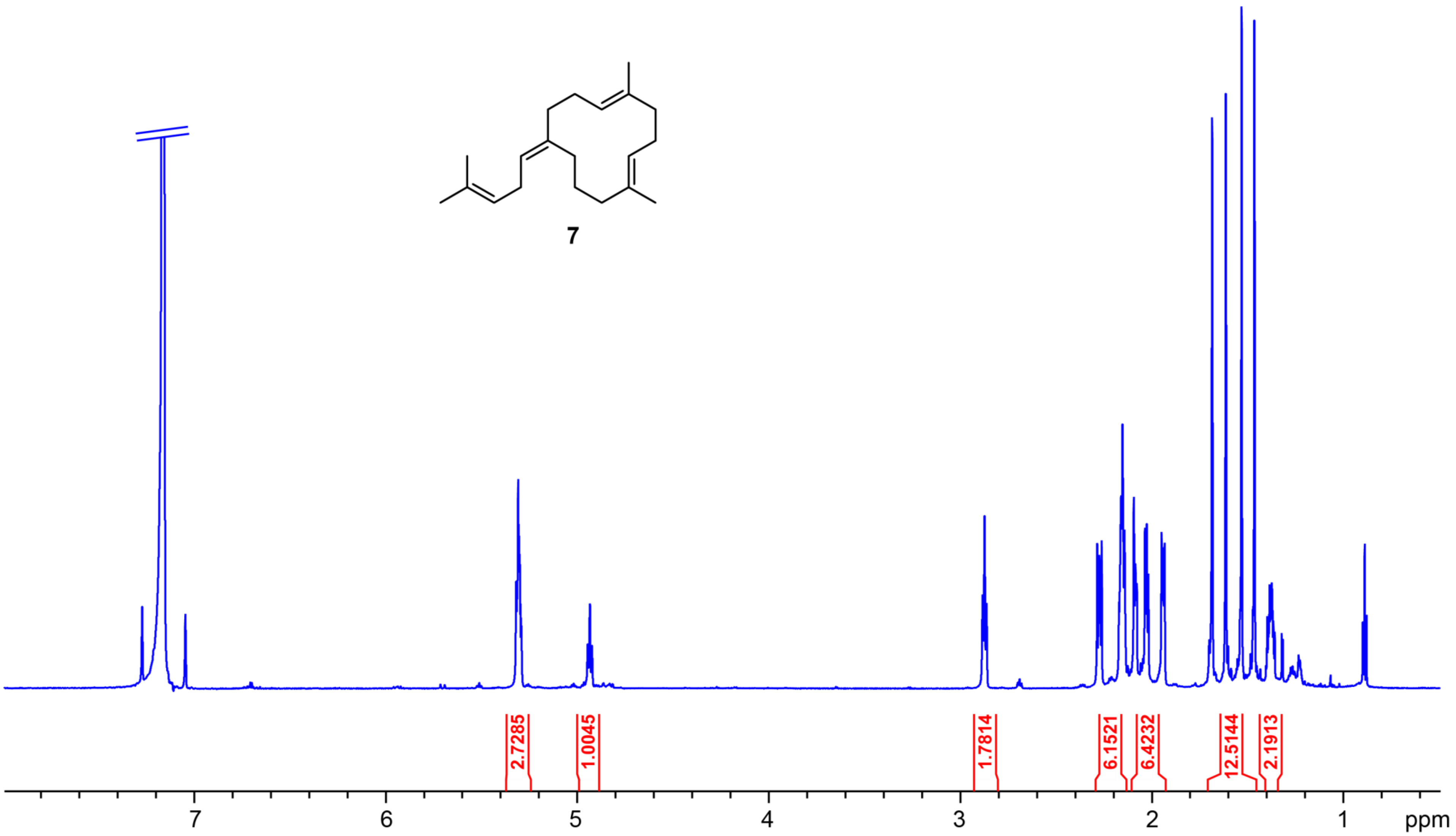

Figure S29. ^{1}H-NMR spectrum (700 MHz, C_6D_6) of **7**.

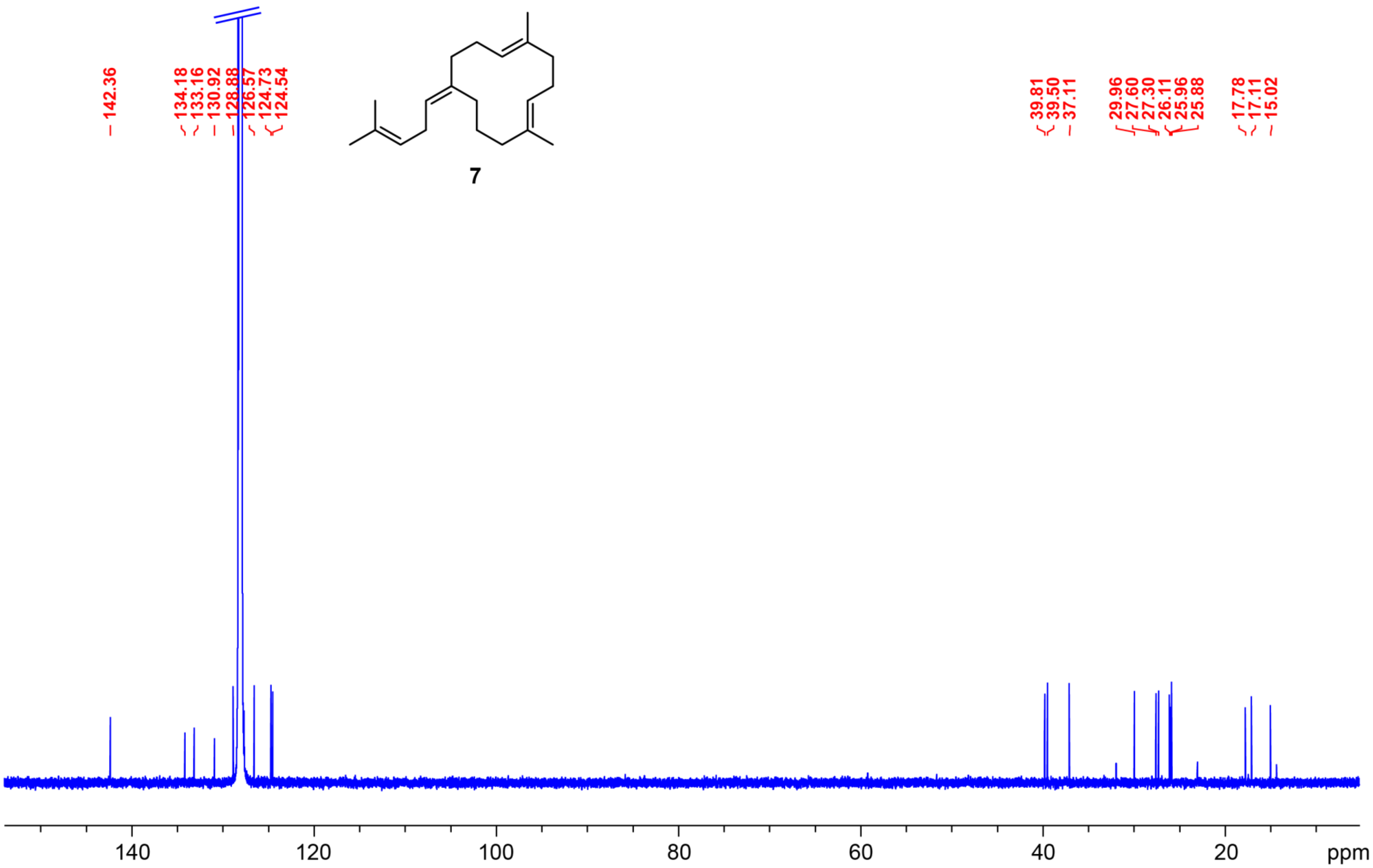

Figure S30. ^{13}C-NMR spectrum (176 MHz, C_6D_6) of **7**.

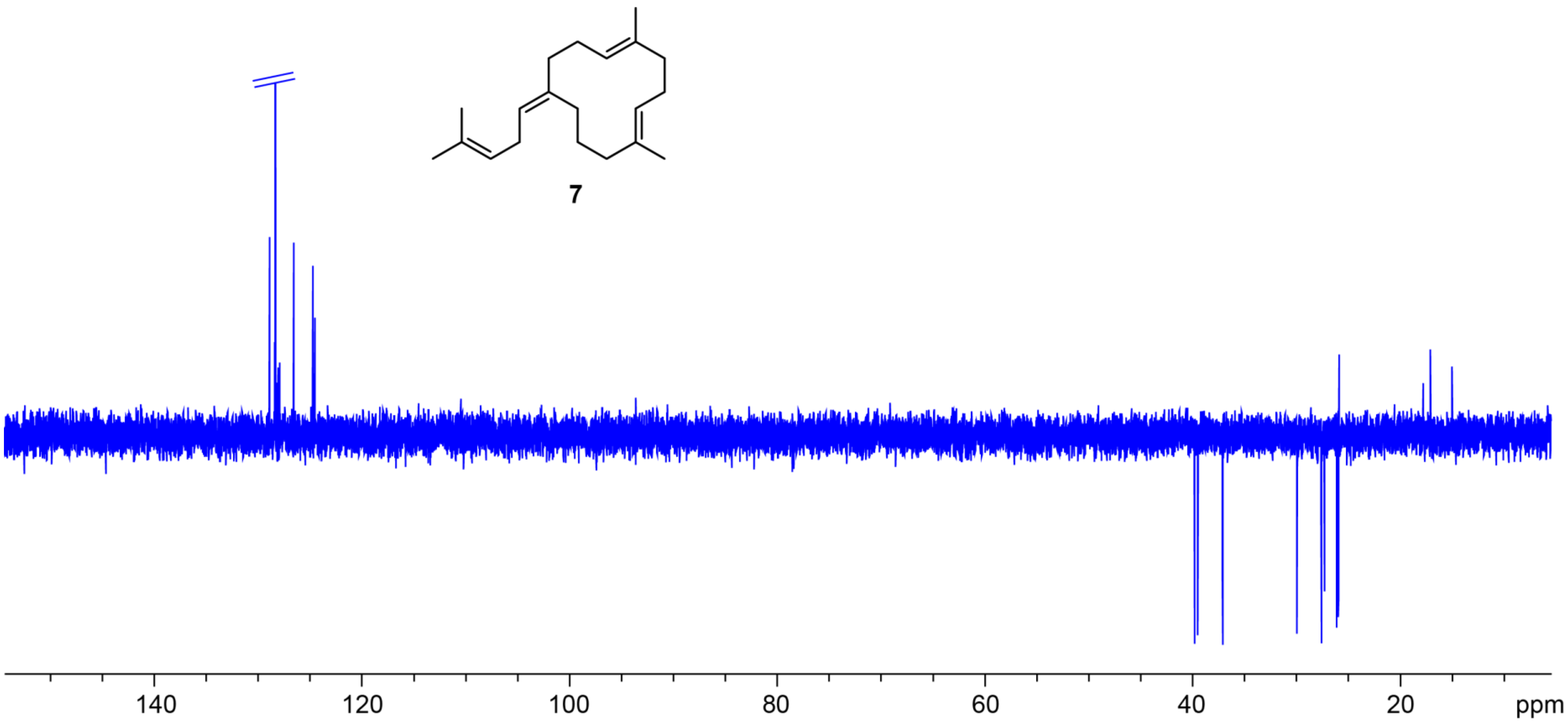

Figure S31. ^{13}C-DEPT135 spectrum (176 MHz, C_6D_6) of **7**.

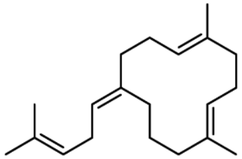

7

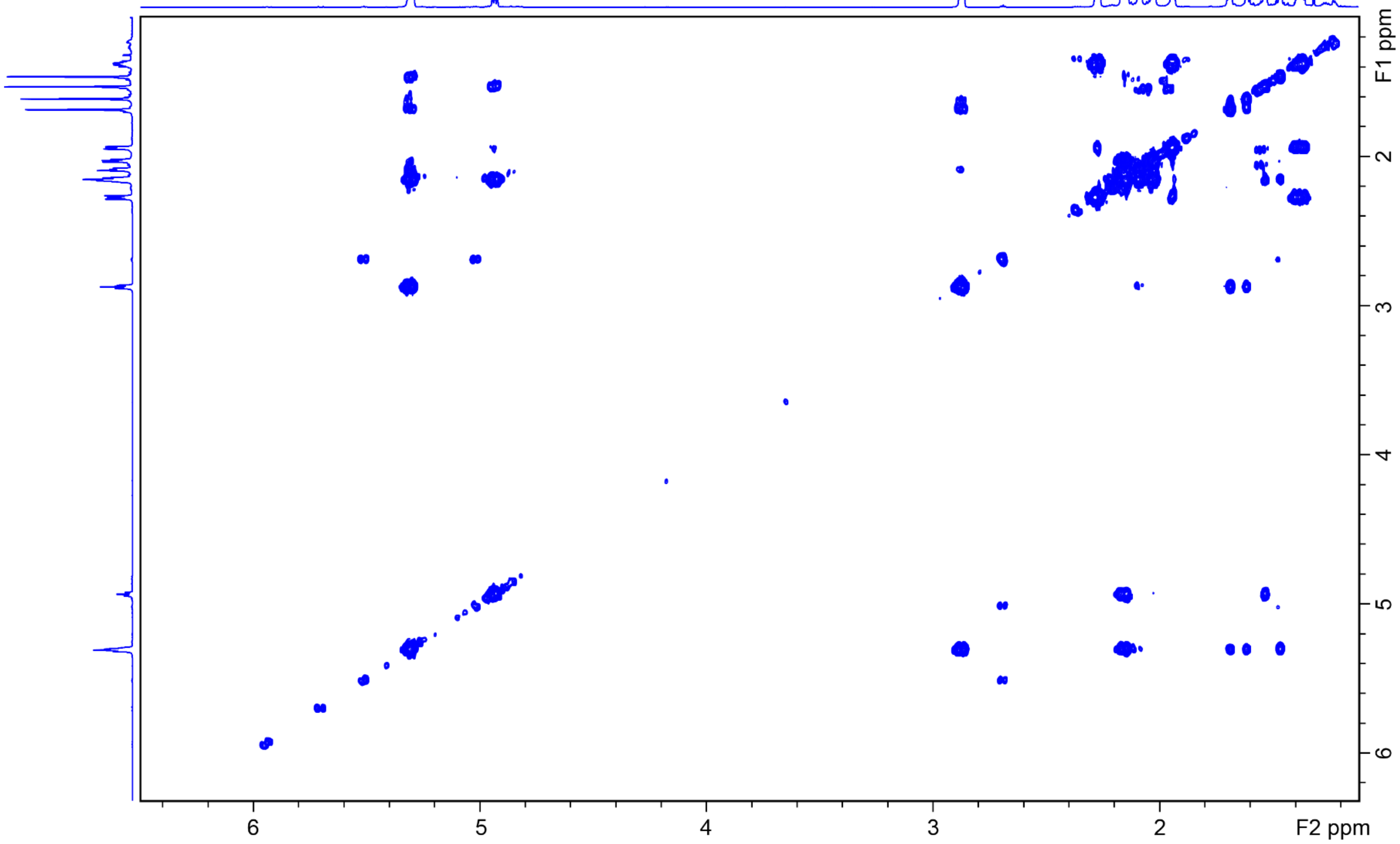

Figure S32. $^{1}H,^{1}H$-COSY spectrum (C_6D_6) of **7**.

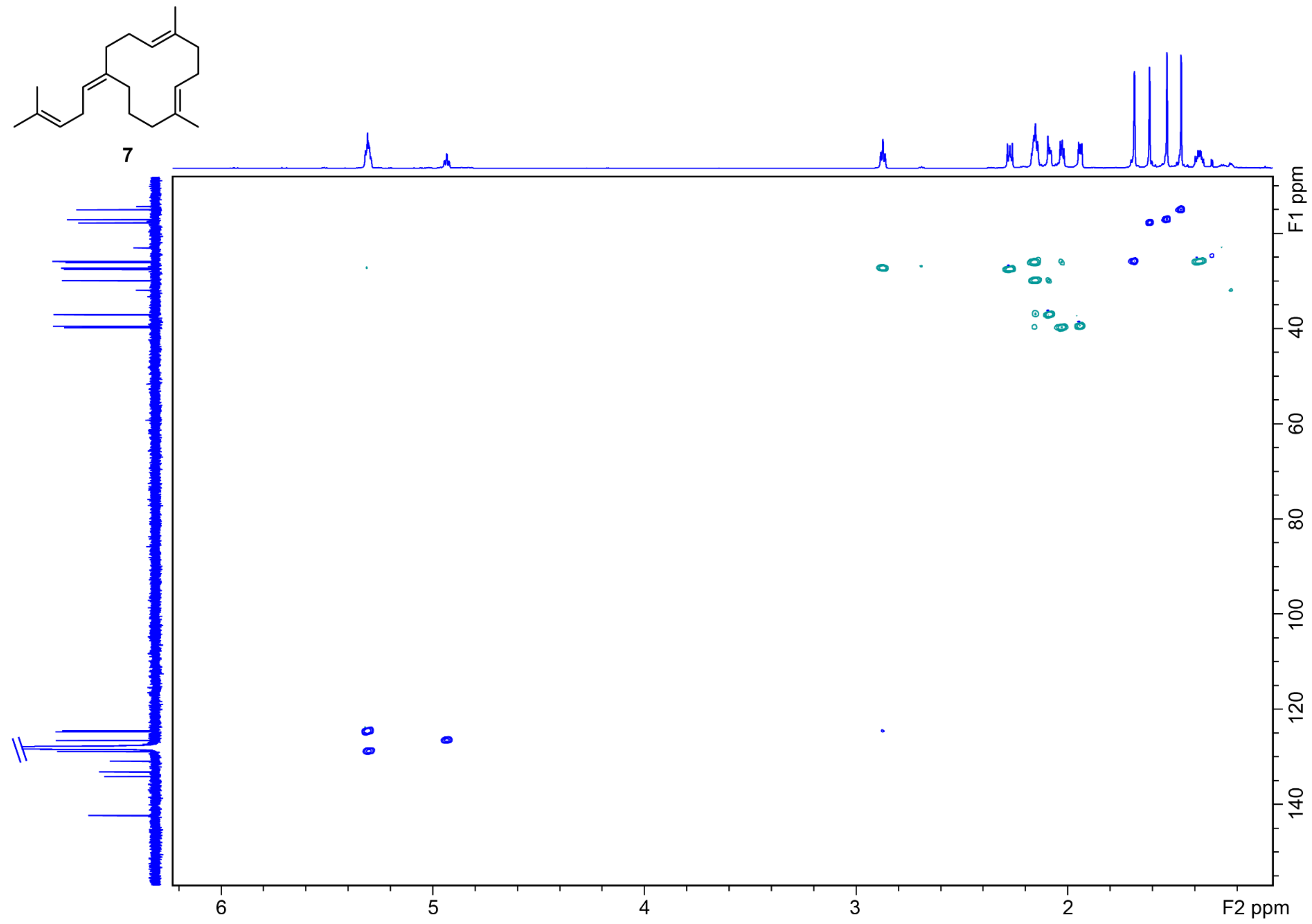

Figure S33. HSQC spectrum (C_6D_6) of **7**.

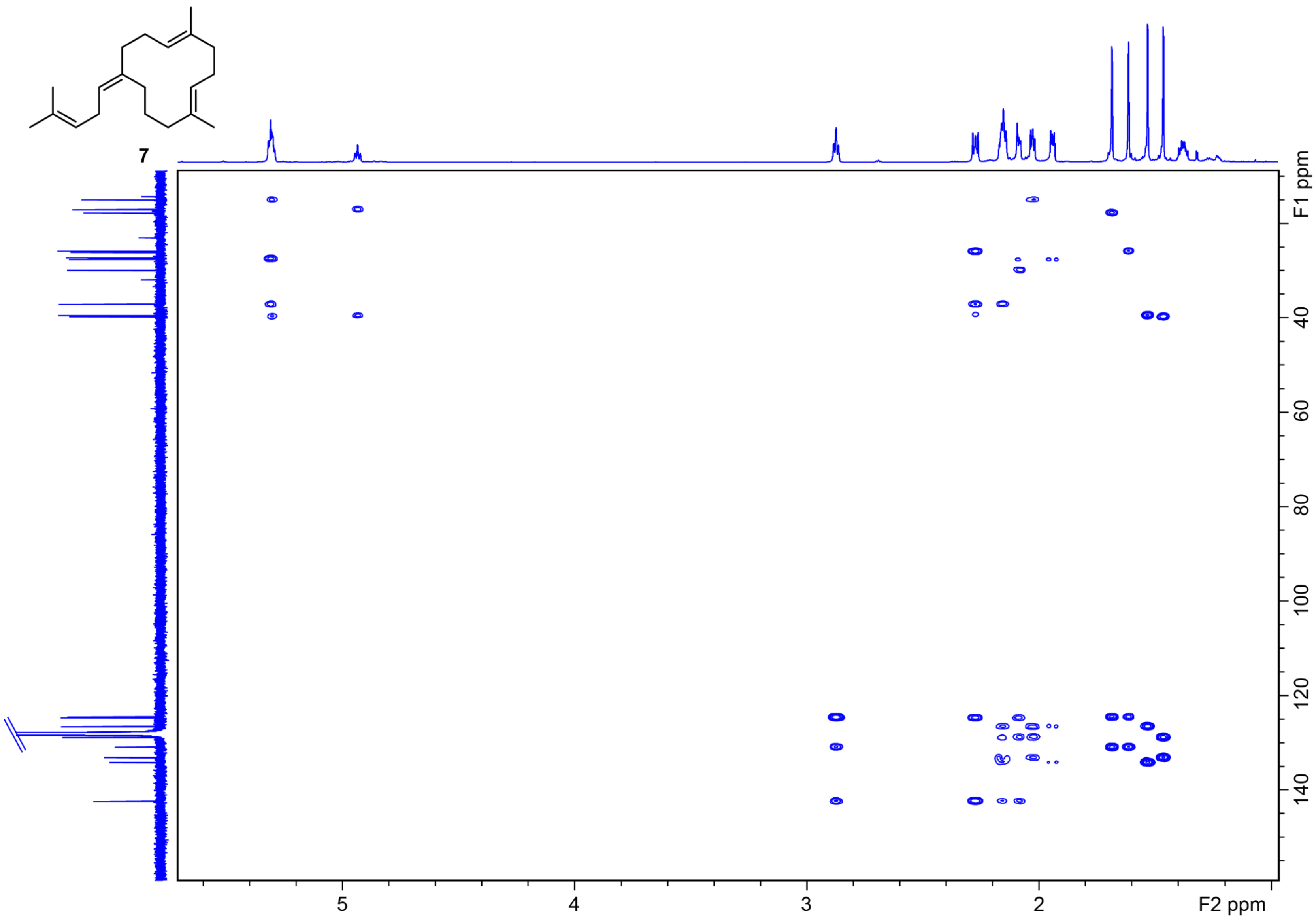

Figure S34. HMBC spectrum (C_6D_6) of **7**.

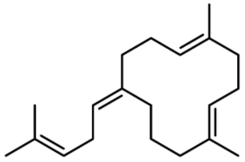

7

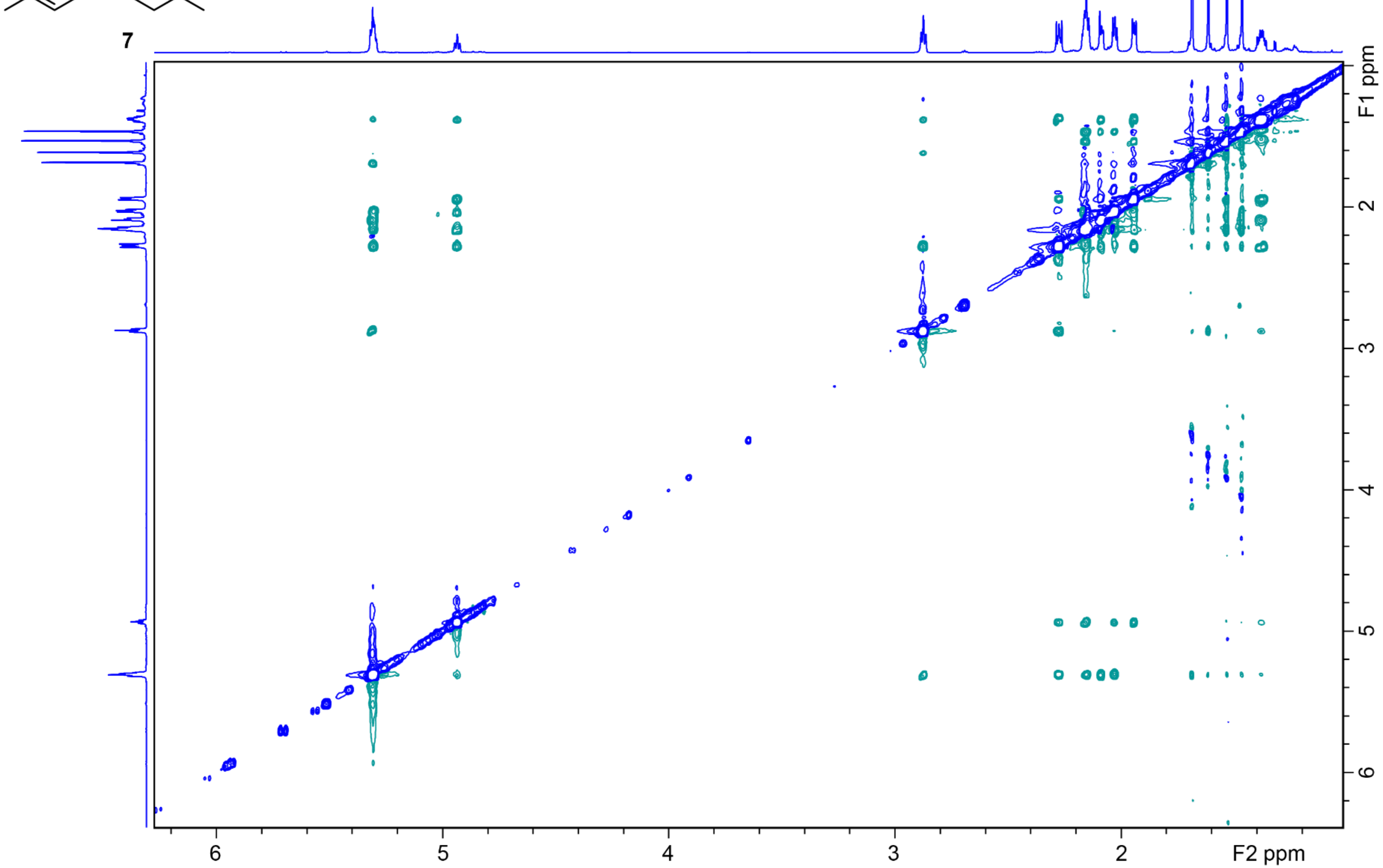

Figure S35. NOESY spectrum (C_6D_6) of **7**.

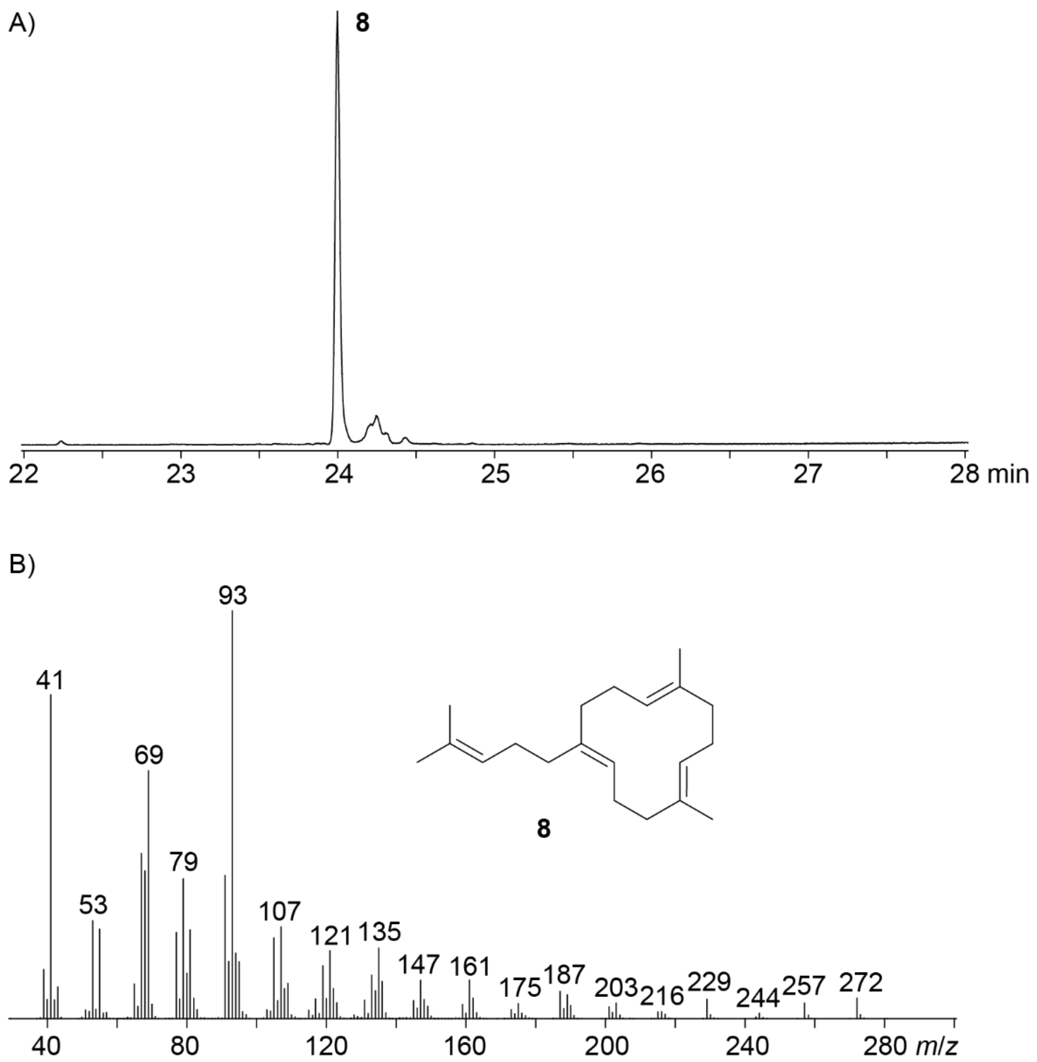

Figure S36. Product mixture formed from iso-FPP and IPP with GGPPS and SaS. A) Total ion chromatogram of the crude extract from the enzyme incubation, B) EI mass spectrum of **8**.

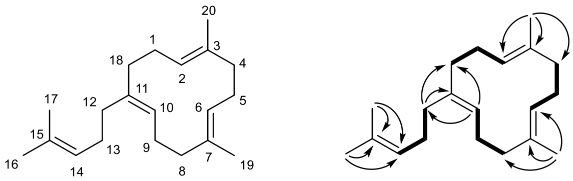

8

Figure S37. Structure elucidation of prenylpseudogermacrene B (**8**). Bold: ^{1}H,^{1}H-COSY, single-headed arrows: key HMBC, and double-headed arrows: key NOESY correlations.

Table S6. NMR data of prenylpseudogermacrene B (**8**) in C_6D_6 recorded at 298 K.

C[a]	type	^{13}C[b]	^{1}H[b]
1	CH_2	26.06	2.10 (m, 2H)
2	CH	127.09	4.84 (m)
3	C_q	133.34	–
4	CH_2	40.21	2.05 (m, 2H)
5	CH_2	25.53	2.09 (m, 2H)
6	CH	126.89	4.83 (m)
7	C_q	133.14	–
8	CH_2	40.33	2.05 (m, 2H)
9	CH_2	25.27	2.13 (m, 2H)
10	CH	127.85	4.82 (m)
11	C_q	137.15	–
12	CH_2	29.42	2.00 (m, 2H)
13	CH_2	27.96	2.13 (m, 2H)
14	CH	125.18	5.27 (thept, ^{3}J = 7.1, ^{4}J = 1.4)
15	C_q	131.26	–
16	CH_3	25.91	1.68 (br s)
17	CH_3	17.75	1.59 (br s)
18	CH_2	37.20	2.09 (m, 2H)
19	CH_3	15.28	1.43 (br s)
20	CH_3	15.21	1.42 (br s)

[a] Carbon numbering as shown in Figure S37. [b] Chemical shifts δ in ppm, multiplicity: s = singlet, t = triplet, hept = heptet, m = multiplet, br = broad, coupling constants *J* are given in Hertz.

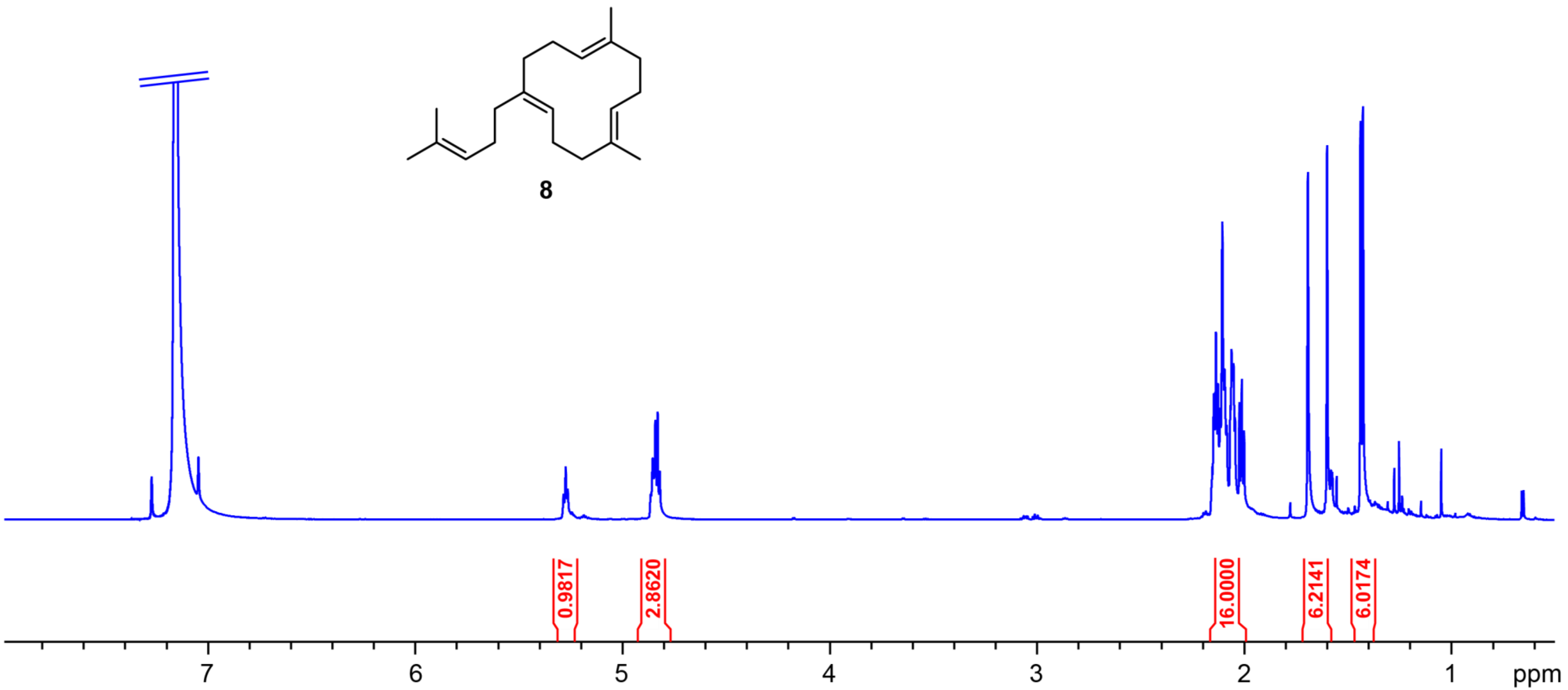

Figure S38. ^{1}H-NMR spectrum (700 MHz, C_6D_6) of **8**.

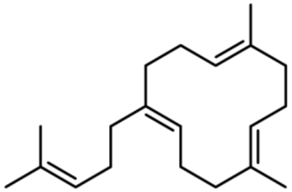

8

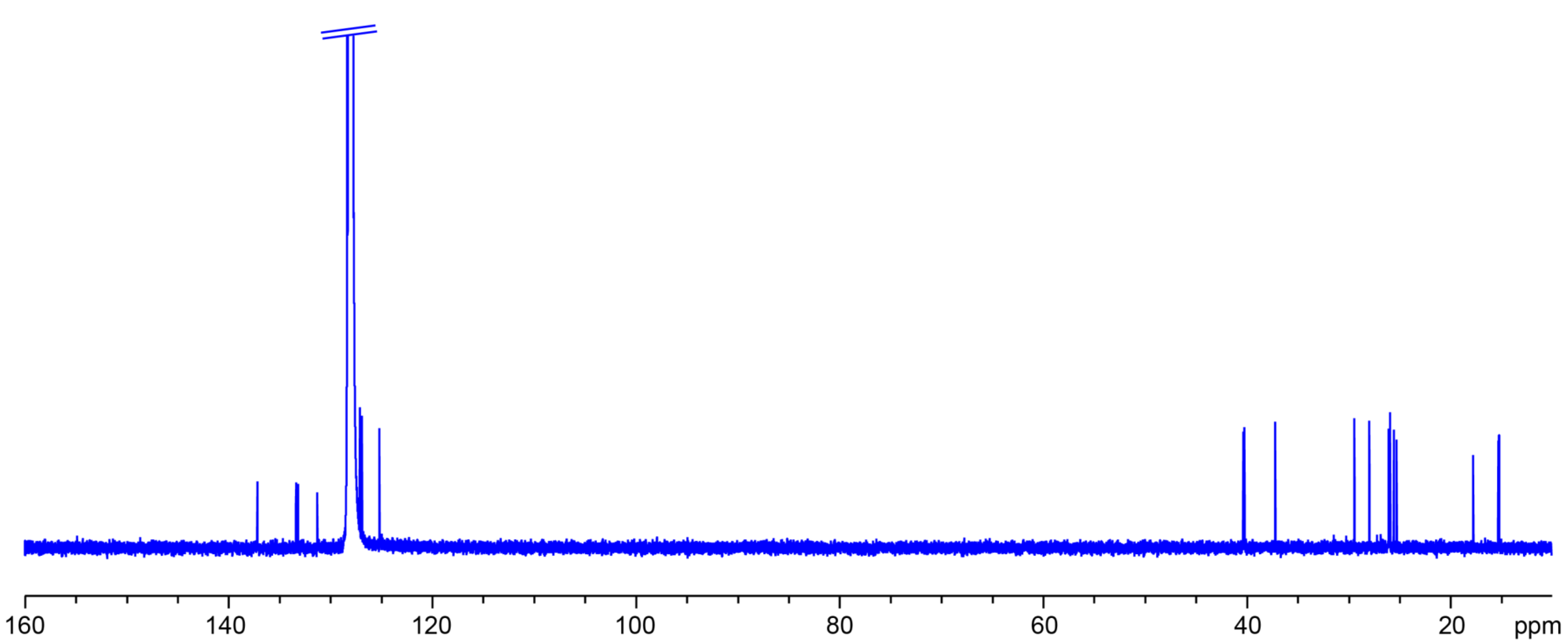

Figure S39. ^{13}C-NMR spectrum (176 MHz, C_6D_6) of **8**.

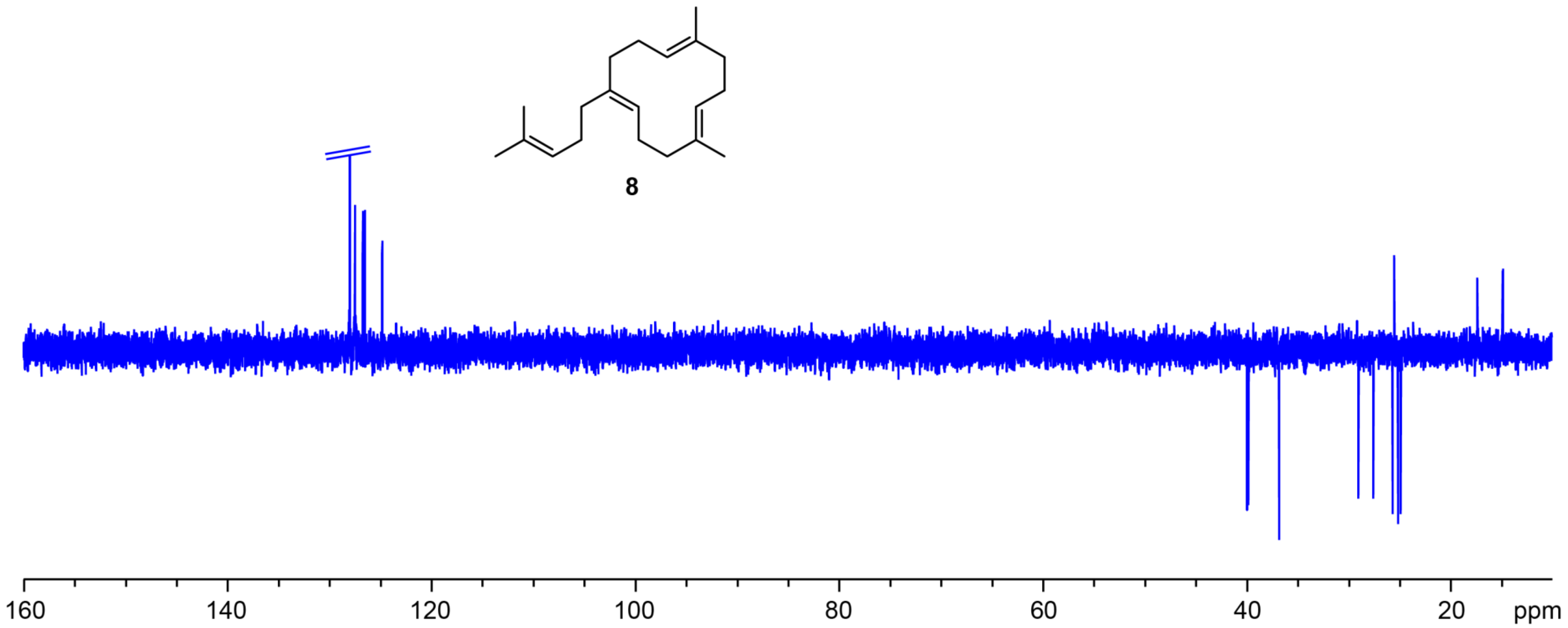

Figure S40. ^{13}C-DEPT135 spectrum (176 MHz, C_6D_6) of **8**.

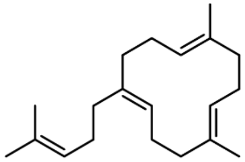

8

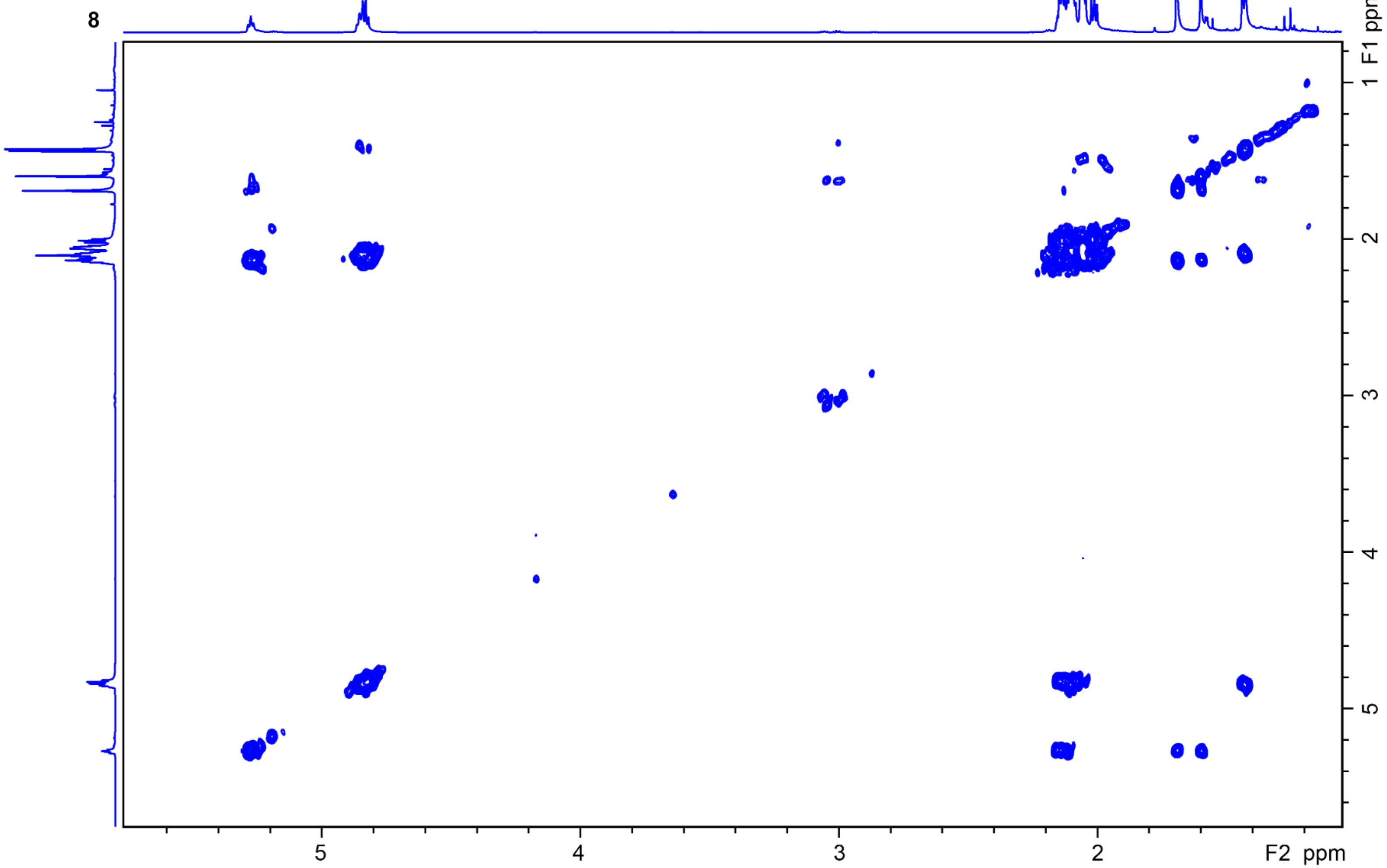

Figure S41. $^{1}H,^{1}H$-COSY spectrum (C_6D_6) of **8**.

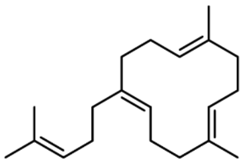

8

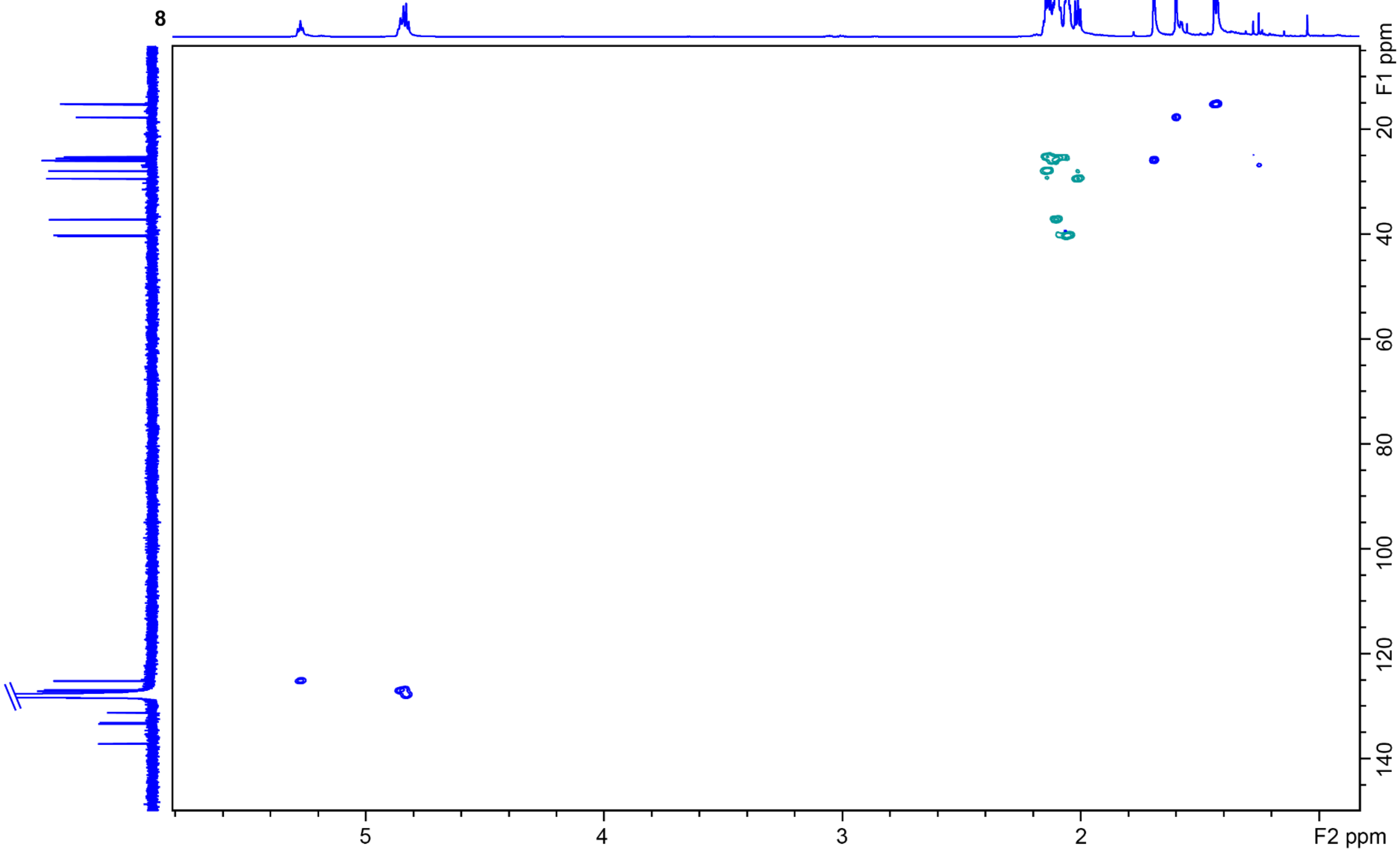

Figure S42. HSQC spectrum (C_6D_6) of **8**.

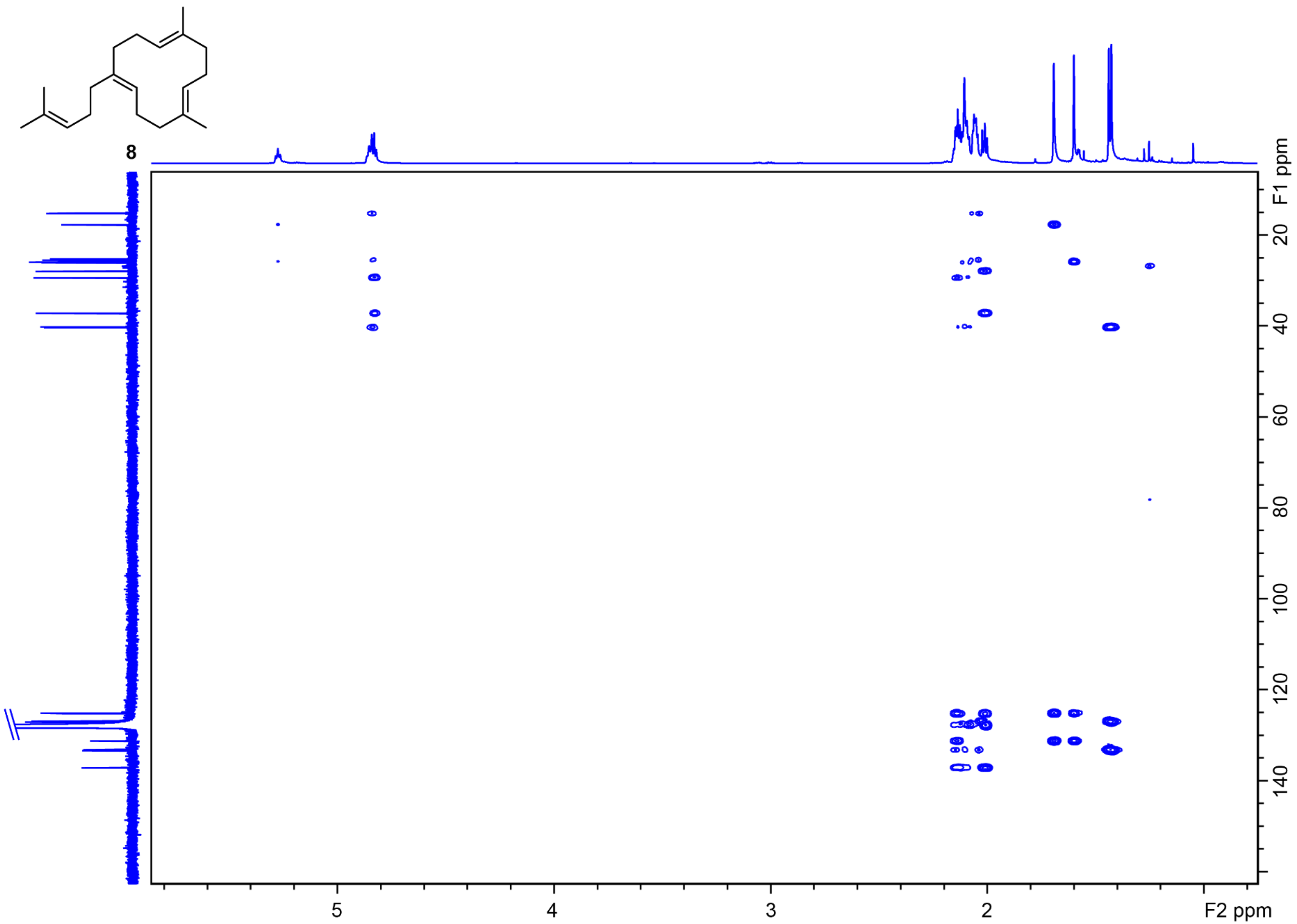

Figure S43. HMBC spectrum (C_6D_6) of **8**.

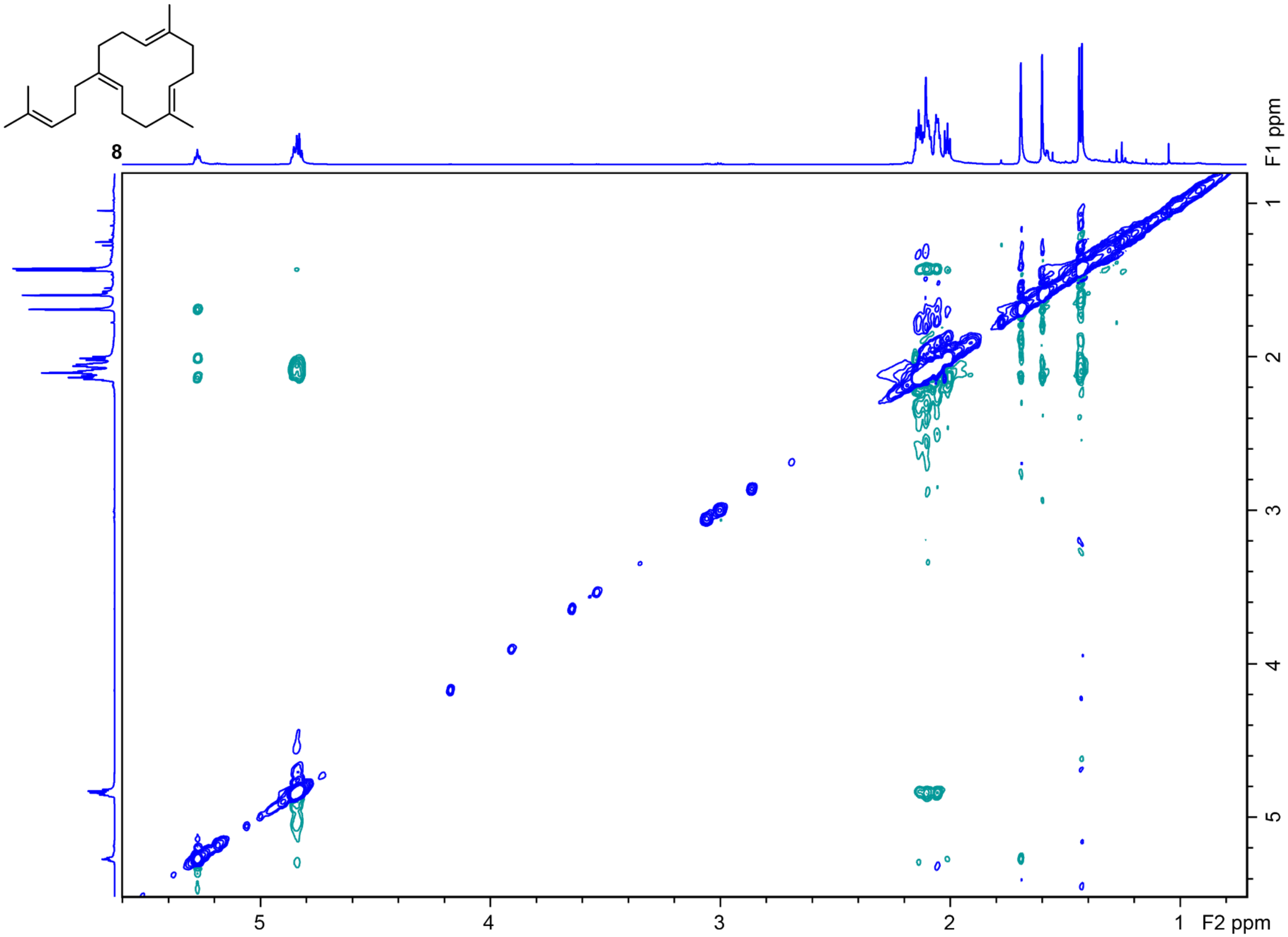

Figure S44. NOESY spectrum (C_6D_6) of **8**.

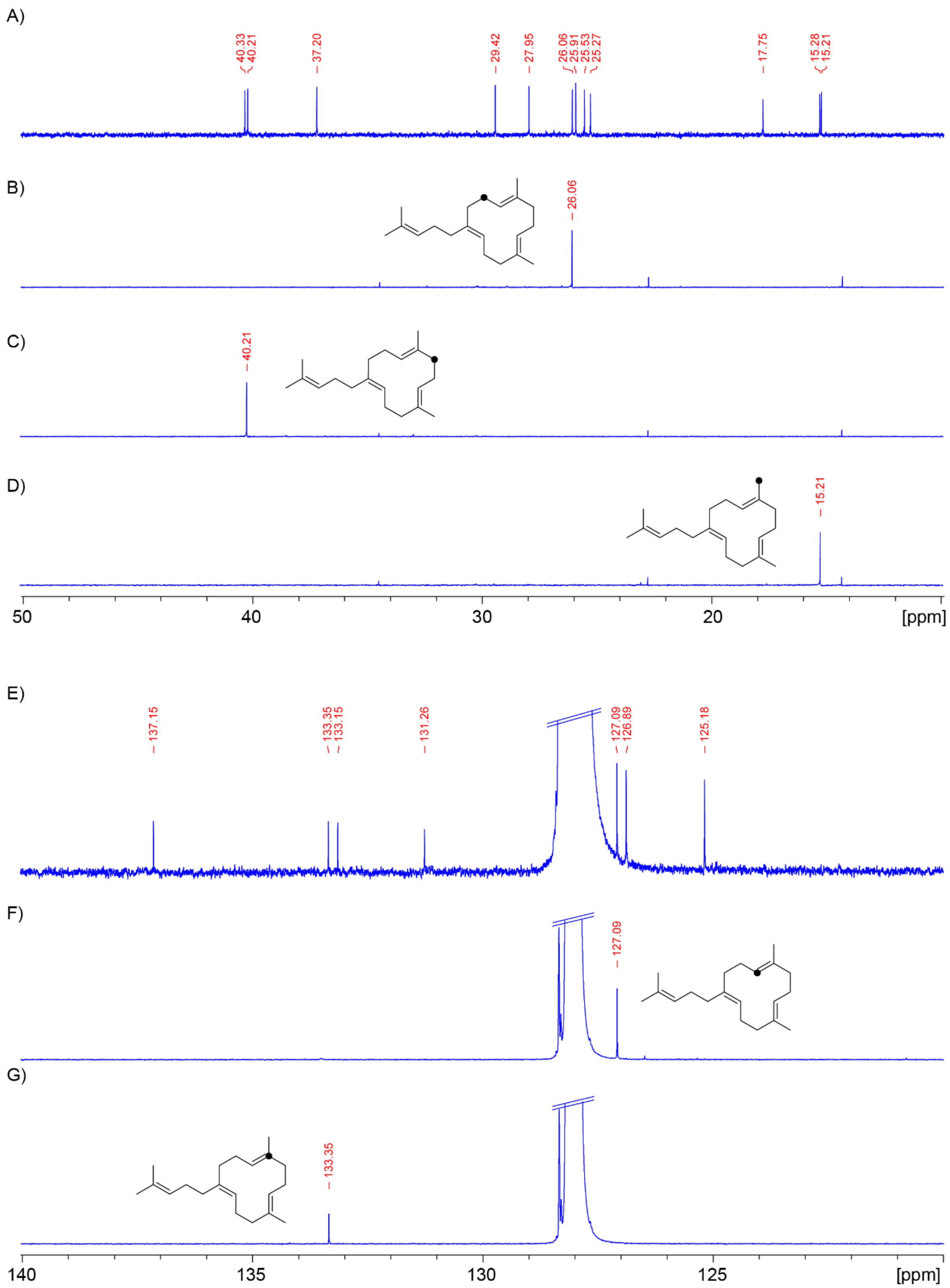

Figure S45. ^{13}C-NMR spectra of A) unlabelled **8** (aliphatic region), ^{13}C-labelled **8** obtained with GGPPS and SaS from iso-FPP and B) (1-^{13}C)IPP, C) (4-^{13}C)IPP and D) (5-^{13}C)IPP, E) unlabelled **8** (olefinic region), ^{13}C-labelled **8** obtained with GGPPS and SaS from iso-FPP and F) (2-^{13}C)IPP and G) (3-^{13}C)IPP. The labelling experiments allowed for an unambiguous assignment of the ^{13}C-NMR shifts of carbons in corresponding positions of the macrocycle in pseudo-C_3 symmetric **8**.

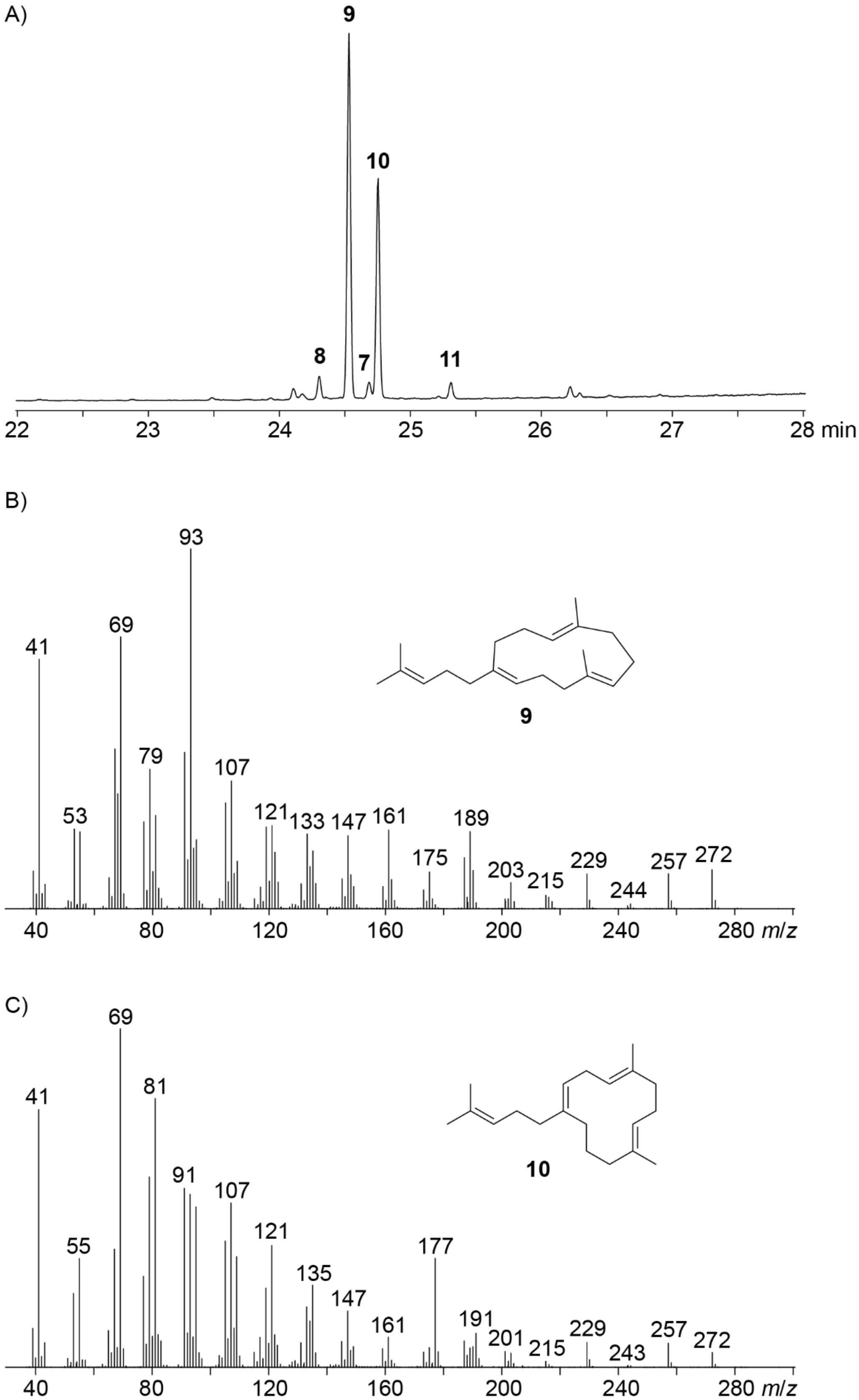

Figure S46. Product mixture formed from iso-FPP and IPP with AbVS. A) Total ion chromatogram of the crude extract from the enzyme incubation, B) EI mass spectrum of **9**, C) EI mass spectrum of **10**. The minor compound **11** is explained below.

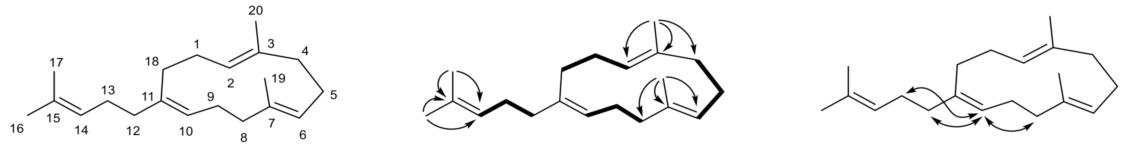

9

Figure S47. Structure elucidation of prenylpseudogermacrene C (**9**). Bold: ^{1}H,^{1}H-COSY, single-headed arrows: key HMBC, and double-headed arrows: key NOESY correlations.

Table S7. NMR data of prenylpseudogermacrene C (**9**) in C_6D_6 recorded at 298 K.

C[a]	type	^{13}C[b]	^{1}H[b]
1	CH_2	25.04	2.15 (m, 2H)
2	CH	126.64	4.98 (t, ^{3}J = 7.3)
3	C_q	132.02	–
4	CH_2	39.10	1.99 (m, 2H)
5	CH_2	25.36	2.08 (m, 2H)
6	CH	125.39	4.93 (t, ^{3}J = 7.6)
7	C_q	135.45	–
8	CH_2	39.47	2.04 (m, 2H)
9	CH_2	28.40	2.12 (m, 2H)
10	CH	127.08	5.20 (t, ^{3}J = 7.5)
11	C_q	137.57	–
12	CH_2	36.32	2.13 (m, 2H)
13	CH_2	27.34	2.20 (m, 2H)
14	CH	125.21	5.27 (thept, ^{3}J = 6.8, ^{2}J = 1.4)
15	C_q	131.12	–
16	CH_3	17.78	1.58 (br s)
17	CH_3	25.89	1.68 (br s)
18	CH_2	29.28	2.00 (m, 2H)
19	CH_3	17.99	1.53 (br s)
20	CH_3	15.58	1.49 (br s)

[a] Carbon numbering as shown in Figure S47. [b] Chemical shifts δ in ppm, multiplicity: s = singlet, t = triplet, m = multiplet, hept = heptet, br = broad, coupling constants J are given in Hertz.

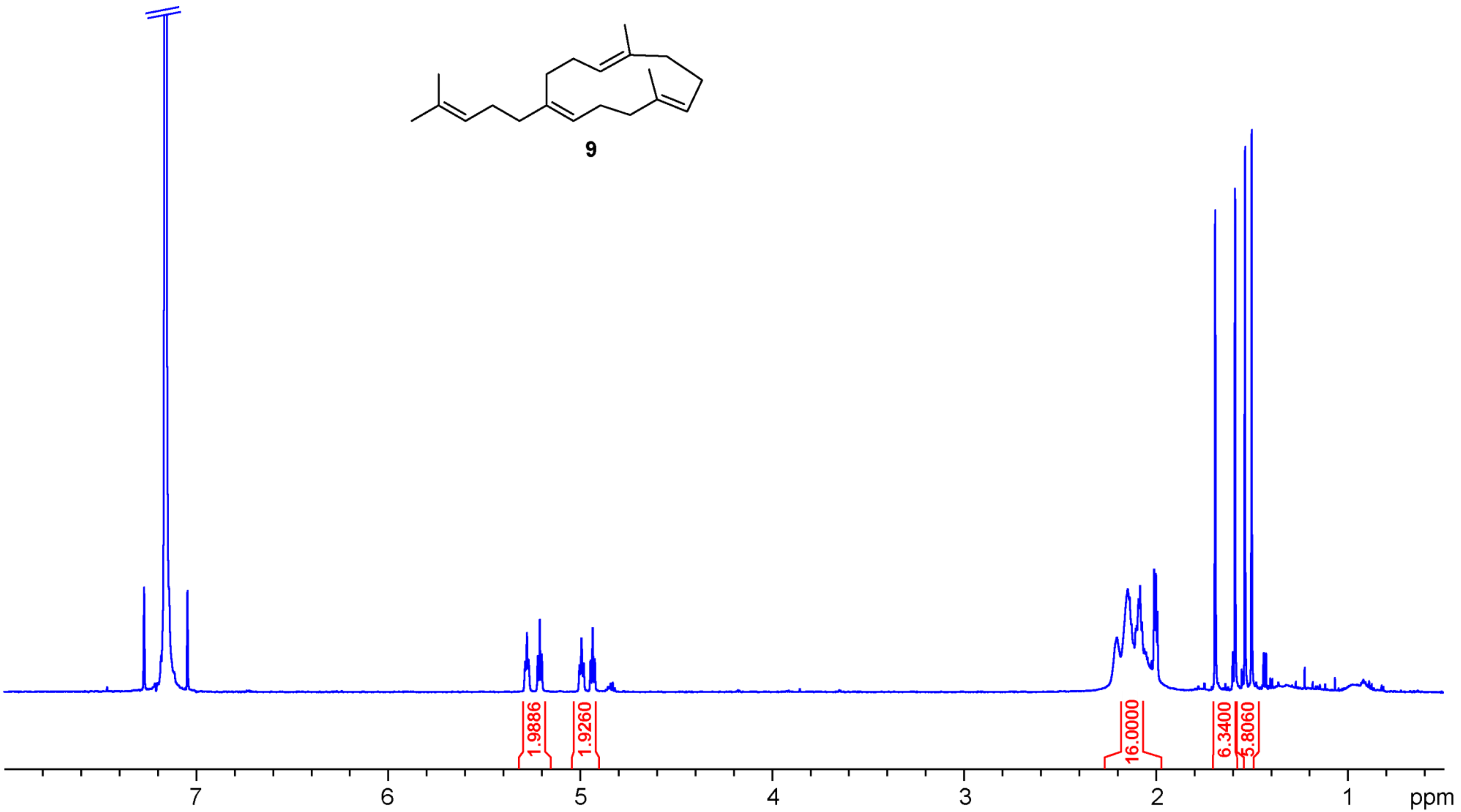

Figure S48. ^{1}H-NMR spectrum (700 MHz, C_6D_6) of **9**.

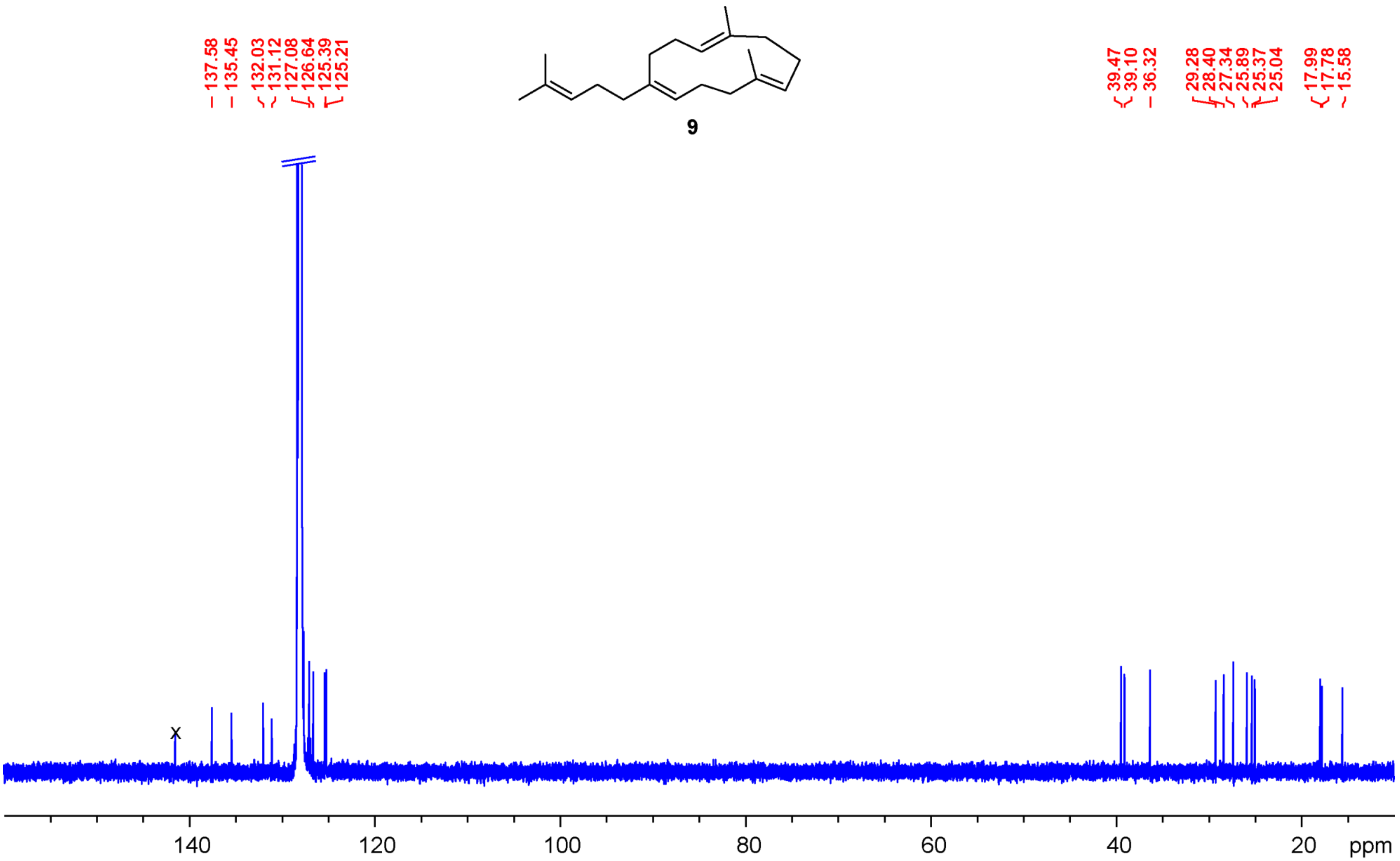

Figure S49. ^{13}C-NMR spectrum (176 MHz, C_6D_6) of **9**.

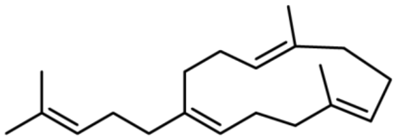

9

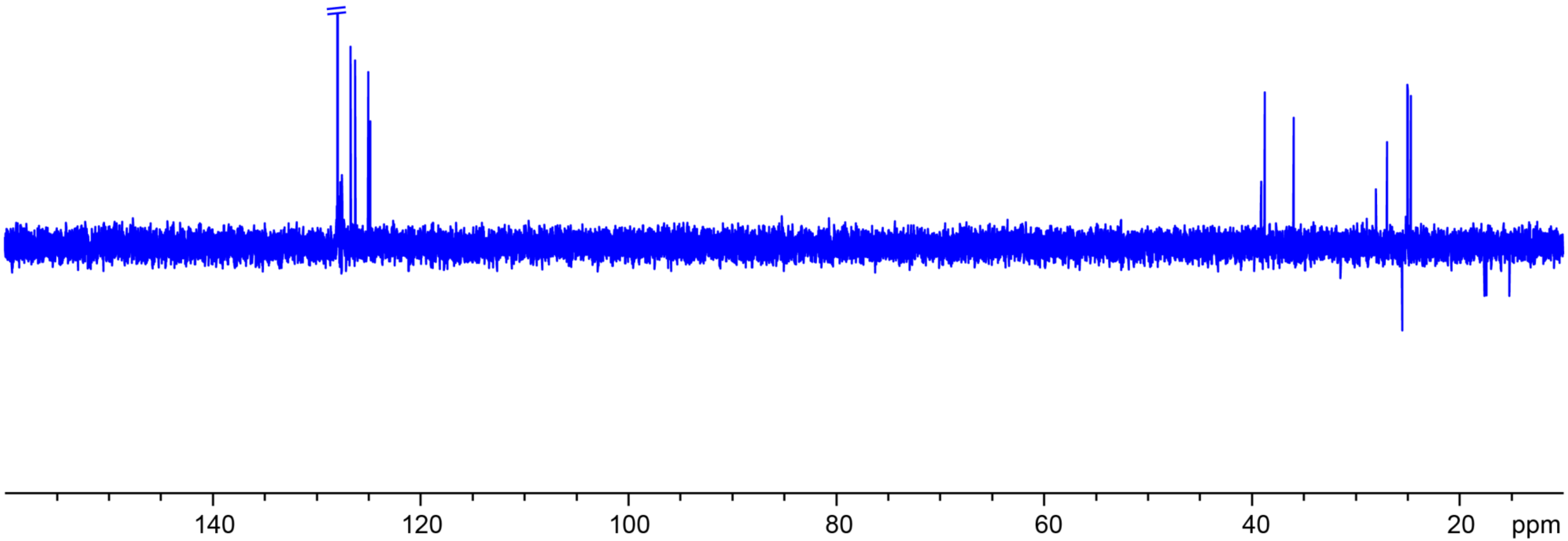

Figure S50. ^{13}C-DEPT135 spectrum (176 MHz, C_6D_6) of **9**.

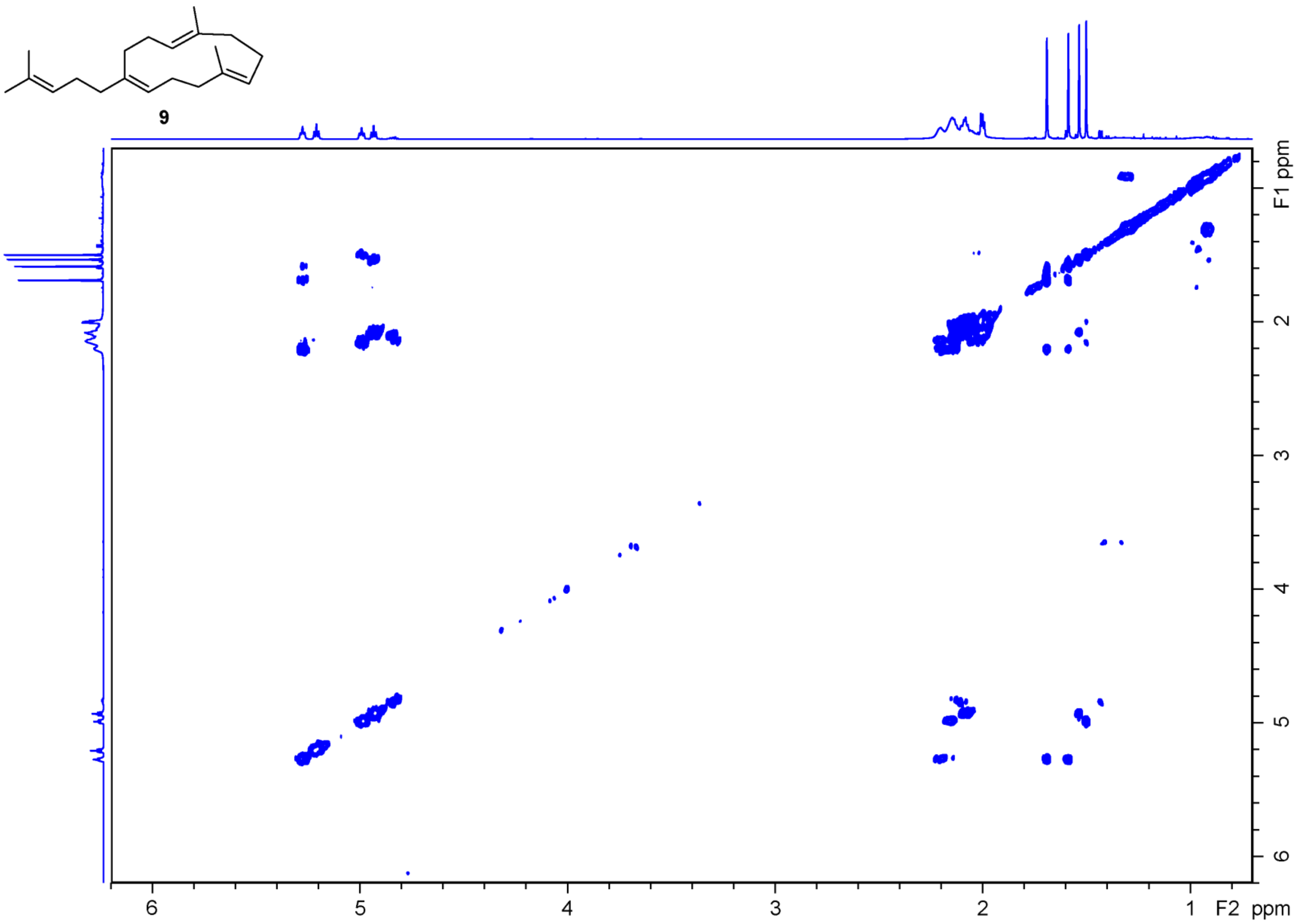

Figure S51. ^{1}H,^{1}H-COSY spectrum (C_6D_6) of **9**.

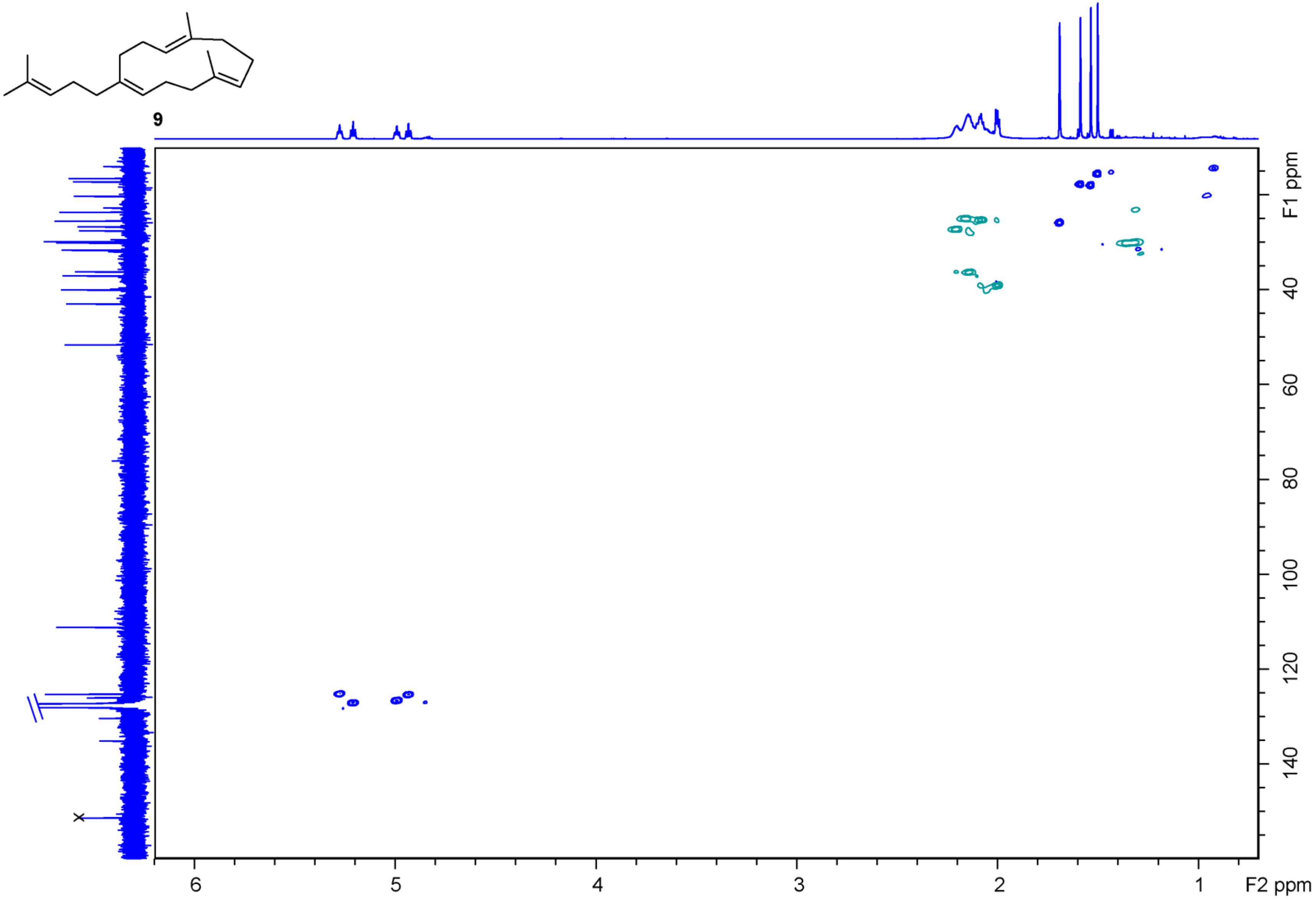

Figure S52. HSQC spectrum (C_6D_6) of **9**.

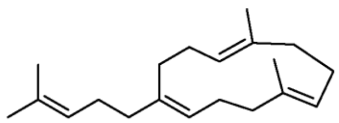

9

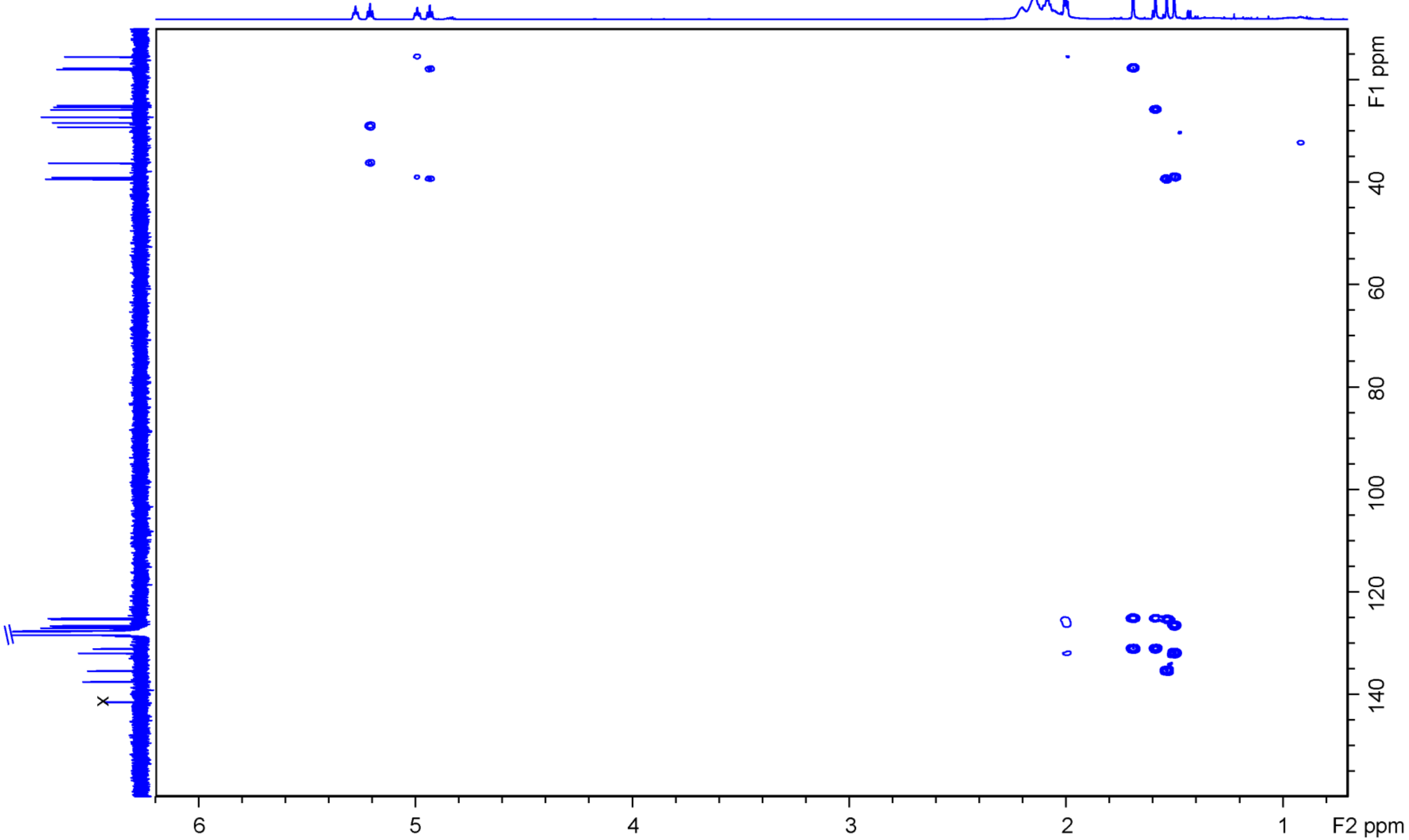

Figure S53. HMBC spectrum (C_6D_6) of **9**.

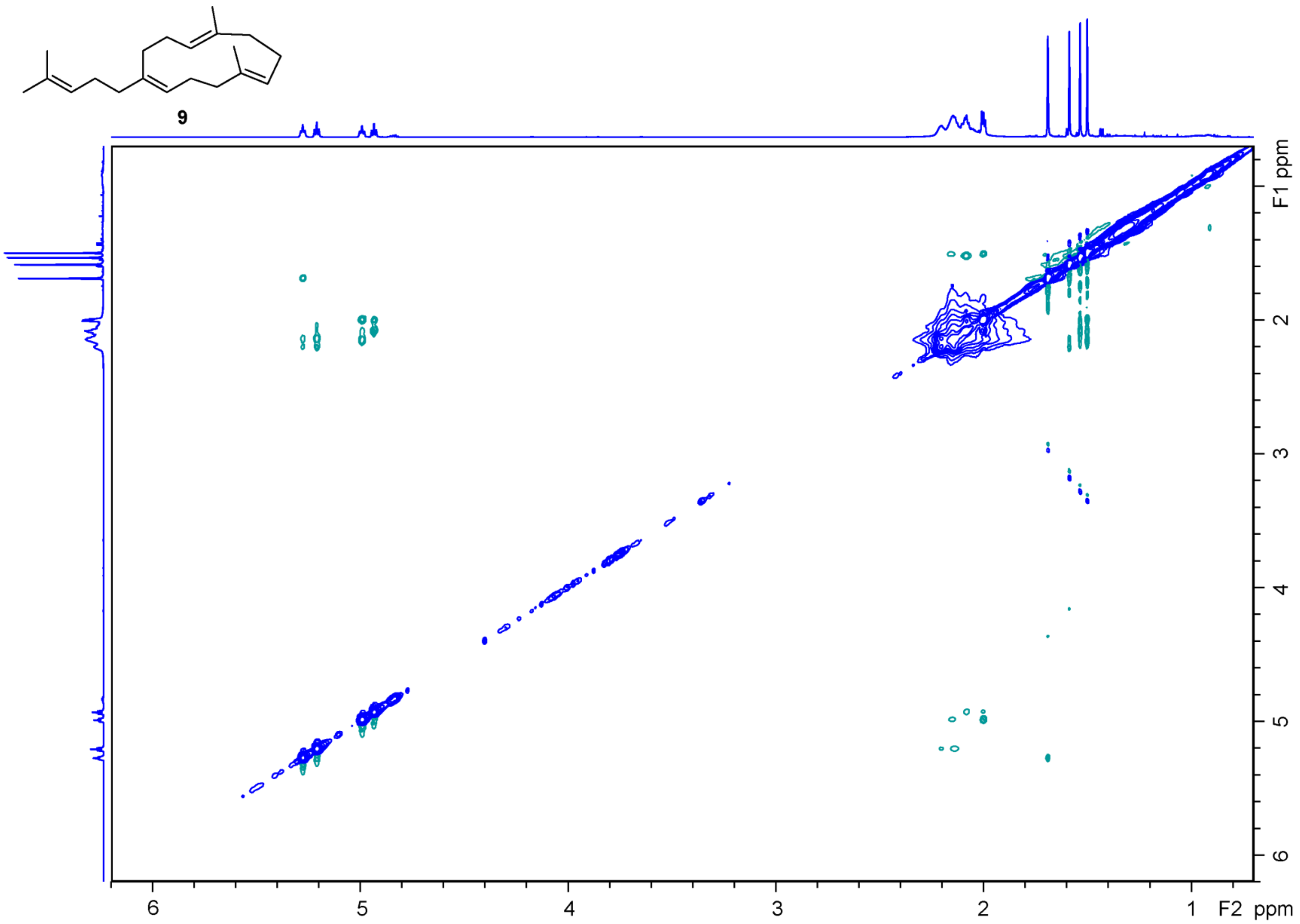

Figure S54. NOESY spectrum (C_6D_6) of **9**.

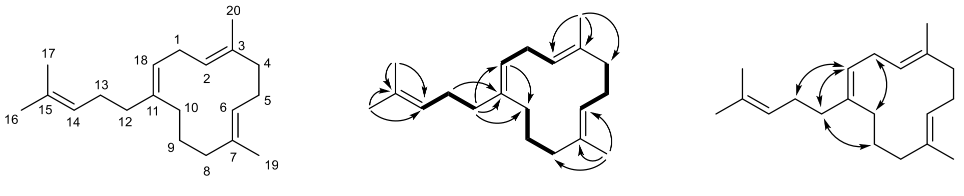

10

Figure S55. Structure elucidation of prenylpseudogermacrene D (**10**). Bold: ^{1}H,^{1}H-COSY, single-headed arrows: key HMBC, and double-headed arrows: key NOESY correlations.

Table S8. NMR data of prenylpseudogermacrene D (**10**) in C_6D_6 recorded at 298 K.

C[a]	type	^{13}C[b]	^{1}H[b]
1	CH_2	26.96	2.68 (t, ^{3}J = 6.9)
2	CH	126.99	5.01 (t, ^{3}J = 6.8)
3	C_q	131.71	–
4	CH_2	39.43	2.04 (m, 2H)
5	CH_2	25.15	2.13 (m, 2H)
6	CH	125.86	4.96 (t, ^{3}J = 7.8)
7	C_q	133.42	–
8	CH_2	37.37	1.95 (m, 2H)
9	CH_2	26.08	1.54 (m, 2H)
10	CH_2	26.57	2.05 (m, 2H)
11	C_q	142.41	–
12	CH_2	36.33	2.14 (m, 2H)
13	CH_2	27.34	2.21 (m, 2H)
14	CH	125.17	5.29 (thept, ^{3}J = 6.8, ^{4}J = 1.4)
15	C_q	131.13	–
16	CH_3	25.90	1.69 (br s)
17	CH_3	17.82	1.59 (br s)
18	CH	122.73	5.50 (t, ^{3}J = 7.3)
19	CH_3	17.45	1.53 (br s)
20	CH_3	15.12	1.46 (br s)

[a] Carbon numbering as shown in Figure S55. [b] Chemical shifts δ in ppm, multiplicity: s = singlet, t = triplet, hept = heptet, m = multiplet, br = broad, coupling constants *J* are given in Hertz.

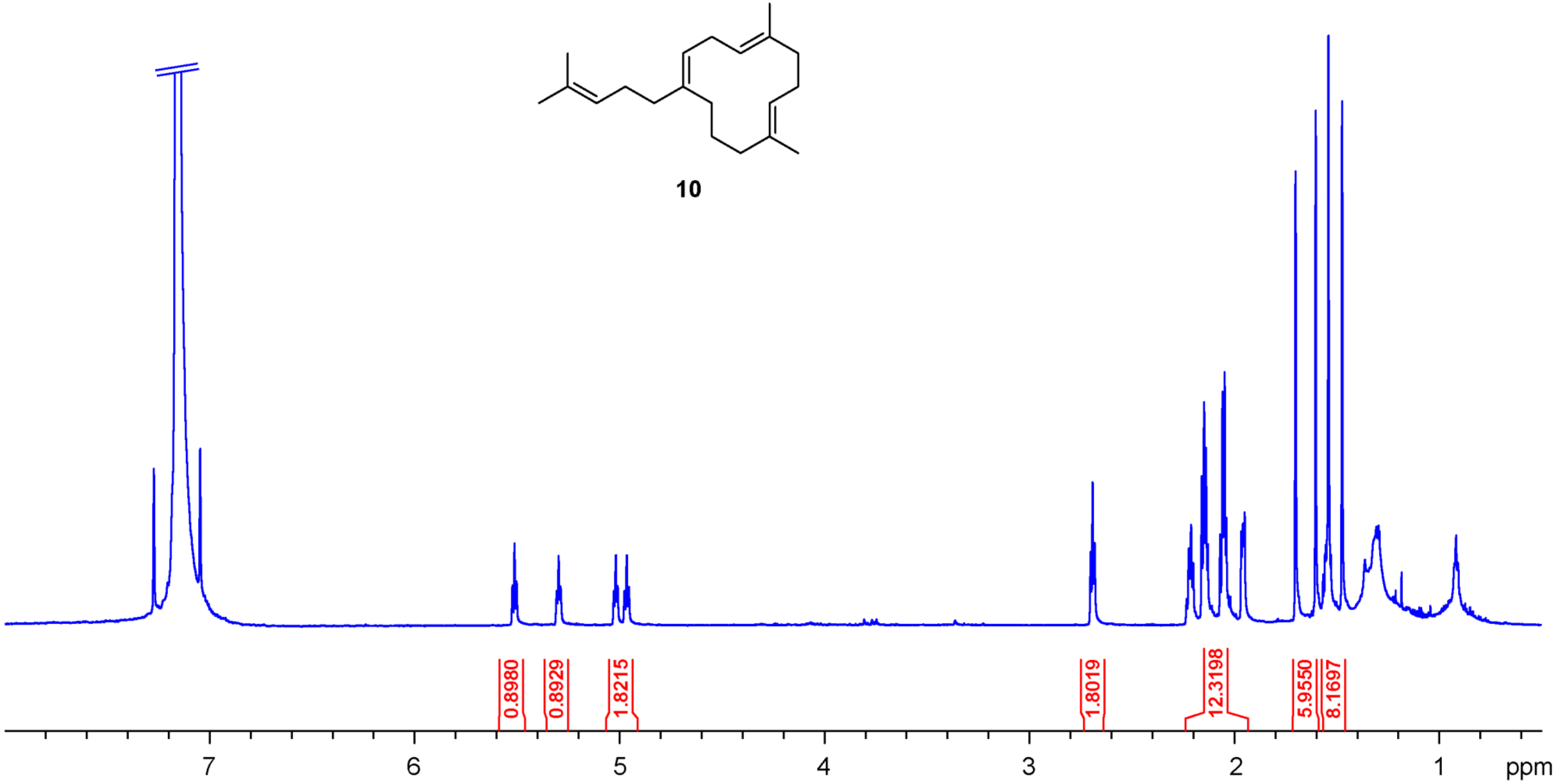

Figure S56. ^{1}H-NMR spectrum (700 MHz, C_6D_6) of **10**.

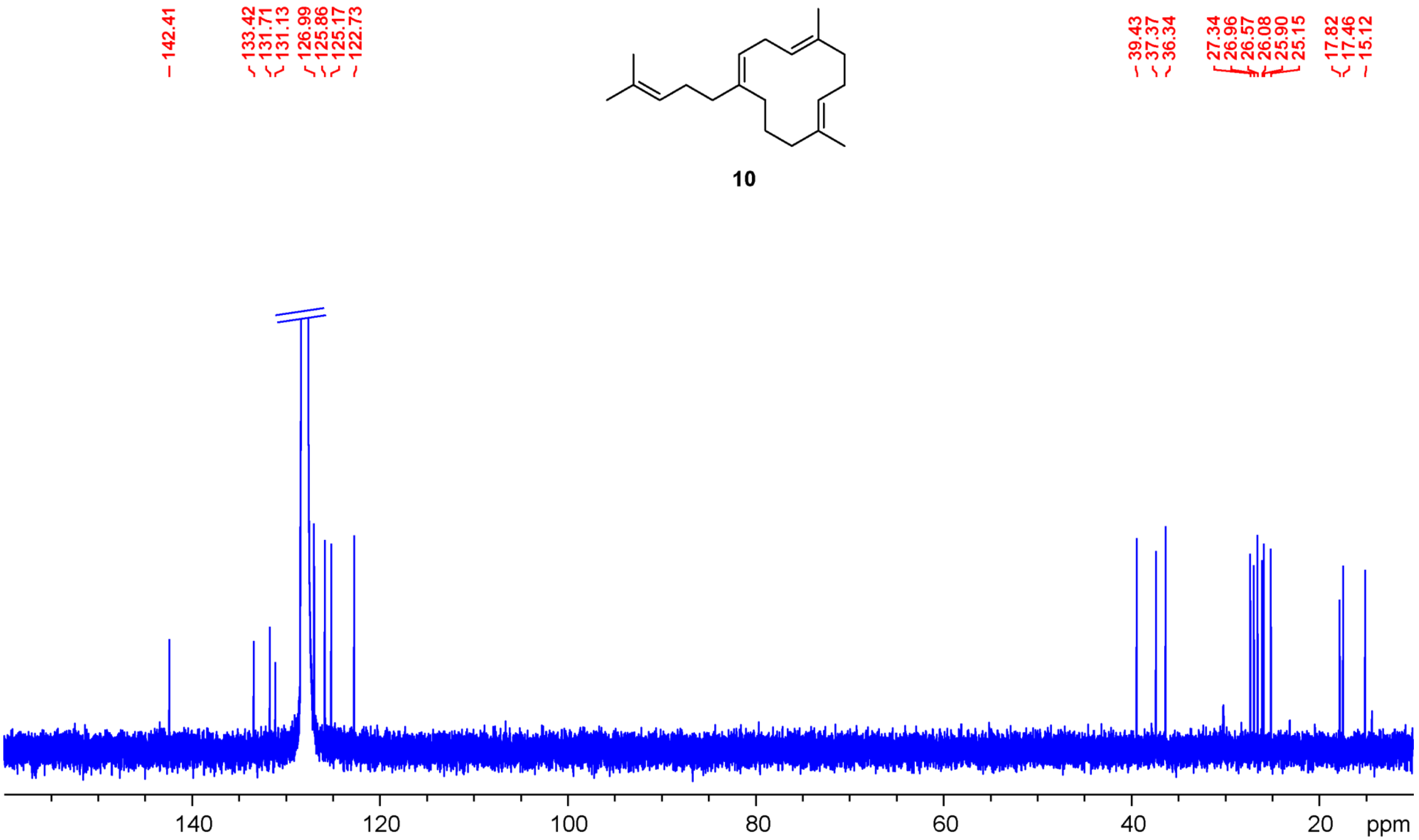

Figure S57. ^{13}C-NMR spectrum (176 MHz, C_6D_6) of **10**.

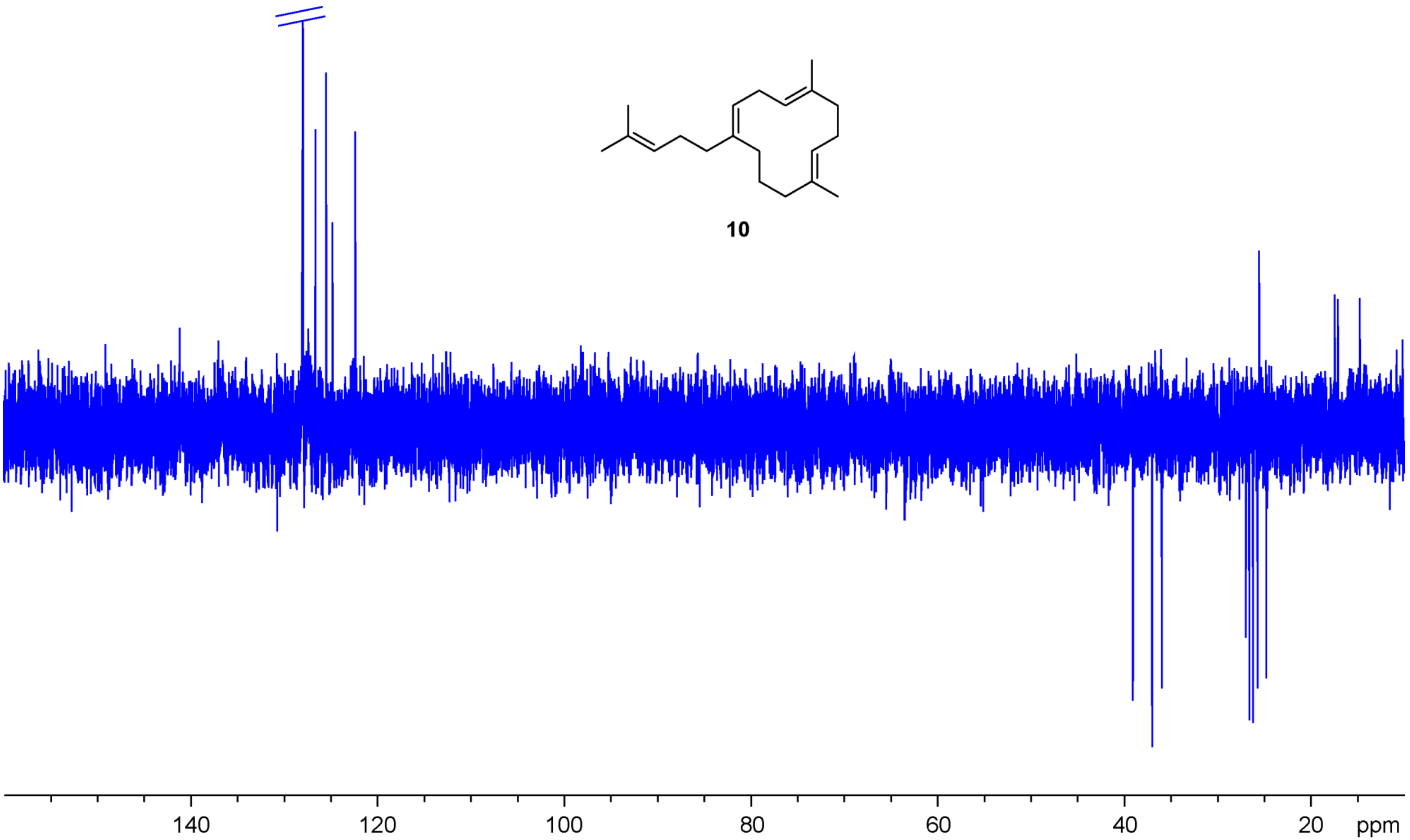

Figure S58. ^{13}C-DEPT135 spectrum (176 MHz, C_6D_6) of **10**.

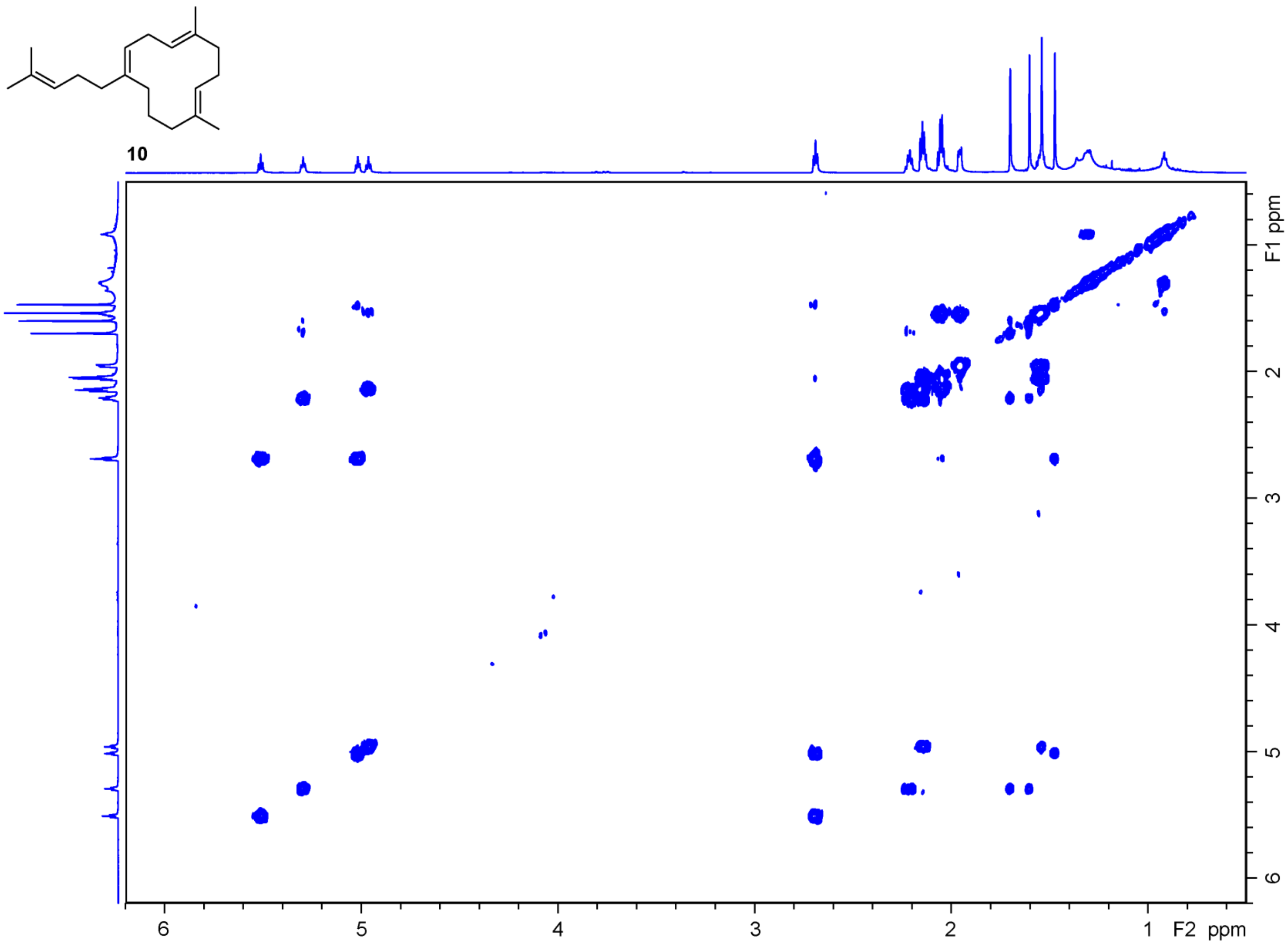

Figure S59. $^{1}H,^{1}H$-COSY spectrum (C_6D_6) of **10**.

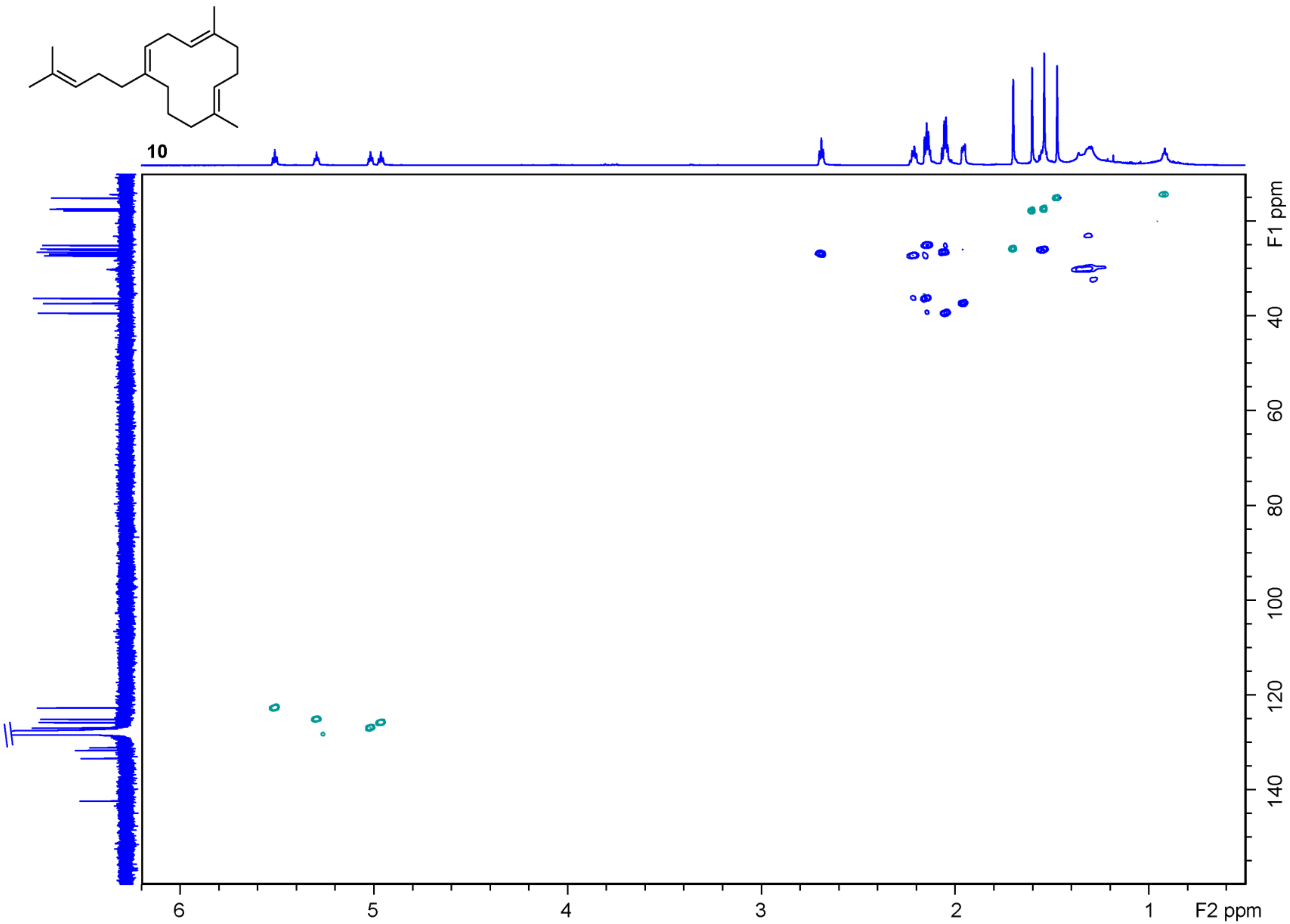

Figure S60. HSQC spectrum (C_6D_6) of **10**.

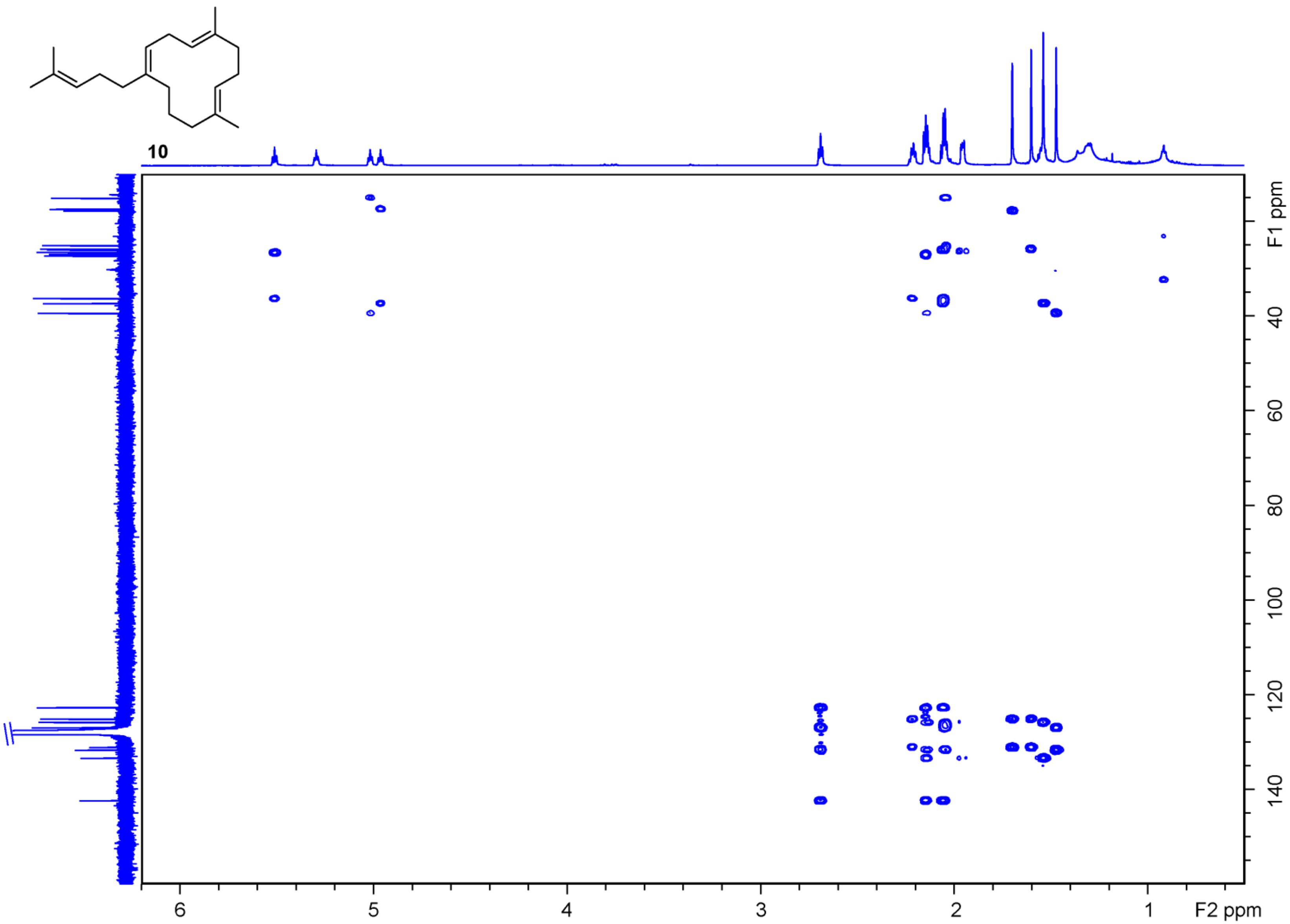

Figure S61. HMBC spectrum (C_6D_6) of **10**.

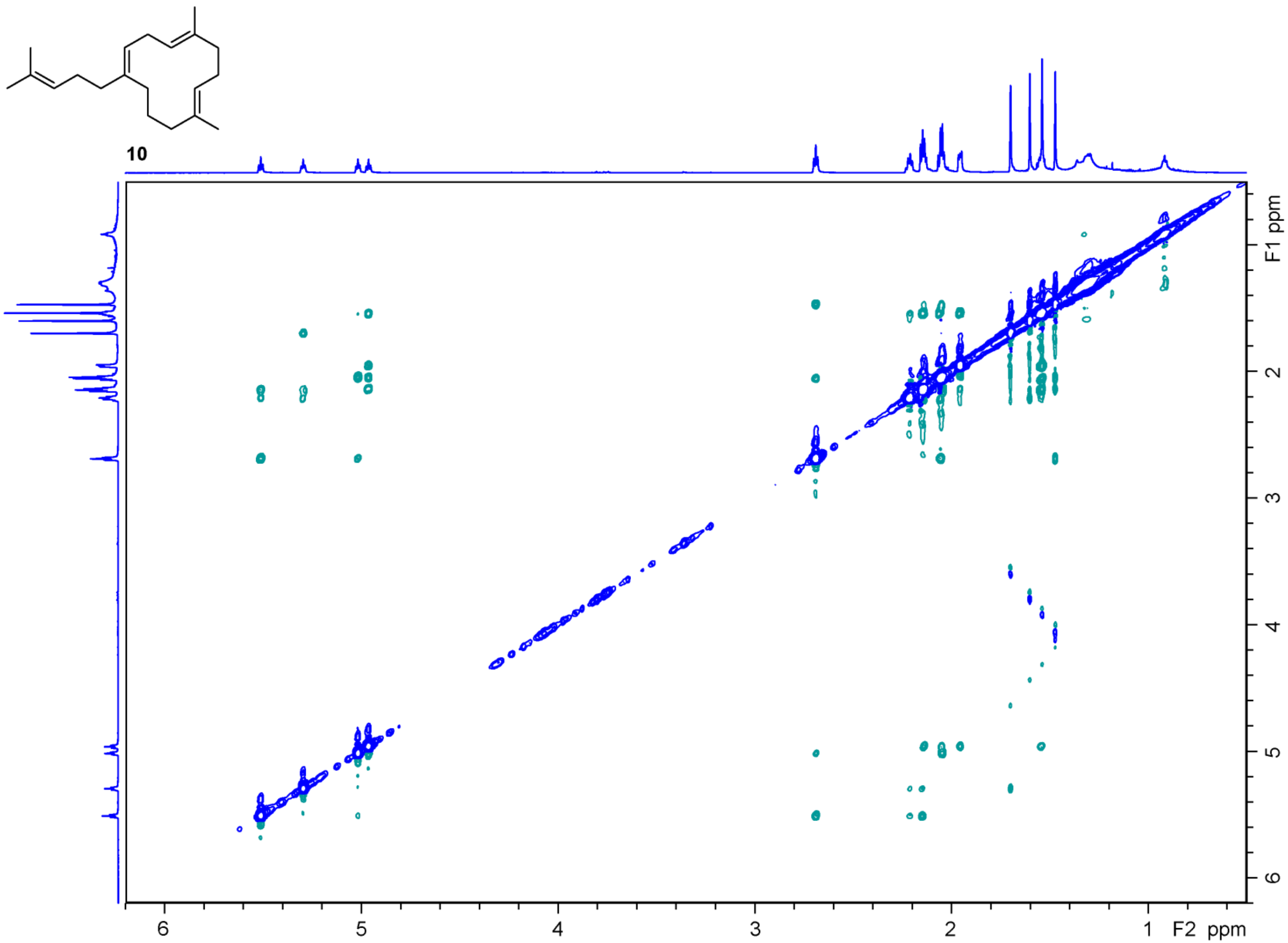

Figure S62. NOESY spectrum (C_6D_6) of **10**.

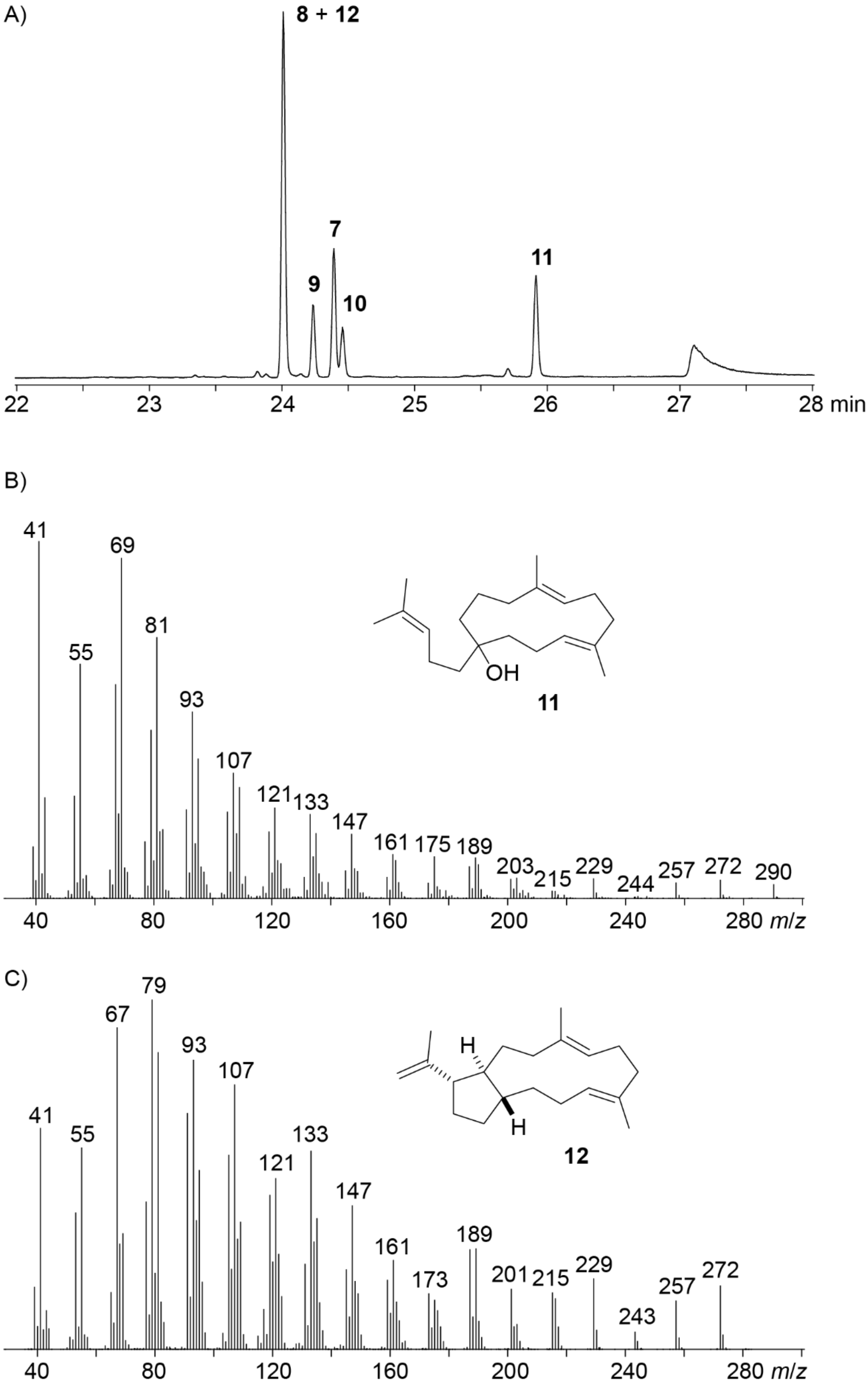

Figure S63. Product mixture formed from iso-FPP and IPP with GGPPS and NrPS. A) Total ion chromatogram of the crude extract from the enzyme incubation, B) EI mass spectrum of **11**, C) EI mass spectrum of **12**.

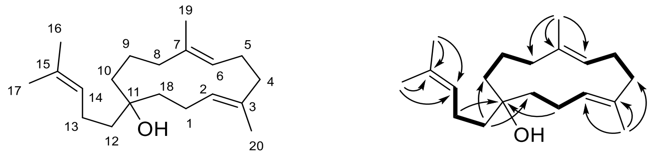

11

Figure S64. Structure elucidation of prenylpseudohedycaryol (**11**). Bold: ^{1}H,^{1}H-COSY, single-headed arrows: key HMBC, and double-headed arrows: key NOESY correlations.

Table S9. NMR data of prenylpseudohedycaryol (**11**) in C_6D_6 recorded at 298 K.

C[a]	type	^{13}C[b]	^{1}H[b]
1	CH_2	23.62	2.38 (m)
			1.95 (m)
2	CH	129.30	5.03 (br t, ^{3}J = 7.5)
3	C_q	132.58	–
4	CH_2	40.10	2.03 (m, 2H)
5	CH_2	25.77	2.19 (m)
			2.07 (m)
6	CH	126.77	4.87 (br t, ^{3}J = 7.3)
7	C_q	134.32	–
8	CH_2	38.90	1.91 (dt, ^{4}J = 3.9, ^{3}J = 7.3)
9	CH_2	21.94	1.32 (m)
			1.21 (m)
10	CH_2	36.55	1.51 (m)
			1.37 (m)
11	C_q	74.12	–
12	CH_2	41.32	1.46 (m)
			1.42 (m)
13	CH_2	22.61	2.13 (m)
14	CH	125.64	5.27 (thept, ^{3}J = 7.2, ^{4}J = 1.3)
15	C_q	131.11	–
16	CH_3	25.90	1.70 (br s)
17	CH_3	17.74	1.62 (br s)
18	CH_2	37.75	1.49 (m)
			1.43 (m)
19	CH_3	17.23	1.51 (br s)
20	CH_3	15.17	1.49 (br s)

[a] Carbon numbering as shown in Figure S64. [b] Chemical shifts δ in ppm, multiplicity: s = singlet, d = doublet, t = triplet, hept = heptet, m = multiplet, br = broad, coupling constants J are given in Hertz.

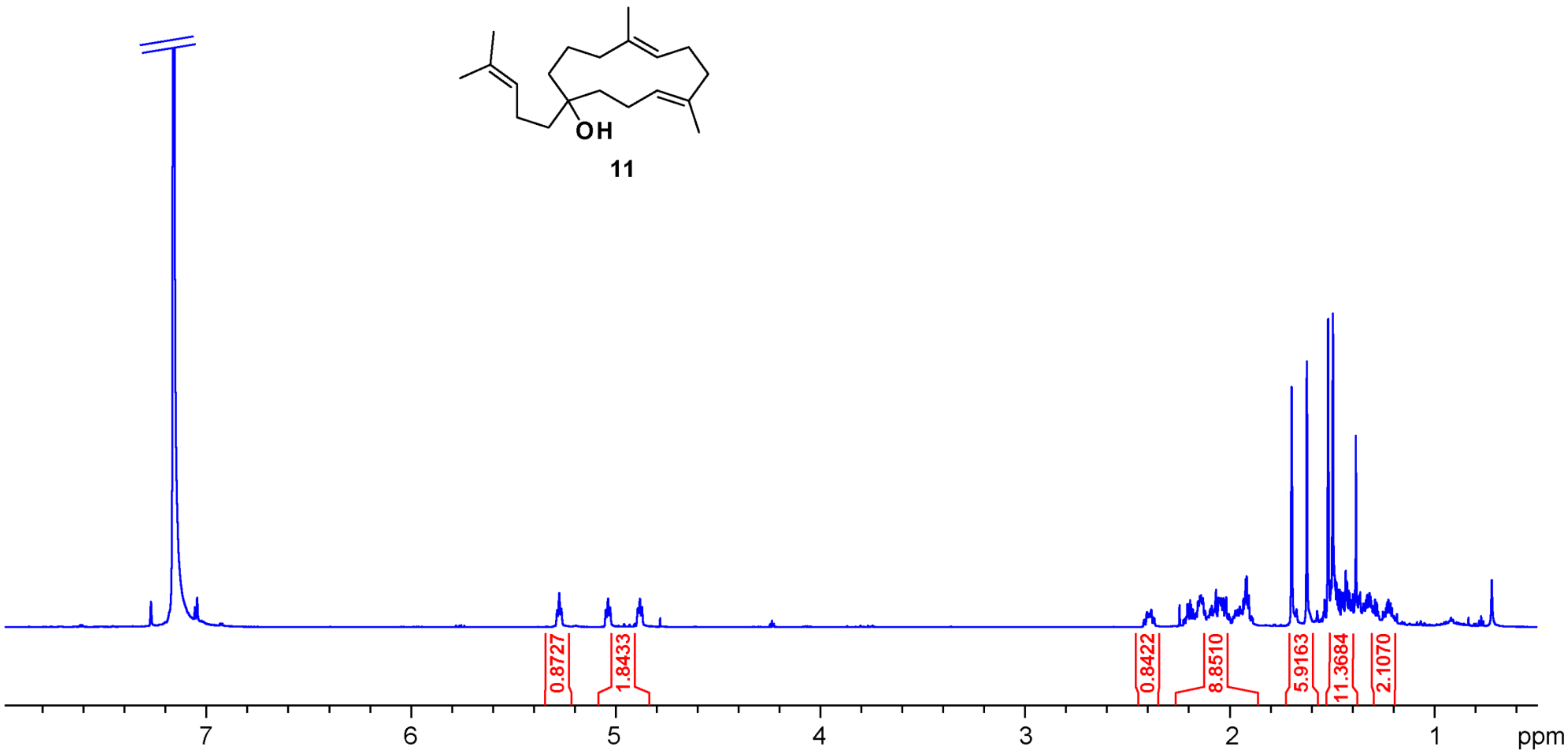

Figure S65. ^{1}H-NMR spectrum (700 MHz, C_6D_6) of **11**.

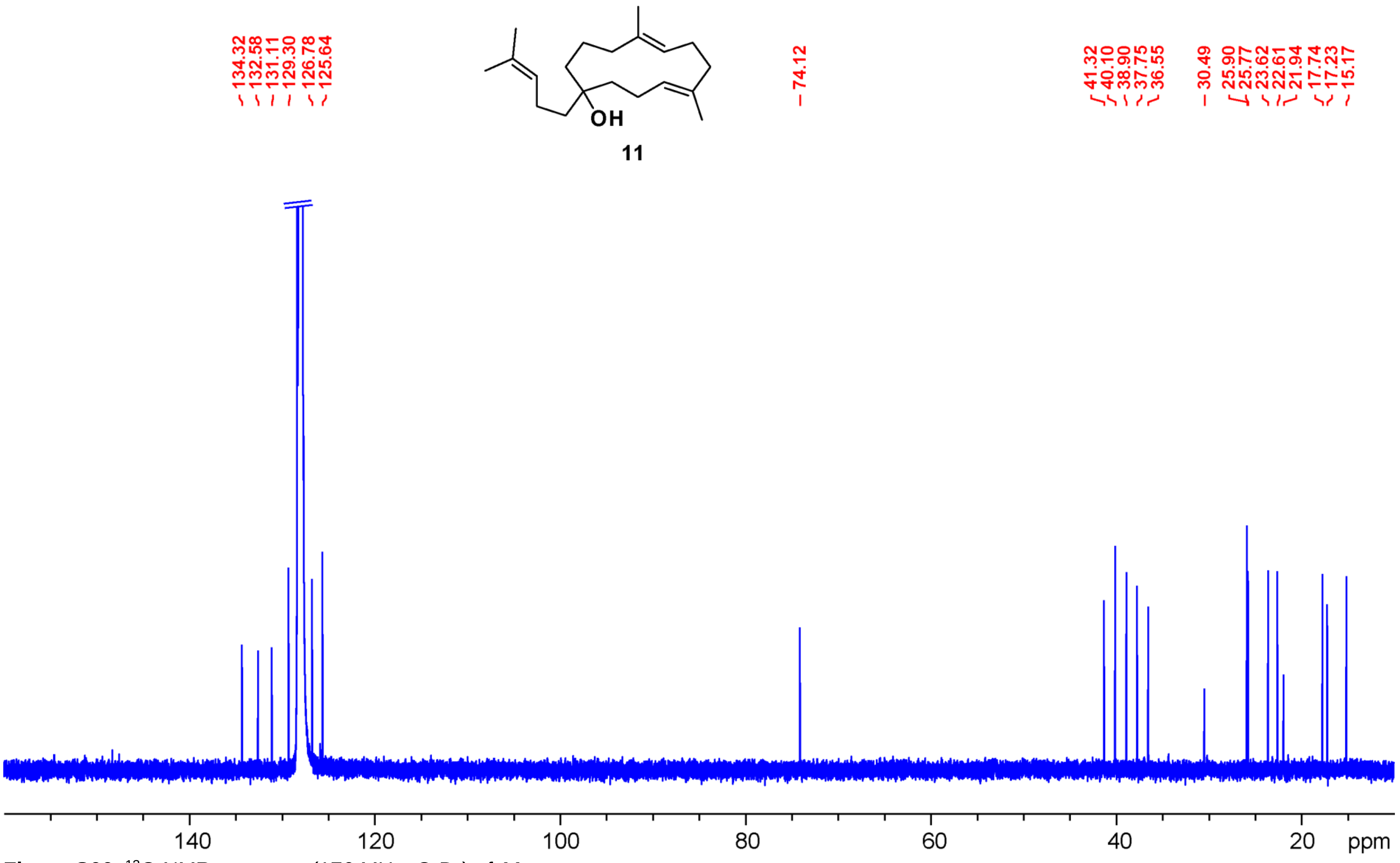

Figure S66. ^{13}C-NMR spectrum (176 MHz, C_6D_6) of **11**.

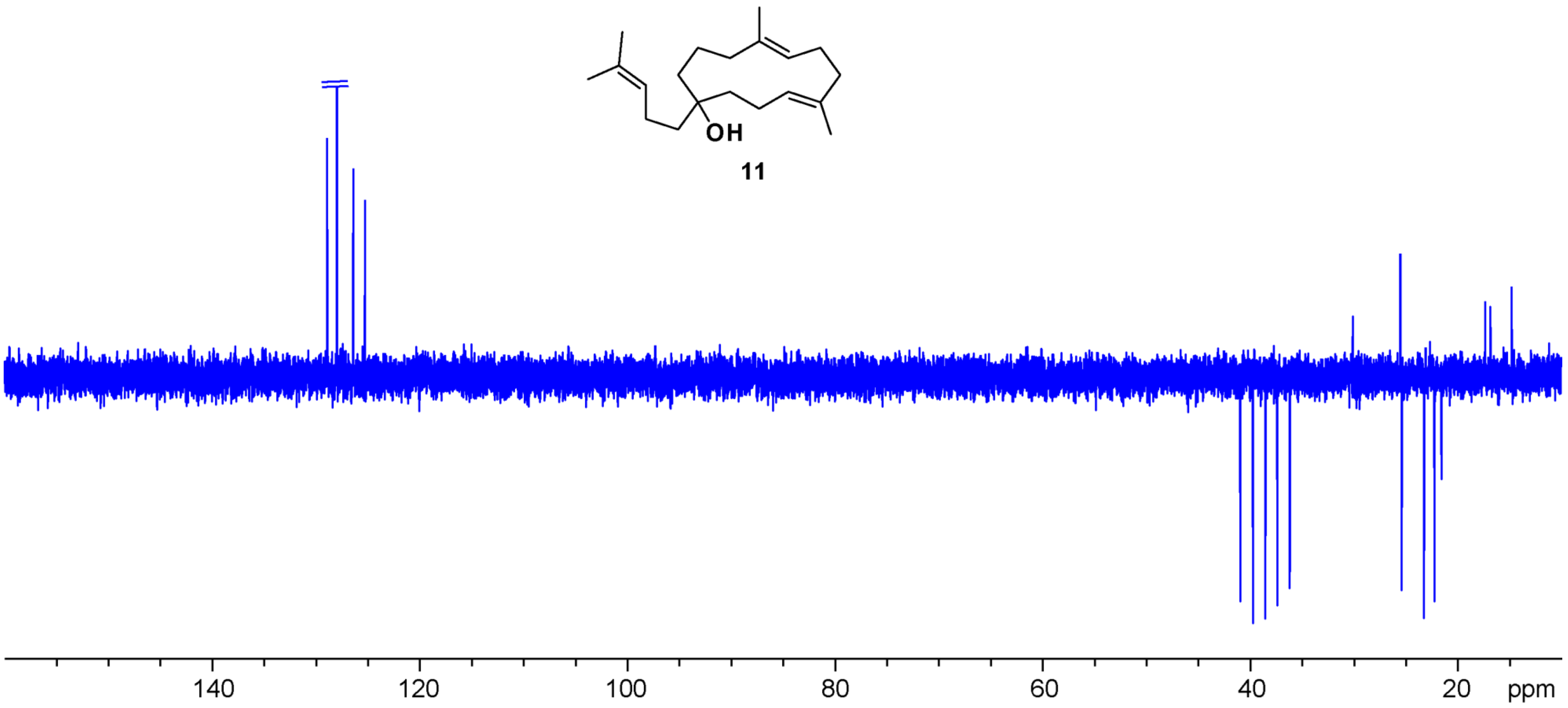

Figure S67. ^{13}C-DEPT135 spectrum (176 MHz, C_6D_6) of **11**.

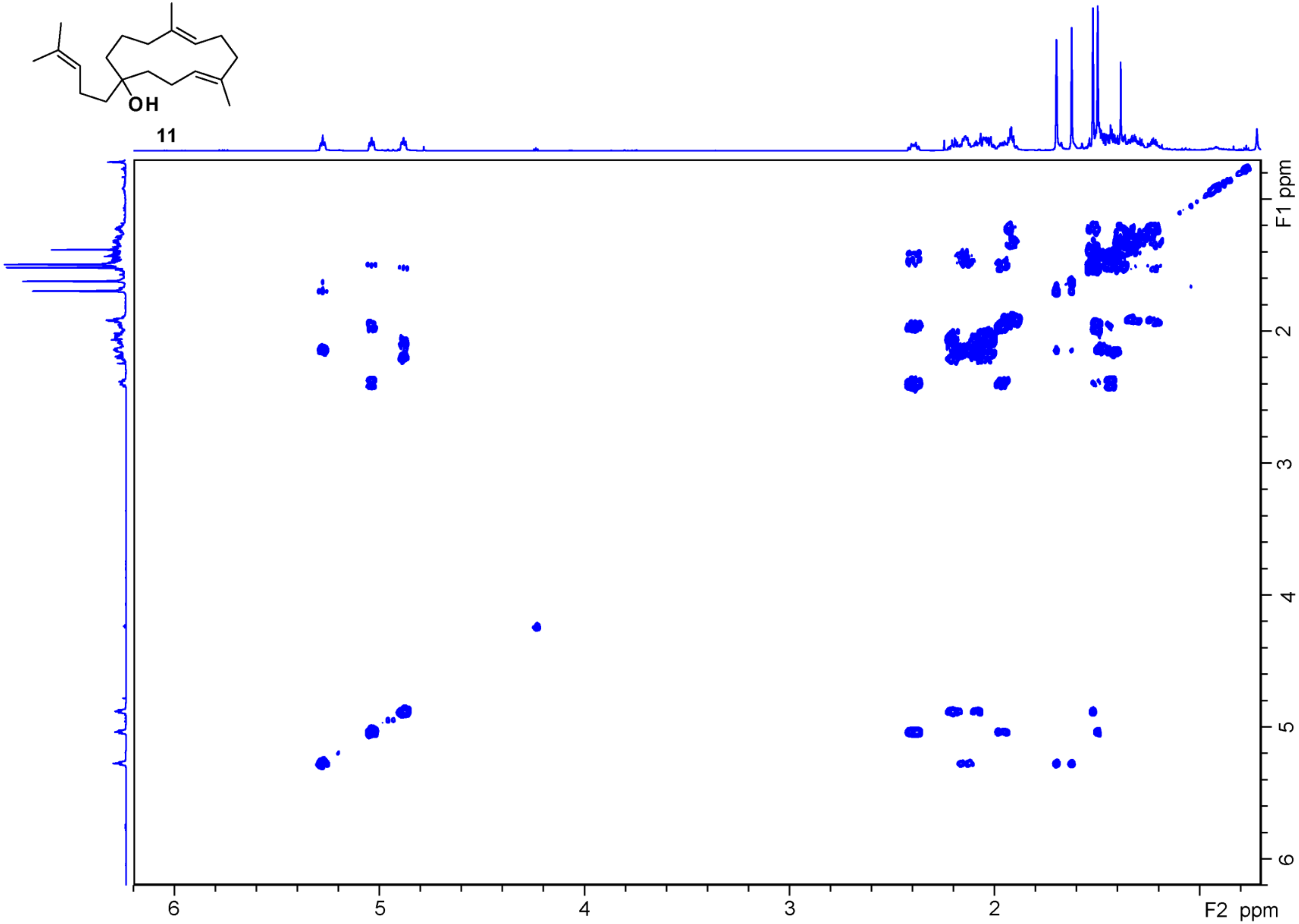

Figure S68. $^{1}H,^{1}H$-COSY spectrum (C_6D_6) of **11**.

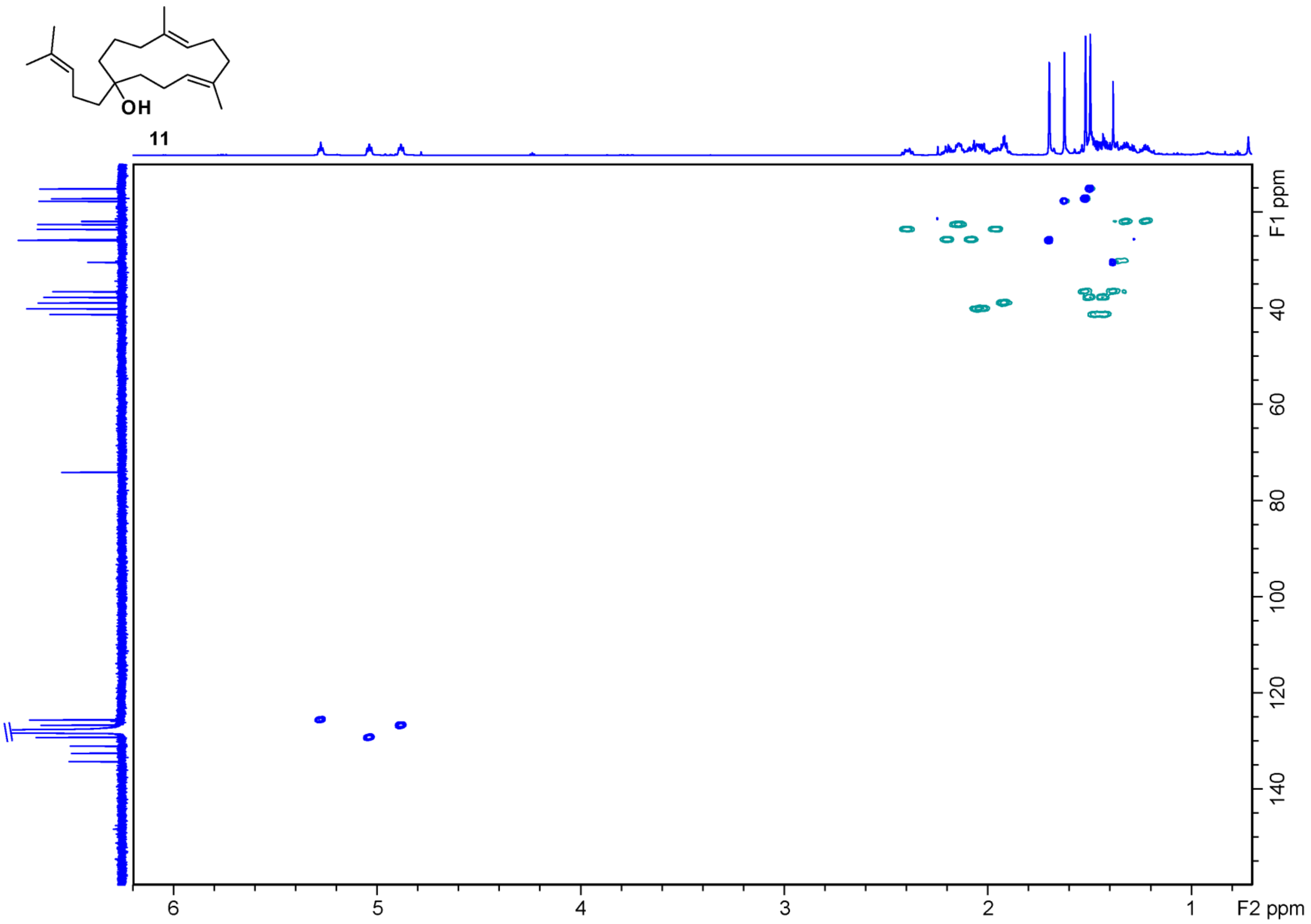

Figure S69. HSQC spectrum (C_6D_6) of **11**.

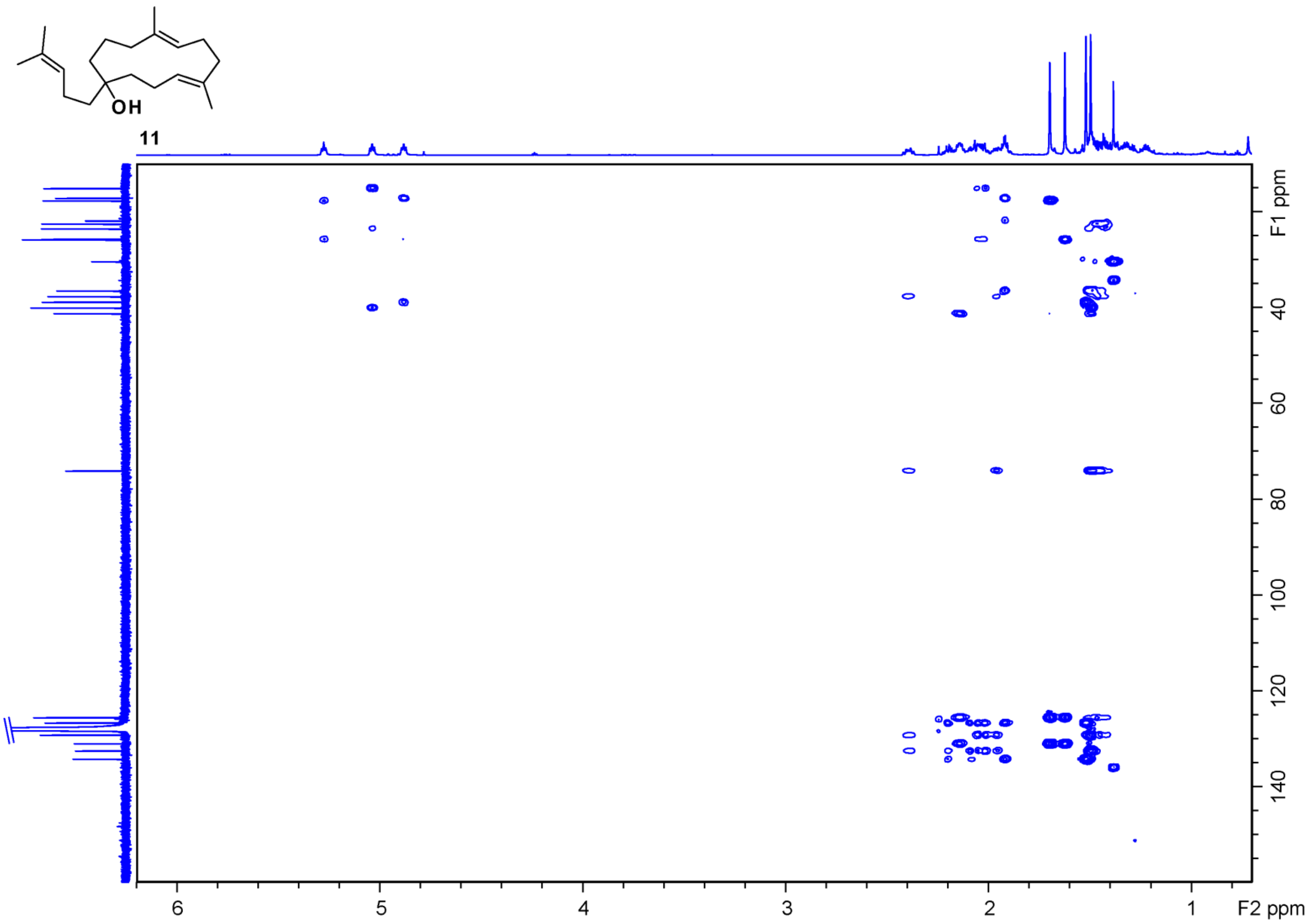

Figure S70. HMBC spectrum (C_6D_6) of **11**.

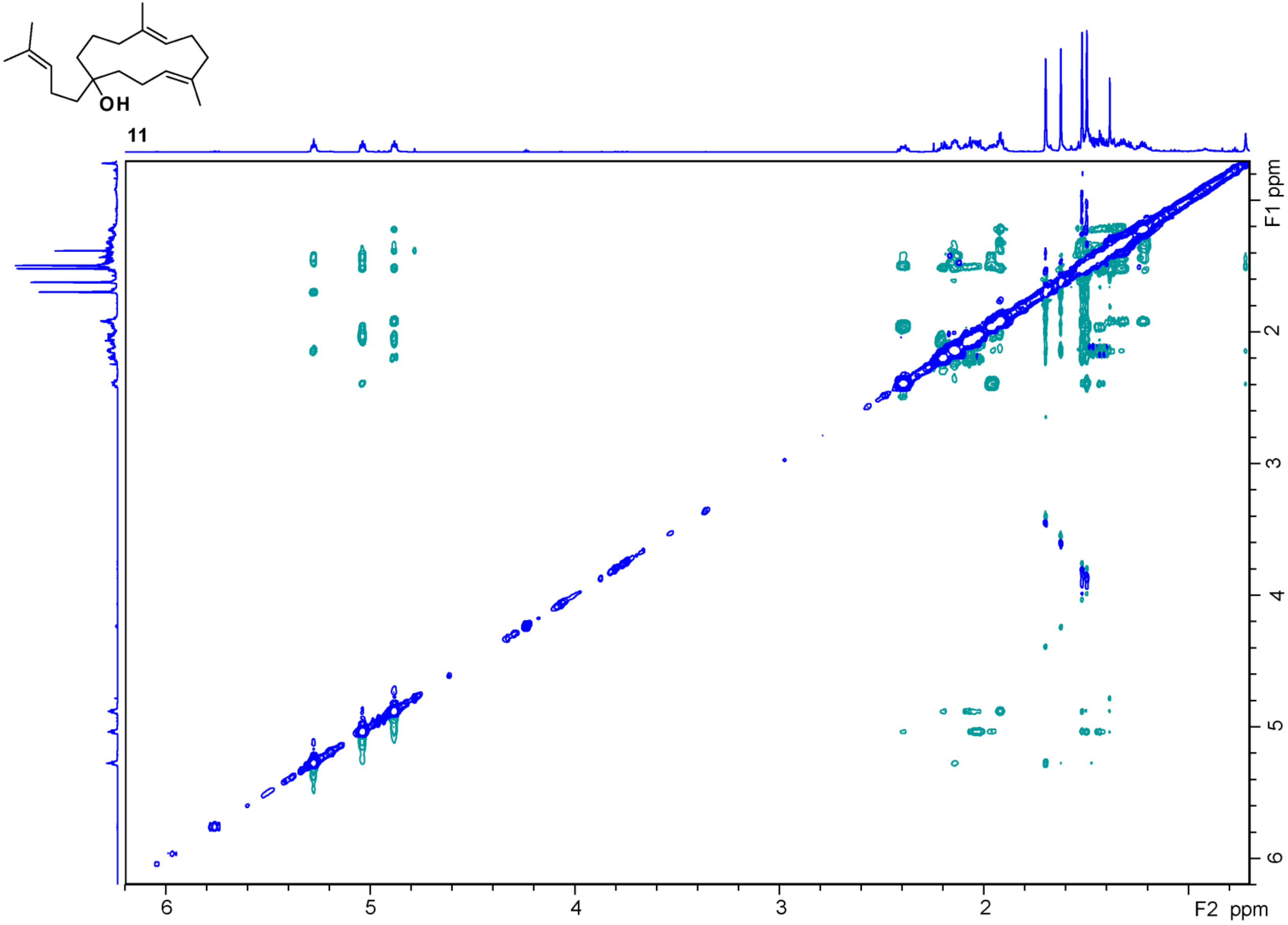

Figure S71. NOESY spectrum (C_6D_6) of **11**.

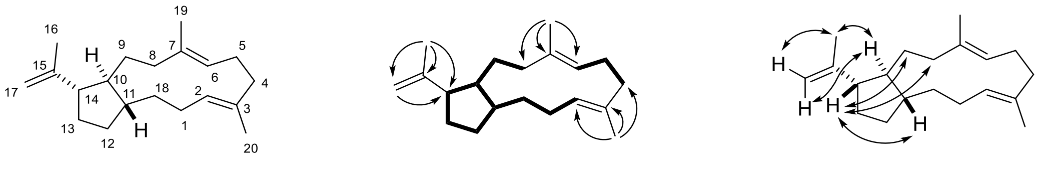

12

Figure S72. Structure elucidation of pseudodollabella-3,7,18-triene (**12**). Bold: $^{1}H,^{1}H$-COSY, single-headed arrows: key HMBC, and double-headed arrows: key NOESY correlations.

Table S10. NMR data of pseudodollabella-3,7,18-triene (**12**) in C_6D_6 recorded at 298 K.

C[a]	type	^{13}C[b]	^{1}H[b]
1	CH_2	25.45	2.09 (m)
			1.96 (m)
2	CH	127.52	5.17 (t, ^{3}J = 7.8)
3	C_q	133.77	–
4	CH_2	39.55	2.08 (m, 2H)
5	CH_2	25.72	2.13 (m)
			2.09 (m)
6	CH	125.79	4.96 (t, ^{3}J = 7.1)
7	C_q	135.66	–
8	CH_2	36.45	2.10 (m)
			2.03 (m)
9	CH_2	27.60	1.49 (m, 2H)
10	CH	49.11	1.54 (m)
11	CH	40.19	1.89 (m)
12	CH_2	30.15	1.31 (m, 2H)
13	CH_2	30.09	1.68 (m)
			1.55 (m)
14	CH	54.07	2.28 (q, ^{3}J = 9.0)
15	C_q	148.33	–
16	CH_2	110.54	4.87 (m, H_Z)
			4.81 (dq, ^{4}J = 1.4, 1.1, H_E)
17	CH_3	19.52	1.67 (br s)
18	CH_2	34.85	1.32 (m)
			1.24 (m)
19	CH_3	16.07	1.54 (br s)
20	CH_3	15.12	1.48 (br s)

[a] Carbon numbering as shown in Figure S72. [b] Chemical shifts δ in ppm, multiplicity: s = singlet, d = doublet, t= triplet, q = quartet, m = multiplet, br = broad, coupling constants J are given in Hertz.

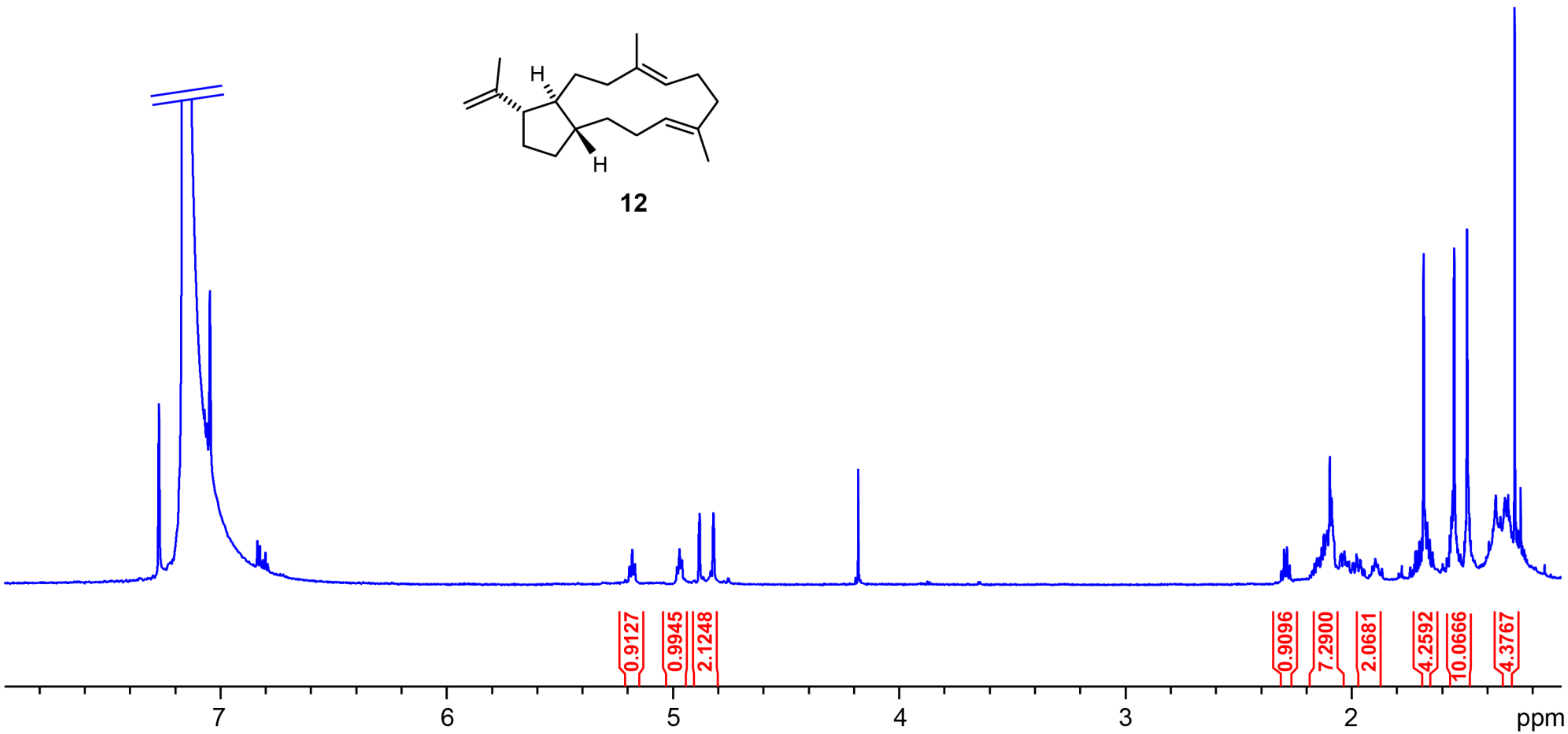

Figure S73. ^{1}H-NMR spectrum (700 MHz, C_6D_6) of **12**.

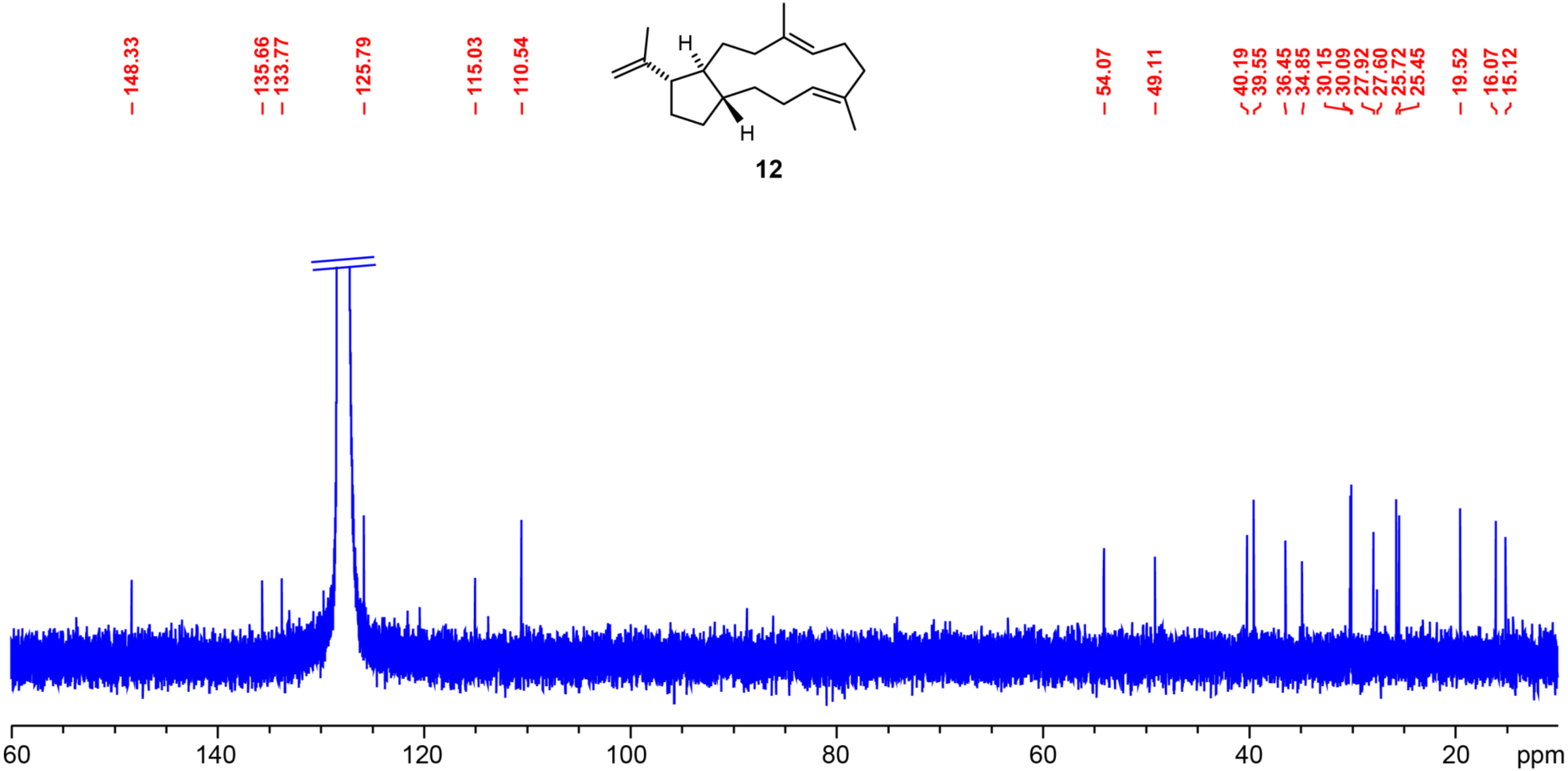

Figure S74. ^{13}C-NMR spectrum (176 MHz, C_6D_6) of **12**.

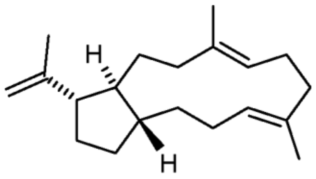

12

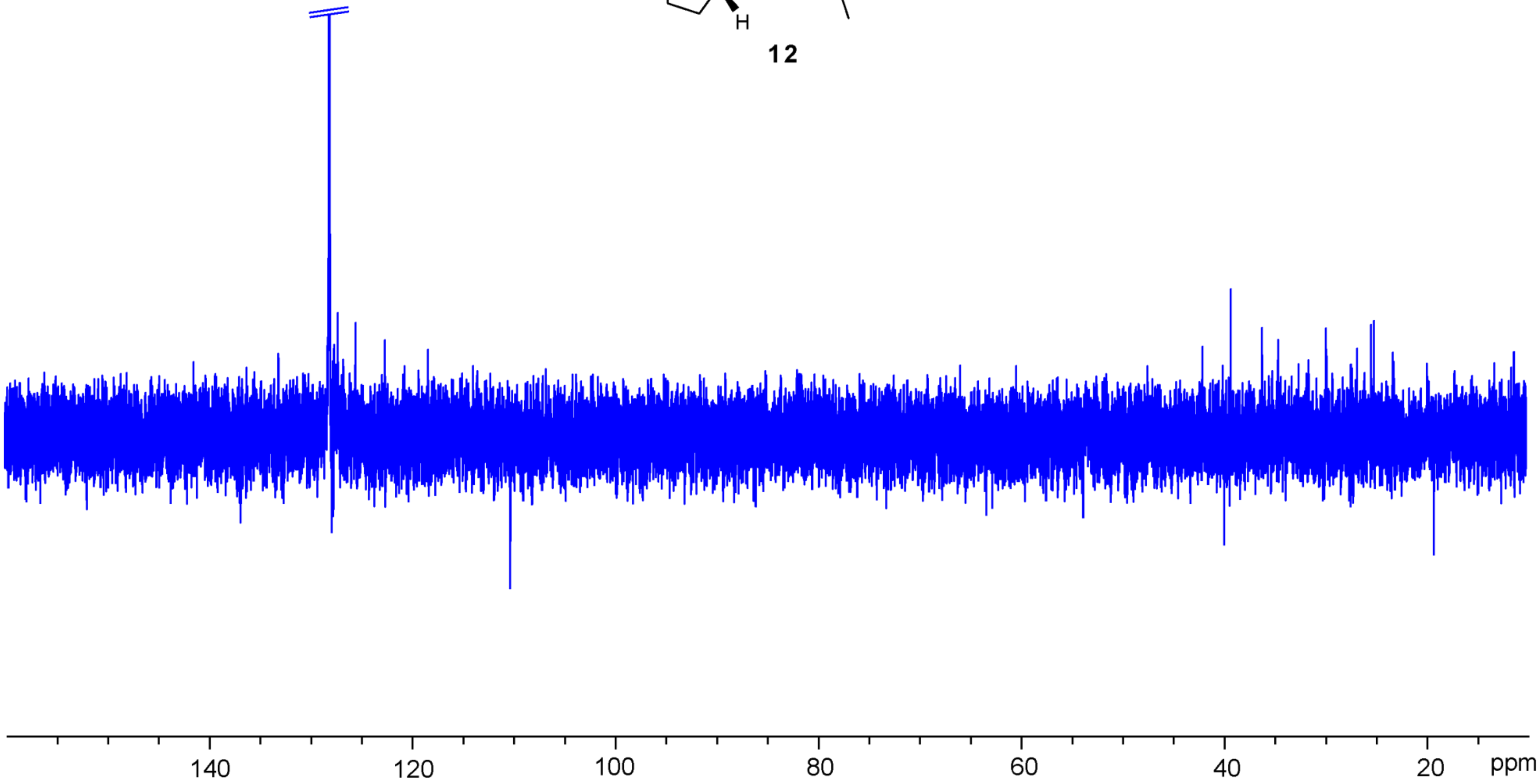

Figure S75. ^{13}C-DEPT135 spectrum (176 MHz, C_6D_6) of **12**.

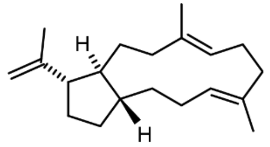

12

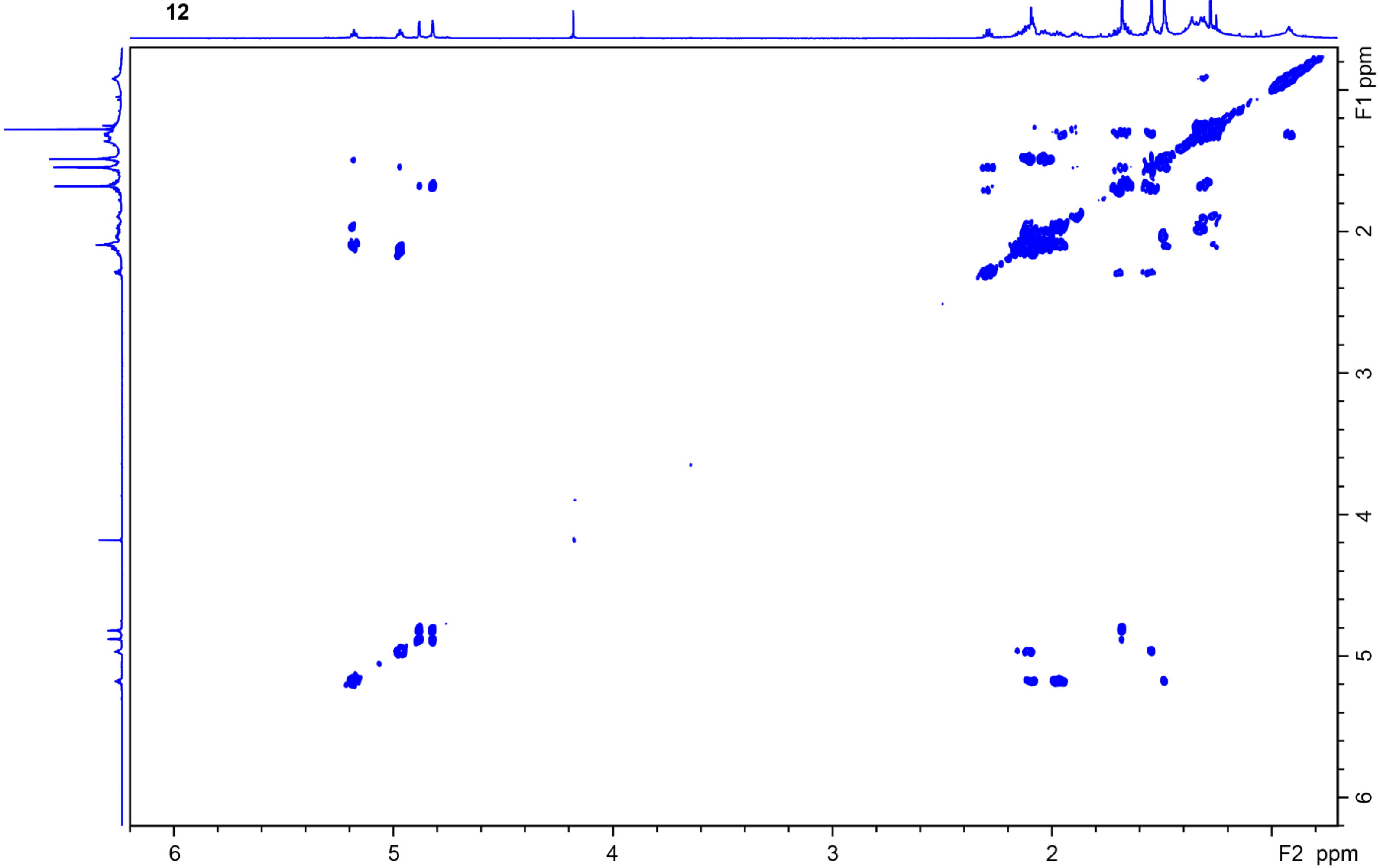

Figure S76. $^{1}H,^{1}H$-COSY spectrum (C_6D_6) of **12**.

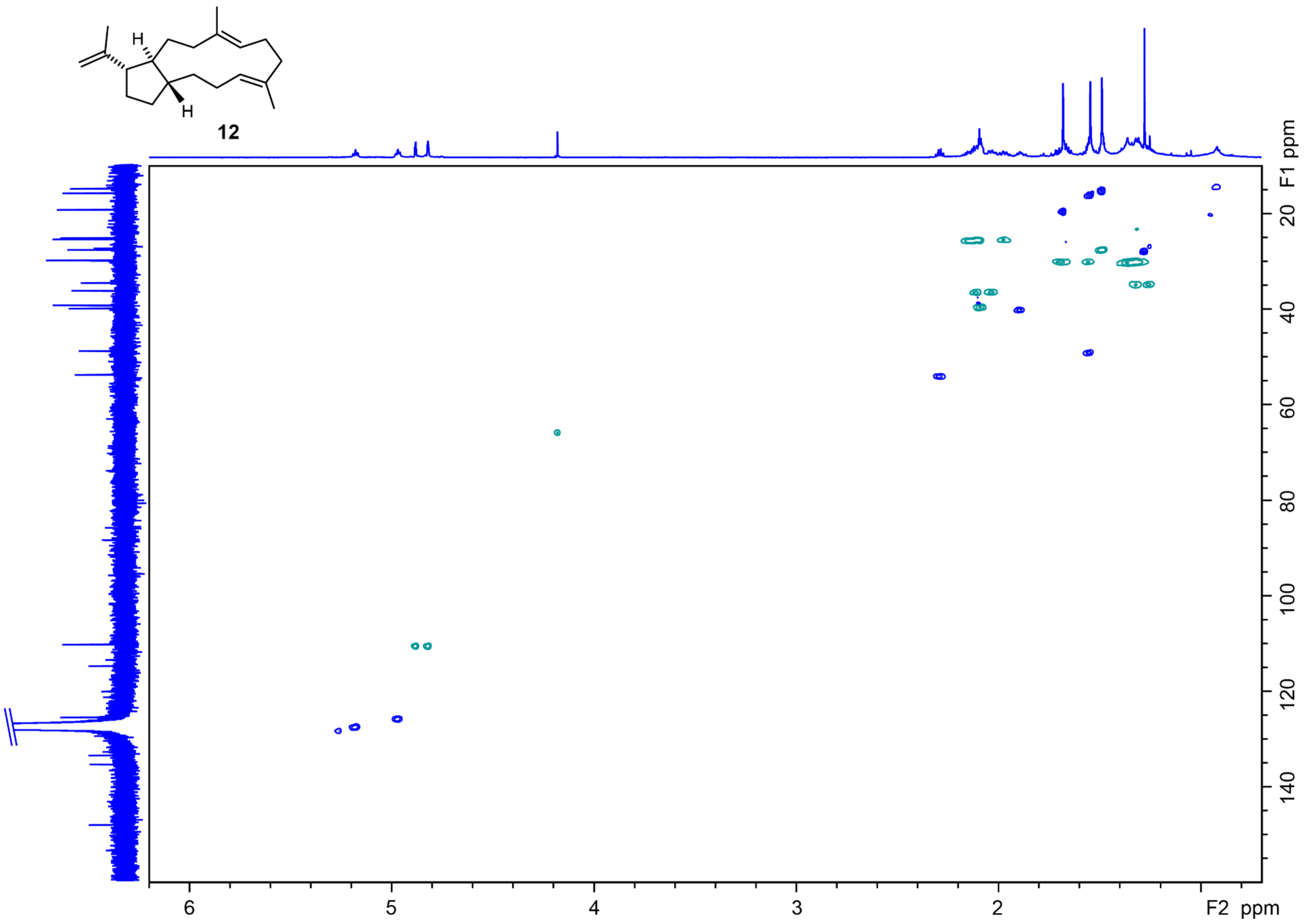

Figure S77. HSQC spectrum (C_6D_6) of **12**.

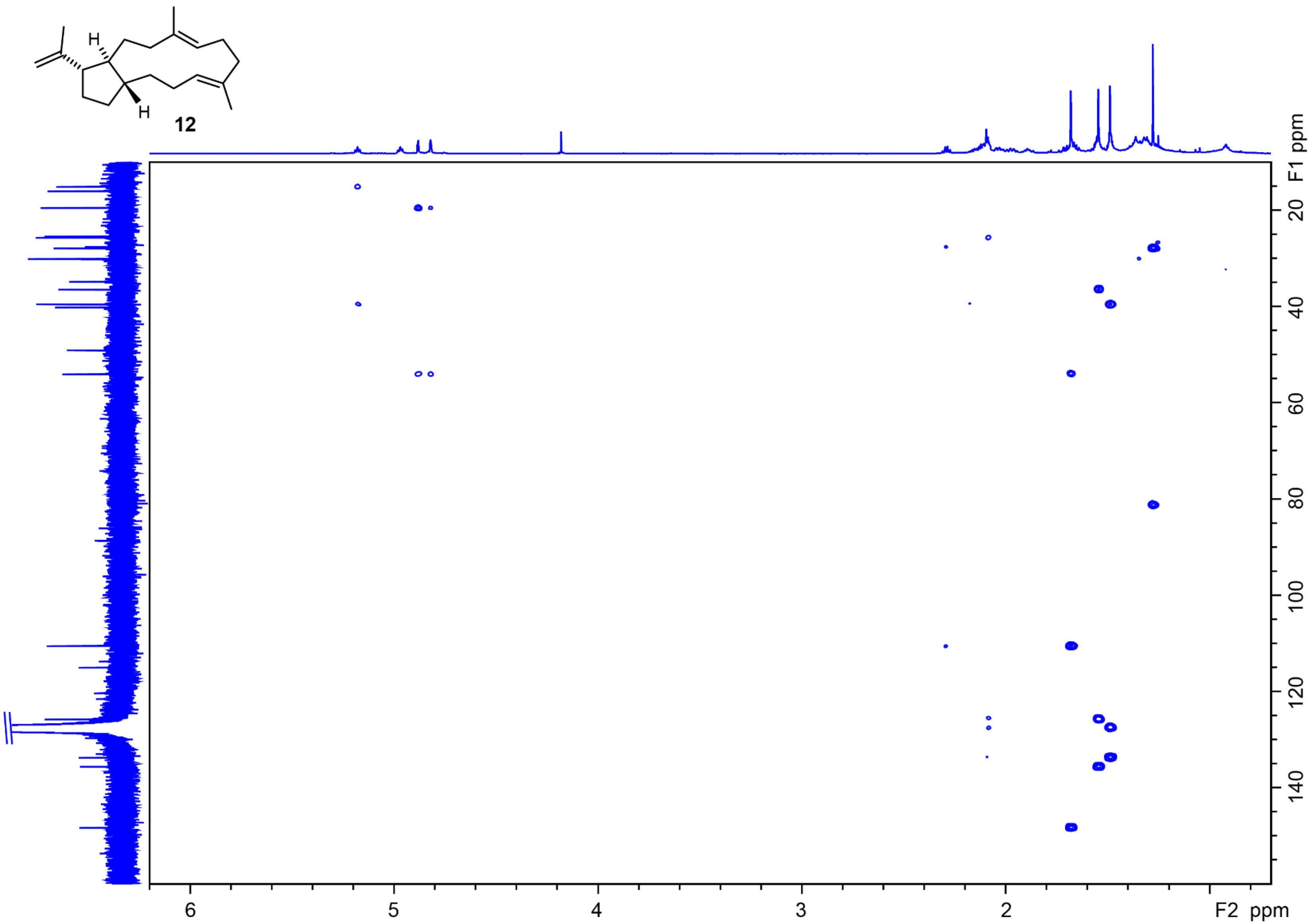

Figure S78. HMBC spectrum (C_6D_6) of **12**.

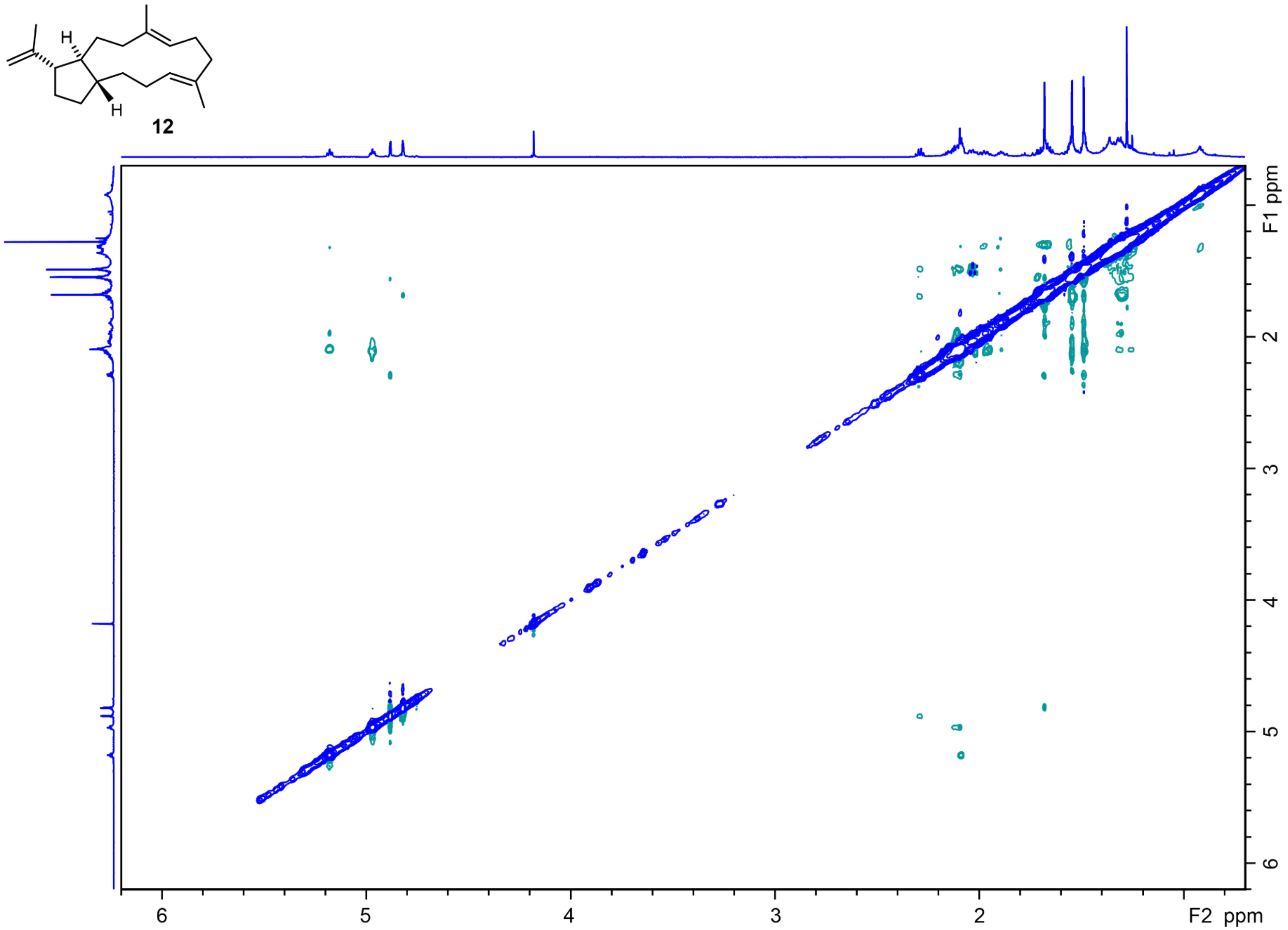

Figure S79. NOESY spectrum (C_6D_6) of **12**.

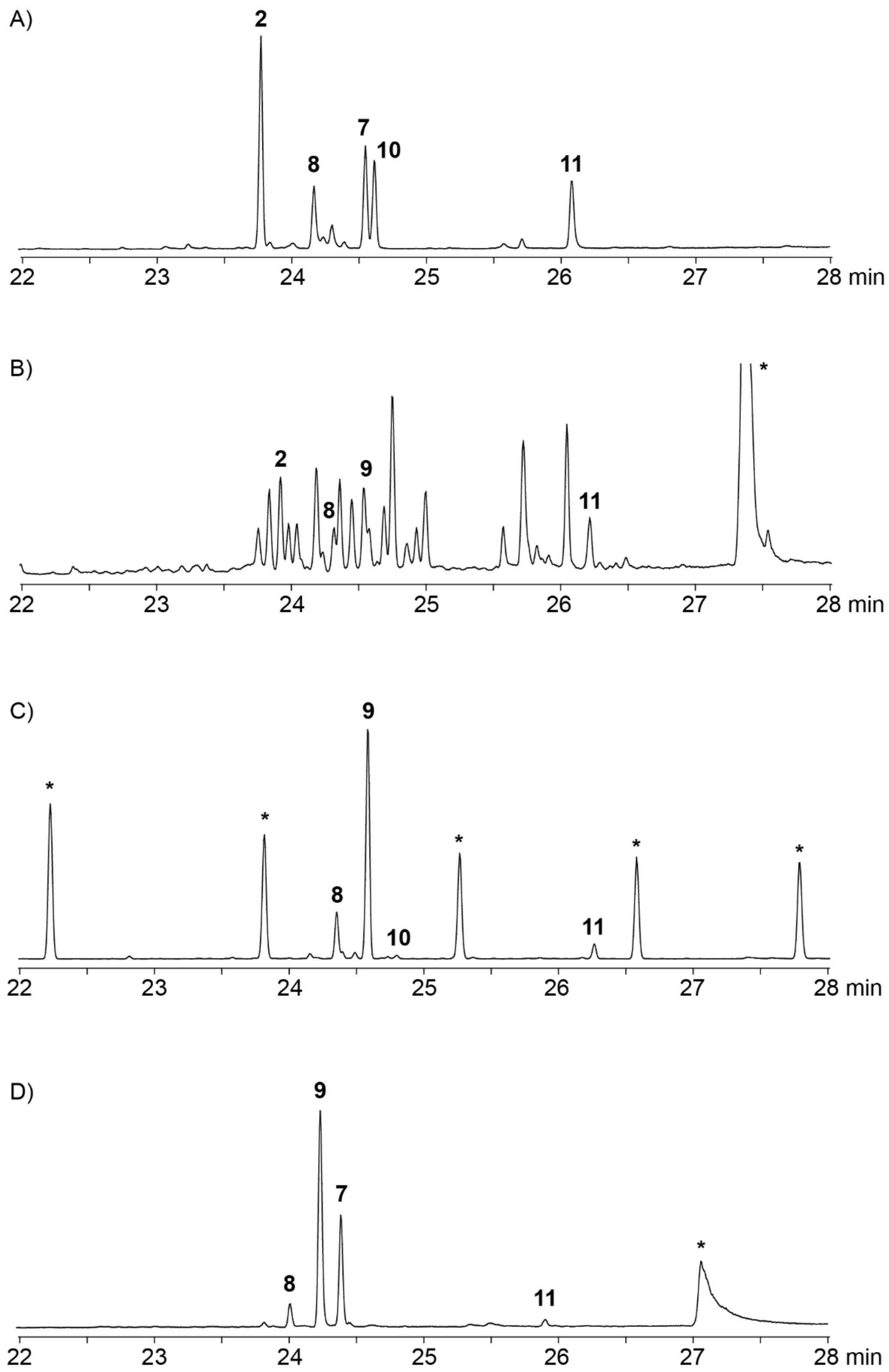

Figure S80. Product mixture formed from iso-FPP and IPP with GGPPS and A) HdS, B) PcS, C) TdS, and D) SvS. Retention times of compounds may vary, because the GC/MS measurements were performed at different times using GC columns of different ages. Asterisks indicate contaminants, unlabelled peaks represent unknown diterpenes.

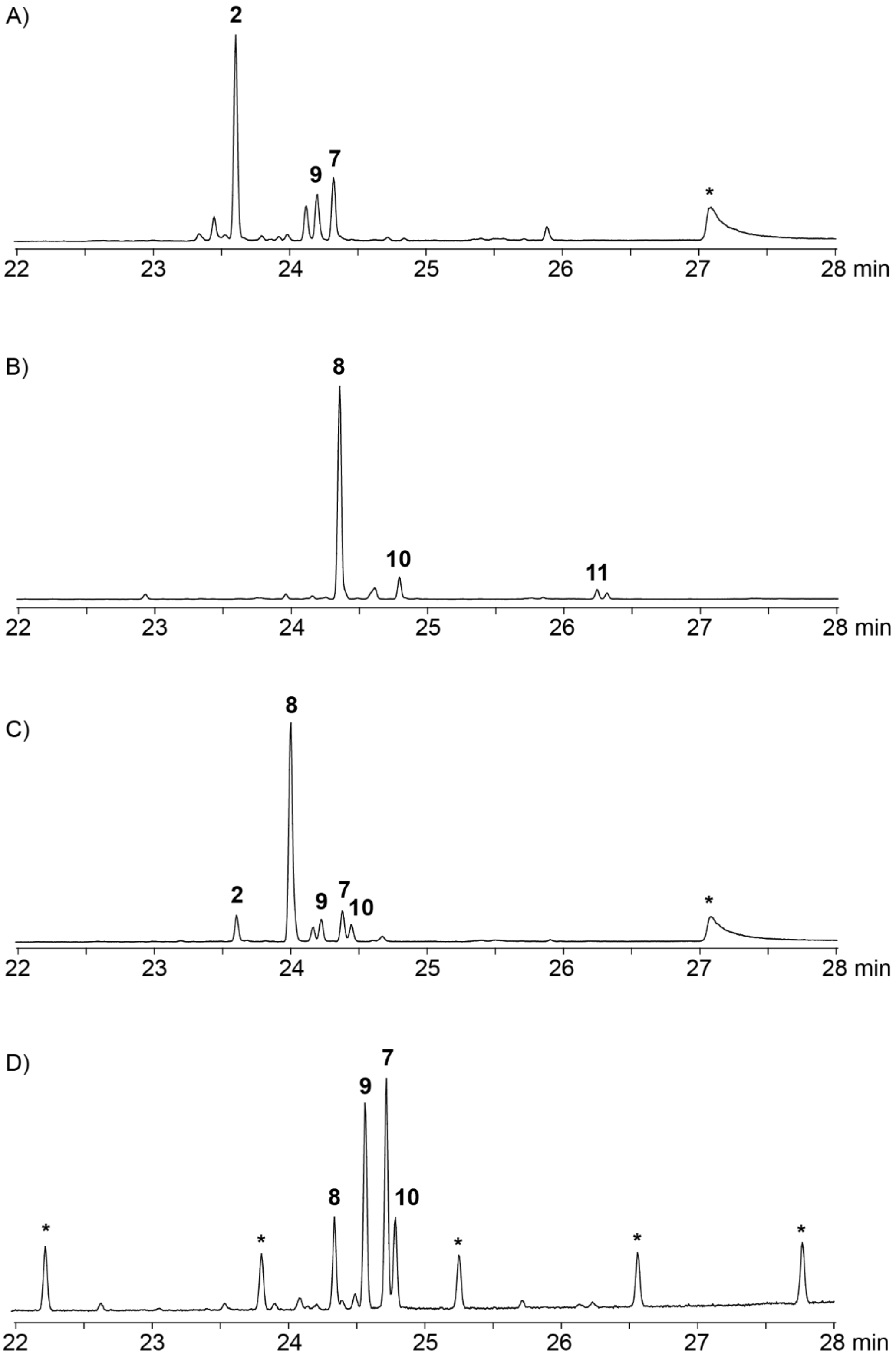

Figure S81. Product mixture formed from iso-FPP and IPP with GGPPS and A) CwWS, B) CpCS, C) CotB2, and D) CgDS. Retention times of compounds may vary, because the GC/MS measurements were performed at different times using GC columns of different ages. Asterisks indicate contaminants, unlabelled peaks represent unknown diterpenes.

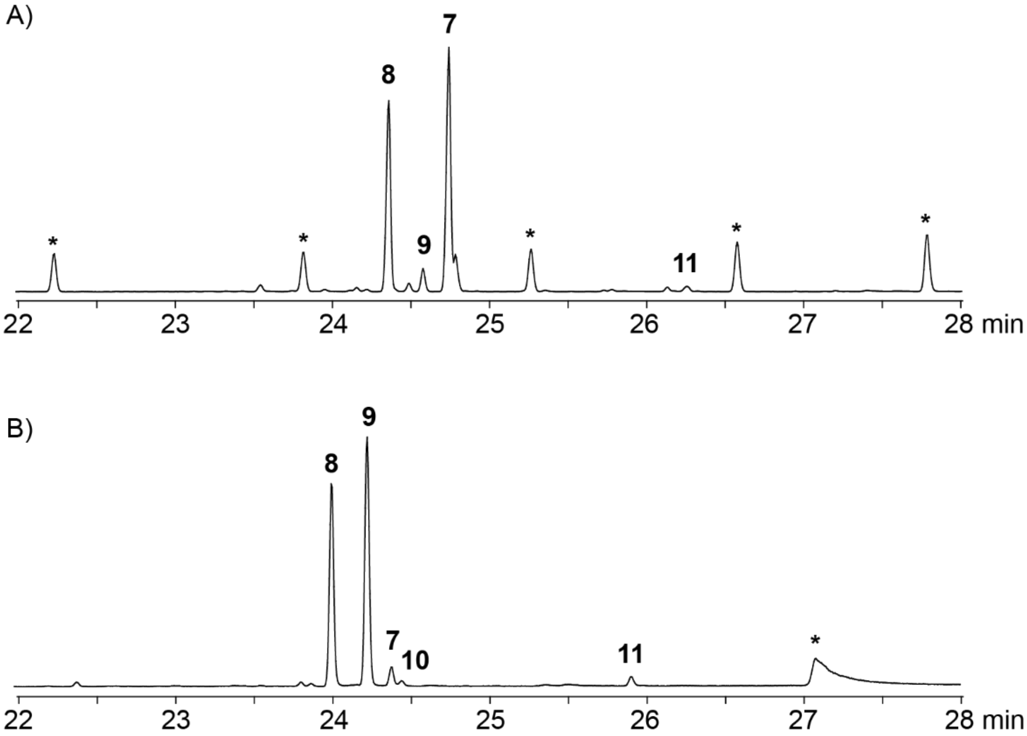

Figure S82. Product mixture formed from iso-FPP and IPP with GGPPS and A) CaCS, and B) CyS. Retention times of compounds may vary, because the GC/MS measurements were performed at different times using GC columns of different ages. Asterisks indicate contaminants, unlabelled peaks represent unknown diterpenes.

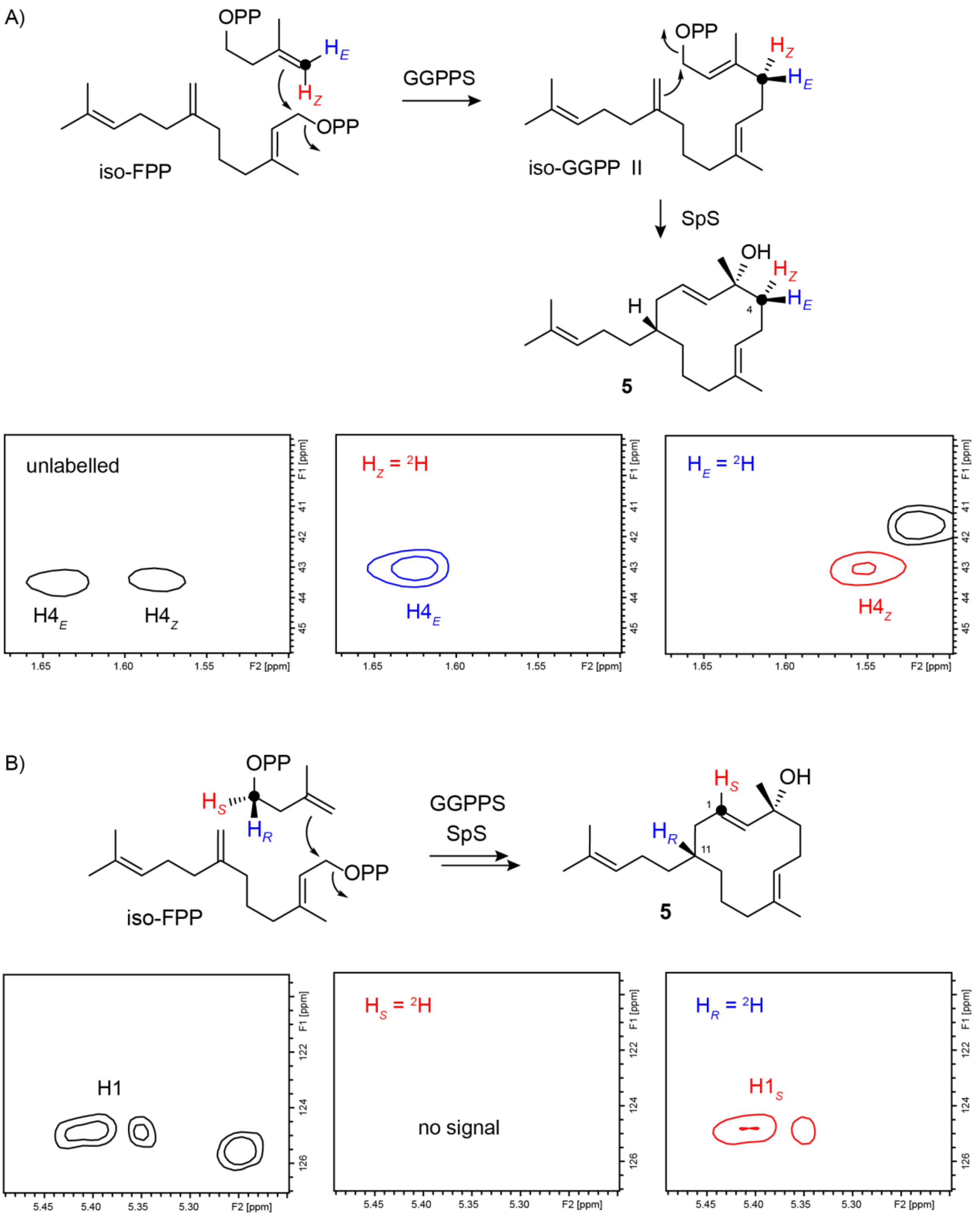

Figure S83. The absolute configuration of **5**. A) Conversion of (*E*)- and (*Z*)-(4-^{13}C,4-^{2}H)IPP with iso-FPP, GGPPS and SpS and HSQC analysis of the products, revealing the absolute configuration for **5** as shown. B) Analogous experiments with (*R*)- and (*S*)-(1-^{13}C,1-^{2}H)IPP show the specific migration of the 1-*pro-R* hydrogen to C11, while the 1-*pro-S* hydrogen remains at C1.

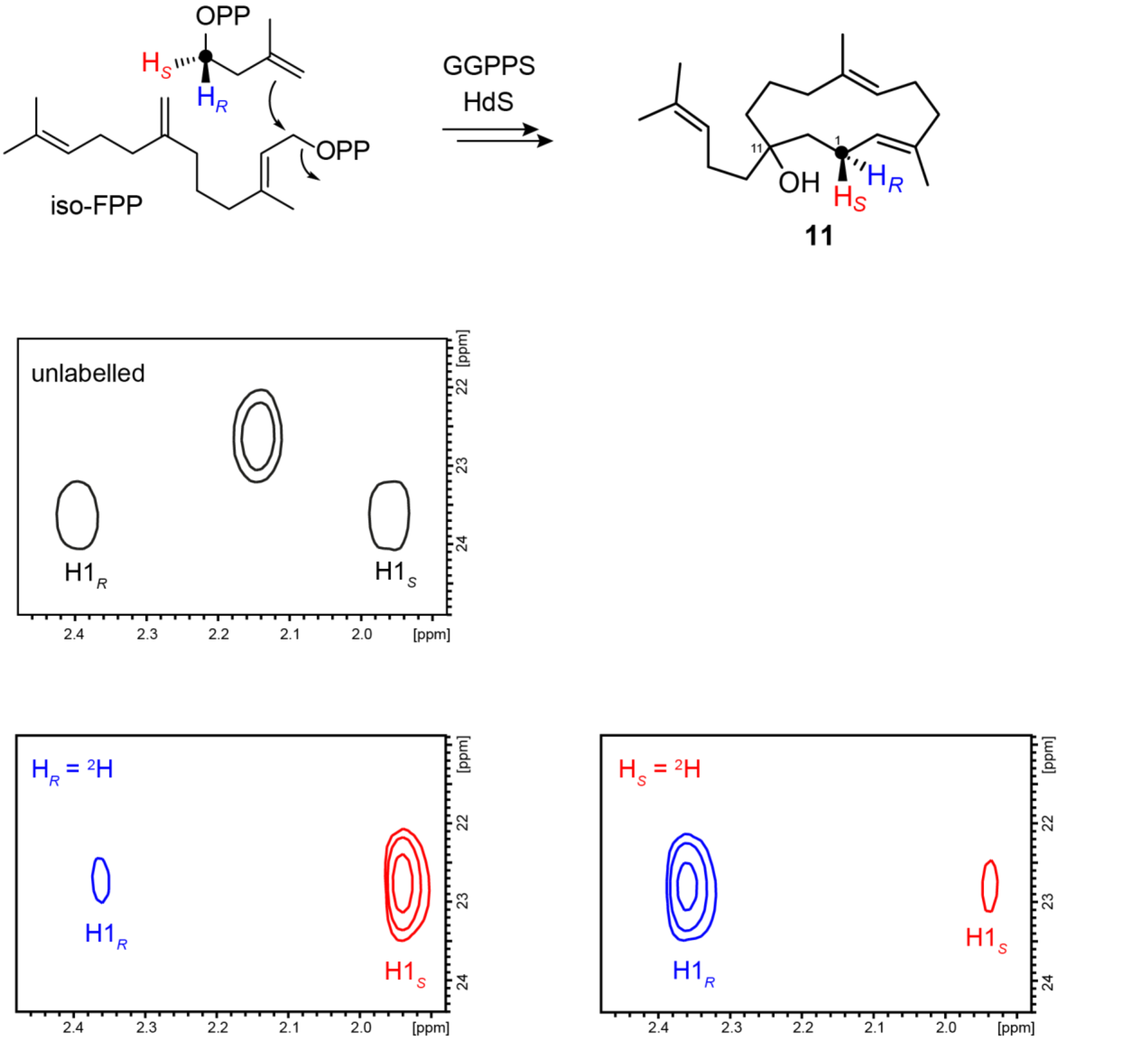

Figure S84. The absolute configuration of **11** obtained with HdS. Peak integration shows an enantiomeric purity of 90% *ee*.

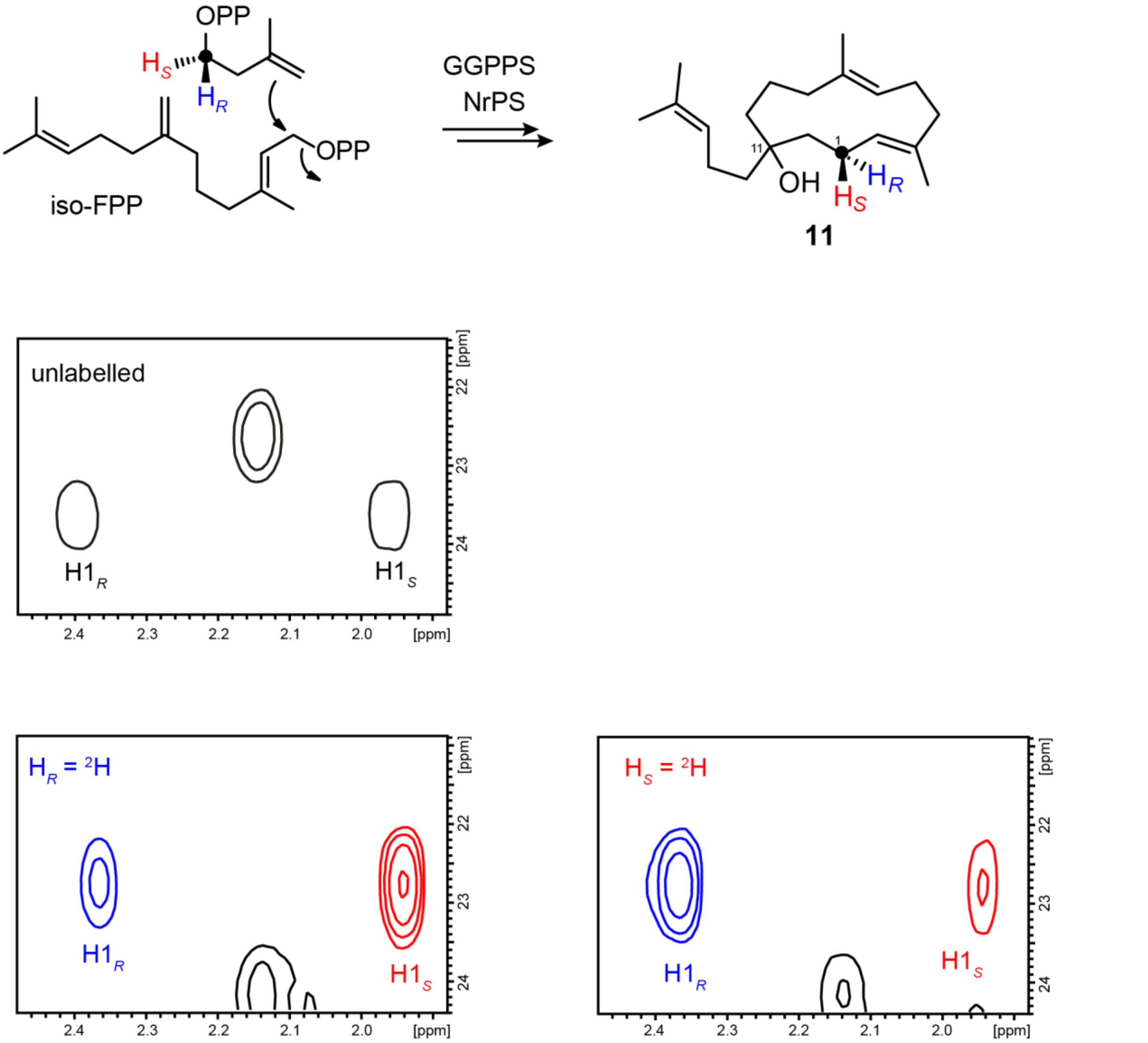

Figure S85. The absolute configuration of **11** obtained with NrPS. Peak integration shows an enantiomeric purity of 70% *ee*.

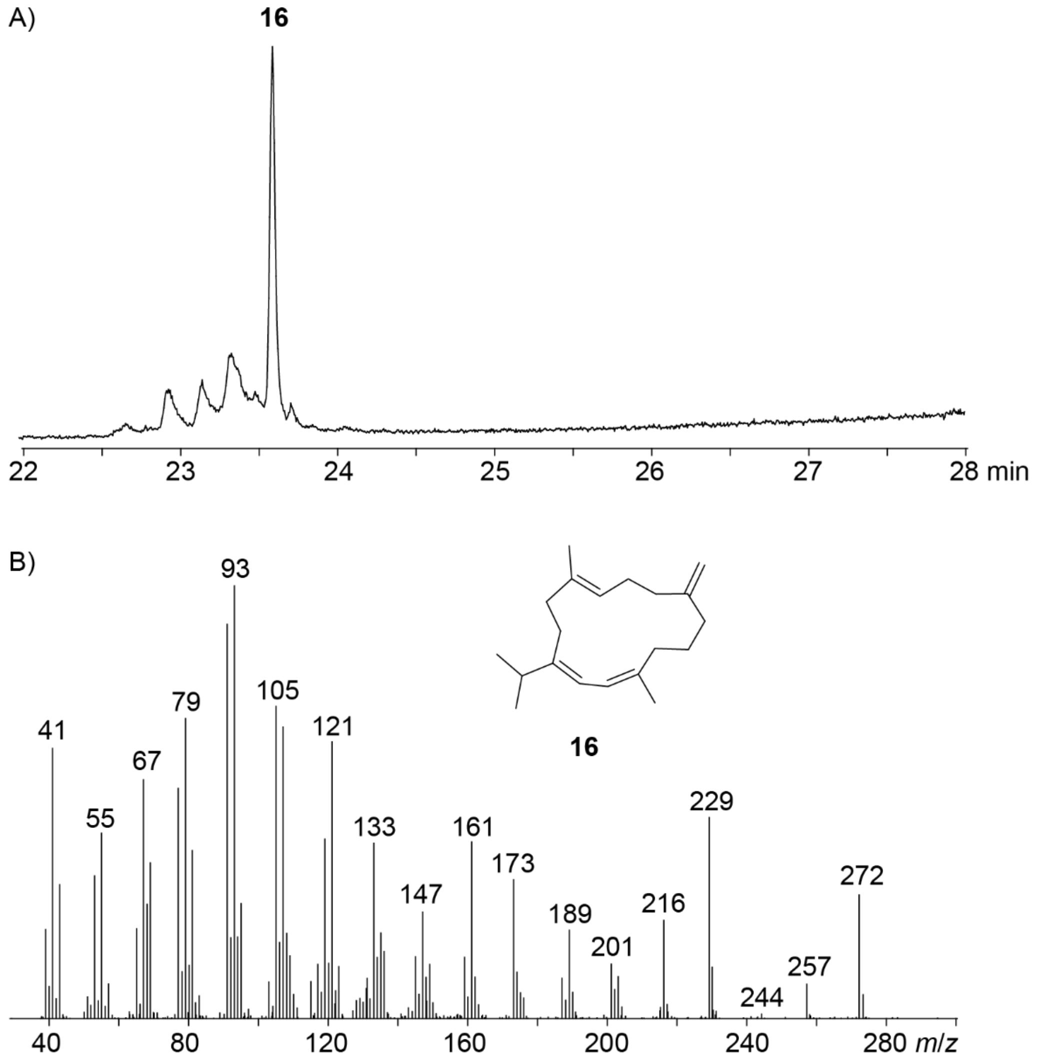

Figure S86. Product mixture formed from iso-GGPP I with PcS. A) Total ion chromatogram of the crude extract from the enzyme incubation, B) EI mass spectrum of **16**.

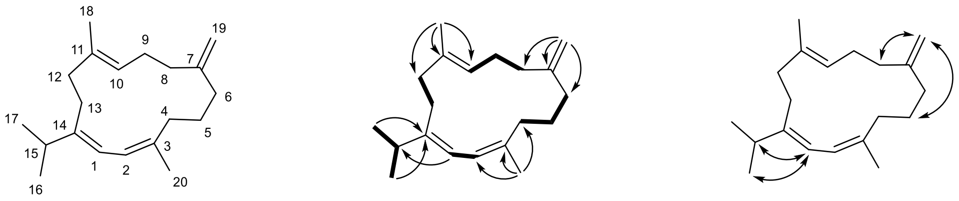

16

Figure S87. Structure elucidation of iso-β-pinacene (**16**). Bold: $^{1}H,^{1}H$-COSY, single-headed arrows: key HMBC, and double-headed arrows: key NOESY correlations.

Table S11. NMR data of iso-β-pinacene (**16**) in C_6D_6 recorded at 298 K.

C[a]	type	^{13}C[b]	^{1}H[b]
1	CH	120.98	6.15 (d, ^{3}J = 10.5)
2	CH	124.73	6.11 (d, ^{3}J = 10.5)
3	C_q	135.15	–
4	CH_2	30.45	2.69 (m)
			1.75 (m)
5	CH_2	24.61	1.72 (m)
			1.56 (m)
6	CH_2	31.57	1.97 (m)
			1.68 (m)
7	C_q	151.24	–
8	CH_2	38.41	2.24 (m)
			1.99 (m)
9	CH_2	28.22	2.12 (m)
			2.05 (m)
10	CH	128.72	4.91 (t, ^{3}J = 6.7)
11	C_q	132.23	–
12	CH_2	38.74	2.11 (m)
			2.05 (m)
13	CH_2	27.73	2.70 (m)
			1.82 (m)
14	C_q	144.16	–
15	CH	32.71	2.20 (hept, ^{3}J = 6.7)
16	CH_3	23.87 (br)	1.04 (br s)
17	CH_3	21.35 (br)	1.11 (br s)
18	CH_3	15.90	1.53 (s)
19	CH_2	107.51	4.80 (br s, H_Z)
			4.75 (m, H_E)
20	CH_3	23.55	1.67 (d, ^{4}J = 1.0)

[a] Carbon numbering as shown in Figure S87. [b] Chemical shifts δ in ppm, multiplicity: s = singlet, d = doublet, t = triplet, hept = heptet, m = multiplet, br = broad, coupling constants J are given in Hertz.

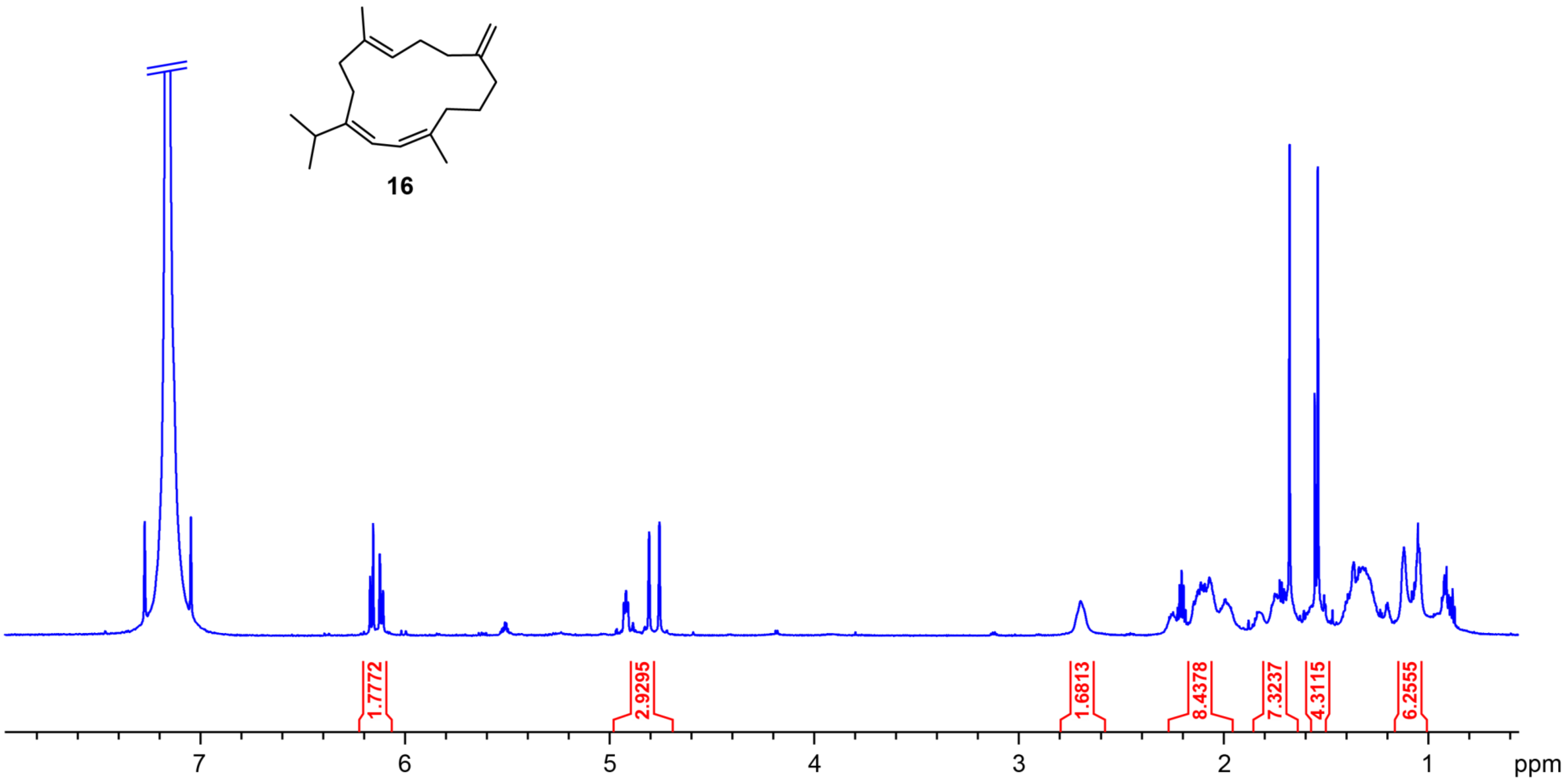

Figure S88. ^{1}H-NMR spectrum (700 MHz, C_6D_6) of **16**.

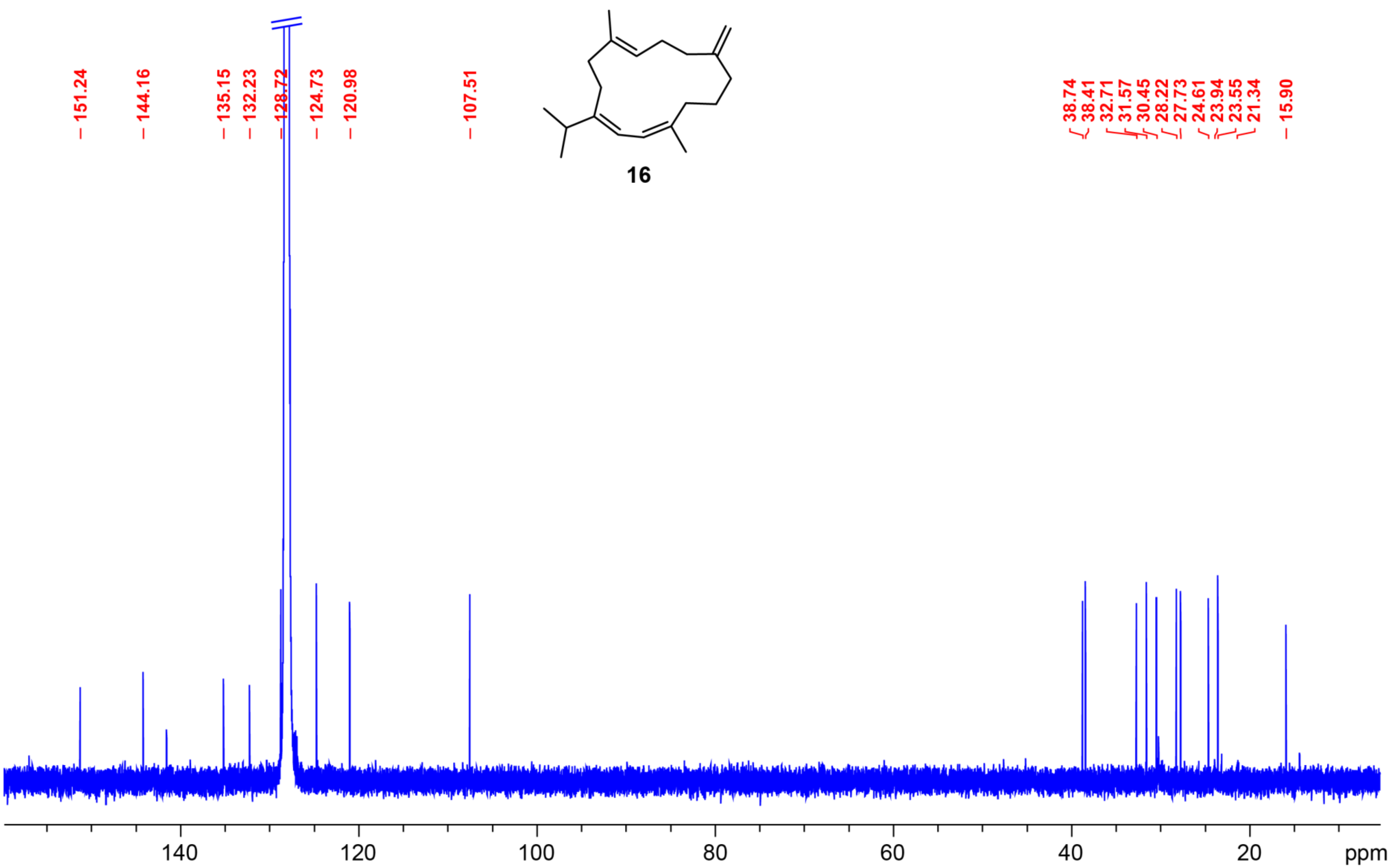

Figure S89. ^{13}C-NMR spectrum (176 MHz, C_6D_6) of **16**.

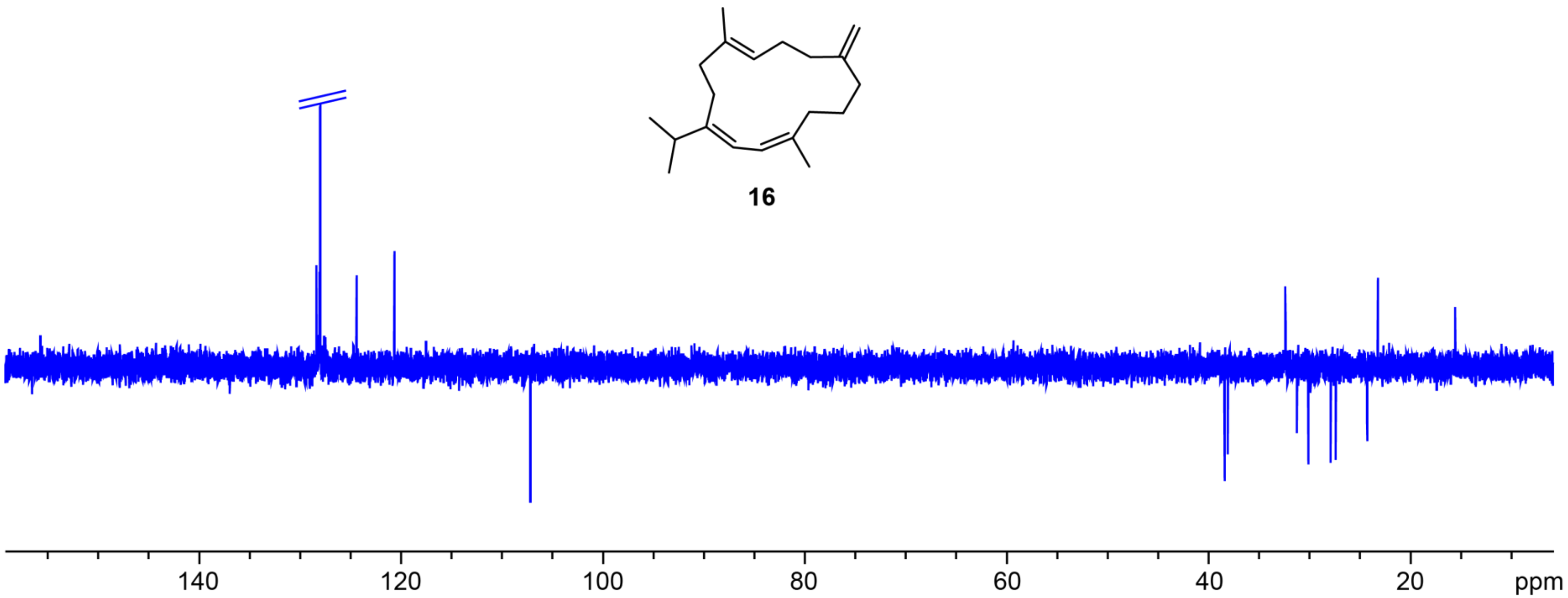

Figure S90. ^{13}C-DEPT135 spectrum (176 MHz, C_6D_6) of **16**.

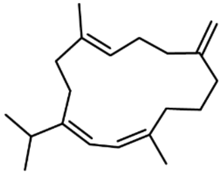

16

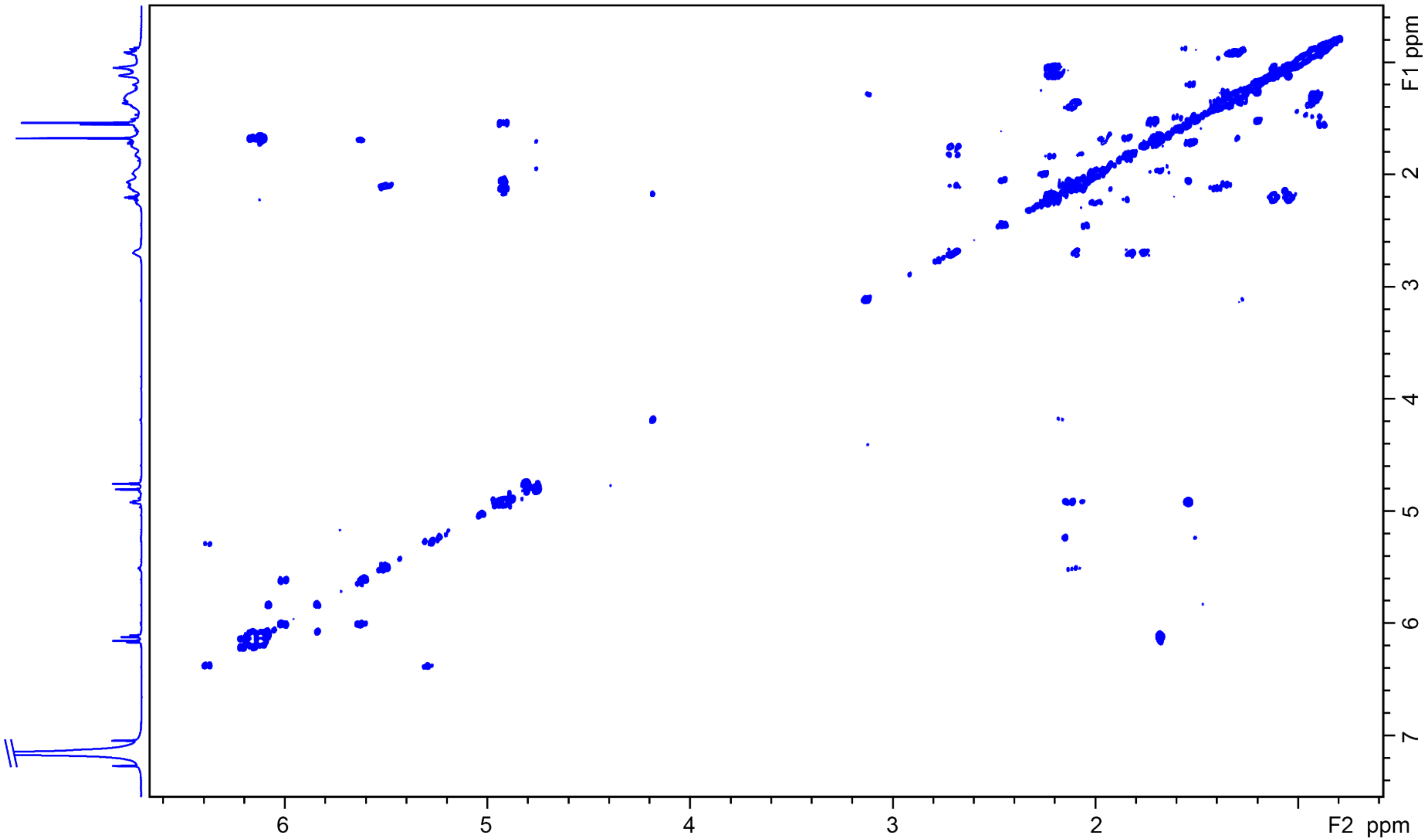

Figure S91. $^{1}H,^{1}H$-COSY spectrum (C_6D_6) of **16**.

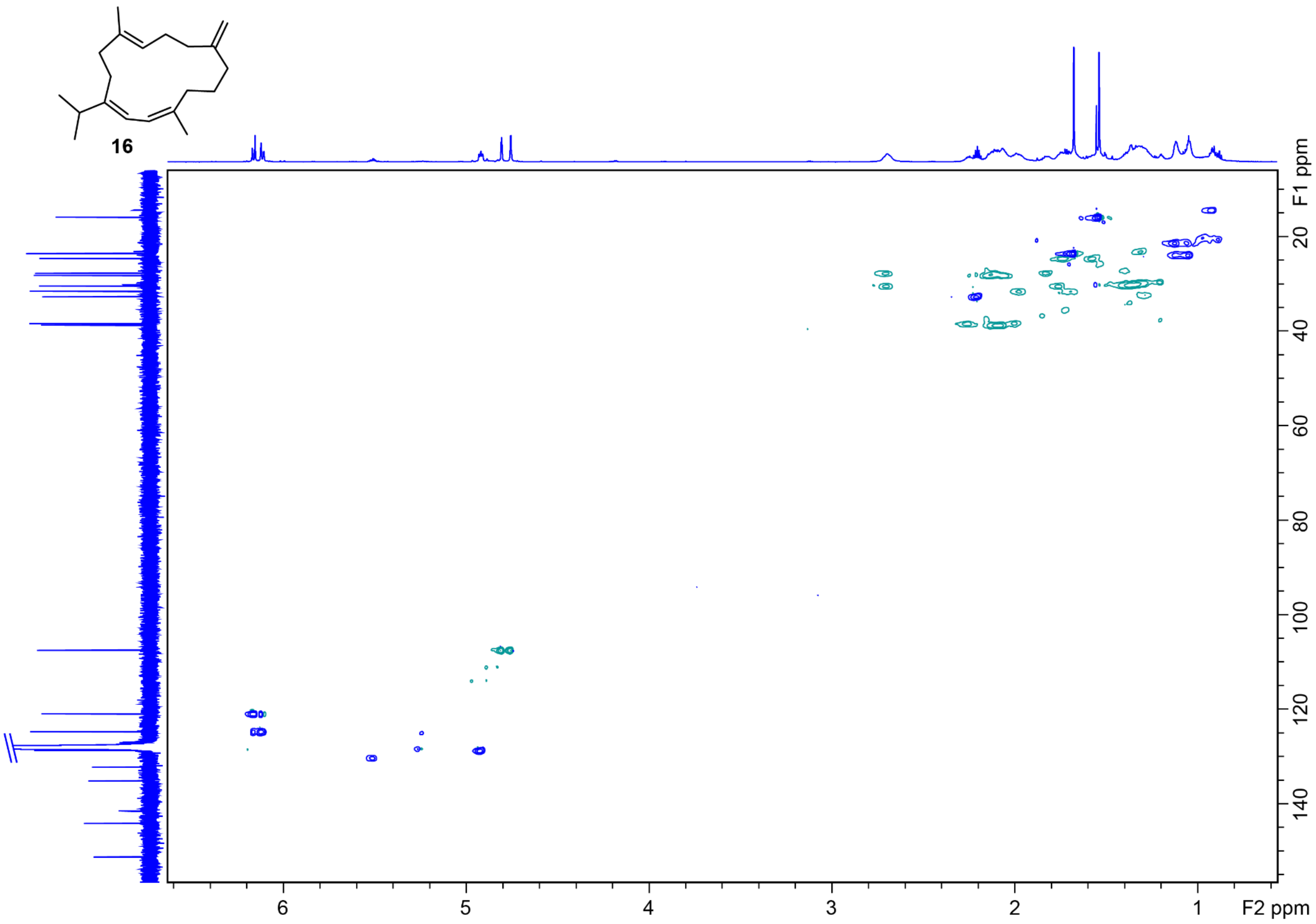

Figure S92. HSQC spectrum (C_6D_6) of **16**.

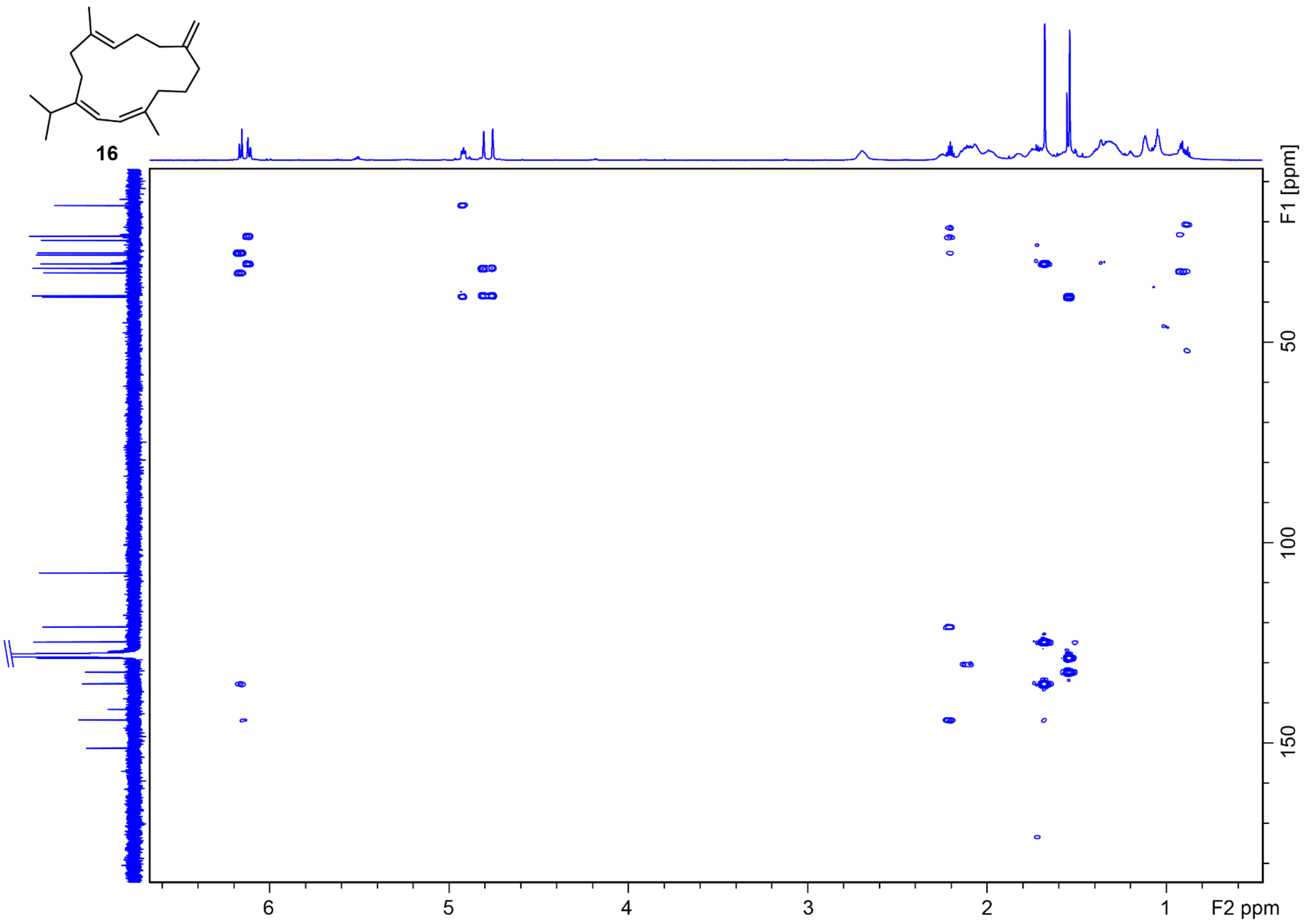

Figure S93. HMBC spectrum (C_6D_6) of **16**.

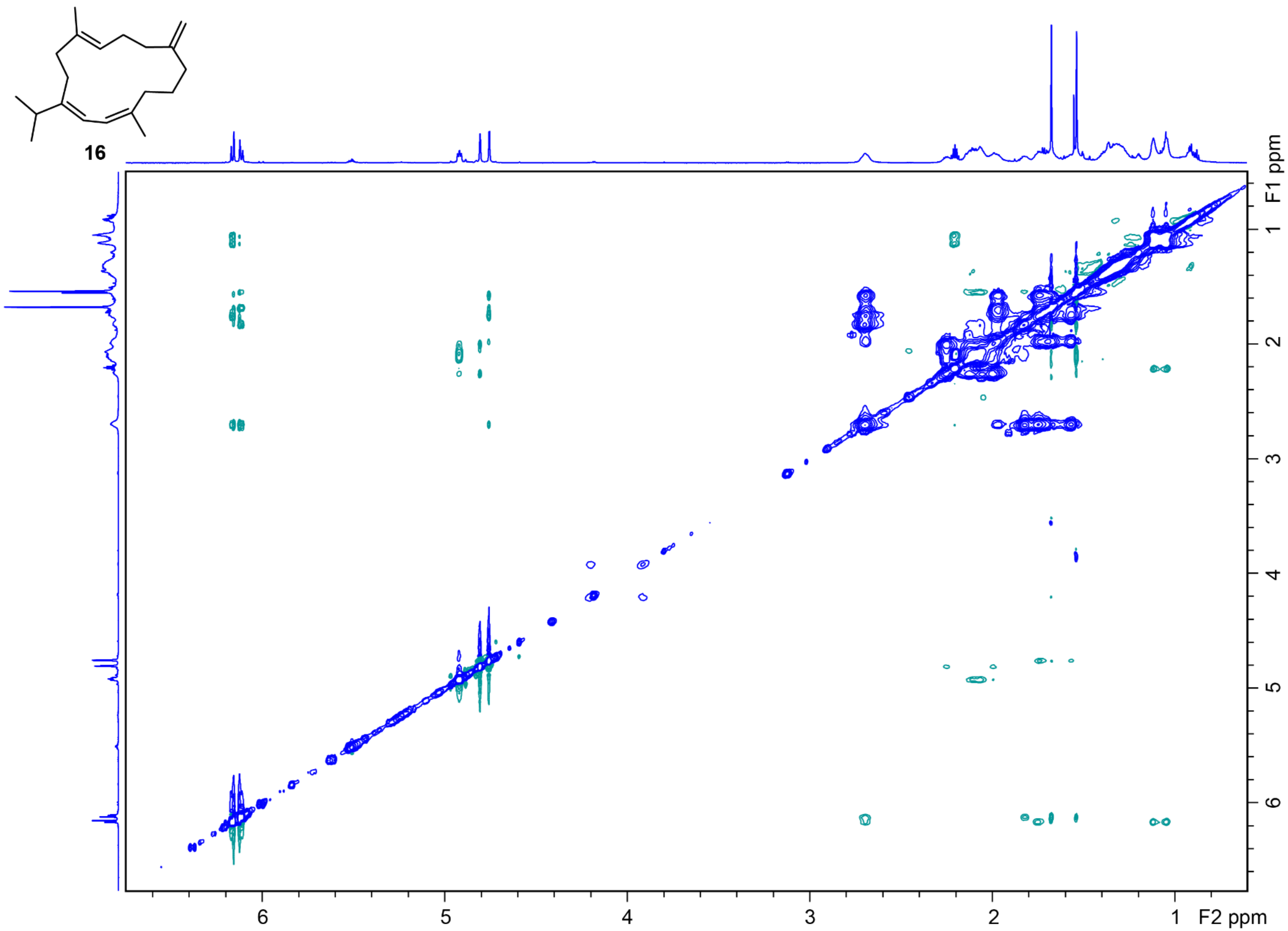

Figure S94. NOESY spectrum (C_6D_6) of **16**.

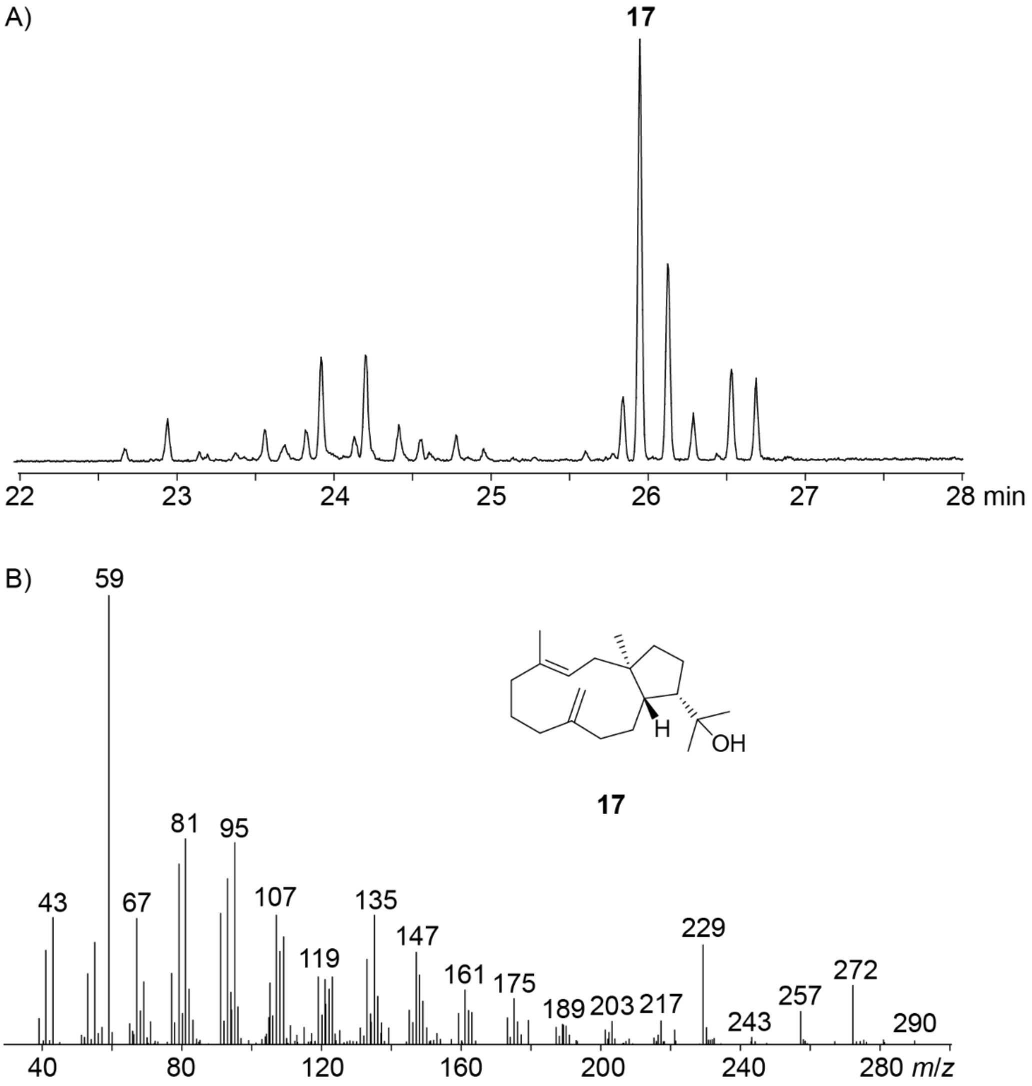

Figure S95. Product mixture formed from iso-GGPP I with HdS. A) Total ion chromatogram of the crude extract from the enzyme incubation, B) EI mass spectrum of **17**.

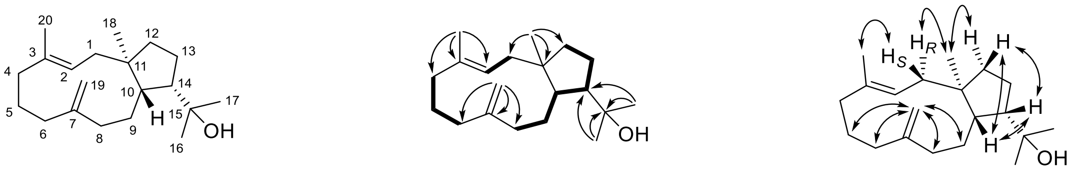

17

Figure S96. Structure elucidation of 18-hydroxydolabella-3,8(17)-diene (**17**). Bold: ^{1}H,^{1}H-COSY, single-headed arrows: key HMBC, and double-headed arrows: key NOESY correlations.

Table S12. NMR data of 18-hydroxydolabella-3,8(17)-diene (**17**) in C_6D_6 recorded at 298 K.

C[a]	type	^{13}C[b]	^{1}H[b]
1	CH_2	44.49	2.15 (dd, ^2J = 13.2, ^3J = 11.5, H_S)
			1.89 (br d, ^2J = 13.2)
2	CH	124.54	5.37 (ddq, ^3J = 11.5, ^3J = 3.6, ^4J = 1.2)
3	C_q	134.82	–
4	CH_2	41.89	2.09 (m)
			1.95 (ddd, ^2J = 13.1, ^3J = 11.6, 3.5)
5	CH_2	24.32	1.77 (m)
			1.60 (m)
6	CH_2	34.69	2.20 (ddd, ^2J = 15.4, ^3J = 8.2, 1.9)
			1.70 (ddd, ^2J = 15.4, ^3J = 10.4, 1.3)
7	C_q	149.03	–
8	CH_2	37.82	2.26 (m)
			2.16 (m)
9	CH_2	24.40	2.08 (m)
			1.47 (m)
10	CH	45.14	1.82 (ddd, ^3J = 9.3, 7.3, 6.1)
11	C_q	45.64	–
12	CH_2	43.66	1.49 (m)
			1.33 (m)
13	CH_2	27.61	1.47 (m, 2H)
14	CH	53.59	2.00 (m)
15	C_q	72.91	–
16	CH_3	32.75	1.10 (s)
17	CH_3	29.27	1.11 (s)
18	CH_3	22.61	1.06 (s)
19	CH_2	109.18	5.03 (m, H_Z)
			4.92 (br s, H_E)
20	CH_3	16.45	1.61 (br s)

[a] Carbon numbering as shown in Figure S96. [b] Chemical shifts δ in ppm, multiplicity: s = singlet, d = doublet, q = quartet, m = multiplet, br = broad, coupling constants J are given in Hertz.

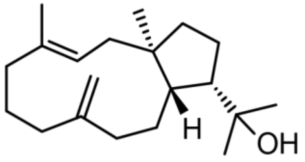

17

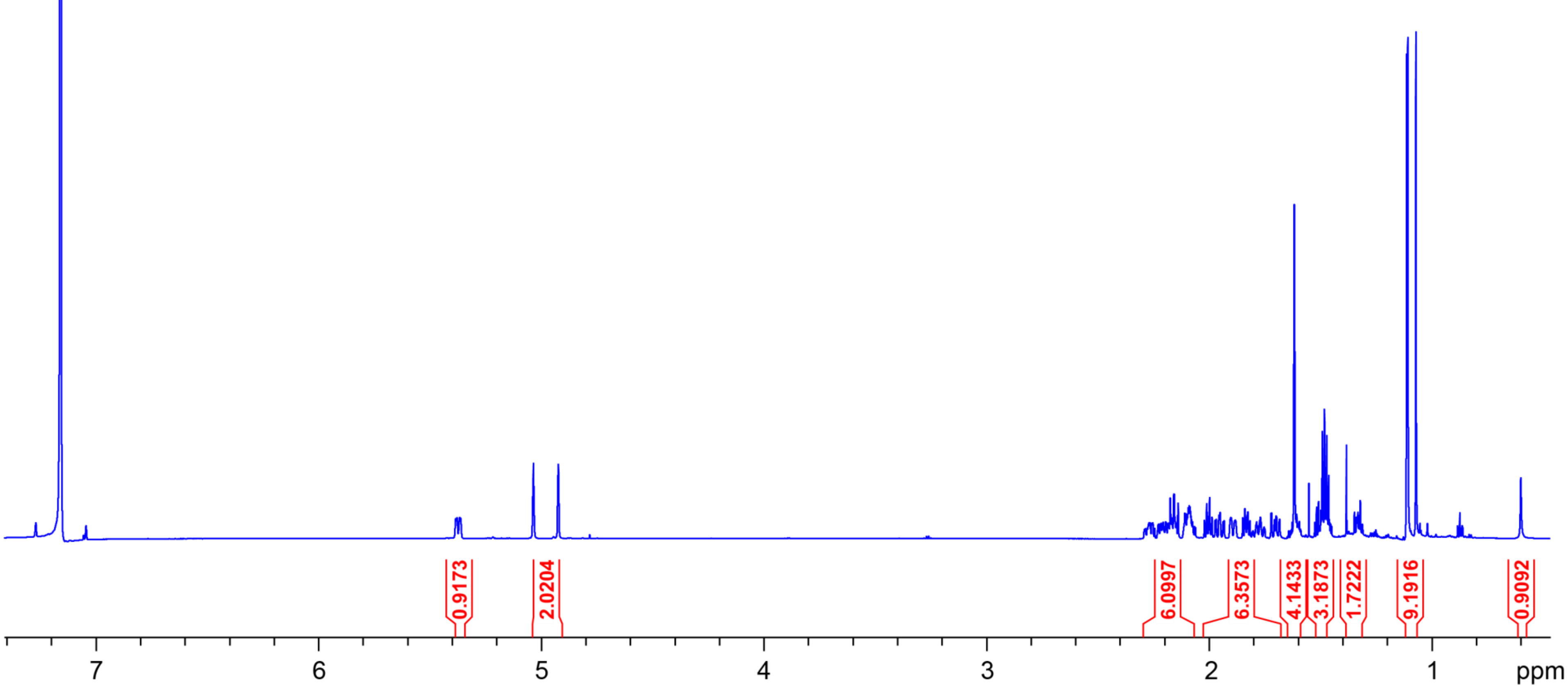

Figure S97. ^{1}H-NMR spectrum (700 MHz, C_6D_6) of **17**.

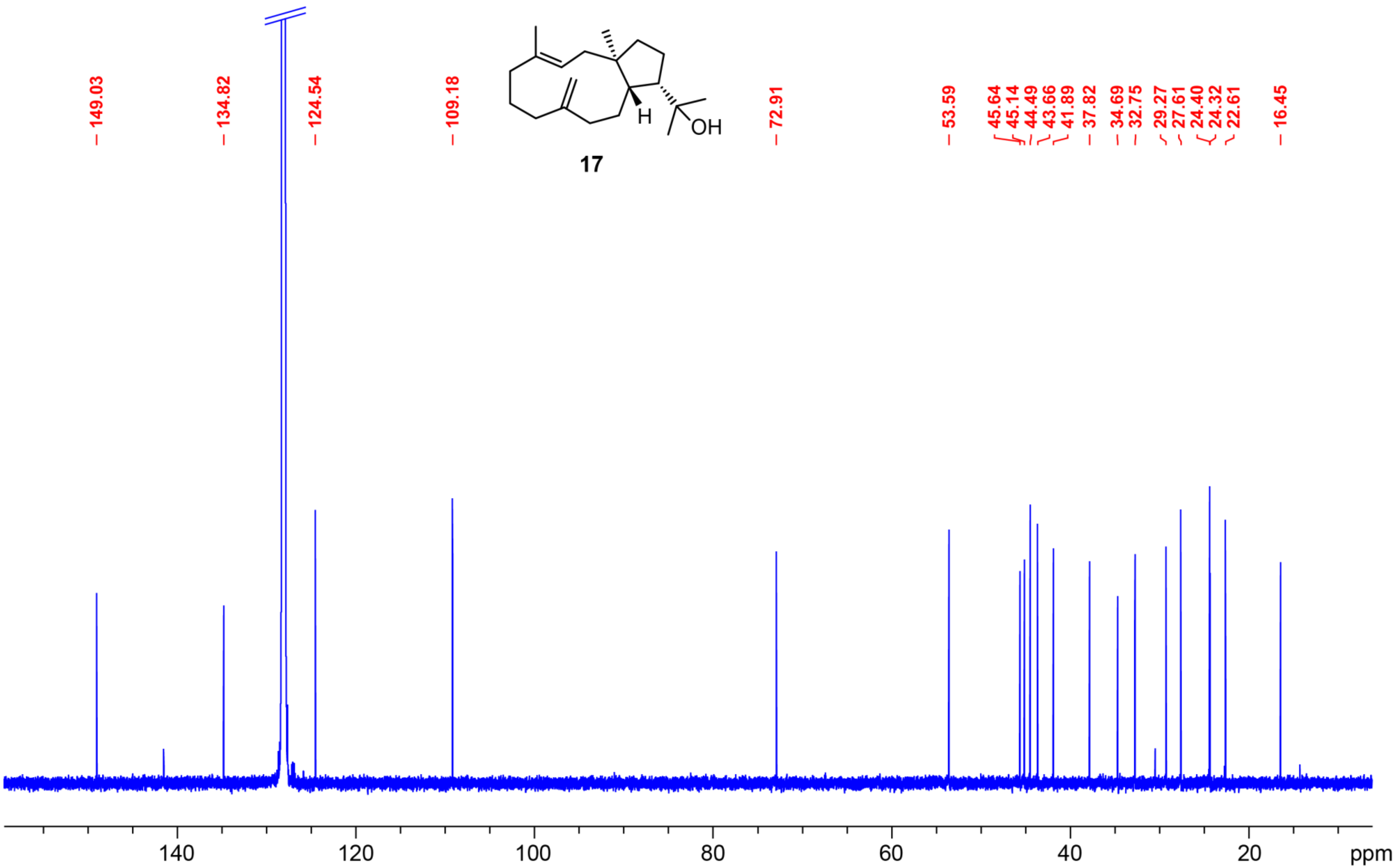

Figure S98. ^{13}C-NMR spectrum (176 MHz, C_6D_6) of **17**.

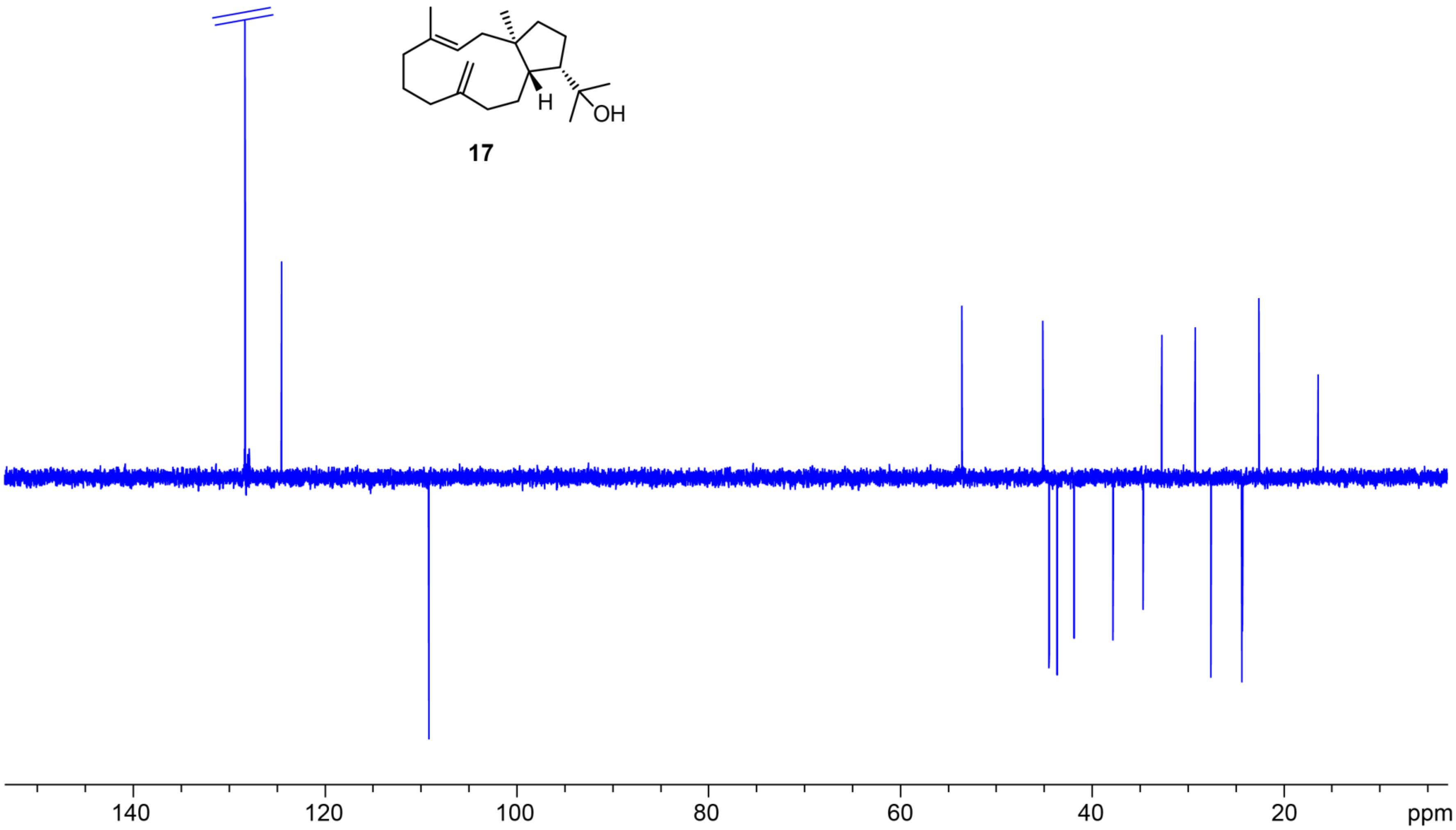

Figure S99. ^{13}C-DEPT135 spectrum (176 MHz, C_6D_6) of **17**.

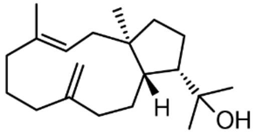

17

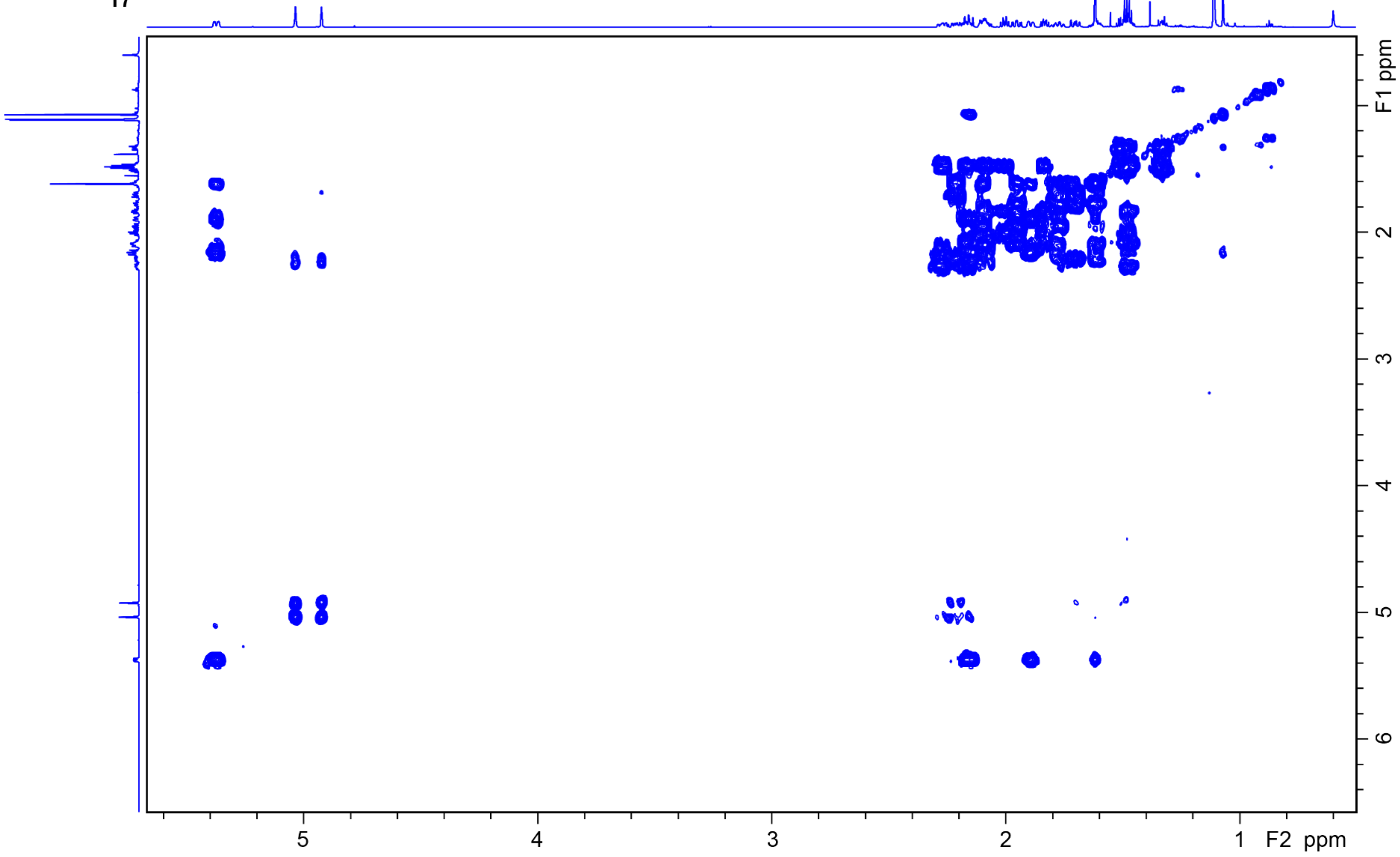

Figure S100. $^{1}H,^{1}H$-COSY spectrum (C_6D_6) of **17**.

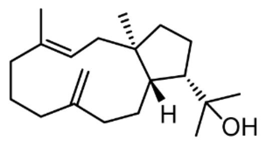

17

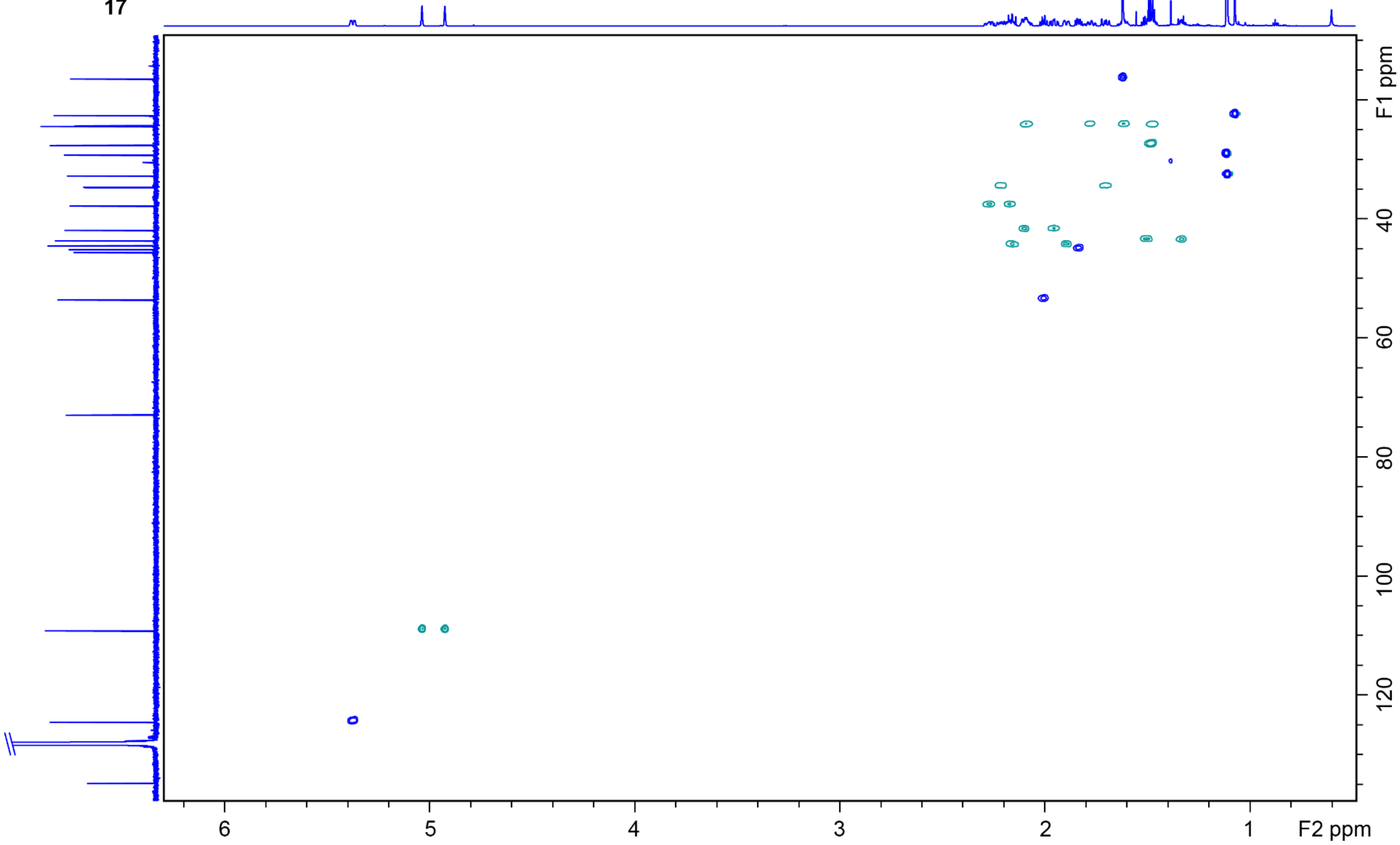

Figure S101. HSQC spectrum (C_6D_6) of **17**.

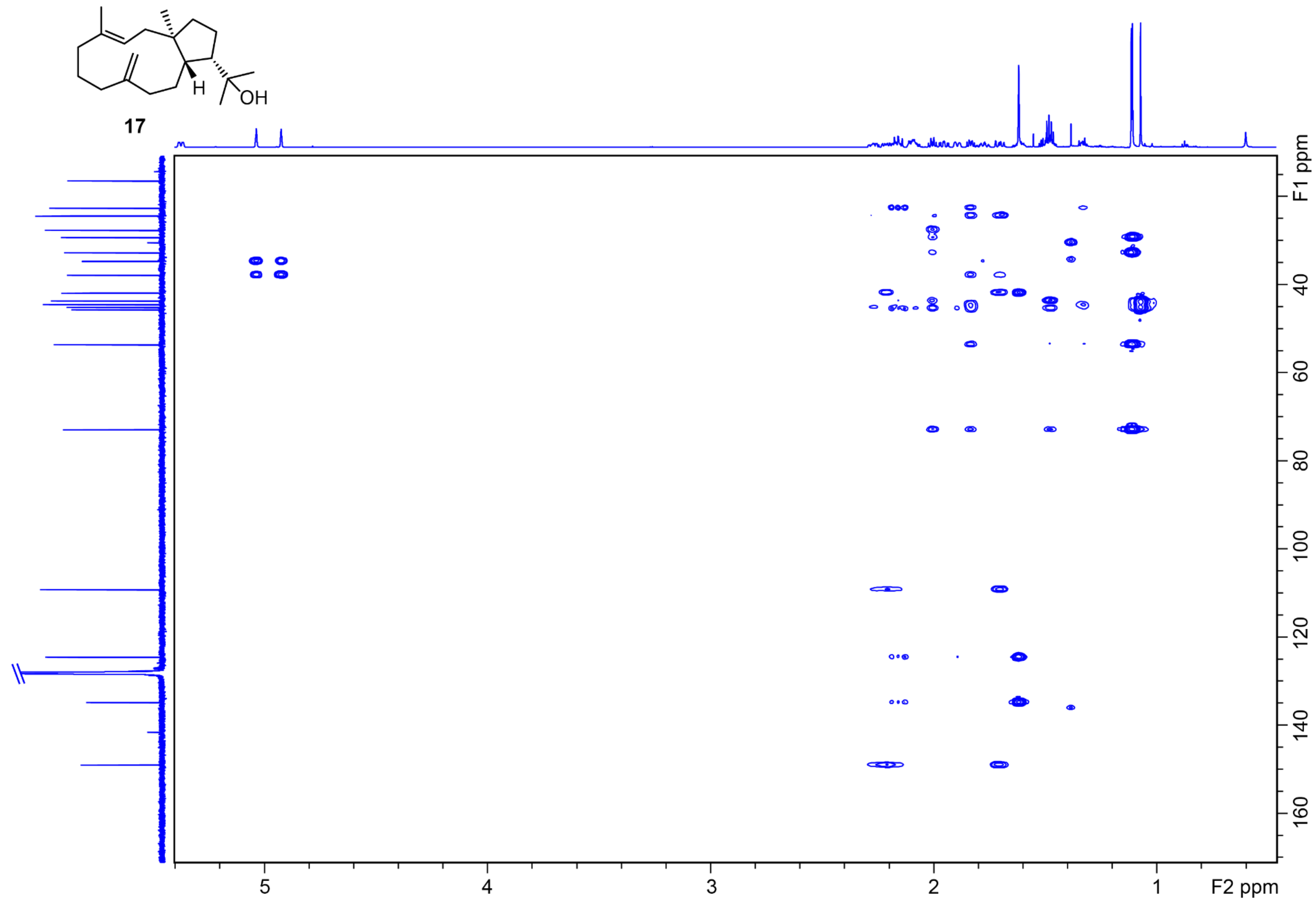

Figure S102. HMBC spectrum (C_6D_6) of **17**.

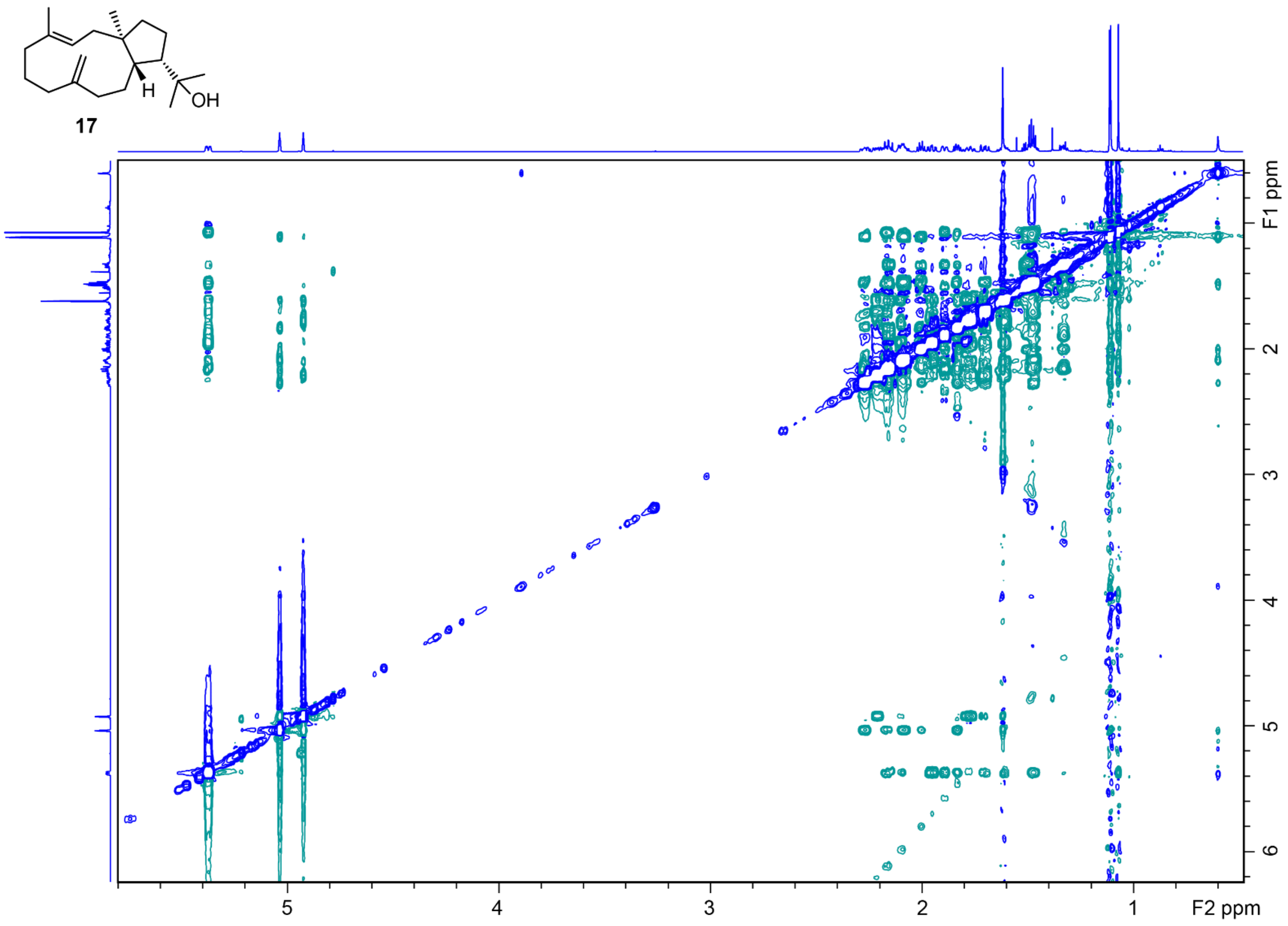

Figure S103. NOESY spectrum (C_6D_6) of **17**.

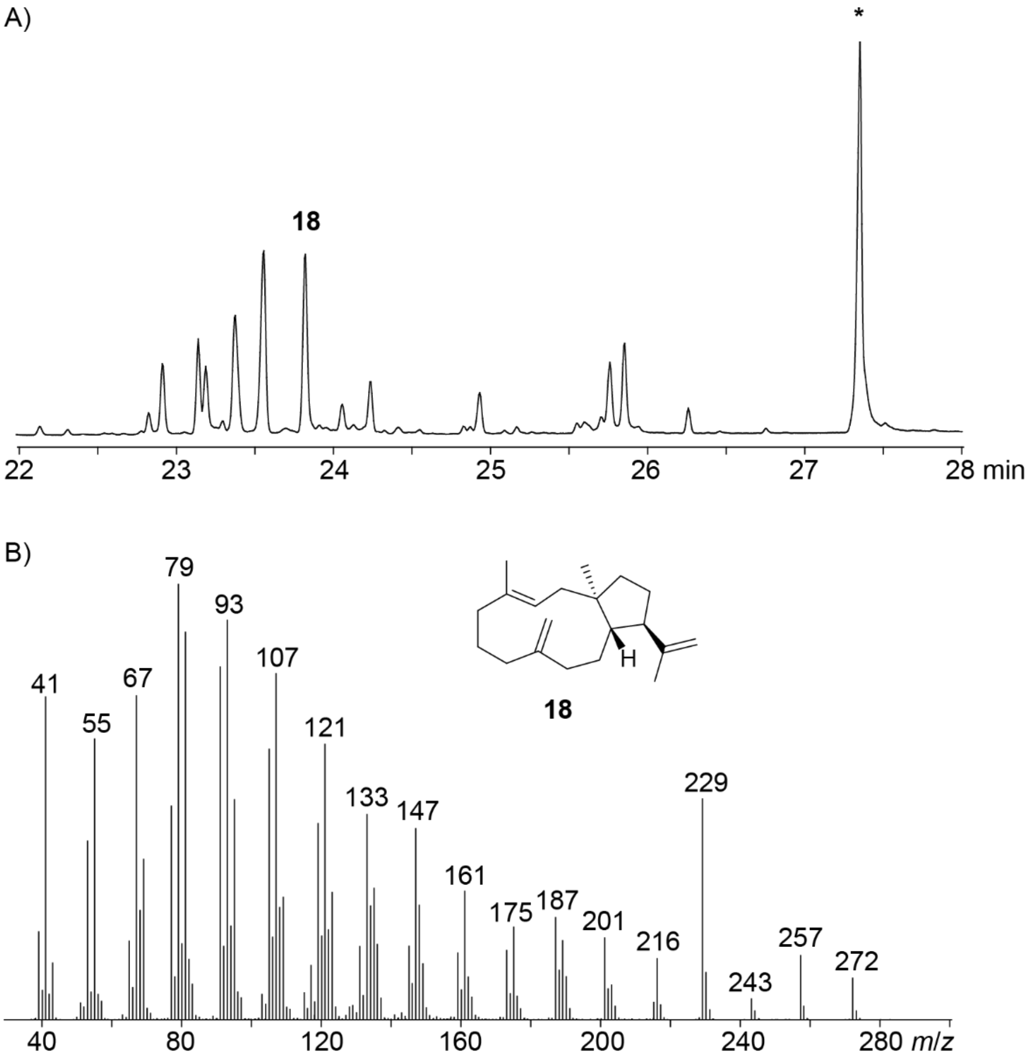

Figure S104. Product mixture formed from iso-GGPP I with NrPS. A) Total ion chromatogram of the crude extract from the enzyme incubation, B) EI mass spectrum of **18**. Asterisks indicate contaminants.

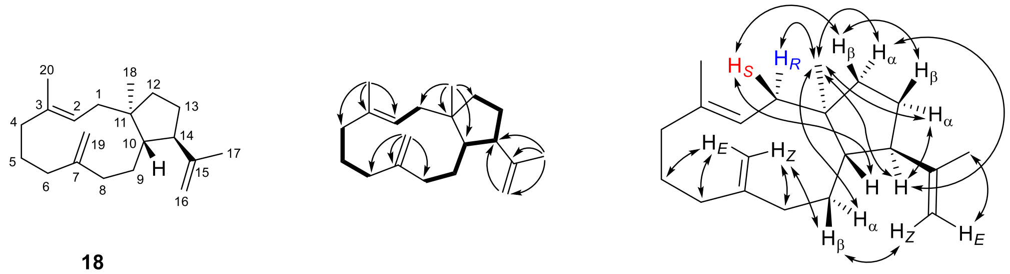

18

Figure S105. Structure elucidation of dolabella-3,8(17),18-triene (**18**). Bold: ^{1}H,^{1}H-COSY, single-headed arrows: key HMBC, and double-headed arrows: key NOESY correlations.

Table S13. NMR data of dolabella-3,8(17),18-triene (**18**) in C_6D_6 recorded at 298 K.

C[a]	type	^{13}C[b]	^{1}H[b]
1	CH_2	43.17	2.19 (m, H_S)
			1.76 (m, H_R)
2	CH	123.86	5.31 (dm, ^{3}J = 11.8)
3	C_q	135.49	–
4	CH_2	41.61	2.03 (m)
			1.97 (m)
5	CH_2	25.08	1.73 (m)
			1.61 (m)
6	CH_2	35.84	2.18 (m)
			1.62 (m)
7	C_q	148.81	–
8	CH_2	35.45	2.09 (m, 2H)
9	CH_2	27.35	1.48 (m, 2H)
10	CH	44.26	1.42 (m)
11	C_q	45.10	–
12	CH_2	44.02	1.50 (m, H_β)
			1.35 (m, H_α)
13	CH_2	29.76	1.56 (m, H_α)
			1.50 (m, H_β)
14	CH	57.76	2.11 (m)
15	C_q	148.08	–
16	CH_2	111.80	4.88 (m, H_Z)
			4.83 (m, H_E)
17	CH_3	18.77	1.71 (dd, ^{4}J = 1.3, 0.7)
18	CH_3	22.82	0.98 (s)
19	CH_2	108.76	4.87 (m, H_E)
			4.83 (m, H_Z)
20	CH_3	16.65	1.60 (br s)

[a] Carbon numbering as shown in Figure S105. [b] Chemical shifts δ in ppm, multiplicity: s = singlet, d = doublet, t = triplet, hept = heptet, m = multiplet, br = broad, coupling constants J are given in Hertz.

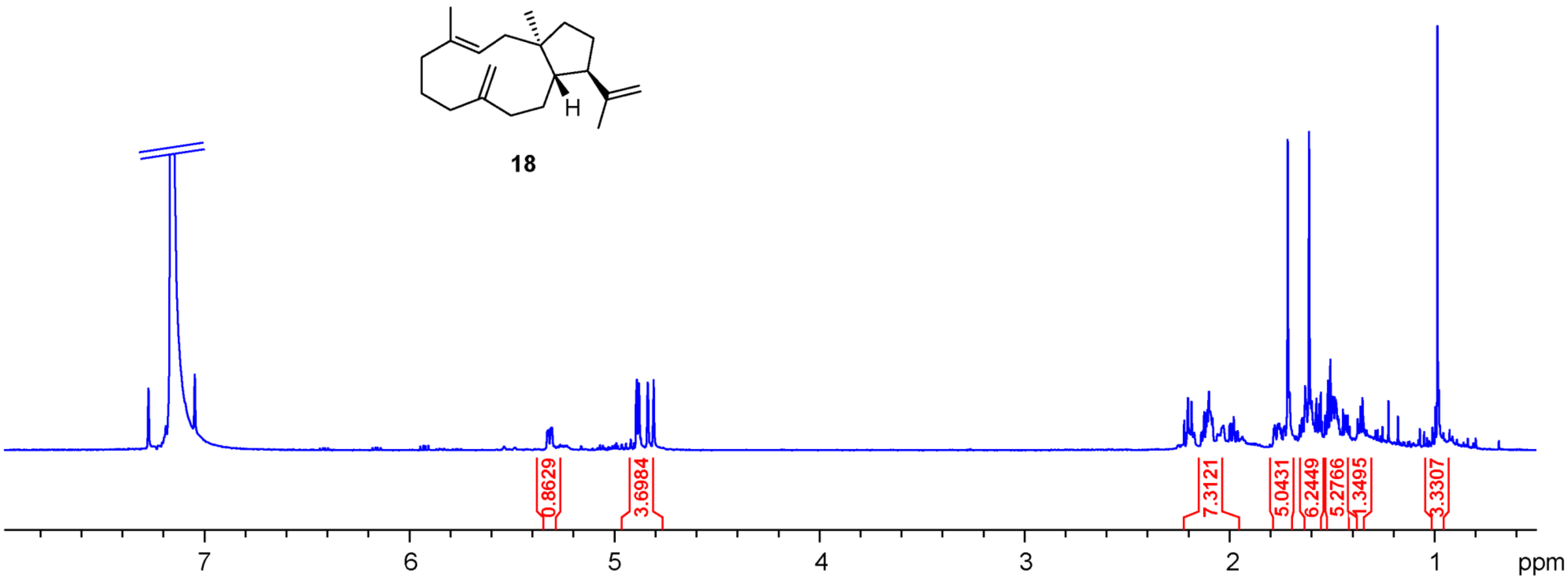

Figure S106. ^{1}H-NMR spectrum (700 MHz, C_6D_6) of **18**.

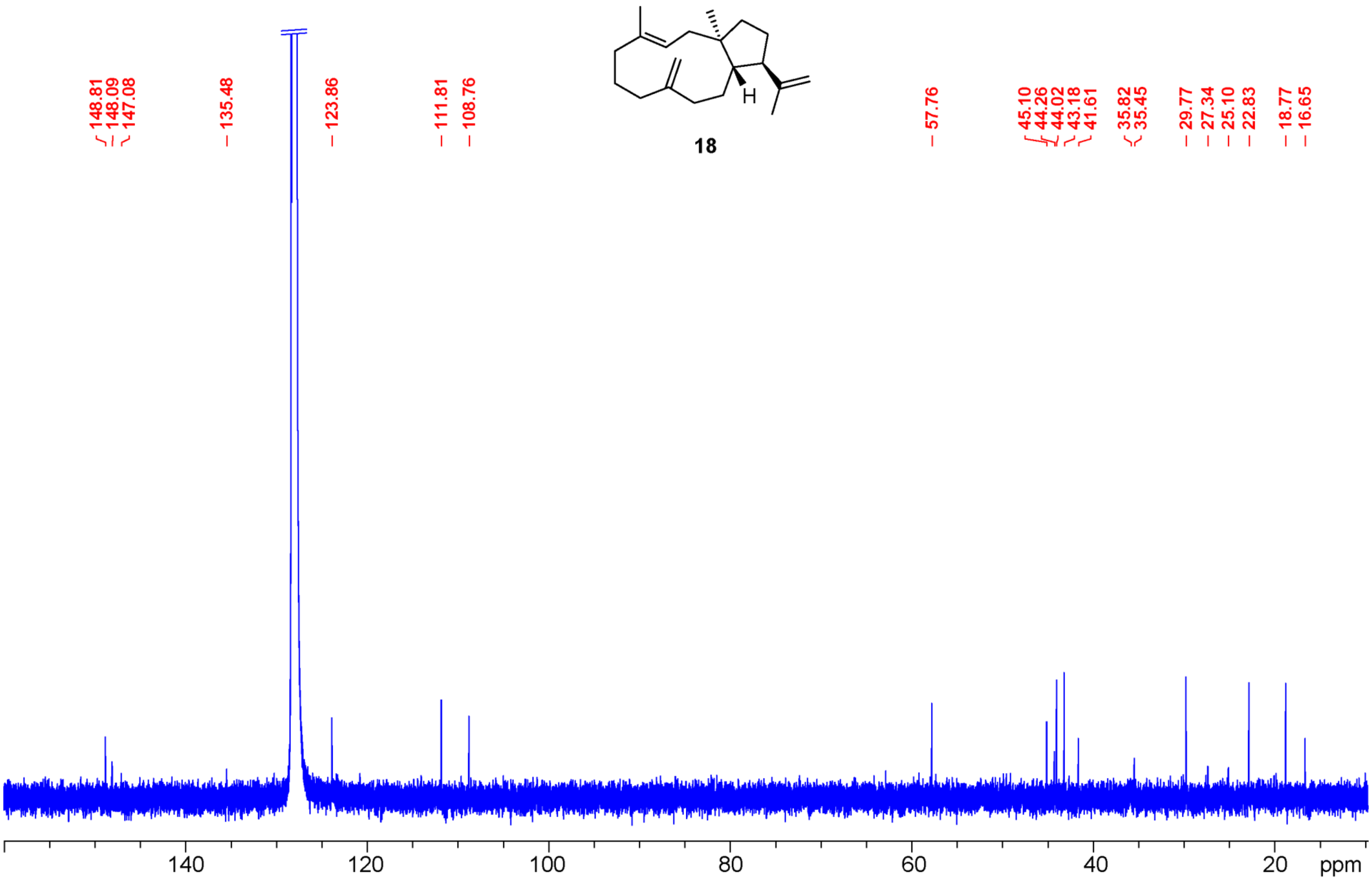

Figure S107. ^{13}C-NMR spectrum (176 MHz, C_6D_6) of **18**.

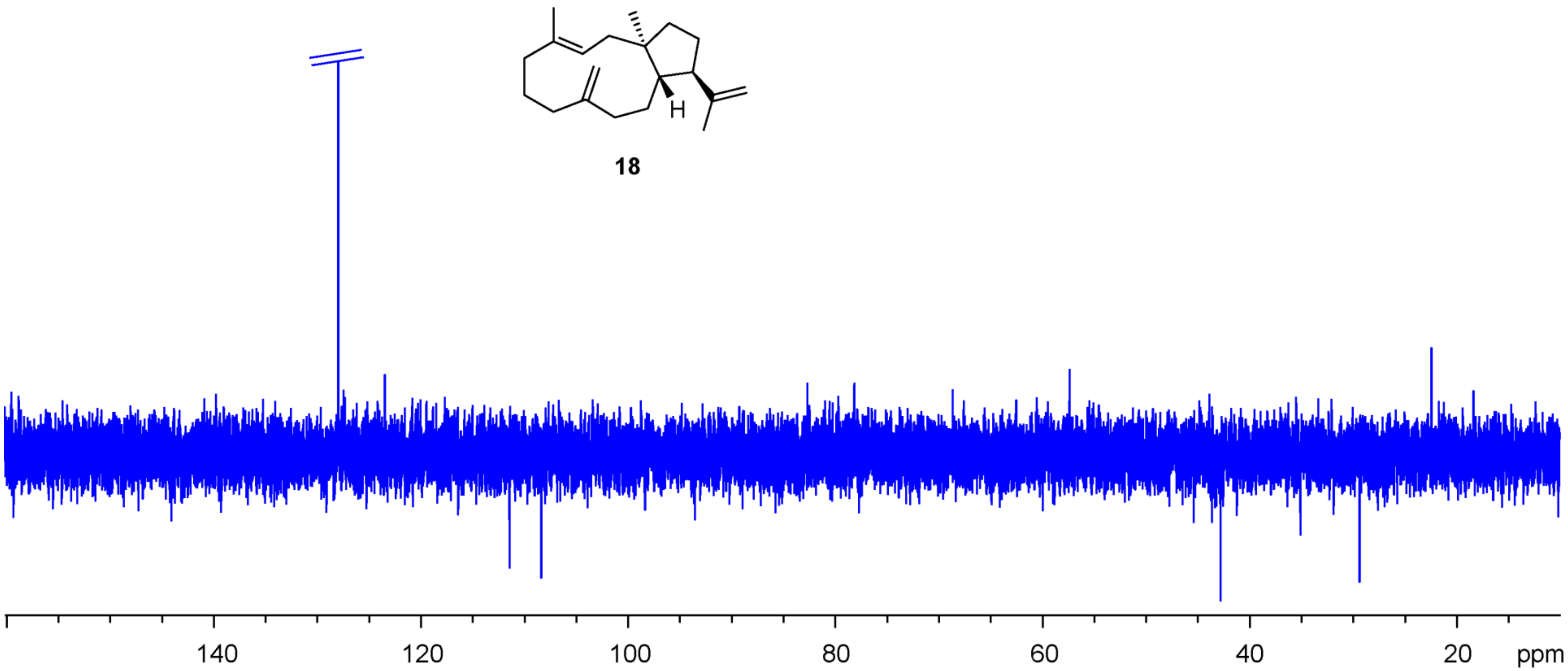

Figure S108. ^{13}C-DEPT135 spectrum (176 MHz, C_6D_6) of **18**.

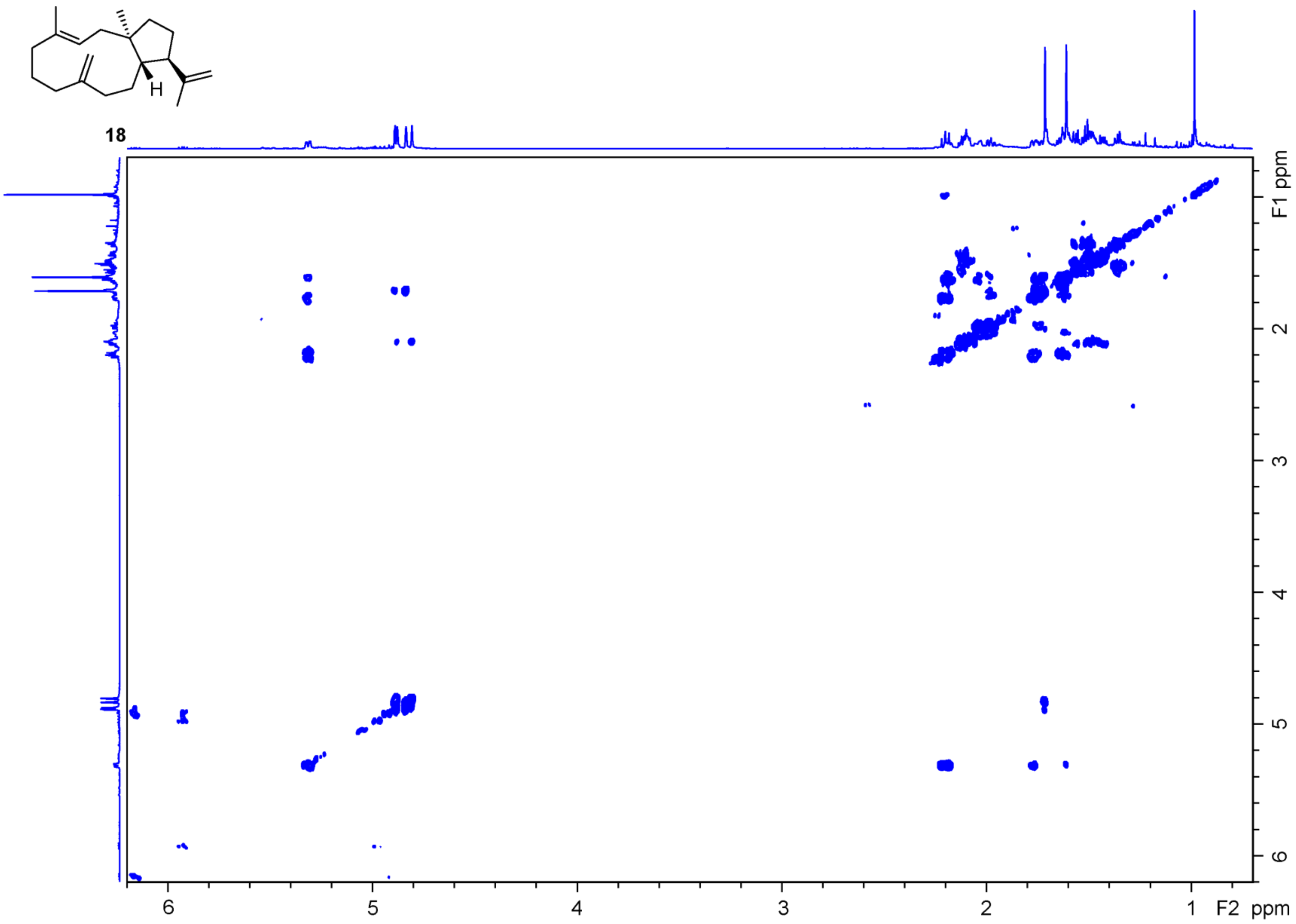

Figure S109. $^{1}H,^{1}H$-COSY spectrum (C_6D_6) of **18**.

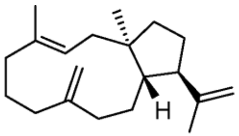

18

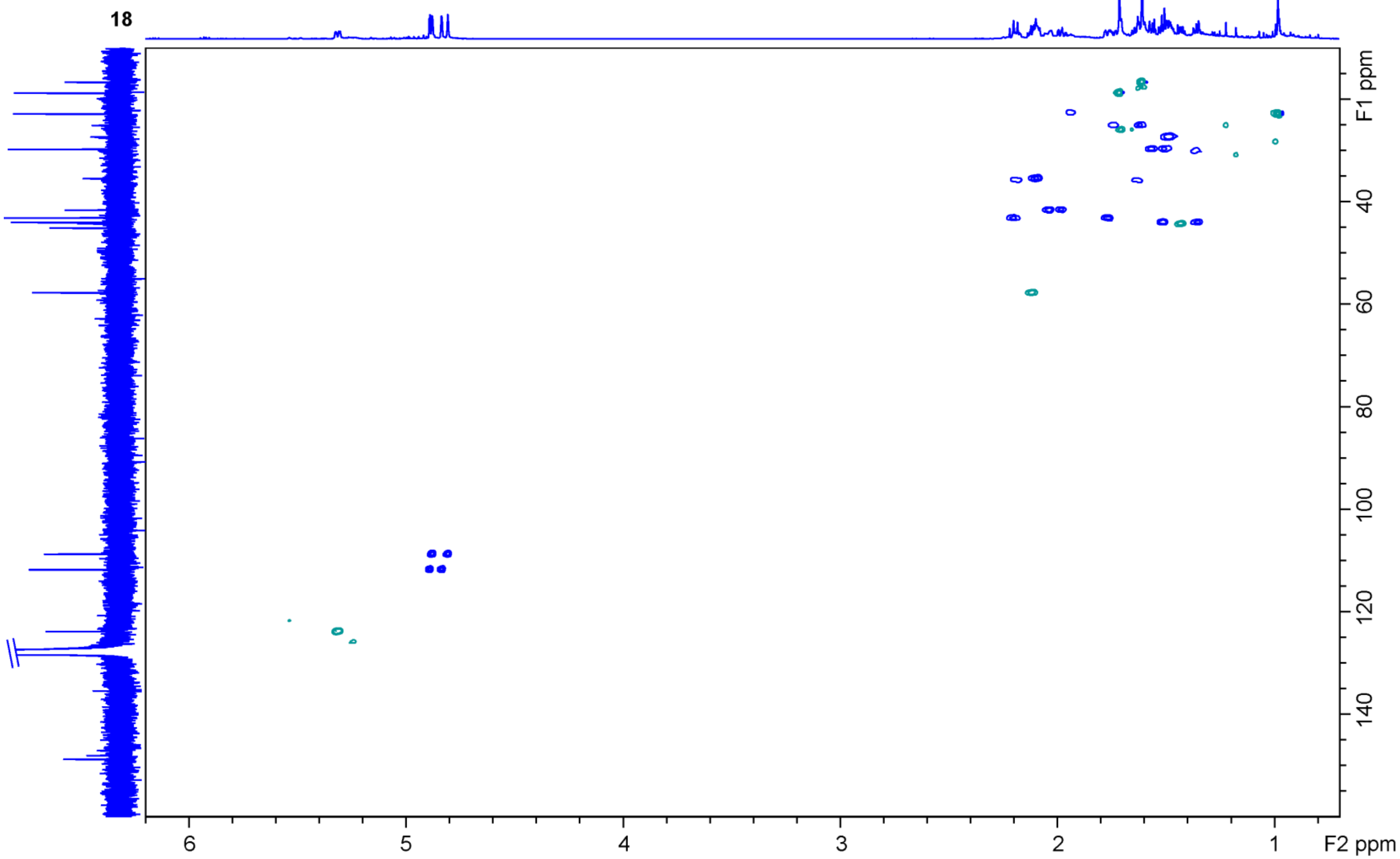

Figure S110. HSQC spectrum (C_6D_6) of **18**.

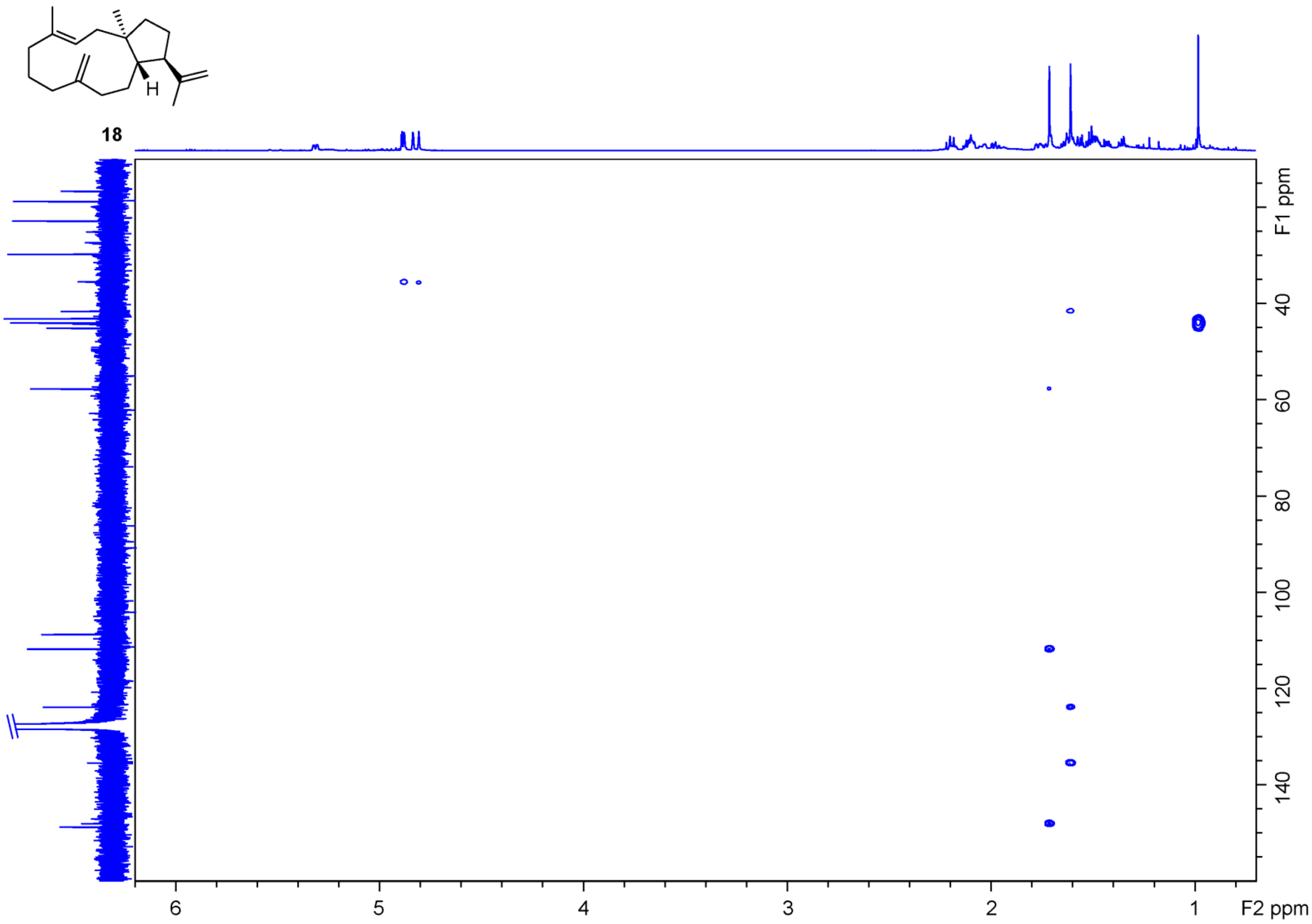

Figure S111. HMBC spectrum (C_6D_6) of **18**.

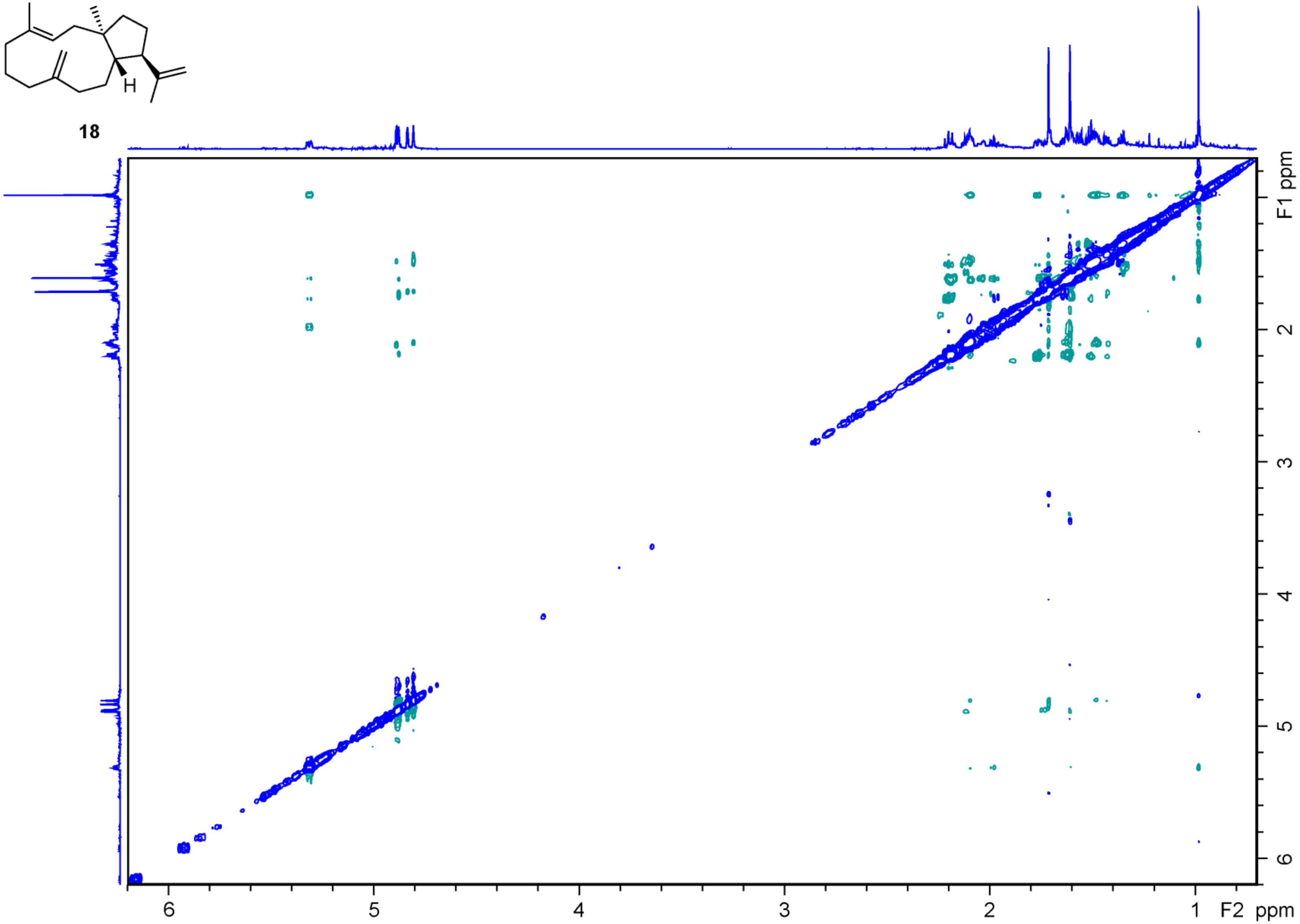

Figure S112. NOESY spectrum (C_6D_6) of **18**.

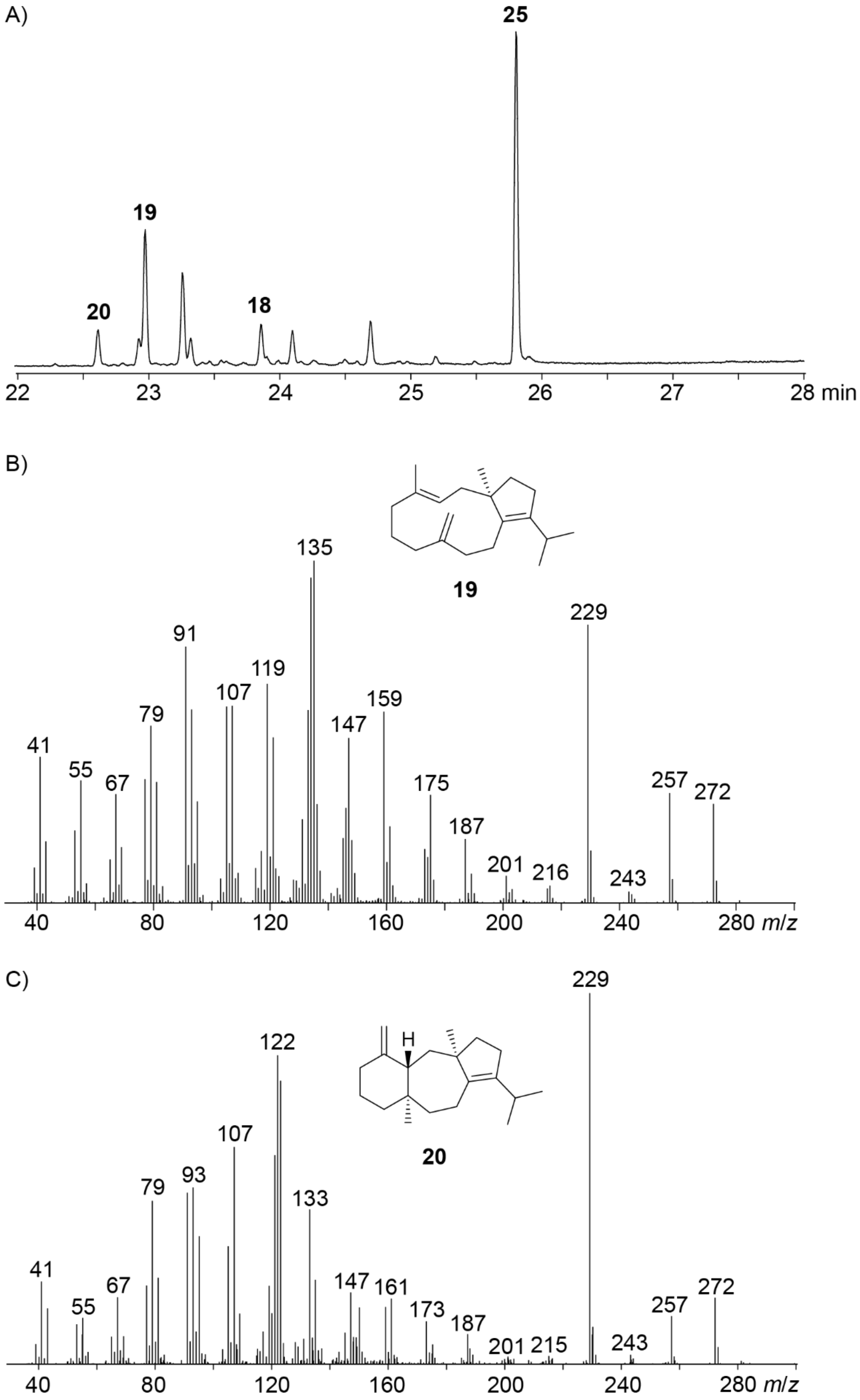

Figure S113. Product mixture formed from iso-GGPP I with CgDS. A) Total ion chromatogram of the crude extract from the enzyme incubation, B) EI mass spectrum of **19**, C) EI mass spectrum of **20**. Compound **25** was isolated from a different enzyme (AbVS) and is explained below.

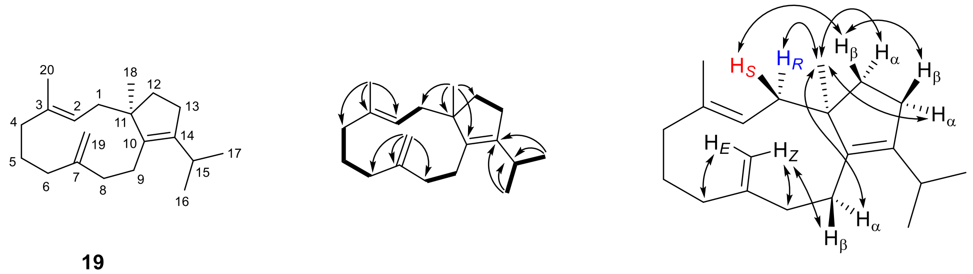

19

Figure S114. Structure elucidation of dolabella-3,8(17),11-triene (**19**). Bold: ^{1}H,^{1}H-COSY, single-headed arrows: key HMBC, and double-headed arrows: key NOESY correlations.

Table S14. NMR data of dolabella-3,8(17),11-triene (**19**) in C_6D_6 recorded at 298 K.

C[a]	type	^{13}C[b]	^{1}H[b]
1	CH$_2$	40.48	2.36 (m, H$_S$)
			1.95 (m, H$_R$)
2	CH	124.24	5.51 (m)
3	C$_q$	133.93	–
4	CH$_2$	41.55	2.00 (m)
			1.96 (m)
5	CH$_2$	26.54	1.77 (m)
			1.32 (m)
6	CH$_2$	33.24	2.20 (m)
			1.96 (m)
7	C$_q$	152.05	–
8	CH$_2$	35.67	2.35 (m)
			1.92 (m)
9	CH$_2$	24.86	2.16 (m, H$_\beta$)
			2.03 (m, H$_\alpha$)
10	C$_q$	140.56	–
11	C$_q$	51.61	–
12	CH$_2$	40.31	1.67 (m, H$_\beta$)
			1.62 (m, H$_\alpha$)
13	CH$_2$	26.84	2.22 (m, H$_\beta$)
			2.16 (m, H$_\alpha$)
14	C$_q$	142.72	–
15	CH	27.77	2.78 (hept, ^{3}J = 6.7)
16	CH$_3$	21.74	1.02 (d, ^{3}J = 6.8)
17	CH$_3$	21.19	0.95 (d, ^{3}J = 6.8)
18	CH$_3$	25.03	1.11 (s)
19	CH$_2$	110.76	4.81 (m, H$_Z$)
			4.77 (m, H$_E$)
20	CH$_3$	16.40	1.52 (br s)

[a] Carbon numbering as shown in Figure S114. [b] Chemical shifts δ in ppm, multiplicity: s = singlet, d = doublet, t = triplet, hept = heptet, m = multiplet, br = broad, coupling constants J are given in Hertz.

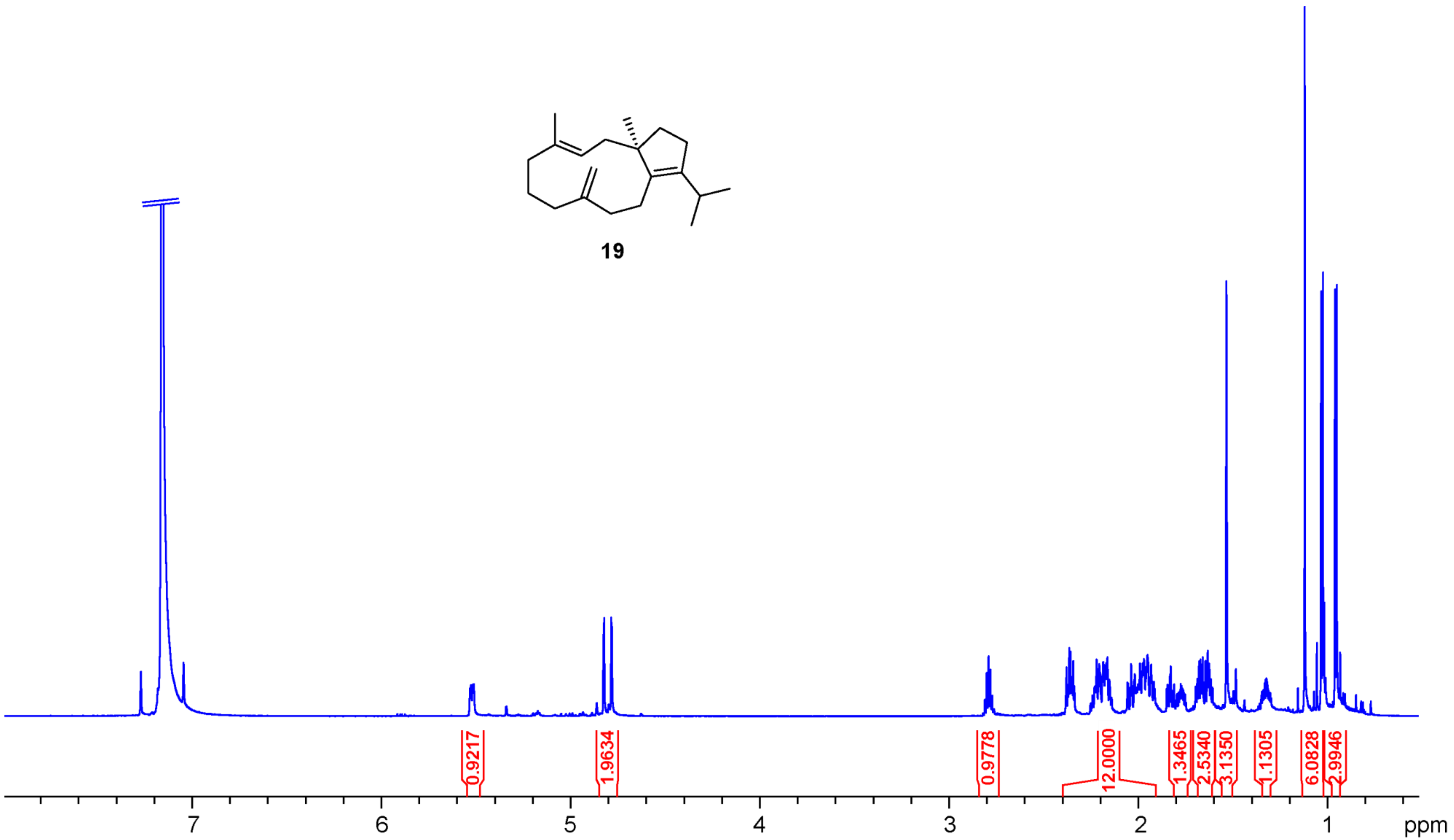

Figure S115. ^{1}H-NMR spectrum (700 MHz, C_6D_6) of **19**.

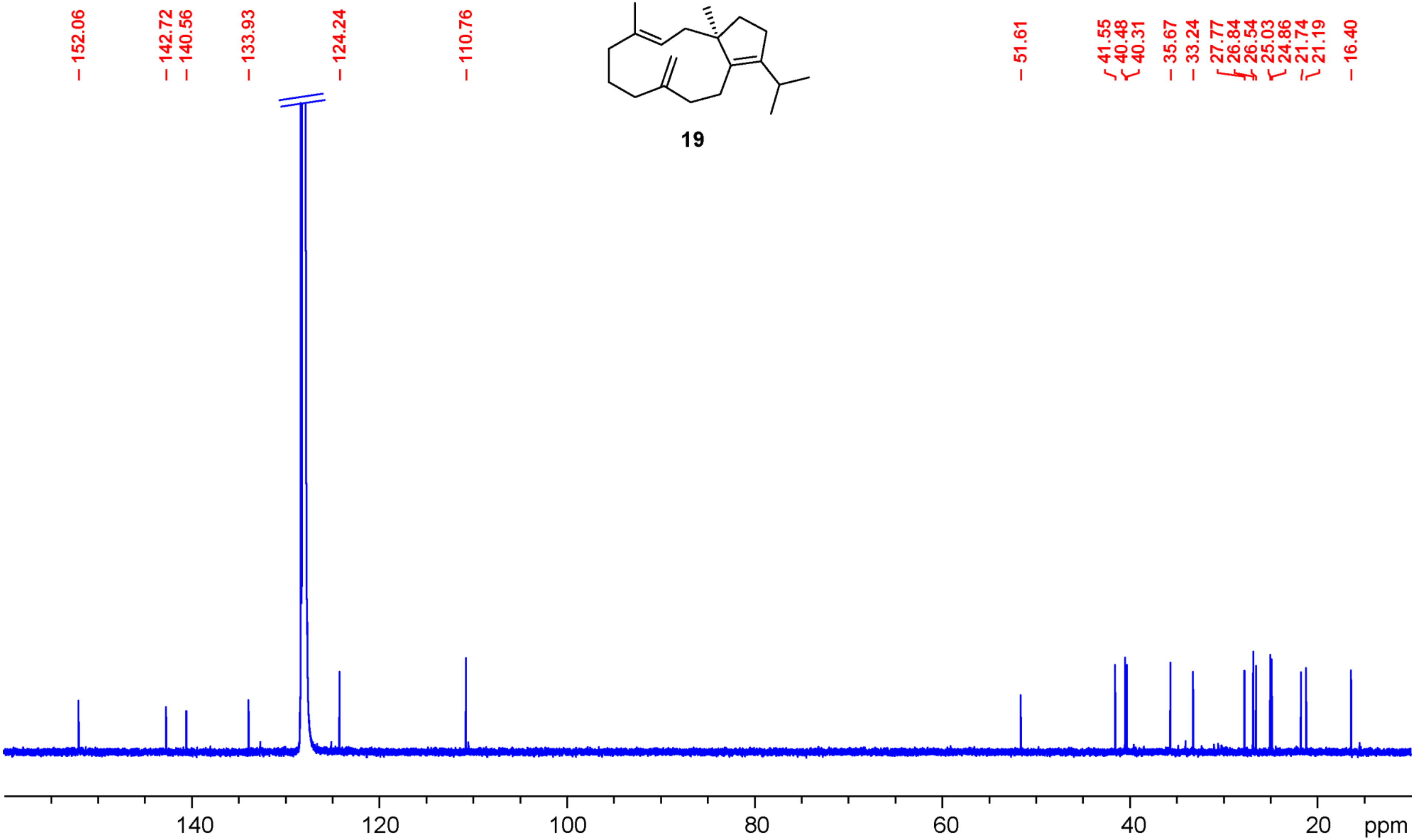

Figure S116. ^{13}C-NMR spectrum (176 MHz, C_6D_6) of **19**.

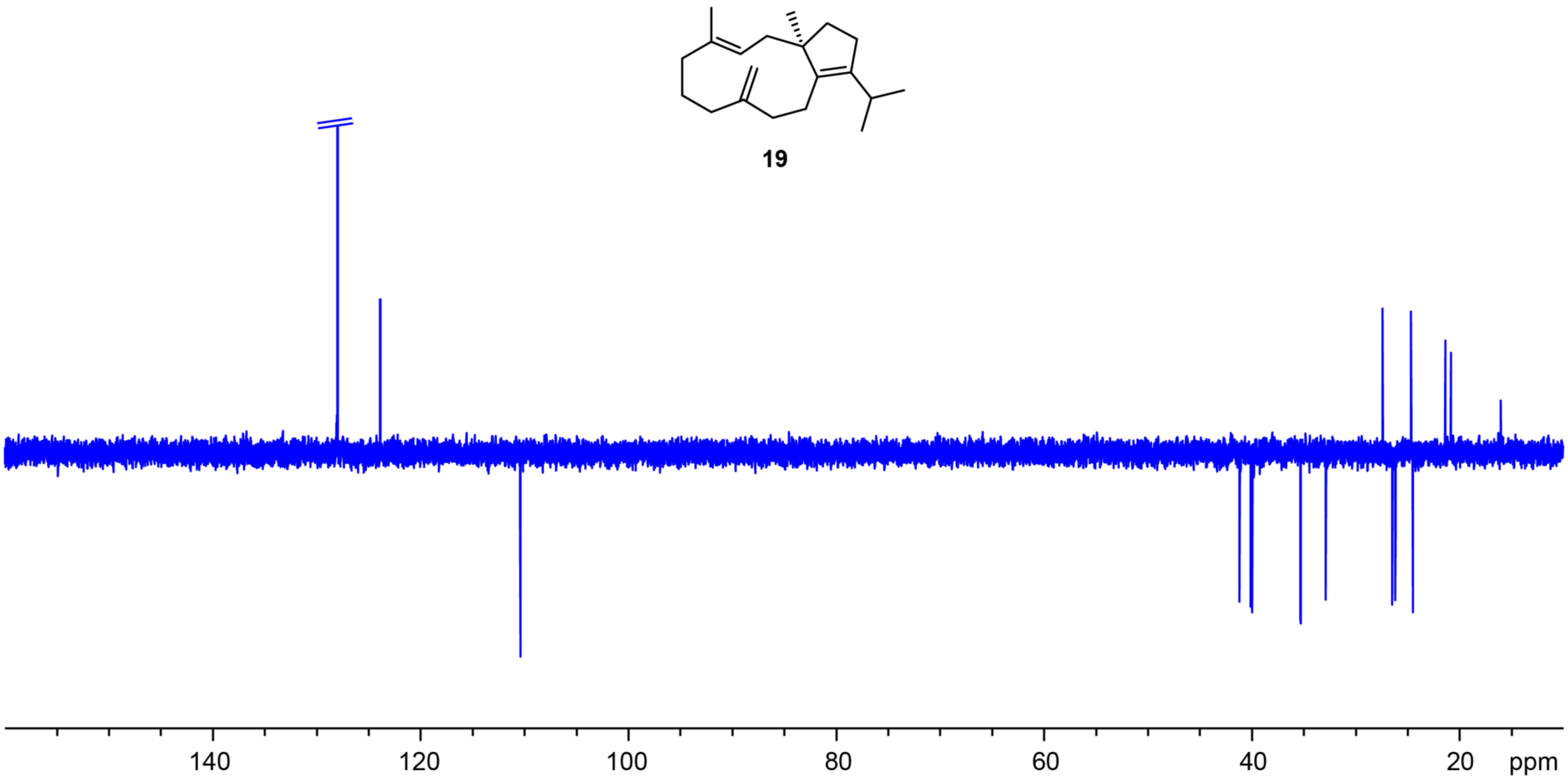

Figure S117. ^{13}C-DEPT135 spectrum (176 MHz, C_6D_6) of **19**.

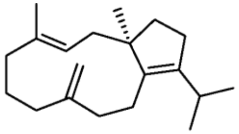

19

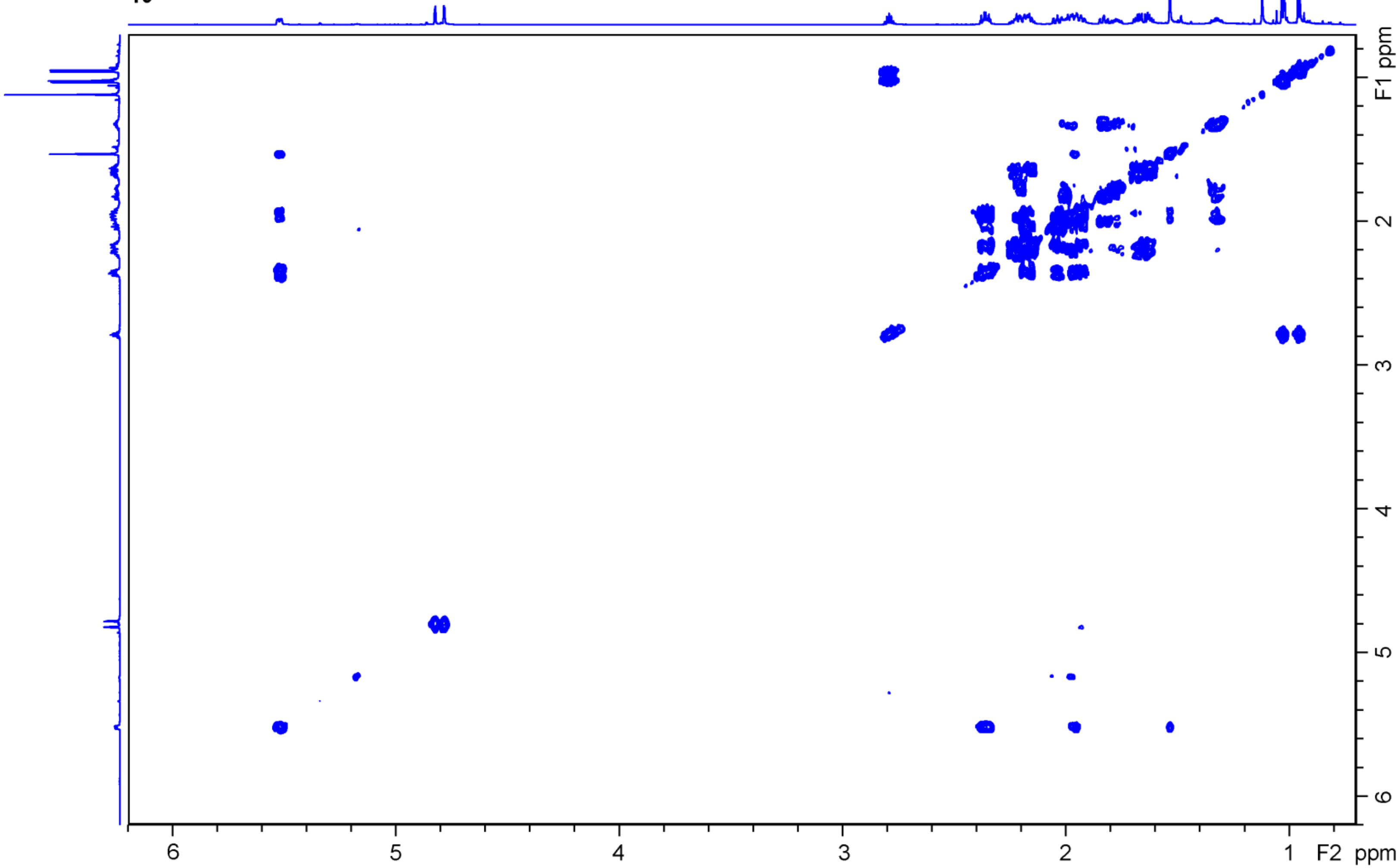

Figure S118. $^1H,^1H$-COSY spectrum (C_6D_6) of **19**.

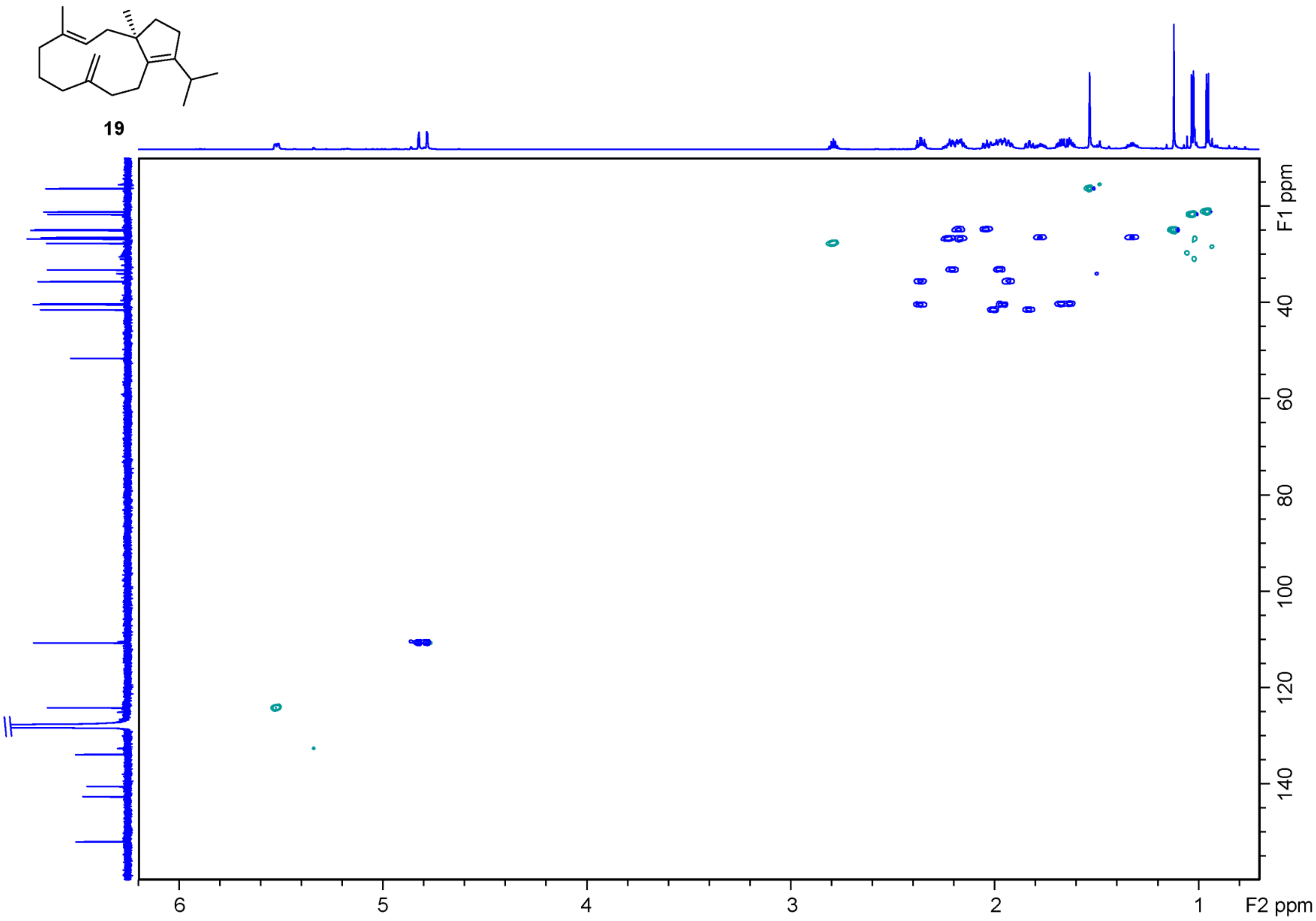

Figure S119. HSQC spectrum (C_6D_6) of **19**.

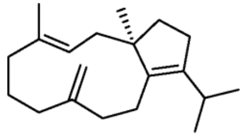

19

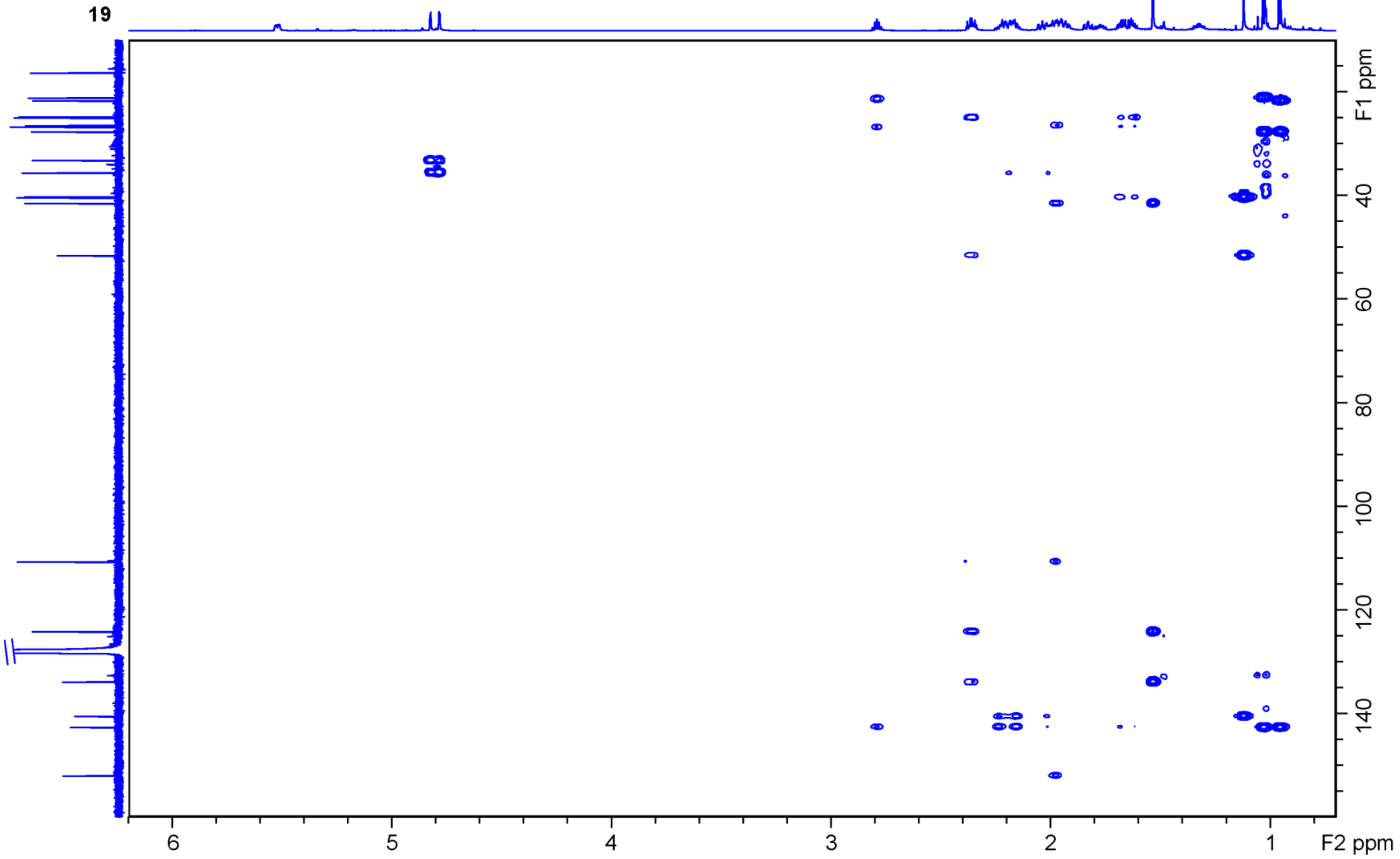

Figure S120. HMBC spectrum (C_6D_6) of **19**.

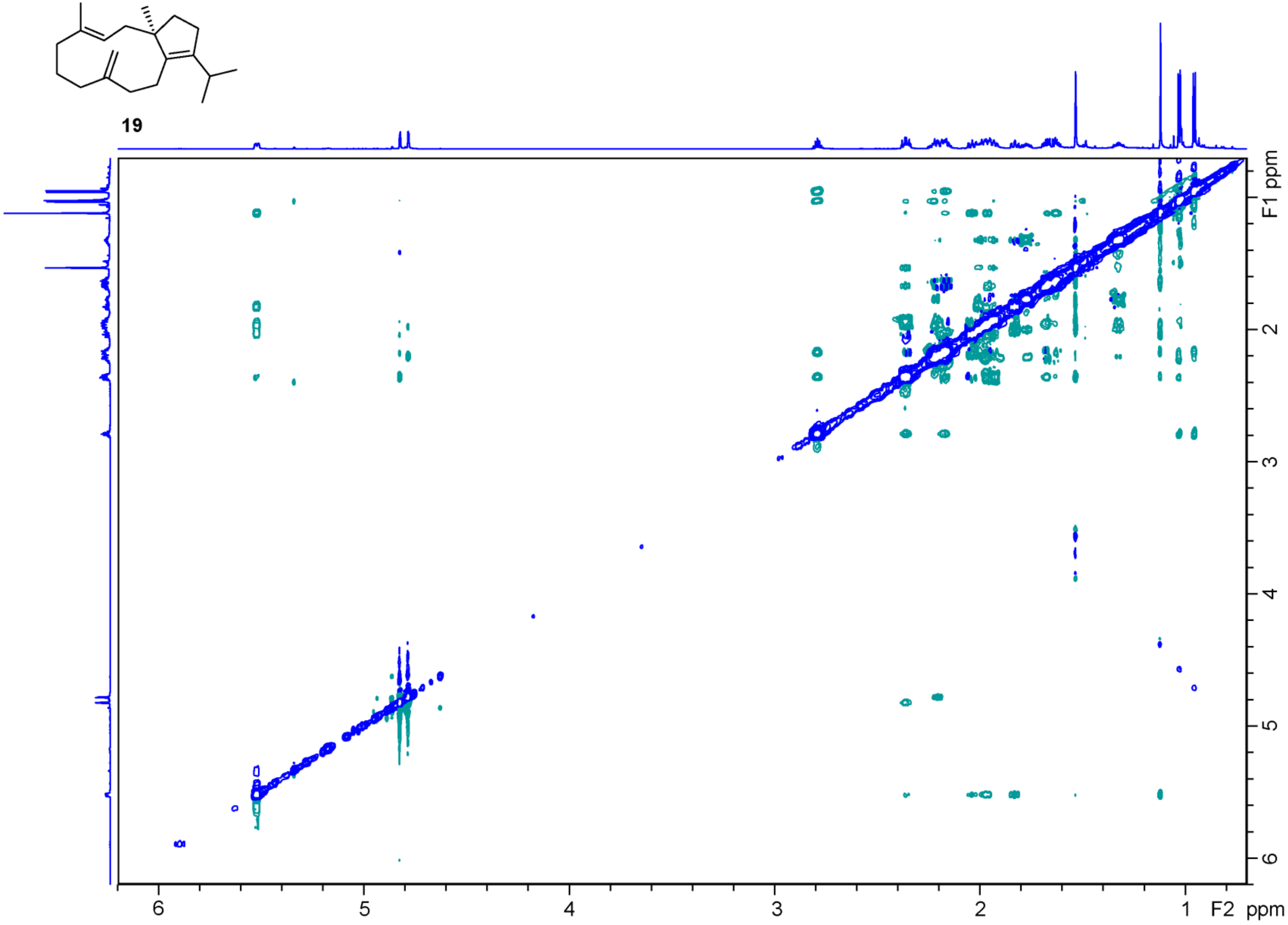

Figure S121. NOESY spectrum (C_6D_6) of **19**.

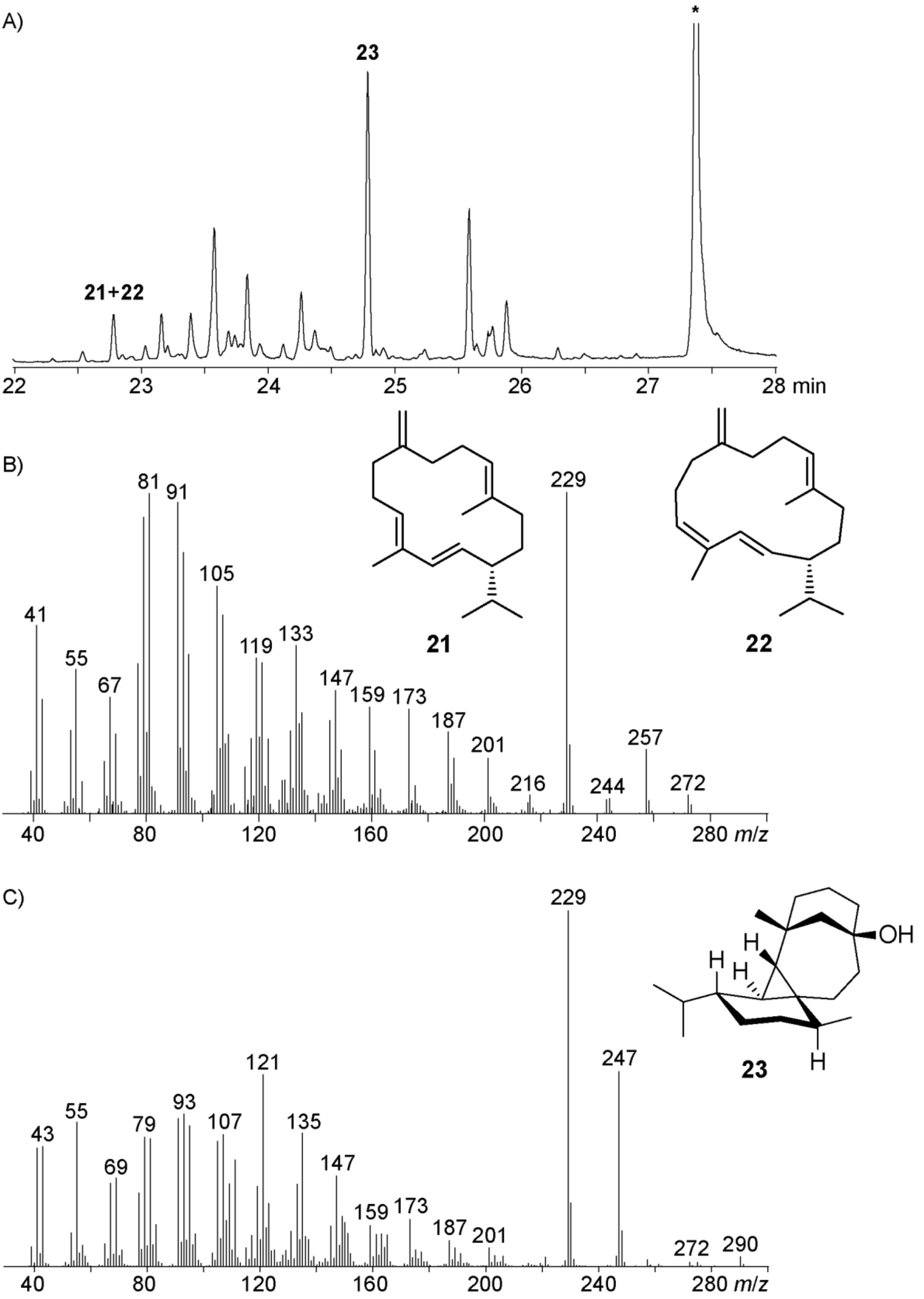

Figure S122. Product mixture formed from iso-GGPP I with SaS. A) Total ion chromatogram of the crude extract from the enzyme incubation (asterisks indicate contaminants), B) EI mass spectrum of the mixture of **21** and **22**, C) EI mass spectrum of **23**.

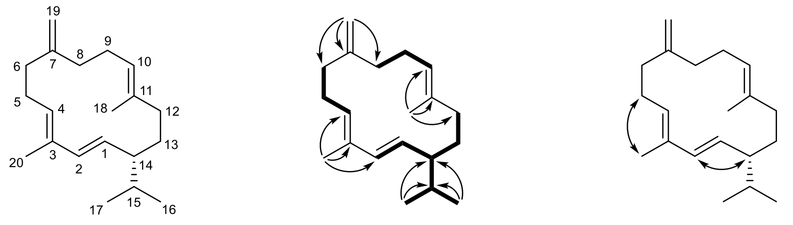

21

Figure S123. Structure elucidation of isothunbergene A (**21**). Bold: $^{1}H,^{1}H$-COSY, single-headed arrows: key HMBC, and double-headed arrows: key NOESY correlations.

Table S15. NMR data of isothunbergene A (**21**) in C_6D_6 recorded at 298 K.

C[a]	type	^{13}C[b]	^{1}H[b]
1	CH	128.01	5.15 (dd, ^{3}J = 15.4, 9.8)
2	CH	137.79	5.88 (d, ^{3}J = 15.5)
3	C_q	134.15	–
4	CH	129.79	5.34 (dd, ^{3}J = 10.0, 5.5)
5	CH_2	27.38	2.34 (dddd, ^{2}J = 13.8, ^{3}J = 9.9, 9.1, 4.7)
			2.12 (m)
6	CH_2	34.91	2.20 (m)
			1.94 (m)
7	C_q	149.02	–
8	CH_2	36.65	2.02 (m)
			1.95 (m)
9	CH_2	24.56	2.10 (m)
			2.04 (m)
10	CH	126.20	5.04 (br t, ^{3}J = 5.5)
11	C_q	133.07	–
12	CH_2	39.36	2.22 (m)
			2.02 (m)
13	CH_2	28.61	1.53 (dddd ^{2}J = 13.7, ^{3}J = 9.2, 3.7, 2.6)
			1.35 (dddd, ^{2}J = 13.7, ^{3}J = 11.6, 9.3, 2.6)
14	CH	52.34	1.75 (m)
15	CH	33.46	1.48 (oct, ^{3}J = 6.6)
16	CH_3	21.10	0.92 (d, ^{3}J = 6.8)
17	CH_3	19.91	0.90 (d, ^{3}J = 6.8)
18	CH_3	15.52	1.49 (br s)
19	CH_2	110.79	4.89 (br s, 2H)
20	CH_3	13.21	1.65 (t, ^{4}J = 0.9)

[a] Carbon numbering as shown in Figure S123. [b] Chemical shifts δ in ppm, multiplicity: s = singlet, d = doublet, t = triplet, oct = octet, m = multiplet, br = broad, coupling constants J are given in Hertz.

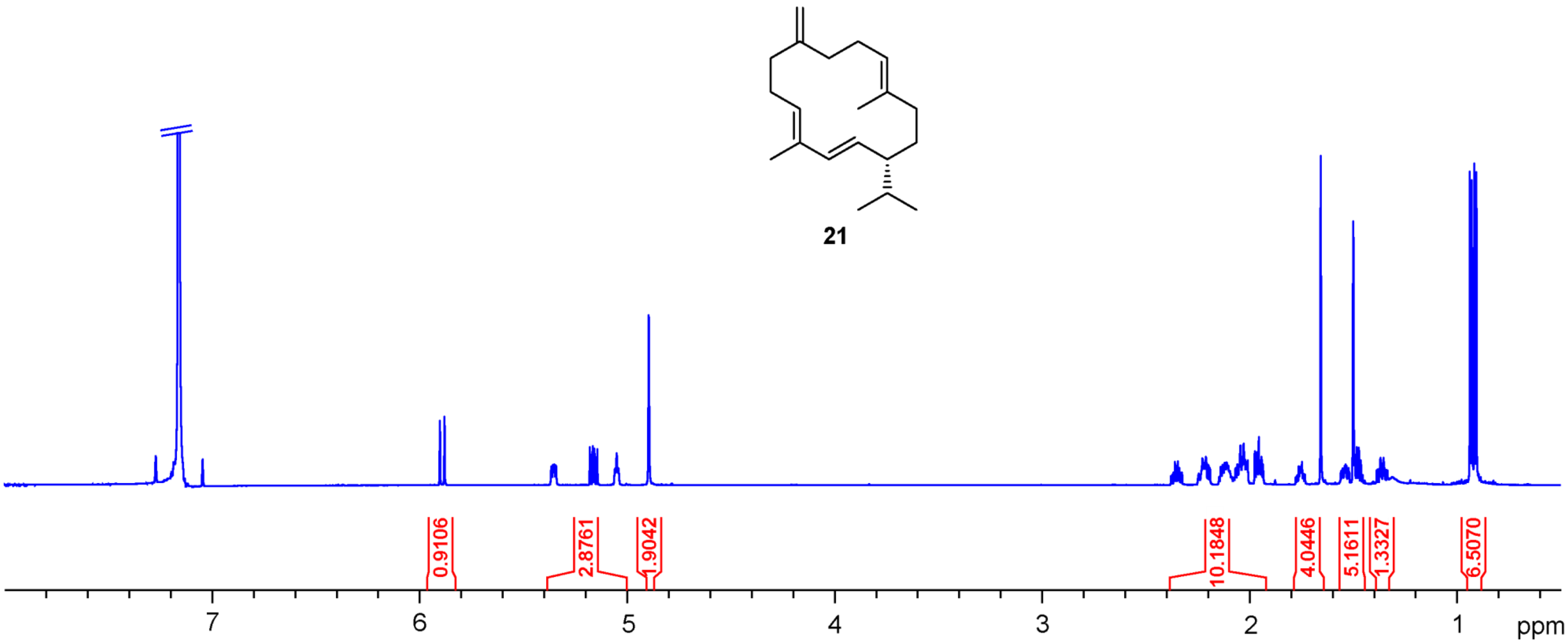

Figure S124. ^{1}H-NMR spectrum (700 MHz, C_6D_6) of **21**.

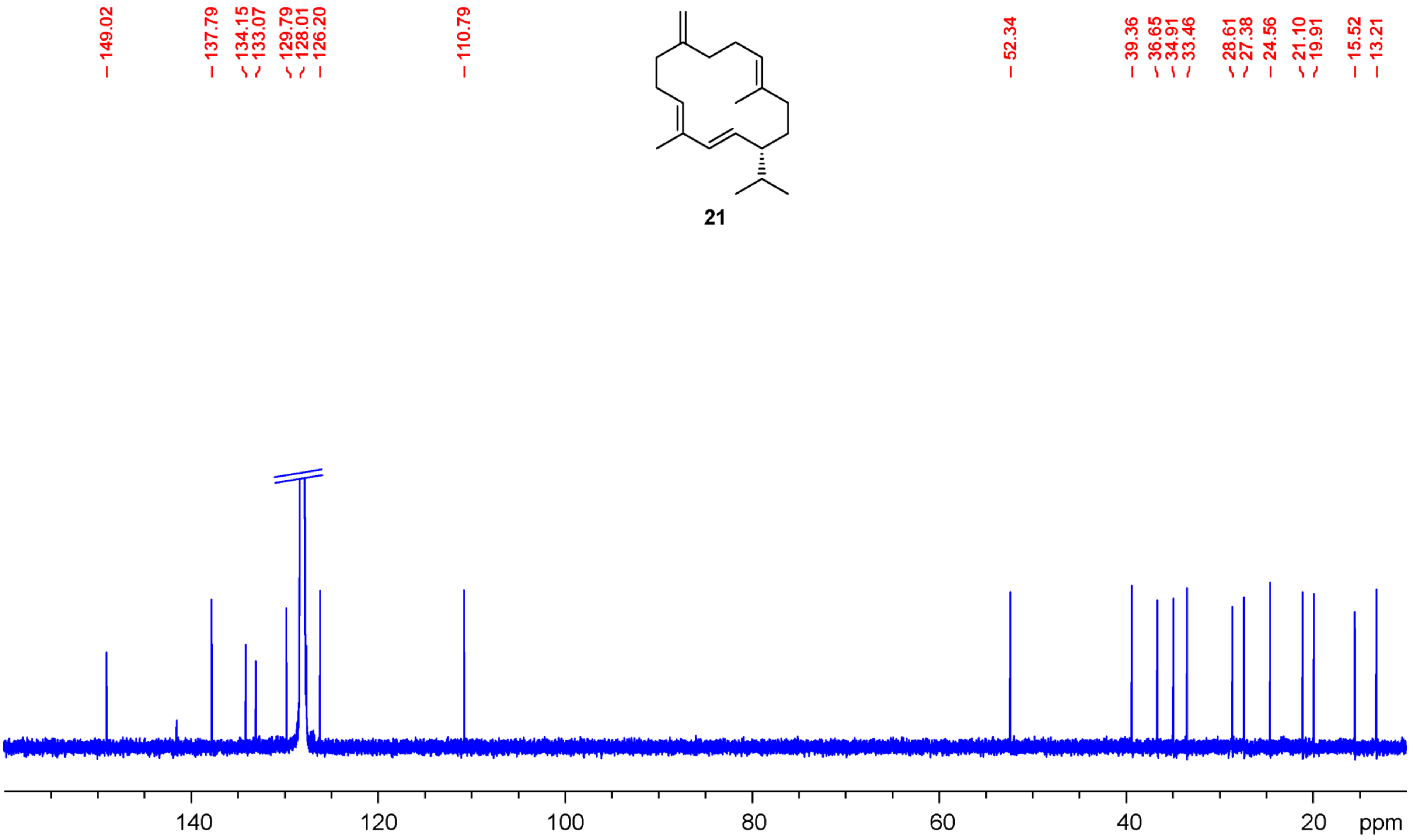

21

Figure S125. ^{13}C-NMR spectrum (176 MHz, C_6D_6) of **21**.

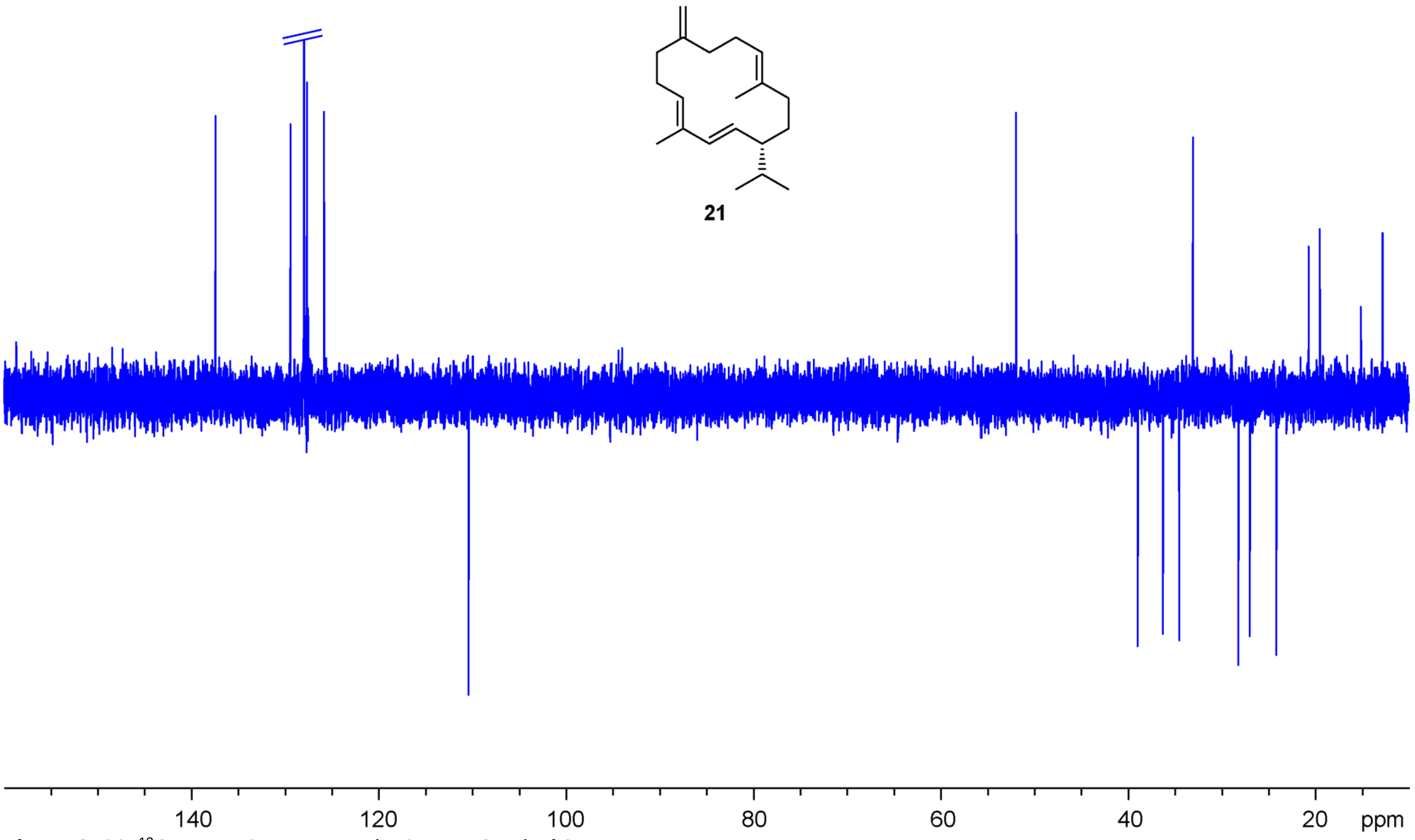

Figure S126. ^{13}C-DEPT135 spectrum (176 MHz, C_6D_6) of **21**.

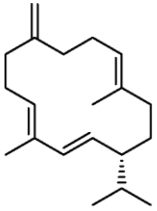

21

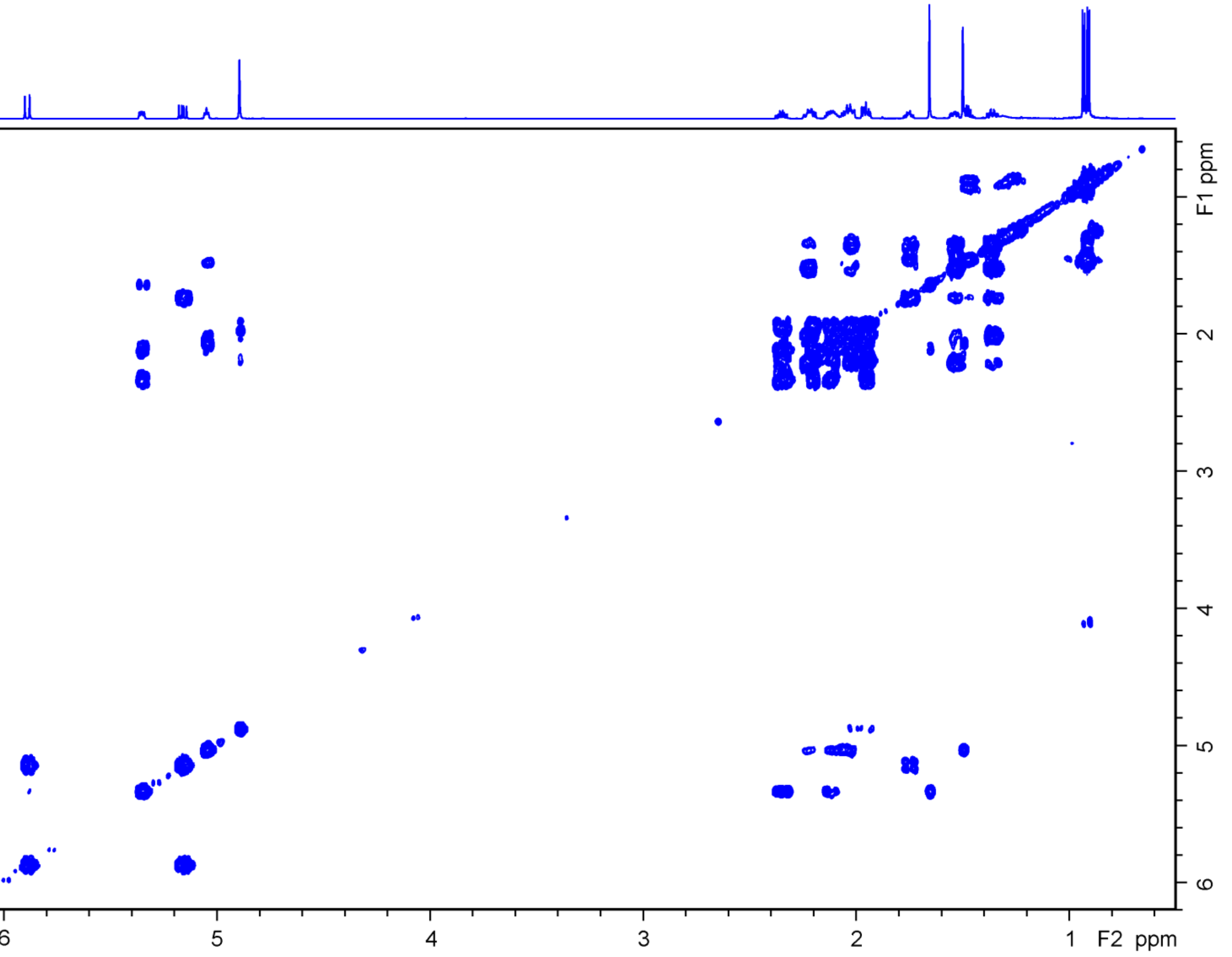

Figure S127. ^{1}H,^{1}H-COSY spectrum (C_6D_6) of **21**.

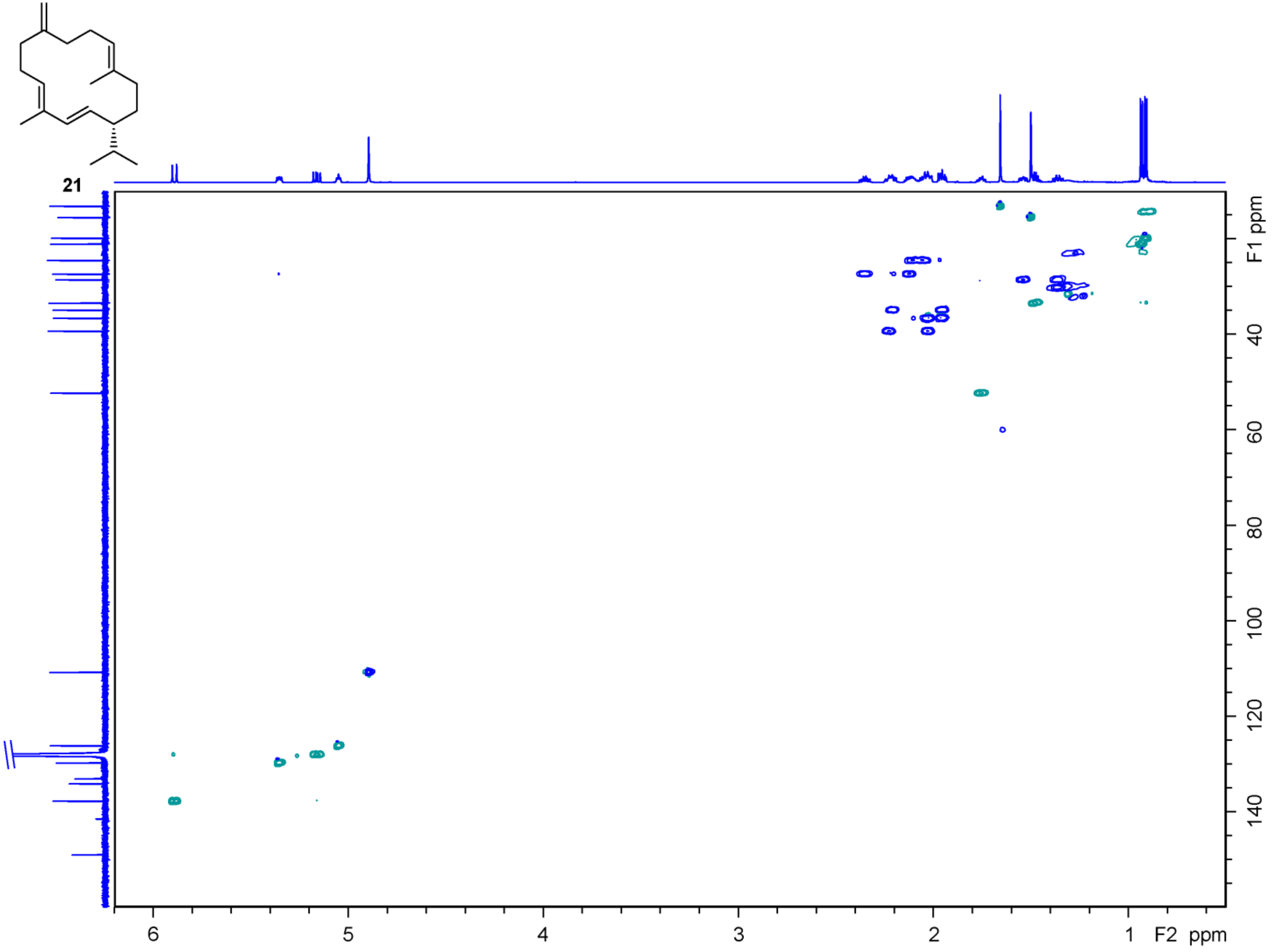

Figure S128. HSQC spectrum (C_6D_6) of **21**.

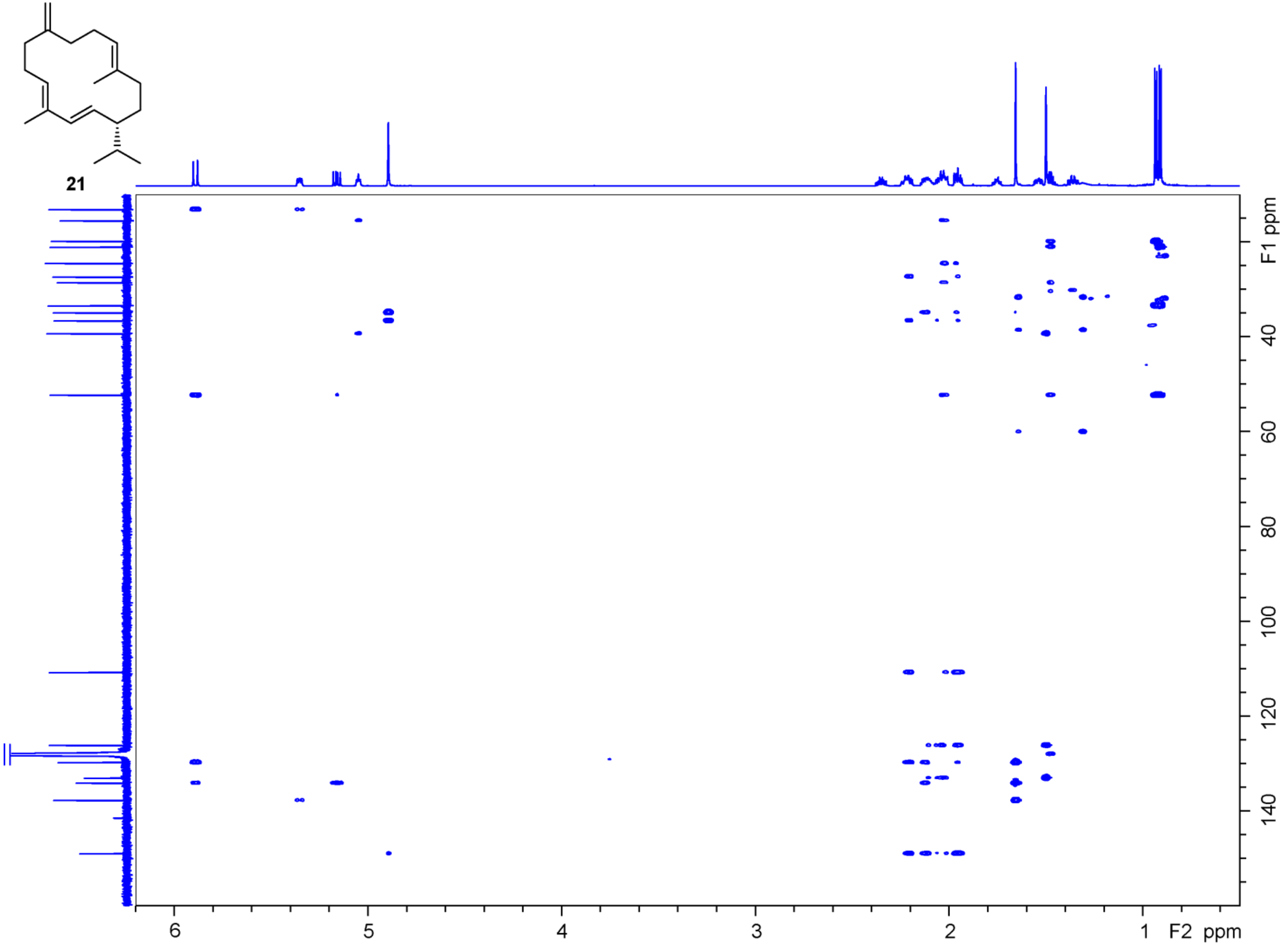

Figure S129. HMBC spectrum (C_6D_6) of **21**.

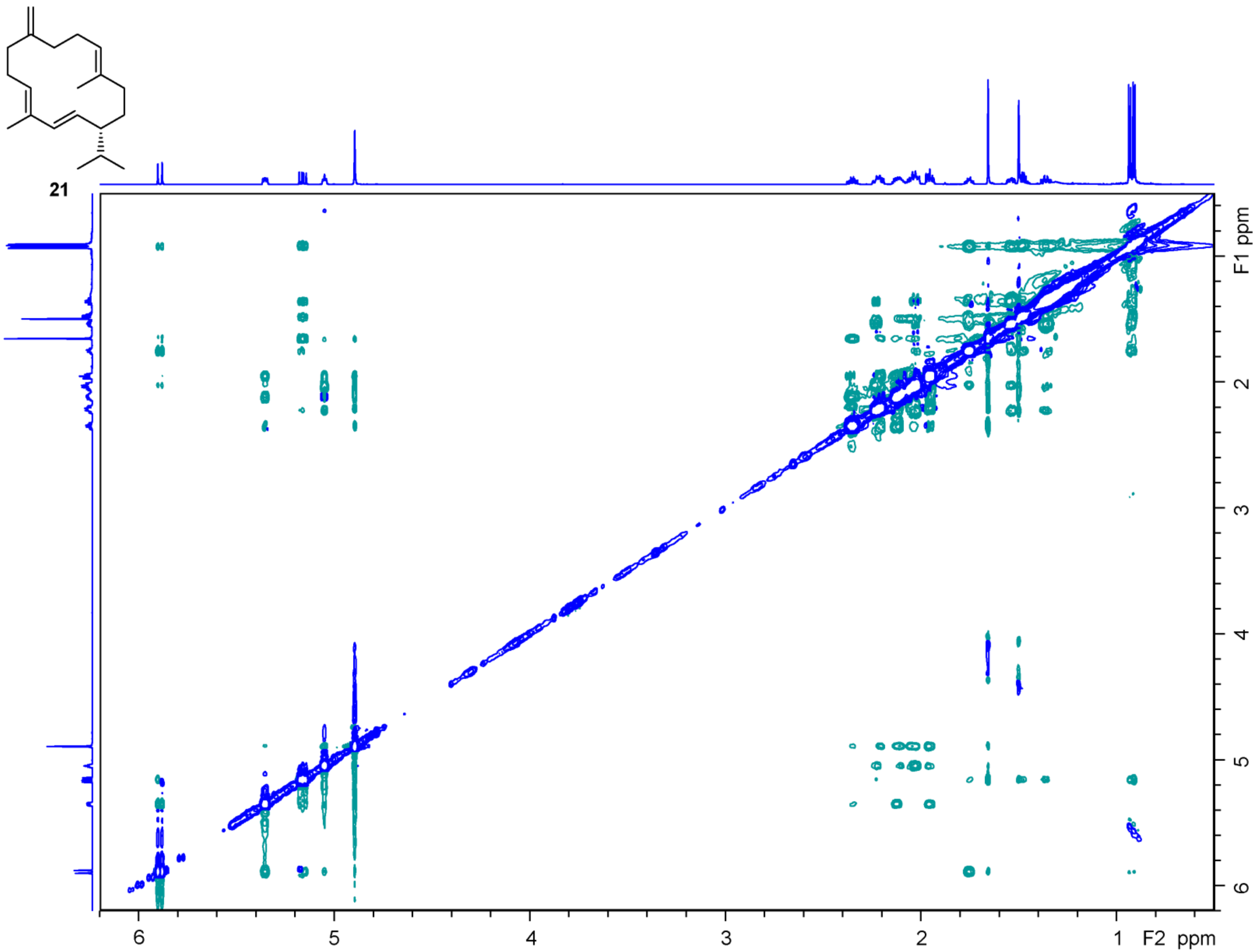

Figure S130. NOESY spectrum (C_6D_6) of **21**.

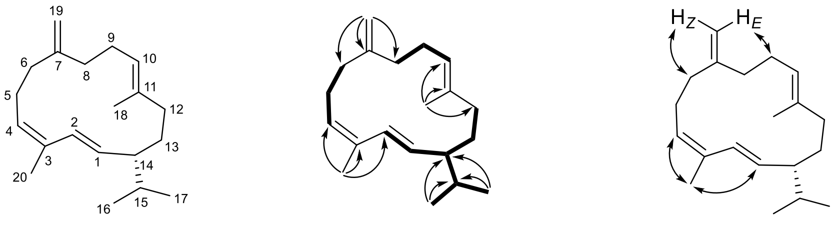

22

Figure S131. Structure elucidation of isothunbergene B (**22**). Bold: ^{1}H,^{1}H-COSY, single-headed arrows: key HMBC, and double-headed arrows: key NOESY correlations.

Table S16. NMR data of isothunbergene B (**22**) in C_6D_6 recorded at 298 K.

C[a]	type	^{13}C[b]	^{1}H[b]
1	CH	130.59	5.28 (dd, ^3J = 15.5, 9.8)
2	CH	131.07	6.37 (d, ^3J = 15.4)
3	C_q	133.24	–
4	CH	128.31	5.20 (dd, ^3J = 10.8, 5.3)
5	CH_2	25.75	2.59 (dddd, ^2J = 13.8, ^3J = 11.0, 8.9, 7.0)
			1.89 (m)
6	CH_2	38.79	2.07 (m, 2H)
7	C_q	146.35	–
8	CH_2	33.22	1.98 (m, 2H)
9	CH_2	24.48	2.32 (dddd, ^2J = 14.9, ^3J = 10.3, 5.9, 3.2)
10	CH	126.44	5.43 (br d, ^3J = 10.3)
11	C_q	131.31	–
12	CH_2	37.96	2.12 (ddd, ^2J = 13.4, ^3J = 13.4, 4.3)
			2.07 (m)
13	CH_2	27.77	1.67 (dddd, ^2J = 13.6, ^3J = 12.8, 4.8, 3.8)
			1.20 (dddd, ^2J = 13.7, ^3J = 12.1, 4.2, 4.2)
14	CH	49.36	1.80 (dddd, ^3J = 11.3, 9.7, 6.0, 3.7)
15	CH	33.46	1.48 (oct, ^3J = 6.8)
16	CH_3	20.91	0.91 (d, ^3J = 6.8)
17	CH_3	20.31	0.88 (d, ^3J = 6.8)
18	CH_3	14.20	1.54 (br s)
19	CH_2	110.46	4.86 (q, ^4J = 1.6, H_E)
			4.82 (m, H_Z)
20	CH_3	20.74	1.87 (t, ^4J = 1.1)

[a] Carbon numbering as shown in Figure S131. [b] Chemical shifts δ in ppm, multiplicity: s = singlet, d = doublet, t = triplet, q = quartet, oct = octet, m = multiplet, br = broad, coupling constants J are given in Hertz.

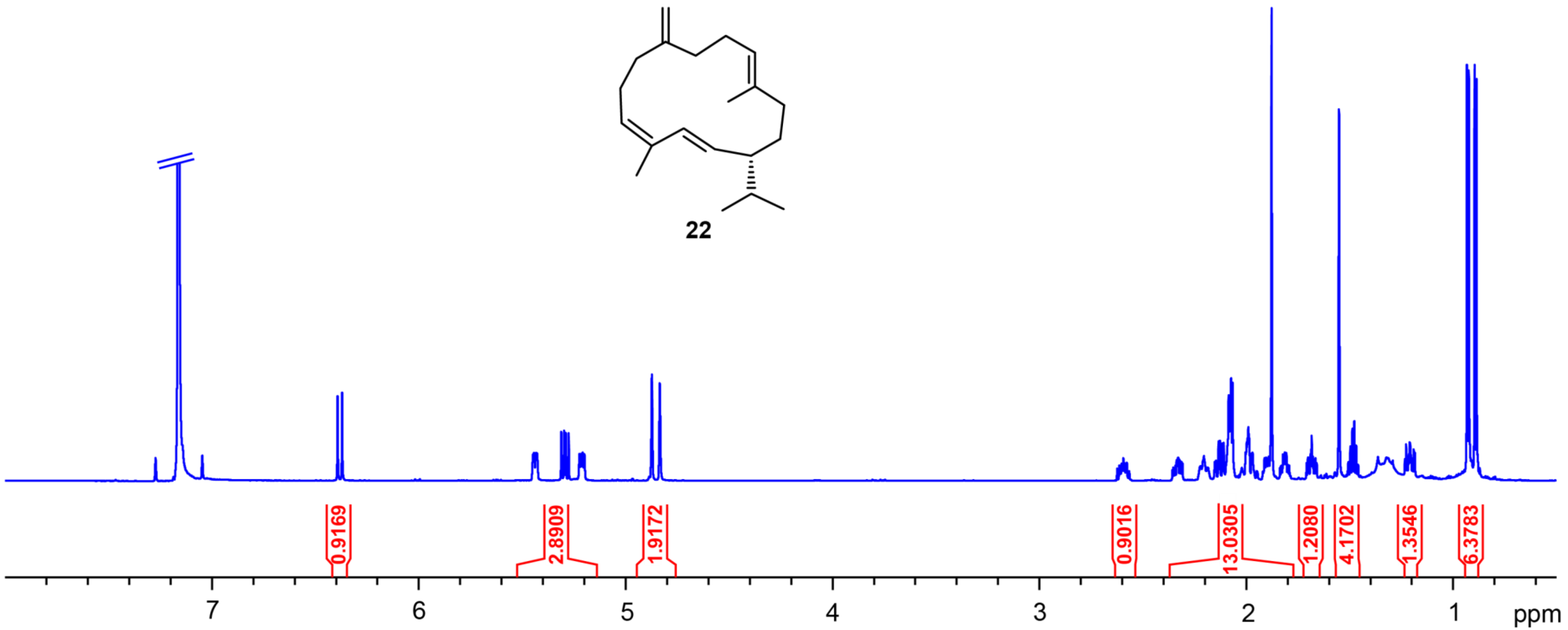

Figure S132. ^{1}H-NMR spectrum (700 MHz, C_6D_6) of **22**.

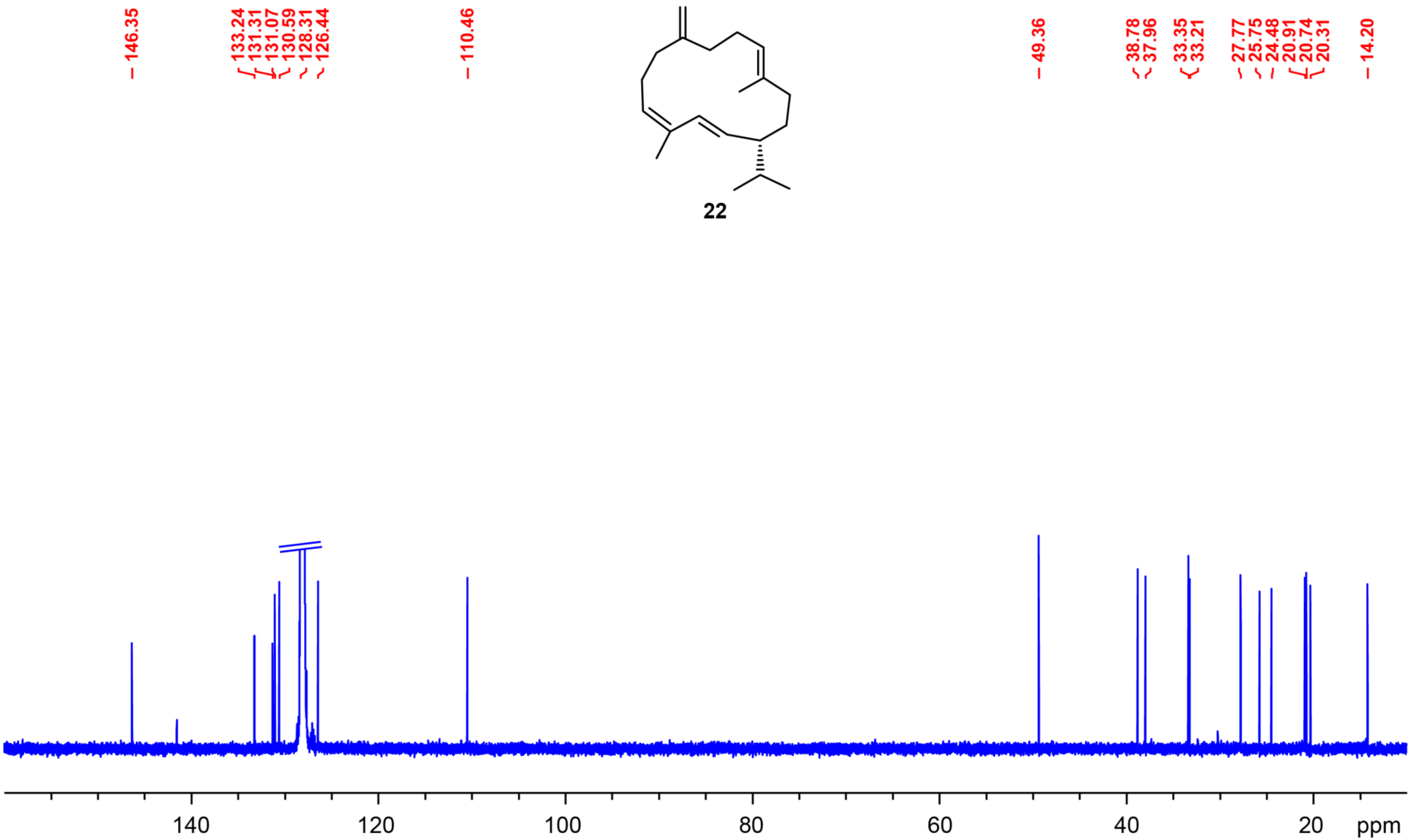

Figure S133. ^{13}C-NMR spectrum (176 MHz, C_6D_6) of **22**.

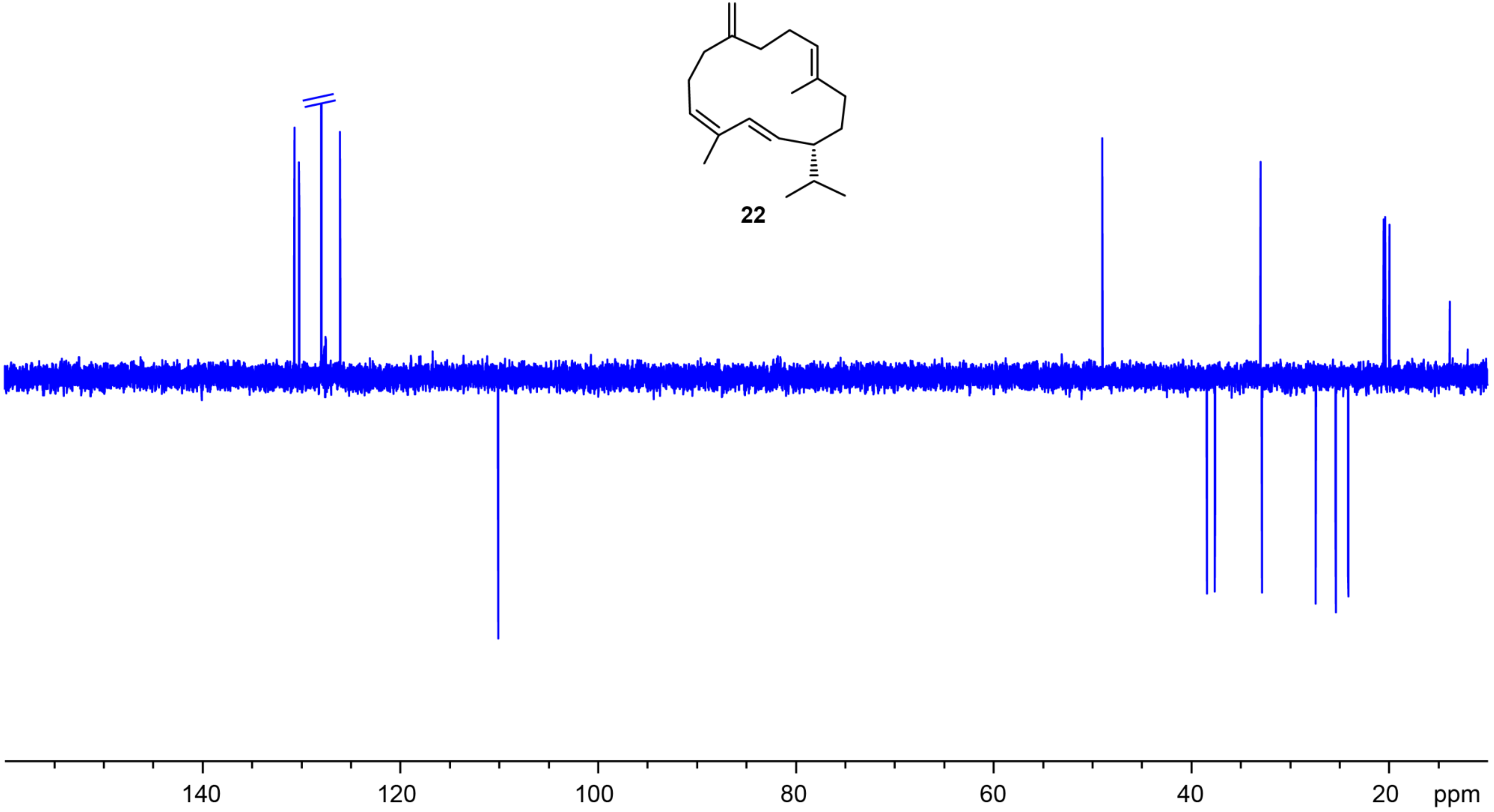

Figure S134. ^{13}C-DEPT135 spectrum (176 MHz, C_6D_6) of **22**.

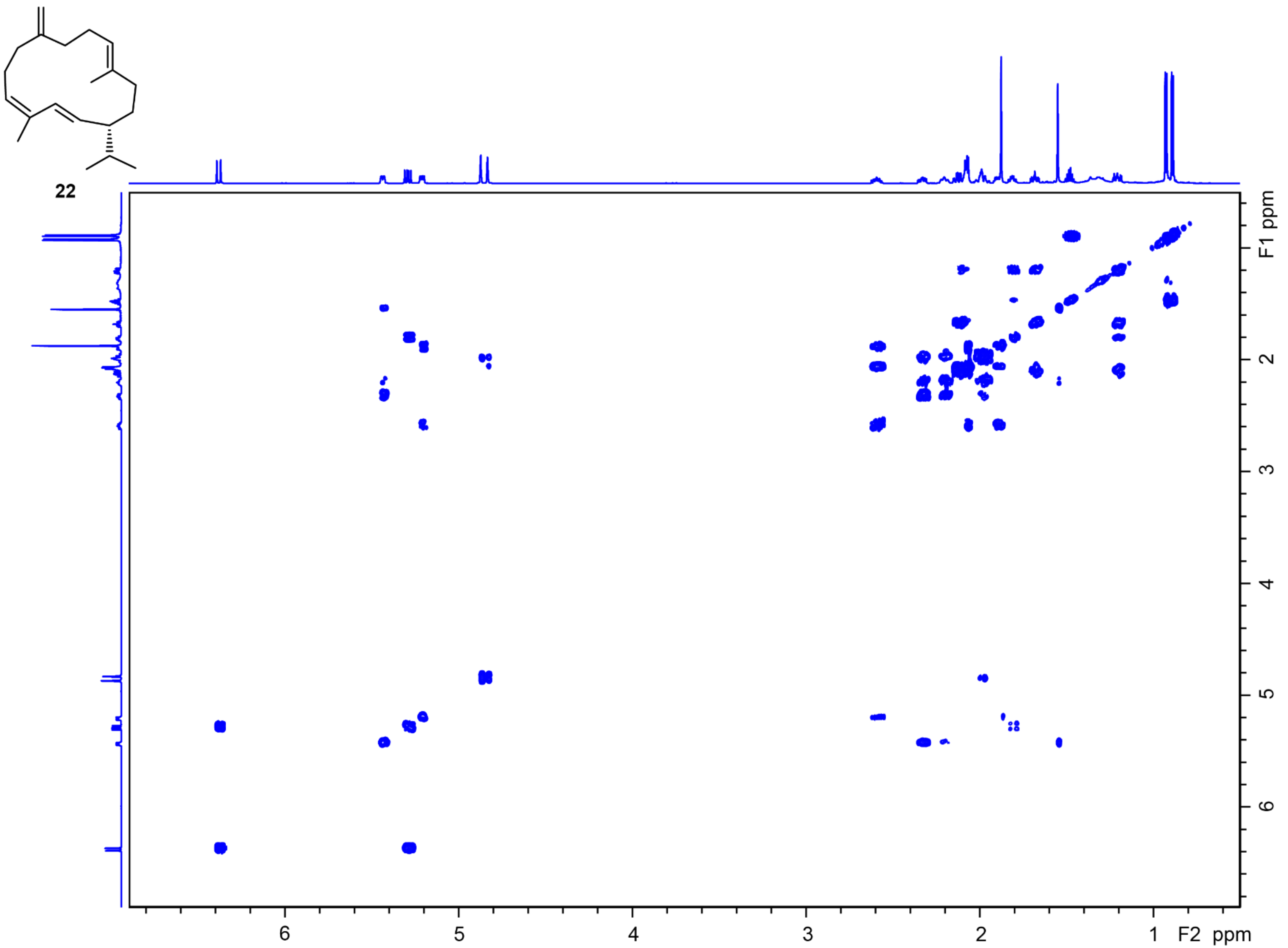

Figure S135. ^{1}H,^{1}H-COSY spectrum (C_6D_6) of **22**.

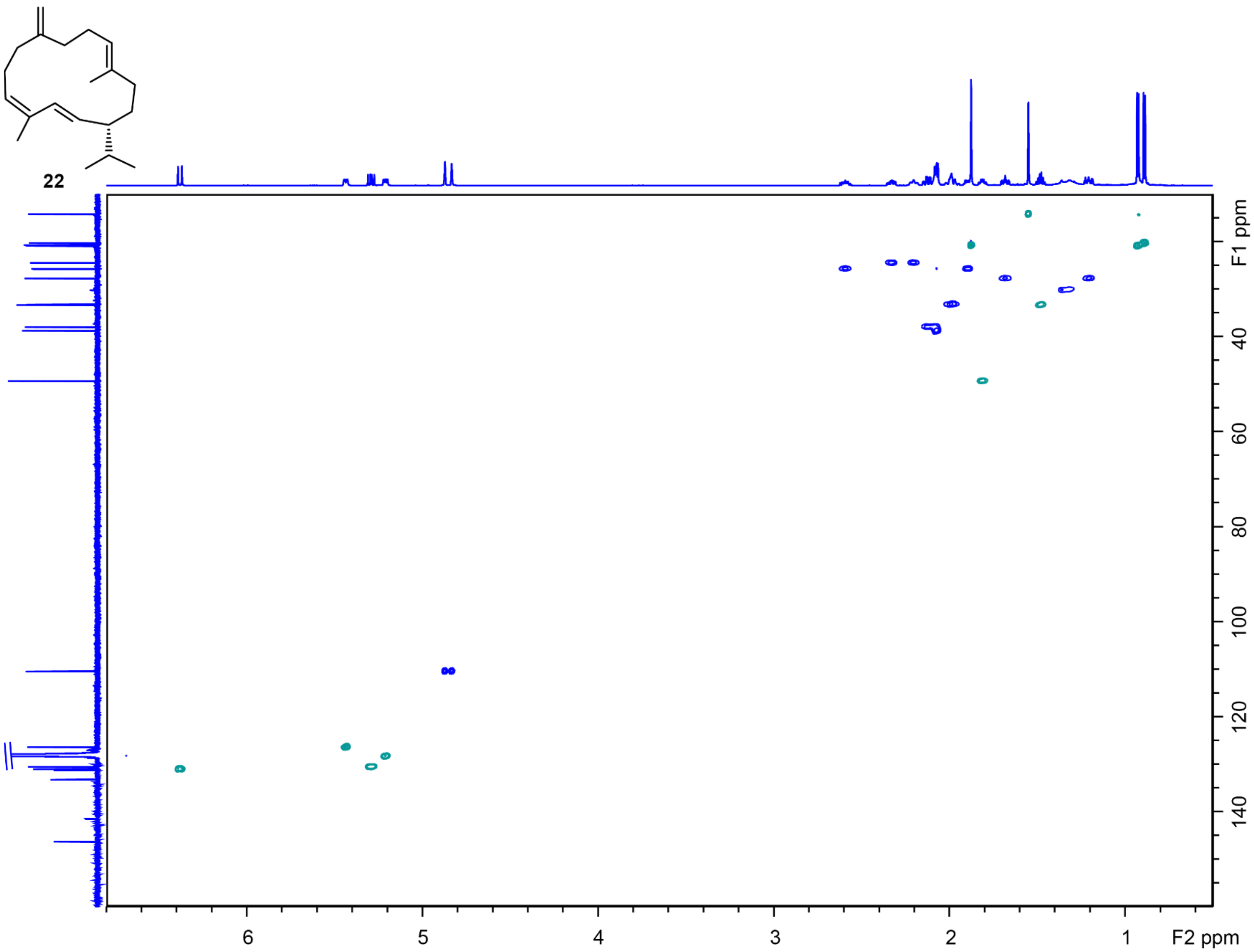

Figure S136. HSQC spectrum (C_6D_6) of **22**.

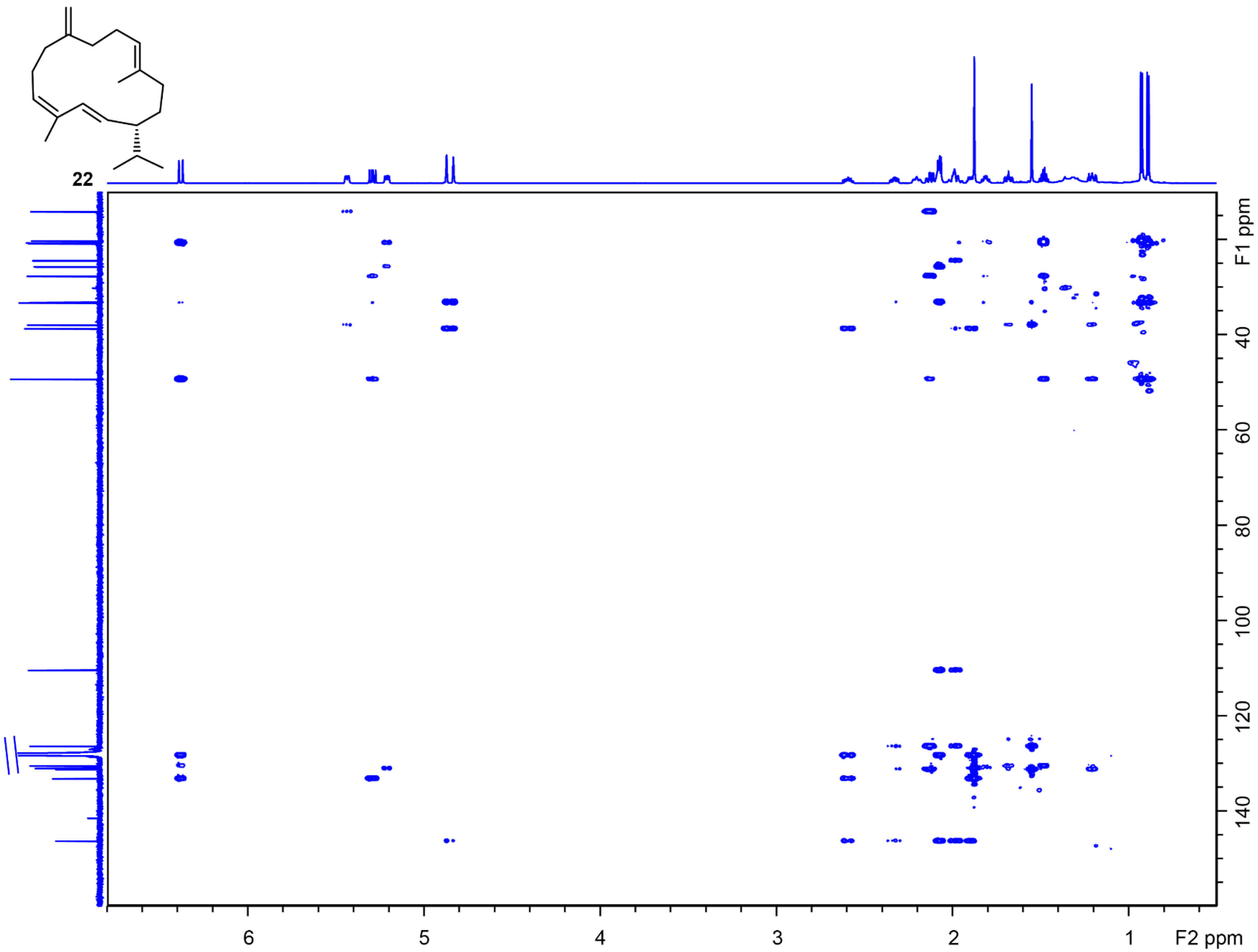

Figure S137. HMBC spectrum (C_6D_6) of **22**.

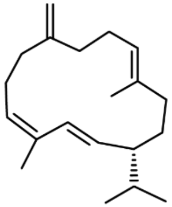
22

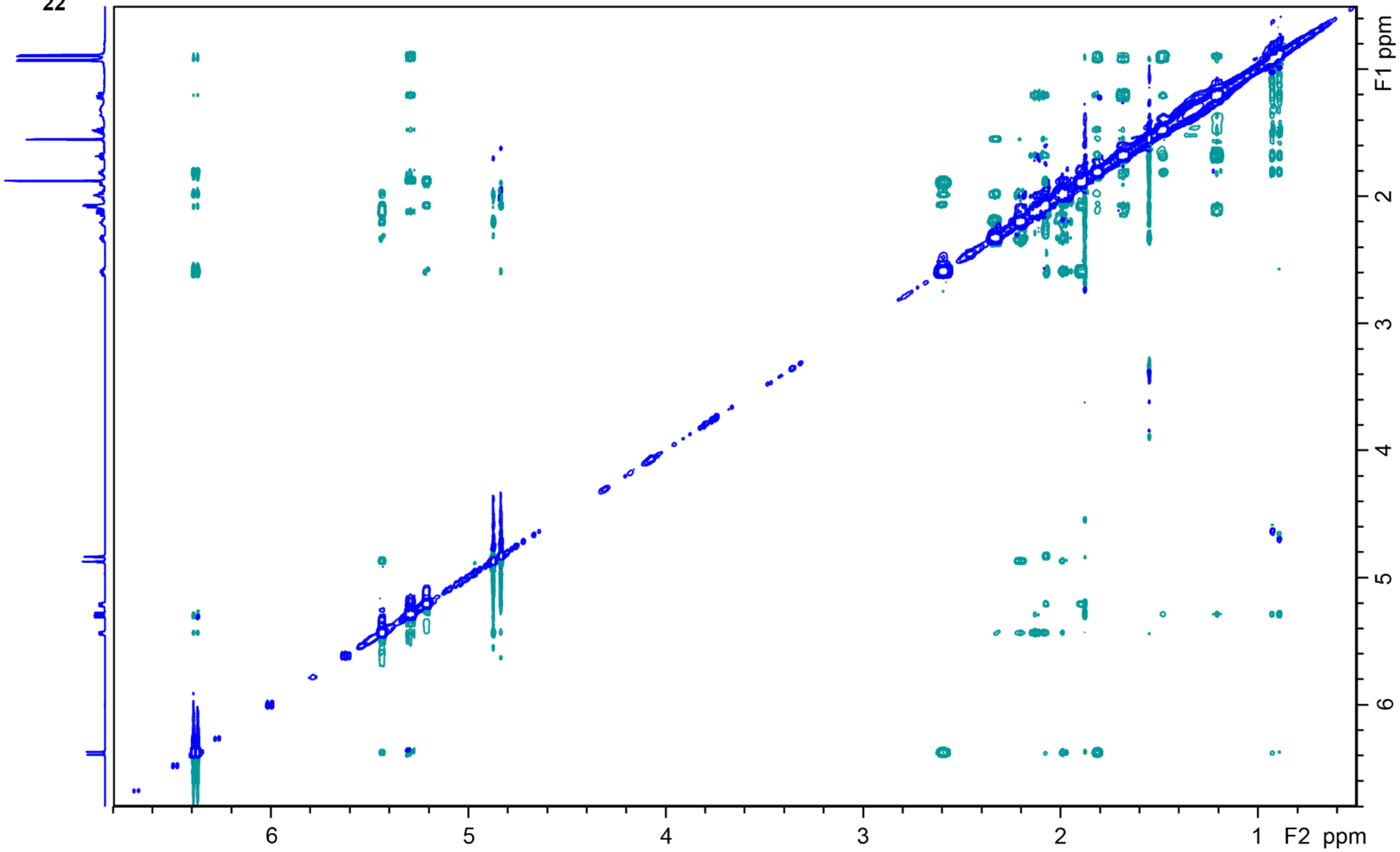

Figure S138. NOESY spectrum (C_6D_6) of **22**.

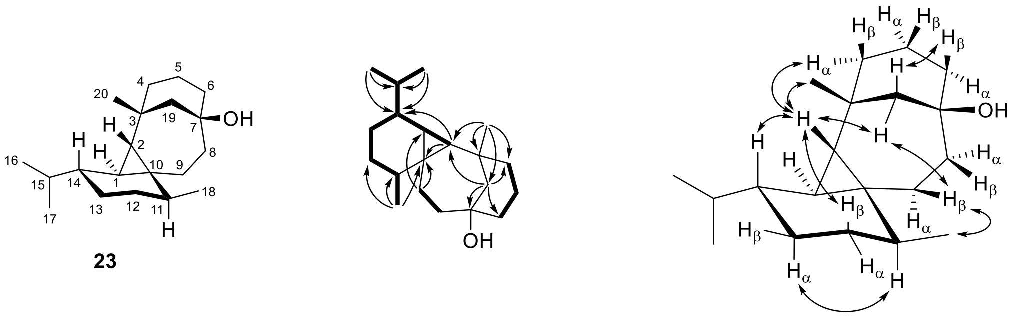

Figure S139. Structure elucidation of albataxenol (**23**). Bold: ^{1}H,^{1}H-COSY, single-headed arrows: key HMBC, and double-headed arrows: key NOESY correlations.

Table S17. NMR data of albataxenol (**23**) in C_6D_6 recorded at 344 K.

C[a]	type	^{13}C[b]	^{1}H[b]
1	CH	23.58	1.05 (m)
2	CH	36.92	0.37 (d, ^3J = 6.5)
3	C_q	33.66	–
4	CH_2	36.84	1.68 (m, H_α)
			1.04 (m, H_β)
5	CH_2	22.99	1.66 (m)
			1.50 (m)
6	CH_2	40.19	1.58 (m, H_α)
			1.16 (m, H_β)
7	C_q	71.34	–
8	CH_2	37.38	1.68 (m)
			second signal missing due to line broadening
9	CH_2	31.34	2.14 (m, H_β)
			1.56 (m, H_α)
10	C_q	29.34	–
11	CH	37.75	1.42 (m)
12	CH_2	30.55	1.34 (m, H_α)
			0.57 (dddd, ^2J = 13.3, ^3J = 12.2, 12.2, 2.2, H_β)
13	CH_2	27.64	1.30 (m, H_β)
			0.70 (dddd, ^2J = 13.2, ^3J = 12.3, 12.3, 2.2, H_α)
14	CH	45.27	1.08 (m)
15	CH	34.25	1.53 (m)
16	CH_3	19.89	0.96 (d, J = 6.6)
17	CH_3	20.42	0.92 (d, J = 6.8)
18	CH_3	20.58	1.02 (d, J = 6.4)
19	CH_2	51.48	1.87 (ddd, ^2J = 12.6, ^4J = 2.0, 2.0)
			1.00 (d, ^2J = 13.0)
20	CH_3	38.53	1.08 (s)

[a] Carbon numbering as shown in Figure S139. [b] Chemical shifts δ in ppm, multiplicity: s = singlet, d = doublet, m = multiplet, coupling constants J are given in Hertz.

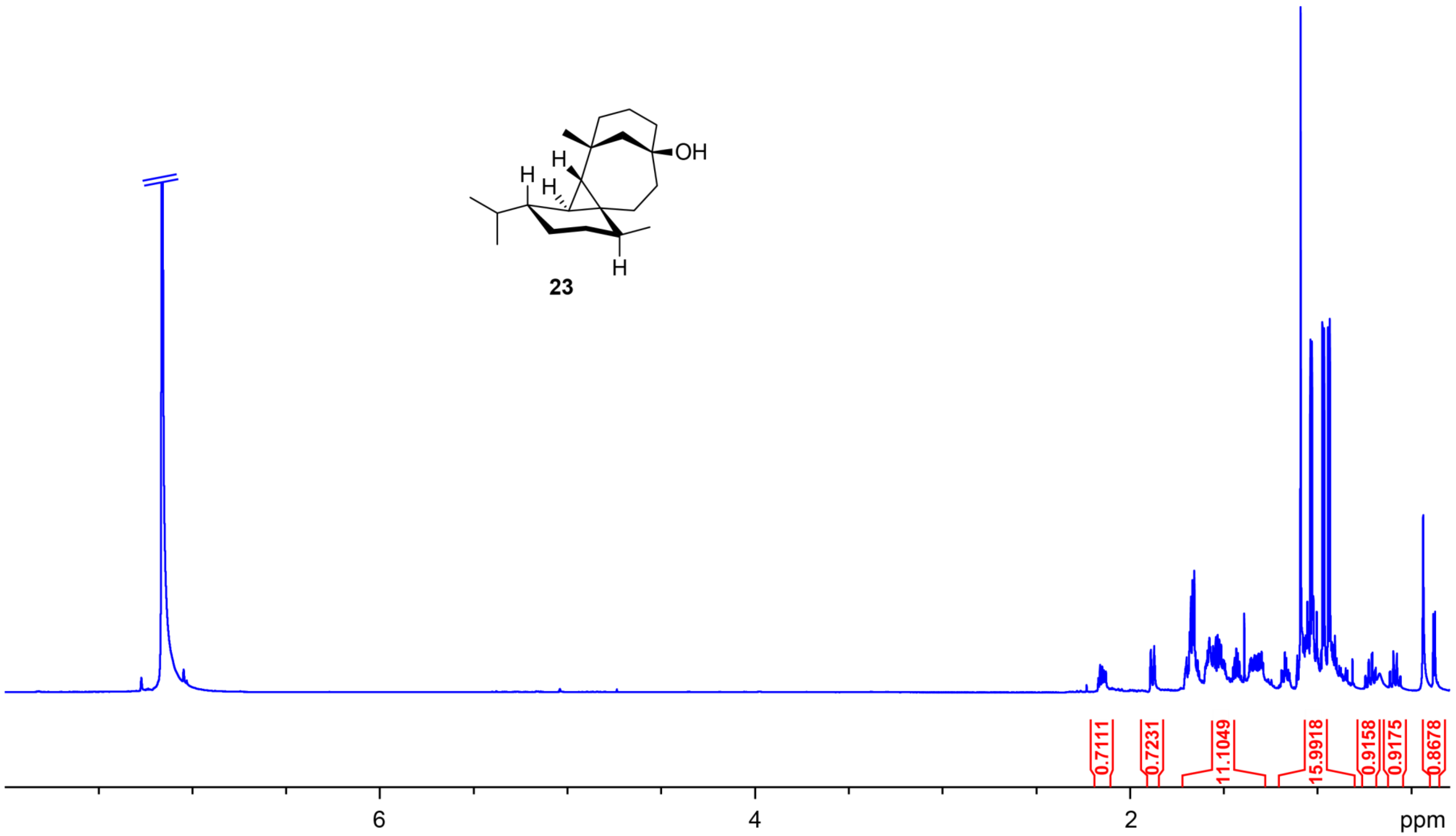

Figure S140. ^{1}H-NMR spectrum (700 MHz, C_6D_6) of **23**.

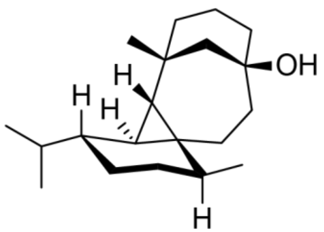

23

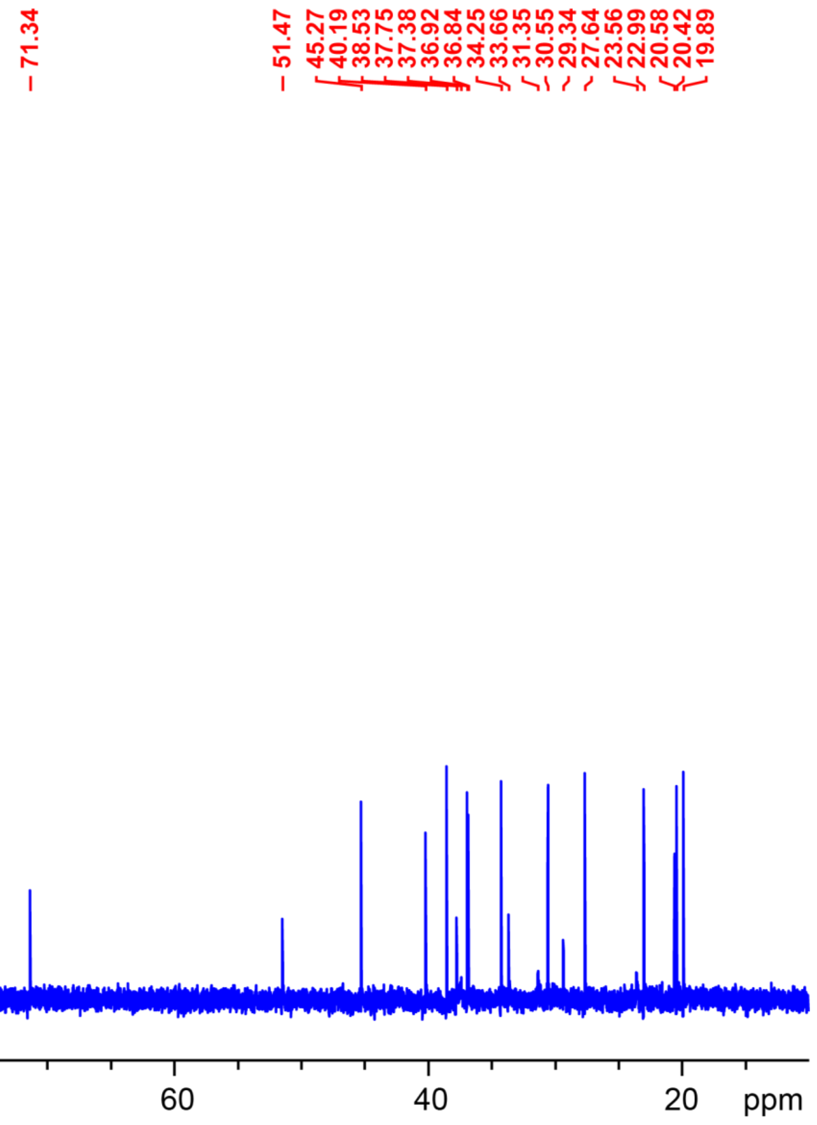

Figure S141. ^{13}C-NMR spectrum (176 MHz, C_6D_6) of **23**.

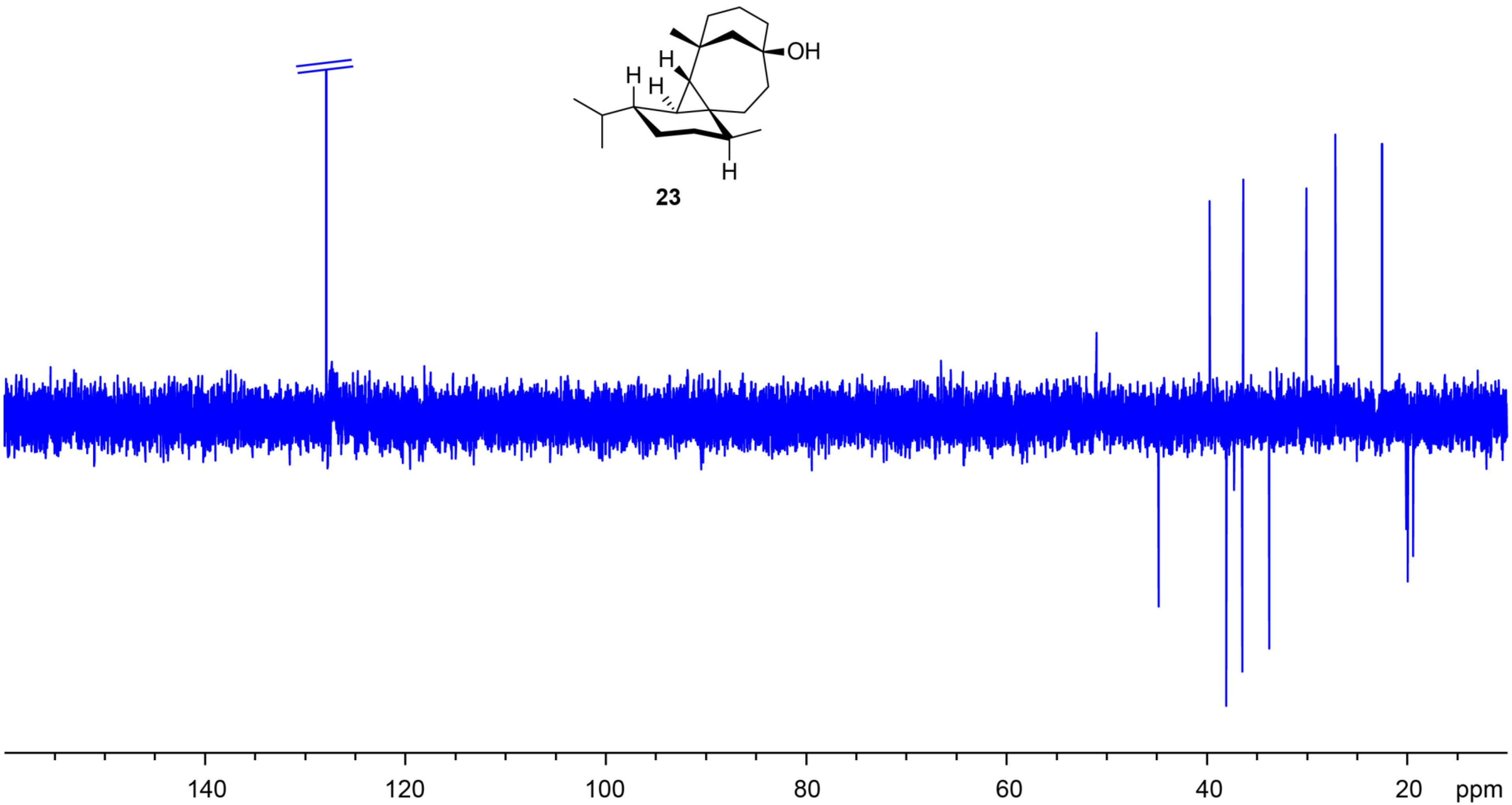

Figure S142. ^{13}C-DEPT135 spectrum (176 MHz, C_6D_6) of **23**.

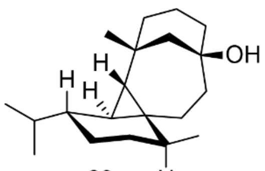

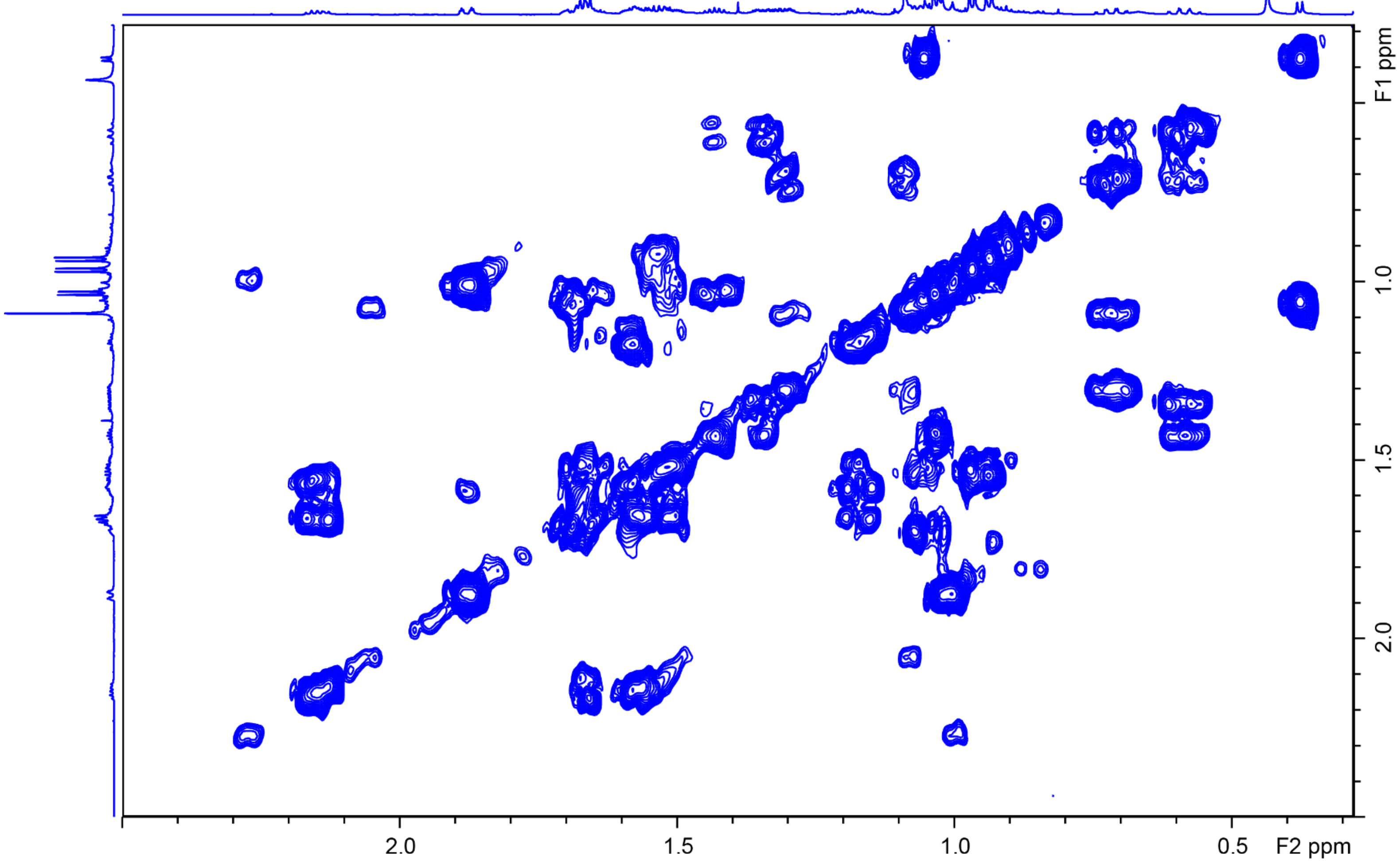

Figure S143. $^1H,^1H$-COSY spectrum (C_6D_6) of **23**.

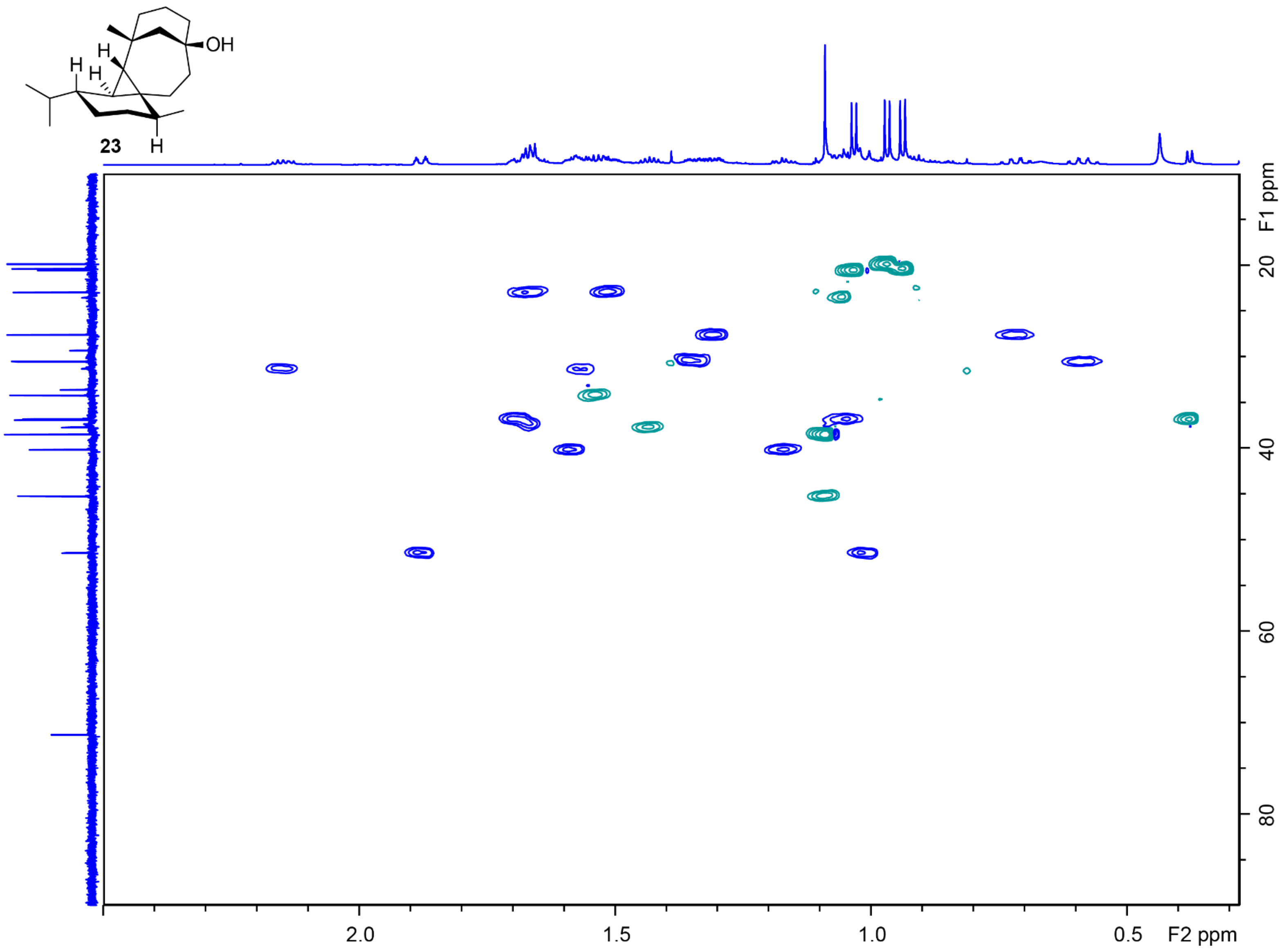

Figure S144. HSQC spectrum (C_6D_6) of **23**.

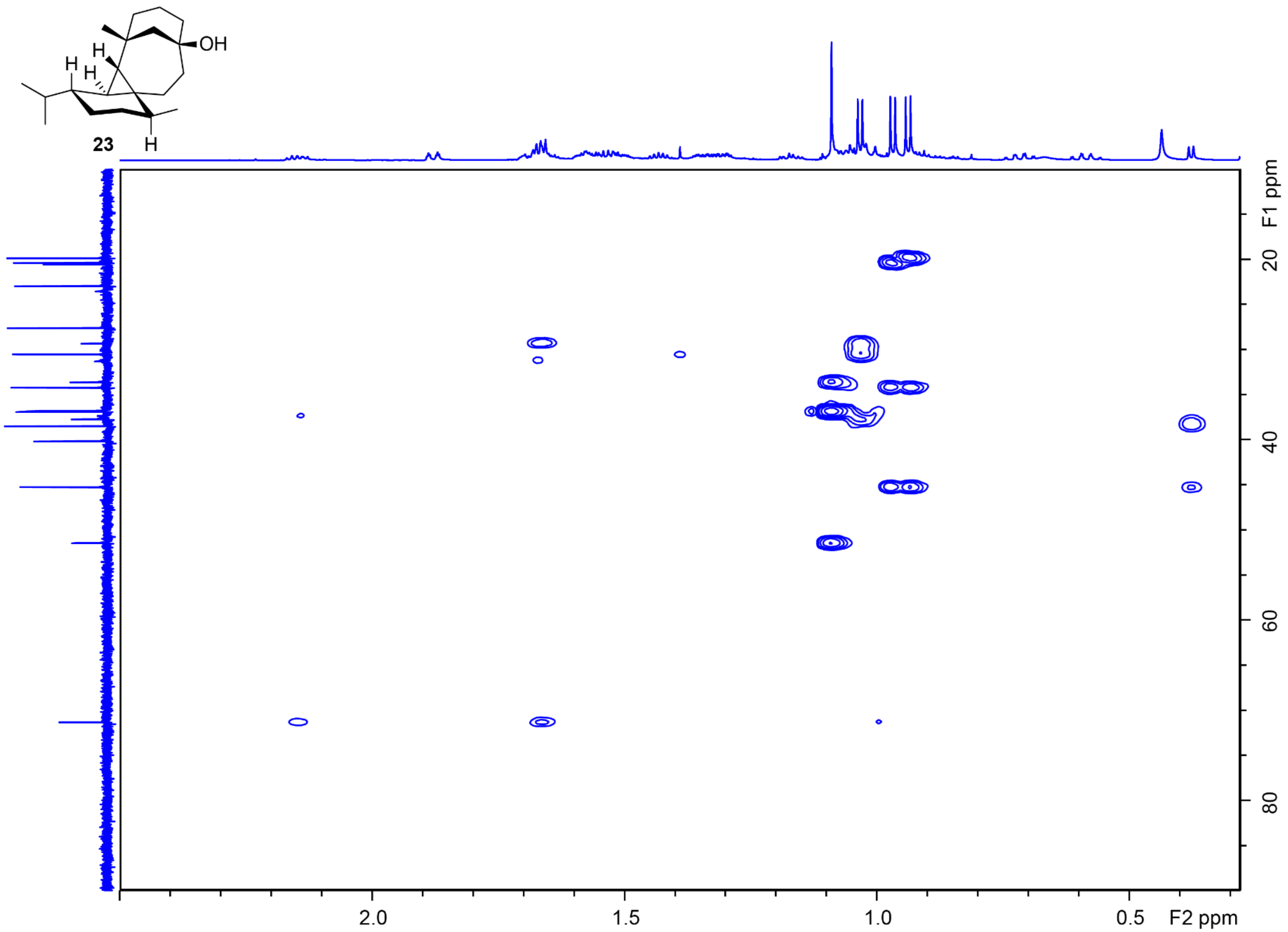

Figure S145. HMBC spectrum (C_6D_6) of **23**.

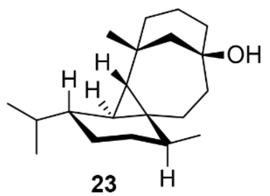

23

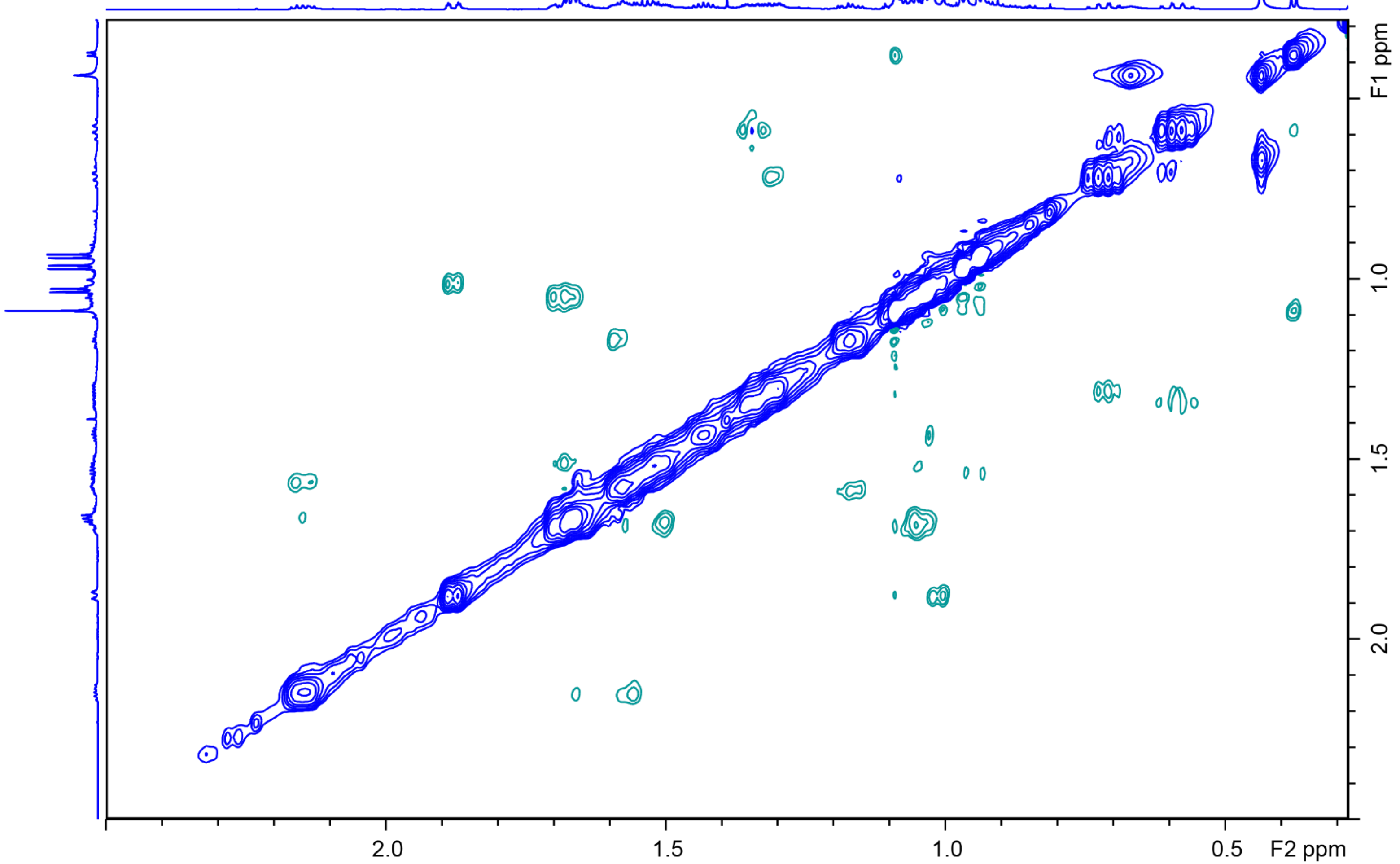

Figure S146. NOESY spectrum (C_6D_6) of **23**.

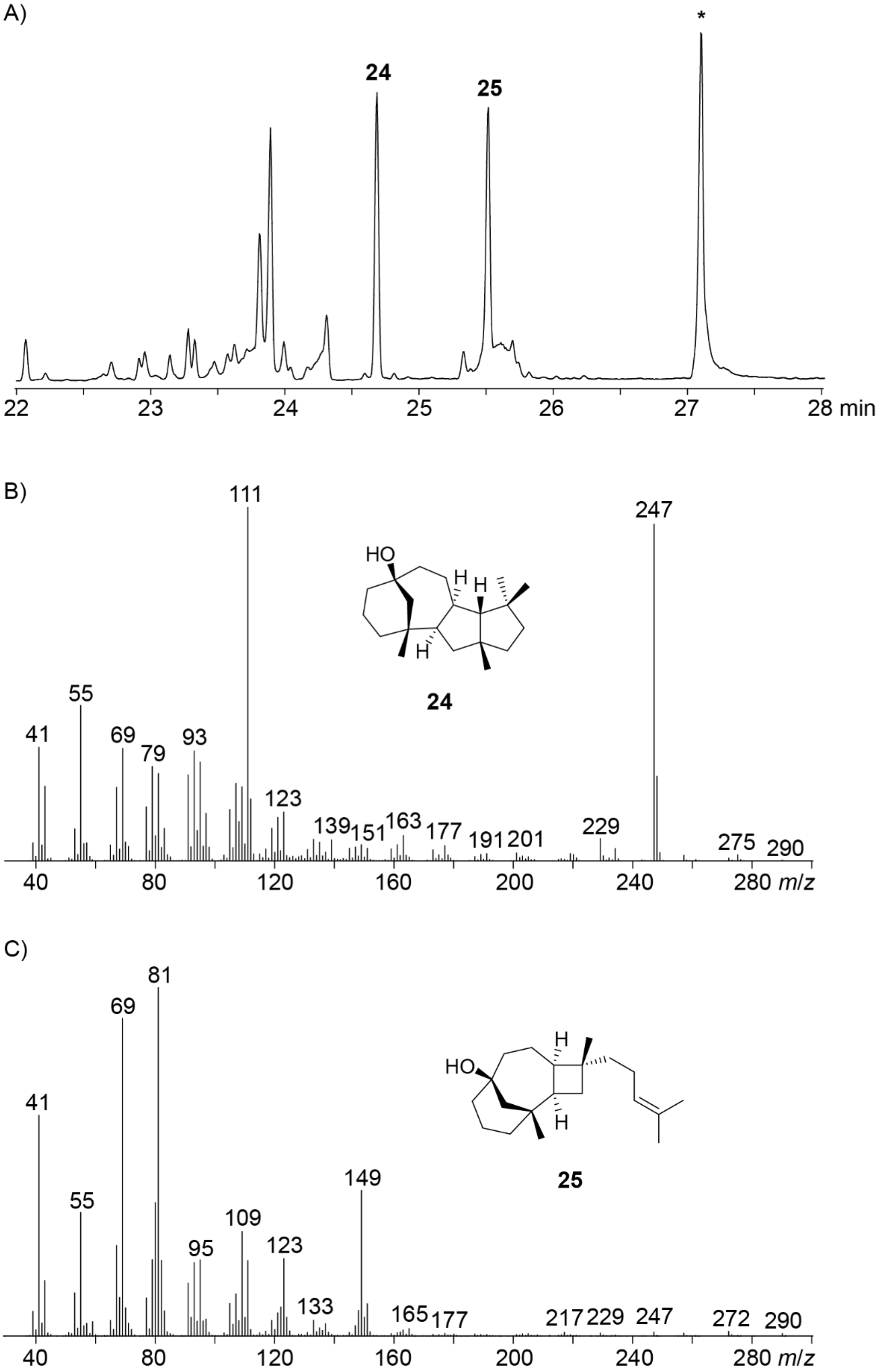

Figure S147. Product mixture formed from iso-GGPP I with AbVS. A) Total ion chromatogram of the crude extract from the enzyme incubation (asterisks indicate contaminants), B) EI mass spectrum of **24**, C) EI mass spectrum of **25**.

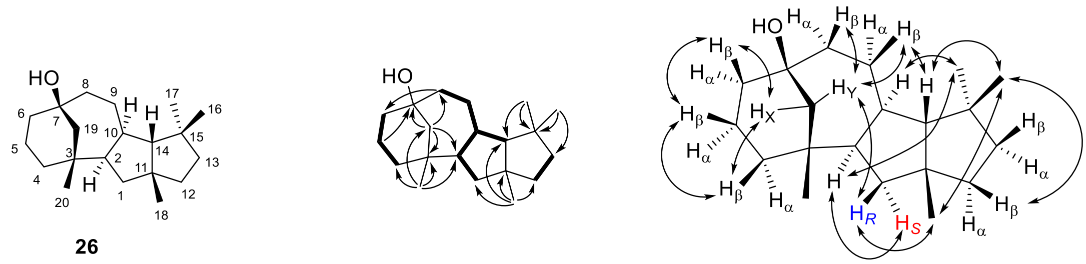

26

Figure S148. Structure elucidation of variexenol A (**24**). Bold: $^{1}H,^{1}H$-COSY, single-headed arrows: key HMBC, and double-headed arrows: key NOESY correlations.

Table S18. NMR data of variexenol A (**24**) in C_6D_6 recorded at 298 K.

C[a]	type	^{13}C[b]	^{1}H[b]
1	CH_2	43.39	1.51 (m, H_R)
			1.45 (m, H_S)
2	CH	53.42	1.65 (m)
3	C_q	36.01	–
4	CH_2	42.41	1.41 (m, H_{α})
			1.09 (m, H_{β})
5	CH_2	22.37	1.61 (m, H_{α})
			1.50 (m, H_{β})
6	CH_2	43.50	1.62 (m, H_{α})
			1.14 (m, H_{β})
7	C_q	71.89	–
8	CH_2	43.00	1.93 (m, H_{β})
			1.65 (m, H_{α})
9	CH_2	30.33	1.76 (m, H_{β})
			0.98 (m, H_{α})
10	CH	46.49	1.90 (m)
11	C_q	47.35	–
12	CH_2	41.67	1.53 (m, H_{β})
			1.47 (m, H_{α})
13	CH_2	42.36	1.39 (m, 2H)
14	CH	71.71	1.19 (s)
15	C_q	42.85	–
16	CH_3	32.82	1.00 (s)
17	CH_3	24.79	0.94 (s)
18	CH_3	32.09	1.19 (s)
19	CH_2	43.77	2.06 (d, ^{2}J = 13.5, H_Y)
			0.74 (d, ^{2}J = 13.5, H_X)
20	CH_3	34.39	0.80 (s)

[a] Carbon numbering as shown in Figure S148. [b] Chemical shifts δ in ppm, multiplicity: s = singlet, d = doublet, m = multiplet, coupling constants J are given in Hertz.

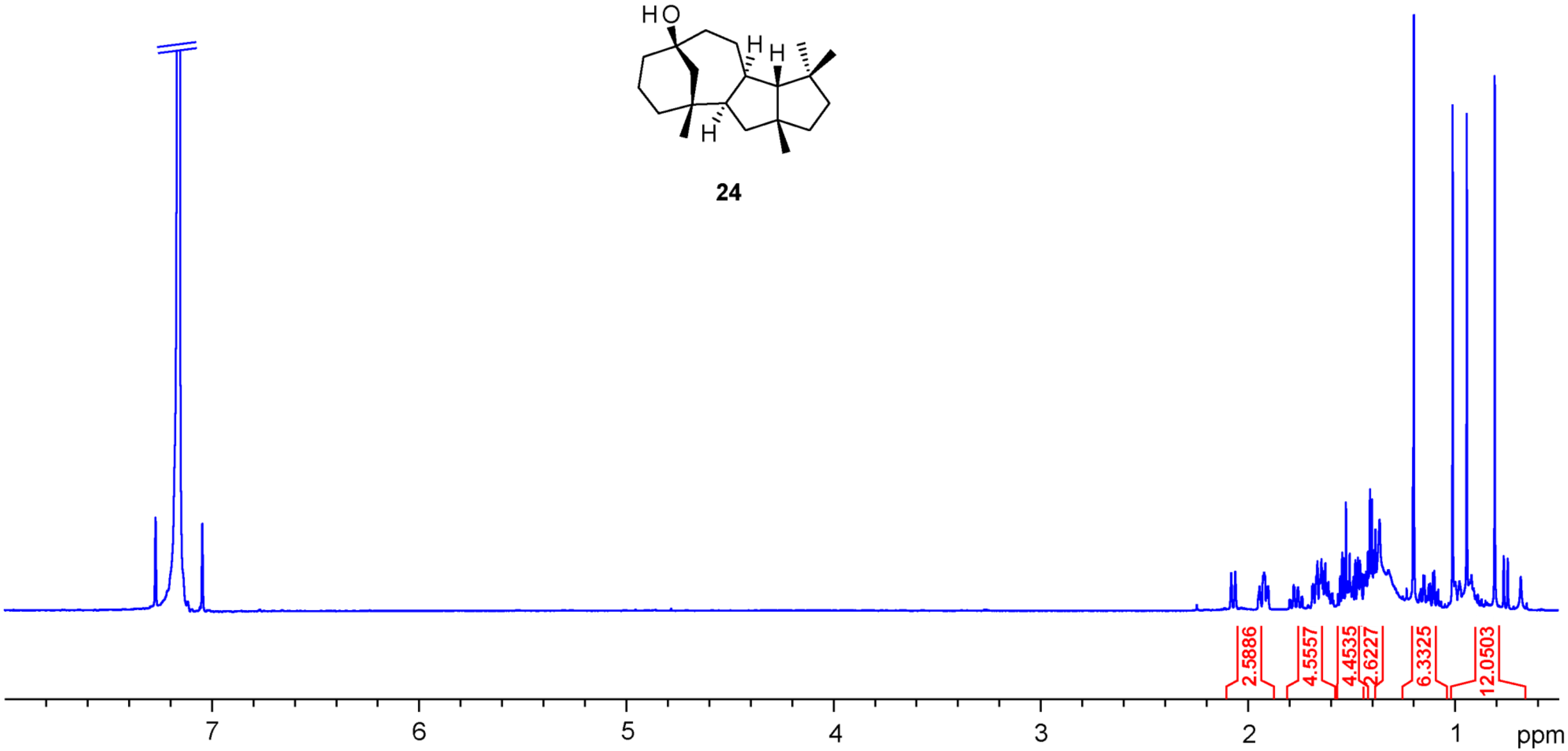

Figure S149. ^{1}H-NMR spectrum (700 MHz, C_6D_6) of **24**.

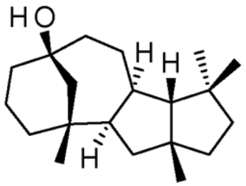

24

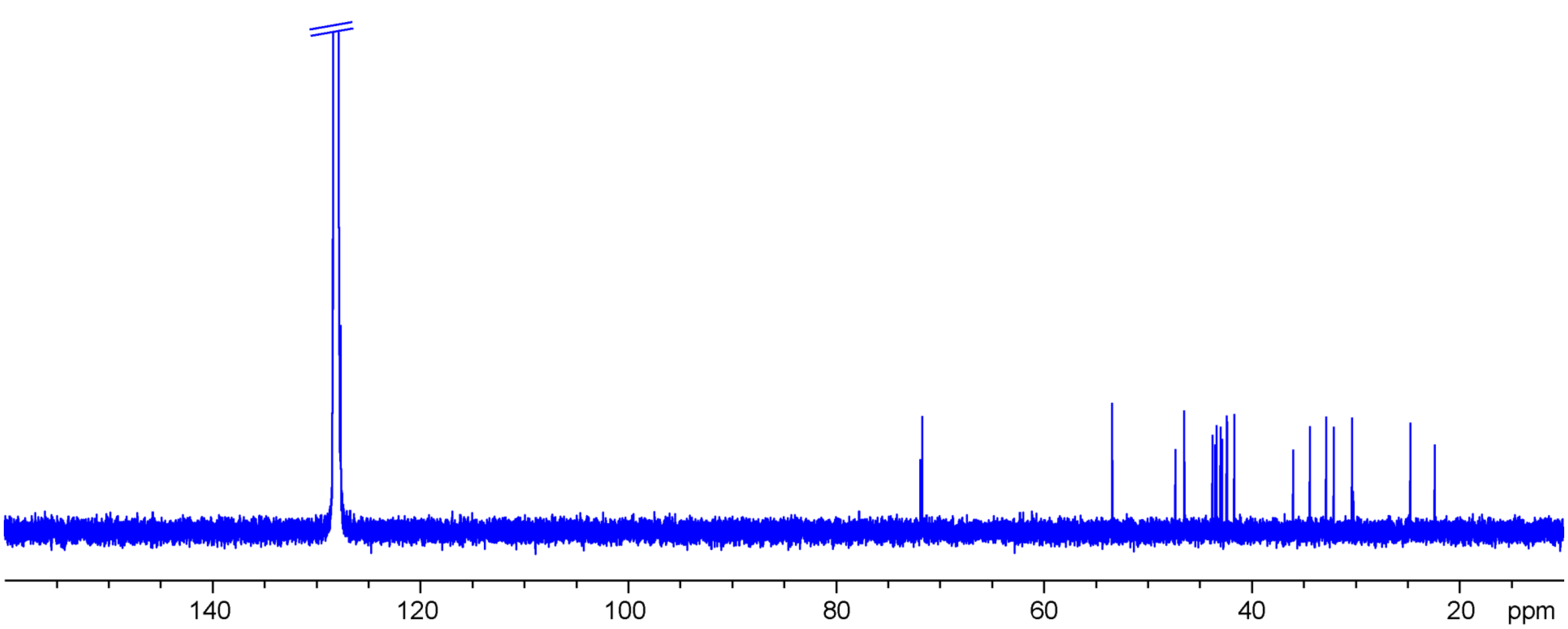

Figure S150. ^{13}C-NMR spectrum (176 MHz, C_6D_6) of **24**.

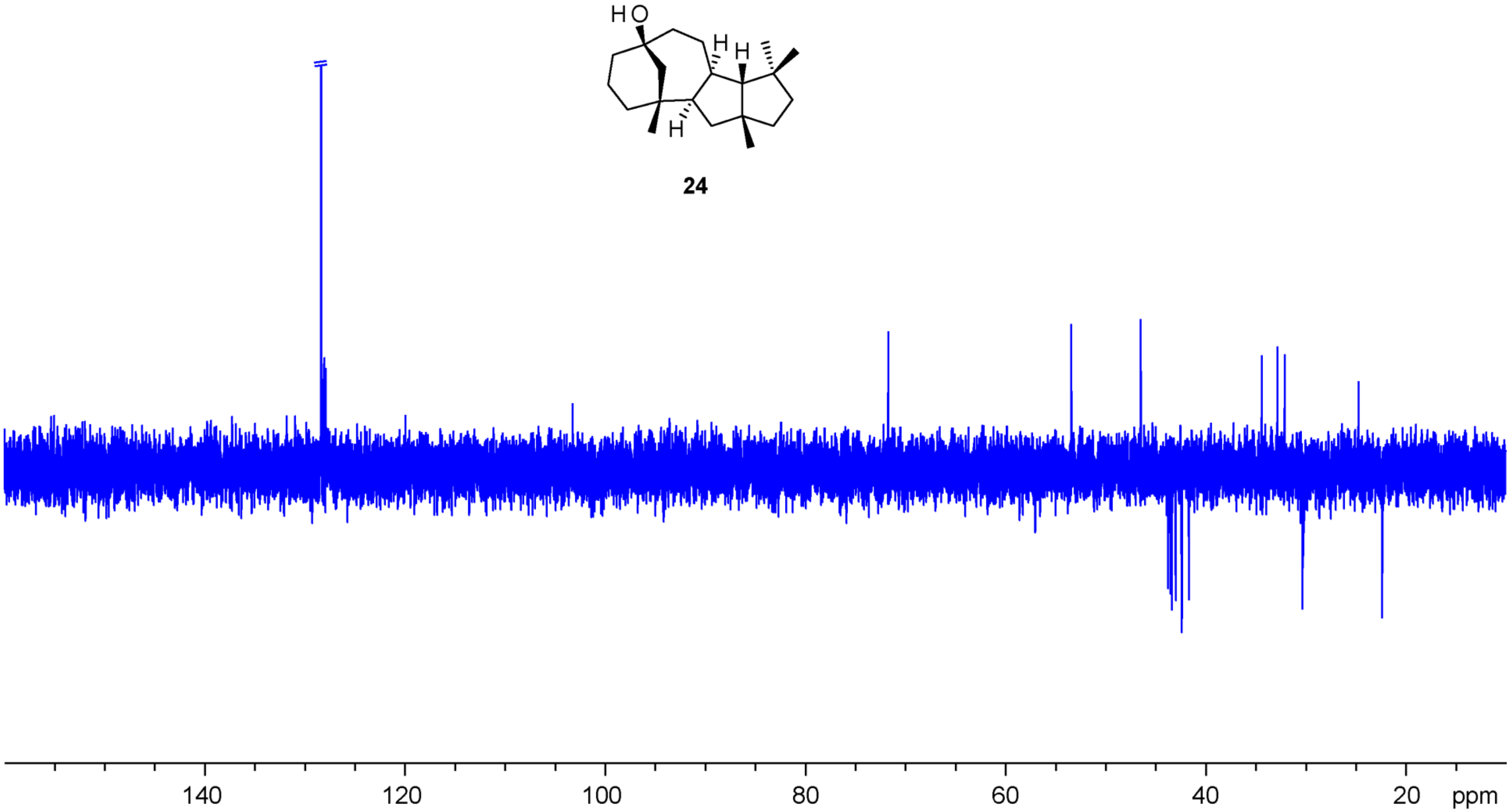

Figure S151. ^{13}C-DEPT135 spectrum (176 MHz, C_6D_6) of **24**.

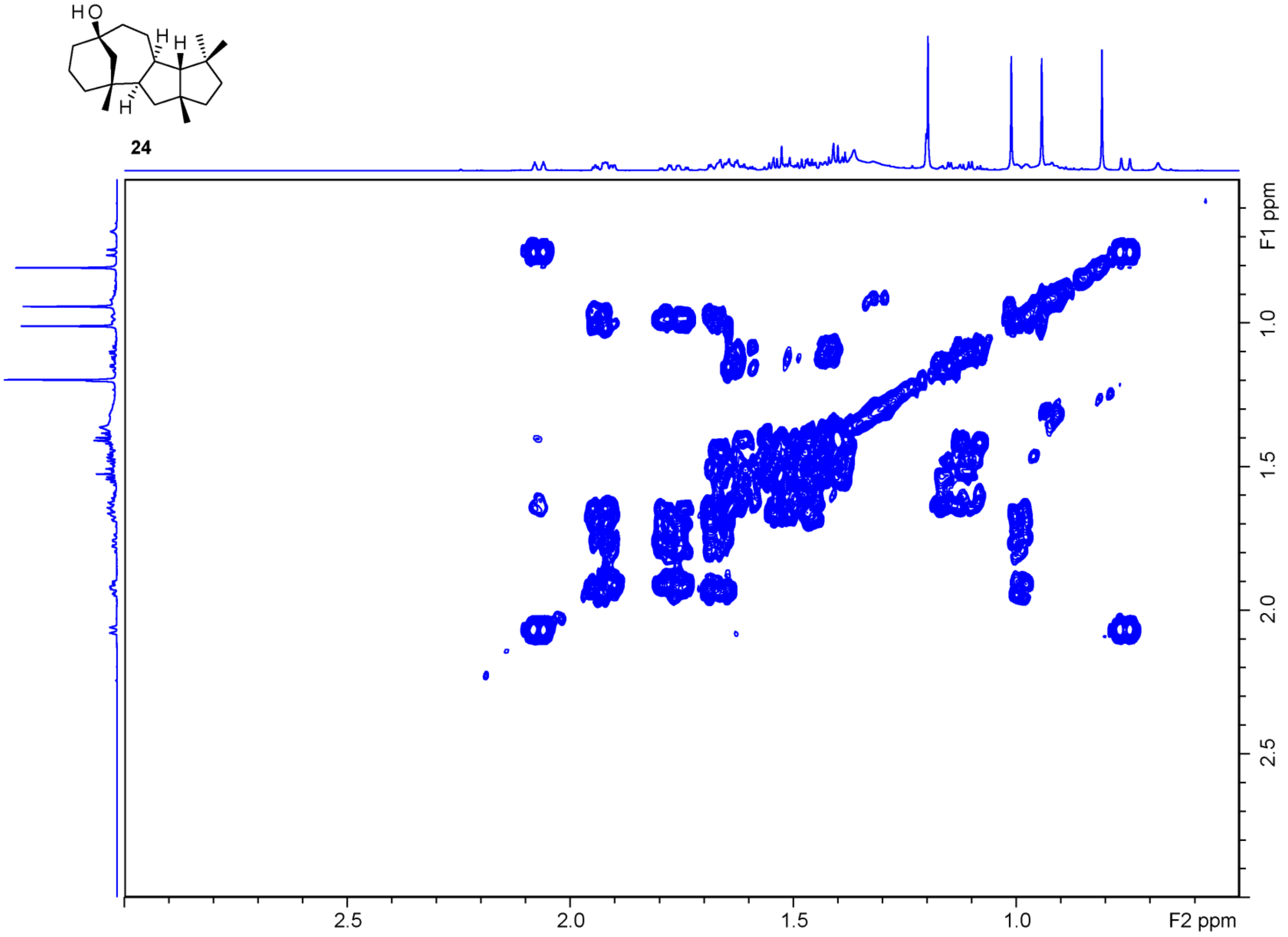

Figure S152. $^{1}H,^{1}H$-COSY spectrum (C_6D_6) of **24**.

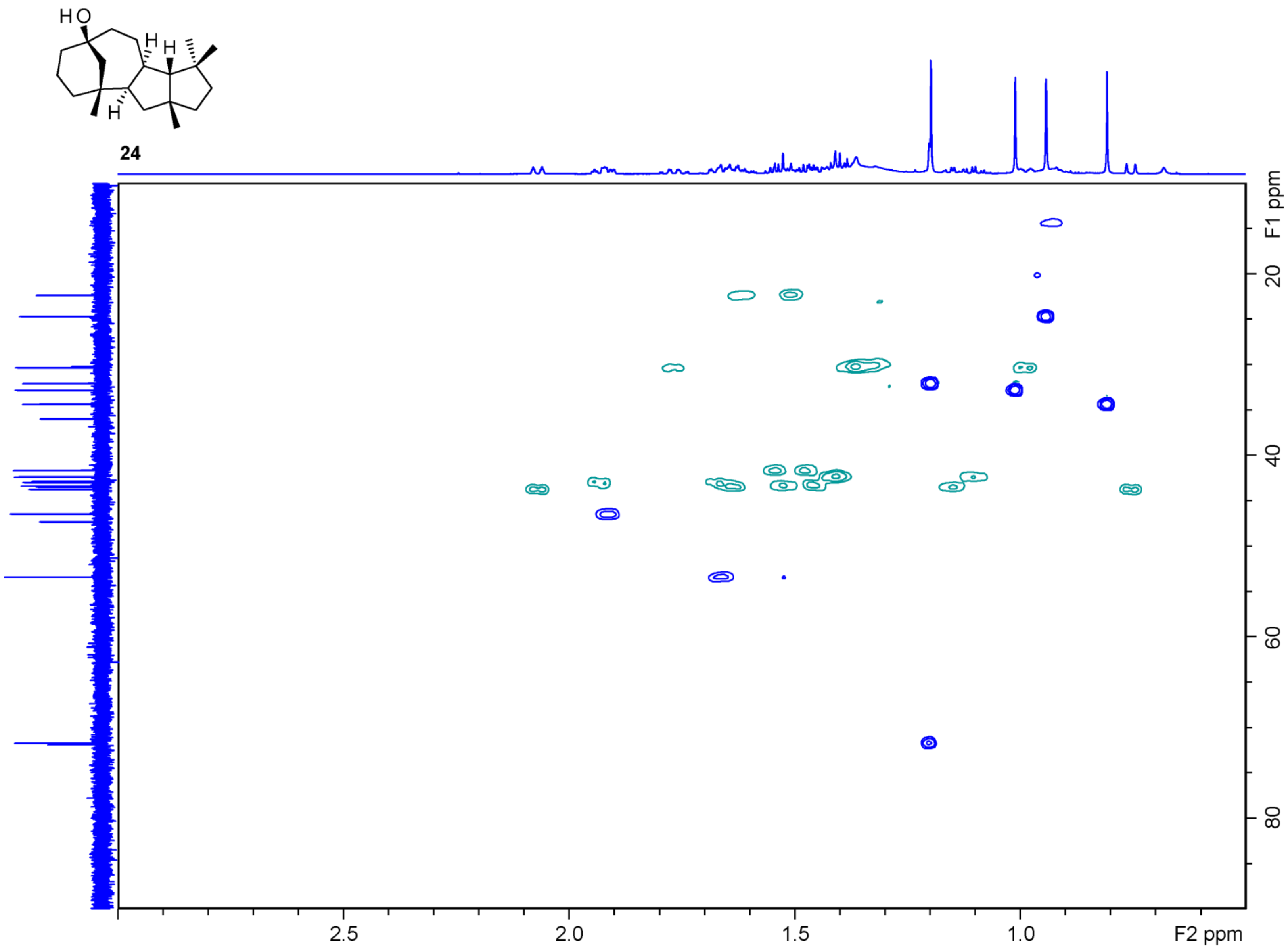

Figure S153. HSQC spectrum (C_6D_6) of **24**.

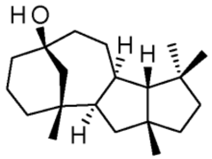

24

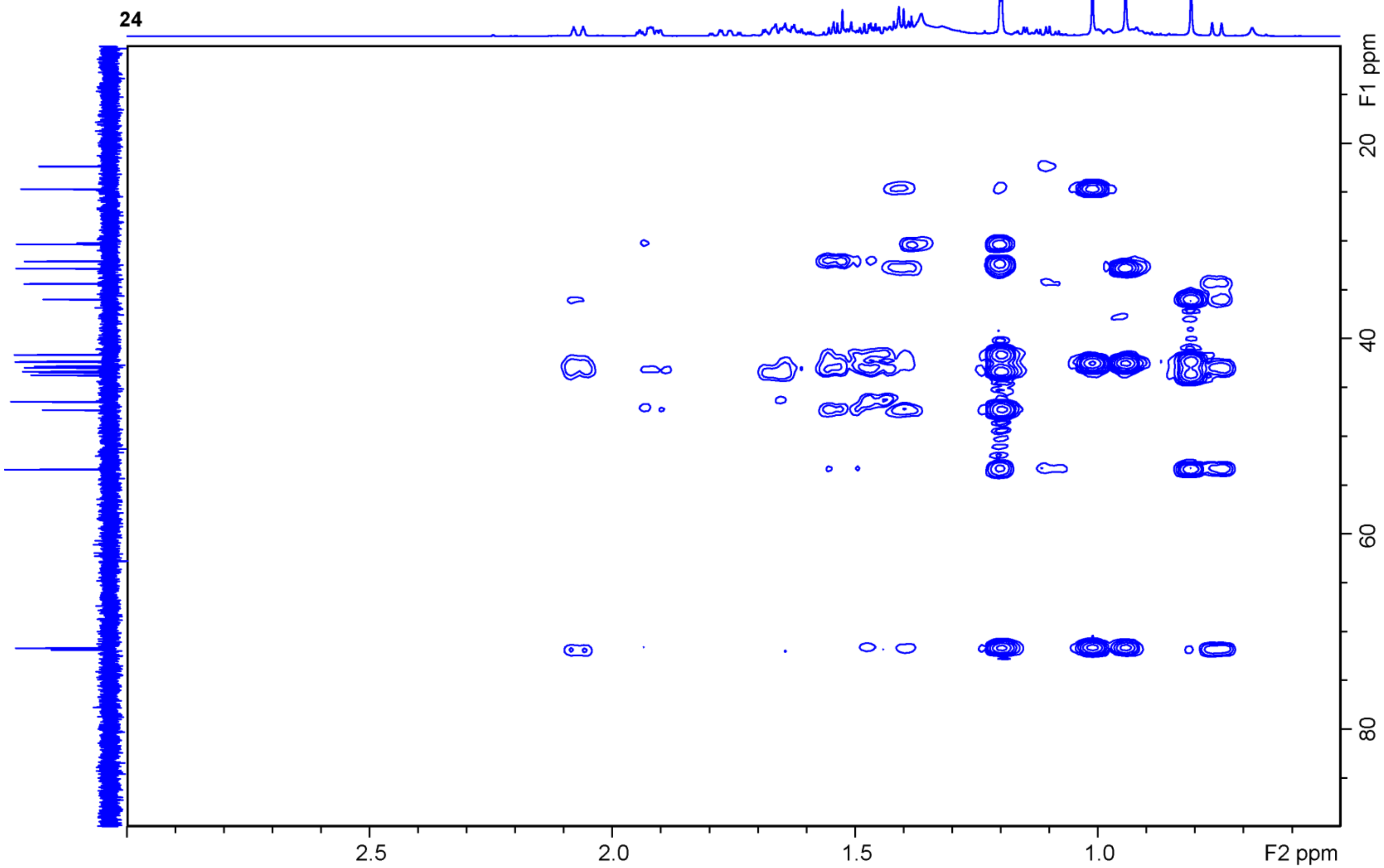

Figure S154. HMBC spectrum (C_6D_6) of **24**.

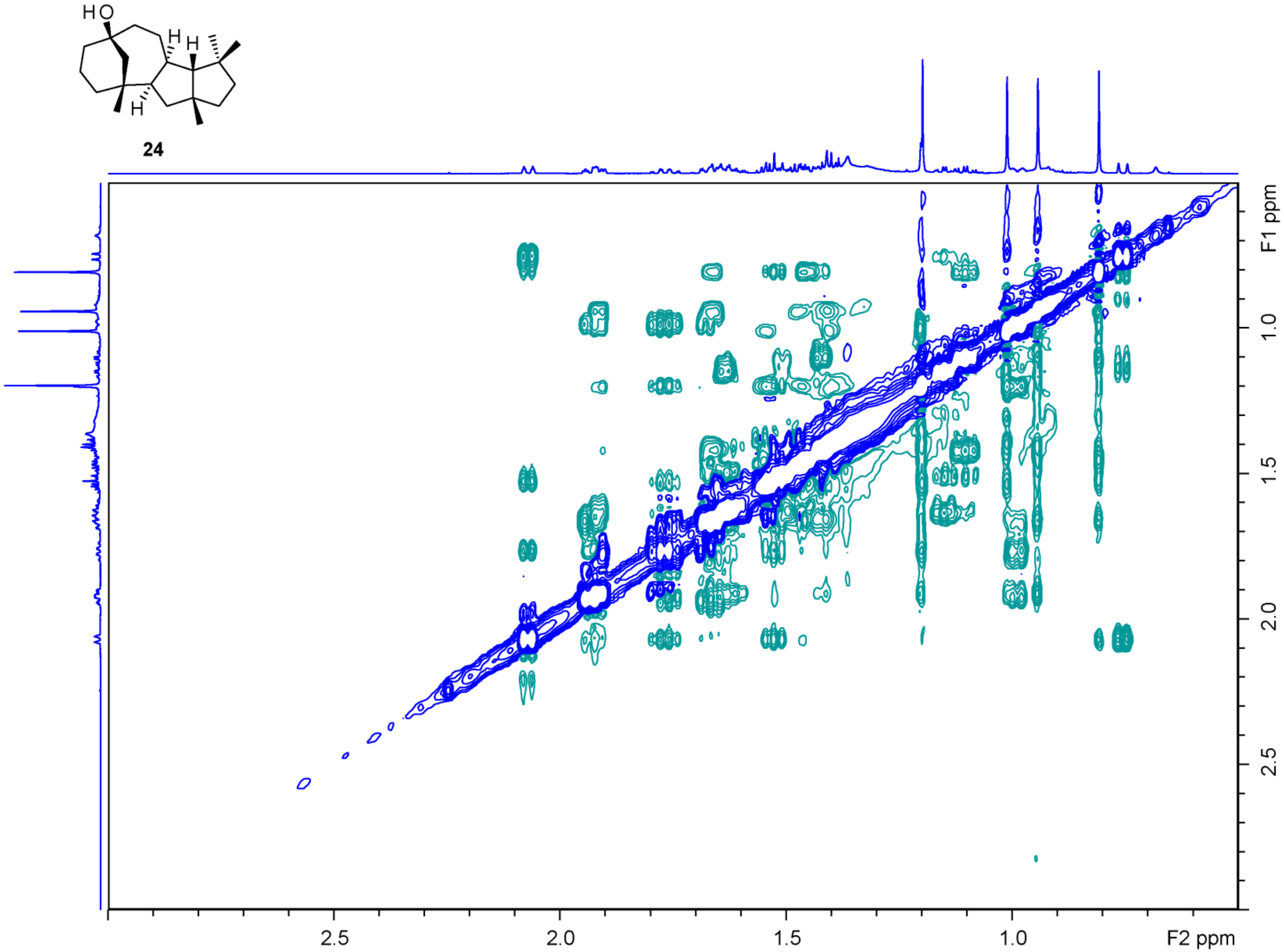

Figure S155. NOESY spectrum (C_6D_6) of **24**.

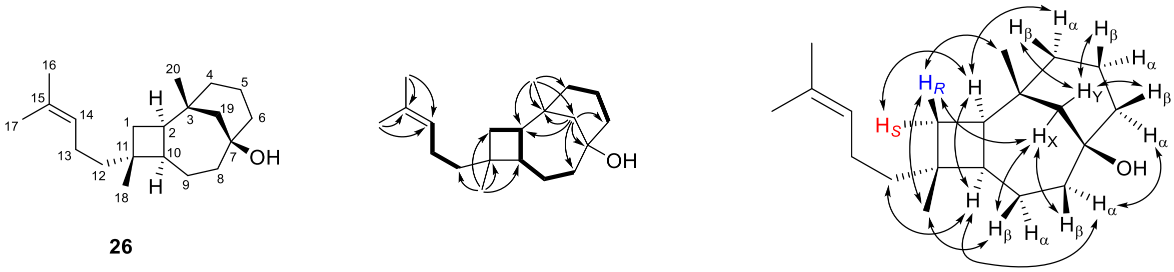

Figure S156. Structure elucidation of variexenol B (**25**). Bold: ^{1}H,^{1}H-COSY, single-headed arrows: key HMBC, and double-headed arrows: key NOESY correlations.

Table S19. NMR data of variexenol B (**25**) in C_6D_6 recorded at 298 K.

C[a]	type	^{13}C[b]	^{1}H[b]
1	CH_2	35.10	1.59 (m, H_S)
			1.51 (m, H_R)
2	CH	42.11	2.03 (m)
3	C_q	36.06	–
4	CH_2	42.81	1.25 (m, H_α)
			1.02 (m, H_β)
5	CH_2	22.17	1.46 (m, H_β)
			1.29 (m, H_α)
6	CH_2	42.91	1.65 (m, H_α)
			1.15 (m, H_β)
7	C_q	71.97	–
8	CH_2	40.50	1.94 (ddd, ^2J = 15.1, ^3J = 7.2, 2.0, H_β)
			1.51 (m, H_α)
9	CH_2	23.20	1.74 (m, H_β)
			1.32 (m, H_α)
10	CH	48.57	1.83 (m)
11	C_q	37.02	–
12	CH_2	41.58	1.55 (m, 2H)
13	CH_2	24.29	2.00 (m, 2H)
14	CH	125.84	5.26 (m)
15	C_q	130.78	–
16	CH_3	25.90	1.70 (br s)
17	CH_3	17.69	1.59 (br s)
18	CH_3	21.46	0.86 (s)
19	CH_2	45.12	2.08 (d, ^2J = 13.3, H_X)
			0.78 (d, ^2J = 13.3, H_Y)
20	CH_3	29.54	0.64 (s)

[a] Carbon numbering as shown in Figure S156. [b] Chemical shifts δ in ppm, multiplicity: s = singlet, d = doublet, m = multiplet, coupling constants J are given in Hertz.

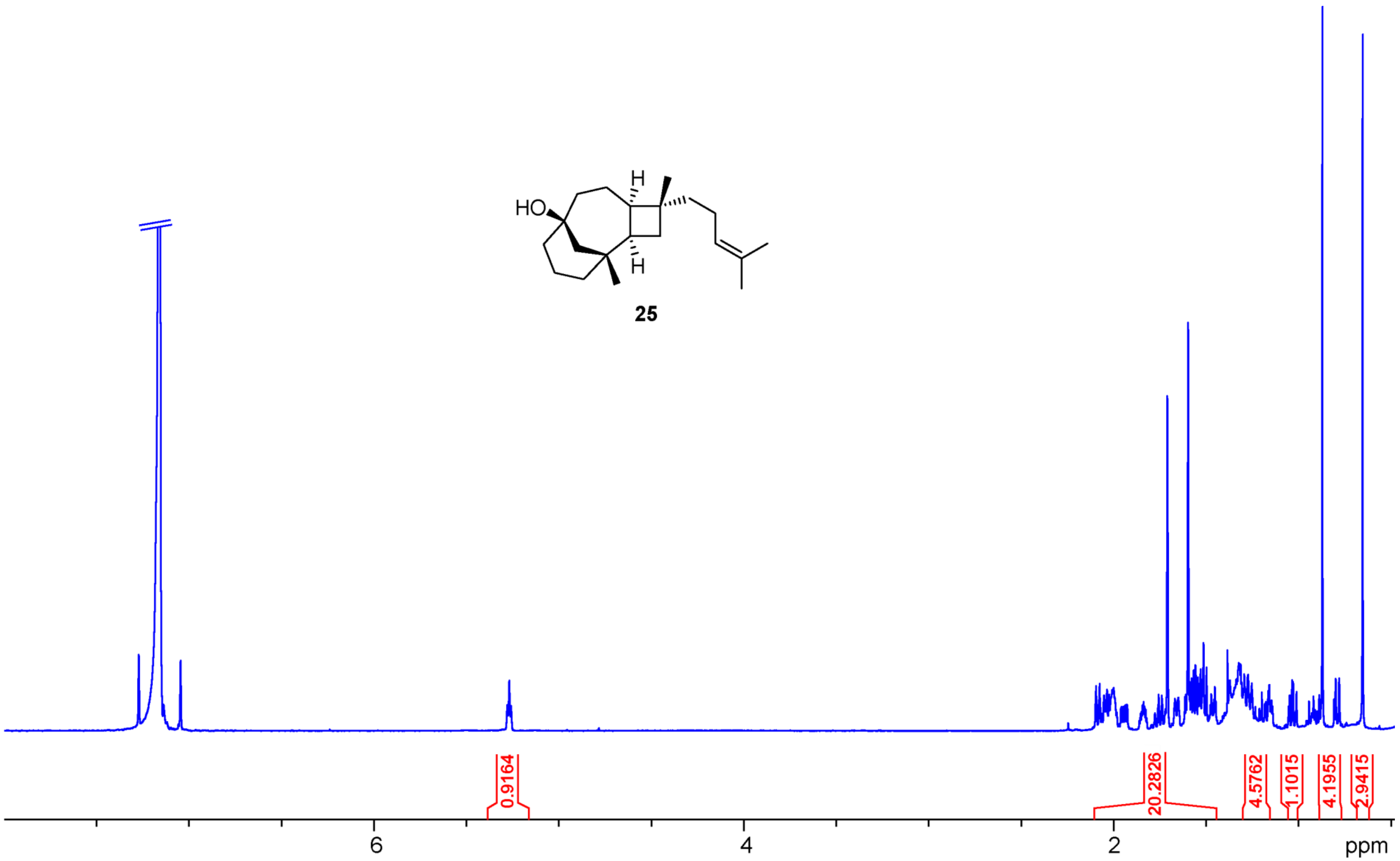

Figure S157. ^{1}H-NMR spectrum (700 MHz, C_6D_6) of **25**.

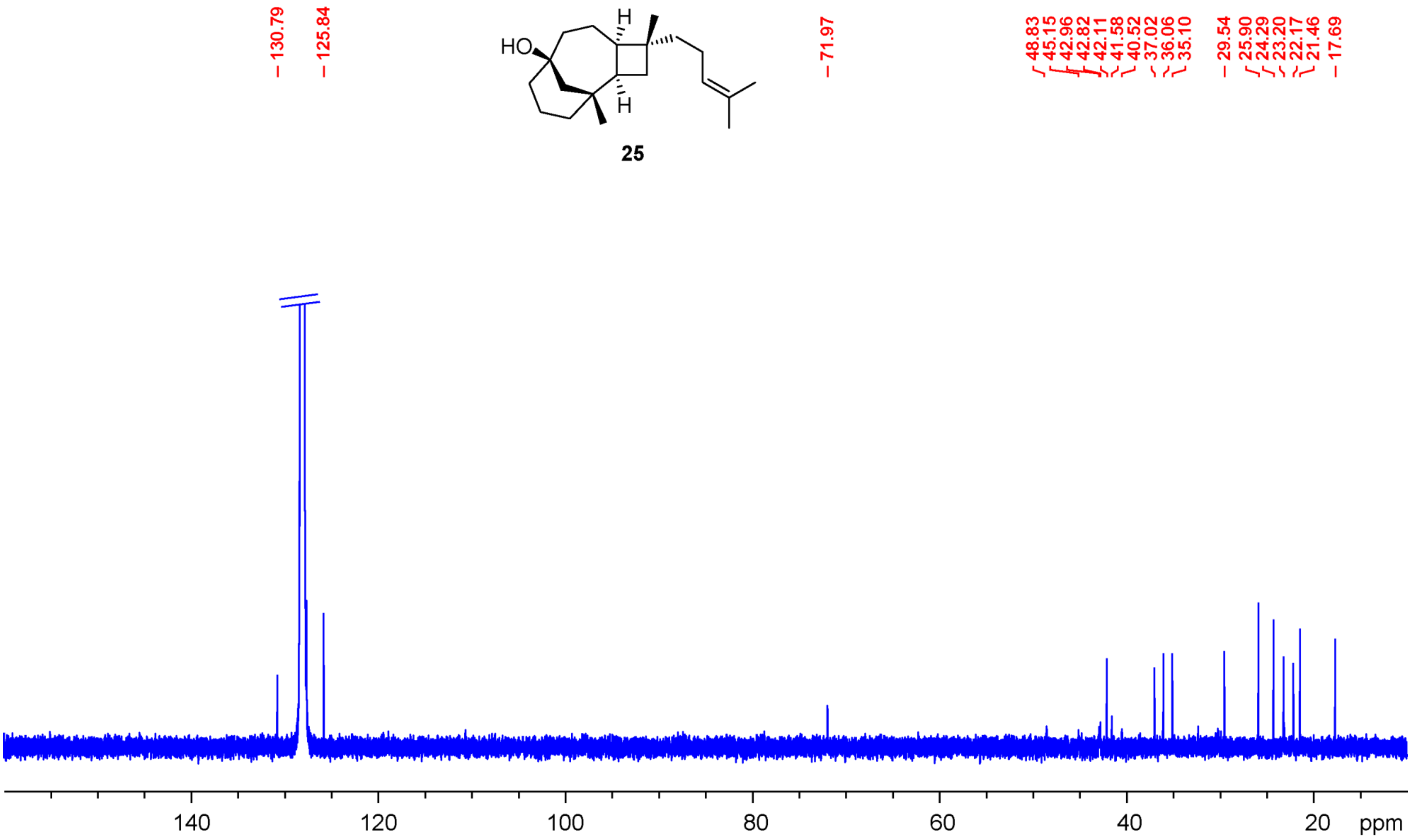

Figure S158. ^{13}C-NMR spectrum (176 MHz, C_6D_6) of **25**.

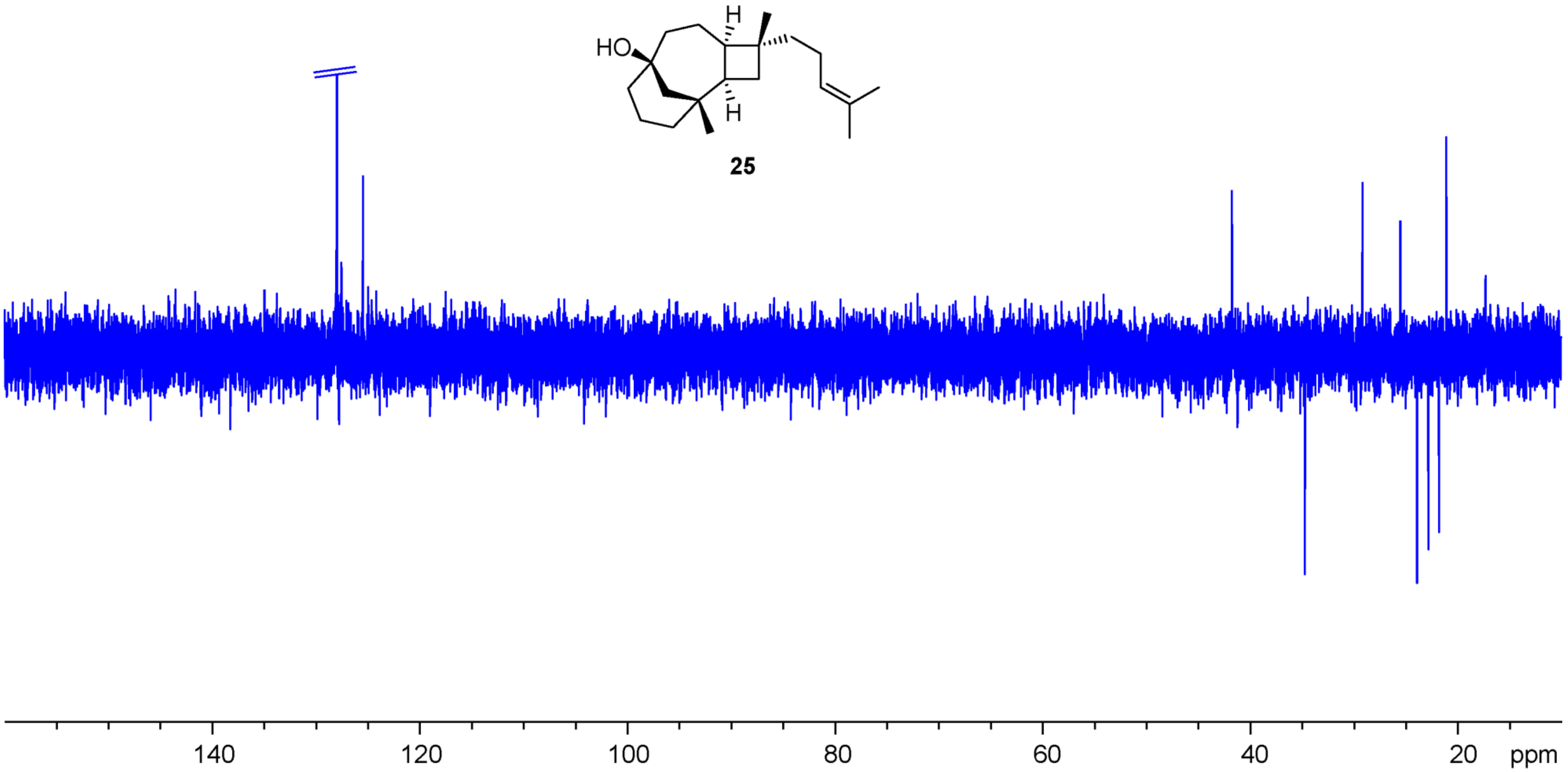

Figure S159. ^{13}C-DEPT135 spectrum (176 MHz, C_6D_6) of **25**.

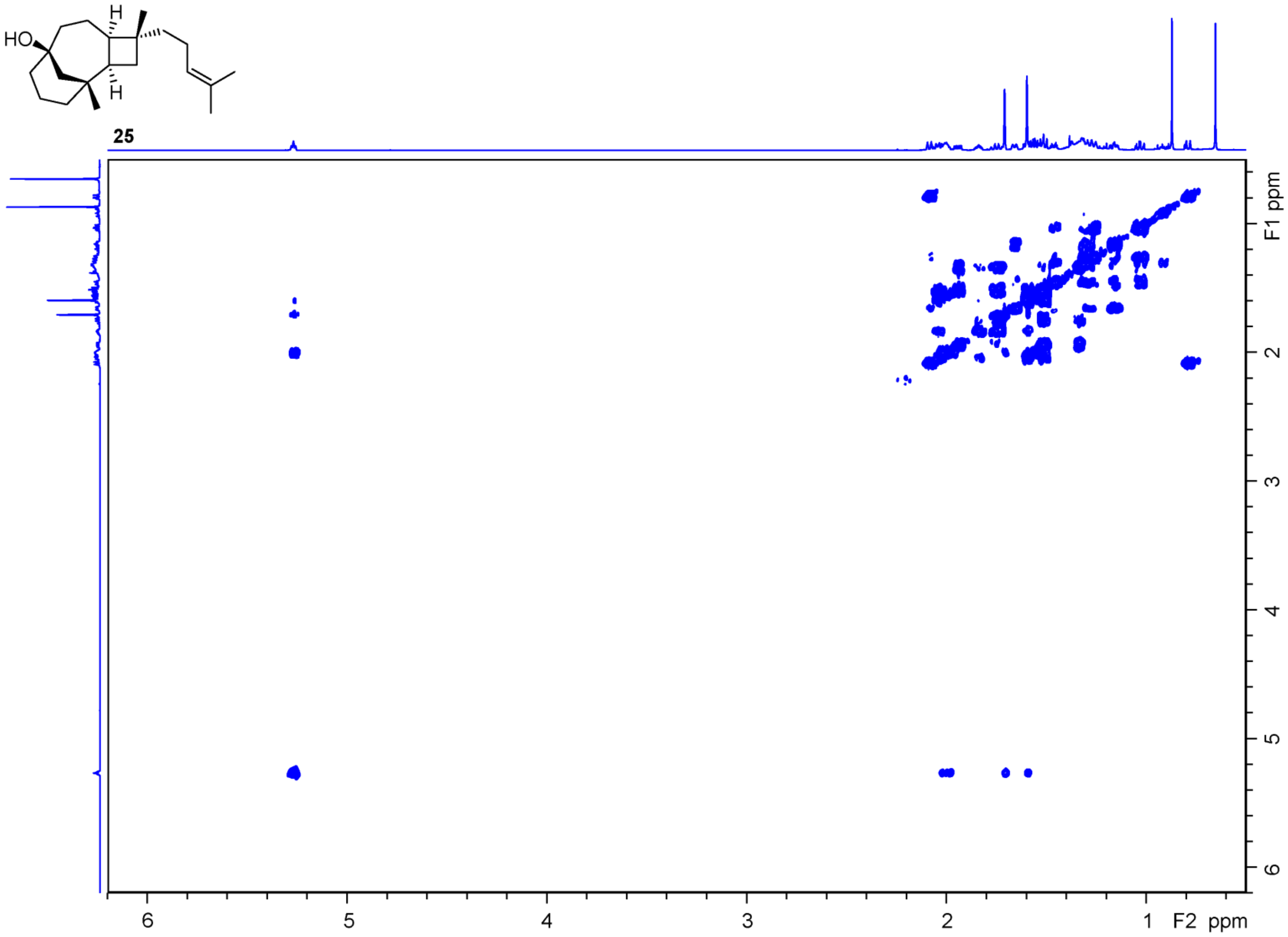

Figure S160. $^{1}H,^{1}H$-COSY spectrum (C_6D_6) of **25**.

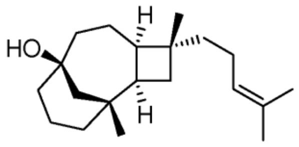

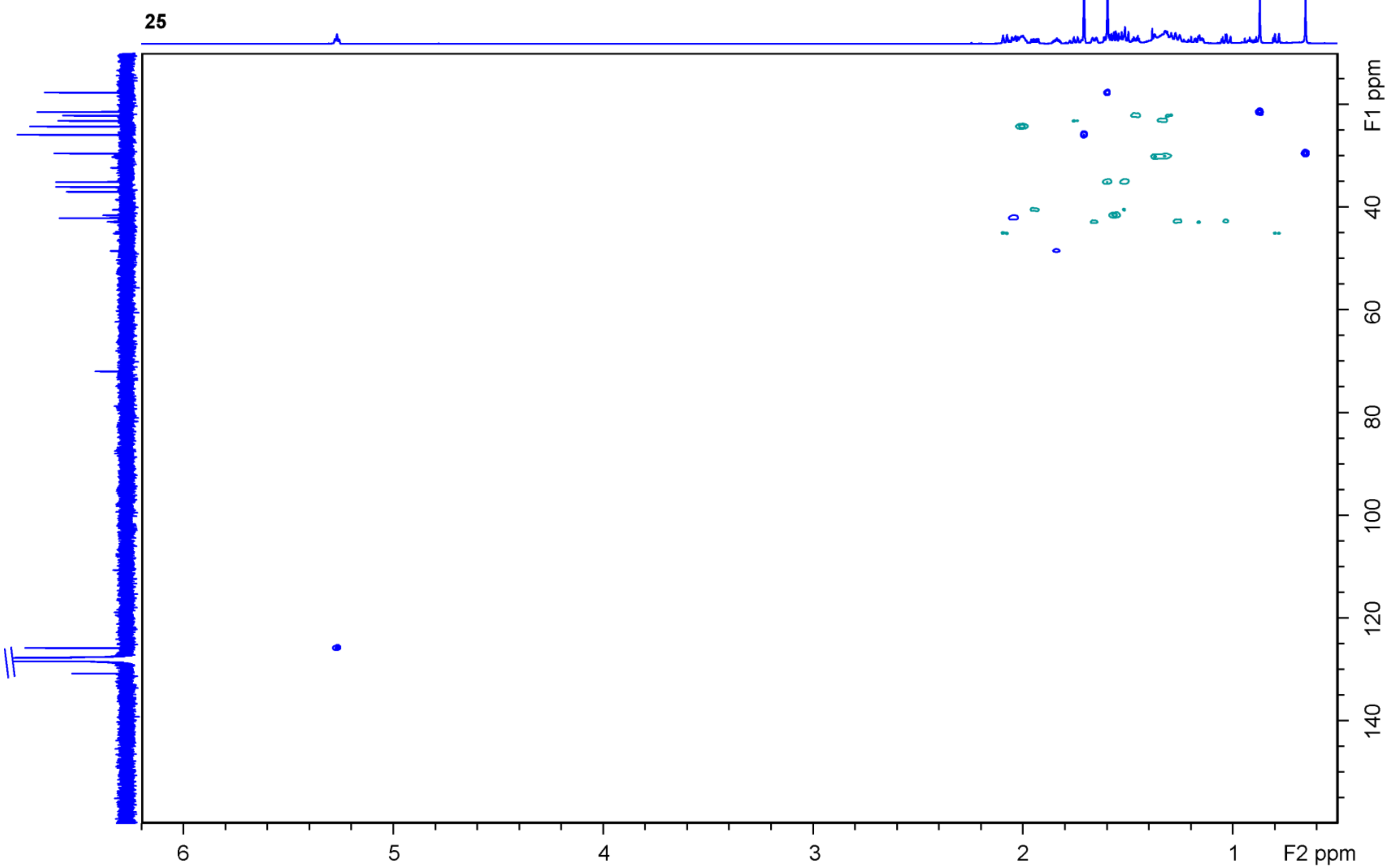

Figure S161. HSQC spectrum (C_6D_6) of **25**.

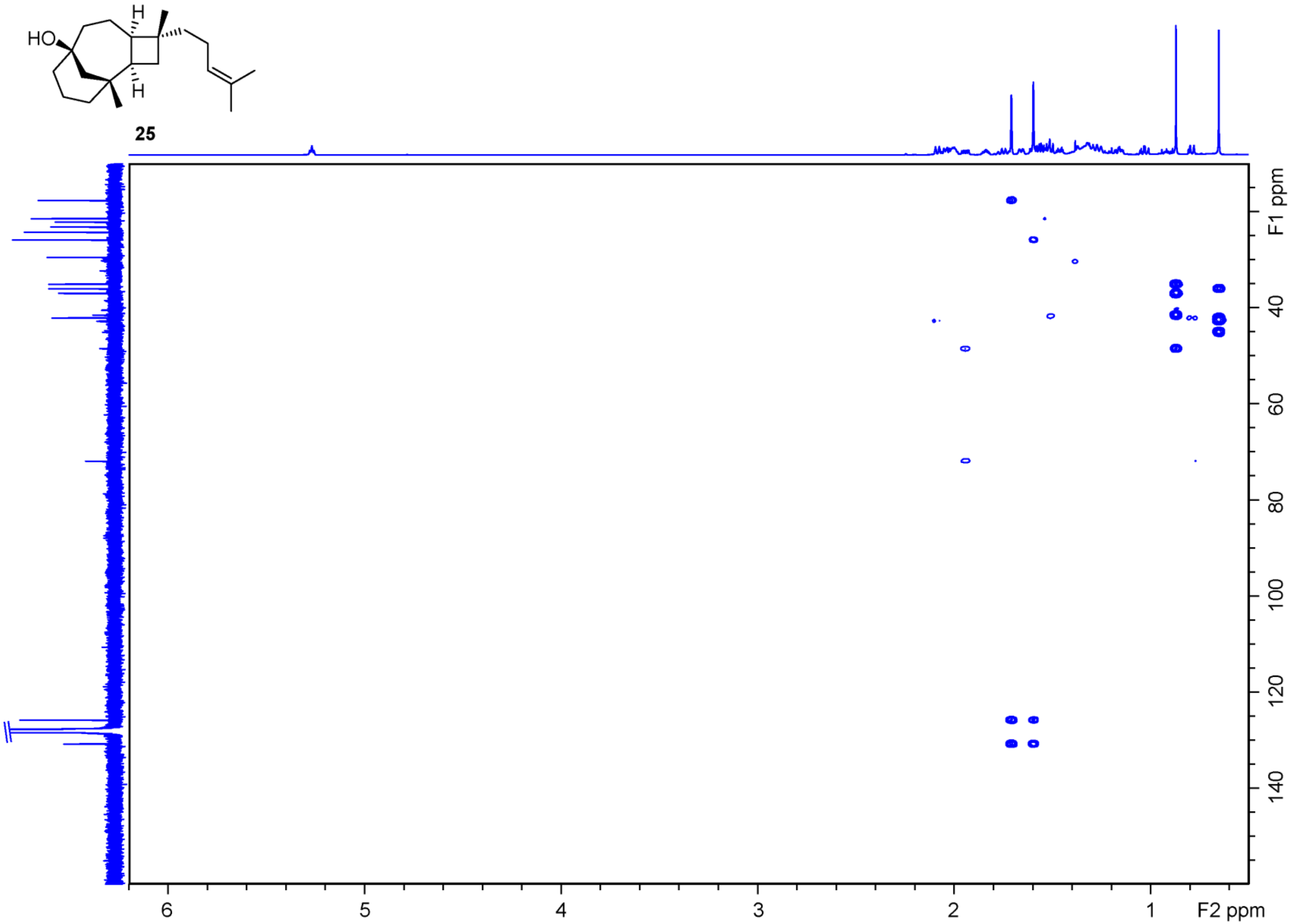

Figure S162. HMBC spectrum (C_6D_6) of **25**.

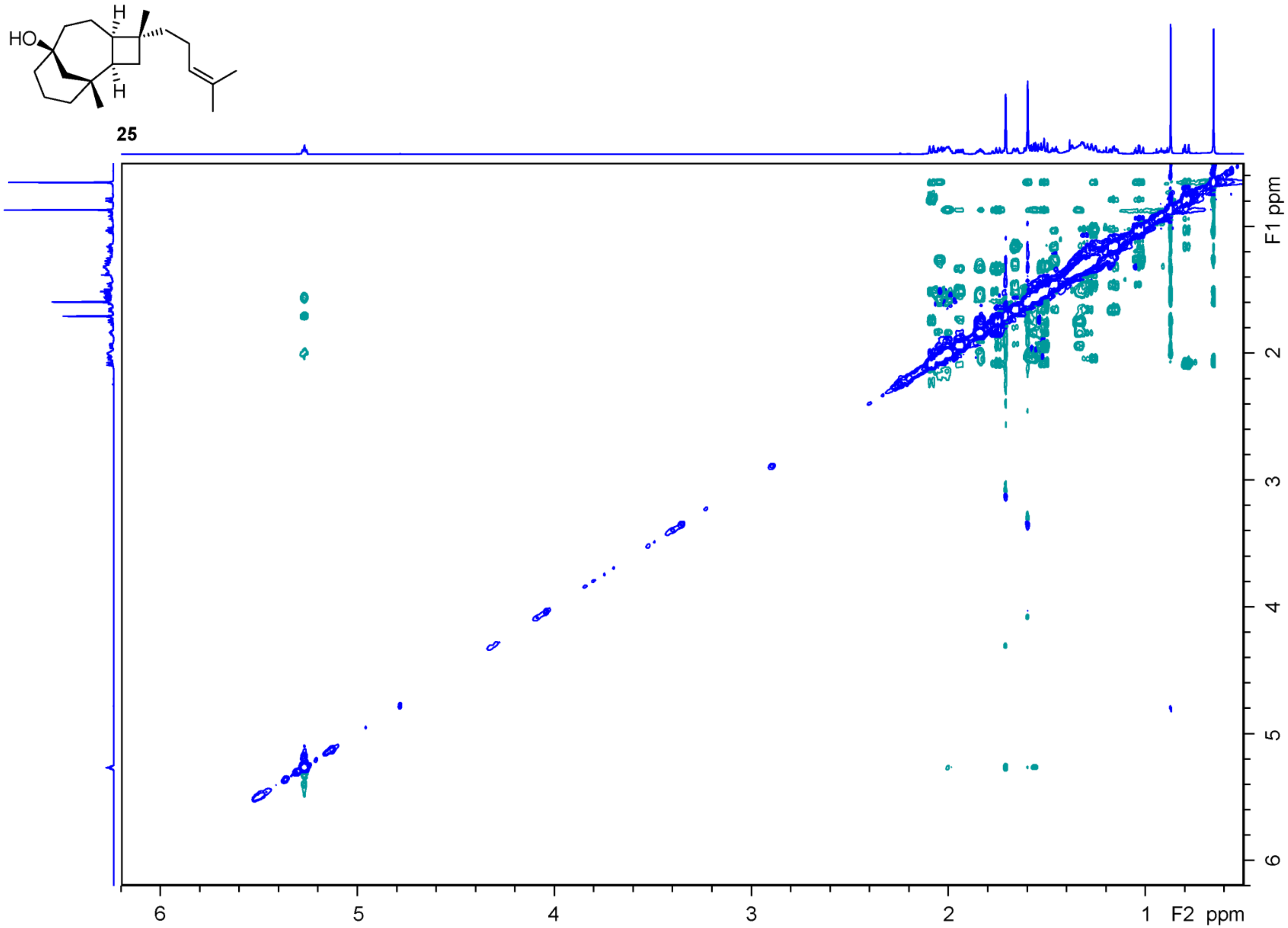

Figure S163. NOESY spectrum (C_6D_6) of **25**.

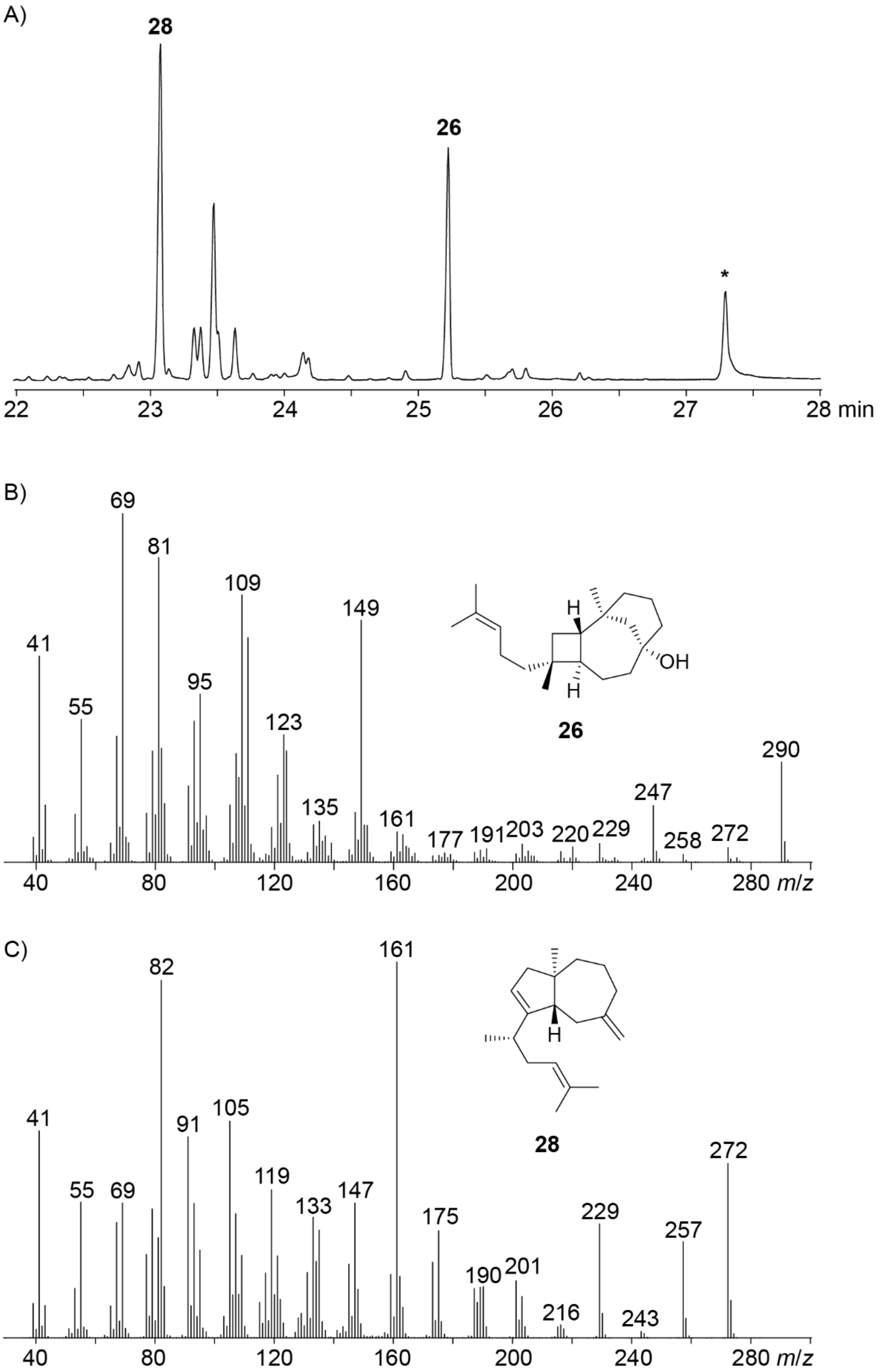

Figure S164. Product mixture formed from iso-GGPP I with CotB2. A) Total ion chromatogram of the crude extract from the enzyme incubation (asterisks indicate contaminants), B) EI mass spectrum of **26**, C) EI mass spectrum of **28**. Compound **27** is instable under GC conditions.

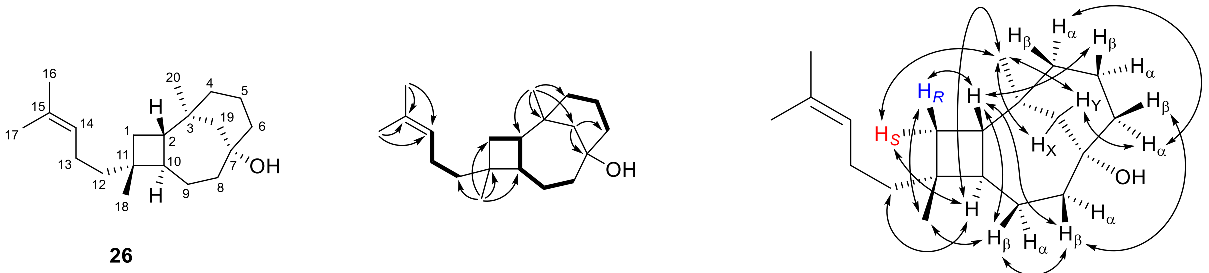

26

Figure S165. Structure elucidation of 2,3,7-*triepi*-variexenol B (**26**). Bold: ^{1}H,^{1}H-COSY, single-headed arrows: key HMBC, and double-headed arrows: key NOESY correlations.

Table S20. NMR data of 2,3,7-*triepi*-variexenol B (**26**) in C_6D_6 recorded at 298 K.

C[a]	type	^{13}C[b]	^{1}H[b]
1	CH_2	35.69	1.46 (dd, ^2J = 9.4, ^3J = 8.1, H_R)
			1.33 (dd, ^2J = 9.4, ^3J = 10.8, H_S)
2	CH	39.85	1.95 (m)
3	C_q	32.85	–
4	CH_2	38.24	1.22 (m, H_β)
			0.95 (m, H_α)
5	CH_2	21.00	1.59 (m, H_β)
			1.49 (m, H_α)
6	CH_2	40.09	1.64 (m, H_β)
			1.20 (m, H_α)
7	C_q	73.08	–
8	CH_2	40.19	1.69 (m, H_β)
			1.40 (m, H_α)
9	CH_2	23.39	1.56 (m, H_α)
			1.25 (m, H_β)
10	CH	45.62	1.61 (m)
11	C_q	37.99	–
12	CH_2	45.42	1.38 (m, 2H)
13	CH_2	23.71	1.98 (m, 2H)
14	CH	125.77	5.22 (m)
15	C_q	130.70	–
16	CH_3	25.91	1.70 (br s)
17	CH_3	17.69	1.59 (br s)
18	CH_3	18.14	0.96 (s)
19	CH_2	50.42	1.64 (m, H_X)
			0.90 (m, H_Y)
20	CH_3	27.17	0.77 (s)

[a] Carbon numbering as shown in Figure S165. [b] Chemical shifts δ in ppm, multiplicity: s = singlet, d = doublet, m = multiplet, br = broad, coupling constants J are given in Hertz.

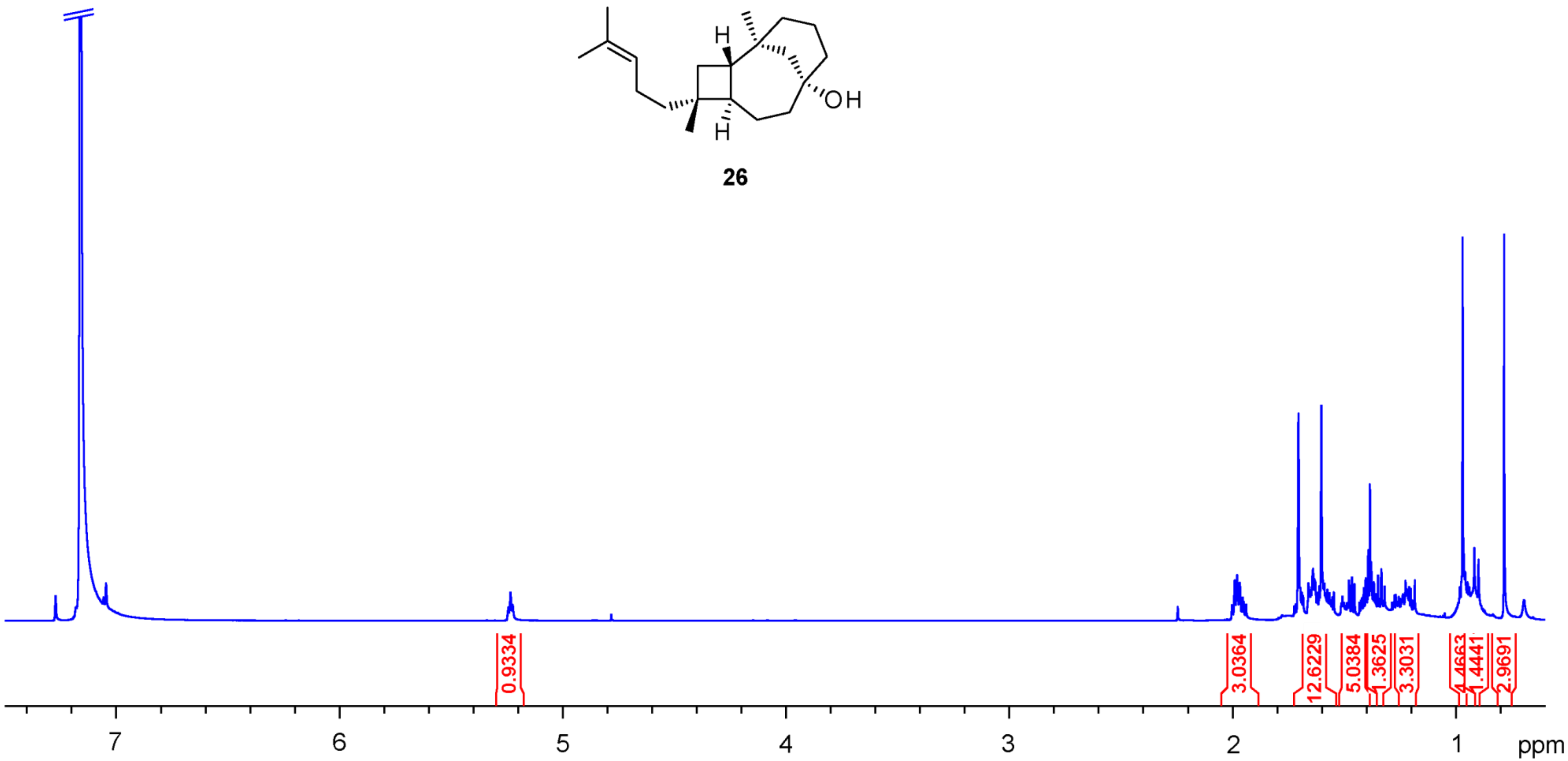

Figure S166. ^{1}H-NMR spectrum (700 MHz, C_6D_6) of **26**.

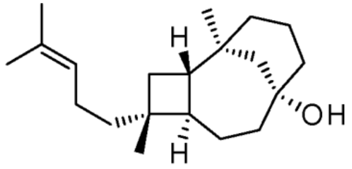

26

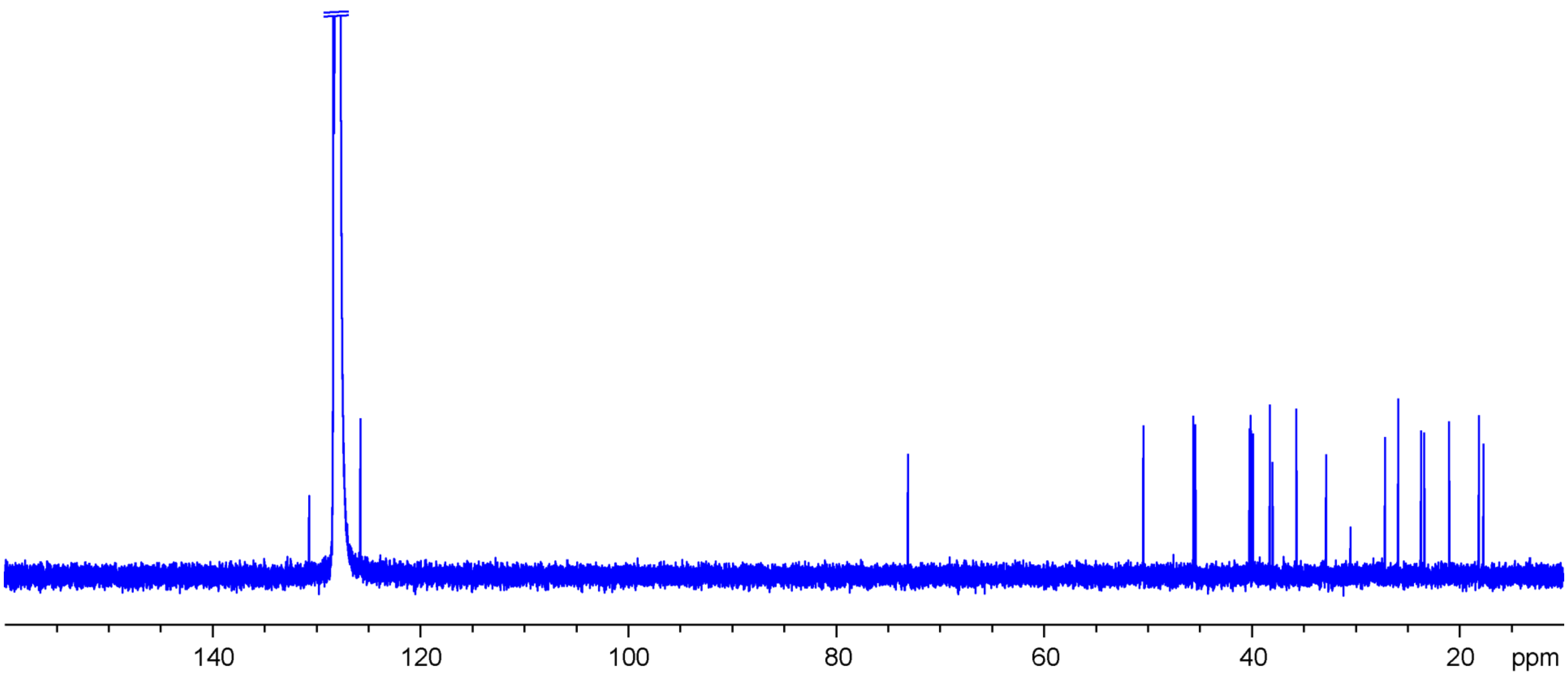

Figure S167. ^{13}C-NMR spectrum (176 MHz, C_6D_6) of **26**.

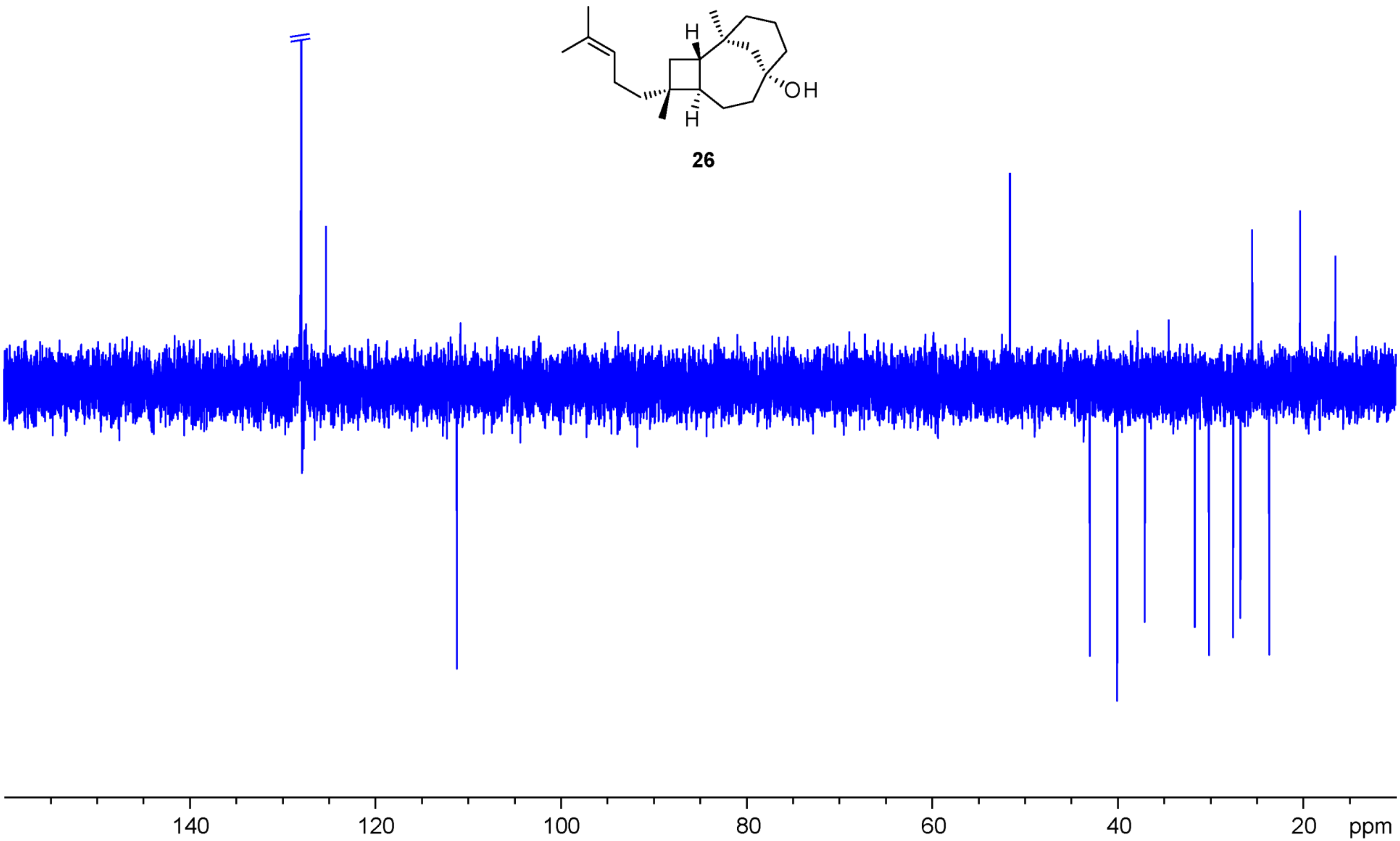

Figure S168. ^{13}C-DEPT135 spectrum (176 MHz, C_6D_6) of **26**.

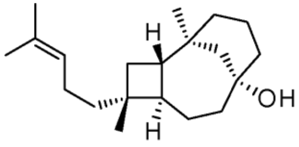

26

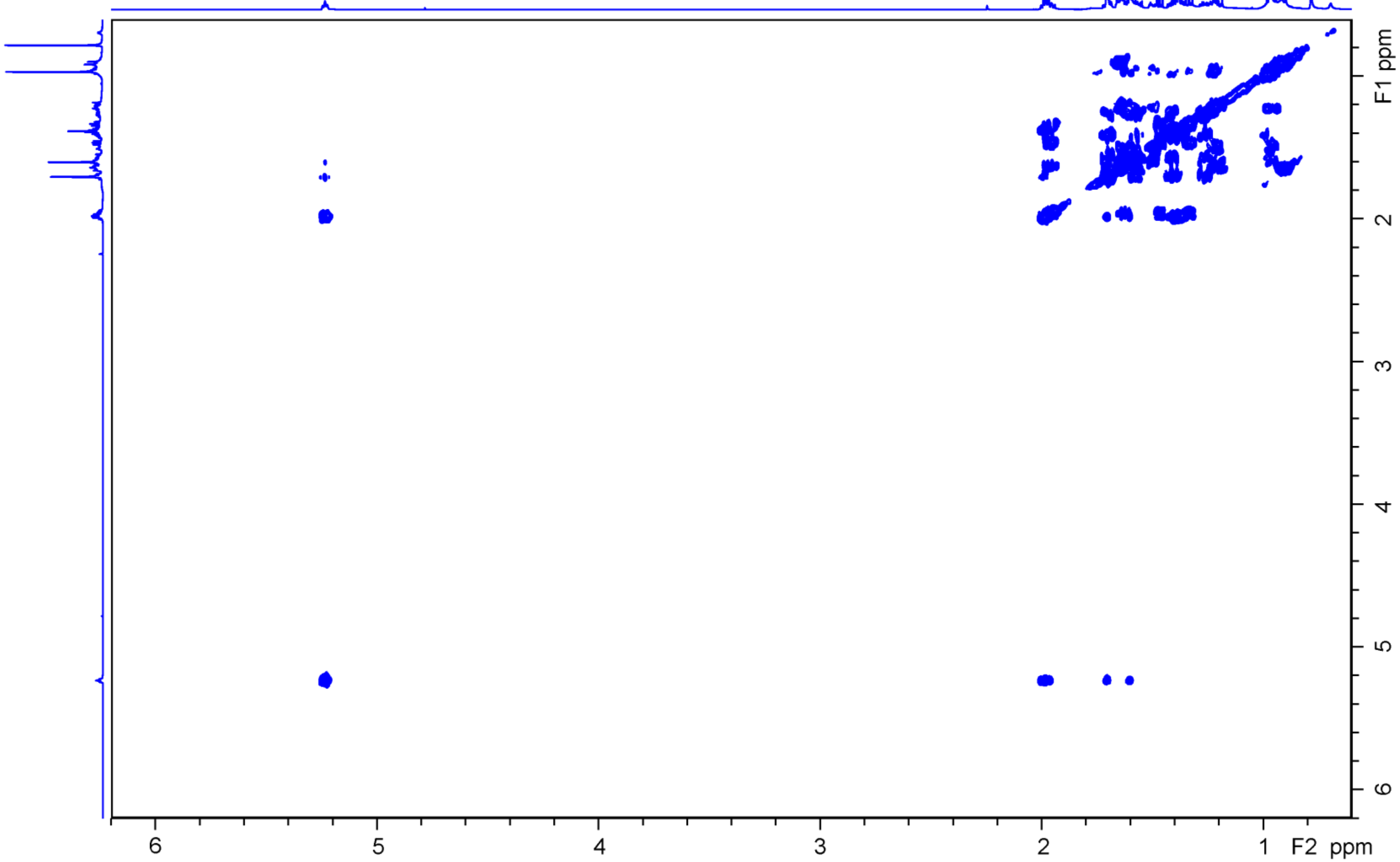

Figure S169. $^{1}H,^{1}H$-COSY spectrum (C_6D_6) of **26**.

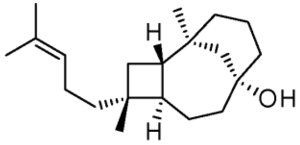

26

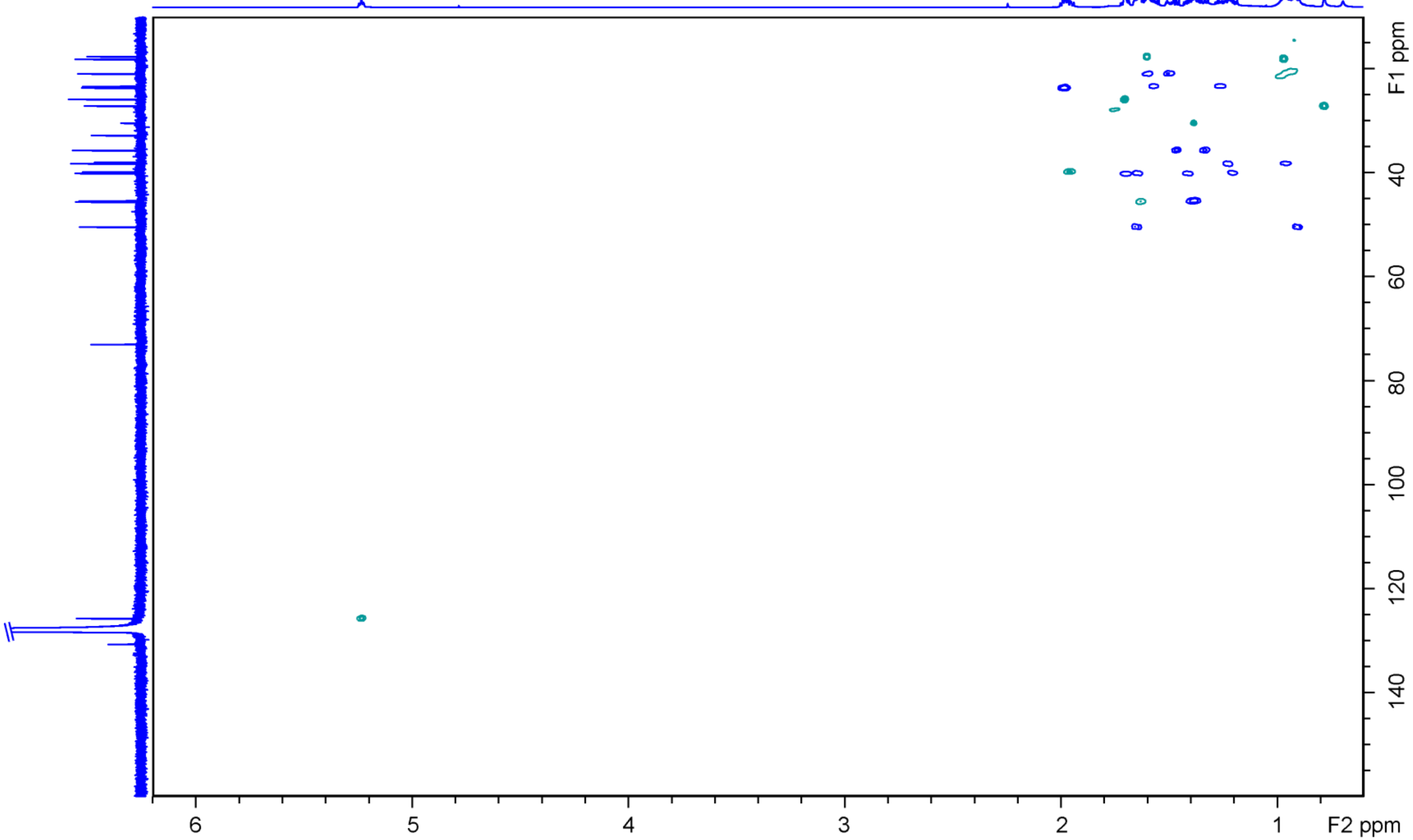

Figure S170. HSQC spectrum (C_6D_6) of **26**.

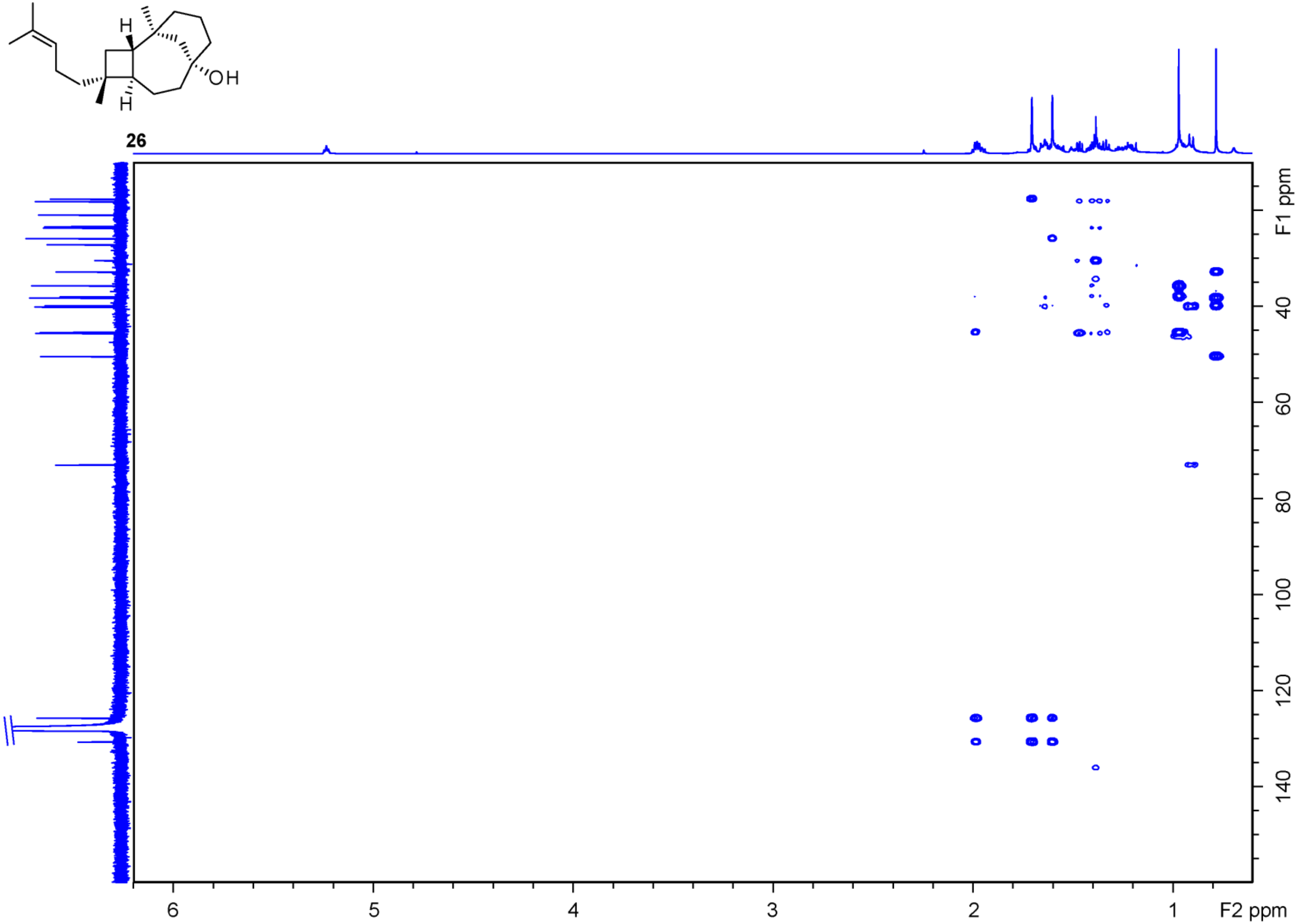

Figure S171. HMBC spectrum (C_6D_6) of **26**.

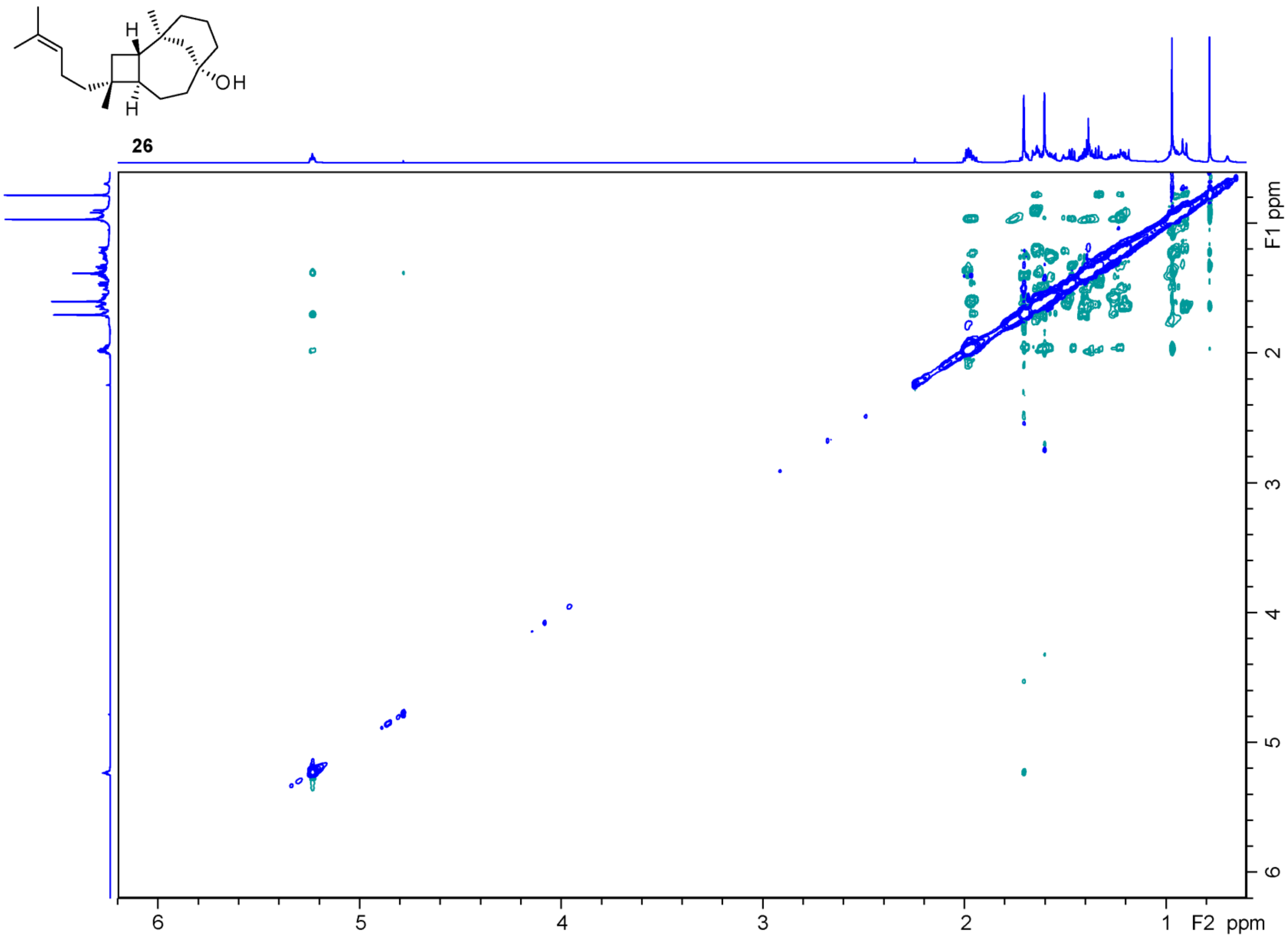

Figure S172. NOESY spectrum (C_6D_6) of **26**.

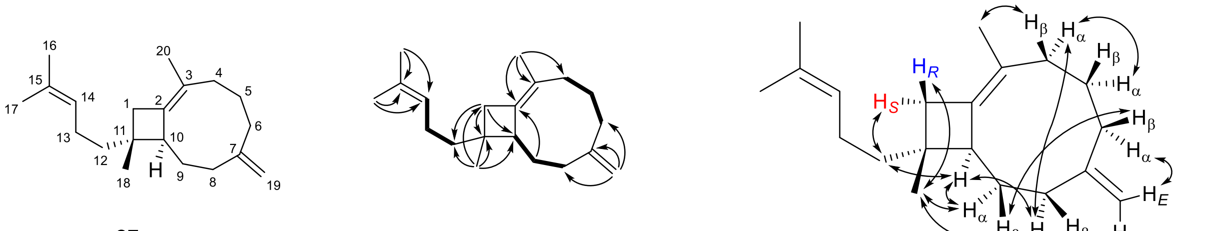

27

Figure S173. Structure elucidation of isoxeniaphyllene (**27**). Bold: ^{1}H,^{1}H-COSY, single-headed arrows: key HMBC, and double-headed arrows: key NOESY correlations.

Table S21. NMR data of isoxeniaphyllene (**27**) in C_6D_6 recorded at 298 K.

C[a]	type	^{13}C[b]	^{1}H[b]
1	CH$_2$	40.40	2.30 (br d, ^2J = 15.1, H$_S$)
			2.26 (br d, ^2J = 15.1, H$_R$)
2	C$_q$	135.53	–
3	C$_q$	126.41	–
4	CH$_2$	32.05	2.32 (m, H$_α$)
			1.83 (m, H$_β$)
5	CH$_2$	27.13	1.61 (m, H$_β$)
			1.54 (m, H$_α$)
6	CH$_2$	30.51	2.21 (m, H$_β$)
			2.04 (m, H$_α$)
7	C$_q$	151.78	–
8	CH$_2$	37.45	2.51 (m, H$_β$)
			2.19 (m, H$_α$)
9	CH$_2$	27.93	1.69 (m, H$_α$)
			1.50 (m, H$_β$)
10	CH	51.98	2.38 (m)
11	C$_q$	36.60	–
12	CH$_2$	43.36	1.50 (m, 2H)
13	CH$_2$	24.02	2.00 (m, 2H)
14	CH	125.68	5.20 (m)
15	C$_q$	130.74	–
16	CH$_3$	25.88	1.67 (br s)
17	CH$_3$	17.65	1.56 (br s)
18	CH$_3$	20.70	0.95 (s)
19	CH$_2$	111.57	4.88 (m, H$_Z$)
			4.80 (m, H$_E$)
20	CH$_3$	16.90	1.47 (br s)

[a] Carbon numbering as shown in Figure S173. [b] Chemical shifts δ in ppm, multiplicity: s = singlet, d = doublet, m = multiplet, br = broad, coupling constants J are given in Hertz.

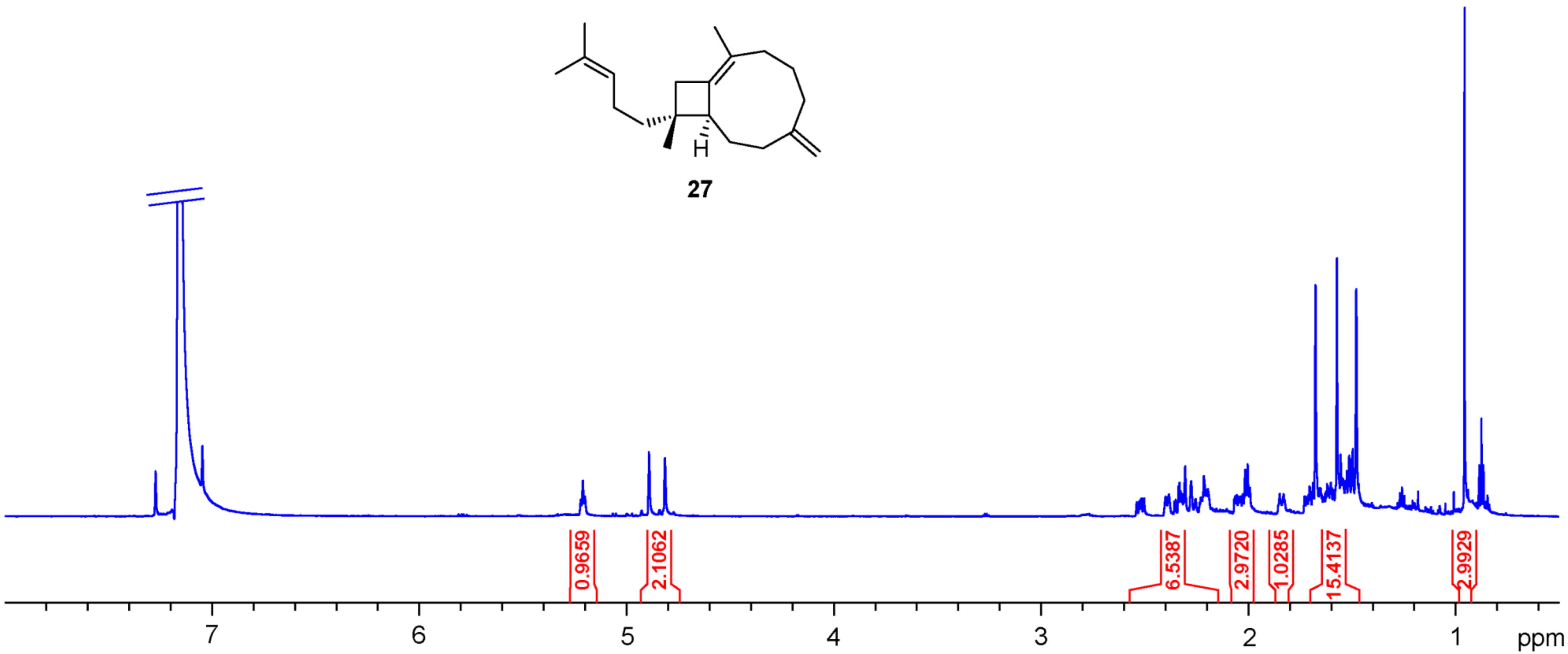

Figure S174. ^{1}H-NMR spectrum (700 MHz, C_6D_6) of **27**.

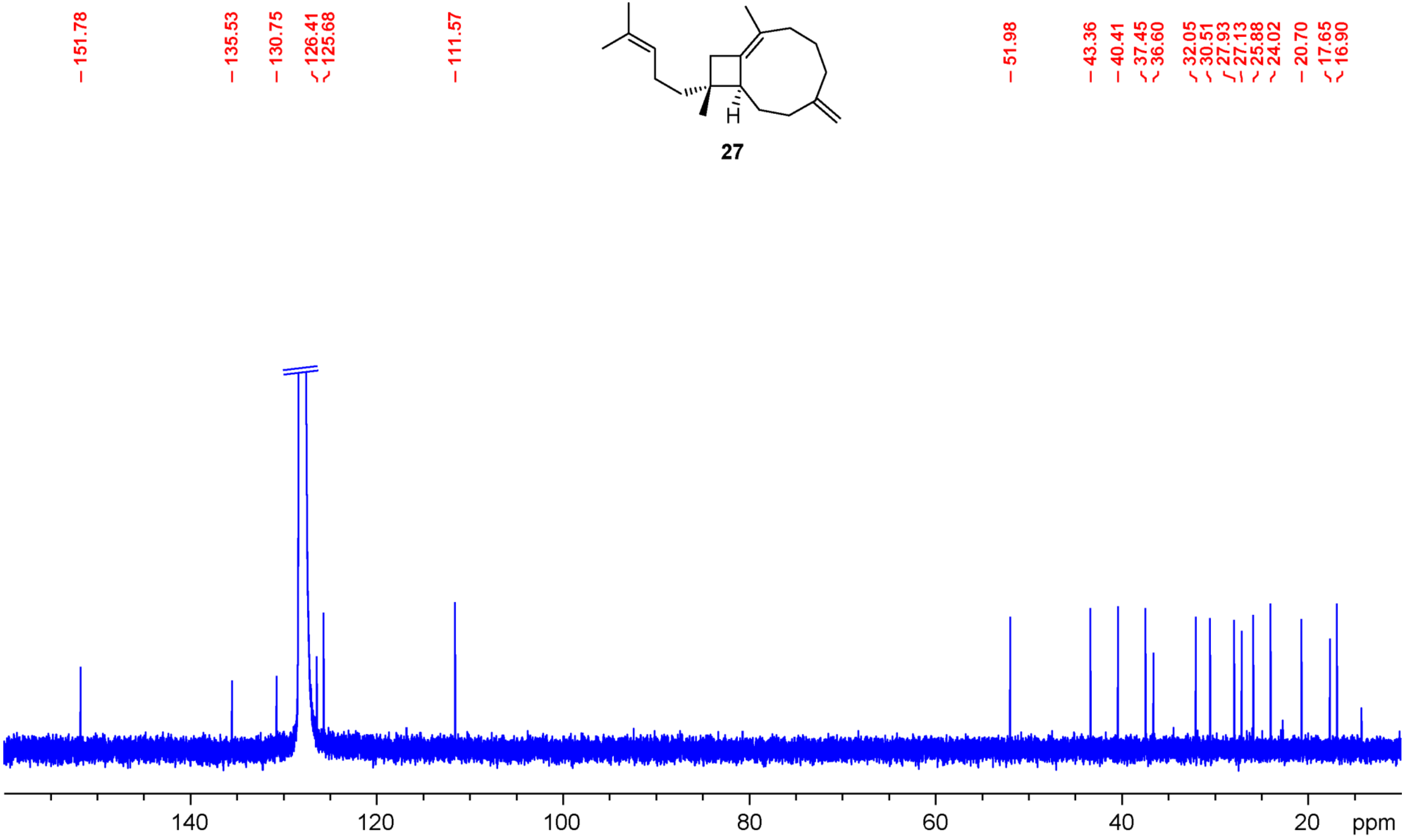

Figure S175. ^{13}C-NMR spectrum (176 MHz, C_6D_6) of **27**.

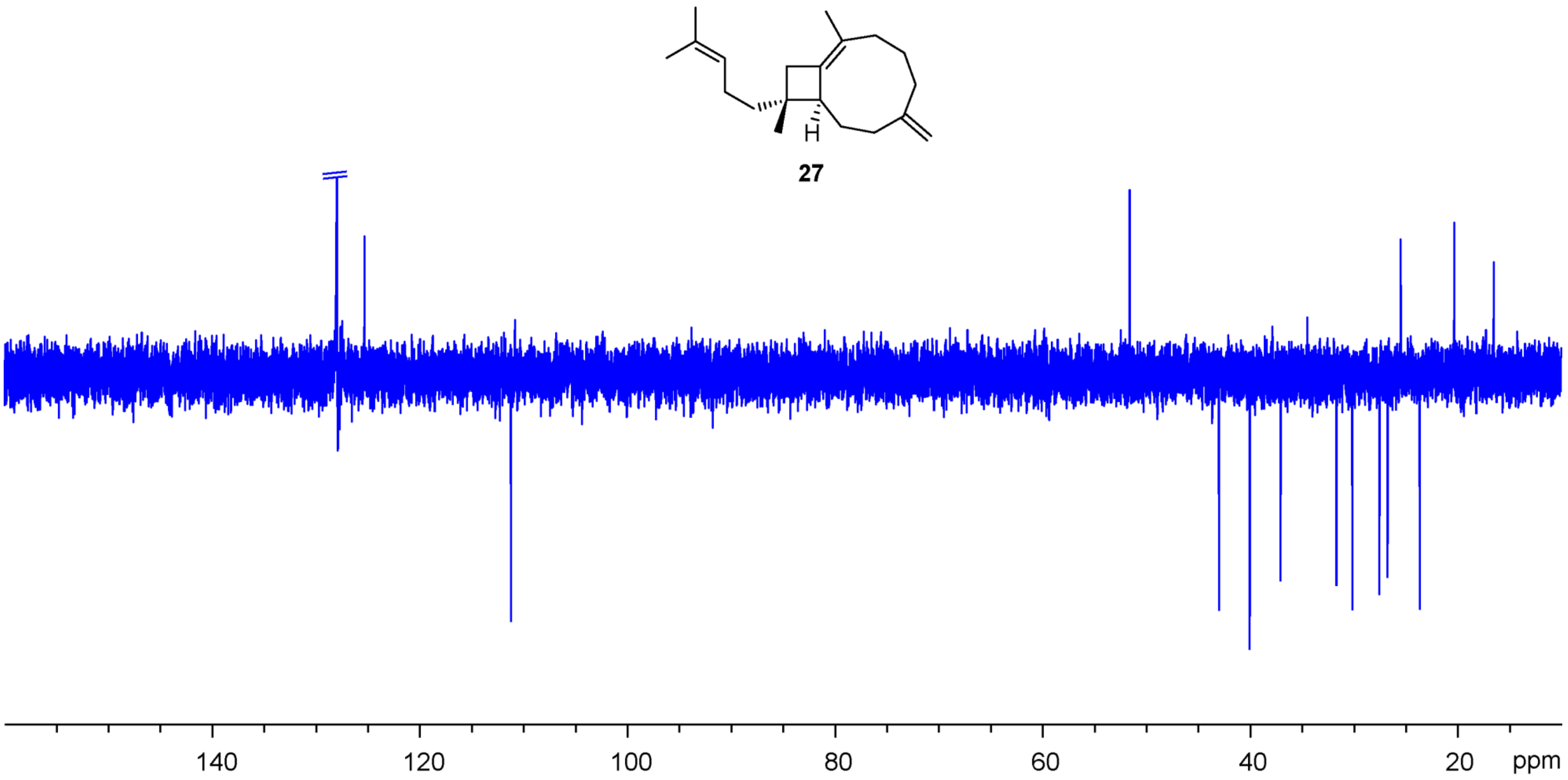

Figure S176. ^{13}C-DEPT135 spectrum (176 MHz, C_6D_6) of **27**.

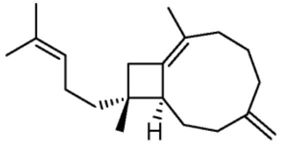

27

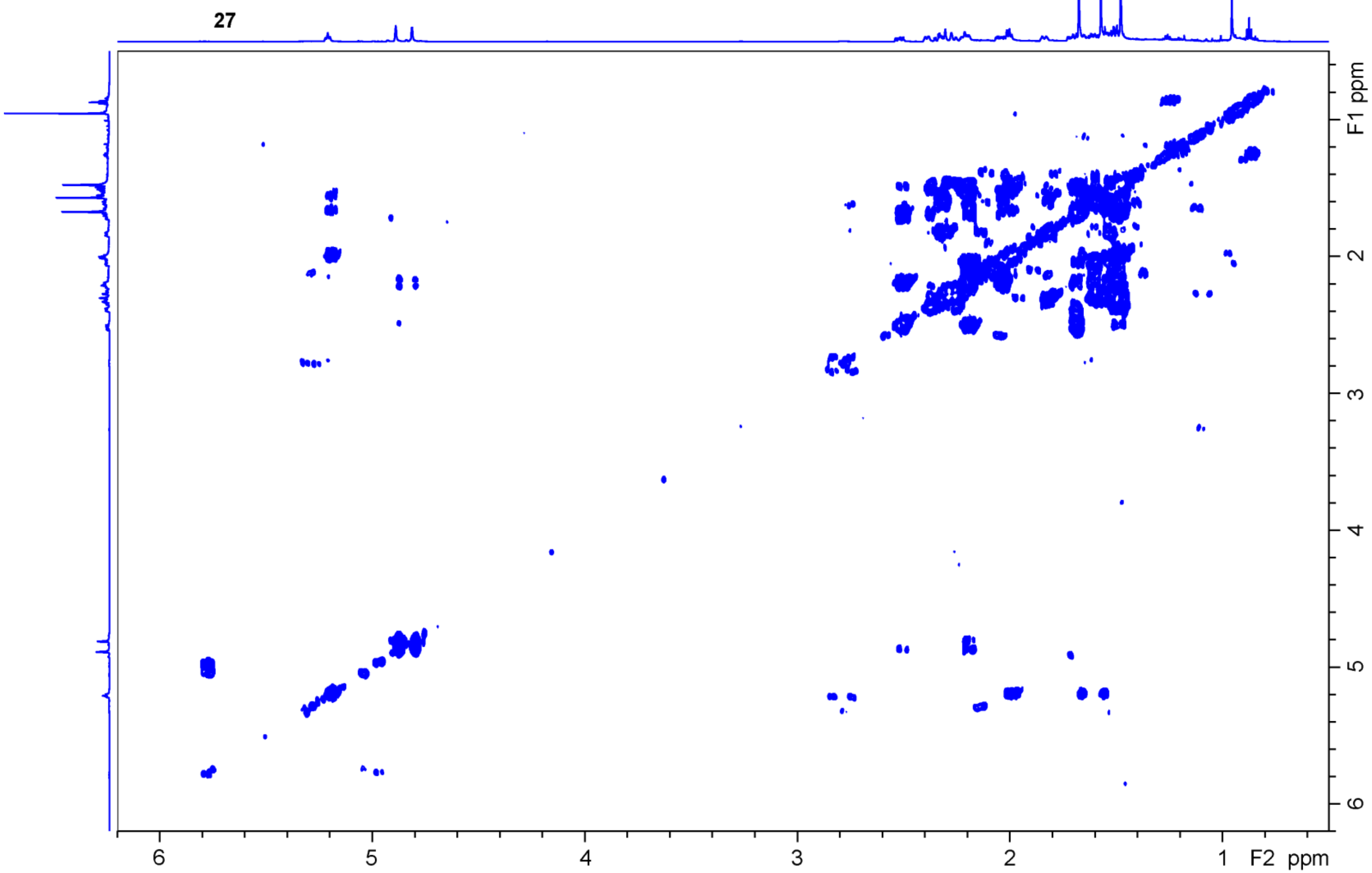

Figure S177. $^{1}H,^{1}H$-COSY spectrum (C_6D_6) of **27**.

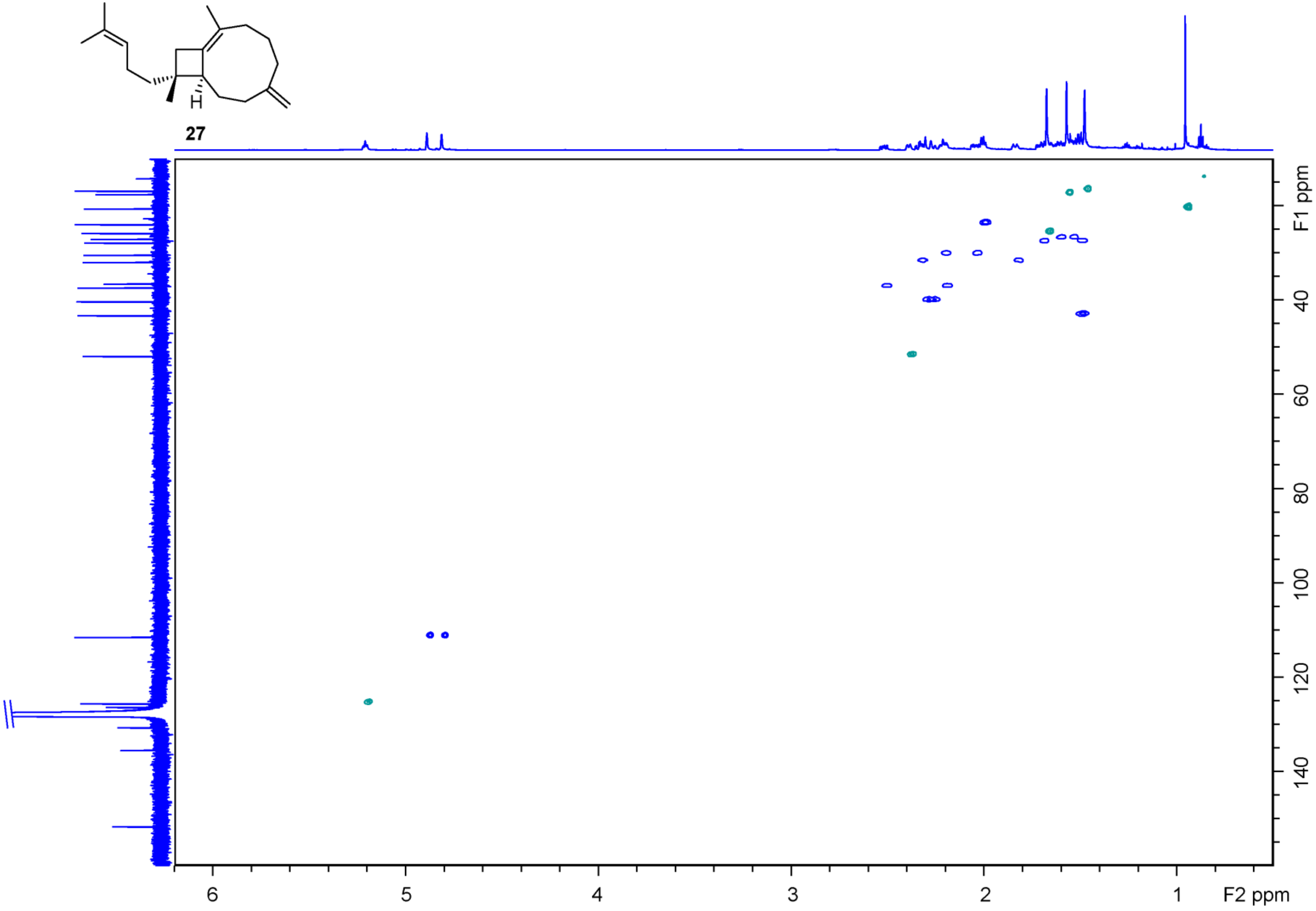

Figure S178. HSQC spectrum (C_6D_6) of **27**.

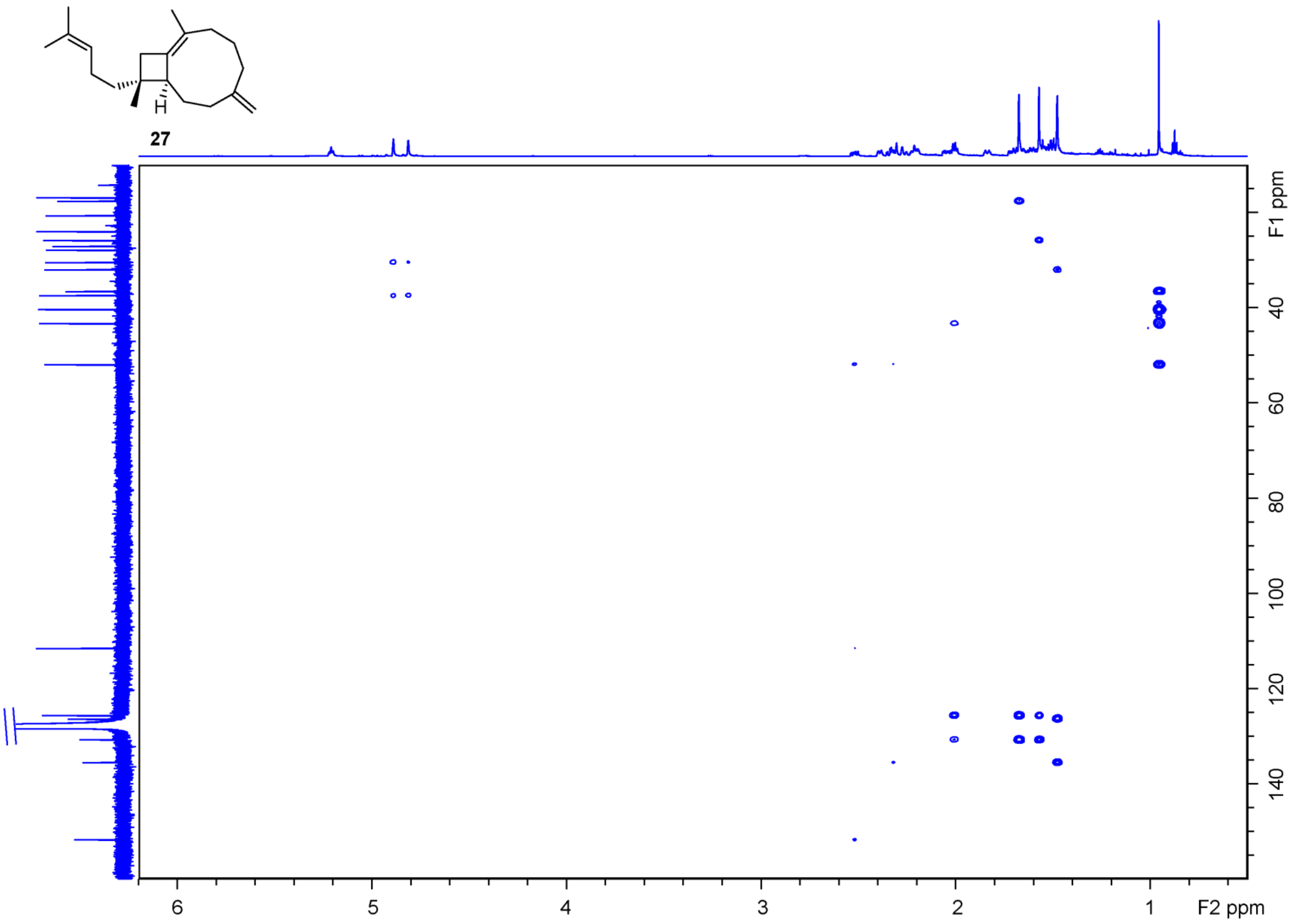

Figure S179. HMBC spectrum (C_6D_6) of **27**.

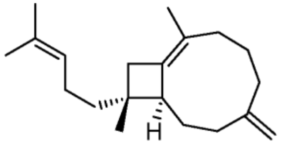
27

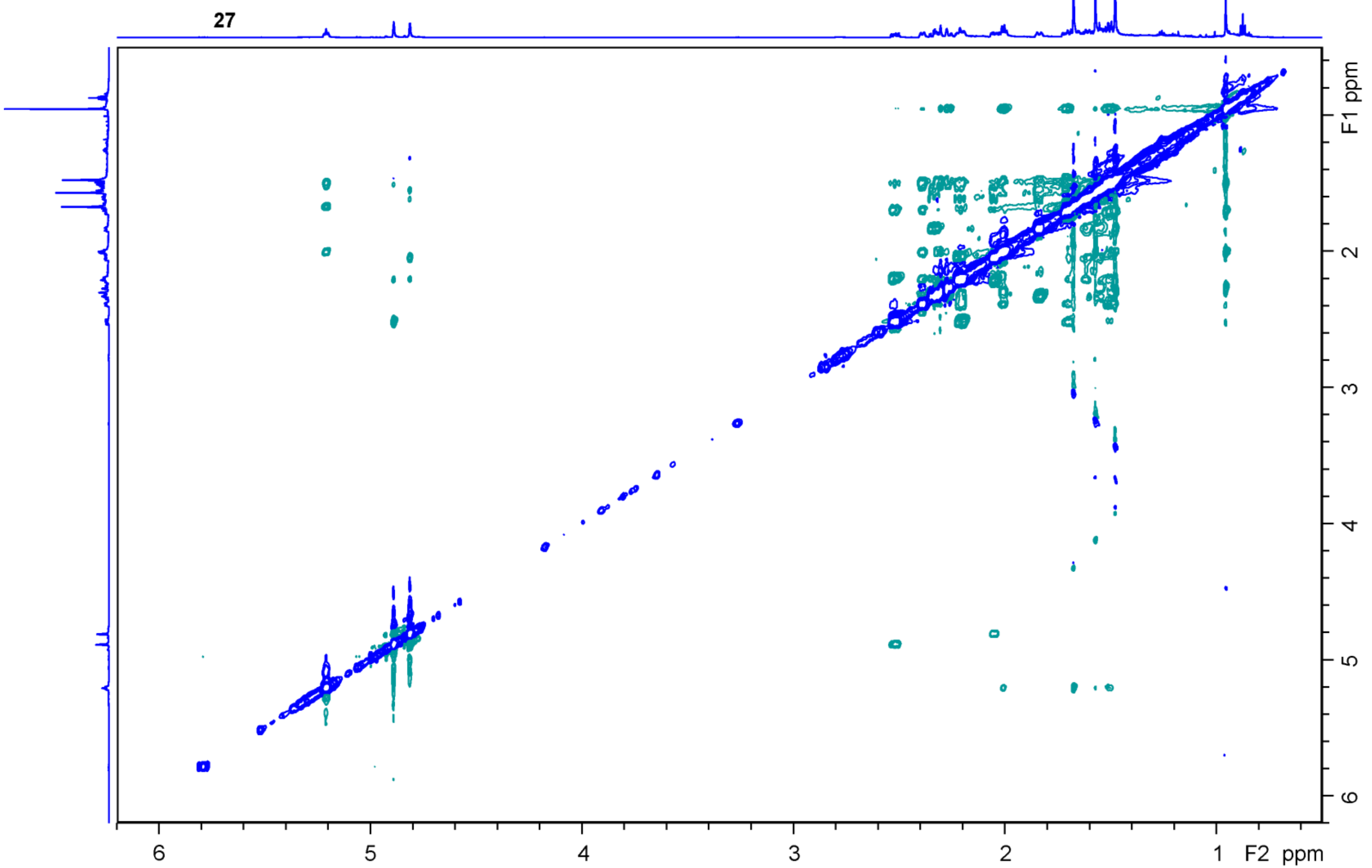

Figure S180. NOESY spectrum (C_6D_6) of **27**.

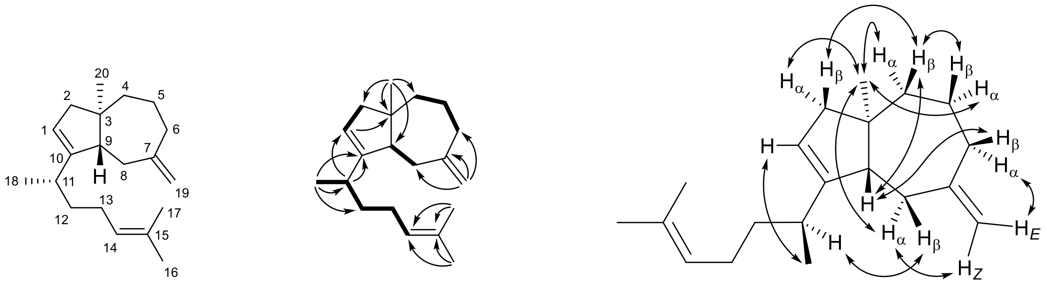

28

Figure S181. Structure elucidation of prenylisodauca-3,7(14)-diene (**28**). Bold: $^{1}H,^{1}H$-COSY, single-headed arrows: key HMBC, and double-headed arrows: key NOESY correlations.

Table S22. NMR data of prenylisodauca-3,7(14)-diene (**28**) in C_6D_6 recorded at 298 K.

C[a]	type	^{13}C[b]	^{1}H[b]
1	CH	120.24	5.32 (m)
2	CH_2	48.68	2.15 (m, H_β)
			1.96 (m, H_α)
3	C_q	46.94	–
4	CH_2	42.85	1.67 (m, H_α)
			1.39 (m, H_β)
5	CH_2	29.02	1.74 (m, H_β)
			1.51 (m, H_α)
6	CH_2	35.97	2.31 (dd, ^{2}J = 13.0, ^{3}J = 5.9, H_α)
			2.01 (m, H_β)
7	C_q	151.56	–
8	CH_2	33.95	2.67 (m, H_β)
			2.19 (dd, ^{2}J = 16.3, ^{3}J = 13.2, H_α)
9	CH	50.47	2.78 (m)
10	C_q	150.68	–
11	CH	32.30	2.05 (m)
12	CH_2	35.88	1.70 (m)
			1.42 (m)
13	CH_2	26.72	2.11 (m, 2H)
14	CH	125.51	5.32 (thept, ^{3}J = 7.2, ^{4}J = 1.5)
15	C_q	131.10	–
16	CH_3	25.92	1.69 (br s)
17	CH_3	17.85	1.60 (br s)
18	CH_3	20.79	0.95 (d, ^{3}J = 6.9)
19	CH_2	111.46	4.89 (m, H_E)
			4.80 (m, H_Z)
20	CH_3	19.73	0.99 (s)

[a] Carbon numbering as shown in Figure S181. [b] Chemical shifts δ in ppm, multiplicity: s = singlet, d = doublet, hept = heptet, m = multiplet, br = broad, coupling constants J are given in Hertz.

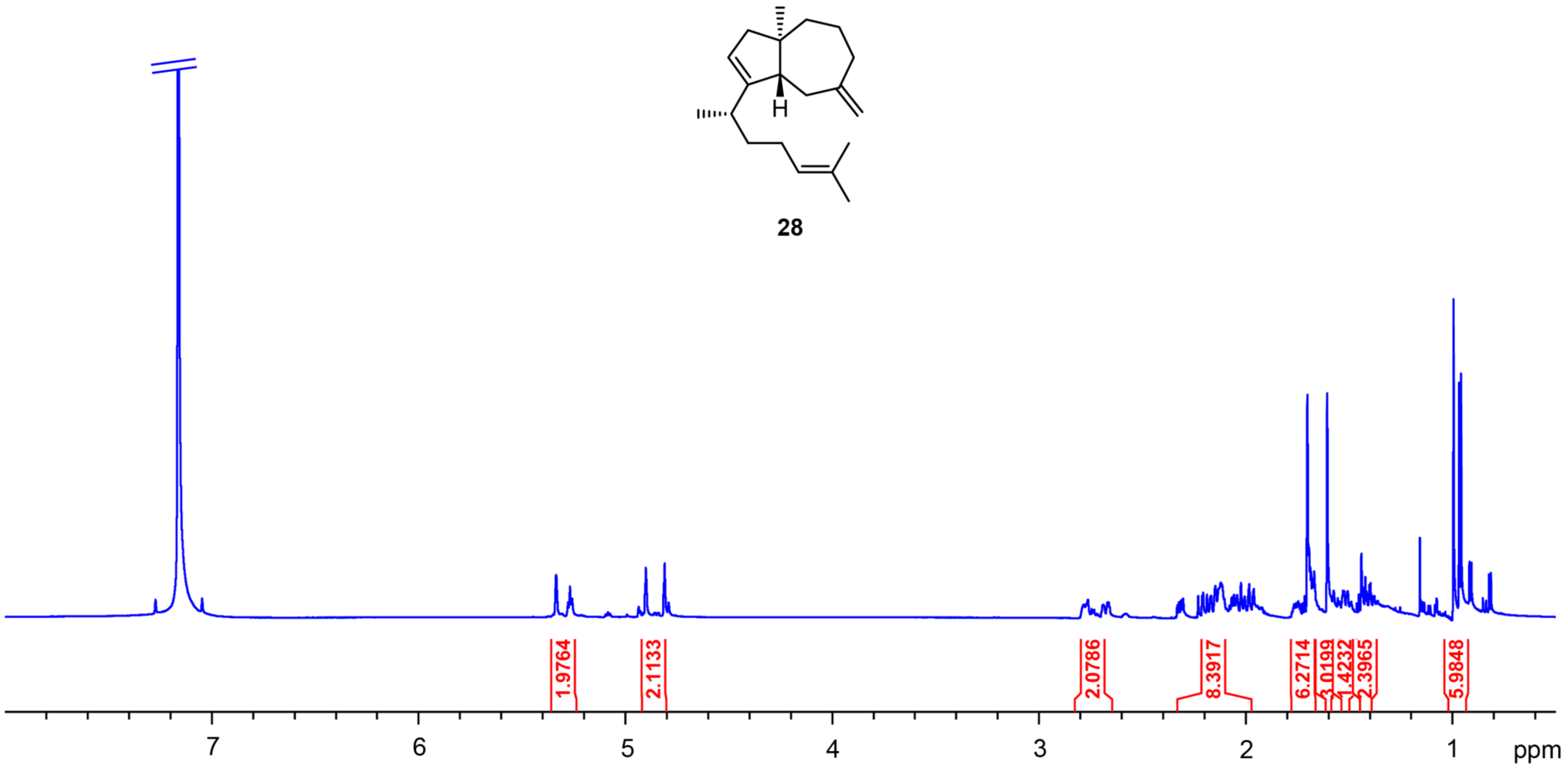

Figure S182. ^{1}H-NMR spectrum (700 MHz, C_6D_6) of **28**.

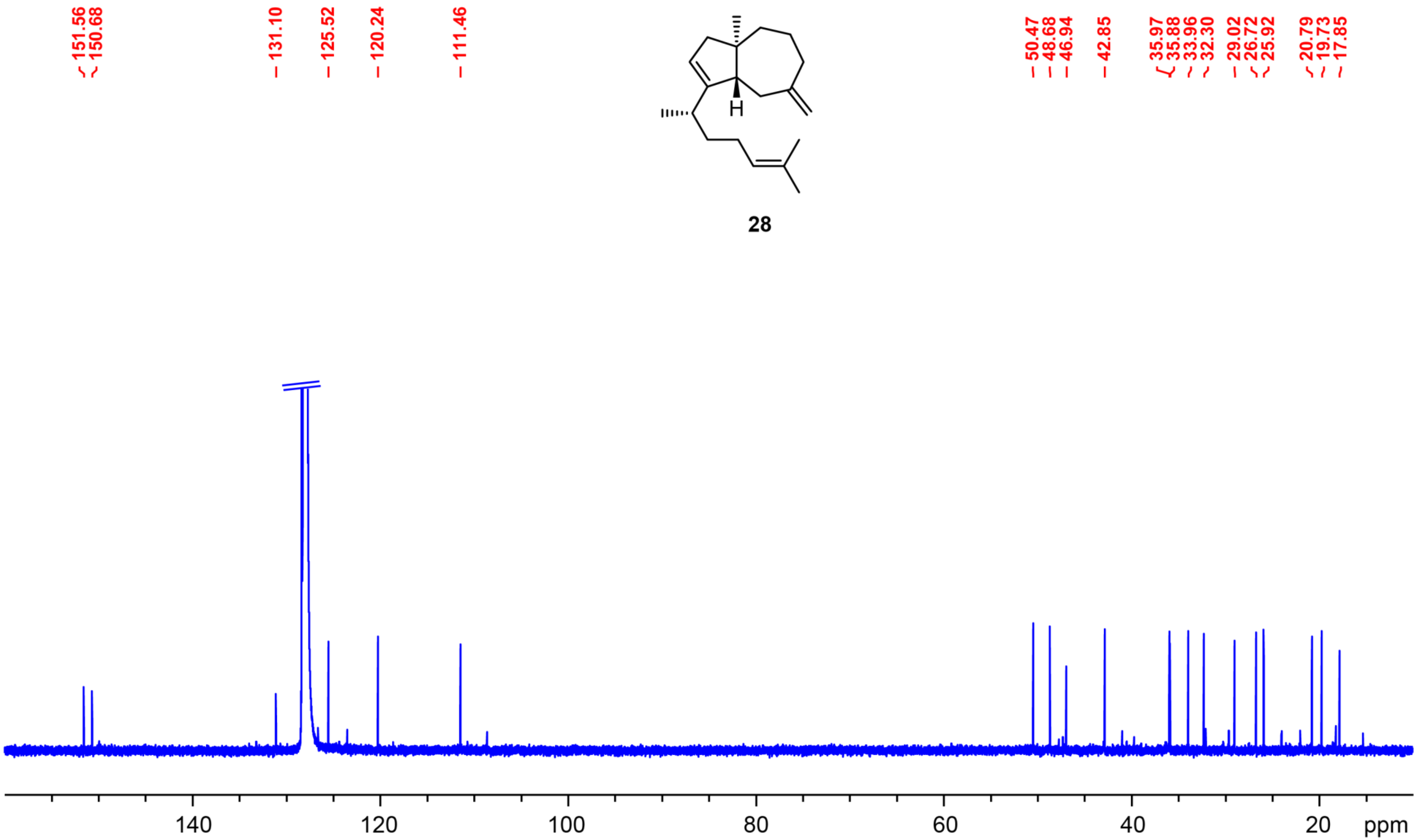

Figure S183. ^{13}C-NMR spectrum (176 MHz, C_6D_6) of **28**.

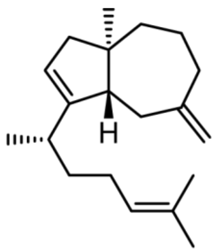

28

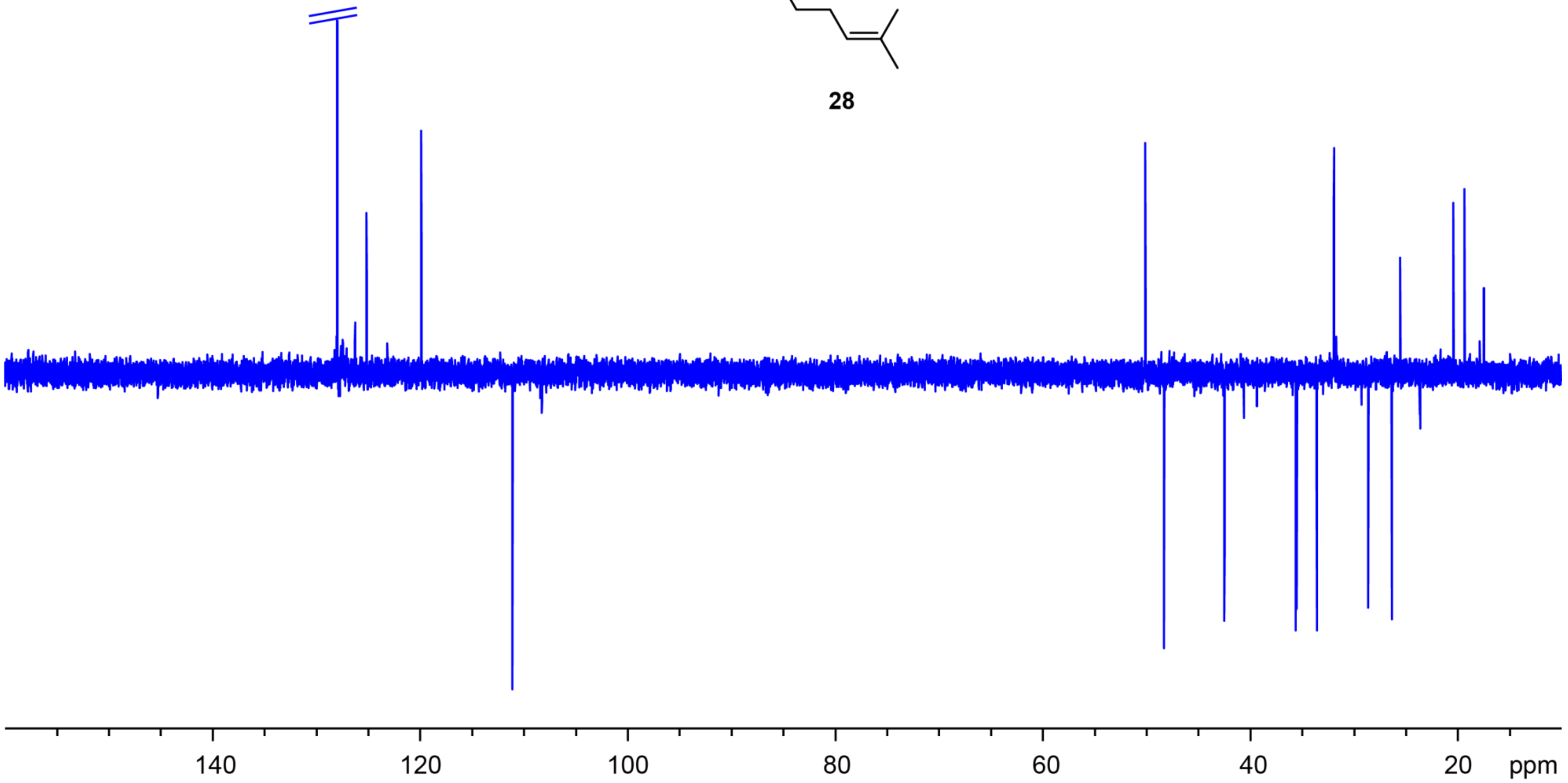

Figure S184. ^{13}C-DEPT135 spectrum (176 MHz, C_6D_6) of **28**.

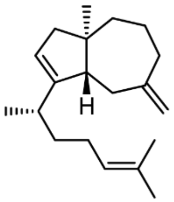

28

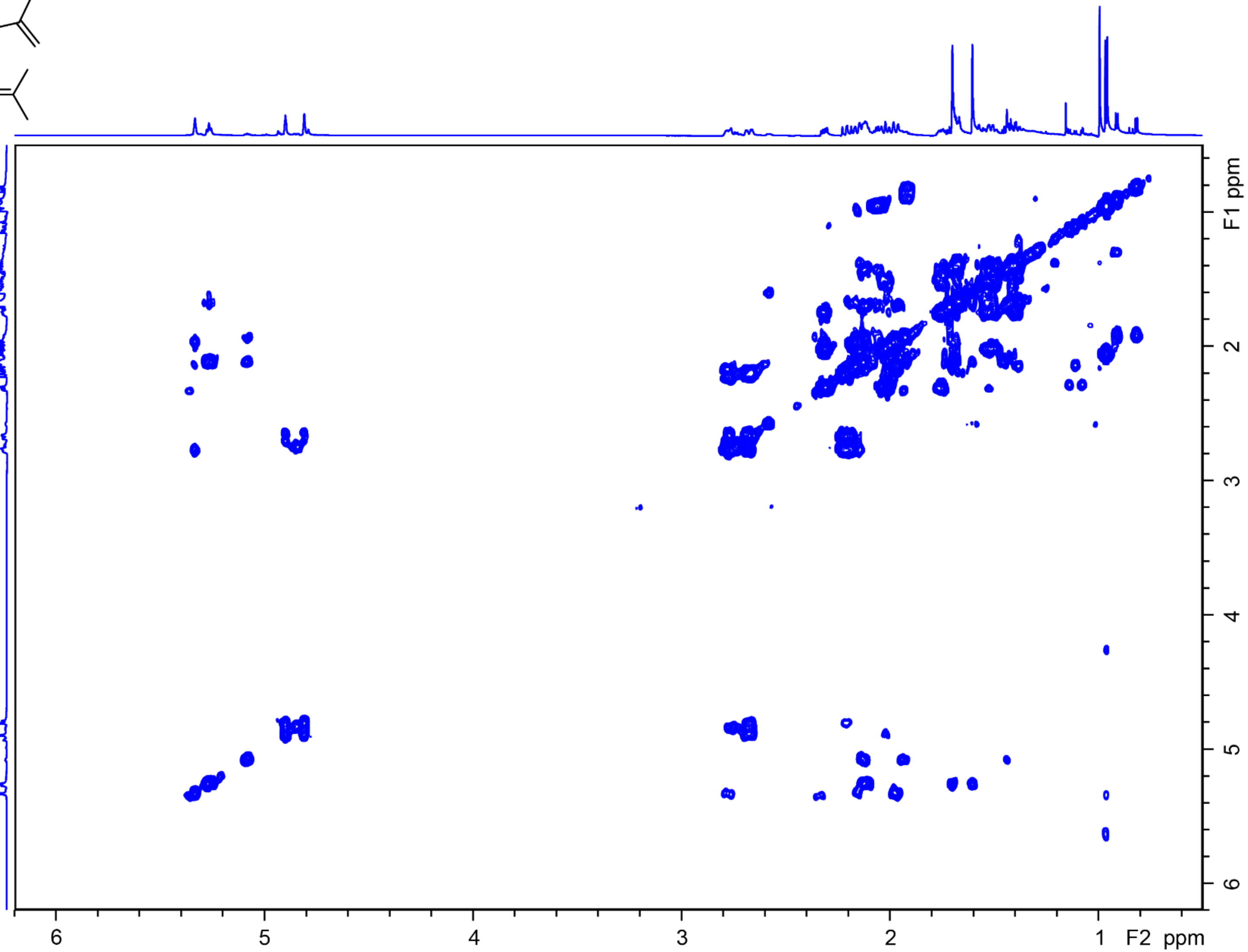

Figure S185. $^{1}H,^{1}H$-COSY spectrum (C_6D_6) of **28**.

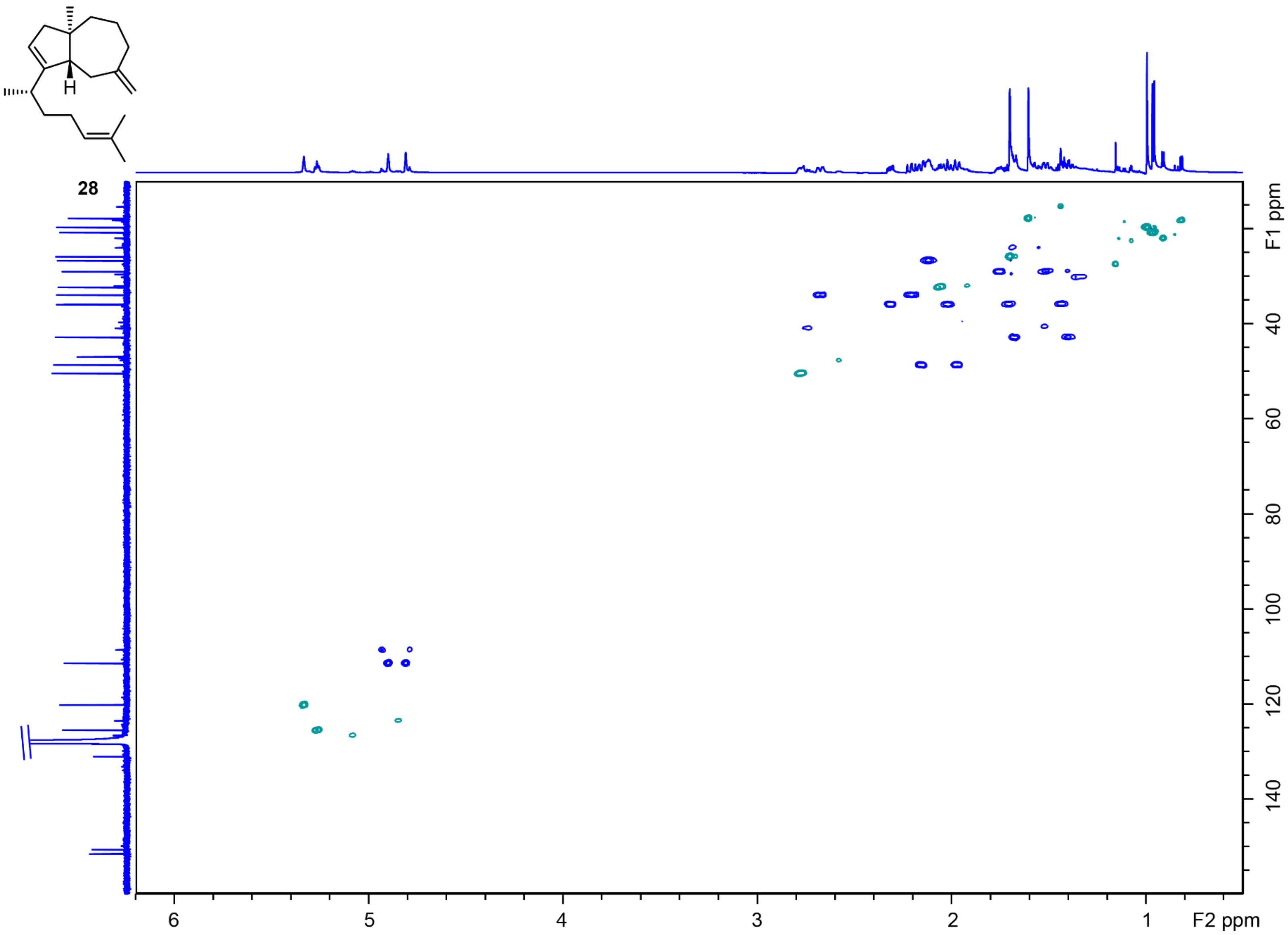

Figure S186. HSQC spectrum (C_6D_6) of **28**.

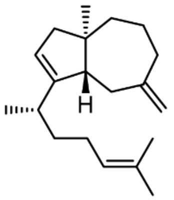

28

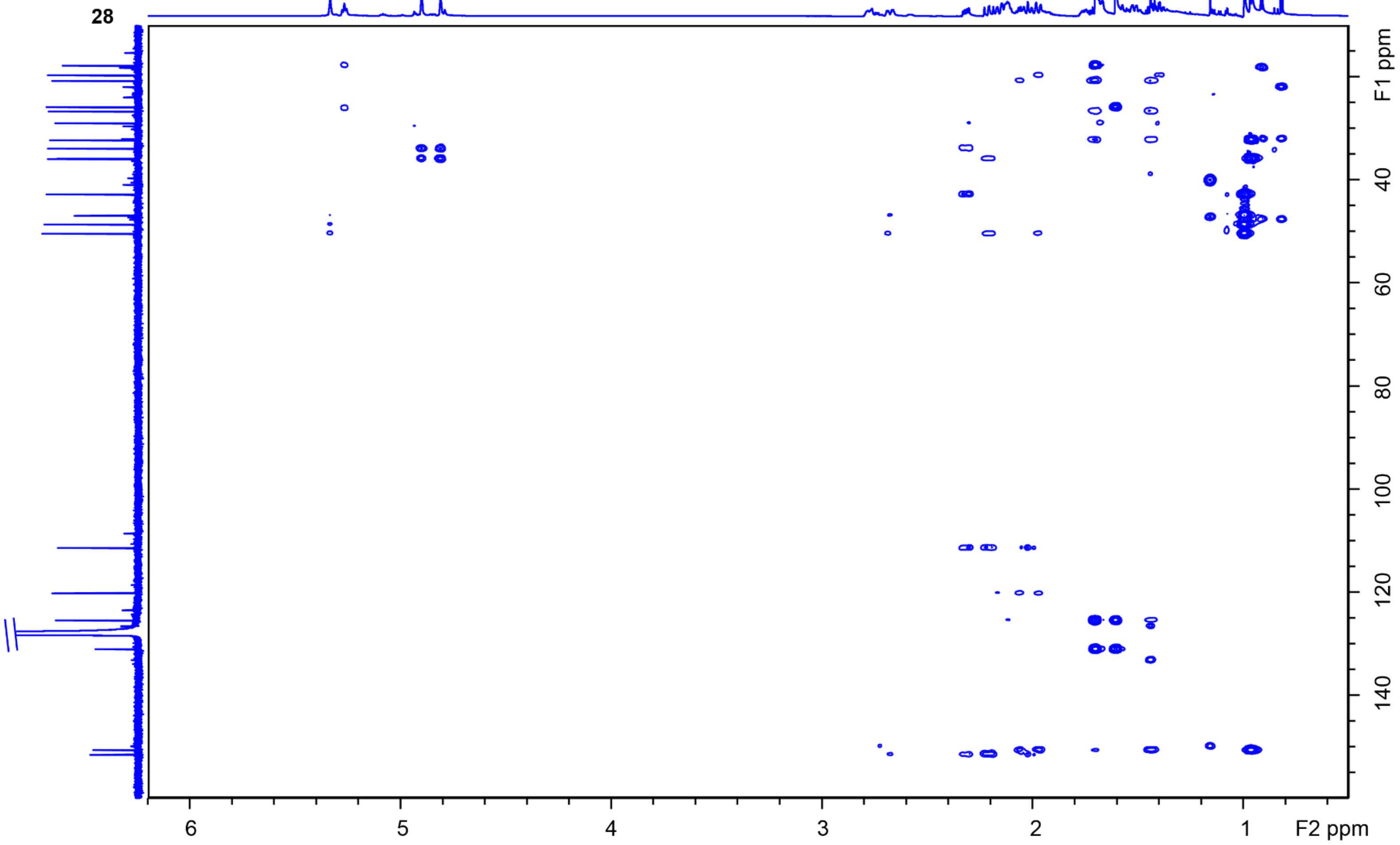

Figure S187. HMBC spectrum (C_6D_6) of **28**.

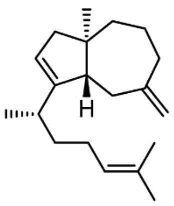
28

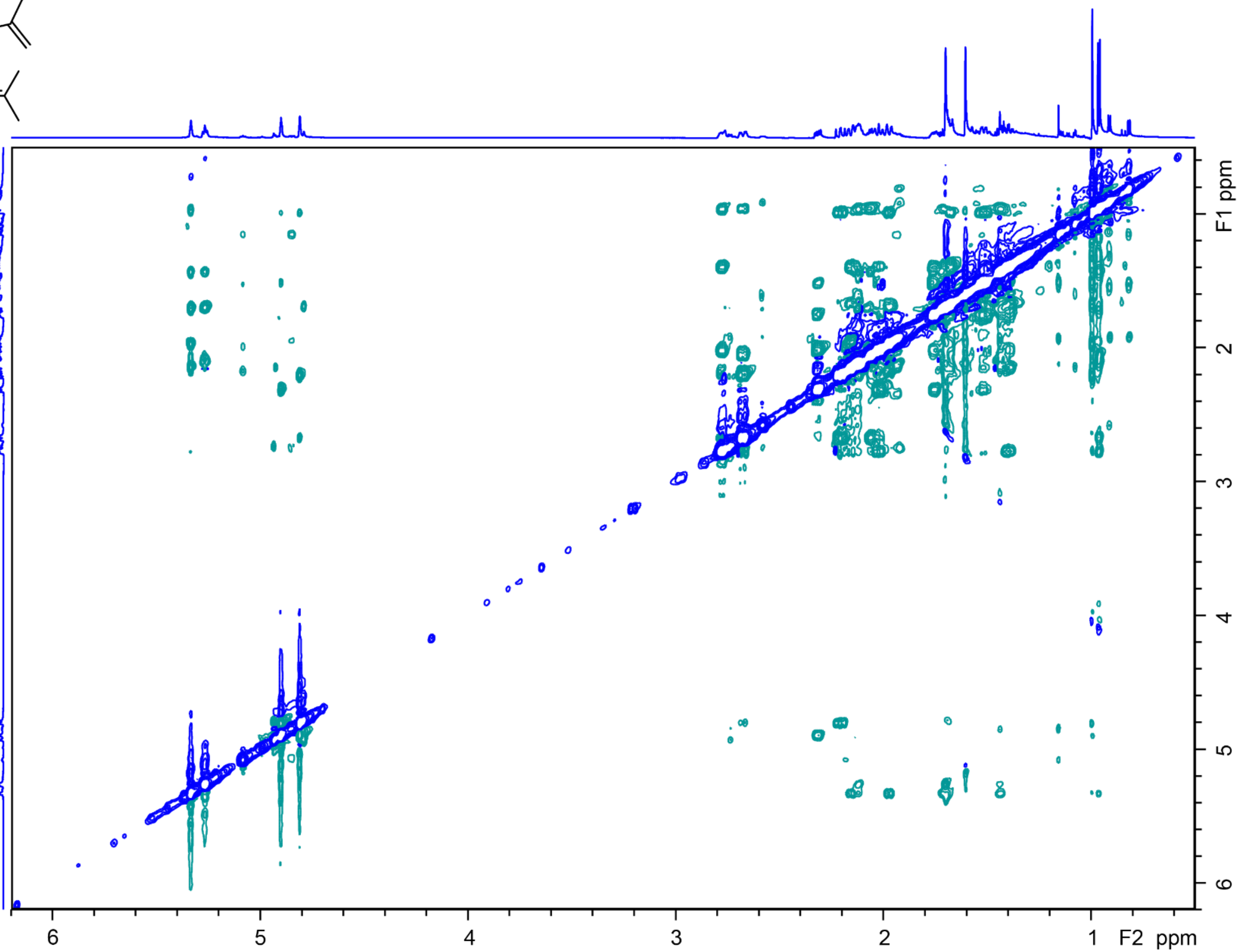

Figure S188. NOESY spectrum (C_6D_6) of **28**.

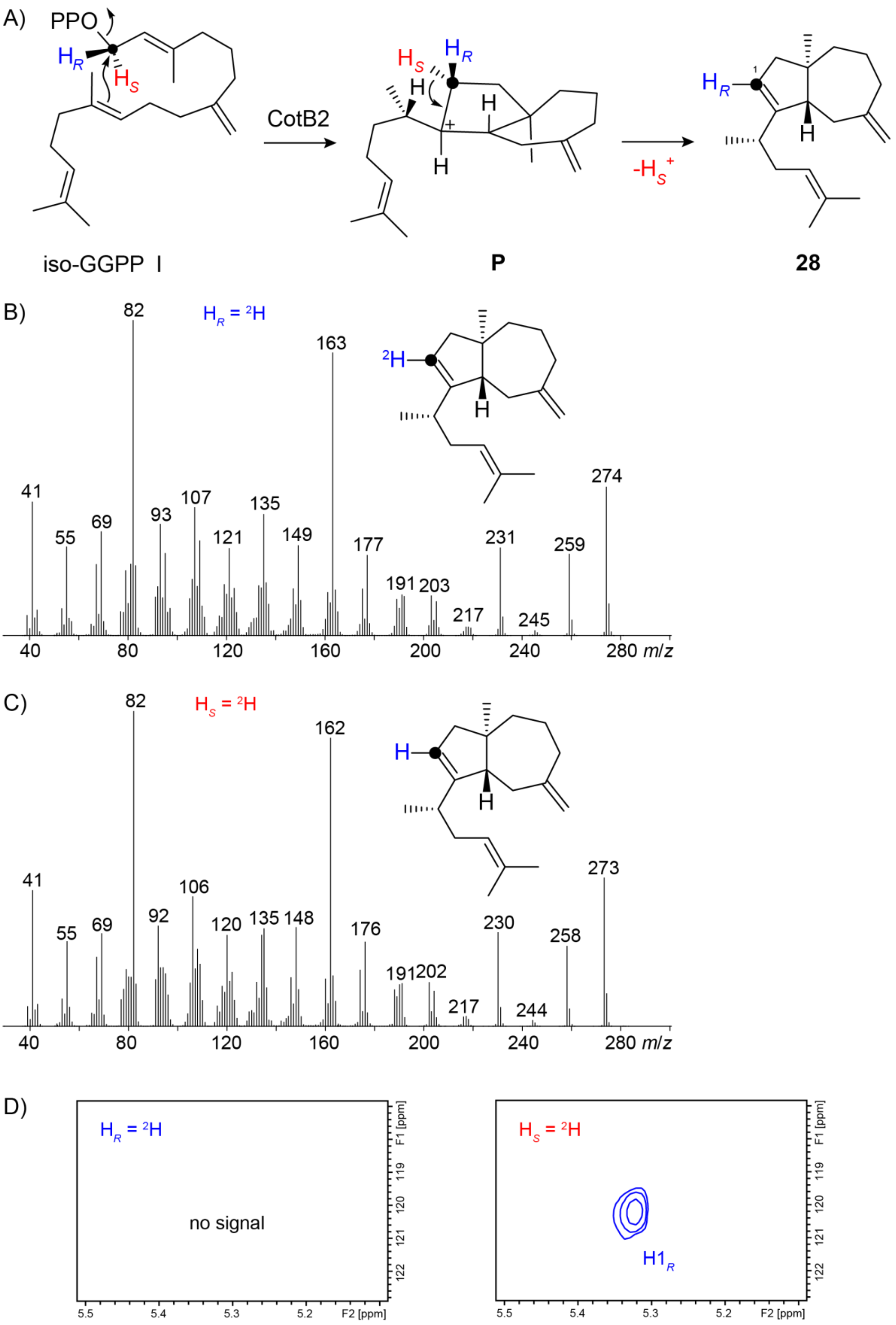

Figure S189. A) The deprotonation to **28** proceeds with loss of the 1-*pro-S* proton. EI mass spectra of the products obtained from B) (*R*)-(1-^{13}C,1-^{2}H)-iso-GGPP I and C) (*S*)-(1-^{13}C,1-^{2}H)-iso-GGPP I. D) HSQC analysis of the products shows that the 1-*pro-R* hydrogen remains bound to C1 (no signal, if this hydrogen is substituted by deuterium).

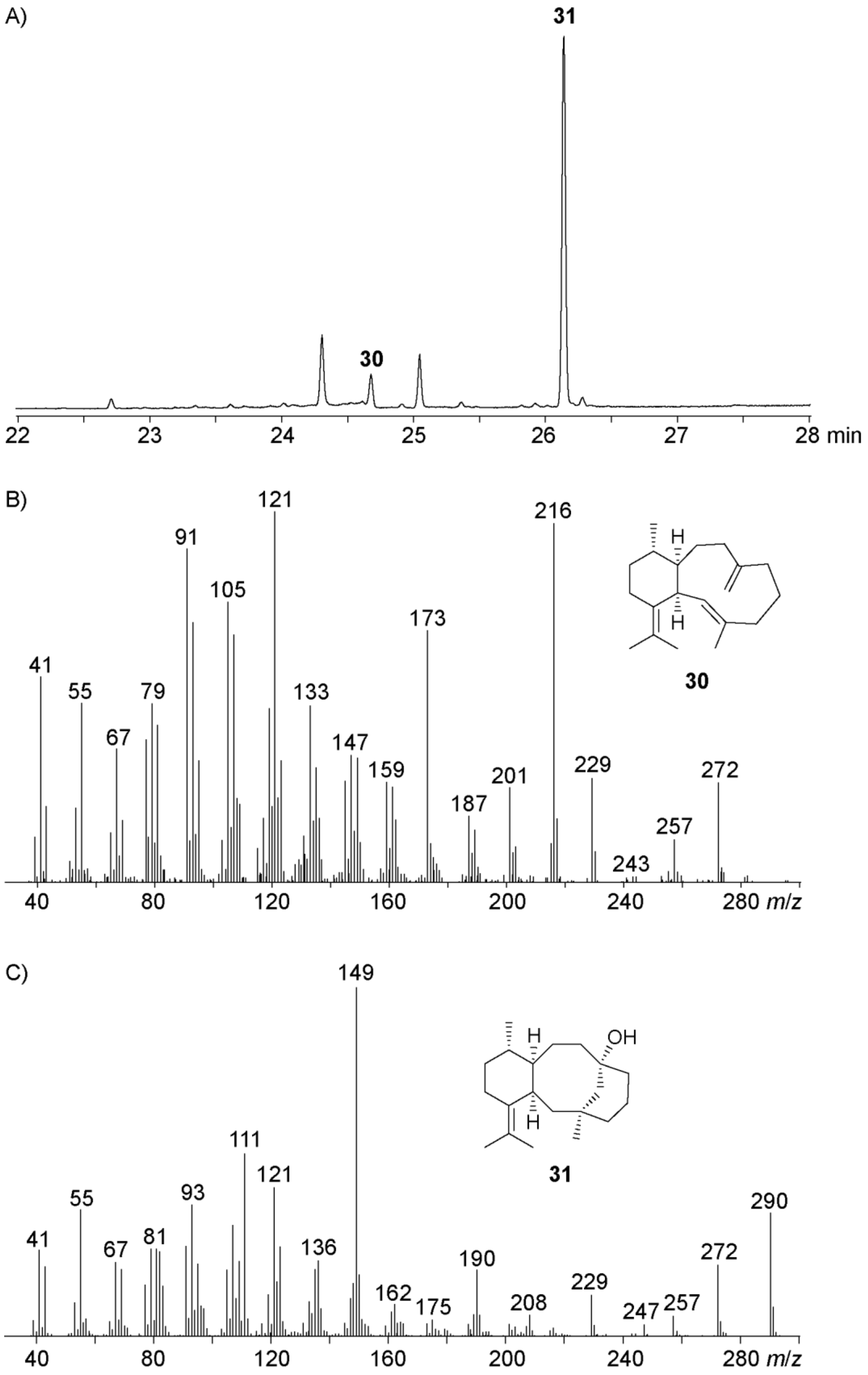

Figure S190. Product mixture formed from iso-GGPP I with CaCS. A) Total ion chromatogram of the crude extract from the enzyme incubation, B) EI mass spectrum of **30**, C) EI mass spectrum of **31**.

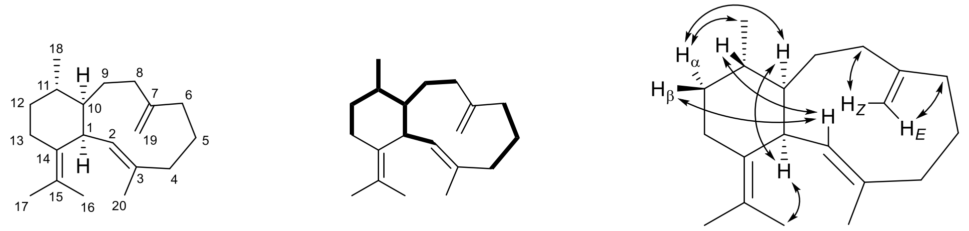

30

Figure S191. Structure elucidation of precatenulixenol (**30**). Bold: ^{1}H,^{1}H-COSY, single-headed arrows: key HMBC, and double-headed arrows: key NOESY correlations.

Table S23. NMR data of precatenulixenol (**30**) in C_6D_6 recorded at 298 K.

C[a]	type	^{13}C[b]	^{1}H[b]
1	CH	44.39	3.60 (dd, ^{3}J = 11.2, 4.8)
2	CH	126.67	5.54 (d, ^{3}J = 11.2)
3	C_q	133.15	–
4	CH_2	40.97	2.02 (m)
			1.98 (m)
5	CH_2	28.99	1.76 (m, 2H)
6	CH_2	38.05	2.34 (m)
			1.62 (m)
7	C_q	153.58	–
8	CH_2	31.38	2.13 (m)
			2.00 (m)
9	CH_2	30.56	2.05 (m)
			1.34 (m)
10	CH	49.31	1.34 (m)
11	CH	29.31	1.72 (m)
12	CH_2	36.86	1.71 (m, H_β)
			1.00 (m, H_α)
13	CH_2	26.72	2.57 (m)
			1.95 (m)
14	C_q	134.91	–
15	C_q	120.04	–
16	CH_3	20.48	1.74 (d, ^{4}J = 2.0)
17	CH_3	20.49	1.64 (d, ^{4}J = 1.1)
18	CH_3	20.68	0.83 (d, ^{3}J = 6.3)
19	CH_2	109.77	4.90 (br s, H_E)
			4.87 (br s, H_Z)
20	CH_3	17.34	1.71 (d, ^{4}J = 1.2)

[a] Carbon numbering as shown in Figure S191. [b] Chemical shifts δ in ppm, multiplicity: s = singlet, d = doublet, m = multiplet, br = broad, coupling constants J are given in Hertz.

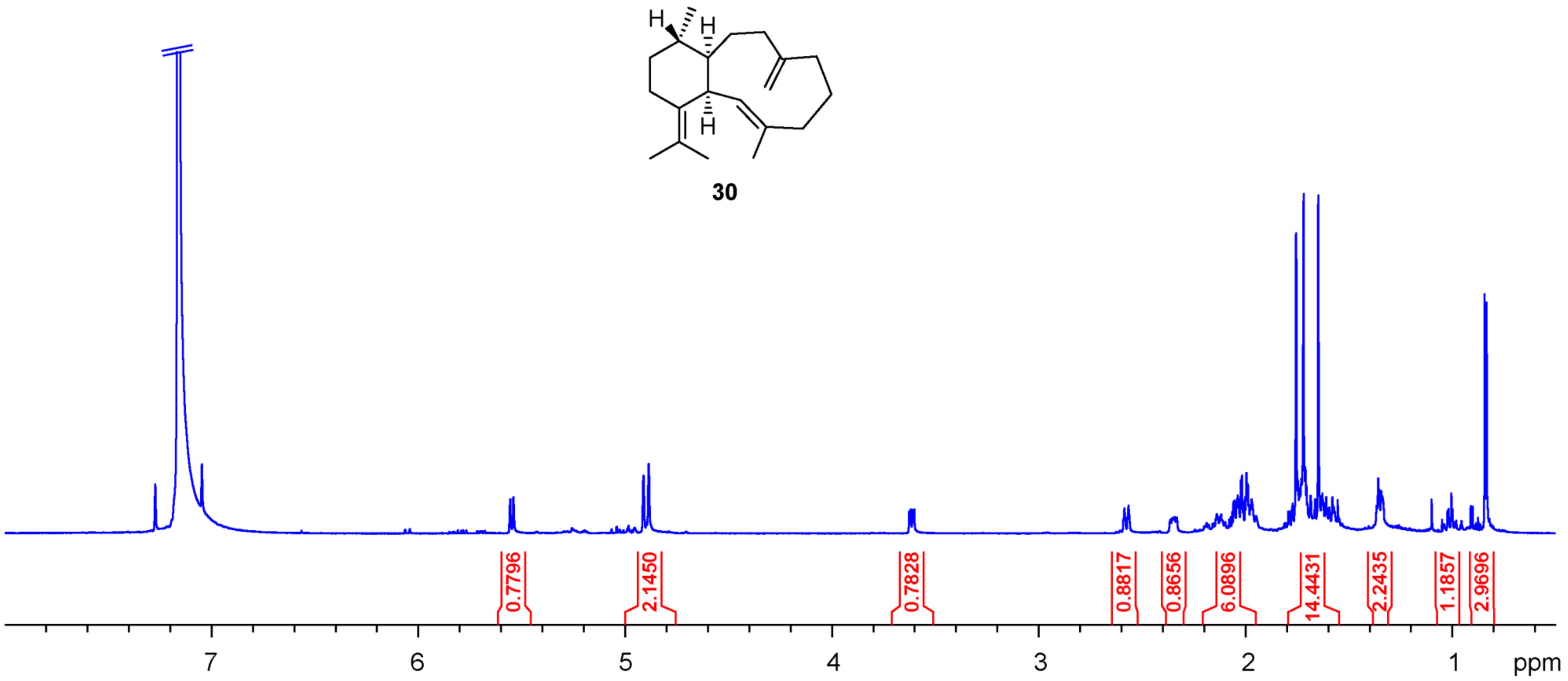

Figure S192. ^{1}H-NMR spectrum (700 MHz, C_6D_6) of **30**.

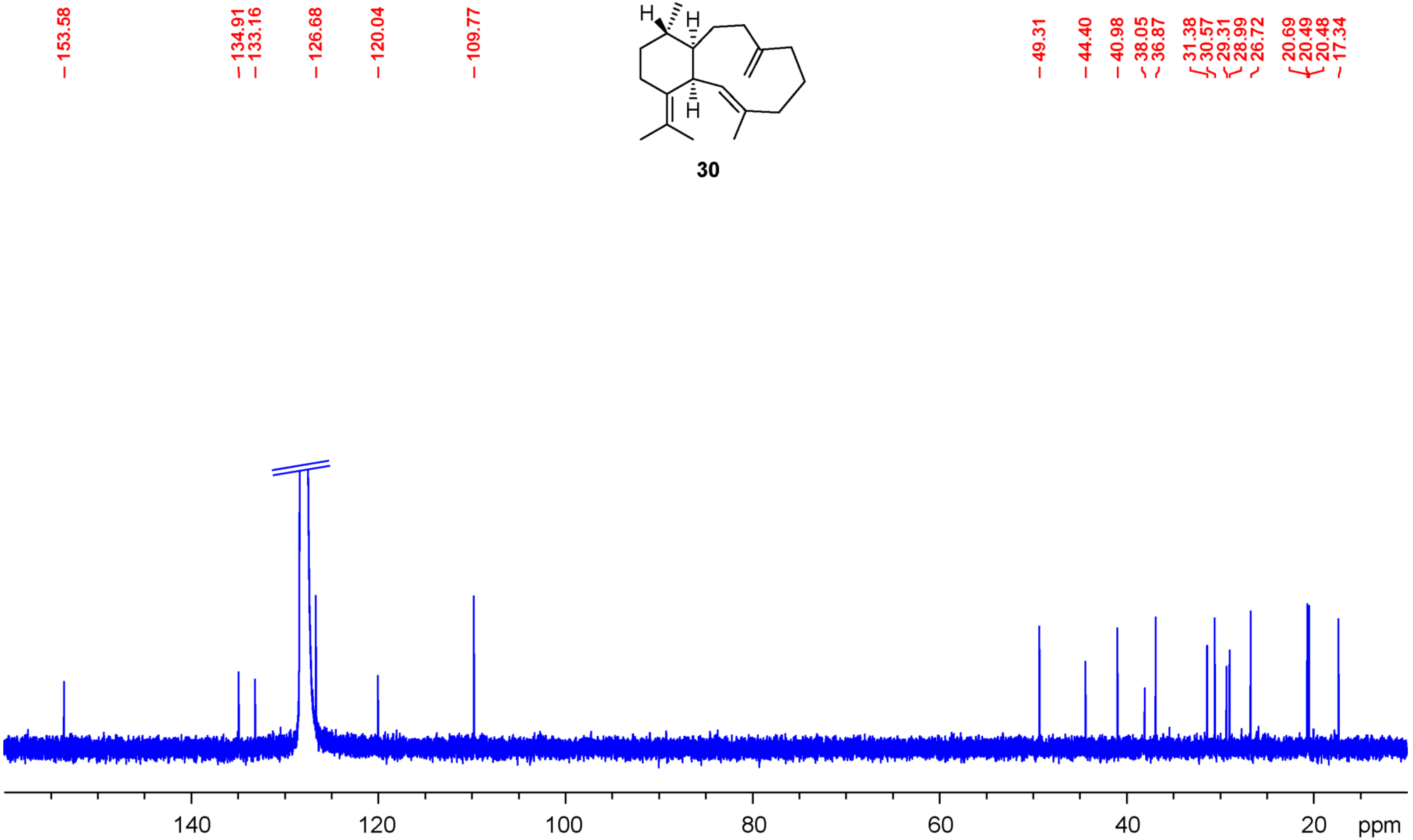

Figure S193. ^{13}C-NMR spectrum (176 MHz, C_6D_6) of **30**.

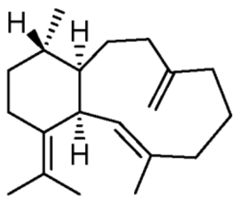

30

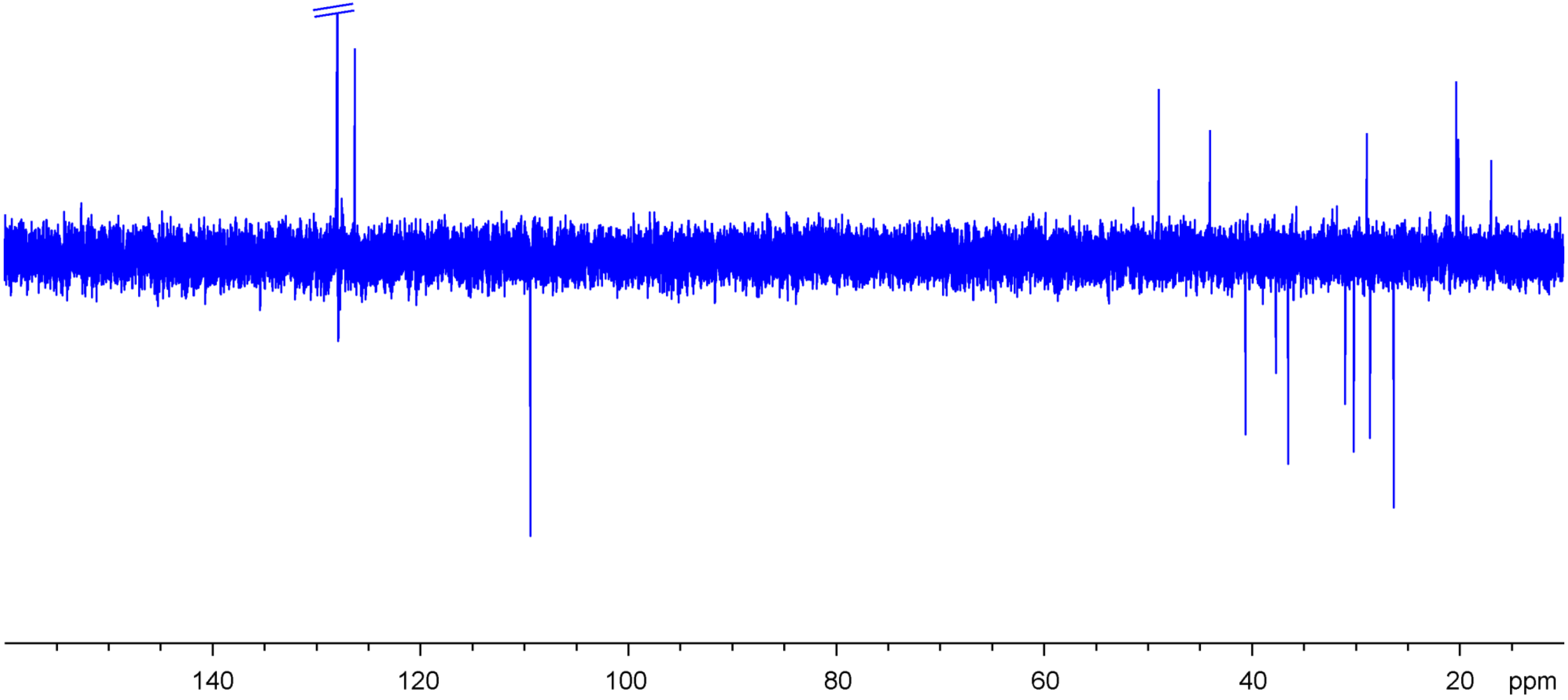

Figure S194. ^{13}C-DEPT135 spectrum (176 MHz, C_6D_6) of **30**.

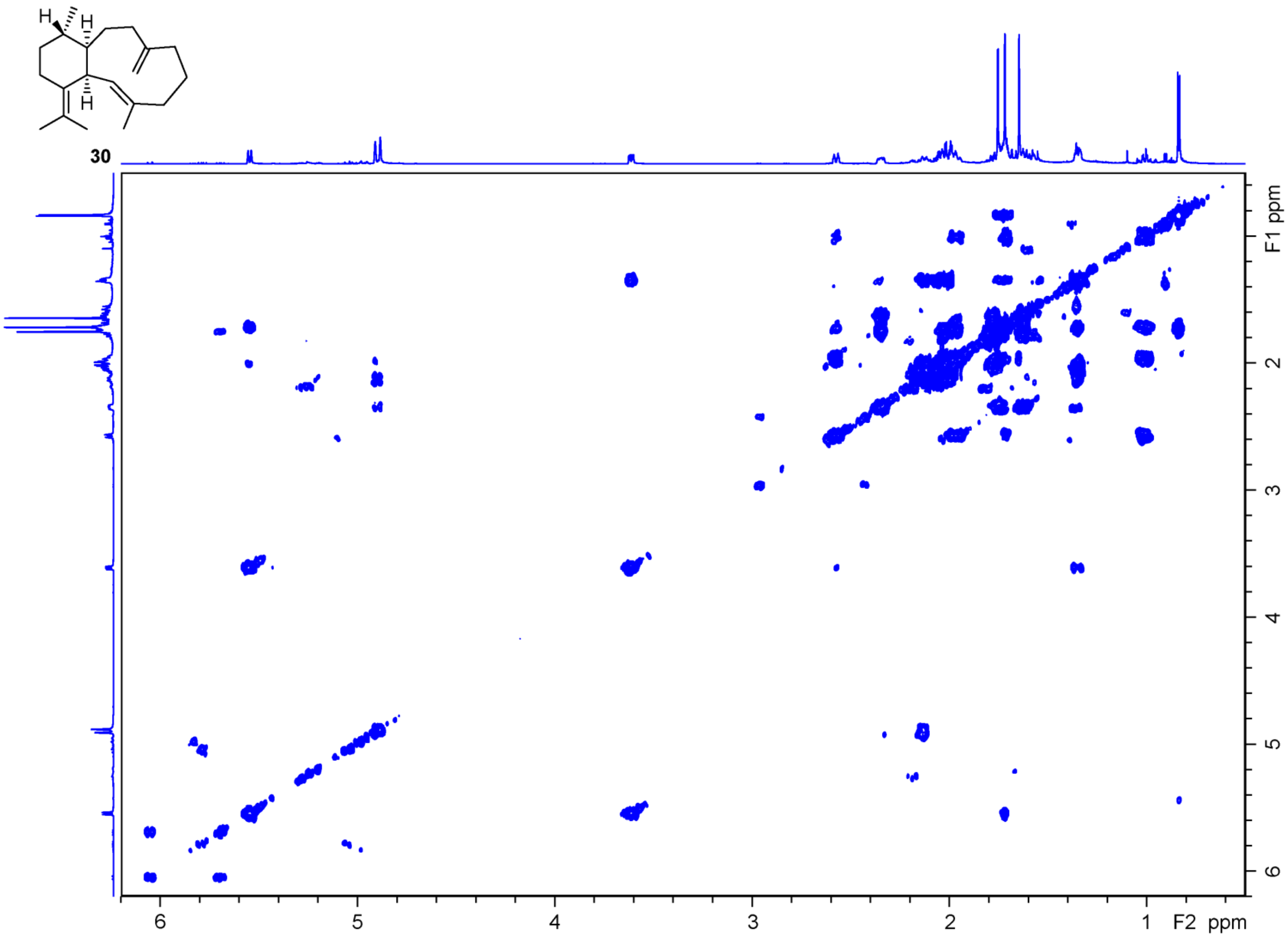

Figure S195. $^{1}H,^{1}H$-COSY spectrum (C_6D_6) of **30**.

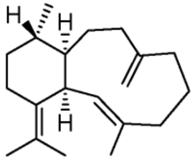

30

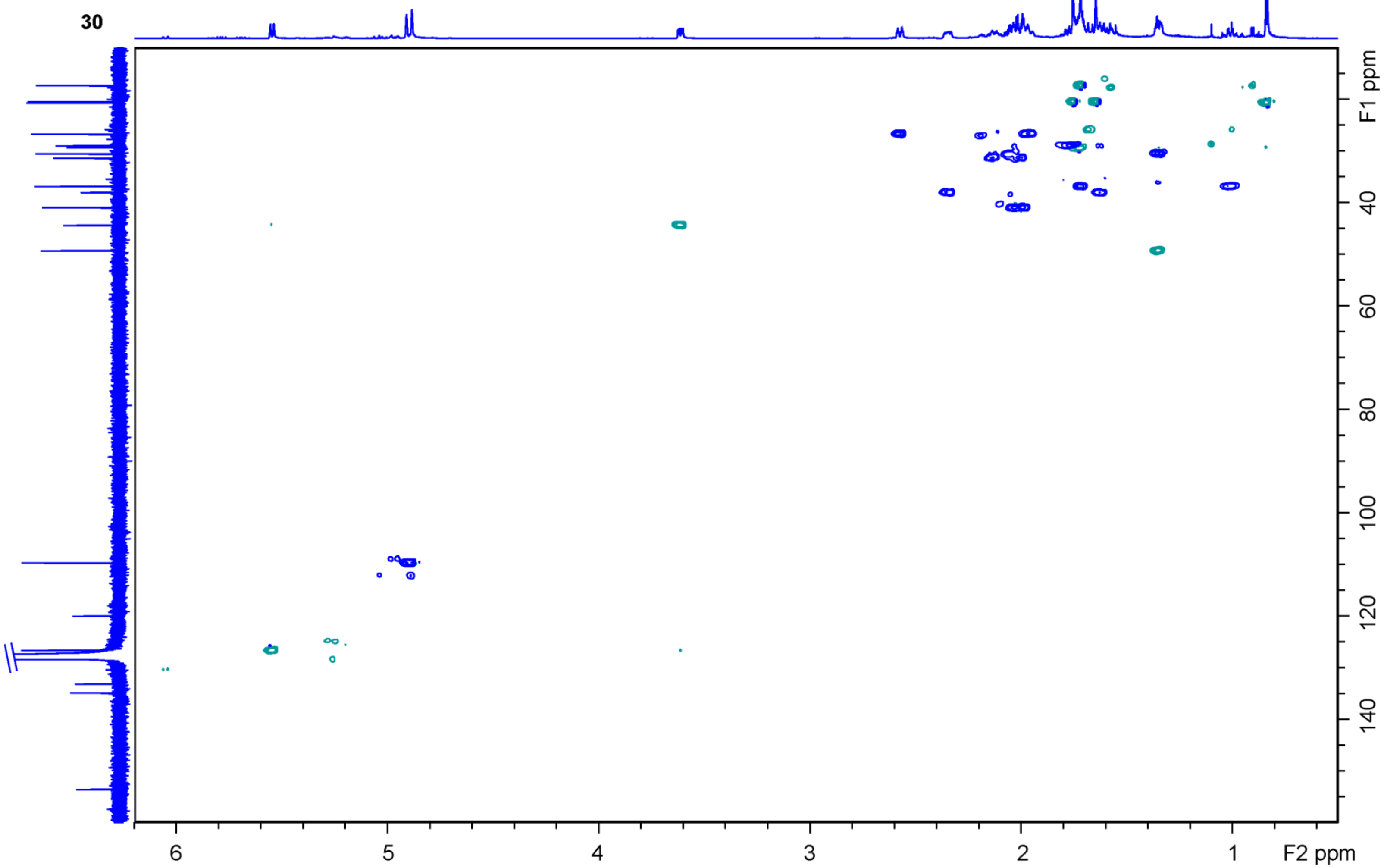

Figure S196. HSQC spectrum (C_6D_6) of **30**.

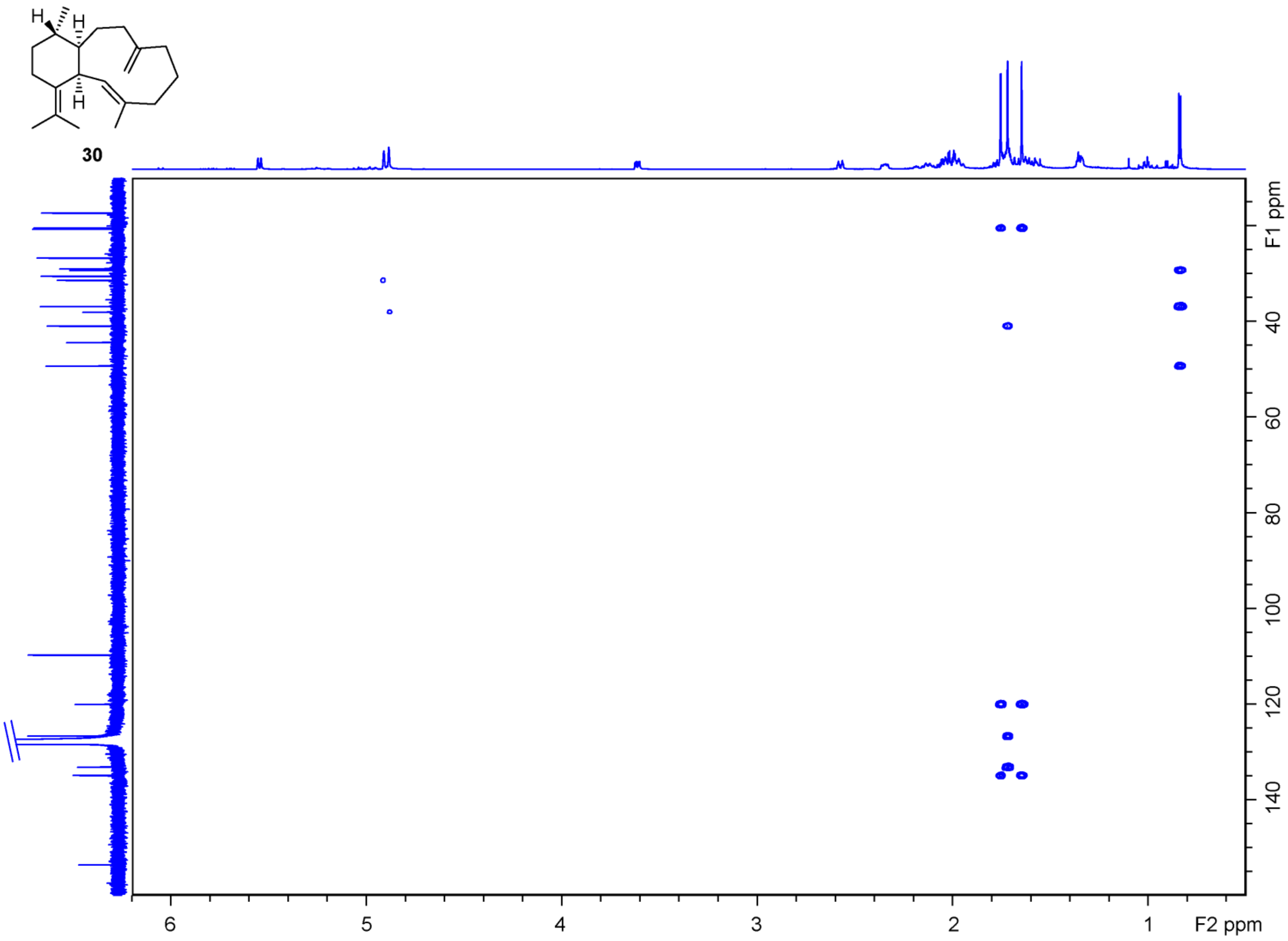

Figure S197. HMBC spectrum (C_6D_6) of **30**.

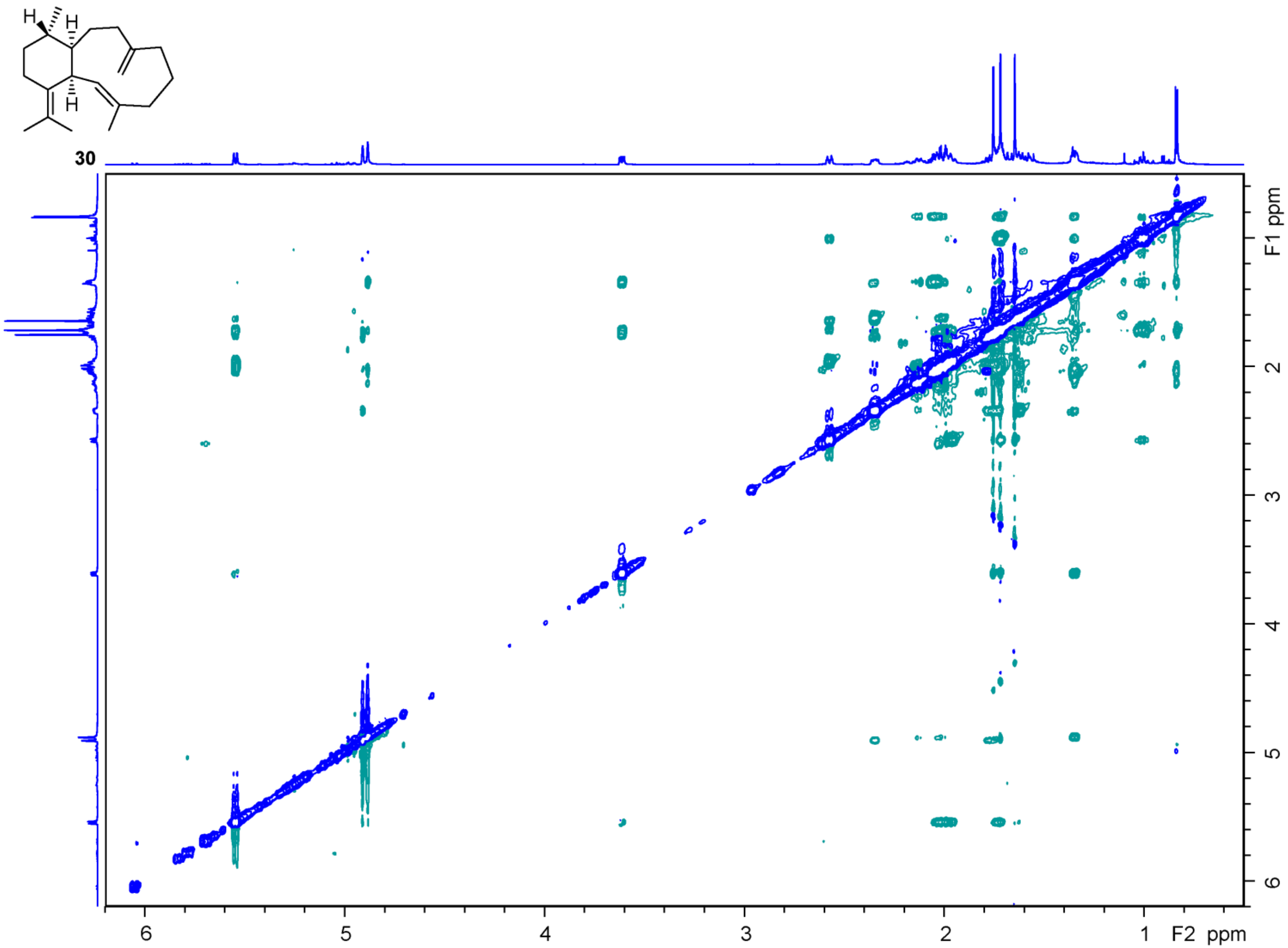

Figure S198. NOESY spectrum (C_6D_6) of **30**.

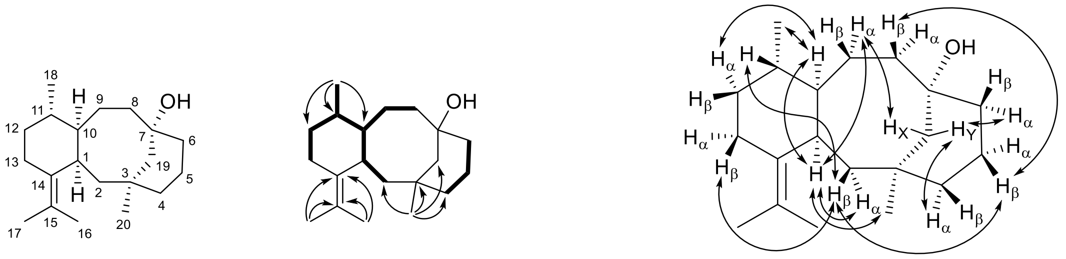

31

Figure S199. Structure elucidation of catenulixenol (**31**). Bold: ^{1}H,^{1}H-COSY, single-headed arrows: key HMBC, and double-headed arrows: key NOESY correlations.

Table S24. NMR data of catenulixenol (**31**) in C_6D_6 recorded at 298 K.

C[a]	type	^{13}C[b]	^{1}H[b]
1	CH	38.48	2.75 (br d, ^3J = 6.8)
2	CH_2	38.07	2.00 (dd, ^2J = 14.9, ^3J = 7.5, H_β)
			0.61 (d, ^2J = 14.9, H_α)
3	C_q	34.22	–
4	CH_2	39.46	1.15 (m, H_β)
			0.99 (m, H_α)
5	CH_2	21.25	1.44 (m, 2H)
6	CH_2	41.21	1.54 (m, H_β)
			1.22 (m, H_α)
7	C_q	72.05	–
8	CH_2	30.70	1.76 (dd, ^2J = 15.5, ^3J = 9.0, H_β)
			1.22 (m, H_α)
9	CH_2	23.97	1.81 (m, H_β)
			1.54 (m, H_α)
10	CH	47.22	1.17 (m)
11	CH	28.82	1.70 (m)
12	CH_2	37.40	1.66 (m, H_β)
			0.96 (m, H_α)
13	CH_2	26.69	2.48 (dm, ^2J = 13.8, H_α)
			1.78 (m, H_β)
14	C_q	139.51	–
15	C_q	116.42	–
16	CH_3	20.20	1.74 (d, ^4J = 1.8)
17	CH_3	20.18	1.63 (d, ^4J = 1.0)
18	CH_3	20.10	0.81 (d, ^3J = 6.3)
19	CH_2	44.92	2.03 (ddd, ^2J = 13.5, ^4J = 2.4, 2.4, H_X)
			0.75 (ddd, ^2J = 13.6, ^4J = 1.4, 1.4, H_Y)
20	CH_3	32.46	0.88 (d, ^4J = 0.6)

[a] Carbon numbering as shown in Figure S199. [b] Chemical shifts δ in ppm, multiplicity: s = singlet, d = doublet, m = multiplet, coupling constants J are given in Hertz.

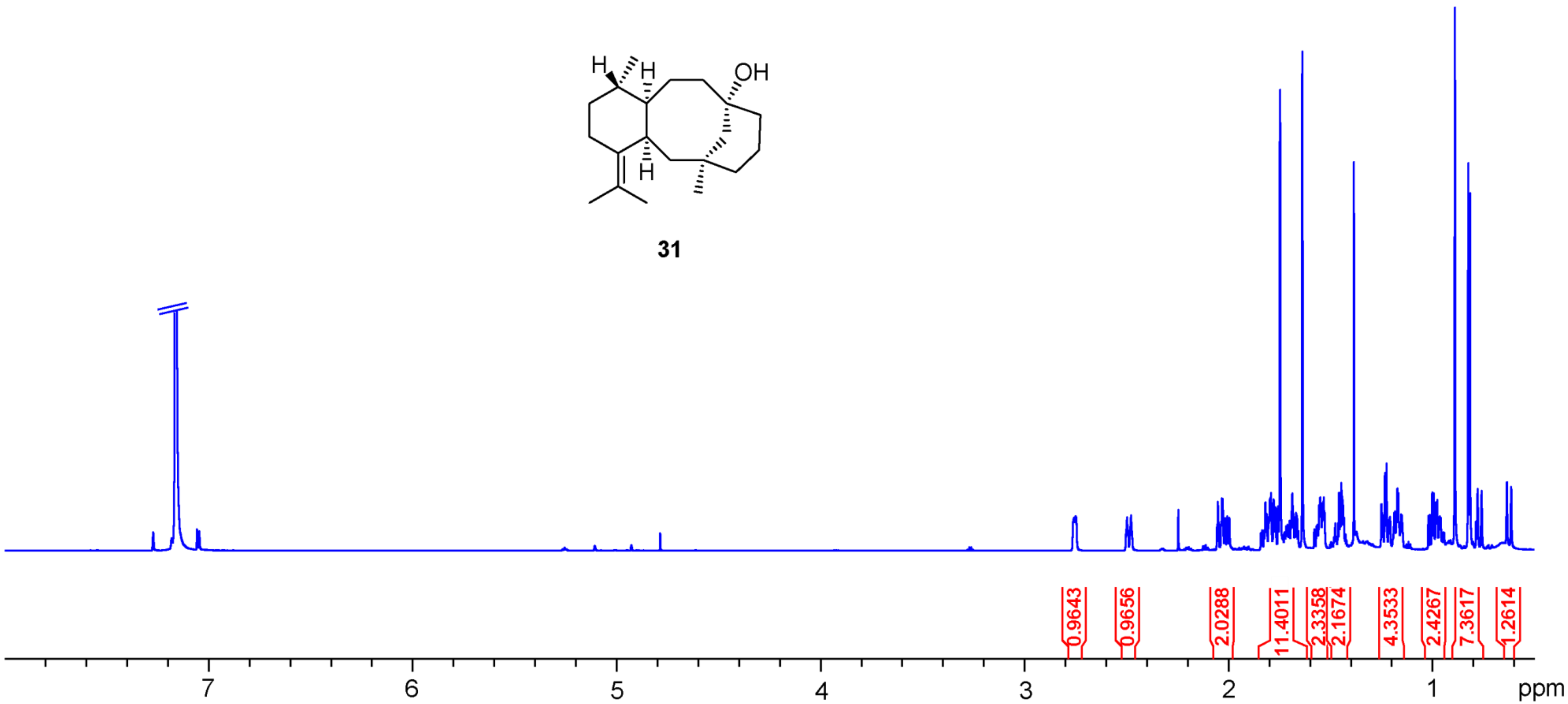

Figure S200. ^{1}H-NMR spectrum (700 MHz, C_6D_6) of **31**.

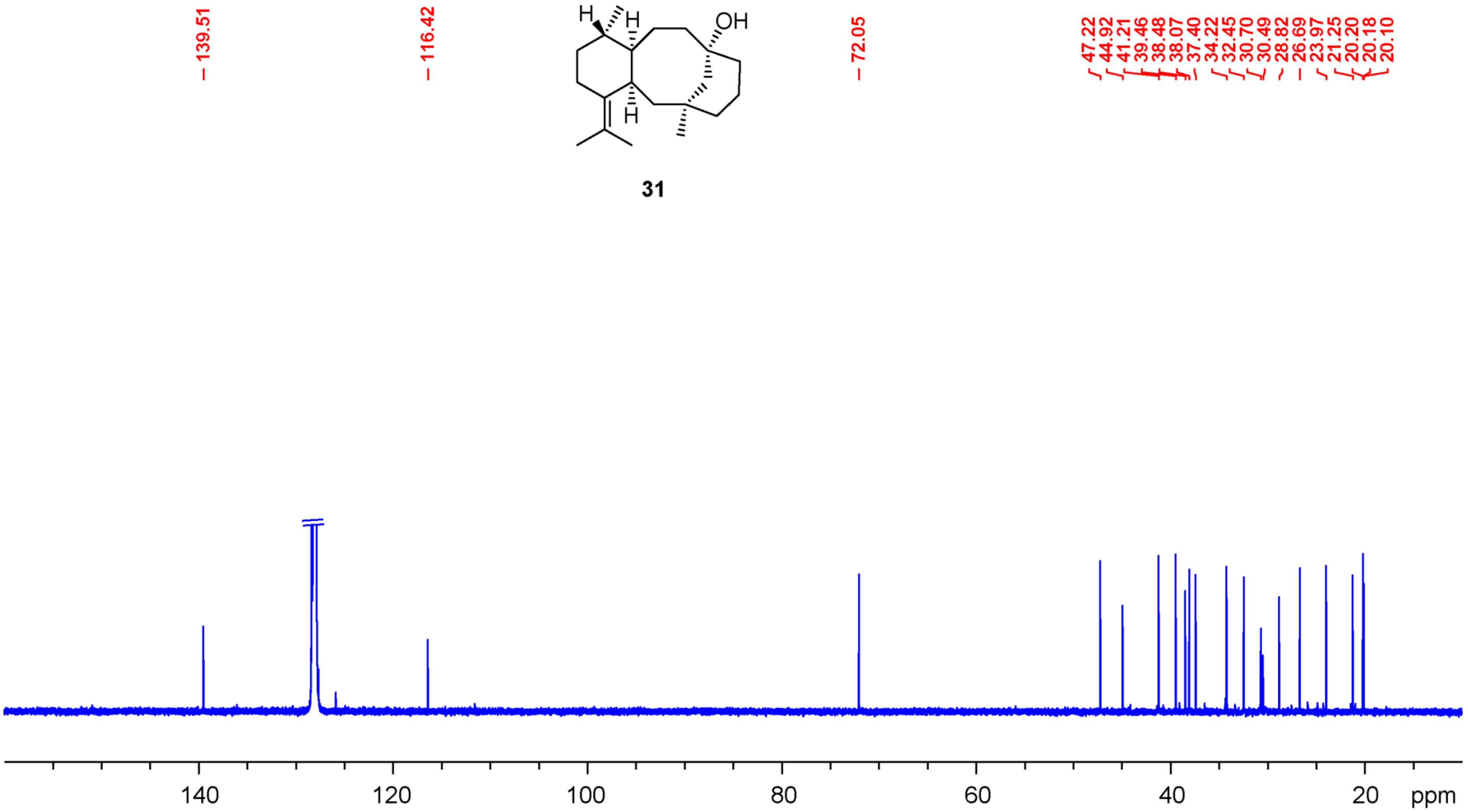

Figure S201. ^{13}C-NMR spectrum (176 MHz, C_6D_6) of **31**.

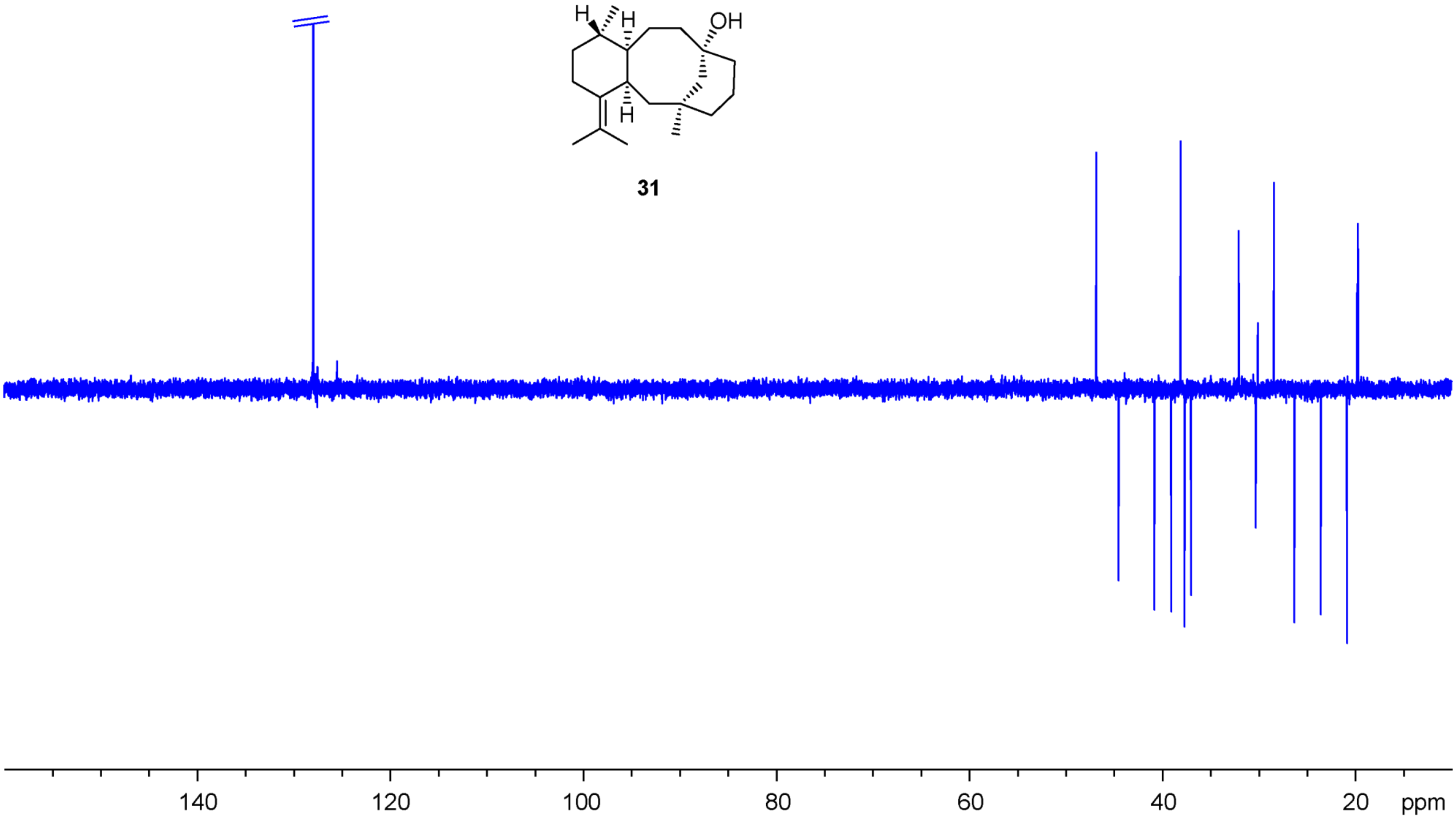

Figure S202. ^{13}C-DEPT135 spectrum (176 MHz, C_6D_6) of **31**.

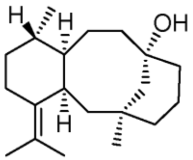

31

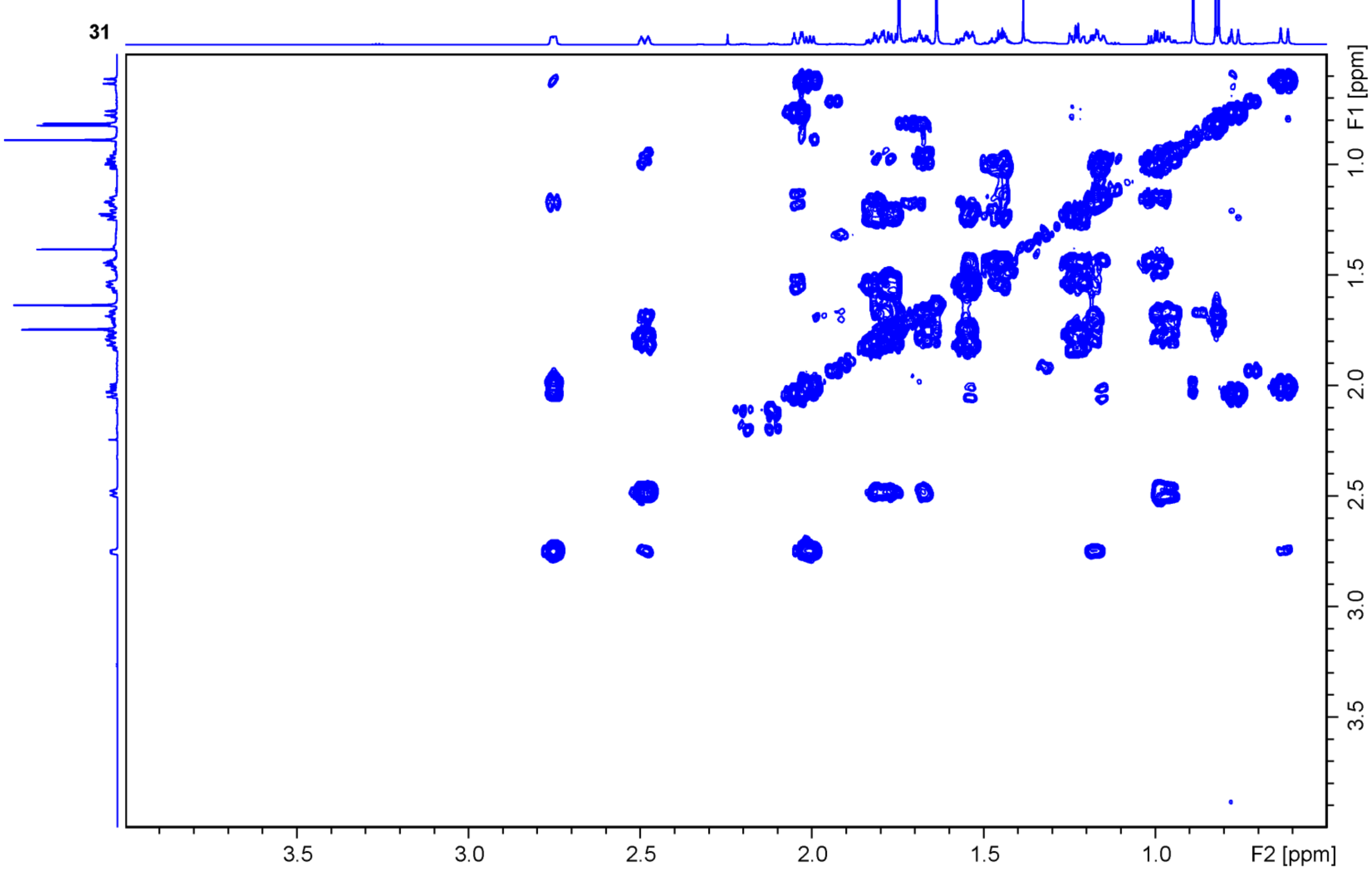

Figure S203. $^{1}H,^{1}H$-COSY spectrum (C_6D_6) of **31**.

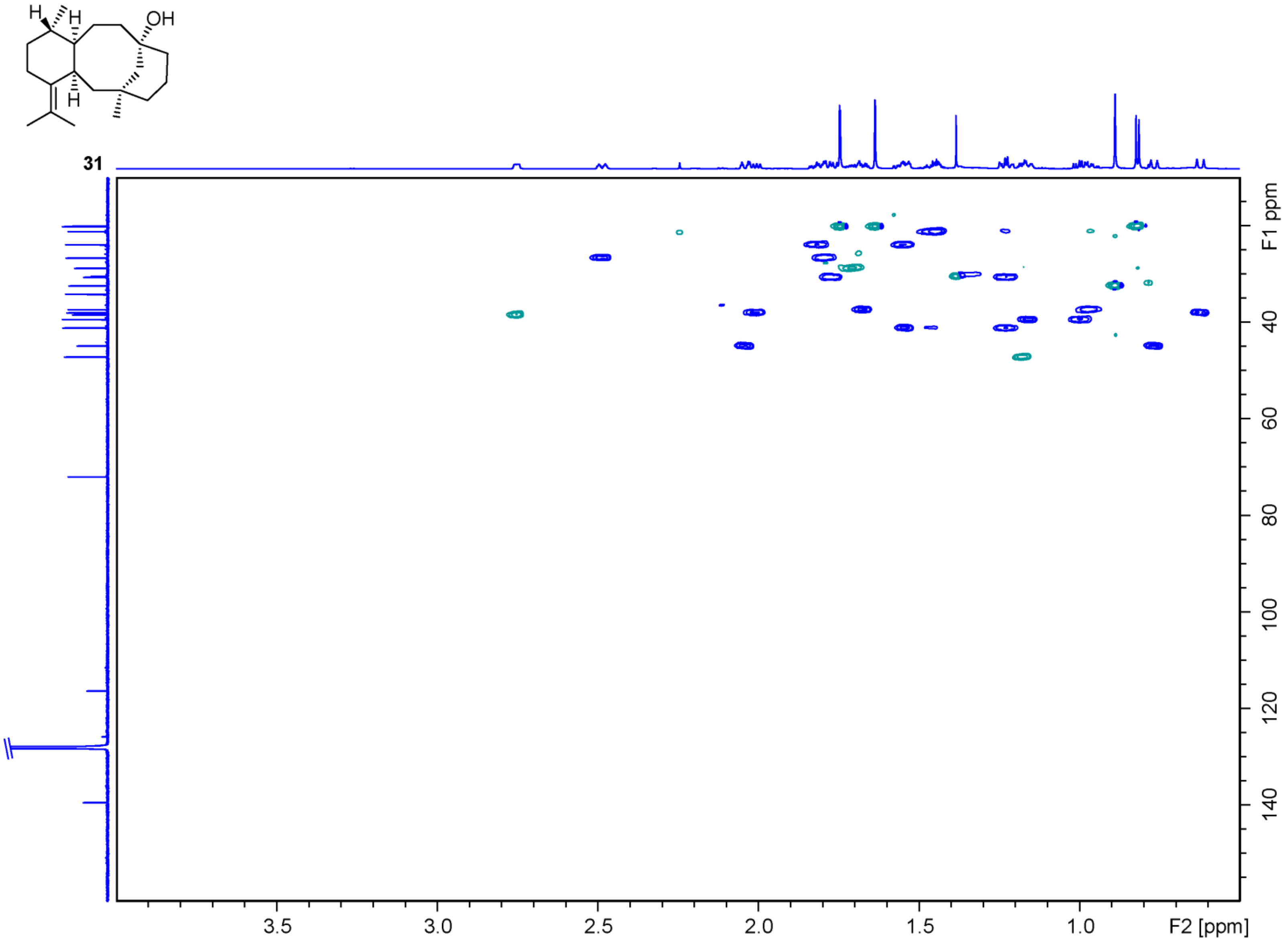

Figure S204. HSQC spectrum (C_6D_6) of **31**.

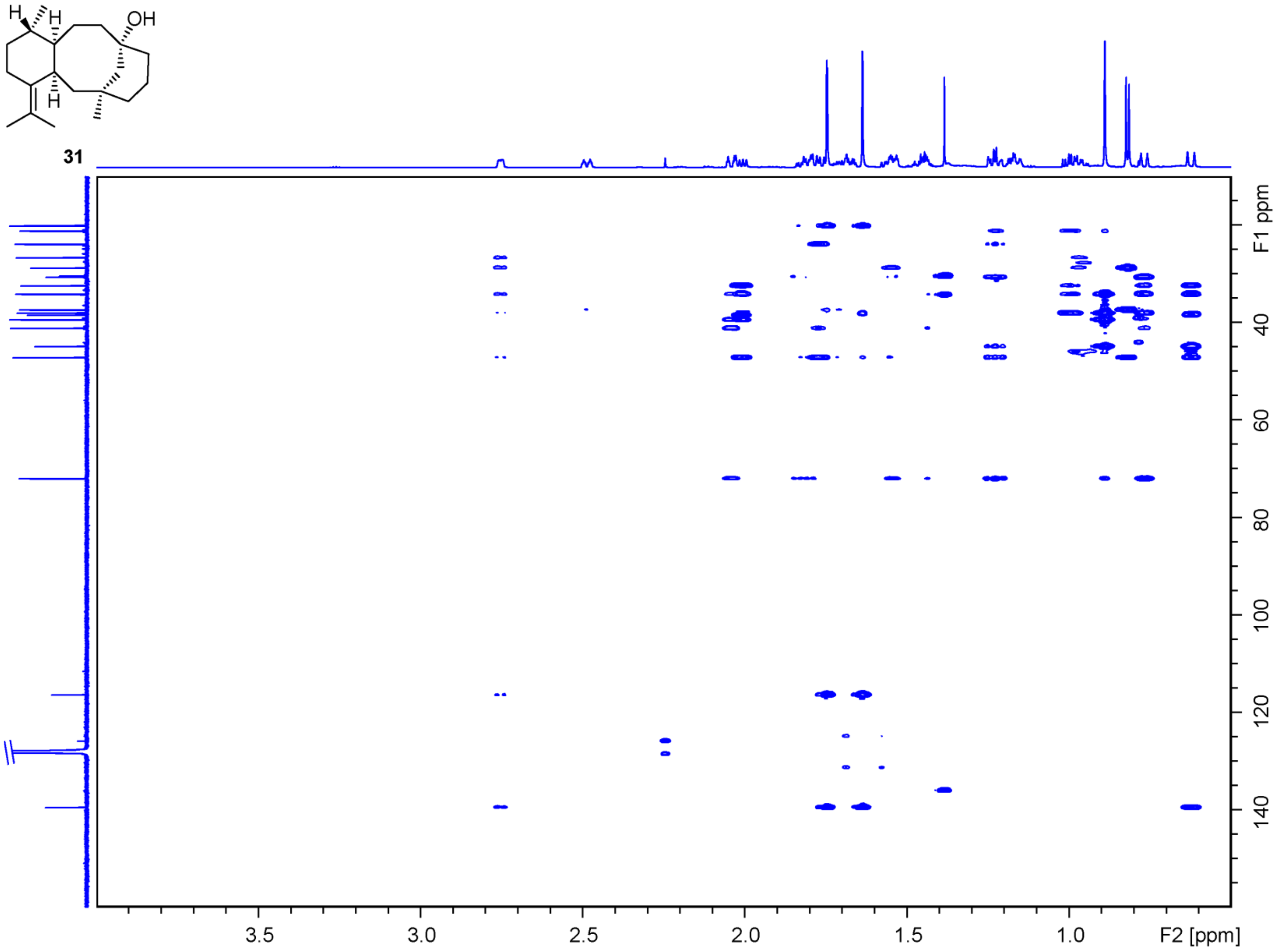

Figure S205. HMBC spectrum (C_6D_6) of **31**.

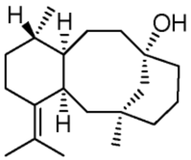

31

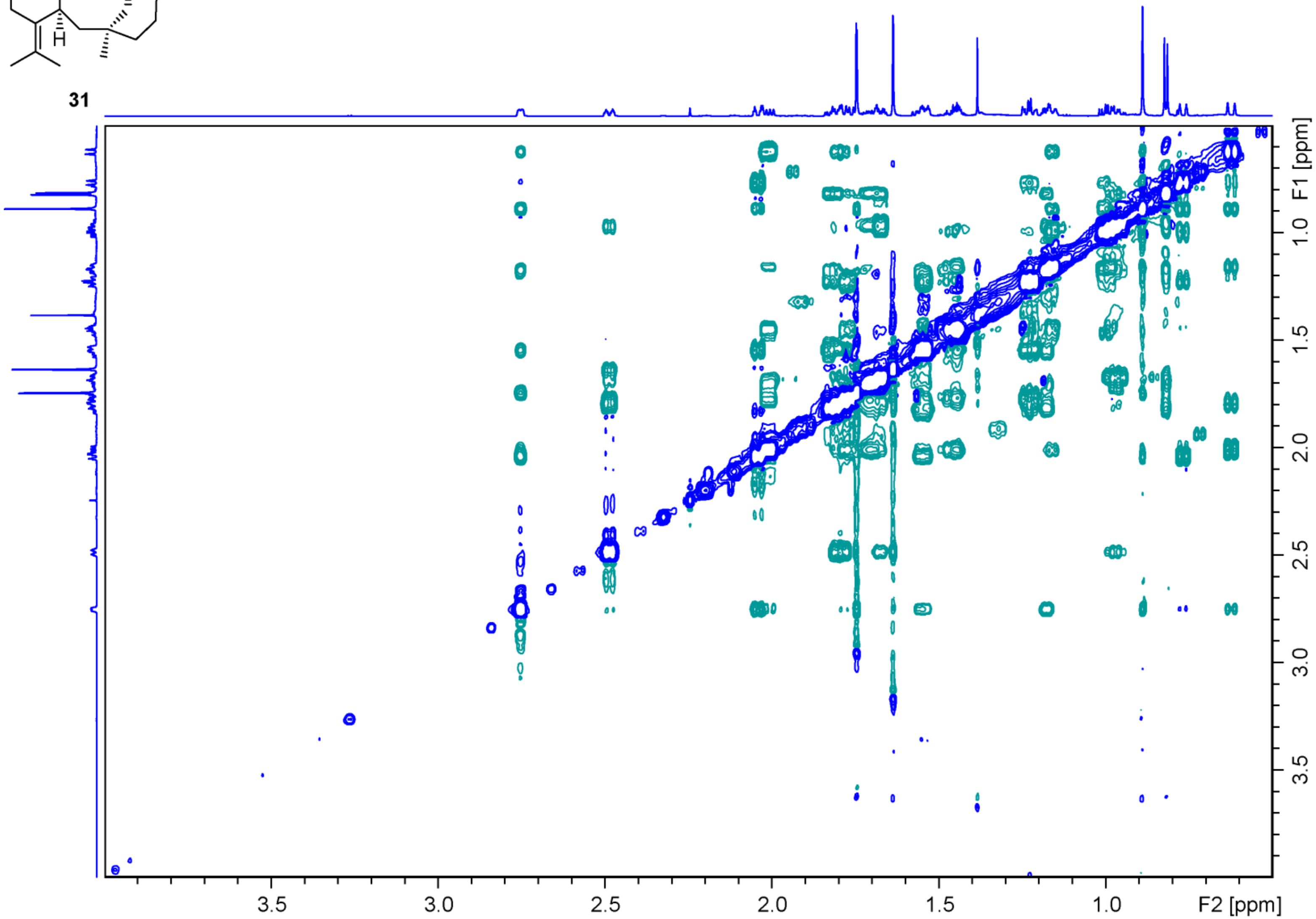

Figure S206. NOESY spectrum (C_6D_6) of **31**.

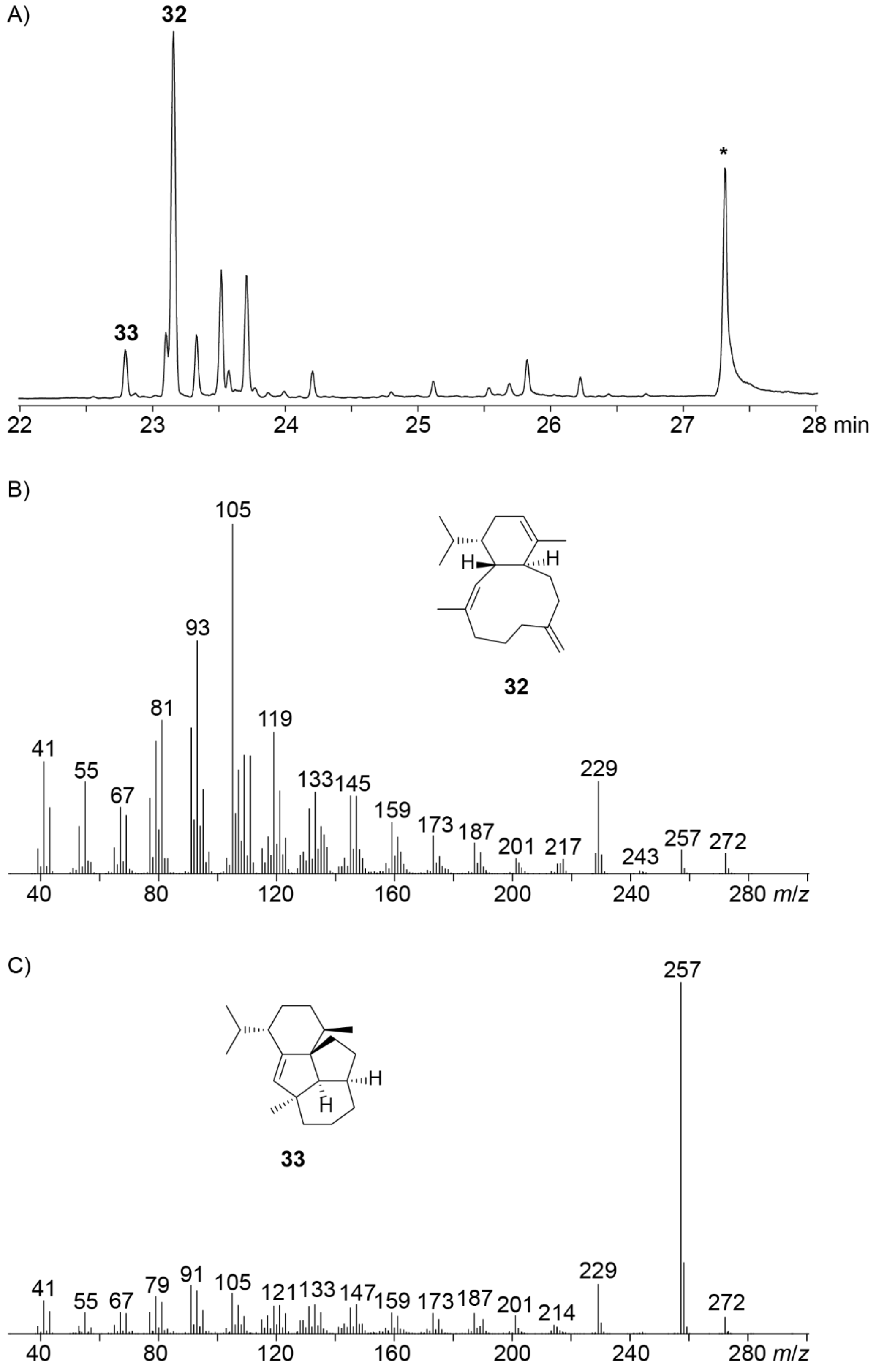

Figure S207. Product mixture formed from iso-GGPP I with CwWS. A) Total ion chromatogram of the crude extract from the enzyme incubation, B) EI mass spectrum of **32**, C) EI mass spectrum of **33**.

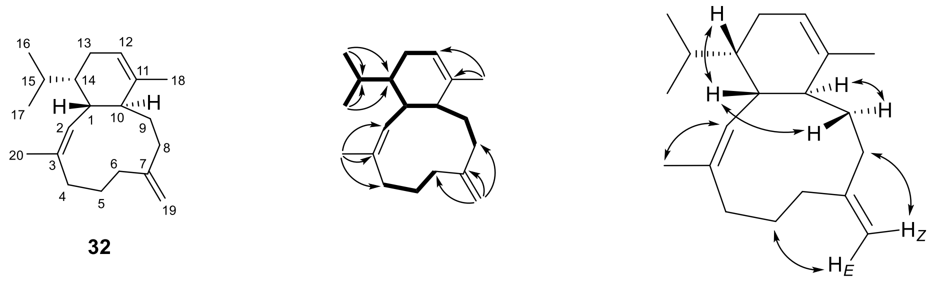

32

Figure S208. Structure elucidation of prewanjuxenene (**32**). Bold: ^{1}H,^{1}H-COSY, single-headed arrows: key HMBC, and double-headed arrows: key NOESY correlations.

Table S25. NMR data of prewanjuxenene (**32**) in C_6D_6 recorded at 298 K.

C[a]	type	^{13}C[b]	^{1}H[b]
1	CH	39.65	2.86 (m)
2	CH	128.41	5.44 (br d, ^{3}J = 11.6)
3	C_q	134.36	–
4	CH_2	28.87	2.84 (m)
			1.64 (ddd, ^{2}J = 13.1, ^{3}J = 4.1, 4.1)
5	CH_2	23.64	1.83 (m)
			1.48 (m)
6	CH_2	33.60	2.19 (ddd, ^{2}J = 17.5, ^{3}J = 13.1, 2.4)
			2.01 (dm, ^{2}J = 17.5)
7	C_q	149.74	–
8	CH_2	38.66	2.44 (ddd, ^{2}J = 12.5, ^{3}J = 3.4, 3.4)
			1.74 (dd, ^{2}J = 12.6, ^{3}J = 4.5)
9	CH_2	34.67	1.79 (m)
			1.43 (m)
10	CH	48.34	1.95 (m)
11	C_q	134.96	–
12	CH	122.78	5.52 (m)
13	CH_2	26.48	1.97 (m)
			1.79 (m)
14	CH	40.40	1.22 (m)
15	CH	30.04	1.45 (m)
16	CH_3	22.62	0.98 (d, ^{3}J = 6.5)
17	CH_3	21.13	0.87 (d, ^{3}J = 6.5)
18	CH_3	22.36	1.67 (s)
19	CH_2	108.52	5.02 (br s, H_Z)
			4.91 (br s, H_E)
20	CH_3	23.91	1.67 (s)

[a] Carbon numbering as shown in Figure S208. [b] Chemical shifts δ in ppm, multiplicity: s = singlet, d = doublet, m = multiplet, br = broad, coupling constants J are given in Hertz.

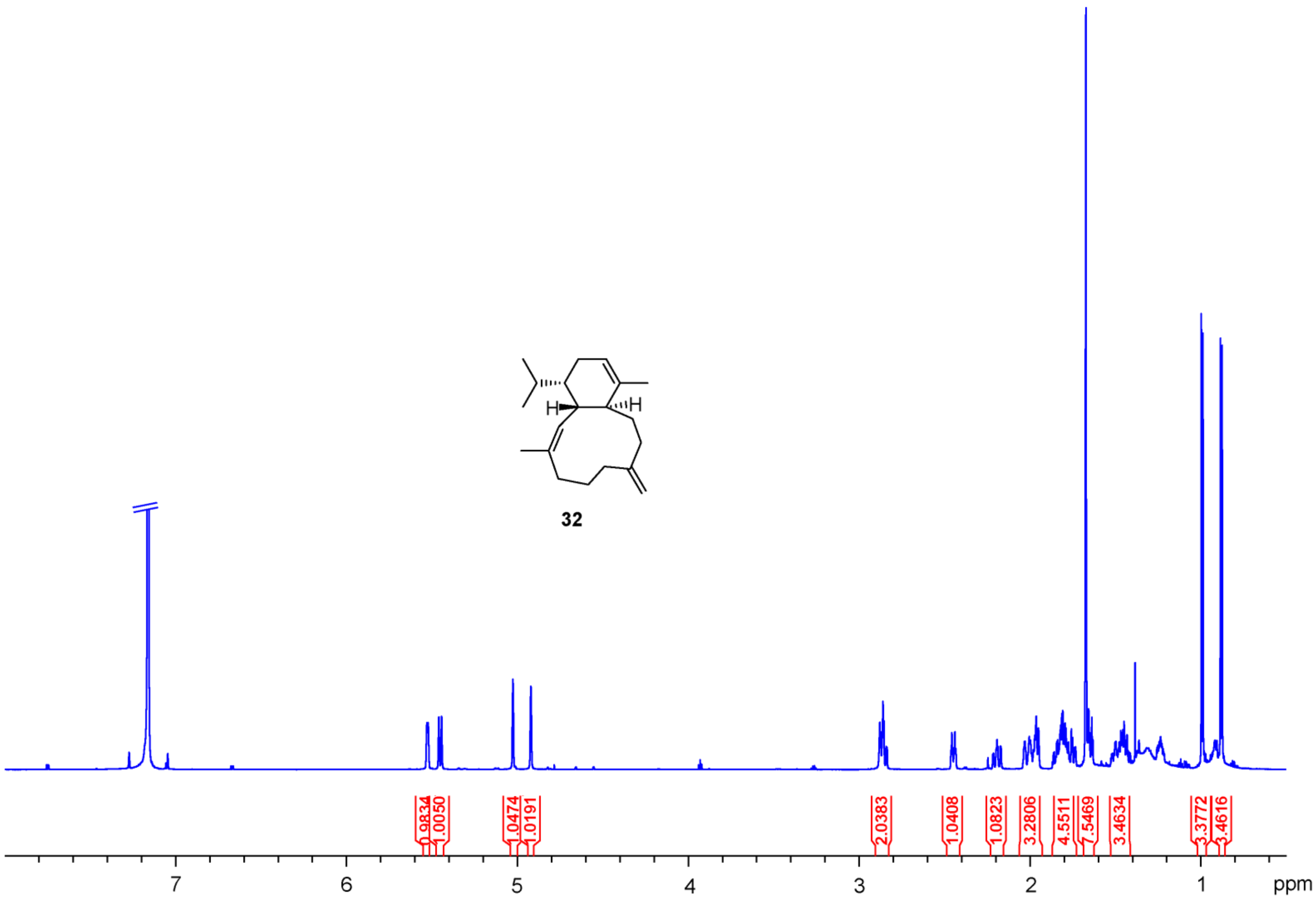

Figure S209. ^{1}H-NMR spectrum (700 MHz, C_6D_6) of **32**.

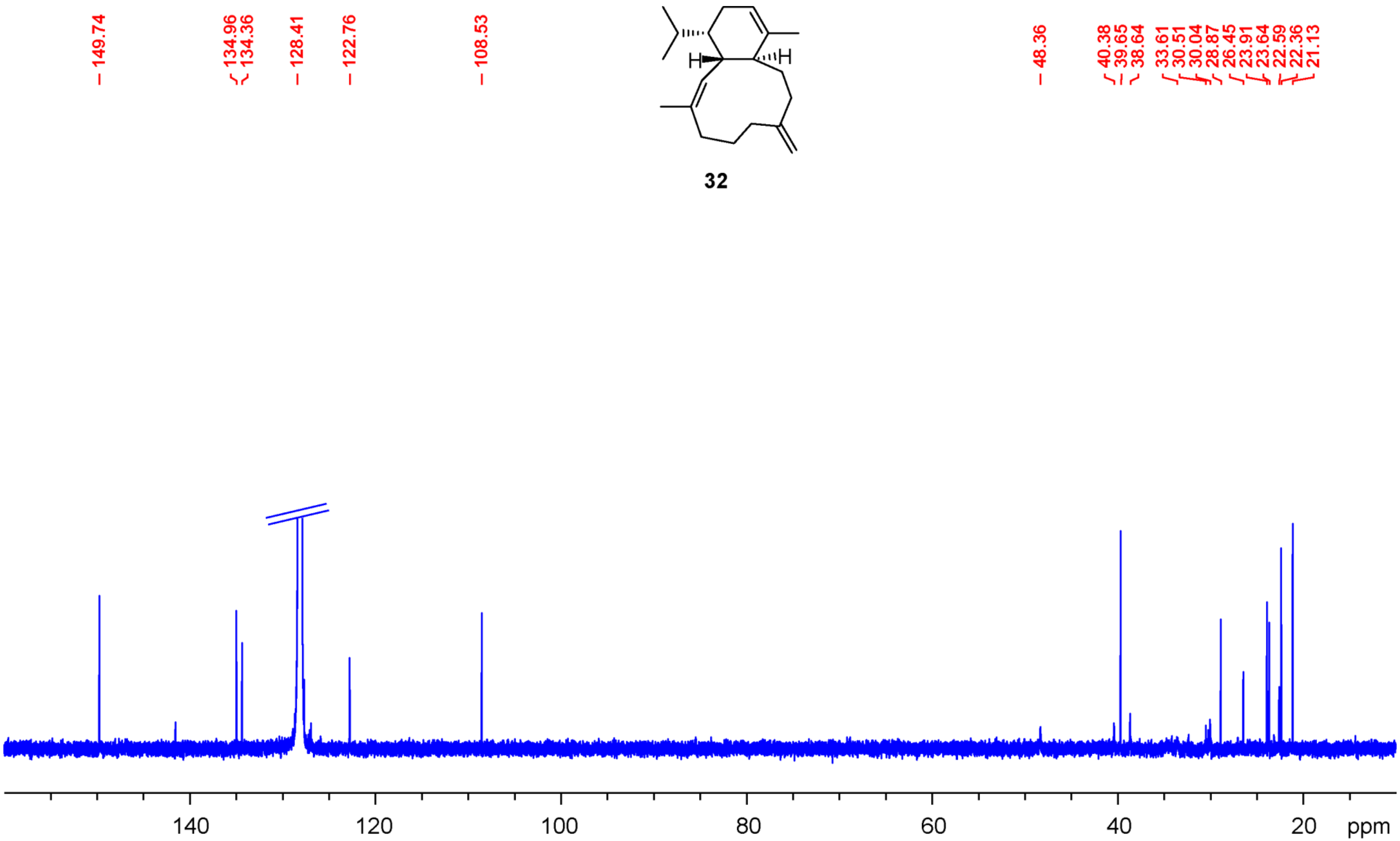

Figure S210. ^{13}C-NMR spectrum (176 MHz, C_6D_6) of **32**.

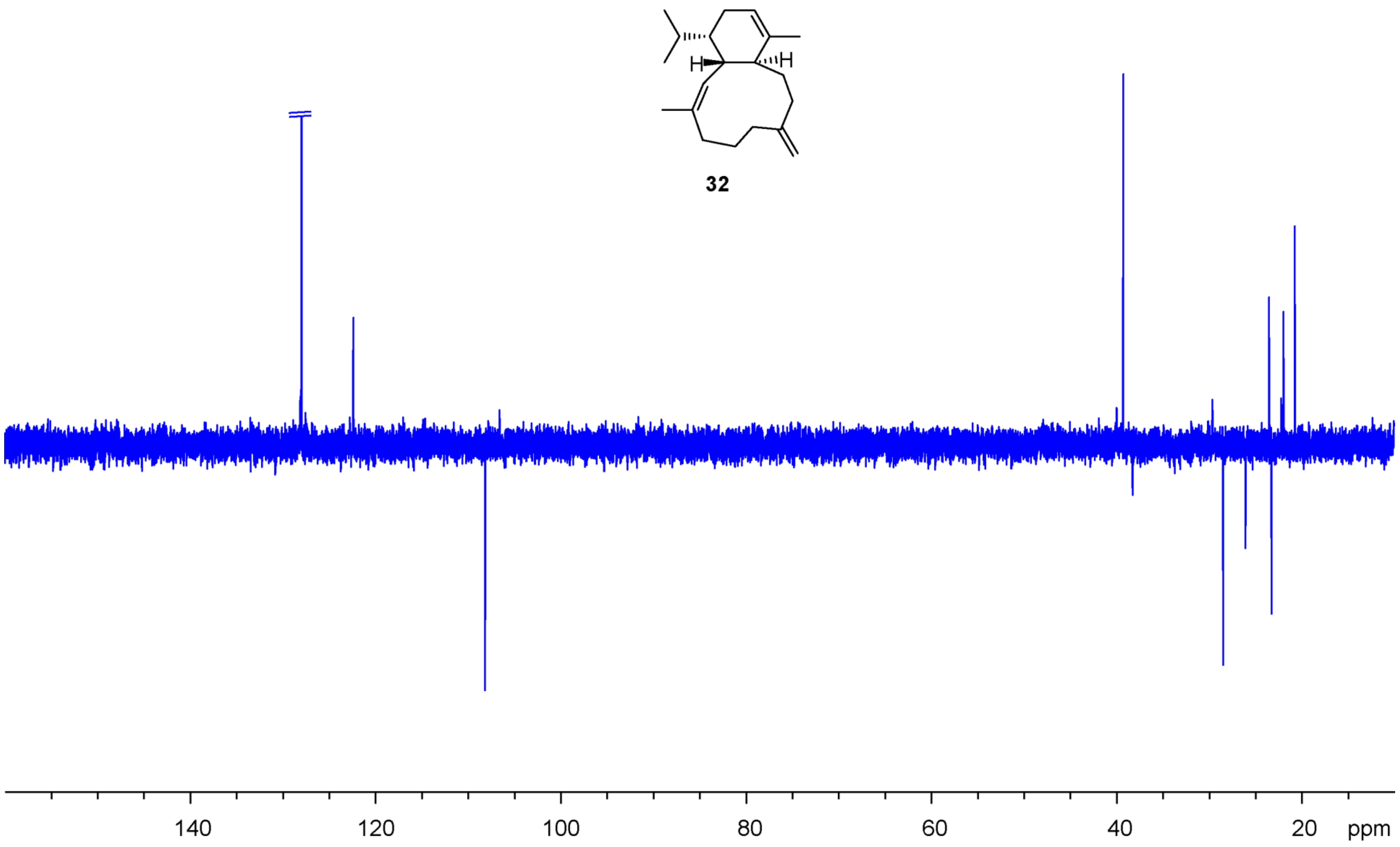

Figure S211. ^{13}C-DEPT135 spectrum (176 MHz, C_6D_6) of **32**.

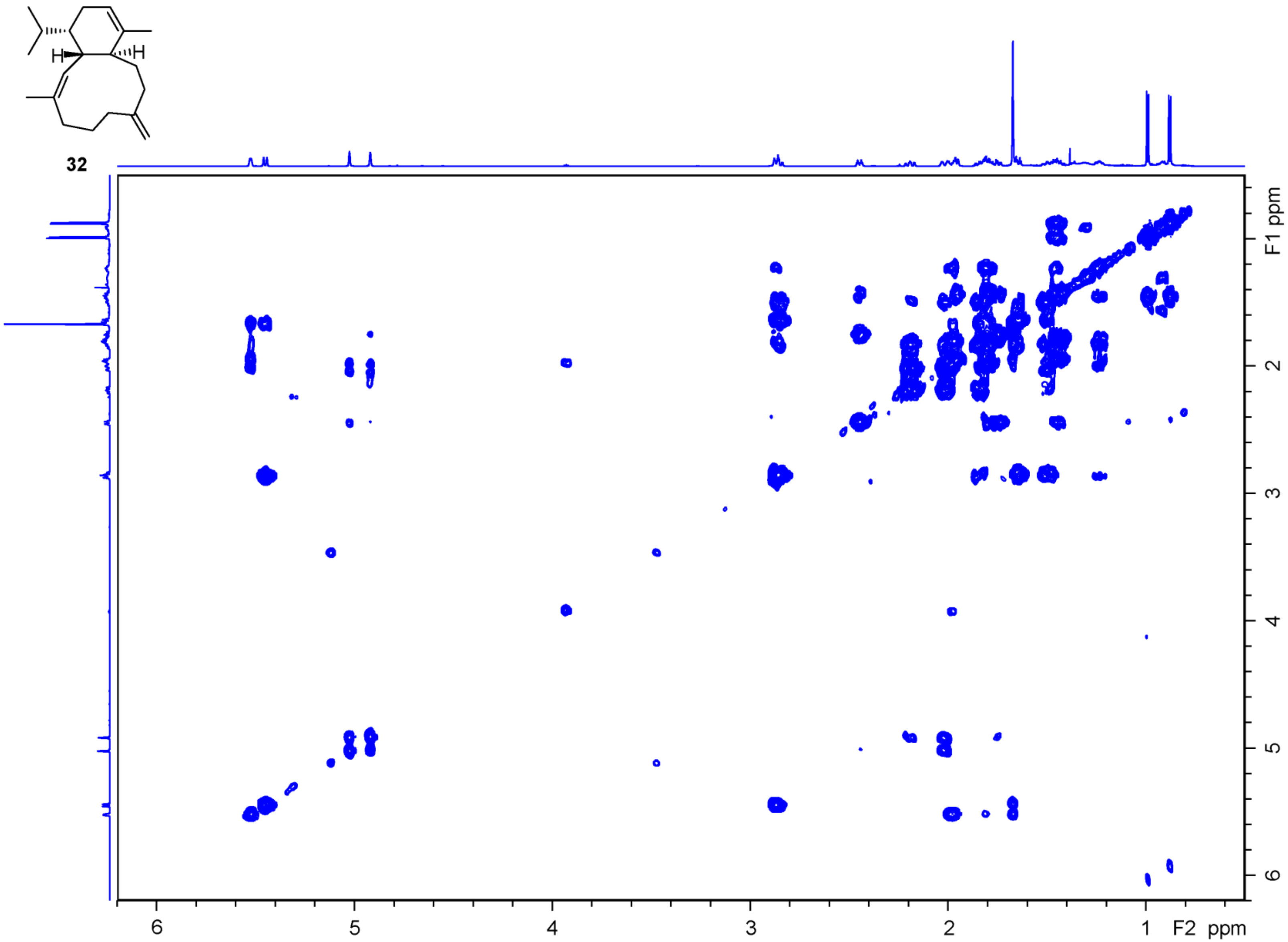

Figure S212. ^{1}H,^{1}H-COSY spectrum (C_6D_6) of **32**.

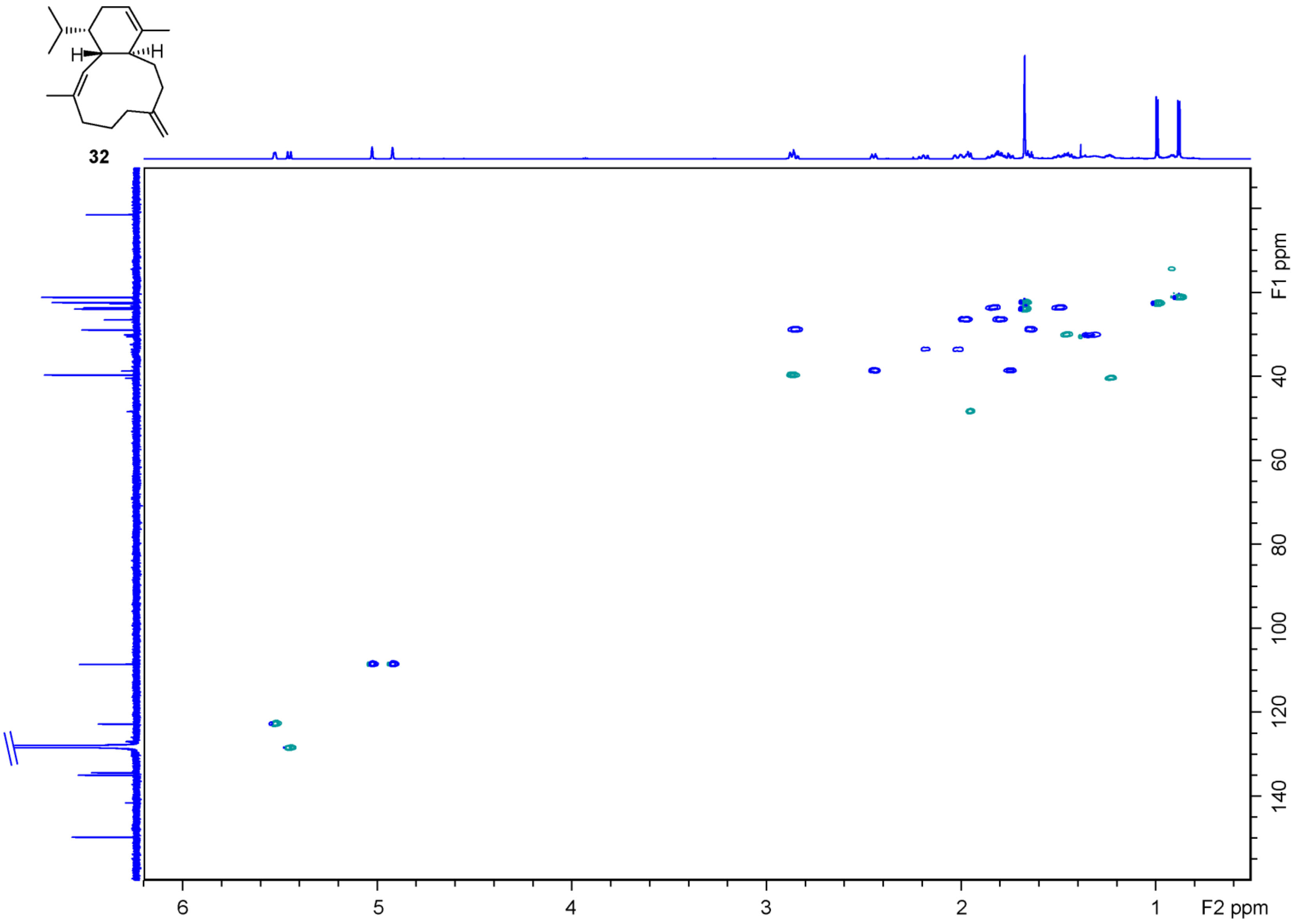

Figure S213. HSQC spectrum (C_6D_6) of **32**.

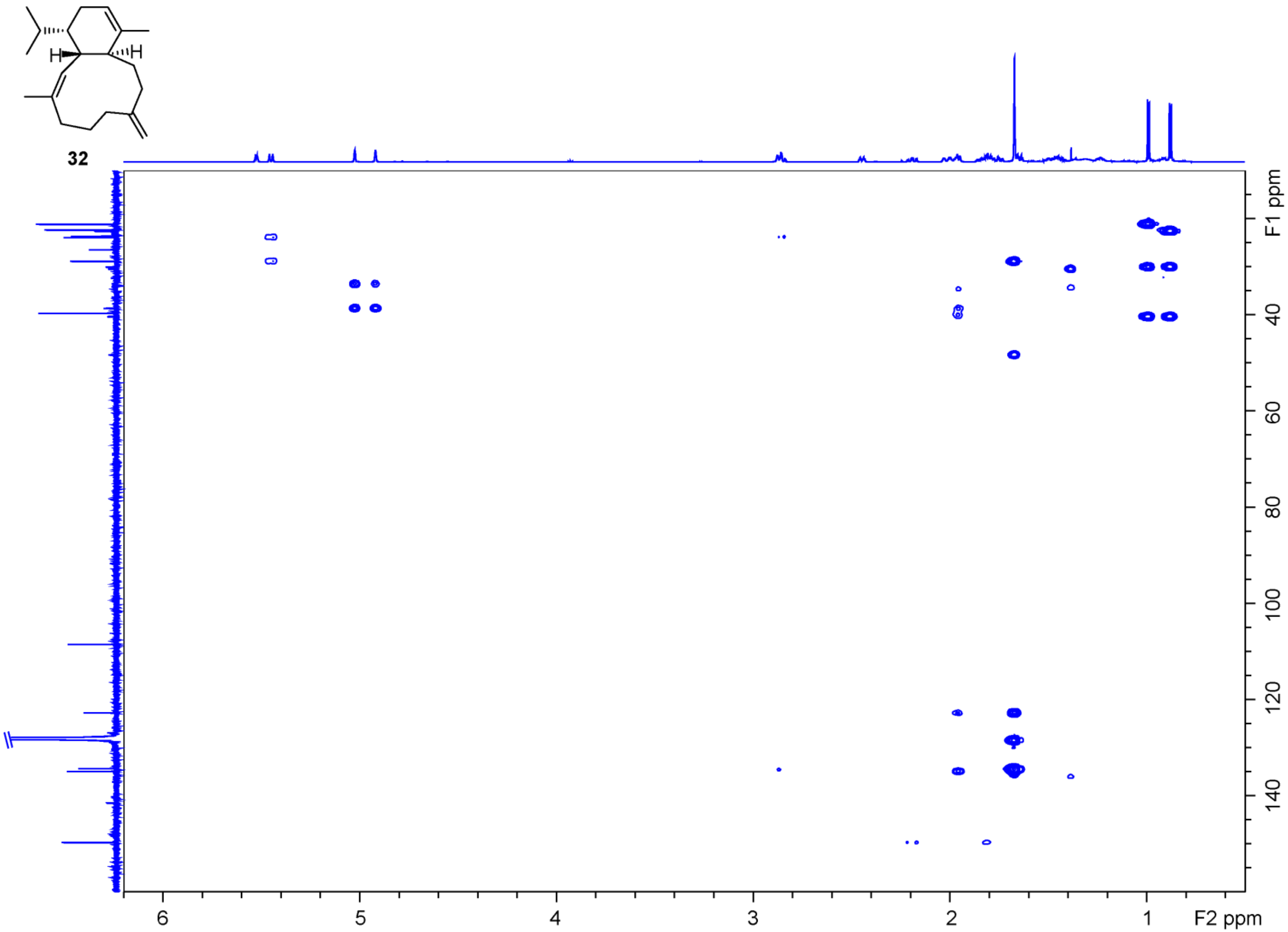

Figure S214. HMBC spectrum (C_6D_6) of **32**.

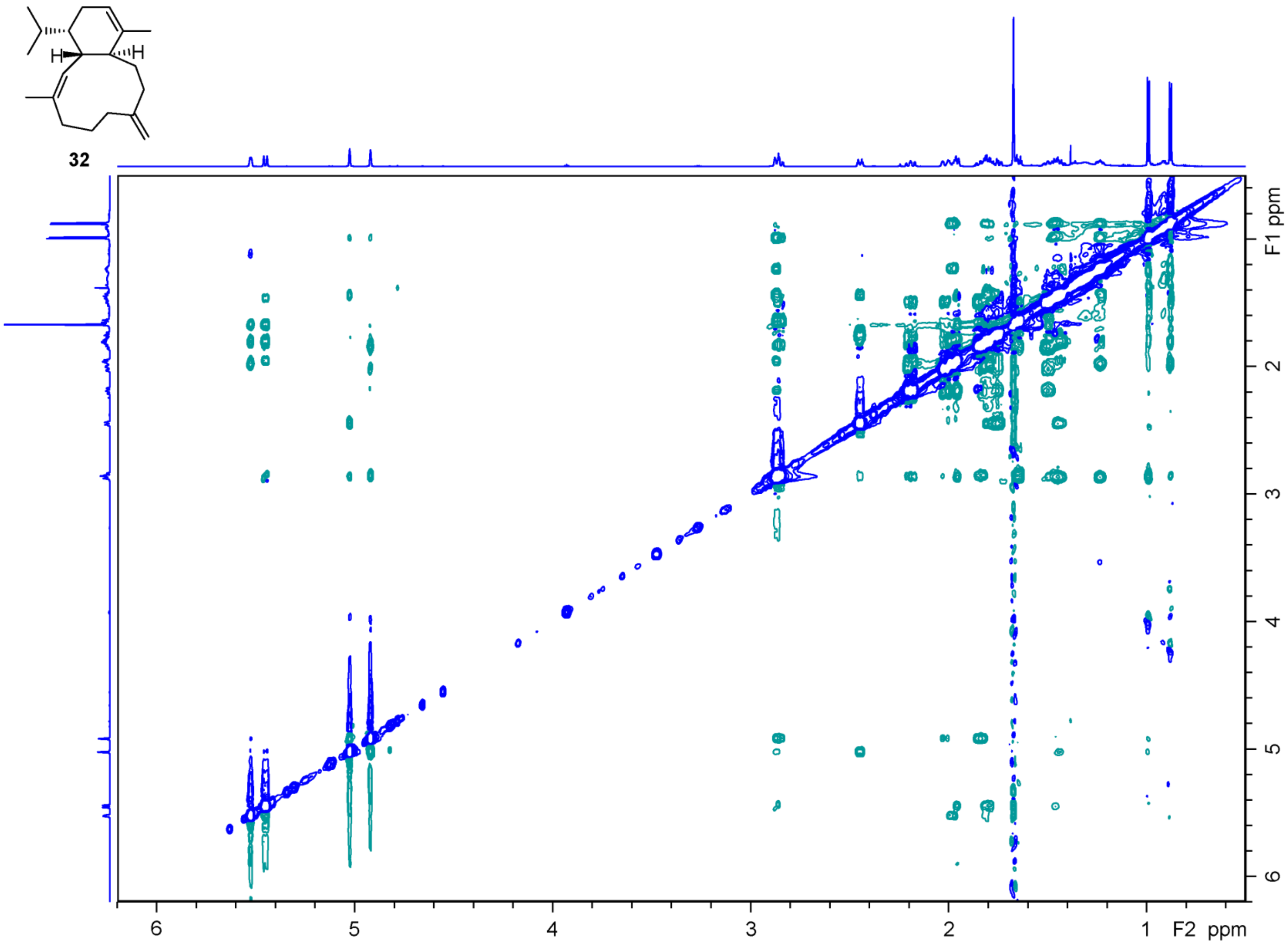

Figure S215. NOESY spectrum (C_6D_6) of **32**.

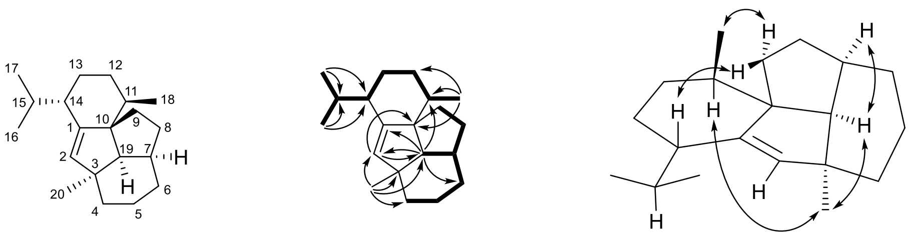

Figure S216. Structure elucidation of wanjuxenene (**33**). Bold: $^{1}H,^{1}H$-COSY, single-headed arrows: key HMBC, and double-headed arrows: key NOESY correlations.

Table S26. NMR data of wanjuxenene (**33**) in C_6D_6 recorded at 298 K.

C[a]	type	^{13}C[b]	^{1}H[b]
1	C_q	149.55	–
2	CH	130.25	4.89 (br s)
3	C_q	45.65	–
4	CH_2	38.15	1.72 (m)
			1.24 (m)
5	CH_2	18.85	1.41 (m)
			1.38 (m)
6	CH_2	31.13	1.42 (m)
			1.35 (m)
7	CH	39.25	1.97 (m)
8	CH_2	27.78	1.59 (m)
			1.41 (m)
9	CH_2	26.79	1.54 (m)
			1.36 (m)
10	C_q	67.08	–
11	CH	41.86	1.68 (m)
12	CH_2	33.06	1.51 (m)
			1.16 (m)
13	CH_2	32.15	1.82 (m)
			0.89 (m)
14	CH	43.62	1.67 (m)
15	CH	29.41	1.78 (m)
16	CH_3	22.50	1.01 (d, ^{3}J = 6.5)
17	CH_3	20.01	0.92 (d, ^{3}J = 6.7)
18	CH_3	17.21	0.90 (d, ^{3}J = 6.8)
19	CH	51.66	1.80 (d, ^{3}J = 10.2)
20	CH_3	34.34	1.06 (s)

[a] Carbon numbering as shown in Figure S216. [b] Chemical shifts δ in ppm, multiplicity: s = singlet, d = doublet, m = multiplet, br = broad, coupling constants J are given in Hertz.

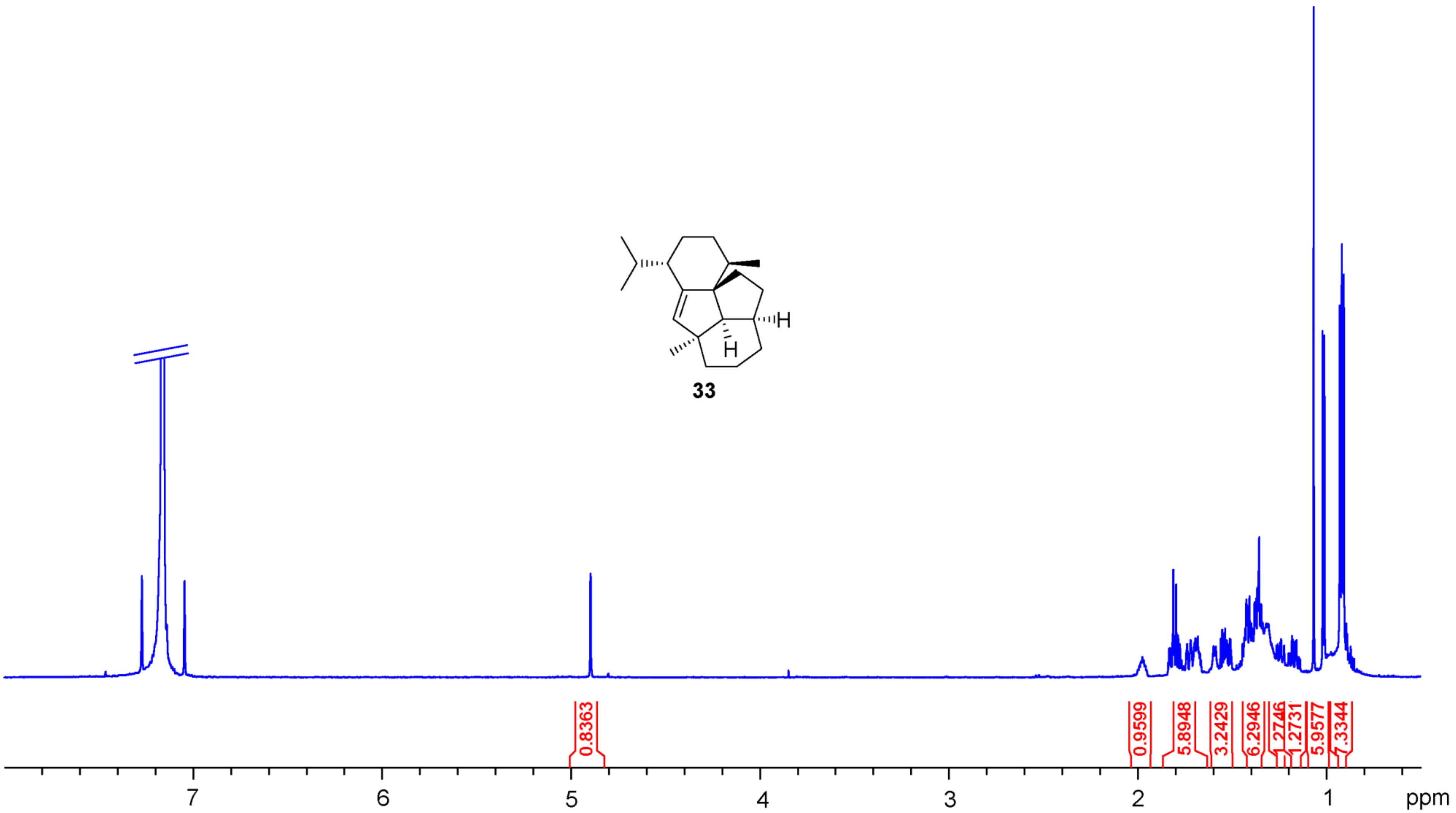

Figure S217. ^{1}H-NMR spectrum (700 MHz, C_6D_6) of **33**.

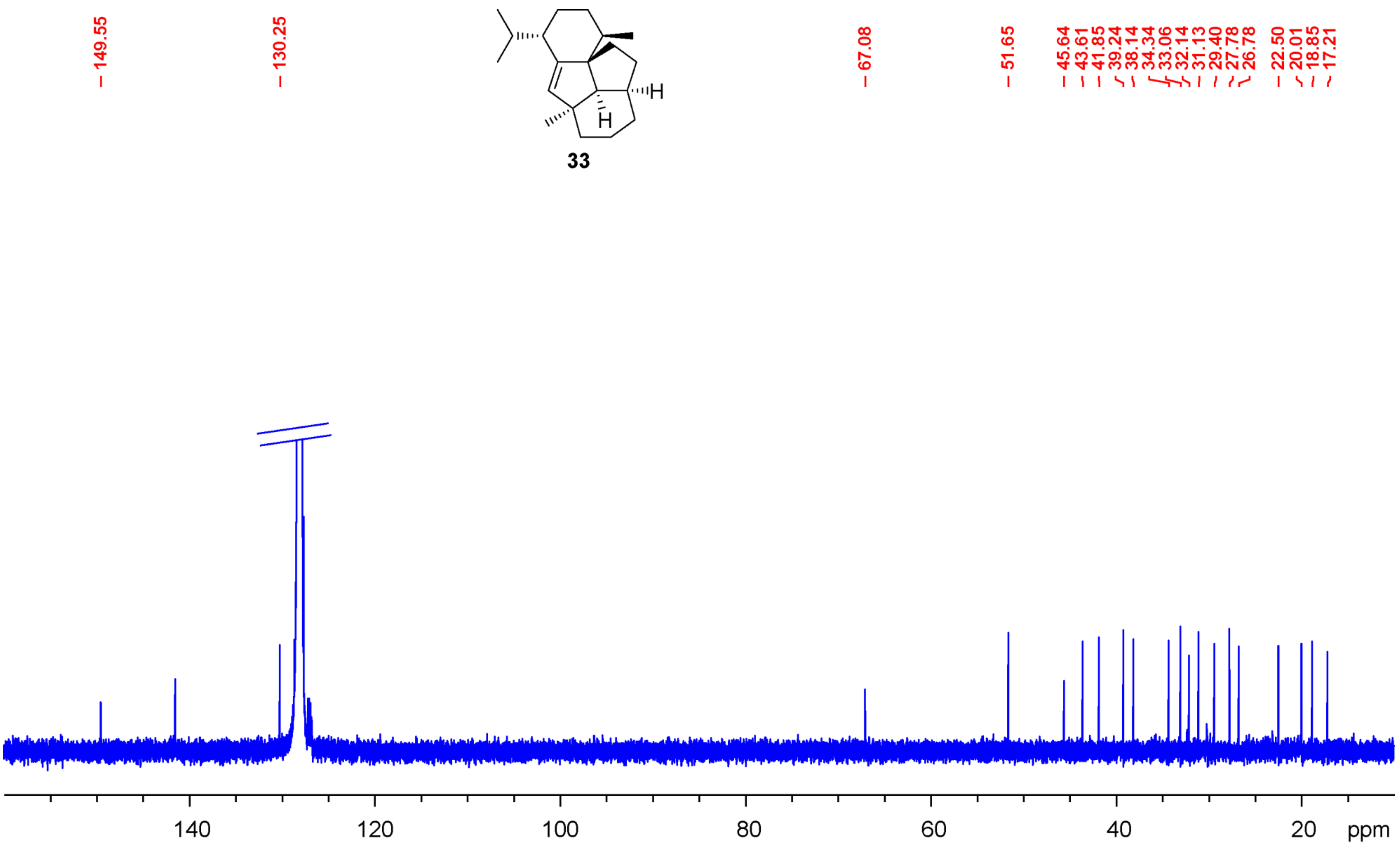

Figure S218. ^{13}C-NMR spectrum (176 MHz, C_6D_6) of **33**.

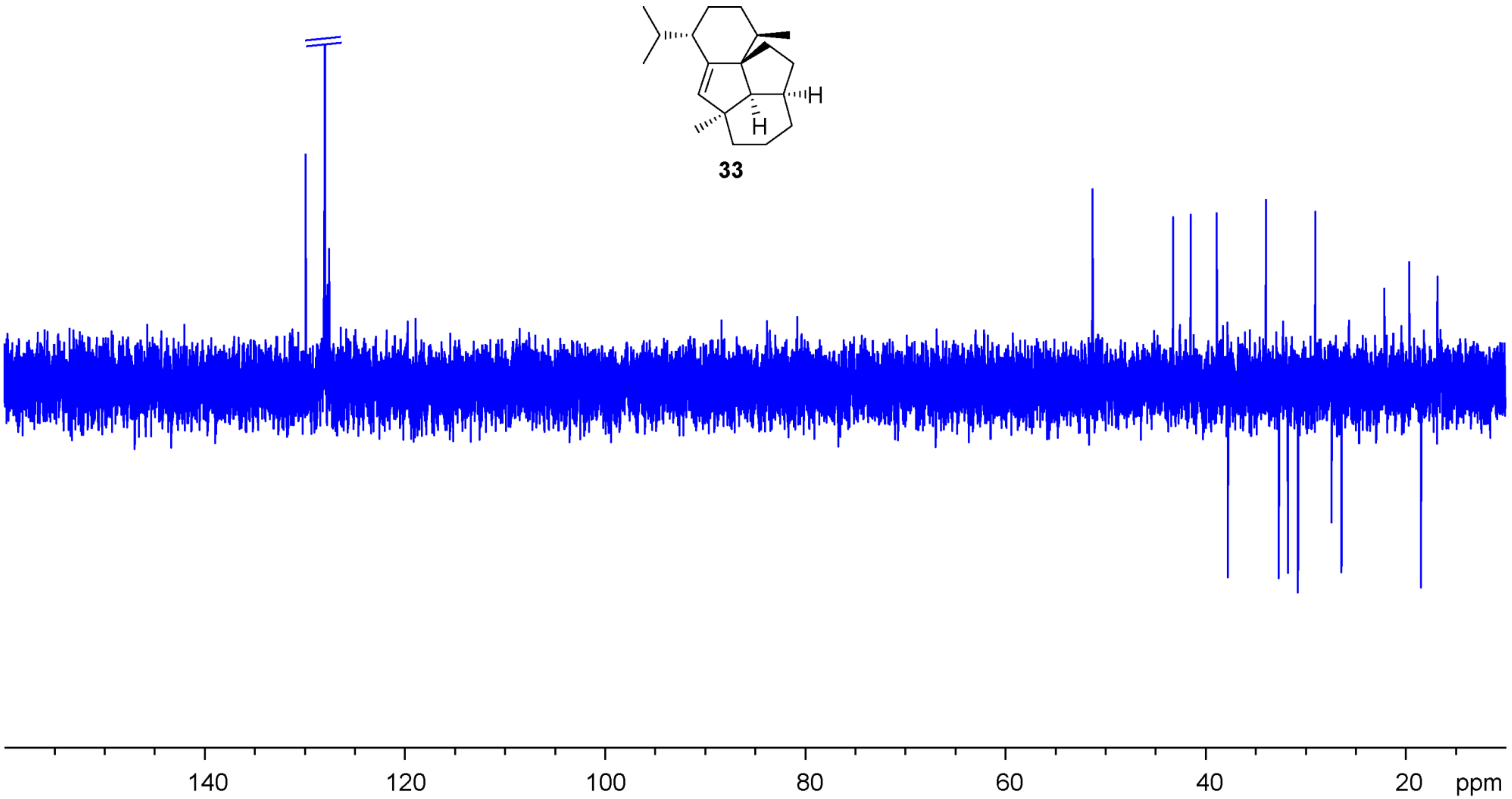

Figure S219. ^{13}C-DEPT135 spectrum (176 MHz, C_6D_6) of **33**.

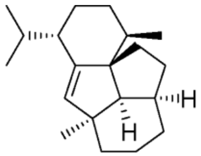

33

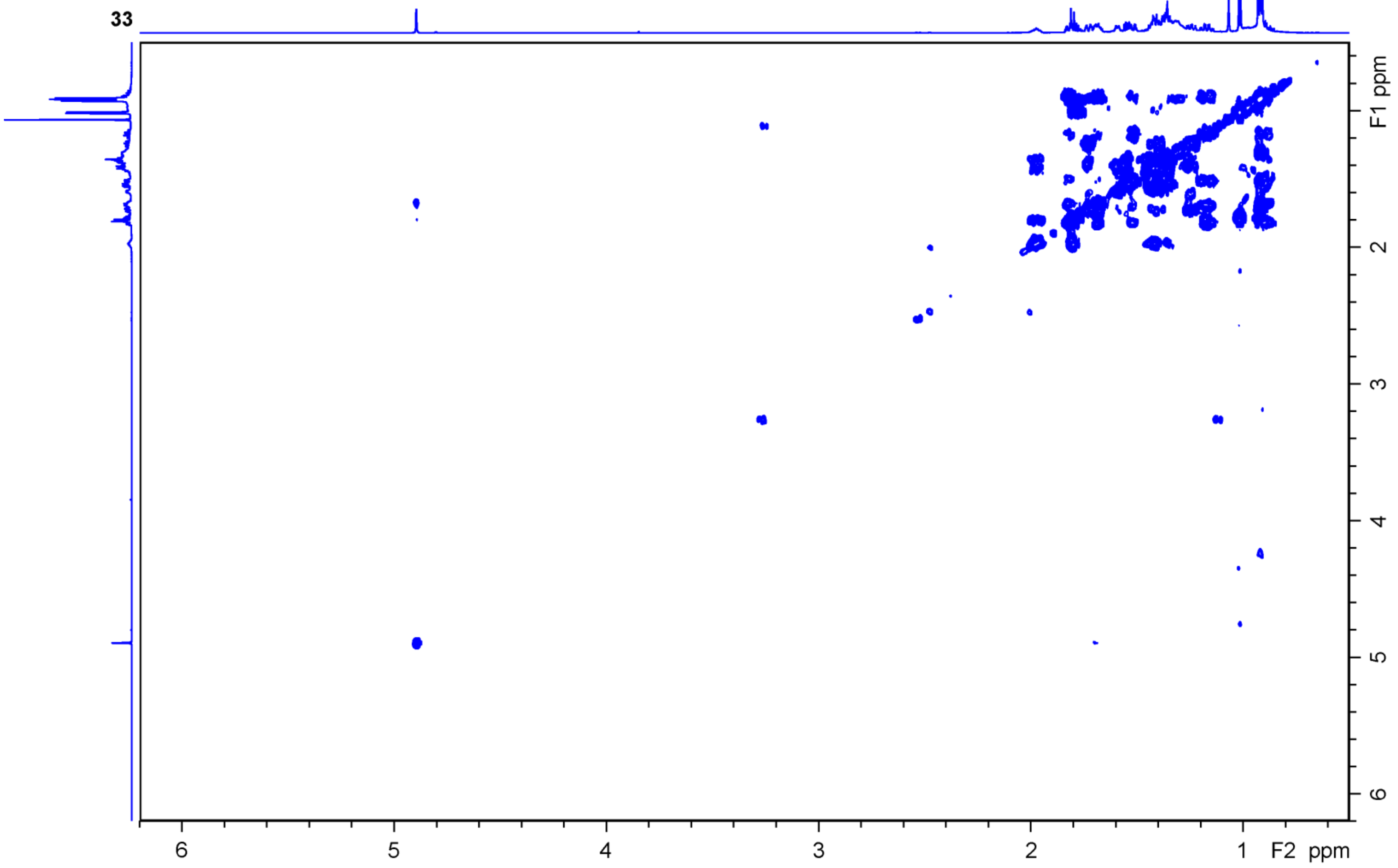

Figure S220. $^{1}H,^{1}H$-COSY spectrum (C_6D_6) of **33**.

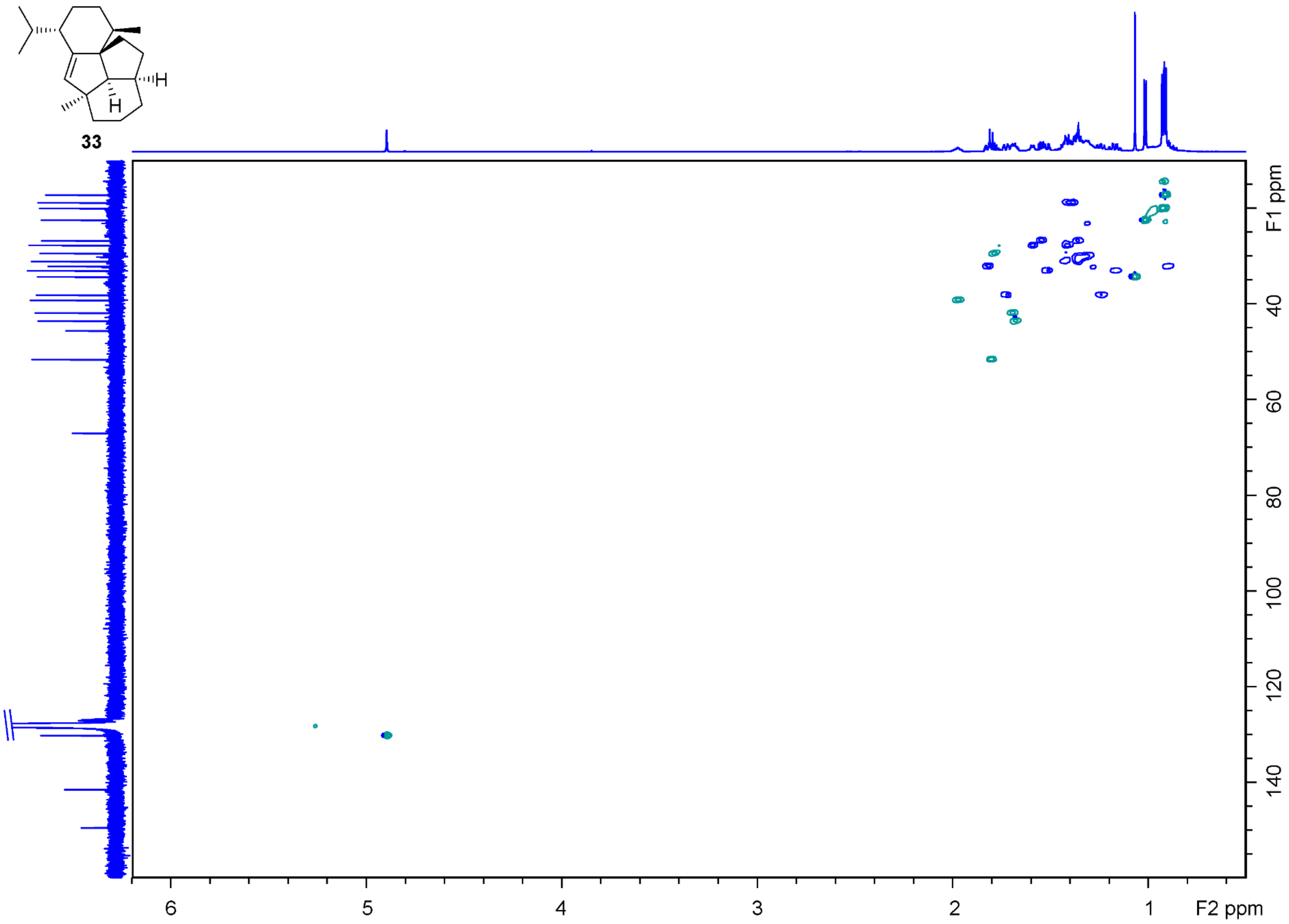

Figure S221. HSQC spectrum (C_6D_6) of **33**.

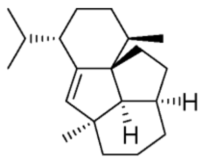

33

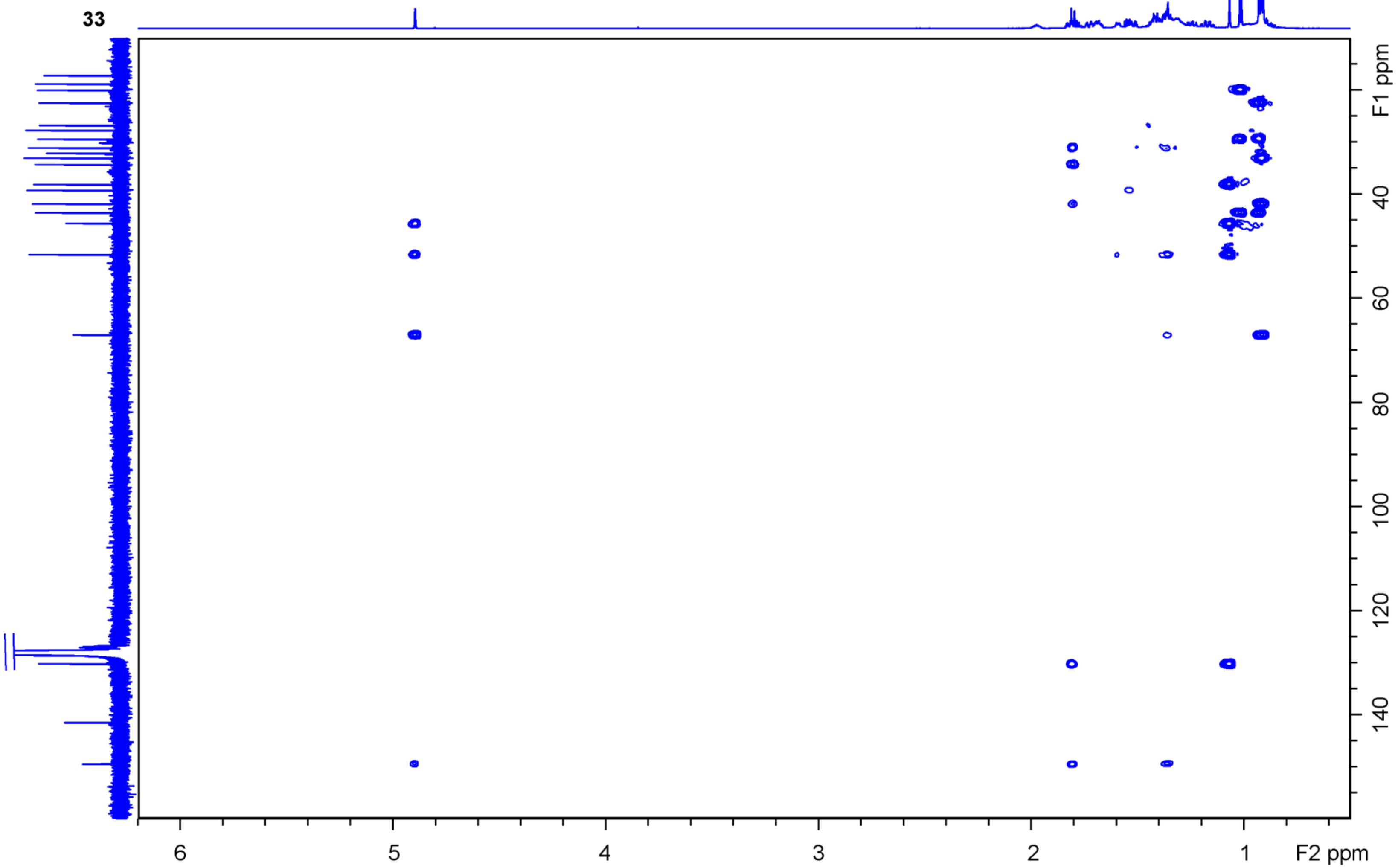

Figure S222. HMBC spectrum (C_6D_6) of **33**.

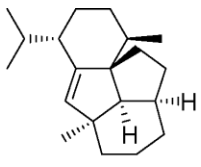

33

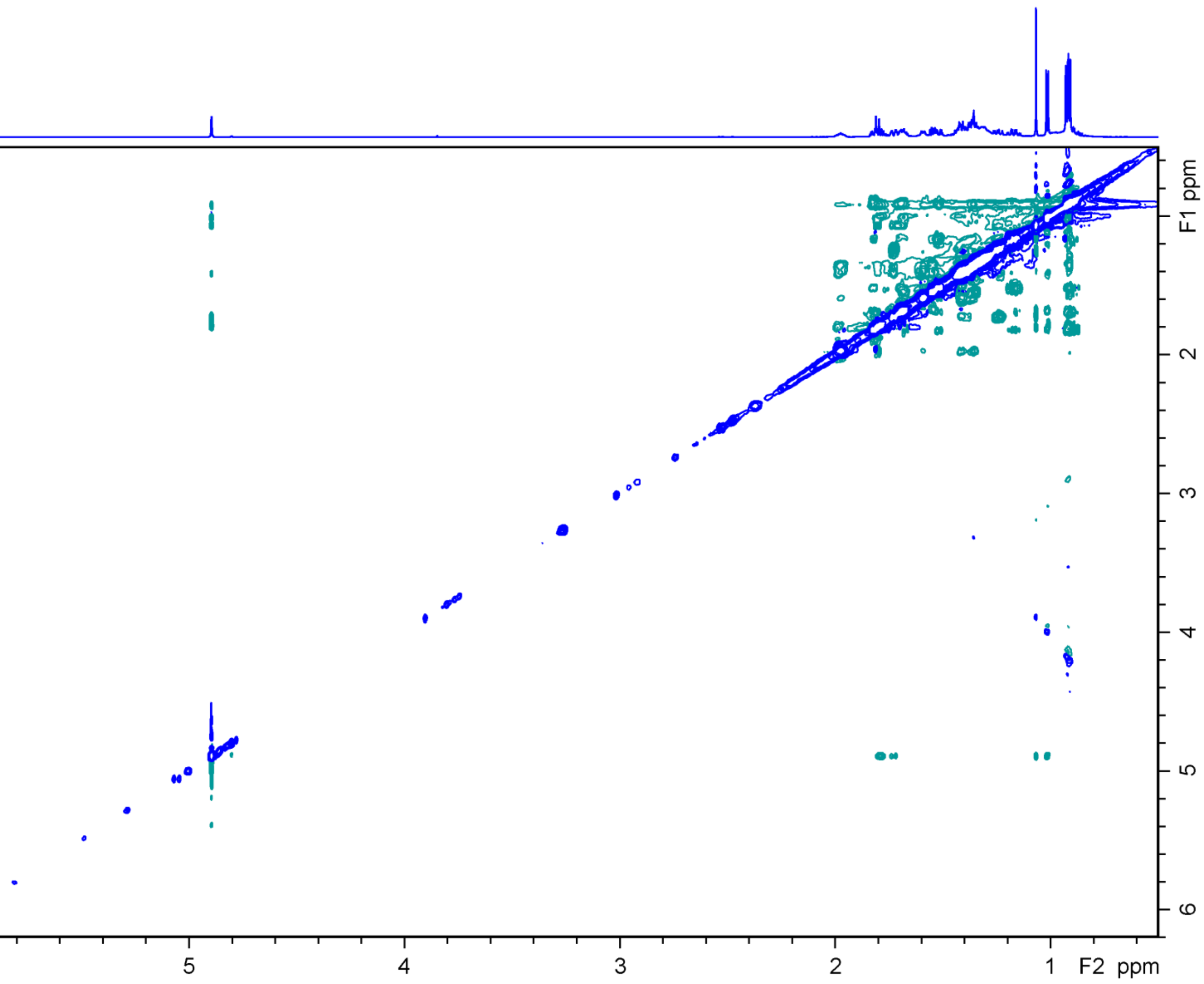

Figure S223. NOESY spectrum (C_6D_6) of **33**.

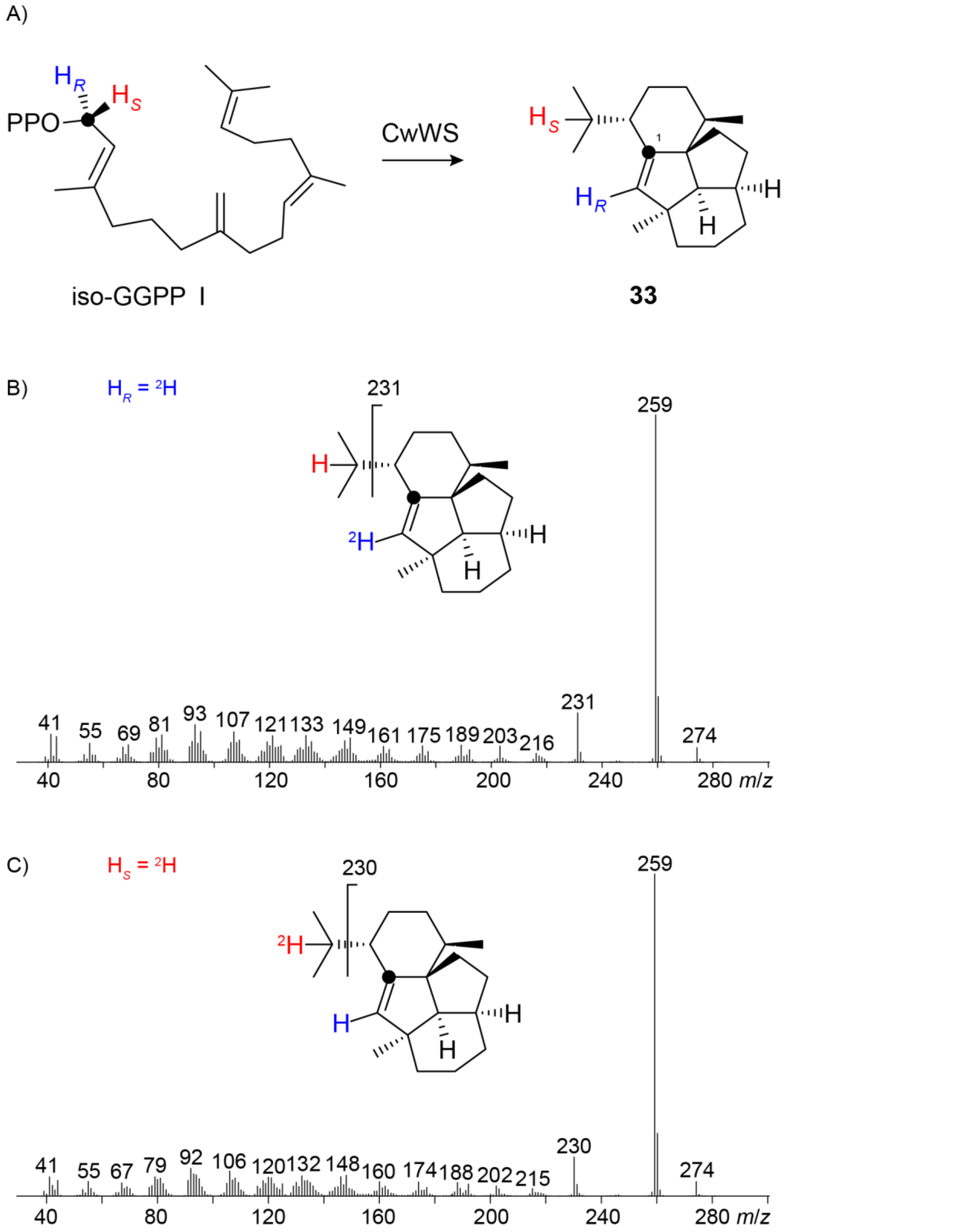

Figure S224. The 1,2-hydride shift from **T** to **U** in the biosynthesis of **33**. Conversion of (*R*)- and (*S*)-(1-^{13}C,1-^{2}H)-iso-GGPP with CwWS and GC/MS analysis of the products. EI mass spectra of the product A) from (*R*)-(1-^{13}C,1-^{2}H)-iso-GGPP showing cleavage of an unlabelled iPr group ($[M–43]^+$) and B) from (*S*)-(1-^{13}C,1-^{2}H)-iso-GGPP showing cleavage of a labelled iPr group ($[M–44]^+$).

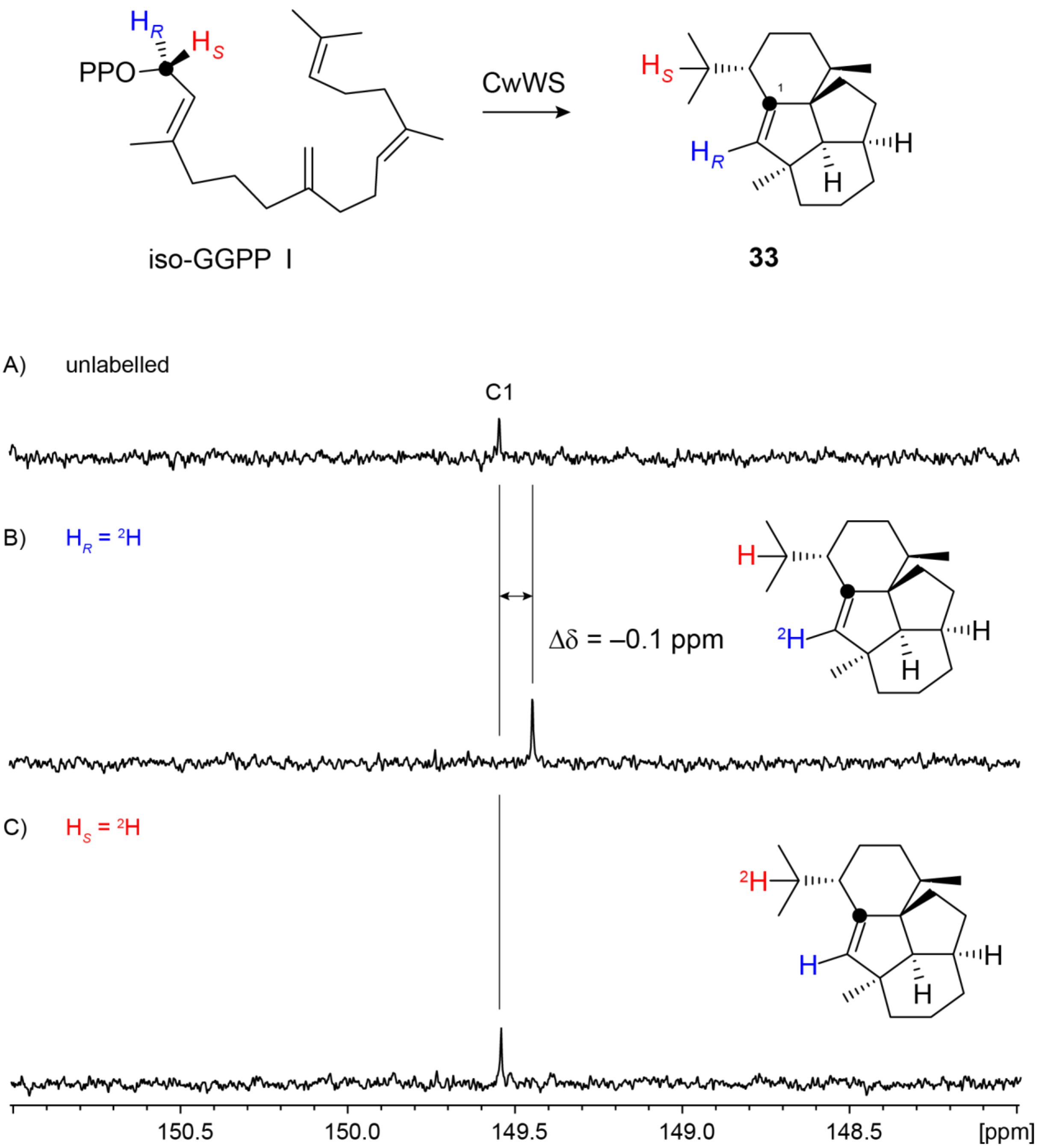

Figure S225. The 1,2-hydride shift from **Z** to **Aa** in the biosynthesis of **34**. ^{13}C-NMR spectra showing the region of C1 for A) unlabelled **33**, B) labelled **33** obtained from (*R*)-(1-^{13}C,1-^{2}H)-iso-GGPP I, and C) labelled **33** obtained from (*S*)-(1-^{13}C,1-^{2}H)-iso-GGPP I. The upfield shift in B) indicates a deuterium atom in a neighbouring position.

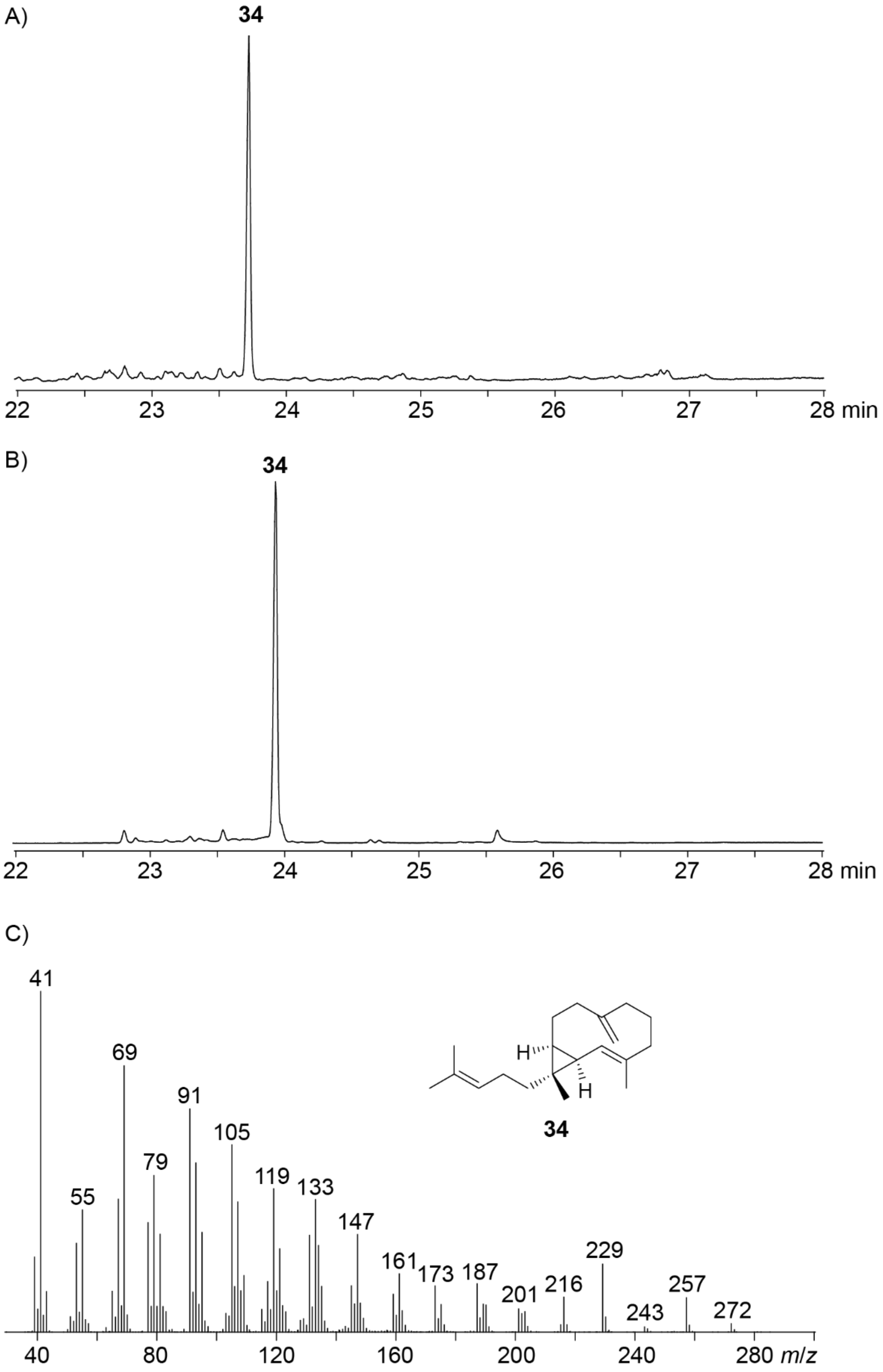

Figure S226. Product mixture formed from iso-GGPP I with SpS and SoS. Total ion chromatograms of the crude extracts from the enzyme incubations with A) SpS and B) SoS. C) EI mass spectrum of **34**. Retention times of **34** in A) and B) are different, because GC columns (HP5-MS) of different ages were used.

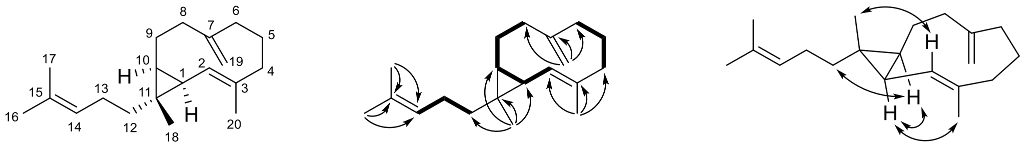

34

Figure S227. Structure elucidation of isocneorubin Y (**34**). Bold: ^{1}H,^{1}H-COSY, single-headed arrows: key HMBC, and double-headed arrows: key NOESY correlations.

Table S27. NMR data of isocneorubin Y (**34**) in C_6D_6 recorded at 298 K.

C[a]	type	^{13}C[b]	^{1}H[b]
1	CH	25.97	1.35 (m)
2	CH	122.62	4.88 (br d, ^{3}J = 10.6)
3	C_q	133.24	–
4	CH_2	41.95	2.11 (m)
			1.83 (ddd, ^{2}J = 12.3, ^{3}J = 12.3, 4.7)
5	CH_2	22.86	1.66 (m)
			1.52 (m)
6	CH_2	31.02	1.98 (m)
			1.54 (m)
7	C_q	147.86	–
8	CH_2	39.18	2.11 (m)
			2.05 (m)
9	CH_2	22.21	1.54 (m)
			1.32 (m)
10	CH	29.00	0.55 (ddd, ^{3}J = 12.1, 9.1, 2.9)
11	C_q	22.91	–
12	CH_2	43.69	1.31 (m)
			1.26 (m)
13	CH_2	25.59	2.16 (m, 2H)
14	CH	125.46	5.22 (thept, ^{3}J = 7.3, ^{4}J = 1.4)
15	C_q	130.74	–
16	CH_3	25.91	1.68 (br s)
17	CH_3	17.68	1.57 (br s)
18	CH_3	13.06	1.09 (s)
19	CH_2	108.17	4.82 (br s, H_Z)
			4.66 (br s, H_E)
20	CH_3	17.10	1.60 (d, ^{4}J = 1.2)

[a] Carbon numbering as shown in Figure S227. [b] Chemical shifts δ in ppm, multiplicity: s = singlet, d = doublet, m = multiplet, coupling constants J are given in Hertz.

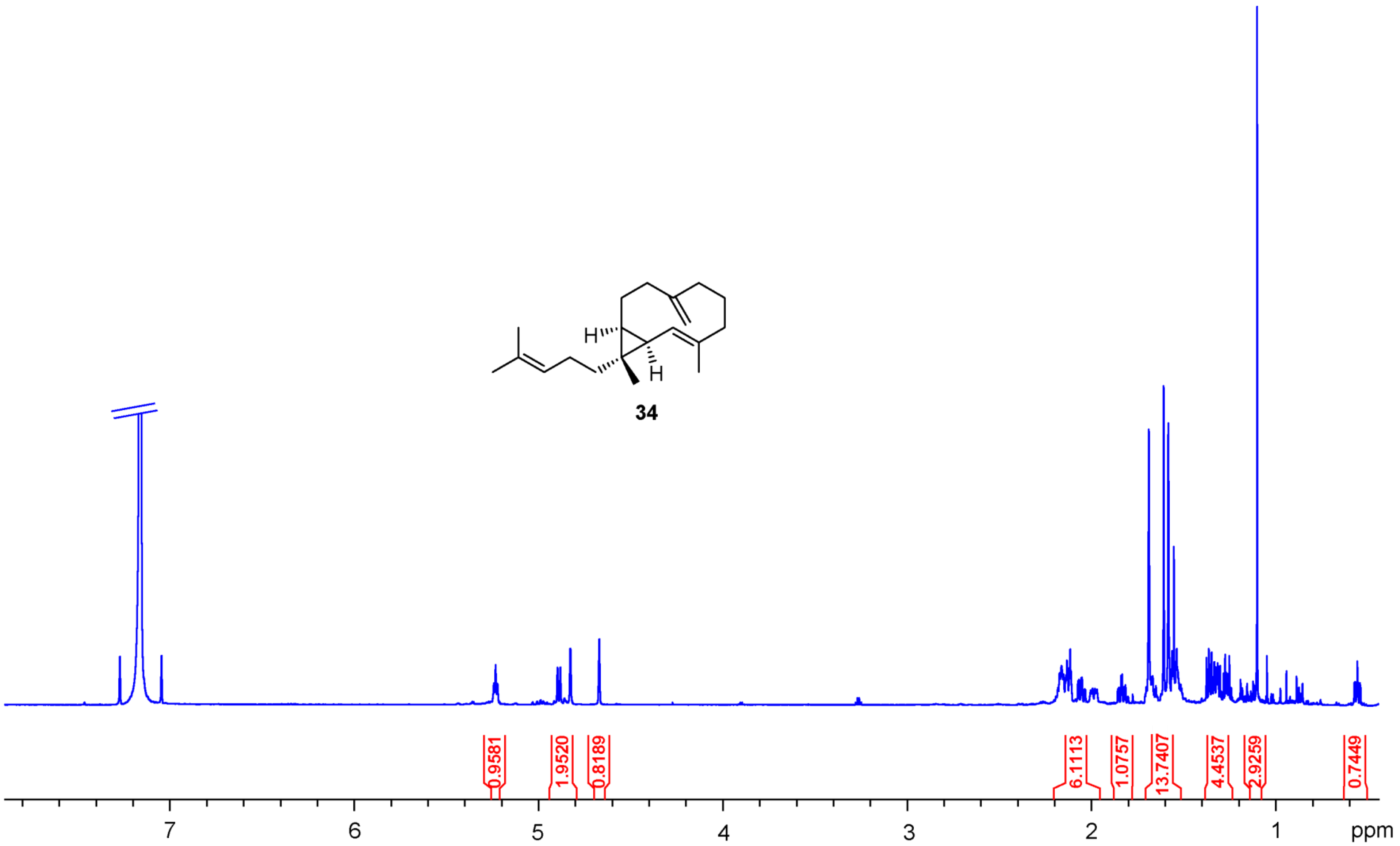

Figure S228. ^{1}H-NMR spectrum (700 MHz, C_6D_6) of **34**.

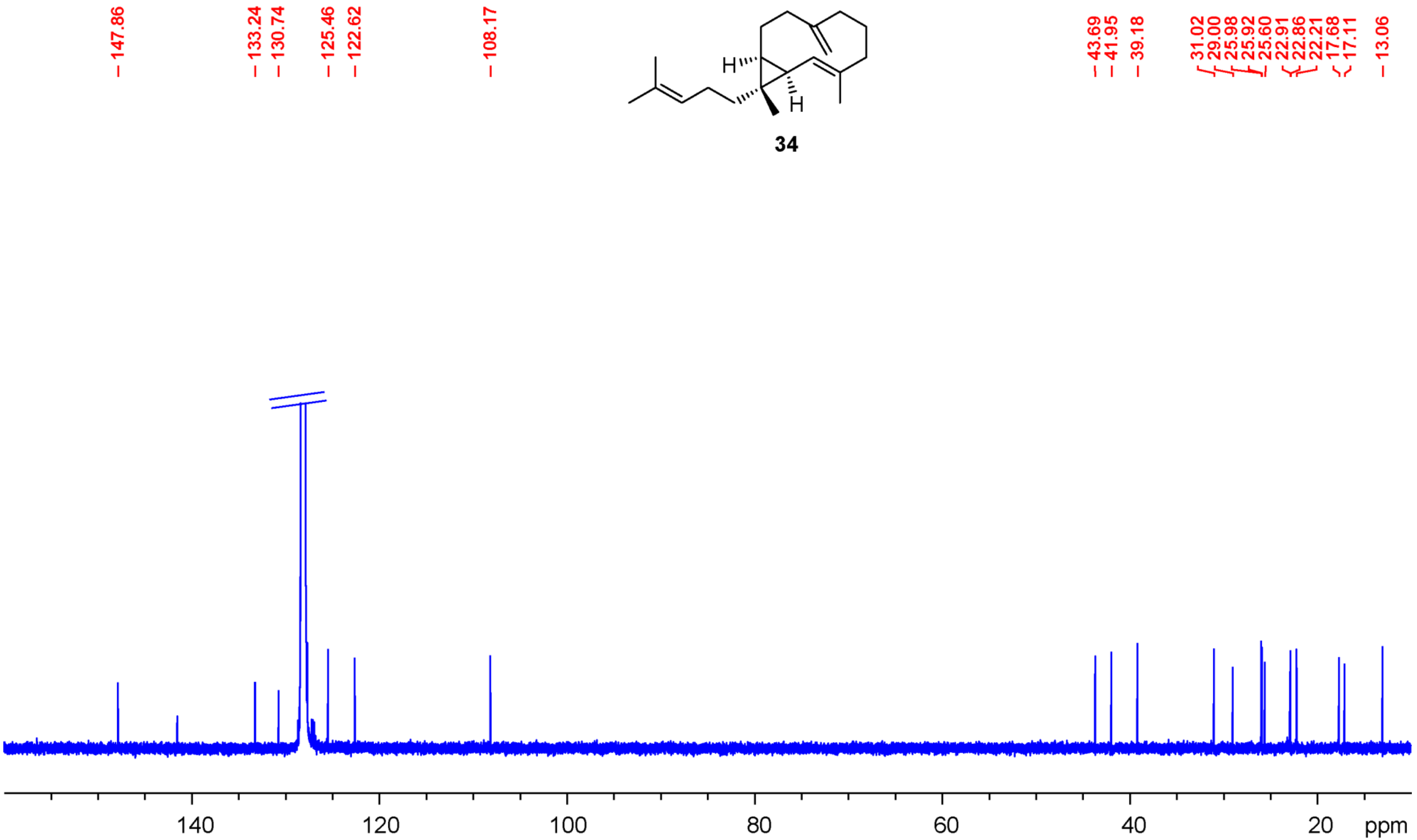

Figure S229. ^{13}C-NMR spectrum (176 MHz, C_6D_6) of **34**.

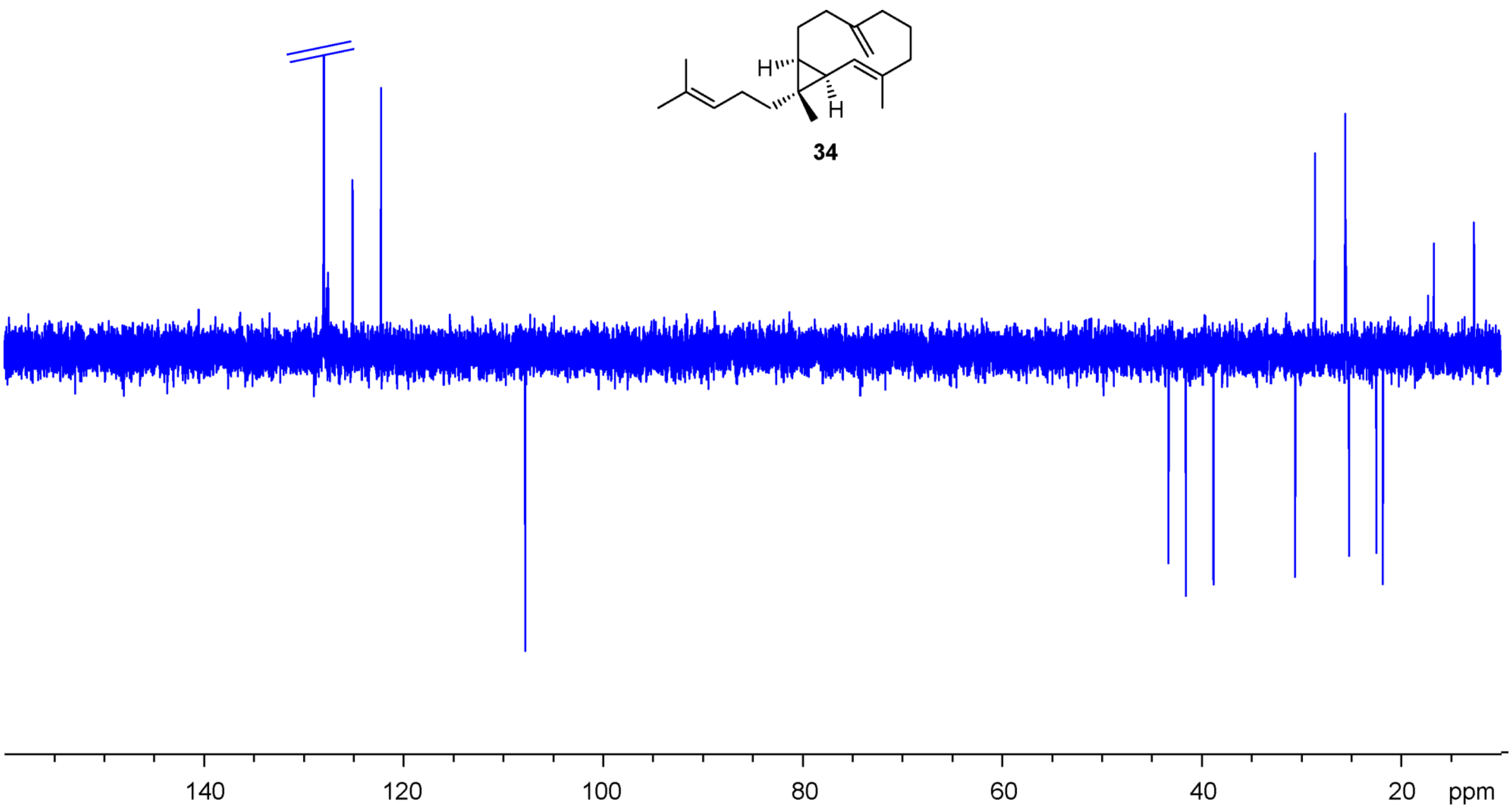

Figure S230. ^{13}C-DEPT135 spectrum (176 MHz, C_6D_6) of **34**.

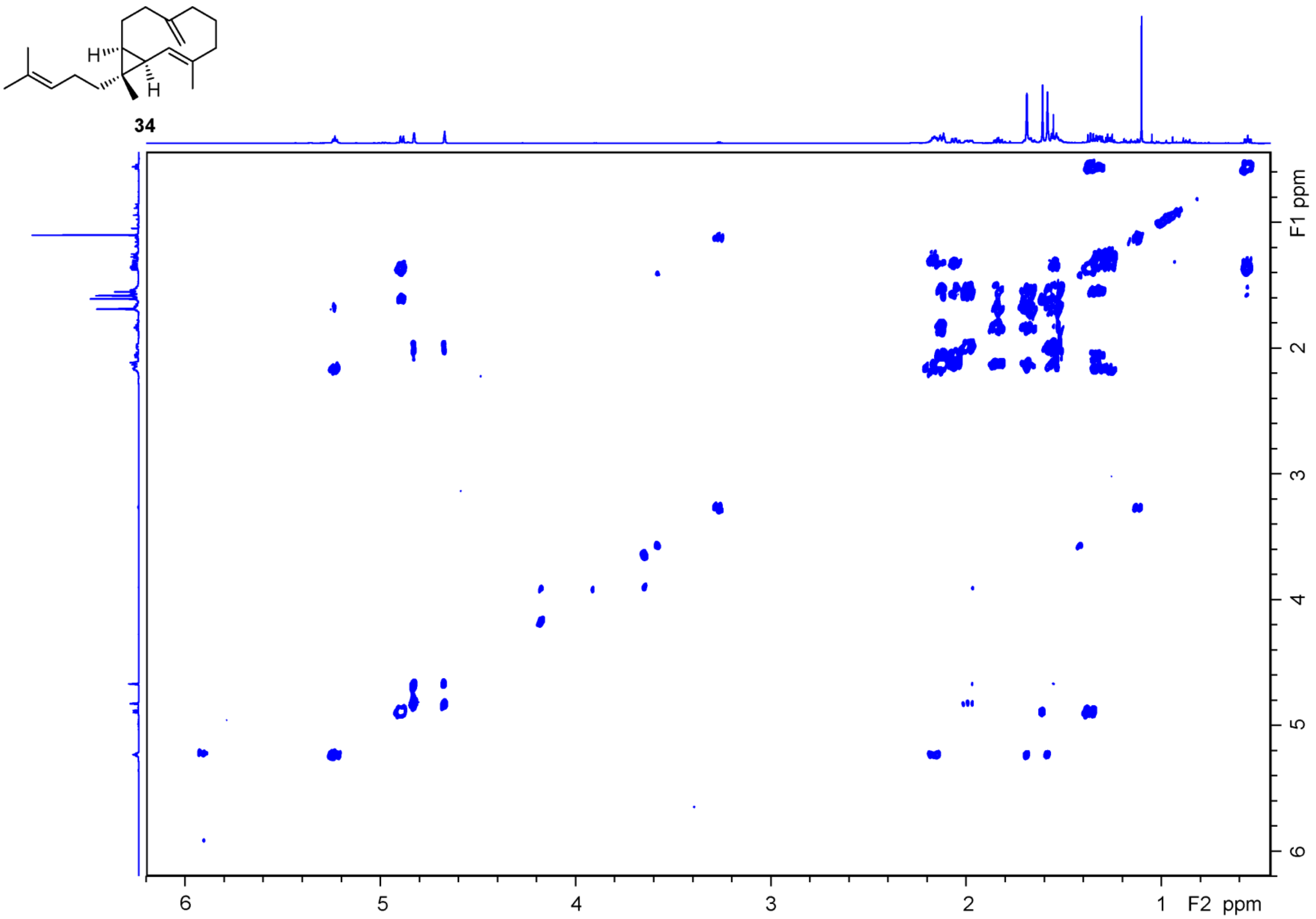

Figure S231. $^{1}H,^{1}H$-COSY spectrum (C_6D_6) of **34**.

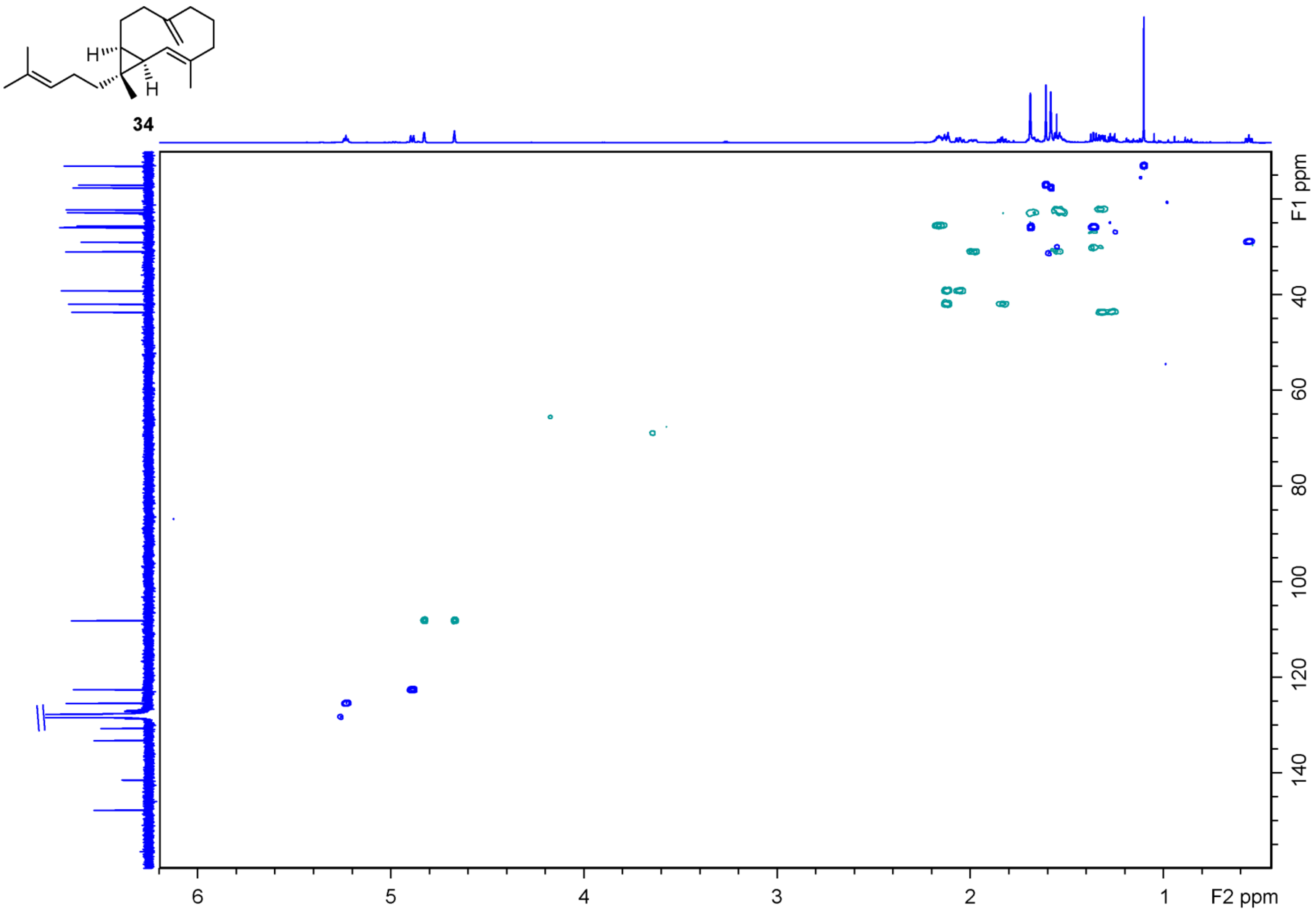

Figure S232. HSQC spectrum (C_6D_6) of **34**.

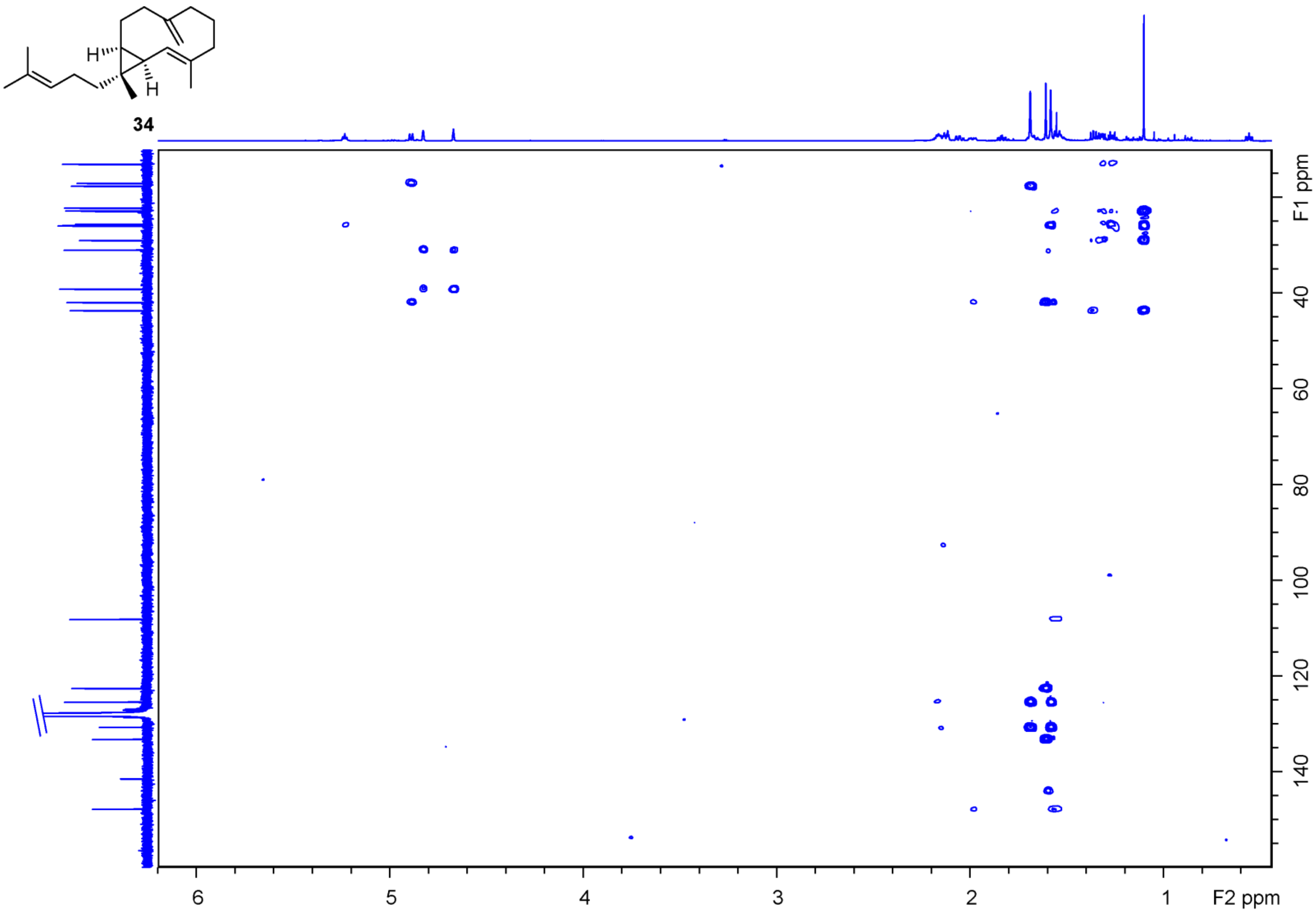

Figure S233. HMBC spectrum (C_6D_6) of **34**.

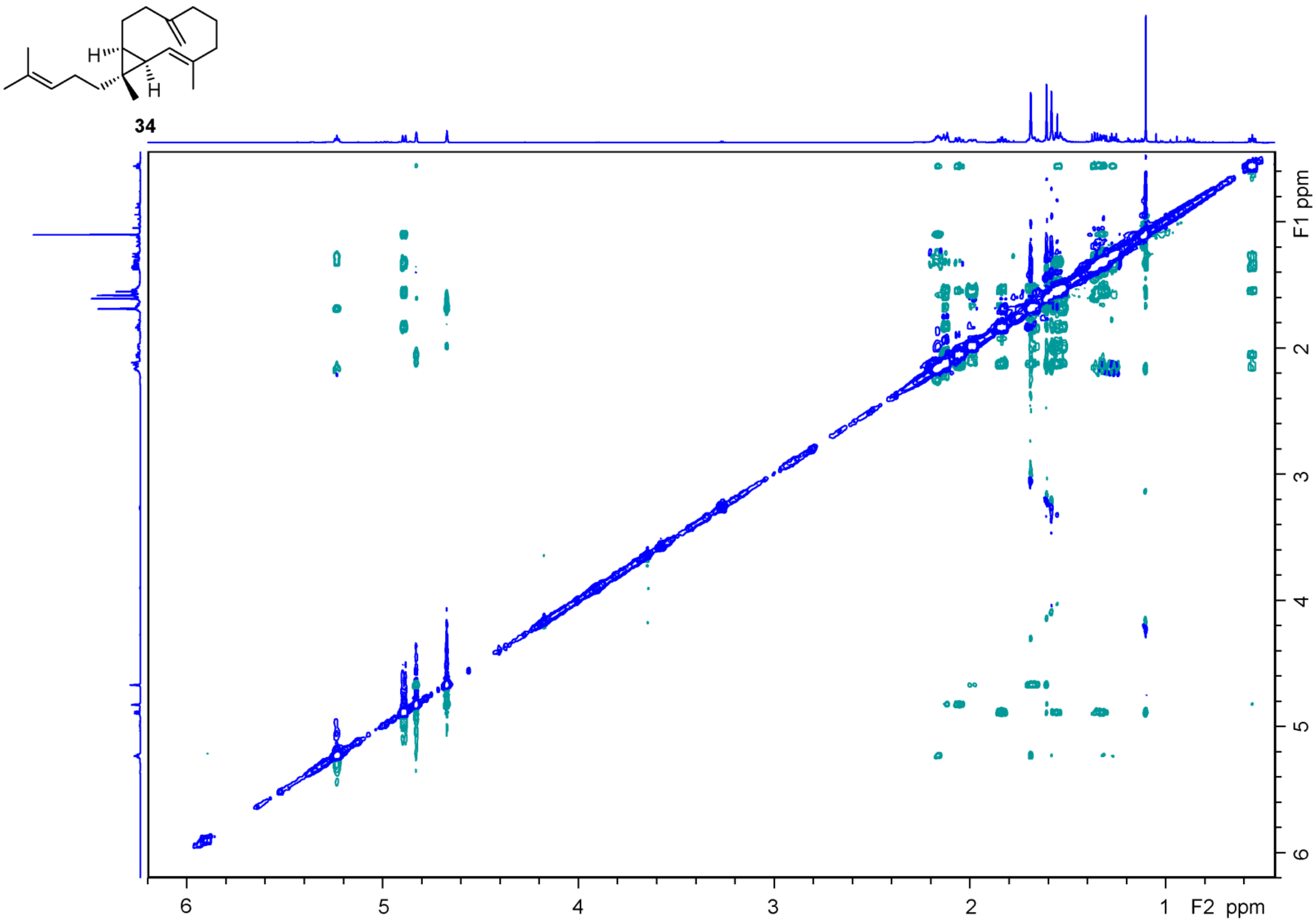

Figure S234. NOESY spectrum (C_6D_6) of **34**.

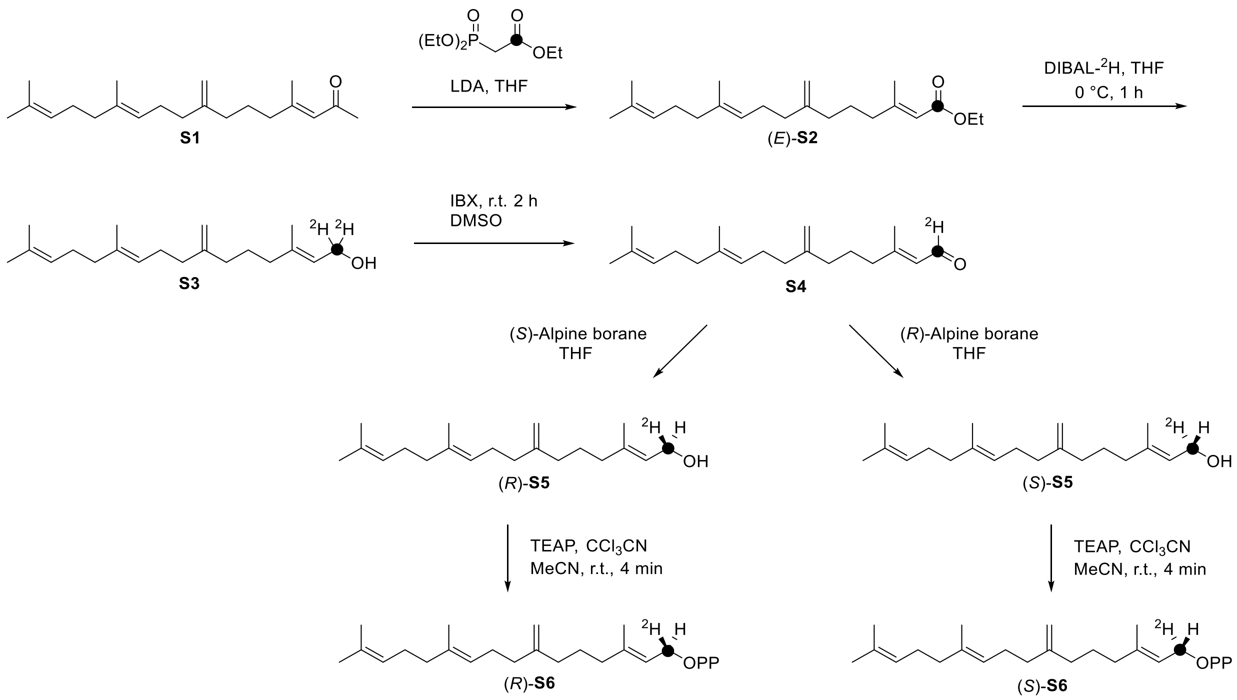

Scheme S13. Synthesis of (*R*)- and (*S*)-(1-^{13}C,1-^{2}H)-iso-GGPP (**S6**).

Synthetic procedures

Synthesis of ethyl (1-^{13}C,1-^{2}H)-(2*E*,10*E*)-3,11,15-trimethyl-7-methylenehexadeca-2,10,14-trienoate (S2)

A solution of diisopropylamine (1.28 g, 12.6 mmol, 1.05 eq) dissolved in dry THF (50 mL) was cooled to 0 °C. *n*-Buli (7.9 mL, 1.6 M in hexane, 1.05 eq) was added dropwise and the reaction was stirred for 1 h at 0 °C. The reaction mixture was cooled to –78 °C and triethyl (1-^{13}C)phosphonoacetate (2.70 g, 12.0 mmol, 1.00 eq) was added. After stirring the reaction mixture for 1 h at –78 °C the methyl ketone **S1**[18] (3.15 g, 12.0 mmol, 1.00 eq) was added. The reaction mixture was allowed to warm to room temperature and stirred overnight. Water (50 mL) was added to quench the reaction. The aqueous phase was extracted with Et_2O (three times), and the combined layers were dried with $MgSO_4$ and concentrated under reduced pressure. Purification by column chromatography (cyclohexane/EtOAc, 13:1) yielded pure (*E*)-**S2** (1.38 g, 4.13 mmol, 34 %) as a colorless oil.

(*E*)-S2. TLC (pentane, 10:1): R_f = 0.59. GC (HP5-MS): *I* = 2330. ^{1}H-NMR (C_6D_6, 500 MHz): 5.82 (dhex, $^{4}J_{H,H}$ = 1.2, $^{3}J_{H,C}$ = 2.6, 1.3, 1H), 5.25 (m, 1H), 4.84 (br s, 1H), 4.77 (br s, 1H), 4.06 (qd, $^{3}J_{H,H}$ = 7.1, $^{3}J_{H,C}$ = 3.1, 2H), 2.19 (m, 3H), 2.17 (m, 2H), 2.10 (m, 2H), 2.02 (m, 2H), 1.84 (m, 4H), 1.68 (q, $^{4}J_{H,C}$ = 1.3, 3H), 1.60 (br s, 3H), 1.57 (br s, 3H), 1.42 (m, 2H), 1.02 (t, $^{3}J_{H,H}$ = 7.1, 3H) ppm. ^{13}C-NMR (C_6D_6, 125 MHz): 166.47 ($^{13}C_q$), 159.55 (d, $^{2}J_{C,C}$ = 2.1, C_q), 148.91 (C_q), 135.31 (C_q), 131.22 (C_q), 124.91 (CH), 124.60 (CH), 116.36 (d, $^{1}J_{C,C}$ = 75.8, CH), 109.82 (CH_2), 59.40 (d, $^{2}J_{C,C}$ = 2.2, CH_2), 40.54 (d, $^{3}J_{C,C}$ = 7.0, CH_2), 40.21 (CH_2), 36.33 (CH_2), 35.82 (CH_2), 27.20 (CH_2), 26.76 (CH_3), 25.88 (CH_2), 25.69 (CH_2), 18.73 (d, $^{3}J_{C,C}$ = 1.5, CH_3), 17.77 (CH_3), 16,14 (CH_3), 14.46 (d, $^{3}J_{C,C}$ = 2.2, CH_3) ppm. IR (diamond ATR): ṽ = 2976 (m), 2928 (s), 2856 (m), 1674 (s), 1640 (s), 1443 (m), 1381 (m), 1210 (s), 1127 (s), 1070 (m), 1039 (m), 983 (w), 888 (m), 725 (w) cm^{-1}. HRMS (EI): $[M]^+$ calcd. for $^{13}CC_{21}H_{36}O_2^+$ *m*/*z* 333.2794; found *m*/*z* 333.2773.

Synthesis of (1-^{13}C,1-^{2}H)-(2*E*,10*E*)-3,11,15-trimethyl-7-methylenehexadeca-2,10,14-trien-1-ol (S3)

To a cooled (0 °C) solution of (*E*)-**S2** (1.36 g, 4 mmol, 1.00 eq) in THF (25 mL) was added DIBAL-^{2}H (0.7 M in toluene, 2.40 eq) and the reaction mixture was stirred for 1 h at room temperature. The mixture was cooled to 0 °C again and a saturated solution of Na-K-tartrate was added. The resulting slurry was stirred for 2 h to dissolve the precipitate and the aqueous phase was extracted with Et_2O (three times). The organic layers were dried with $MgSO_4$ and concentrated under reduced pressure. The residue was purified by column chromatography (pentane/Et_2O, 3:1) to yield the alcohol **S3** (897 mg, 3.06 mmol, 77%) as a colorless oil.

S3. TLC (petroleum/Et_2O, 1:1): R_f = 0.48. GC (HP5-MS): *I* = 2204. ^{1}H-NMR (C_6D_6, 500 MHz): 5.37 (br s, 1H), 5.29 (thex, $^{3}J_{H,H}$ = 6.9, $^{4}J_{H,H}$ = 1.2, 1H), 5.24 (thept, $^{3}J_{H,H}$ = 6.9, $^{4}J_{H,H}$ = 1.4, 1H), 4.89 (m, 1H), 4.86 (m, 1H), 2.19 (m, 4H), 2.10 (m, 4H), 1.99 (t, $^{3}J_{H,H}$ = 7.7, 2H), 1.91 (t, $^{3}J_{H,H}$ = 7.7, 2H), 1.68 (d, $^{4}J_{H,H}$ = 1.0, 3H), 1.60 (br s, 3H), 1.56 (br s, 3H), 1.53 (m, 2H), 1.45 (d, $^{4}J_{H,H}$ = 1.4, 3H), 0.63 (br s, OH) ppm. ^{13}C-NMR (C_6D_6, 125 MHz): 149.44 (C_q), 138.31 (C_q), 135.27 (C_q), 131.20 (C_q), 124.93 (CH), 124.85 (CH, $^{1}J_{C,C}$ = 46.8), 124.68 (CH), 109.59 (CH_2), 58.66 (pent, $^{1}J_{C,D}$ = 21.5, $^{13}C^{2}H_2$), 40.21 (CH_2), 39.48 (d, $^{3}J_{C,C}$ = 4.8, CH_2), 36.50 (CH_2), 36.09 (CH_2), 27.21 (CH_2), 26.84 (CH_2), 26.21 (CH_2), 25.88 (CH_3), 17.77 (CH_3), 16.15 (CH_3), 16.09 (d, $^{3}J_{C,C}$ = 4.2 Hz, CH_3) ppm. IR (diamond ATR): ṽ = 3339 (w), 2965 (m), 2927 (s), 2859 (m), 1643 (w), 1440 (m), 1378 (m), 1068 (w), 948 (m), 886 (s), 832 (w) cm^{-1}. HRMS (EI): $[M]^+$ calcd. for $^{13}CC_{19}H_{32}D_2O^+$ *m*/*z* 293.2609; found *m*/*z* 293.2646.

Synthesis of (1-^{13}C,1-^{2}H)-(2*E*,10*E*)-3,11,15-trimethyl-7-methylenehexadeca-2,10,14-trienal (S4)

To a solution of IBX (1.19 g, 4.25 mmol, 1.40 eq) in DMSO (25 mL) was slowly added **S2** (889 mg, 3.03 mmol, 1.00 eq; in 4 mL DMSO) and the reaction mixture was stirred for 2 h at room temperature. The reaction was monitored by TLC analysis. Et_2O (30 mL) was added and the organic layer was washed with sat. NH_4Cl (20 mL). The organic layer was dried with $MgSO_4$ and concentrated under reduced pressure. Purification by column chromatography (petroleum/EtOAc, 4:1) yielded the aldehyde **S4** (707 mg, 2.43 mmol, 80%) as a colorless oil.

S4. TLC (petroleum/EtOAc, 10:1): R_f = 0.38. GC (HP5-MS): *I* = 2230. ^{1}H-NMR (C_6D_6, 500 MHz): 5.82 (br s, 1H), 5.27 (thex, $^{3}J_{H,H}$ = 7.0, $^{4}J_{H,H}$ = 1.2, 1H), 5.23 (thept, $^{3}J_{H,H}$ = 7.0, $^{4}J_{H,H}$ = 1.4, 1H), 4.85 (br s, 1H), 4.75 (br s, 1H), 2.18 (m, 4H), 2.10 (m, 2H), 2.01 (m, 2H), 1.80 (m, 2H), 1.70 (br t, $^{3}J_{H,H}$ = 8.0, 2H), 1.68 (q, $^{4}J_{H,H}$ = 1.0, 3H), 1.60 (br s, 3H), 1.57 (br s, 3H), 1.51 (br s, 3H), 1.31 (m, 2H) ppm. ^{13}C-NMR (C_6D_6, 125 MHz): 189.49 (t, $^{1}J_{C,D}$ = 26.0, $^{13}C^{2}HO$), 161.49 (d, $^{2}J_{C,C}$ = 2.7, C_q), 148.73 (C_q), 135.45 (C_q), 131.27 (C_q), 127.48 (t, $^{2}J_{C,D}$ = 3.65, 0.5 CH, other half of the signal is covered by solvent signal), 124.86 (CH), 124.48 (CH), 109.93 (CH_2), 40.20 (CH_2), 39.97 (d, $^{3}J_{C,C}$ = 5.4, CH_2), 36.31 (CH_2), 35.78 (CH_2), 27.19 (CH_2), 26.75 (CH_2), 25.88 (CH_2), 25.30 (CH_3), 17.77 (CH_3), 16.83 (d, $^{3}J_{C,C}$ = 4.8, CH_3), 16.15 (CH_3) ppm. IR (diamond ATR): ṽ = 2923 (m), 2855 (m), 1631 (s), 1439 (m), 1379 (m), 1187 (w), 1135 (w), 994 (w), 888 (m) cm^{-1}. HRMS (EI): $[M]^+$ calcd. for $^{13}CC_{19}H_{31}D_1O_1^+$ *m*/*z* 290.2453; found *m*/*z* 290.2479.

Synthesis of (*R*)- and (*S*)-(1-^{13}C,1-^{2}H)-(2*E*,10*E*)-3,11,15-trimethyl-7-methylenehexadeca-2,10,14-trien-1-ol (S5)

To a cooled solution (0 °C) of (*R*)- and (*S*)-Alpine borane (1.25 eq; 0.5 M in THF) in dry THF (1.5 mL $mmol^{-1}$) was added aldehyde **S4** (1.00 eq) slowly and the reaction mixture was stirred at room temperature overnight. The reaction mixture was quenched by adding freshly distilled acetaldehyde (50 µL $mmol^{-1}$). Solvents were removed and the residue was subjected to high vacuum for 1 h. The residue was dissolved in Et_2O (2 mL $mmol^{-1}$) and the resulting solution

was cooled to 0 °C. After addition of ethanolamine (1.10 eq), stirring was continued for 10 min until a white precipitate was formed. The precipitate was filtered off and washed three times with Et_2O. The combined organic layers were washed with H_2O and brine, followed by drying with $MgSO_4$. The solvent was removed under reduce pressure and the residue was purified by column chromatography (pentane/Et_2O, 3:1) to yield the alcohols **S5** as colorless oils. The enantiomeric purity was checked by conversion of a small sample (ca. 1 mg) into the Mosher ester with (*R*)-(+)-Mosher chloride (1 mg) and pyridine (1 µL) in $CDCl_3$ (500 µL). After a reaction time of 1 h at 60 °C, the products were directly analysed by ^{1}H-NMR (Figure S235).

(*R*)-S5. Yield: 281 mg (0.96 mmol, 80%, 94% ee). TLC (petroleum/EtOAc, 1:1): R_f = 0.48. GC (HP5-MS): *I* = 2204. ^{1}H-NMR (C_6D_6, 500 MHz): 5.38 (br d, $^3J_{H,H}$ = 5.7, 1H), 5.29 (thex, $^3J_{H,H}$ = 6.8, $^4J_{H,H}$ = 1.2, 1H), 5.24 (thept, $^3J_{H,H}$ = 6.9, $^4J_{H,H}$ = 1.4, 1H), 4.89 (m, 1H), 4.86 (m, 1H), 3.97 (br dd, $^1J_{H,C}$ = 139.63, $^3J_{H,H}$ = 6.5, 1H), 2.19 (m, 4H), 2.10 (m, 4H), 1.98 (t, $^3J_{H,H}$ = 7.7, 2H), 1.91 (t, $^3J_{H,H}$ = 7.7, 2H), 1.68 (m, 3H), 1.60 (br s, 3H), 1.56 (br s, 3H), 1.53 (m, 2H), 1.45 (br s, 3H), 0.58 (br s, OH) ppm. ^{13}C-NMR (C_6D_6, 125 MHz): 149.44 (C_q), 138.23 (C_q), 135.27 (C_q), 131.20 (C_q), 124.95 (d, $^1J_{C,C}$ = 47.5, CH), 124.93 (CH), 124.68 (CH), 109.59 (CH_2), 59.02 (t, $^1J_{C,D}$ = 21.5, $^{13}C^2HH$), 40.21 (CH_2), 39.48 (CH_2), 36.50 (CH_2), 36.09 (CH_2), 27.22 (CH_2), 26.84 (CH_2), 26.21 (CH_2), 25.88 (CH_3), 17.77 (CH_3), 16.15 (CH_3), 16.09 (d, $^3J_{C,C}$ = 4.3, CH_3) ppm. IR (diamond ATR): ṽ = 3336 (w), 2964 (m), 2923 (s), 2857 (m), 1643 (w), 1440 (m), 1411 (m), 1383 (m), 1295 (w), 1164 (w), 1036 (w), 974 (w), 887 (s), 801 (w), 675 (w) cm^{-1}. HRMS (EI): $[M]^+$ calcd. for $^{13}CC_{19}H_{33}D_1O^+$ *m*/*z* 292.2609; found *m*/*z* 292.2608.

(*S*)-S5. Yield: 326 mg (1.12 mmol, 92%, 96% ee). TLC (petroleum/EtOAc, 1:1): R_f = 0.48. GC (HP5-MS): *I* = 2204. Spectroscopic data were the same as for (*R*)-**S4**.

Synthesis of (*R*)- and (*S*)-(1-^{13}C,1-^{2}H)-iso-GGPP trisammonium salt

A solution of bis-triethylammonium phosphate (TEAP) was prepared by adding a solution of H_3PO_4 (0.75 mL) in MeCN (2.8 mL) to a mixture of NEt_3 (3.3 mL) and MeCN (3.0 mL). Alcohols (*R*)- and (*S*)-**S5** (1.00 eq) were dissolved in CCl_3CN (2.5 mL $mmol^{-1}$) and TEAP solution (5 mL $mmol^{-1}$) was added dropwise at room temperature within 1 min. After stirring for 2 min another portion of TEAP solution (5 mL $mmol^{-1}$) was added dropwise. Stirring was continued for 5 min (not longer to suppress formation of triphosphate). The reaction mixture was directly subjected to silica gel chromatography (^{i}PrOH/25% NH_3/H_2O=6:2.5:0.5). Fractions containing the product were lyophilised to yield the diphosphates as white solids.

(*S*)-S6. Yield: 140 mg (0.27 mmol, 26%). ^{1}H-NMR (D_2O, 500 MHz): 5.42 (d, $^3J_{H,H}$ = 6.9, 1H), 5.11 (t, $^3J_{H,H}$ = 6.8, 1H), 5.08 (t, $^3J_{H,H}$ = 6.7, 1H), 4.75 (br s, 1H), 4.72 (br s, 1H), 4.42 (dt, $^1J_{H,C}$ = 146.8, $^3J_{H,H}$ = 6.9, 1H), 2.03 (m, 12H), 1.70 (br s, 3H), 1.65 (br s, 3H), 1.59 (br s, 3H), 1.57 (br s, 3H), 1.55 (quin, $^3J_{H,H}$ = 7.3, 2H) ppm. ^{13}C-NMR (D_2O, 125 MHz): 149.13 (C_q), 142.07 (C_q), 134.79 (C_q), 130.72 (C_q), 124.55 (CH), 124.26 (CH), 120.01 (dd, $^1J_{C,C}$ = 49.5, $^3J_{P,C}$ = 9.8 Hz, CH), 108.97 (CH_2), 62.40 (br t, $^1J_{C,D}$ = 19.2, ^{13}CDO), 39.75 (CH_2), 39.26 (d, $^3J_{C,C}$ = 4.8, CH_2), 36.24 (CH_2), 35.72 (CH_2), 26.75 (CH_2), 26.30 (CH_2), 25.75 (CH_2), 25.44 (CH_3), 17.42 (CH_3), 15.90 (d, $^3J_{C,C}$ = 4.3, CH_3), 15.84 (CH_3) ppm. ^{31}P-NMR (D_2O, 203 MHz): –8.9 (d, $^2J_{P,P}$ = 19.1), –10.5 (dd, $^2J_{C,P}$ = 4.0, $^2J_{P,P}$ = 19.5) ppm. HRMS (EI): $[M]^-$ calcd. for $^{13}CC_{19}H_{34}D_1O_7P_2^-$ *m*/*z* 451.1965; found *m*/*z* 451.1968.

(*R*)-S5. Yield: 65 mg (0.13 mmol, 14%). Spectroscopic data were the same as for (*S*)-**S5**.

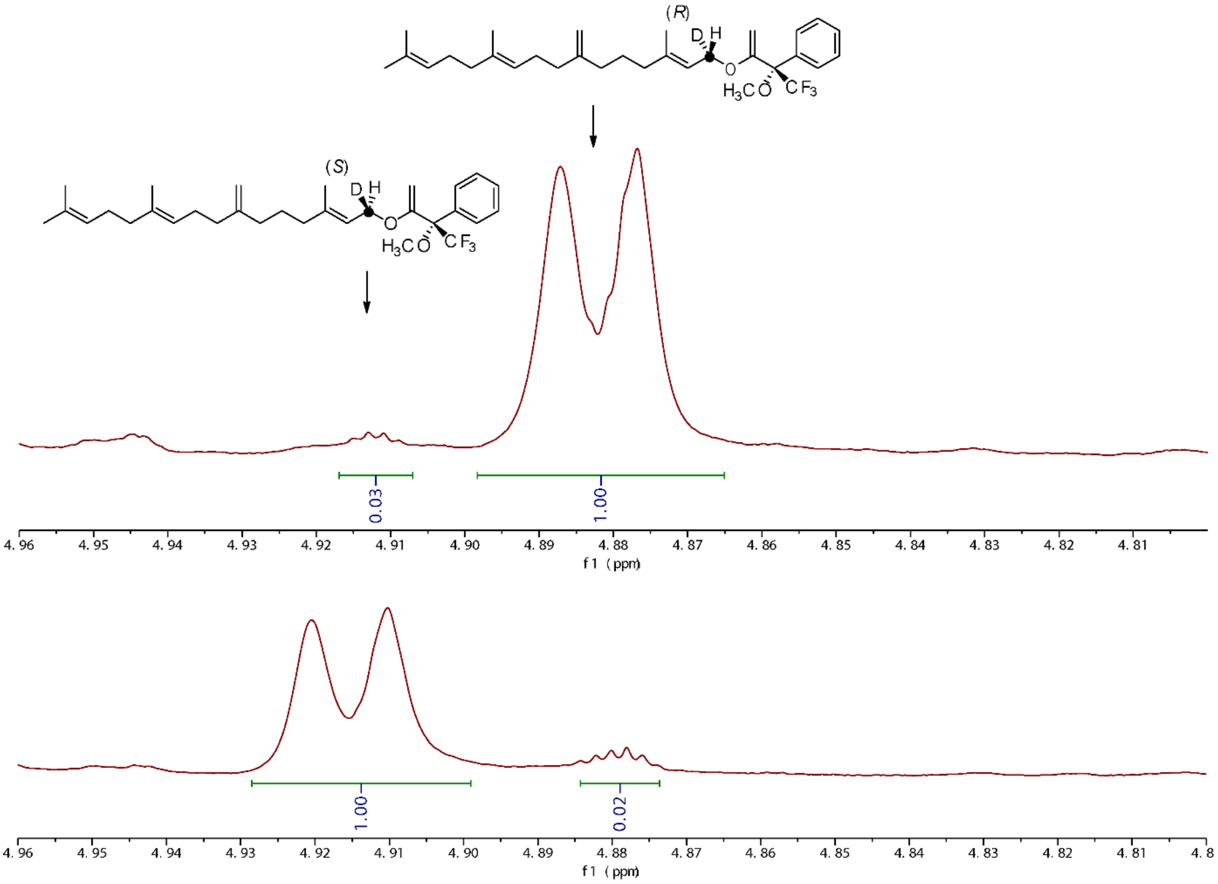

Figure S235. ^{1}H-NMR spectra of Mosher esters obtained from alcohols (*R*)-**S5** (top) and (*S*)-**S5** (bottom).

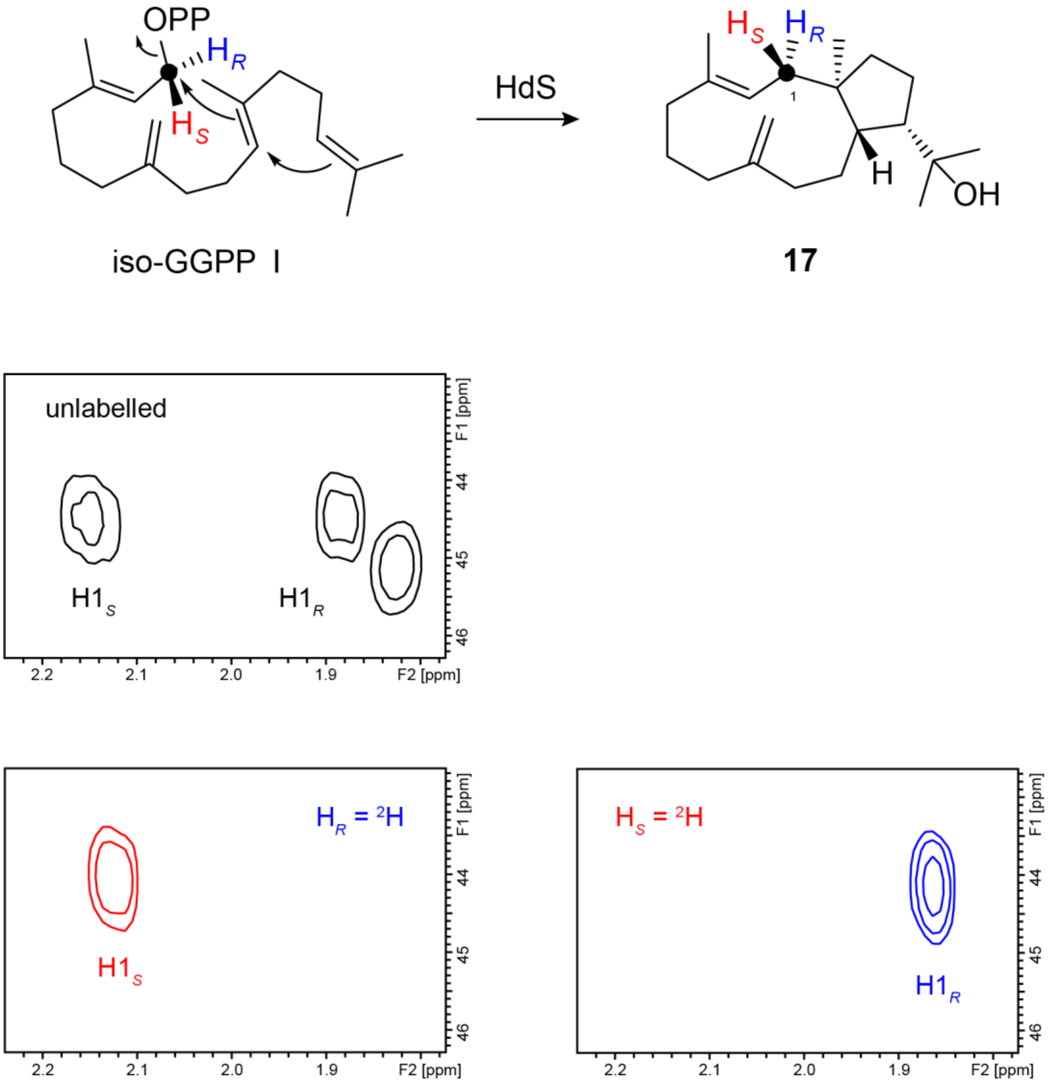

Figure S236. The absolute configuration of **17**. Conversion of (*R*)- and (*S*)-(1-^{13}C,1-^{2}H)-iso-GGPP with HdS and HSQC analysis of the products, revealing the absolute configuration for **17** as shown.

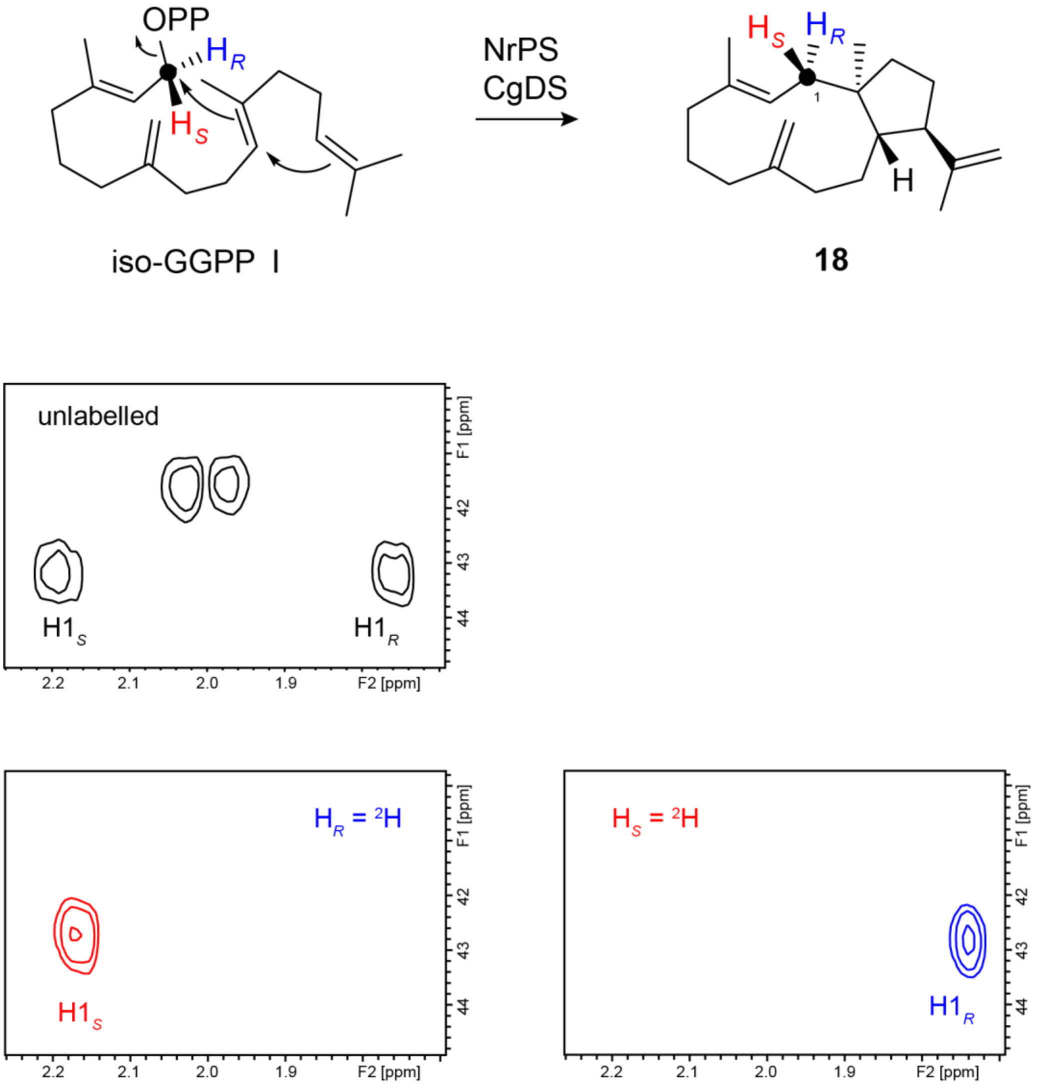

Figure S237. The absolute configuration of **18**. Conversion of (*R*)- and (*S*)-(1-^{13}C,1-^{2}H)-iso-GGPP with NrPS and HSQC analysis of the products, revealing the absolute configuration for **18** as shown. Analogous experiments with CgDS showed the same result.

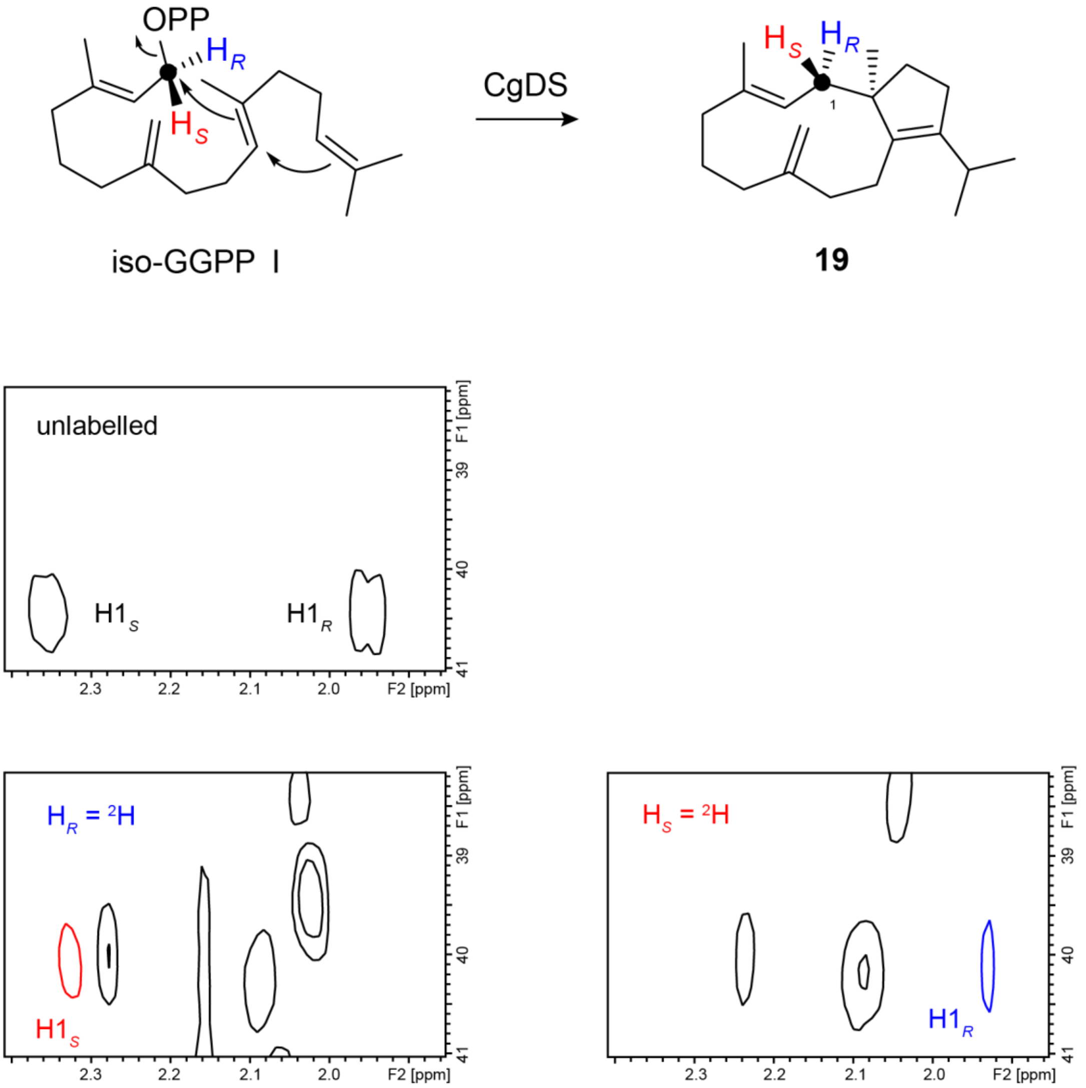

Figure S238. The absolute configuration of **19**. Conversion of (*R*)- and (*S*)-(1-^{13}C,1-^{2}H)-iso-GGPP with CgDS and HSQC analysis of the products, revealing the absolute configuration for **19** as shown.

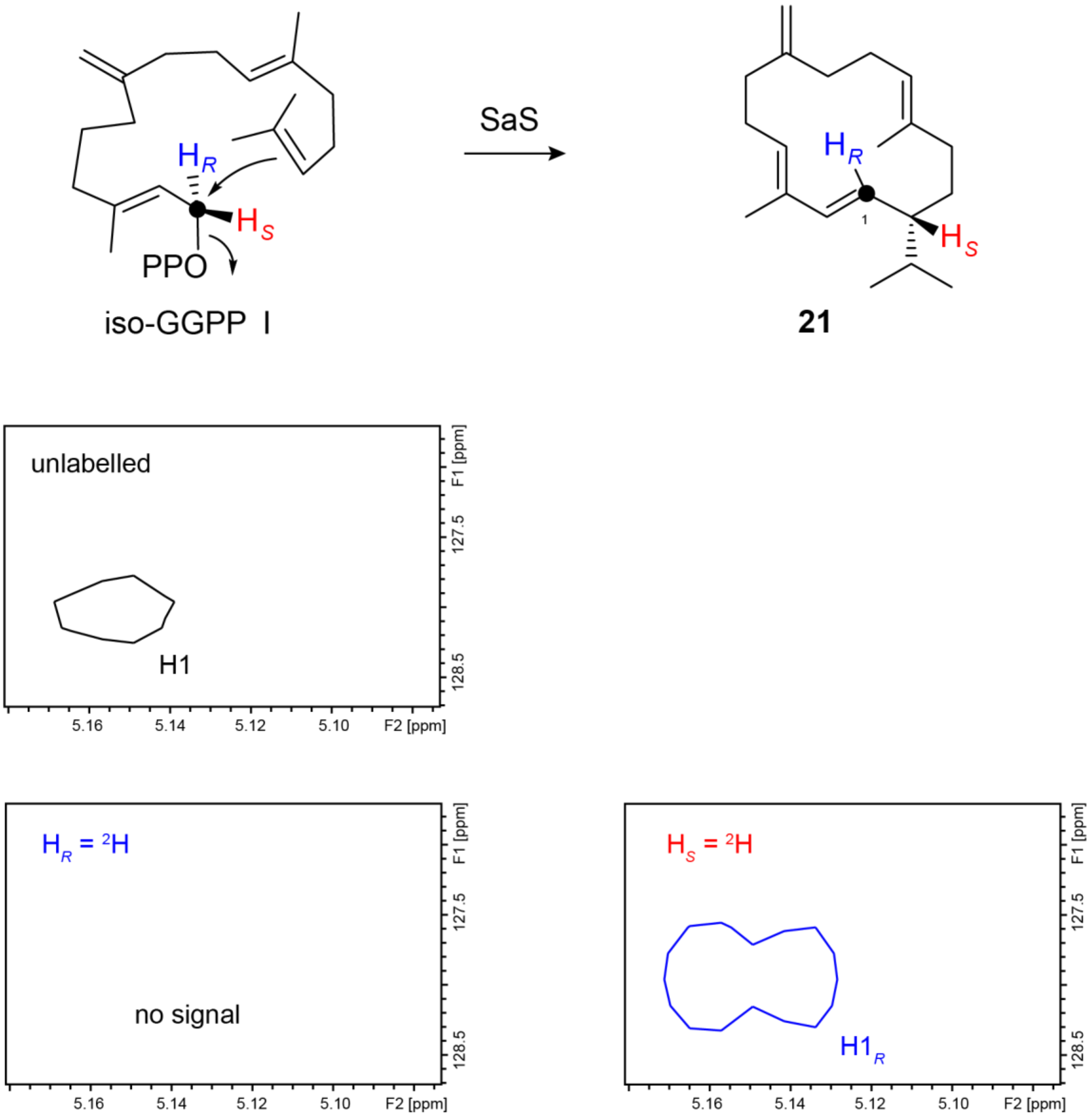

Figure S239. The absolute configuration of **21**. Conversion of (*R*)- and (*S*)-(1-^{13}C,1-^{2}H)-iso-GGPP with SaS and HSQC analysis of the products. No signal is observed from (*R*)-(1-^{13}C,1-^{2}H)-iso-GGPP, showing that the 1-*pro-R* hydrogen remains bound to C1, while (*S*)-(1-^{13}C,1-^{2}H)-iso-GGPP yields a strong signal, showing that the 1-*pro-S* hydrogen migrates into another position. For spiroalbatene biosynthesis from GGPP by SaS the same observations were made, suggesting that the absolute configuration of **21** is analogous to that of spiroalbatene and the natural pathway intermediates (Scheme S4).

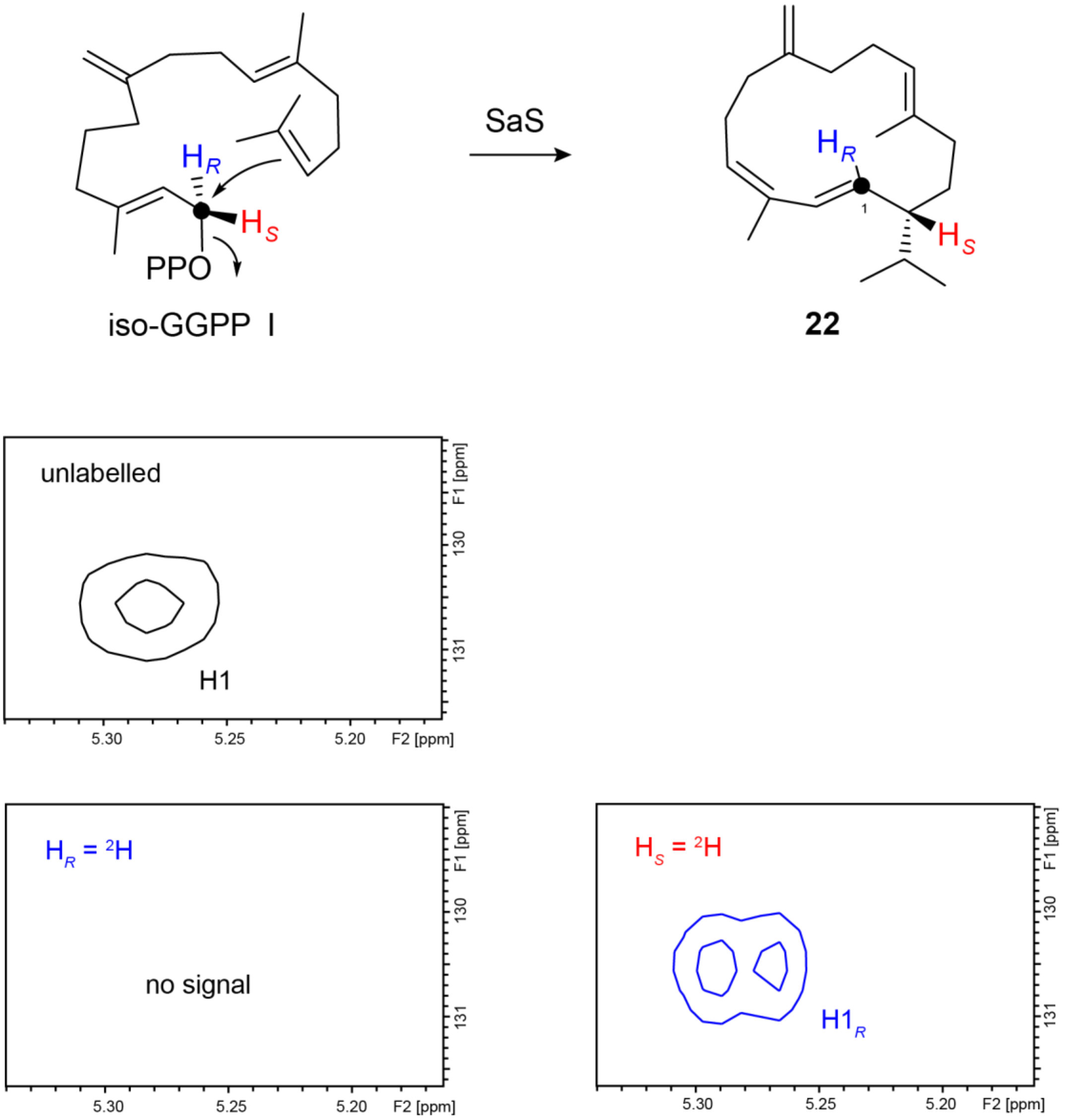

Figure S240. The absolute configuration of **22**. Conversion of (*R*)- and (*S*)-(1-^{13}C,1-^{2}H)-iso-GGPP with SaS and HSQC analysis of the products. No signal is observed from (*R*)-(1-^{13}C,1-^{2}H)-iso-GGPP, showing that the 1-*pro*-*R* hydrogen remains bound to C1, while (*S*)-(1-^{13}C,1-^{2}H)-iso-GGPP yields a strong signal, showing that the 1-*pro*-*S* hydrogen migrates into another position. For spiroalbatene biosynthesis from GGPP by SaS the same observations were made, suggesting that the absolute configuration of **22** is analogous to that of spiroalbatene and the natural pathway intermediates (Scheme S4).

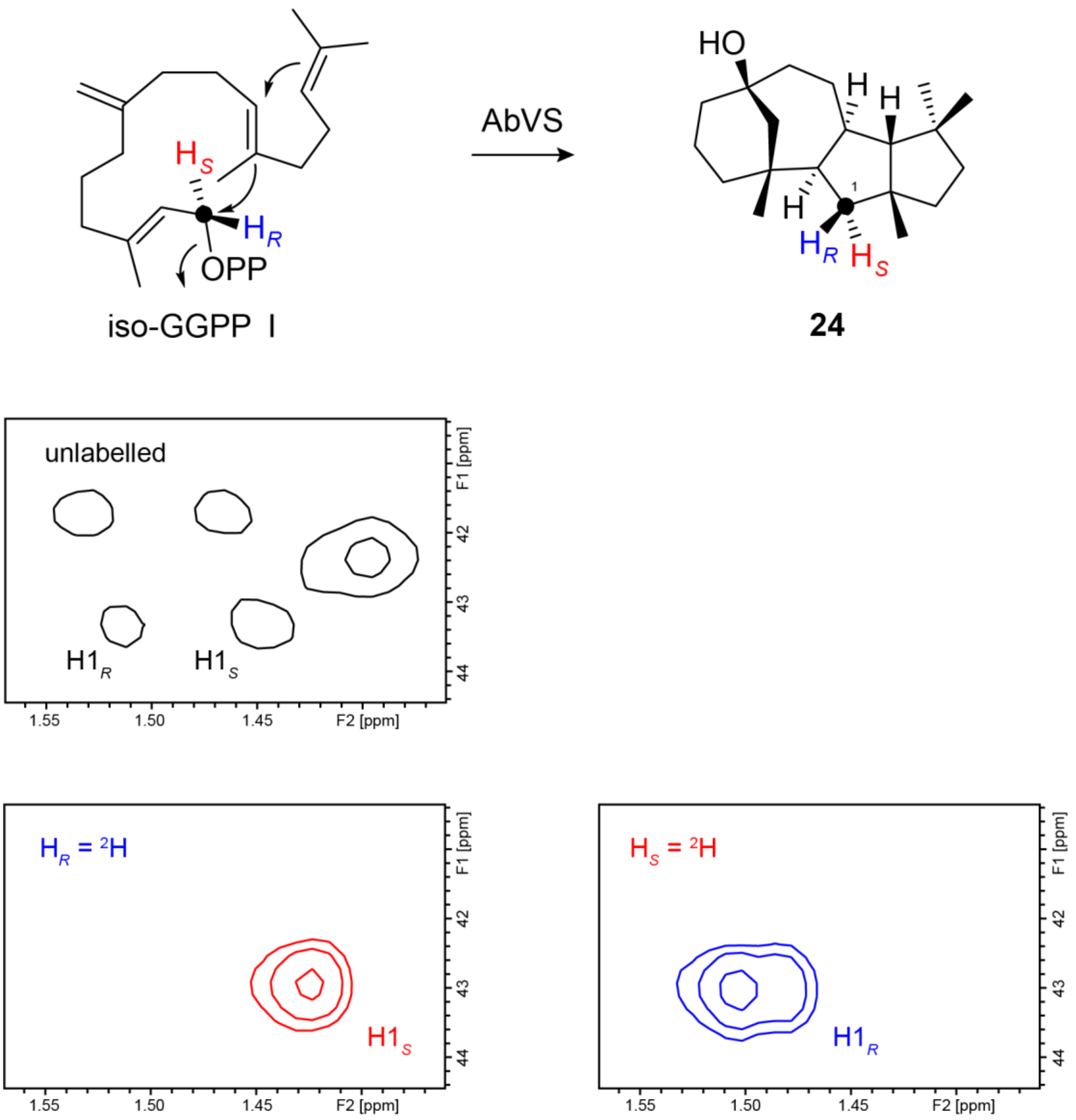

Figure S241. The absolute configuration of **24**. Conversion of (*R*)- and (*S*)-(1-^{13}C,1-^{2}H)-iso-GGPP with AbVS and HSQC analysis of the products, revealing the absolute configuration for **24** as shown.

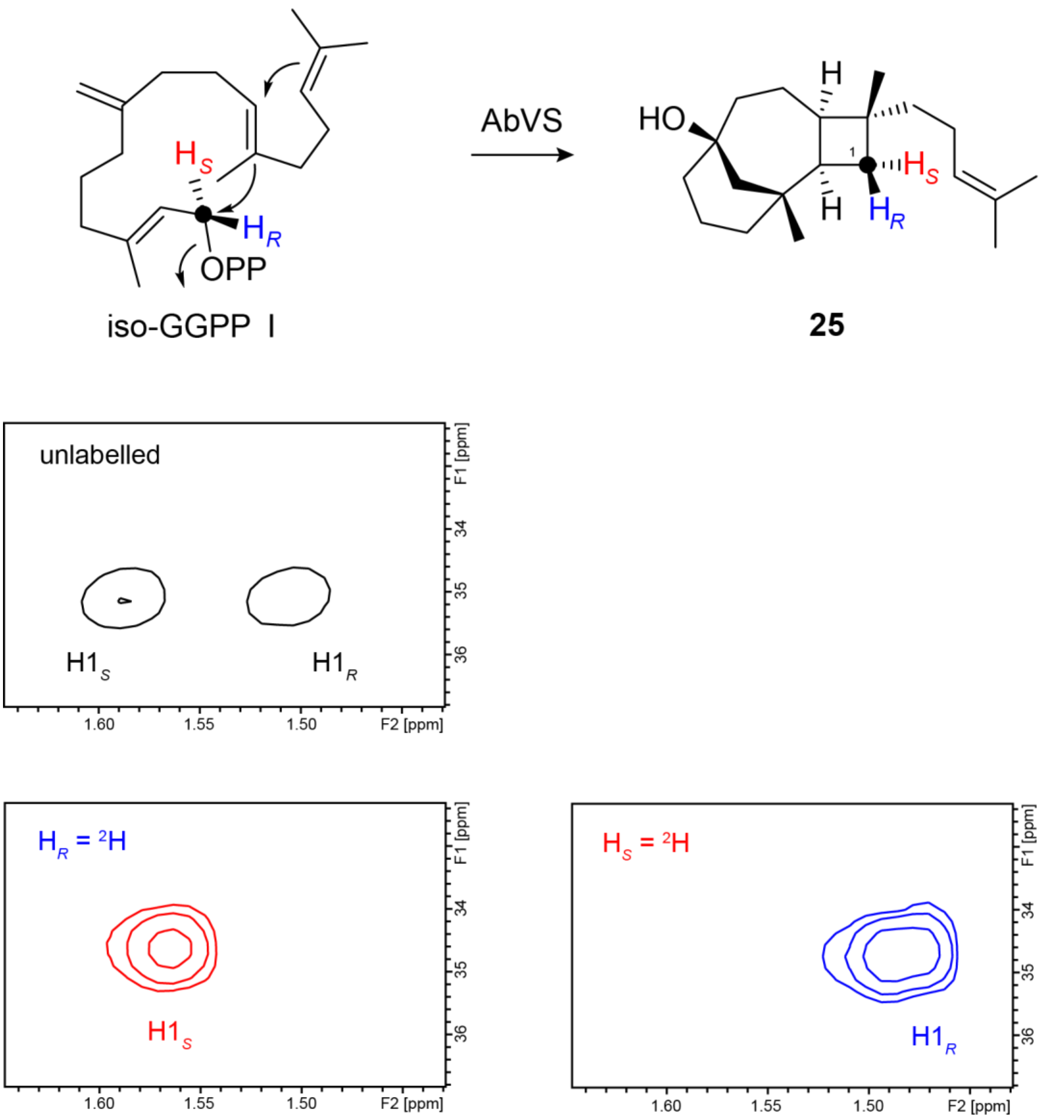

Figure S242. The absolute configuration of **25**. Conversion of (*R*)- and (*S*)-(1-^{13}C,1-^{2}H)-iso-GGPP with AbVS and HSQC analysis of the products, revealing the absolute configuration for **25** as shown.

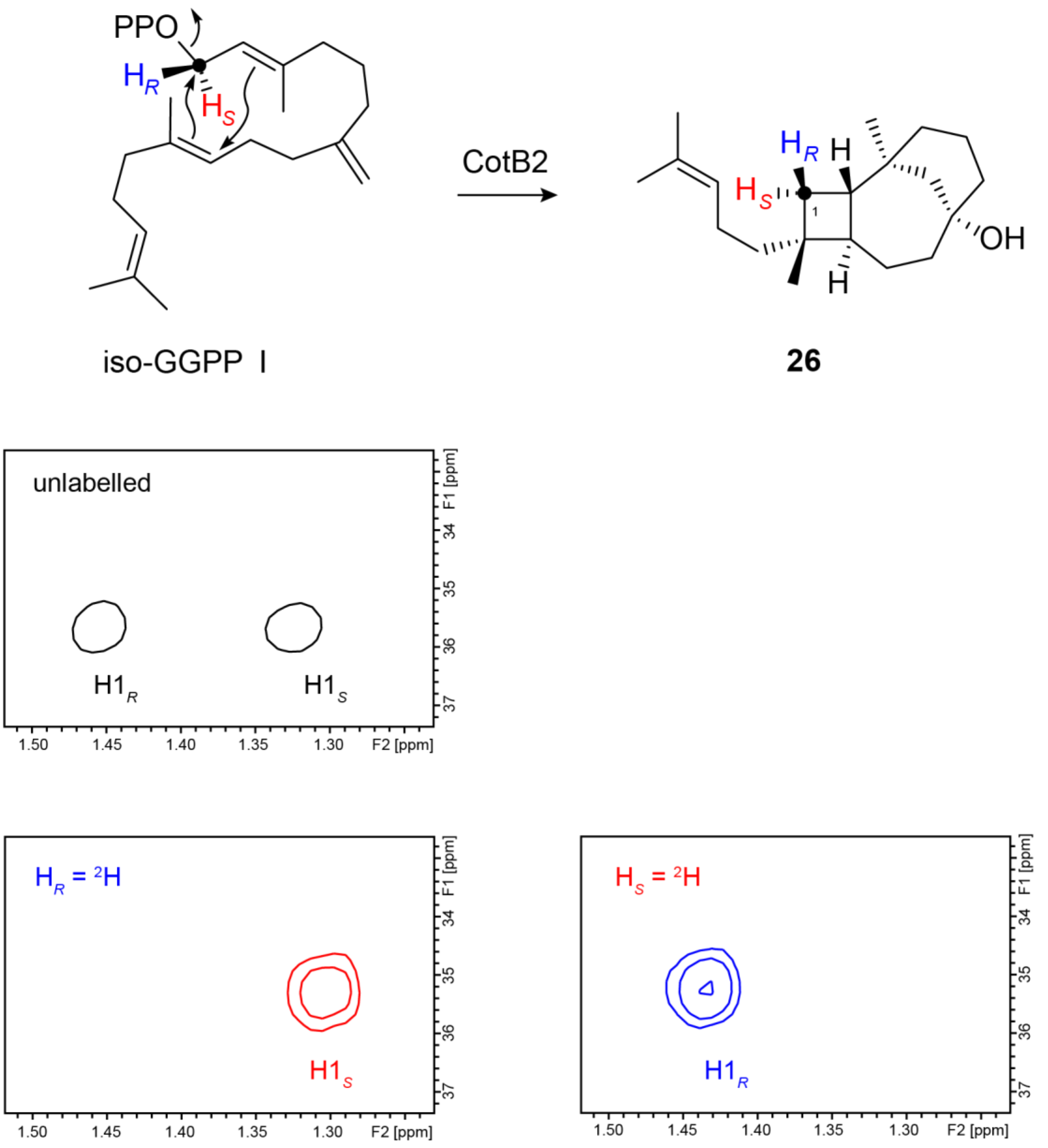

Figure S243. The absolute configuration of **26**. Conversion of (*R*)- and (*S*)-(1-^{13}C,1-^{2}H)-iso-GGPP with CotB2 and HSQC analysis of the products, revealing the absolute configuration for **26** as shown.

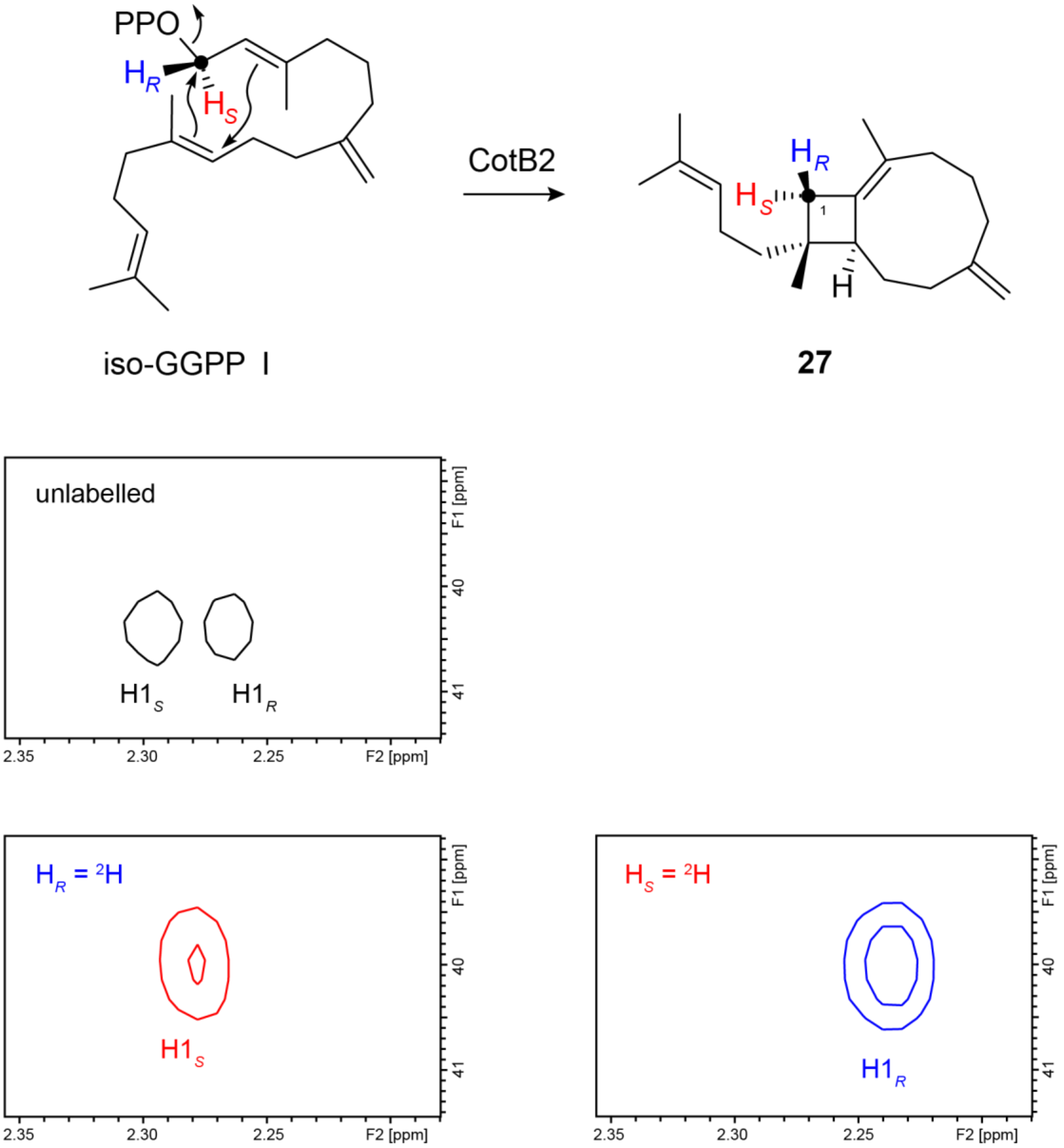

Figure S244. The absolute configuration of **27**. Conversion of (*R*)- and (*S*)-(1-^{13}C,1-^{2}H)-iso-GGPP with CotB2 and HSQC analysis of the products, revealing the absolute configuration for **27** as shown.

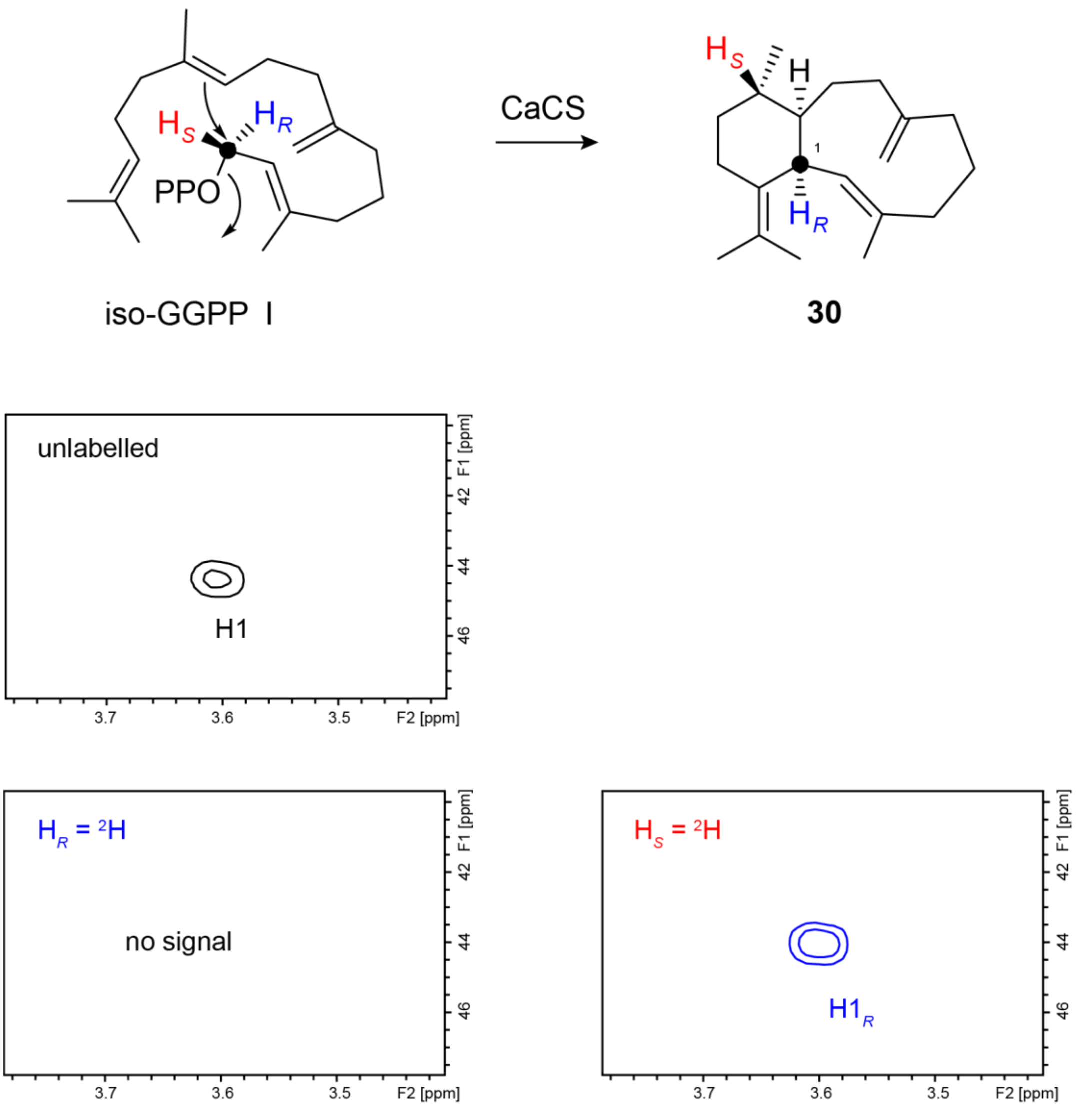

Figure S245. The absolute configuration of **30**. Conversion of (*R*)- and (*S*)-(1-^{13}C,1-^{2}H)-iso-GGPP with CaCS and HSQC analysis of the products. The same hydrogen as in the biosynthesis of the natural CaCS product catenul-14-en-6-ol (1-*pro*-*S*) migrates away from C1, suggesting an analogous absolute configuration for **30** as shown.

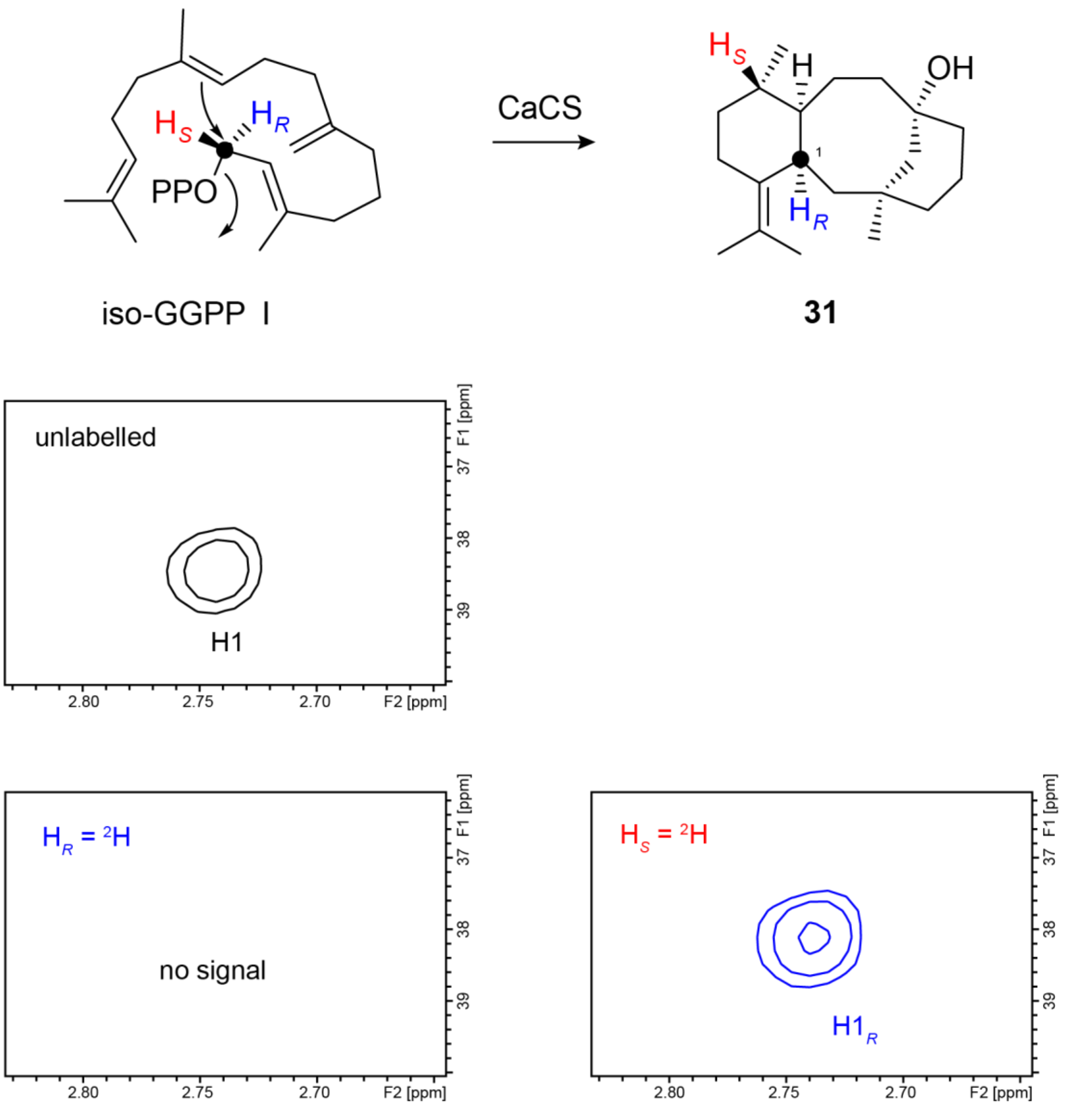

Figure S246. The absolute configuration of **31**. Conversion of (*R*)- and (*S*)-(1-^{13}C,1-^{2}H)-iso-GGPP with CaCS and HSQC analysis of the products. The same hydrogen as in the biosynthesis of the natural CaCS product catenul-14-en-6-ol (1-*pro*-*S*) migrates away from C1, suggesting an analogous absolute configuration for **31** as shown.

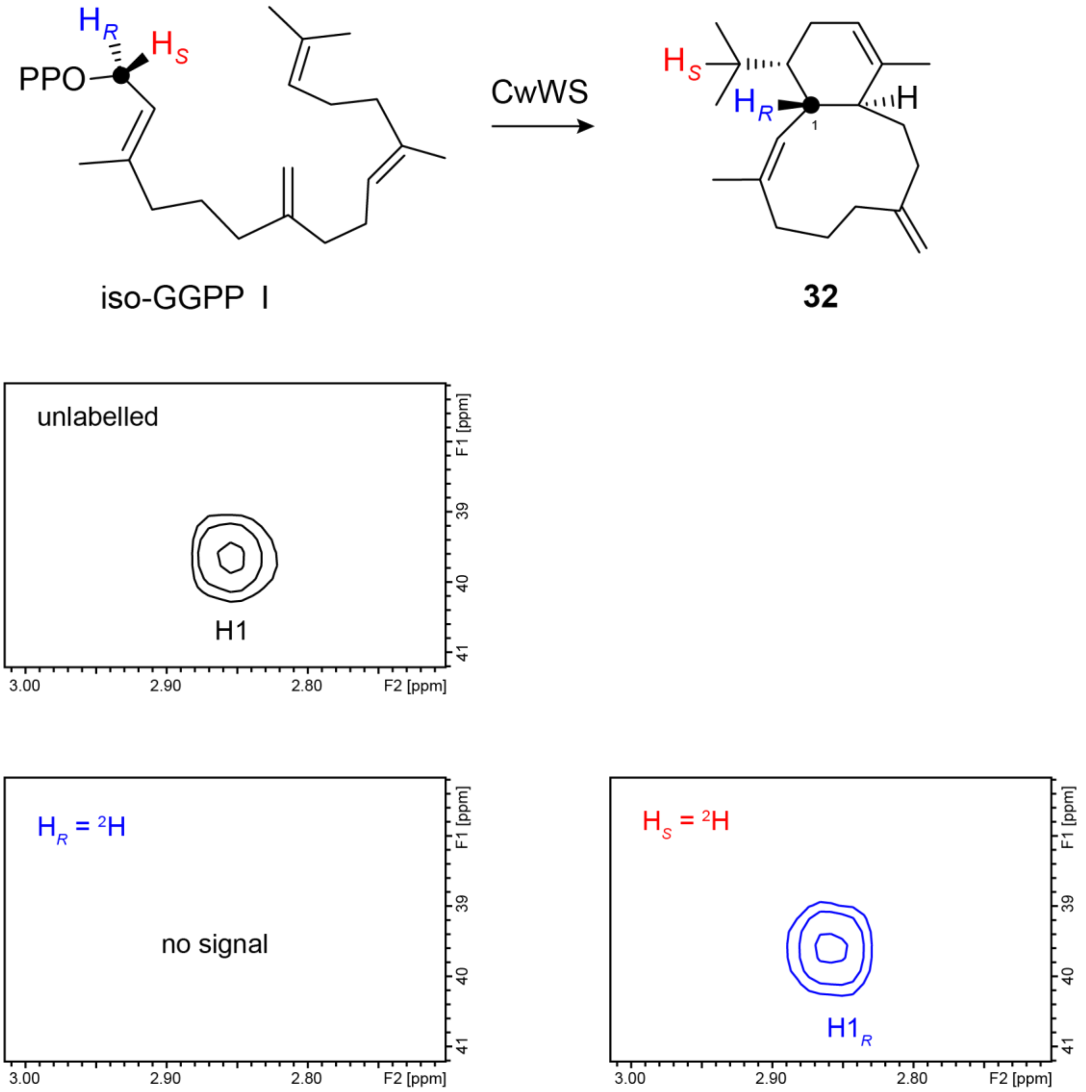

Figure S247. The absolute configuration of **32**. Conversion of (*R*)- and (*S*)-(1-^{13}C,1-^{2}H)-iso-GGPP with CwWS and HSQC analysis of the products. The same hydrogen as in the biosynthesis of the natural CwWS product wanju-2,6-diene (1-*pro*-*S*) migrates away from C1, suggesting an analogous absolute configuration for **32** as shown.

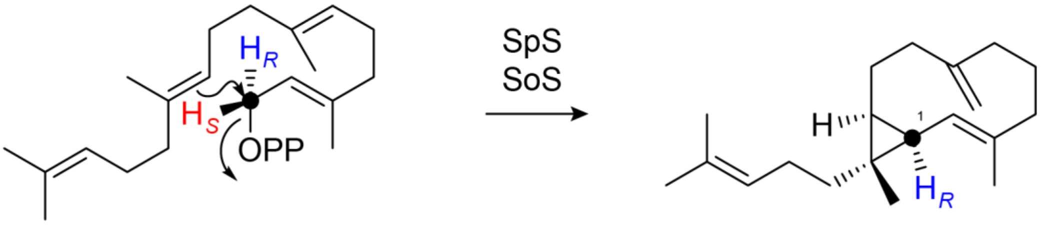

iso-GGPP I

34

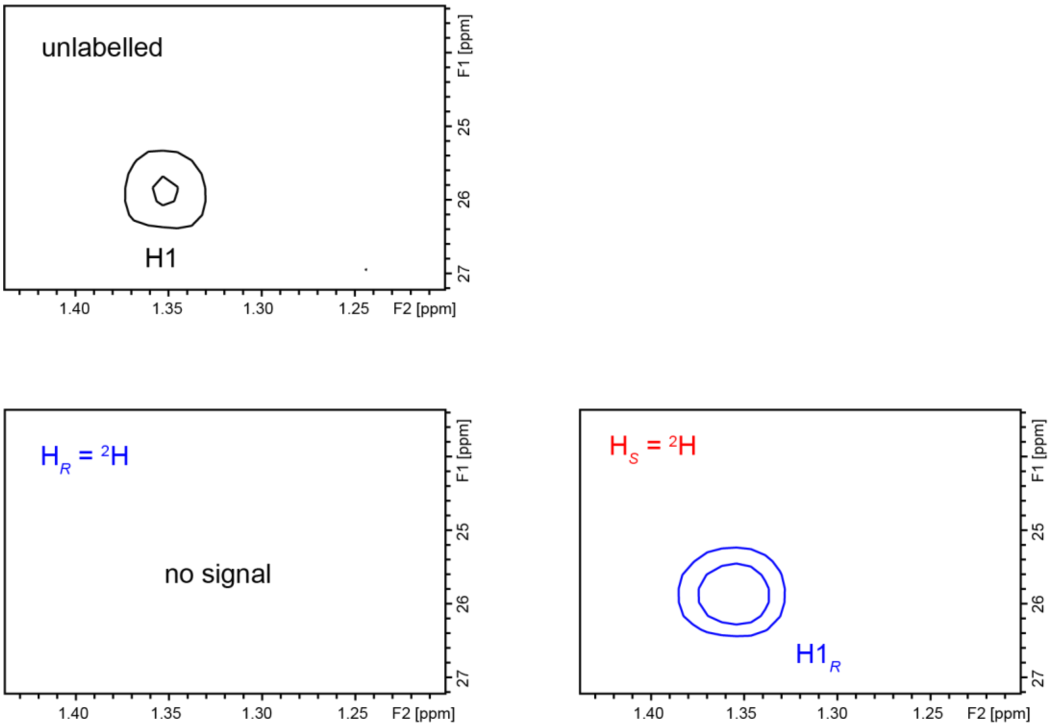

Figure S248. The absolute configuration of **34**. Conversion of (*R*)- and (*S*)-(1-^{13}C,1-^{2}H)-iso-GGPP with SpS and HSQC analysis of the products, revealing the absolute configuration for **34** as shown. Analogous experiments with SoS showed the same result.

References

[1] G. R. Fulmer, A. J. M. Miller, N. H. Sherden, H. E. Gottlieb, A. Nudelman, B. M. Stoltz, J. E. Bercaw, K. I. Goldberg, *Organometallics* **2010**, *29*, 2176-2179.

[2] J. S. Dickschat, K. A. K. Pahirulzaman, P. Rabe, T. A. Klapschinski, *ChemBioChem* **2014**, *15*, 810-814

[3] R. D. Giets, R. H. Schiestl, *Nat. Protoc*. **2007**, *2*, 31.

[4] M. M. Bradford, *Anal. Biochem.* **1976**, *72*, 248.

[5] L. Lauterbach, J. Rinkel, J. S. Dickschat, *Angew. Chem.* **2018**, *130*, 8412-8415; *Angew. Chem. Int. Ed.* **2018**, *57*, 8280-8283.

[6] P. Rabe, J. Rinkel, E. Dolja, T. Schmitz, B. Nubbemeyer, T. H. Luu, J. S. Dickschat, *Angew. Chem.* **2017**, *129*, 2820-2823; *Angew. Chem. Int. Ed.* **2017**, *56*, 2776-2779.

[7] J. Rinkel, L. Lauterbach, J. S. Dickschat, *Angew. Chem.* **2017**, *129*, 16603-16607; *Angew. Chem. Int. Ed.* **2017**, *56*, 16385-16389.

[8] J. Rinkel, L. Lauterbach, J. S. Dickschat, *Angew. Chem.* **2019**, *131*, 461-465; *Angew. Chem. Int. Ed.* **2019**, *58*, 452-455.

[9] J. Rinkel, L. Lauterbach, P. Rabe, J. S. Dickschat, *Angew. Chem.* **2018**, *130*, 3292-3296; *Angew. Chem. Int. Ed.* **2018**, *57*, 3238-3241.

[10] J. Rinkel, S. T. Steiner, G. Bian, R. Chen, T. Liu, J. S. Dickschat, *ChemBioChem* **2020**, *21*, 486-491.

[11] J. Rinkel, S. T. Steiner, J. S. Dickschat, *Angew. Chem.* **2019**, *131*, 9328-9332; *Angew. Chem. Int. Ed.* **2019**, *58*, 9230-9233.

[12] J. Rinkel, P. Rabe, X. Chen, T. G. Köllner, F. Chen, J. S. Dickschat, *Chem. Eur. J.* **2017**, *23*, 10501-10505.

[13] J. S. Dickschat, J. Rinkel, P. Rabe, A. Beyraghdar Kashkooli, H. J. Bouwmeester, *Beilstein J. Org. Chem.* **2017**, *13*, 1770-1780.

[14] G. Bian, J. Rinkel, Z. Wang, L. Lauterbach, A. Hou, Y. Yuan, Z. Deng, T. Liu, J. S. Dickschat, *Angew. Chem.* **2018**, *130*, 16113-16117; *Angew. Chem. Int. Ed.* **2018**, *57*, 15887-15890.

[15] a) S.-Y. Kim, P. Zhao, M. Igarashi, R. Sawa, T. Tomita, M. Nishiyama, T. Kuzuyama, *Chem. Biol.* **2009**, *16*, 736-743; b) A. Meguro, Y. Motoyoshi, K. Teramoto, S. Ueda, Y. Totsuka, Y. Ando, T. Tomita, S.-Y. Kim, T. Kimura, M. Igarashi, R. Sawa, T. Shinada, M. Nishiyama, T. Kuzuyama, *Angew. Chem.* **2015**, *127*, 4427-4430; *Angew. Chem. Int. Ed.* **2015**, *54*, 4353-4356.

[16] G. Li, Y.-W. Guo, J. S. Dickschat, *Angew. Chem.* **2021**, *133*, 1510-1514; *Angew. Chem. Int. Ed.* **2021**, *60*, 1488-1492.

[17] L. Lauterbach, B. Goldfuss, J. S. Dickschat, *Angew. Chem.* **2020**, *132*, 12041-12045; *Angew. Chem. Int. Ed.* **2020**, *59*, 11943-11947.

[18] H. Li, J. S. Dickschat, *Org. Chem. Front.* **2022**, *9*, 795-801.
